# Supplementary material for: Catalytic asymmetric constructions of nitrogen, boron and carbon continuous stereogenic centers
Source: Nat Commun. 2025 Nov 12;16:9955. doi: 10.1038/s41467-025-64905-x (PMC12612116; doi:10.1038/s41467-025-64905-x)
Supplement: Supplementary file 1 — Supplementary Information [file 41467_2025_64905_MOESM1_ESM.pdf]

Supplementary Information

for

Catalytic Asymmetric Constructions of Nitrogen, Boron & Carbon Continuous Stereogenic Centers

Guan Zhang,[1,2] Junyi Jia,[2] Xuzhao Du,[3] Bofan Feng,[2] Kai Yang,[2] Peiyuan Yu,[3] and Qiuling Song[2,4,5]*

[1]College of Chemistry and Materials Science, Fujian Normal University, Fuzhou, Fujian, 350007, China

[2]Key Laboratory of Molecule Synthesis and Function Discovery, Fujian Province University, College of Chemistry at Fuzhou University, Fuzhou, Fujian, 350108, China

[3]Department of Chemistry and Shenzhen Grubbs Institute, Southern University of Science and Technology, Shenzhen, Guangdong, 518055, China

[4]State Key Laboratory of Coordination Chemistry, School of Chemistry and Chemical Engineering Nanjing University, Nanjing, 210093, China

[5]School of Chemistry and Chemical Engineering, Henan Normal University, Xinxiang, Henan, 453007, China

*Corresponding author, Email: qsong@fzu.edu.cn

Contents

1 Experimental studies 2

1.1 General information 2

1.2 General procedure A for preparation of amine boranes 2

1.3 General procedure B for preparation of amine boranes 3

1.4 General procedure C for preparation of diazo compounds 3

1.5 General procedure D for continuous stereogenic centers 3

1.6 General procedure E for continuous stereogenic centers 4

1.7 General procedure F for kinetic resolution 4

1.8 Complementary experiments 4

1.9 Optimization of reaction conditions 5

2 Analysis data of compounds **1**, **2** and **3** 8

3 Mechanistic studies 79

4 The application of the chiral amine borane 82

5 The synthetic tranformations of the amine borane product 85

6 Crystal structure of compound **3o** 88

7 Computational study 89

8 NMR spectra 94

9 Supplementary references 219

1 Experimental studies

1.1 General information

All experiments were conducted with a schlenk tube. Flash column chromatography was performed over silica gel (200-300 mesh). ^{1}H NMR and ^{13}C NMR spectra were recorded at ambient temperature using Bruker AVANCE III 500M spectrometers, Bruker 400M spectrometers, JEOL 500M spectrometers, chemical shifts (in ppm) were referenced to $CDCl_3$ (δ = 7.26 ppm), acetone-d_6 (δ = 2.05 ppm) as internal standards. ^{13}C NMR spectra were obtained by using the same NMR spectrometers and were calibrated with $CDCl_3$ (δ = 77.0 ppm). Data for ^{1}H NMR are recorded as following abbreviations: multiplicity (s = singlet, d = doublet, t = triplet, q = quarter, m = multiplet), coupling constant (J, Hz). High resolution mass spectroscopy (HRMS) analyses were performed at an Exactive Plus (Thermo Scientific) or Agilent Mass Spectrometer. Unless otherwise noted, materials obtained from commercial suppliers were used without further purification.

1.2 General procedure A for preparation of amine boranes

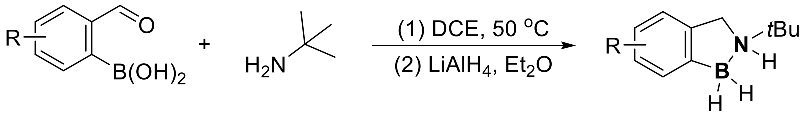

Step 1: The synthesis of the following boranes basically followed this process, and some of the uncommercialized 2-formylphenylboric acid were prepared by coupling *o*-bromobenzaldehyde with bis(pinacolato)diboron to prepare 2-formylphenylboric acid derivatives. A round-bottomed flask was charged with 2-formylbenzeneboronic acid (10 mmol), tert-butylamine (3 equiv) and 1,2-dichlorethane under an air, and then heated up to 50 °C. The reaction was kept in a closed system, and after 6 h, the solvent was removed by vacuum distillation, and the obtained crude product was directly thrown into the next step.

Step 2: The crude product from **Step 1** was dissolved in Et_2O (20 mL), added $LiAlH_4$ (3 equiv) at 0 °C, the reaction was allowed to stir at 0 °C for 4 hours. Then, this reaction was quenched by H_2SO_4 (0.5 M), extracted with ethyl acetate (20 mL × 3) and dried over Na_2SO_4. After removal of the solvent, the crude reaction mixture was purified on short column chromatography (petroleum ether and ethyl acetate) to afford the amine boranes.

1.3 General procedure B for preparation of amine boranes

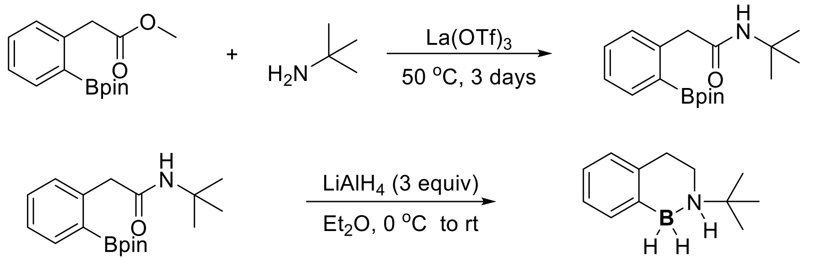

Step 1: A round-bottomed flask was charged with 2-(ethoxycarbonylmethyl)phenylboronic acid pinacol ester (1.49 g, 10 mmol) and tert-butylamine (3.65 g, 50 mmol, 5 equiv) under an air, and then heated up to 50 °C. The reaction was kept in a closed system, and after 3 days, this reaction was quenched by water, extracted with ethyl acetate (20 mL × 3) and dried over Na_2SO_4, and the obtained crude product was directly thrown into the next step.

Step 2: This step was the same as the **step 2** of procedure A.

1.4 General procedure C for preparation of diazo compounds

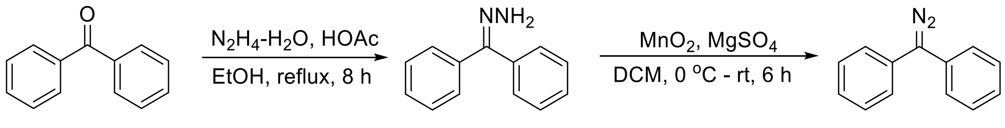

2a

Step 1: By analogy to a modified literature procedure[1], hydrazine monohydrate (0.95 g, 20 mmol, 4 equiv, 95%) was added to benzophenone (0.91 g, 5 mmol, 1 equiv) in ethanol (20 mL). Then HOAc (28.6 uL, 10 mol %) was added and the mixture was heated at reflux for 8 h. After cooling to room temperature, and removal of the solvent to afford the desired products.

Step 2: Benzophenone hydrazone, anhydrous $MgSO_4$ (2.41 g, 4 equiv), and 40 mL DCM was cooled to 0 °C. To this rapidly stirring mixture was added activated MnO_2 (1.74 g, 4 equiv) in one portion. The reaction mixture was warmed to room temperature and kept stirring for 6 h. Then filtered, filtrate was collected. After removal of the solvent under reduced pressure, the residue was purified by Al_2O_3 base with PE/Et_3N = 20:1 as eluent to afford **2a** as a purple solid (824 mg, 85% yield), which was kept at 0 - 8 °C.

1.5 General procedure D for continuous stereogenic centers

In air, a 25 mL schlenk tube was charged with **1** (0.3 mmol, 3 equiv), **2** (0.1 mmol, 1 equiv), CuTc (1.9 mg, 10 mol%), **L1** (9.5 mg, 11 mol%), additive (7.2 mg, 10 mol%). The tube was evacuated and filled with argon for three cycles. Then, 1 mL of Dichloromethane was added under argon. The reaction was allowed to stir at -70 °C for 48 hours. Upon completion, proper amount of silica gel was added to the reaction mixture. After removal of the solvent, the crude reaction mixture was purified on silica gel

(petroleum ether and ethyl acetate) to afford the desired products.

1.6 General procedure E for continuous stereogenic centers

In air, a 25 mL schlenk tube was charged with **1** (0.3 mmol, 3 equiv), **2** (0.1 mmol, 1 equiv), $Cu(MeCN)_4PF_6$ (3.7 mg, 10 mol%), **L1** (17.2 mg, 20 mol%). The tube was evacuated and filled with argon for three cycles. Then, 1 mL of Dichloromethane was added under argon. The reaction was allowed to stir at -70 °C for 48 hours. Upon completion, proper amount of silica gel was added to the reaction mixture. After removal of the solvent, the crude reaction mixture was purified on silica gel (petroleum ether and ethyl acetate) to afford the desired products.

1.7 General procedure F for kinetic resolution

In air, a 25 mL schlenk tube was charged with **1** (0.2 mmol, 2 equiv), **2** (0.1 mmol, 1 equiv), CuTc (1.9 mg,10 mol%), **L1** (9.5 mg,11 mol%), additive (7.2 mg,10 mol%). The tube was evacuated and filled with argon for three cycles. Then, 1 mL of Dichloromethane was added under argon. The reaction was allowed to stir at -70 °C for 48 hours. Upon completion, proper amount of silica gel was added to the reaction mixture. After removal of the solvent, the crude reaction mixture was purified on silica gel (petroleum ether and ethyl acetate) to afford the desired products.

1.8 Complementary experiments

Other amine boranes:

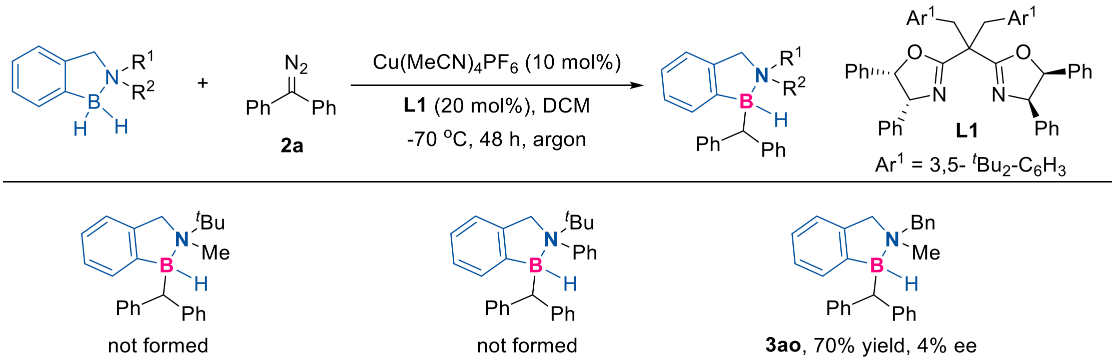

Other carbene precursors:

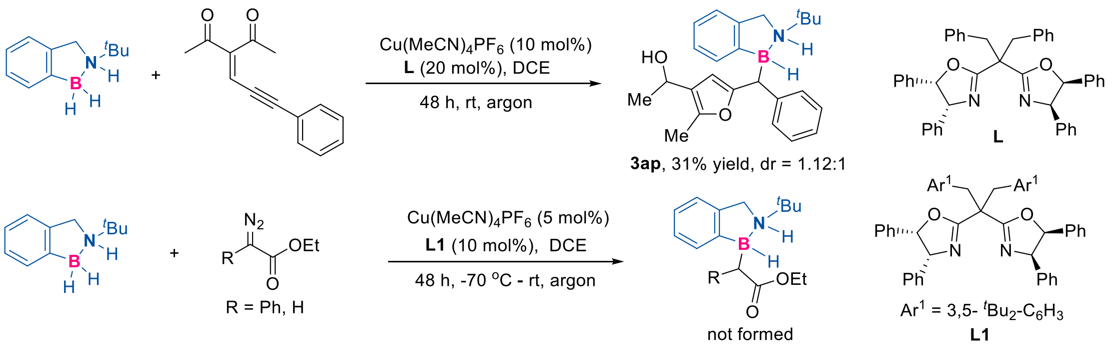

1.9 Optimization of reaction conditions

Table S1. Preliminary investigation of [Cu] catalysts

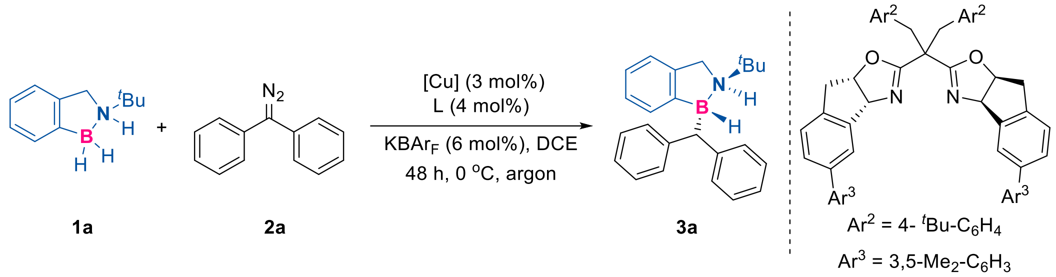

entry	[Cu] catalyst	**3a** ee [%] ^{a}	**3a** dr ^{b}
1	$Cu(MeCN)_4PF_6$	81	>20:1
2	$Cu(MeCN)_4BF_4$	71	>20:1
3	CuTc	81	>20:1
4	CuCl	80	>20:1
5	CuI	80	>20:1
6	CuBr	80	>20:1
7	$Cu(OTf)_2$	81	>20:1
8	$Cu(OAc)_2$	81	>20:1
9	$CuBr_2$	80	>20:1
10	$CuCl_2$	76	>20:1
11	$CuSO_4$	80	>20:1
12^{c}	$Cu(acac)_2$	73	>20:1
13^{c}	$Cu(CF_3COO)_2$	77	>20:1

Conditions: amine borane **1a** (0.3 mmol, 3.0 equiv), (diazomethylene)dibenzene (**2a**) (0.1 mmol, 1.0 equiv), [Cu] catalyst (3 mol%), **L6** (4 mol%), $KBAr_F$ (potassium tetrakis(perfluorophenyl)borate) (6 mol%), DCE (2 mL) at 0 °C, argon, 48 h. ^{a} Enantiomeric excess (ee) was determined by chiral HPLC. ^{b} Diastereomeric ratio (dr) was determined by chiral HPLC and crude ^{1}H NMR. ^{c} room temperature instead of 0 °C.

Table S2. Further investigation of [Cu] catalyst

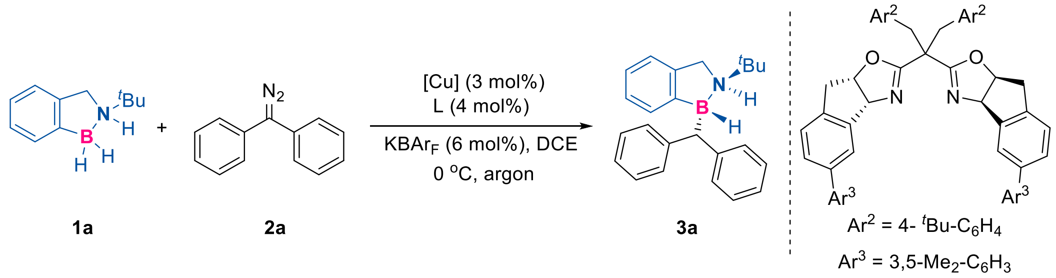

entry	[Cu] catalyst	**3a** ee (%) ^{a}	**3a** yield (%) ^{b}	**3a** dr ^{c}
1	$Cu(MeCN)_4PF_6$	81	71%	>20:1
2	CuTc	81	80%	>20:1
3	$Cu(OTf)_2$	81	70%	>20:1
4	$Cu(OAc)_2$	81	71%	>20:1

Conditions: amine borane **1a** (0.3 mmol, 3.0 equiv), (diazomethylene)dibenzene (**2a**) (0.1 mmol, 1.0 equiv), metal catalyst (3 mol%), **L6** (4 mol%), $KBAr_F$ (6 mol%), DCE (2 mL) at 0 °C, argon, 48 h. ^{a} ee

was determined by chiral HPLC. [b] Yields were determined by crude ^{1}H NMR with CH_2Br_2 as internal standard. [c] dr was determined by chiral HPLC and crude ^{1}H NMR.

Table S3. Investigation of solvents

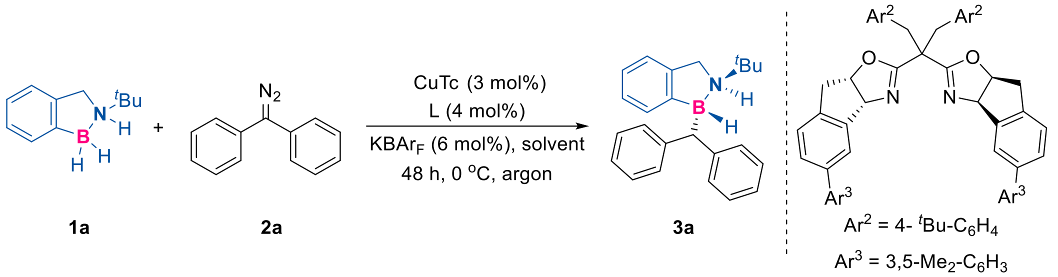

entry	solvent	**3a** ee (%) [a]	**3a** yield (%) [b]	**3a** dr [c]
1	DCM	76	79	>20:1
2	THF	-	trace	>20:1
3	toluene	-	trace	>20:1
4	MeCN	68	65	>20:1
5	Cl_3CH	66	70	>20:1

Conditions: amine borane **1a** (0.3 mmol, 3.0 equiv), (diazomethylene)dibenzene (**2a**) (0.1 mmol, 1.0 equiv), metal catalyst (3 mol%), **L6** (4 mol%), $KBAr_F$ (potassium tetrakis(perfluorophenyl)borate) (6 mol%), solvent (2 mL) at 0 °C, argon, 48 h. [a] ee was determined by chiral HPLC. [b] Yields were determined by crude ^{1}H NMR with CH_2Br_2 as internal standard. [c] dr was determined by chiral HPLC and crude ^{1}H NMR.

Table S4. Investigation of additives

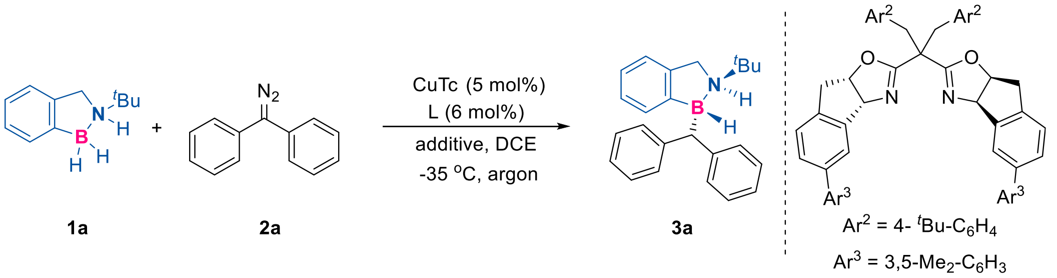

entry	additive	**3a** ee (%) [a]	**3a** yield (%) [b]	**3a** dr [c]
1	$KBAr_F$	89	52	>20:1
2	$[Ph_3C]$ BAr_F	86	50	>20:1
3	[*m*-*i*Pr-Ph_3C] BAr_F	88	46	>20:1
4	$NaBAr_F$	88	48	>20:1

Conditions: amine borane **1a** (0.3 mmol, 3.0 equiv), (diazomethylene)dibenzene (**2a**) (0.1 mmol, 1.0 equiv), CuTc (3 mol%), **L6** (4 mol%), additive (6 mol%), DCE (2 mL) at -35 °C, argon. [a] ee was determined by chiral HPLC. [b] Yields were determined by crude ^{1}H NMR with CH_2Br_2 as internal standard. [c] dr was determined by chiral HPLC and crude ^{1}H NMR.

Table S5. Investigation of ligands

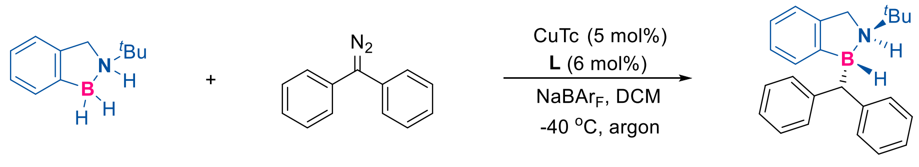

1a **2a** **3a**

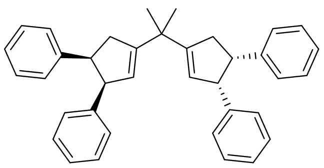

83%, 54% ee, dr > 20: 1

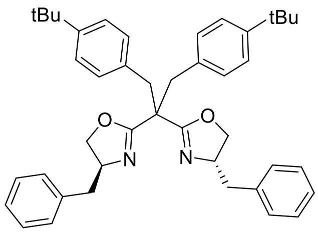

20%, 28% ee, dr > 20: 1

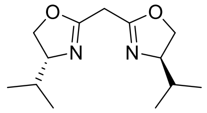

26%, 56% ee, dr > 20: 1

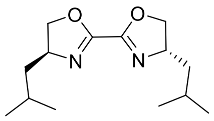

trace

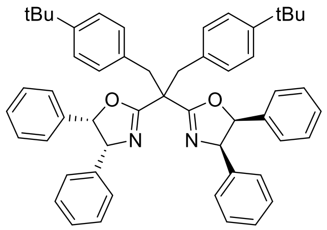

90%, 59% ee, dr > 20: 1

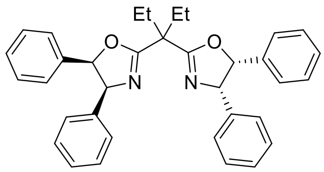

80%, 43% ee, dr > 20: 1

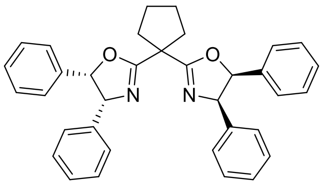

90%, 56% ee, dr > 20: 1

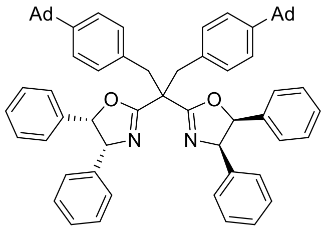

80%, 57% ee, dr > 20: 1

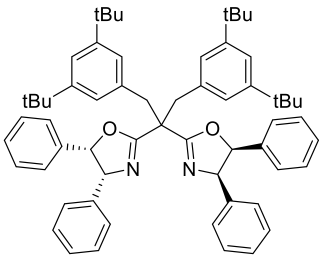

88%, 77% ee, dr > 20: 1

Conditions: amine borane **1a** (0.3 mmol, 3.0 equiv), (diazomethylene)dibenzene (**2a**) (0.1 mmol, 1.0 equiv), metal catalyst (3 mol%), **L** (4 mol%), additive (6 mol%), DCM (2 mL) at -40 °C, argon. ee was determined by chiral HPLC. Yields were determined by crude ^{1}H NMR with CH_2Br_2 as internal standard. dr was determined by chiral HPLC and crude ^{1}H NMR.

2 Analysis data of compounds 1, 2 and 3

2-(tert-butyl)-2,3-dihydro-1H-benzo[c][1,2]azaborole (1a)

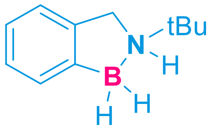

1a

Following the above procedure **A**, isolated yield: 57%, (998 mg), white solid (mp: 109.5 – 111.2 ºC), R_f = 0.3 (silica gel, hexane/DCM = 3:1, v/v), column chromatography (silica gel, hexane/DCM = 2:1, v/v).

^{1}H NMR (500 MHz, $CDCl_3$) δ 7.40 (d, J = 7.2 Hz, 1H), 7.18 (t, J = 7.2 Hz, 1H), 7.07 (t, J = 7.4 Hz, 1H), 6.98 (d, J = 7.5 Hz, 1H), 4.05 – 3.97 (m, 2H), 3.84 (d, J = 6.2 Hz, 1H), 2.75 (br, 2H), 1.34 (s, 9H).

^{13}C NMR (126 MHz, $CDCl_3$) δ 139.1, 129.2, 127.0, 124.8, 121.4, 56.2, 52.2, 26.9.

^{11}B NMR (160 MHz, $CDCl_3$) δ -11.1.

HRMS (ESI) m/z: $[M+H]^+$ Calcd. for $C_{11}H_{19}BN$ 176.1605; Found: 176.1602.

Following the above procedure **F**, racemic amine borane (**1a-1**), isolated yield: 45%, (15.8 mg).

HPLC analysis: DAICEL CHIRALCEL IG, hexane/isopropanol = 80/20, 0.5 mL/min, λ = 267 nm, t_1 = 9.605 min, t_2 = 10.434 min, 90% ee.

[α]$^{25}_D$: -26.05 (*c* 0.3, $CHCl_3$).

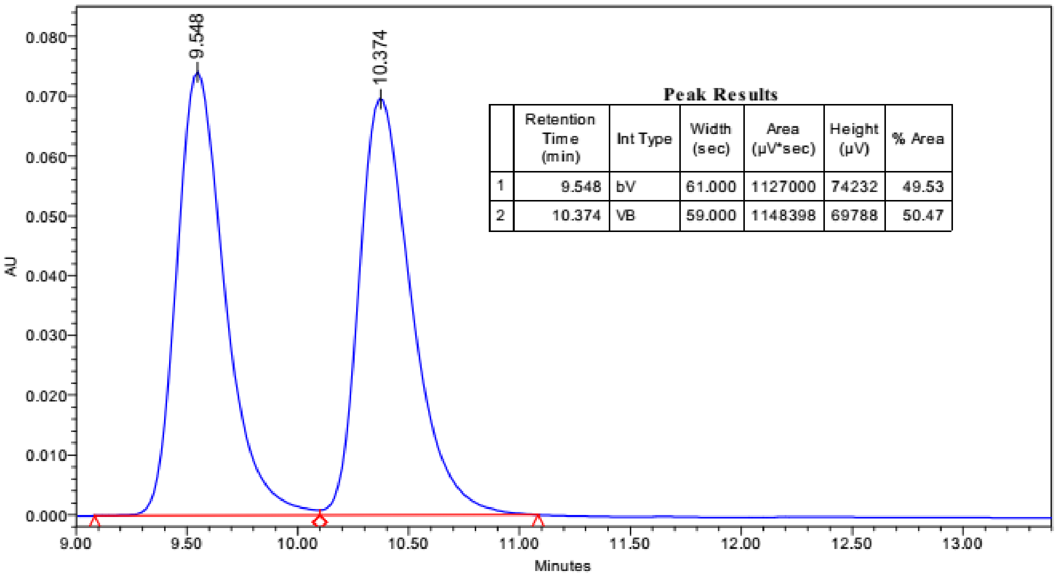

	Retention Time (min)	Int Type	Width (sec)	Area (µV*sec)	Height (µV)	% Area
1	9.548	bV	61.000	1127000	74232	49.53
2	10.374	VB	59.000	1148398	69788	50.47

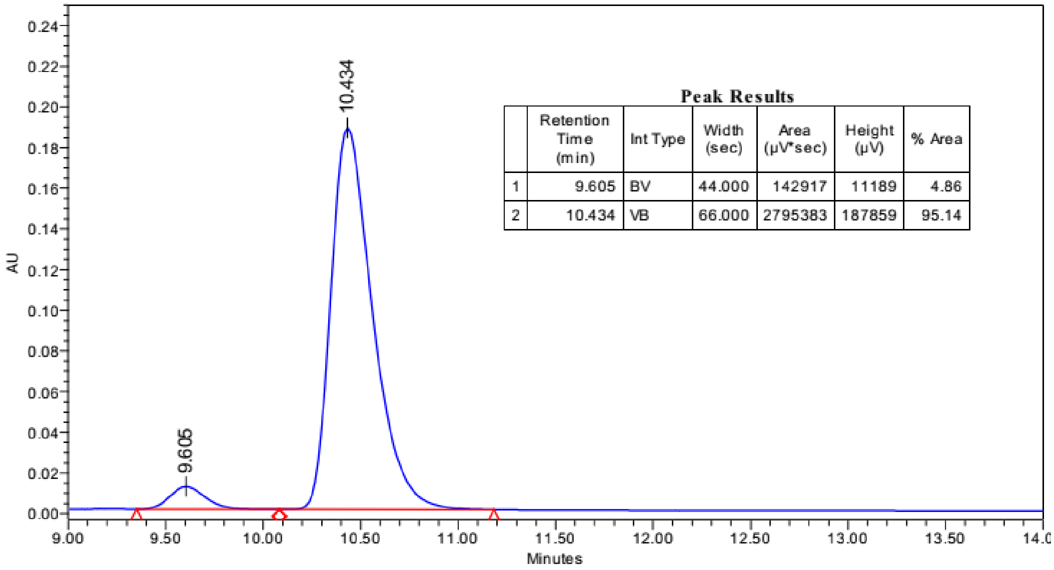

	Retention Time (min)	Int Type	Width (sec)	Area (µV*sec)	Height (µV)	% Area
1	9.605	BV	44.000	142917	11189	4.86
2	10.434	VB	66.000	2795383	187859	95.14

2-(tert-butyl)-5-methoxy-2,3-dihydro-1H-benzo[c][1,2]azaborole (**1b**)

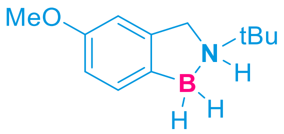

1b

Following the above procedure **A**, isolated yield: 35%, (718 mg), white solid (mp: 140.1 – 142.7 °C), R_f = 0.3 (silica gel, hexane/DCM = 3:1, v/v), column chromatography (silica gel, hexane/DCM = 2:1, v/v).

^{1}H NMR (500 MHz, $CDCl_3$) δ 7.27 (d, J = 8.0 Hz, 1H), 6.78 (dd, J = 8.0, 2.3 Hz, 1H), 6.52 (d, J = 2.2 Hz, 1H), 4.07 (s, 1H), 3.94 (dd, J = 13.6, 10.2 Hz, 1H), 3.77 (s, 3H), 3.71 (dd, J = 13.7, 6.4 Hz, 1H), 2.69 (br, 2H), 1.31 (s, 9H).

^{13}C NMR (126 MHz, $CDCl_3$) δ 157.8, 140.4, 129.9, 113.3, 107.3, 56.2, 55.2, 52.2, 26.8.

^{11}B NMR (160 MHz, $CDCl_3$) δ -11.3.

HRMS (ESI) m/z: $[M+H]^+$ Calcd. for $C_{12}H_{21}BNO$ 206.1711; Found: 206.1713.

2-(tert-butyl)-5-methyl-2,3-dihydro-1H-benzo[c][1,2]azaborole (**1c**)

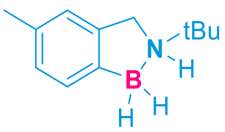

1c

Following the above procedure **A**, isolated yield: 46%, (870 mg), white solid (mp: 131.5 – 133.8 °C), R_f = 0.3 (silica gel, hexane/DCM = 3:1, v/v), column chromatography (silica gel, hexane/DCM = 2:1, v/v).

^{1}H NMR (500 MHz, $CDCl_3$) δ 7.29 (d, J = 7.3 Hz, 1H), 7.02 (d, J = 7.3 Hz, 1H), 6.77 (s, 1H), 4.03 – 3.92 (m, 2H), 3.77 (t, J = 5.8 Hz, 1H), 2.72 (br, 2H), 2.31 (s, 3H), 1.32 (s, 9H).

^{13}C NMR (126 MHz, $CDCl_3$) δ 139.3, 134.2, 129.1, 127.9, 122.1, 56.1, 52.2, 26.9, 21.2.

^{11}B NMR (128 MHz, $CDCl_3$) δ -10.0.

HRMS (ESI) m/z: $[M+H]^+$ Calcd. for $C_{12}H_{21}BN$ 190.1762; Found: 190.1761.

2-(tert-butyl)-5-chloro-2,3-dihydro-1H-benzo[c][1,2]azaborole (1d)

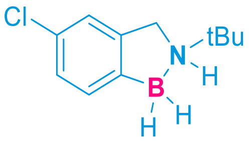

1d

Following the above procedure **A**, isolated yield: 30%, (627 mg), white solid (mp: 132.8 – 135.8 °C), R_f = 0.3 (silica gel, hexane/DCM = 3:1, v/v), column chromatography (silica gel, hexane/DCM = 2:1, v/v).

^{1}H NMR (500 MHz, $CDCl_3$) δ 7.28 (d, J = 7.7 Hz, 1H), 7.14 (dd, J = 7.7, 1.6 Hz, 1H), 6.91 (d, J = 7.8 Hz, 1H), 4.04 (s, 1H), 3.96 (dd, J = 23.6, 10.1 Hz, 1H), 3.84 – 3.73 (m, 1H), 2.68 (br, 2H), 1.32 (s, 9H).

^{13}C NMR (126 MHz, $CDCl_3$) δ 140.8, 130.58 (s), 130.6, 130.3, 127.0, 121.6, 56.5, 51.8, 26.9.

^{11}B NMR (160 MHz, $CDCl_3$) δ -11.2.

HRMS (ESI) m/z: $[M+H]^+$ Calcd. for $C_{11}H_{18}BClN$ 210.1216; Found: 210.1213.

2-(tert-butyl)-6-fluoro-2,3-dihydro-1H-benzo[c][1,2]azaborole (1e)

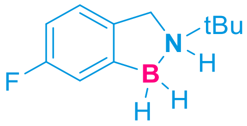

1e

Following the above procedure **A**, isolated yield: 51%, (985 mg), white solid (mp: 108.2 – 110.5 °C), R_f = 0.3 (silica gel, hexane/DCM = 3:1, v/v), column chromatography (silica gel, hexane/DCM = 2:1, v/v).

^{1}H NMR (500 MHz, $CDCl_3$) δ 7.03 (dd, J = 9.0, 2.3 Hz, 1H), 6.89 (dd, J = 8.1, 5.0 Hz, 1H), 6.74 – 6.70 (m, 1H), 4.01 (s, 1H), 3.98 – 3.91 (m, 1H), 3.81 (dd, J = 12.9, 6.0 Hz, 1H), 2.67 (d, J = 46.3 Hz, 2H), 1.32 (s, 9H).

^{13}C NMR (101 MHz, $CDCl_3$) δ 162.8(d, J = 162.8 Hz), 134.2, 122.5 (d, J = 8.0 Hz), 115.2 (d, J = 18.8 Hz), 111.8, 111.5, 56.4, 51.6, 26.9.

^{11}B NMR (128 MHz, $CDCl_3$) δ -10.1.

^{19}F NMR (376 MHz, $CDCl_3$) δ -117.2.

HRMS (ESI) m/z: $[M+Cl]^-$ Calcd. for $C_{11}H_{17}BClFN$ 228.1132; Found: 228.1130.

2-(tert-pentyl)-2,3-dihydro-1H-benzo[c][1,2]azaborole (1f)

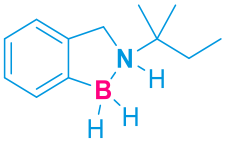

1f

Following the above procedure **A**, isolated yield: 45%, (842 mg), white solid (mp: 72.7 – 75.1 °C), R_f = 0.3 (silica gel, hexane/DCM = 3:1, v/v), column chromatography (silica gel, hexane/DCM = 2:1, v/v).

^{1}H NMR (500 MHz, $CDCl_3$) δ 7.42 (d, J = 7.2 Hz, 1H), 7.19 (t, J = 7.2 Hz, 1H), 7.09 (t, J = 7.3 Hz, 1H), 7.04 (d, J = 7.5 Hz, 1H), 4.11 (d, J = 4.1 Hz, 2H), 3.97 (dd, J = 13.5, 8.7 Hz, 1H), 2.76 (br, 2H), 1.85 – 1.78 (m, 1H), 1.71 – 1.64 (m, 1H), 1.32 (s, 3H), 1.30 (s, 3H), 0.97 (t, J = 7.5 Hz, 3H).

^{13}C NMR (101 MHz, $CDCl_3$) δ 138.7, 129.3, 127.0, 124.8, 121.3, 58.7, 51.8, 32.8, 23.6, 23.4, 8.0.

^{11}B NMR (128 MHz, $CDCl_3$) δ 10.1(t, J = 104.3 Hz).

HRMS (ESI) m/z: $[M+Cl]^-$ Calcd. for $C_{12}H_{20}BNCl$ 224.1383; Found: 224.1387.

2-(2,4,4-trimethylpentan-2-yl)-2,3-dihydro-1H-benzo[c][1,2]azaborole (1g)

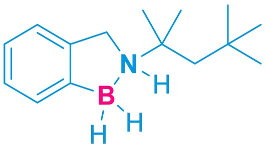

1g

Following the above procedure **A**, isolated yield: 48%, (1.11 g), white solid (mp: 84.1 – 86.9 °C), R_f = 0.3 (silica gel, hexane/DCM = 3:1, v/v), column chromatography (silica gel, hexane/DCM = 2:1, v/v).

^{1}H NMR (500 MHz, $CDCl_3$) δ 7.41 (d, J = 7.2 Hz, 1H), 7.19 (t, J = 7.1 Hz, 1H), 7.11 – 7.04 (m, 2H), 4.32 (s, 1H), 4.18 – 4.11 (m, 1H), 4.09 – 4.02 (m, 1H), 2.84 (d, J = 86.7 Hz, 2H), 1.90 (d, J = 14.8 Hz, 1H), 1.65 (d, J = 14.8 Hz, 1H), 1.45 (s, 3H), 1.43 (s, 3H), 1.09 (s, 9H).

^{13}C NMR (126 MHz, $CDCl_3$) δ 138.6, 129.3, 127.1 124.9, 121.2, 60.4, 51.8, 51.2, 31.5, 26.4, 25.5.

^{11}B NMR (160 MHz, $CDCl_3$) δ -12.1.

HRMS (ESI) m/z: $[M+Cl]^-$ Calcd. for $C_{15}H_{26}BNCl$ 266.1852; Found: 266.1856.

2-(2-phenylpropan-2-yl)-2,3-dihydro-1H-benzo[c][1,2]azaborole (1h)

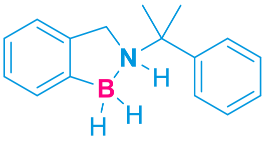

1h

Following the above procedure A, isolated yield: 47%, (1.1 g), white solid (mp: 98.1 – 101.5 °C), R_f = 0.3 (silica gel, hexane/DCM = 3:1, v/v), column chromatography (silica gel, hexane/DCM = 2:1, v/v).

^{1}H NMR (500 MHz, $CDCl_3$) δ 7.52 – 7.47 (m, 4H), 7.45 – 7.40 (m, 2H), 7.17 (t, J = 7.3 Hz, 1H), 7.04 (t, J = 7.4 Hz, 1H), 6.88 (d, J = 7.5 Hz, 1H), 4.71 (s, 1H), 3.92 (dd, J = 13.9, 9.6 Hz, 1H), 3.51 (dd, J = 14.0, 6.2 Hz, 1H), 2.95 (br, 2H), 1.80 (s, 3H), 1.78 (s, 3H).

^{13}C NMR (126 MHz, $CDCl_3$) δ 143.4, 138.6, 129.2, 129.0, 128.1, 127.0, 125.2, 124.8, 121.1, 60.5, 53.4, 29.4, 20.3.

^{11}B NMR (160 MHz, $CDCl_3$) δ -10.6.

HRMS (ESI) m/z: $[M+Cl]^-$ Calcd. for $C_{16}H_{20}BNCl$ 272.1383; Found: 272.1390.

2-(1,3-dihydro-2H-benzo[c][1,2]azaborol-2-yl)-2-methylpropan-1-ol (**1i**)

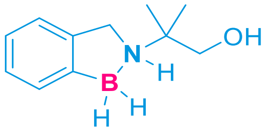

1i

Following the above procedure **A**, isolated yield: 15%, (287 mg), white solid (mp: 75.2 – 77.7 °C), R_f = 0.3 (silica gel, hexane/DCM = 2:1, v/v), column chromatography (silica gel, hexane/DCM = 1:1, v/v).

^{1}H NMR (500 MHz, $CDCl_3$) δ 7.43 (d, J = 7.2 Hz, 1H), 7.20 (t, J = 7.1 Hz, 1H), 7.13 – 7.05 (m, 2H), 4.83 (s, 1H), 4.11 – 4.03 (m, 1H), 3.99 (dd, J = 13.3, 6.3 Hz, 1H), 3.83 (d, J = 11.2 Hz, 1H), 3.47 (d, J = 11.3 Hz, 1H), 2.74 (br, 2H), 2.43 (s, 1H), 1.30 (s, 3H), 1.27 (s, 3H).

^{13}C NMR (126 MHz, $CDCl_3$) δ 138.7, 129.3, 127.0, 124.9, 121.3, 68.3, 58.6, 51.5, 21.7, 20.1.

^{11}B NMR (160 MHz, $CDCl_3$) δ -11.4.

HRMS (ESI) m/z: $[M+H]^+$ Calcd. for $C_{11}H_{19}BNO$ 192.1554; Found: 192.1552.

2-(3-methylbenzyl)-2,3-dihydro-1H-benzo[c][1,2]azaborole (**1j**)

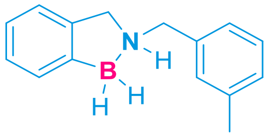

1j

Following the above procedure A, isolated yield: 53%, (1.18 g), white solid (mp: 128.4 – 130.8 °C), R_f = 0.3 (silica gel, hexane/DCM = 3:1, v/v), column chromatography (silica gel, hexane/DCM = 2:1, v/v).

^{1}H NMR (500 MHz, $CDCl_3$) δ 7.41 (d, J = 7.2 Hz, 1H), 7.34 (t, J = 7.5 Hz, 1H), 7.24 (d, J = 7.6 Hz, 1H), 7.19 – 7.12 (m, 3H), 7.06 (t, J = 7.3 Hz, 1H), 6.99 (d, J = 7.5 Hz, 1H), 4.41 (s, 1H), 4.27 (dd, J = 13.7, 3.7 Hz, 1H), 3.92 (dd, J = 13.6, 5.7 Hz, 1H), 3.85 (dd, J = 13.7, 8.0 Hz, 1H), 3.80 (dd, J = 13.7, 9.9 Hz, 1H), 2.87 (br, 2H), 2.42 (s, 3H).

^{13}C NMR (126 MHz, $CDCl_3$) δ 139.0, 134.5, 129.8, 129.8, 129.6, 129.3, 129.0, 127.1, 126.1, 124.9, 121.5, 57.2, 57.1, 21.3.

^{11}B NMR (160 MHz, $CDCl_3$) δ -7.4.

HRMS (ESI) m/z: $[M+H]^+$ Calcd. for $C_{12}H_{19}BN$ 224.1605; Found: 224.1636.

2-(tert-butyl)-1,2,3,4-tetrahydrobenzo[c][1,2]azaborinine (**1k**)

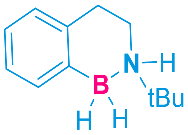

1k

Following the above procedure **B**, isolated yield: 10%, (187 mg), white solid (mp: 112.6 – 115.3 °C), R_f = 0.3 (silica gel, hexane/DCM = 3:1, v/v), column chromatography (silica gel, hexane/DCM = 2:1, v/v).

^{1}H NMR (500 MHz, $CDCl_3$) δ 7.30 (d, *J* = 7.3 Hz, 1H), 7.11 (t, *J* = 7.2 Hz, 1H), 7.03 (t, *J* = 7.0 Hz, 1H), 6.97 (d, *J* = 7.4 Hz, 1H), 3.28 (d, *J* = 11.6 Hz, 1H), 3.06 (s, 1H), 2.9 – 2.8 (m, 2H), 2.84 – 2.76 (m, 1H), 2.46 (br, 2 H), 1.38 (s, 9H).

^{13}C NMR (126 MHz, $CDCl_3$) δ 136.6, 133.0, 126.6, 125.5, 124.1, 57.9, 45.0, 32.5, 26.4.

^{11}B NMR (400 MHz, $CDCl_3$) δ 4.82 (t, *J* = 10.1 Hz).

HRMS (ESI) m/z: $[M+H]^+$ Calcd. for $C_{12}H_{21}BN$ 190.1762; Found: 190.1765.

2-(tert-butyl)-5,6-dimethoxy-2,3-dihydro-1H-1λ^4,2λ^4-benzo[c][1,2]azaborole (1l)

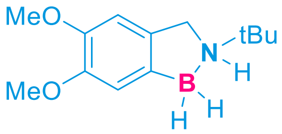

1l

Following the above procedure **A**, isolated yield: 37%, (870 mg), white solid (mp: 134.7– 137.6 °C), R_f = 0.3 (silica gel, hexane/DCM = 3:1, v/v), column chromatography (silica gel, hexane/DCM = 1:1, v/v).

^{1}H NMR (500 MHz, $CDCl_3$) δ 6.91 (s, 1H), 6.54 (s, 1H), 4.06 (s, 1H), 3.95 (t, *J* = 11.4 Hz, 1H), 3.86 (s, 3H), 3.80 (s, 3H), 3.78 – 3.77 (m, 1H), 2.69 (br, 1H), 1.30 (s, 9H).

^{13}C NMR (126 MHz, $CDCl_3$) δ 148.6, 147.0, 130.3, 111.5, 105.6, 56.2, 56.0, 55.7, 52.2, 26.8.

^{11}B NMR (160 MHz, $CDCl_3$) δ -11.2.

HRMS (ESI) m/z: $[M+H]^+$ Calcd. for $C_{13}H_{23}BNO_2$ 236.1817; Found: 236.1813.

Following the above procedure **F**, racemic amine borane (**1l-1**), isolated yield: 40%, (18.8 mg).

HPLC analysis: DAICEL CHIRALCEL AD-H, hexane/isopropanol = 80/20, 0.5 mL/min, λ = 267 nm, t_1 = 14.331 min, t_2 = 16.964 min, 82% ee.

$[\alpha]^{25}_D$: -31.714 (*c* 0.35, $CHCl_3$)

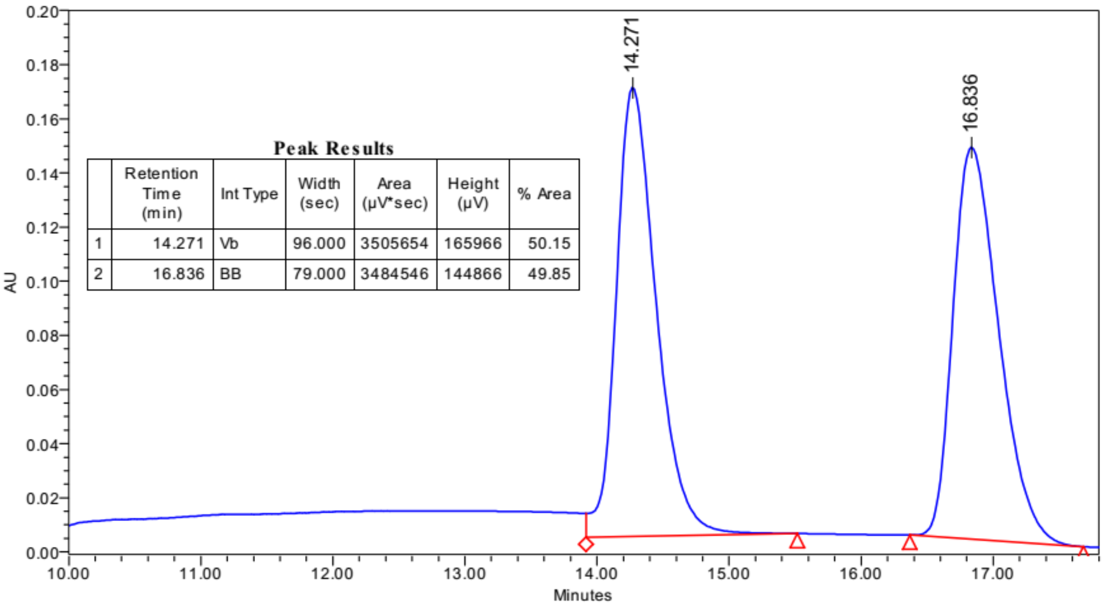

Peak Results

	Retention Time (min)	Int Type	Width (sec)	Area (μV*sec)	Height (μV)	% Area
1	14.271	Vb	96.000	3505654	165966	50.15
2	16.836	BB	79.000	3484546	144866	49.85

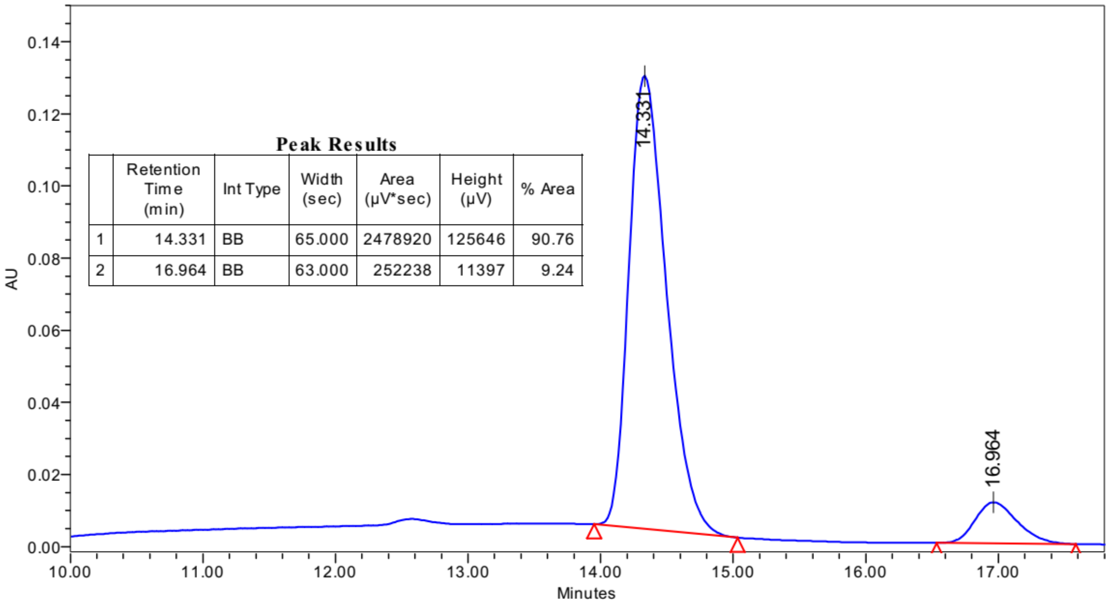

Peak Results

	Retention Time (min)	Int Type	Width (sec)	Area (μV*sec)	Height (μV)	% Area
1	14.331	BB	65.000	2478920	125646	90.76
2	16.964	BB	63.000	252238	11397	9.24

2-(tert-butyl)-6-methoxy-2,3-dihydro-1H-1λ^4,2λ^4-benzo[c][1,2]azaborole (1m)

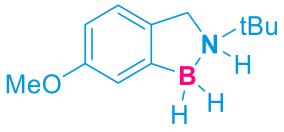

1m

Following the above procedure **A**, isolated yield: 41%, (841 mg), white solid (mp: 110.4 – 112.8 °C), R_f = 0.3 (silica gel, hexane/DCM = 3:1, v/v), column chromatography (silica gel, hexane/DCM = 1:1, v/v).

^{1}H NMR (500 MHz, $CDCl_3$) δ 6.95 (d, *J* = 2.3 Hz, 1H), 6.89 (d, *J* = 8.2 Hz, 1H), 6.63 (dd, *J* = 8.2, 2.5 Hz, 1H), 3.99 – 3.91 (m, 2H), 3.83 – 3.78 (m, 4H), 2.71 (br, 2H), 1.31 (s, 9H).

^{13}C NMR (126 MHz, $CDCl_3$) δ 159.0, 131.1, 122.2, 113.3, 111.5, 56.2, 55.2, 51.7, 26.9.

^{11}B NMR (128 MHz, $CDCl_3$) δ -10.0.

HRMS (ESI) m/z: $[M+H]^+$ Calcd. for $C_{12}H_{21}BNO$ 206.1711; Found: 260.1713.

Following the above procedure **F**, racemic amine borane (**1m-1**),isolated yield: 41%, (16.8 mg).

HPLC analysis: DAICEL CHIRALCEL IC, hexane/isopropanol = 70/30, 0.5 mL/min, λ = 267 nm, t_1 = 17.359 min, t_2 = 19.164 min, 90% ee.

$[\alpha]^{25}_D$: -27.813 (*c* 0.32, $CHCl_3$)

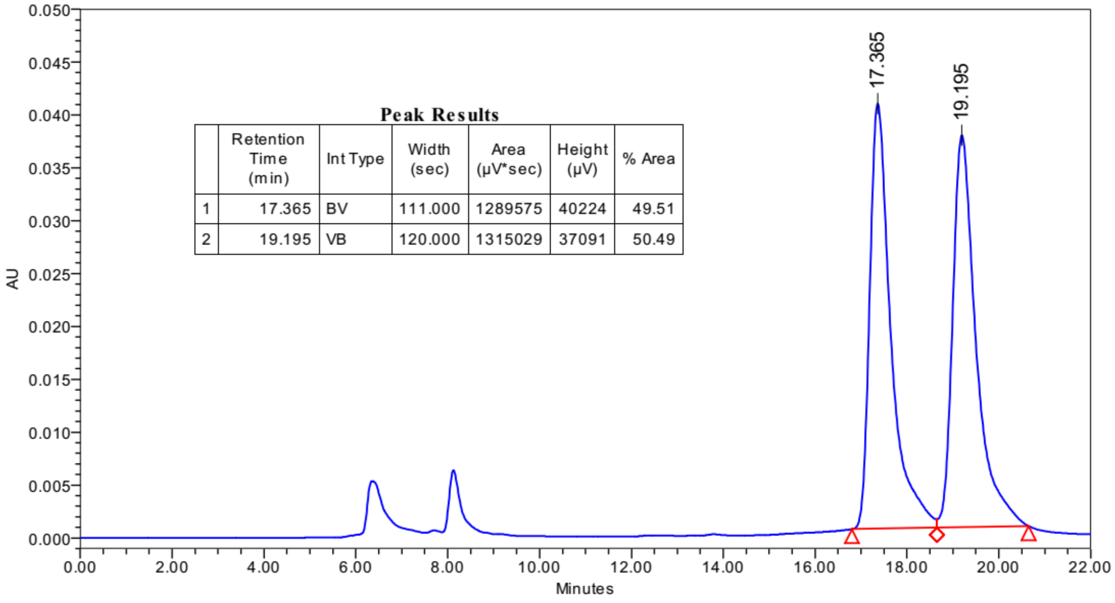

	Retention Time (min)	Int Type	Width (sec)	Area (μV*sec)	Height (μV)	% Area
1	17.365	BV	111.000	1289575	40224	49.51
2	19.195	VB	120.000	1315029	37091	50.49

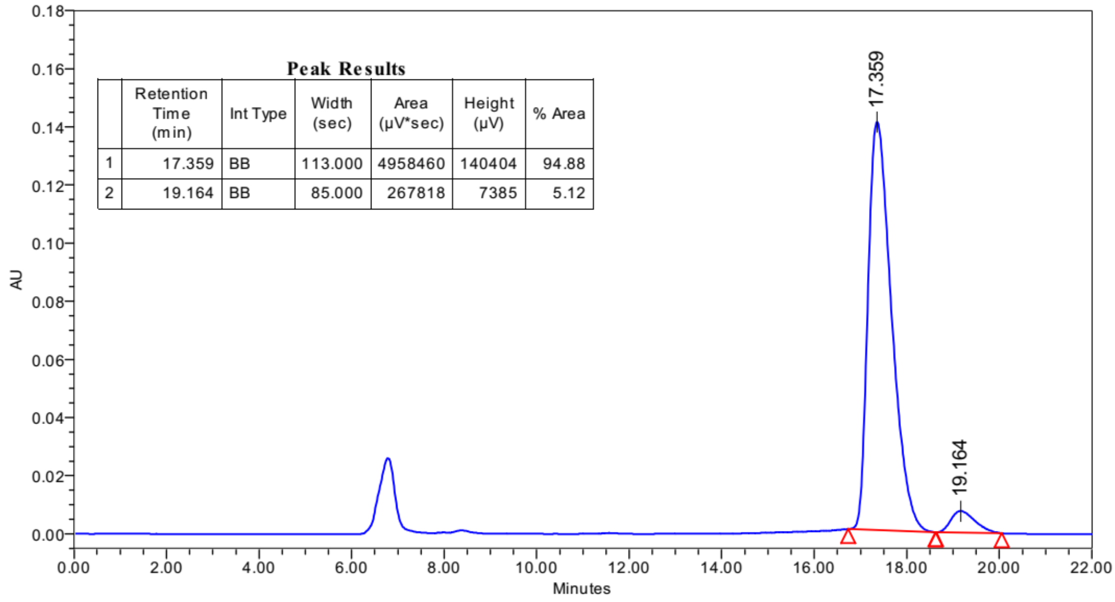

	Retention Time (min)	Int Type	Width (sec)	Area (μV*sec)	Height (μV)	% Area
1	17.359	BB	113.000	4958460	140404	94.88
2	19.164	BB	85.000	267818	7385	5.12

2-(tert-butyl)-5-fluoro-2,3-dihydro-1H-1λ⁴,2λ⁴-benzo[c][1,2]azaborole (1n)

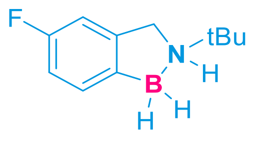

1n

Following the above procedure **A**, isolated yield: 52%, (1 g), white solid (mp: 118.2 – 120.3 ºC), R_f = 0.3 (silica gel, hexane/DCM = 3:1, v/v), column chromatography (silica gel, hexane/DCM = 2:1, v/v).

^{1}H NMR (500 MHz, $CDCl_3$) δ 7.32 – 7.28 (m, 1H), 6.90– 6.82 (m, 1H), 6.67 (d, *J* = 9.4 Hz, 1H), 4.09 (s, 1H), 4.00 (t, *J* = 11.9 Hz, 1H), 3.89 – 3.80 (m, 1H), 2.72 (br, 2H), 1.34 (s, 9H).

^{13}C NMR (101 MHz, $CDCl_3$) δ 162.77 (s), 160.38 (s), 140.35 (d, *J* = 7.5 Hz), 130.22 (d, *J* = 7.5 Hz), 114.0 (d, *J* = 19.9 Hz), 108.4 (d, *J* = 21.2 Hz), 56.5, 52.0 (d, *J* = 3.2 Hz), 27.0.

^{19}F NMR (471 MHz, $CDCl_3$) δ -120.17 (s).

^{11}B NMR (128 MHz, $CDCl_3$) δ -10.13 (t, *J* = 105.4 Hz).

HRMS (ESI) m/z: $[M+Cl]^-$ Calcd. for $C_{11}H_{17}BFNCl$ 228.1132; Found: 228.1129.

Following the above procedure **F**, racemic amine borane (**1n-1**), isolated yield: 45%, (17.4 mg).

HPLC analysis: DAICEL CHIRALCEL AS-H, hexane/isopropanol = 80/20, 0.5 mL/min, λ = 267 nm, t_1 = 8.430 min, t_2 = 9.454 min, 81% ee.

$[\alpha]^{25}_{D}$: -18.333 (*c* 0.3, $CHCl_3$).

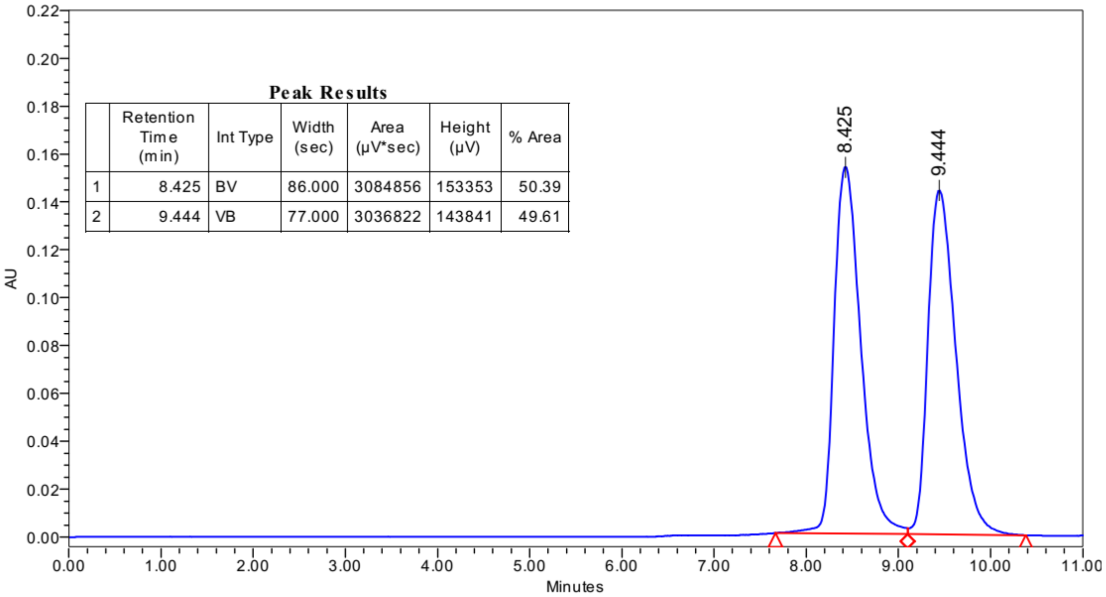

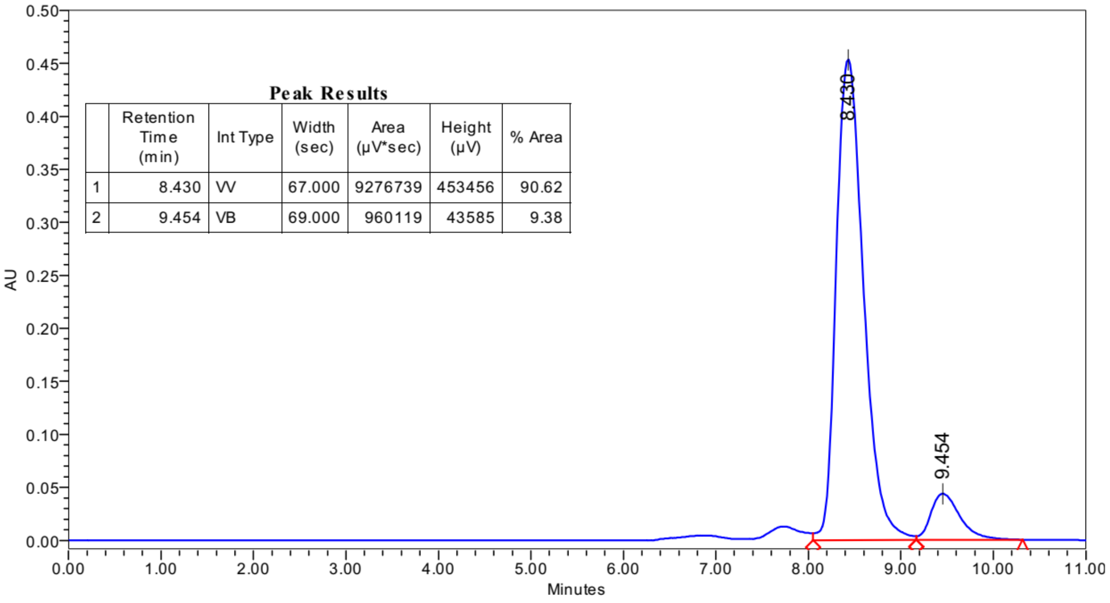

	Retention Time (min)	Int Type	Width (sec)	Area (μV*sec)	Height (μV)	% Area
1	8.430	VV	67.000	9276739	453456	90.62
2	9.454	VB	69.000	960119	43585	9.38

2-(tert-butyl)-2,3-dihydro-1H-1λ^4-benzo[c][1,2]azaborole-1,1,3-d$_3$ (**1a-*d$_3$***)

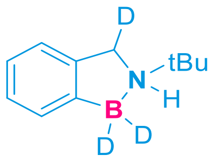

1a-*d$_3$*

Following the above procedure A on 5 mmol scale, isolated yield: 36%, (320 mg), white solid (mp: 97.6 – 98.9 °C), R_f = 0.3 (silica gel, hexane/DCM = 3:1, v/v), column chromatography (silica gel, hexane/DCM = 2:1, v/v).

^{1}H NMR (400 MHz, $CDCl_3$) δ 7.40 (d, *J* = 6.9 Hz, 1H), 7.19 (t, *J* = 7.0 Hz, 1H), 7.08 (t, *J* = 7.1 Hz, 1H), 6.97 (d, *J* = 7.2 Hz, 1H), 3.88 (d, *J* = 71.7 Hz, 2H), 1.33 (s, 9H).

^{13}C NMR (101 MHz, $CDCl_3$) δ 139.0, 129.3, 127.0, 124.9, 121.4, 56.2, 51.9 (t, *J* = 20.9 Hz), 27.0.

^{11}B NMR (128 MHz, $CDCl_3$) δ -10.3.

HRMS (ESI) m/z: $[M+H]^+$ Calcd. for $C_{11}H_{16}D_3BN$ 179.1794; Found: 179.1799.

On the basis of reaction procedure **E**, the amount of **1a-d$_3$** was reduced to 2 equivalent.

HPLC analysis: DAICEL CHIRALCEL IG, hexane/isopropanol = 80/20, 0.5 mL/min, λ = 267 nm, t_1 = 9.742 min, t_2 = 10.618 min, 94% ee.

$[\alpha]^{25}_D$: +12.667 (*c* 0.3, $CHCl_3$).

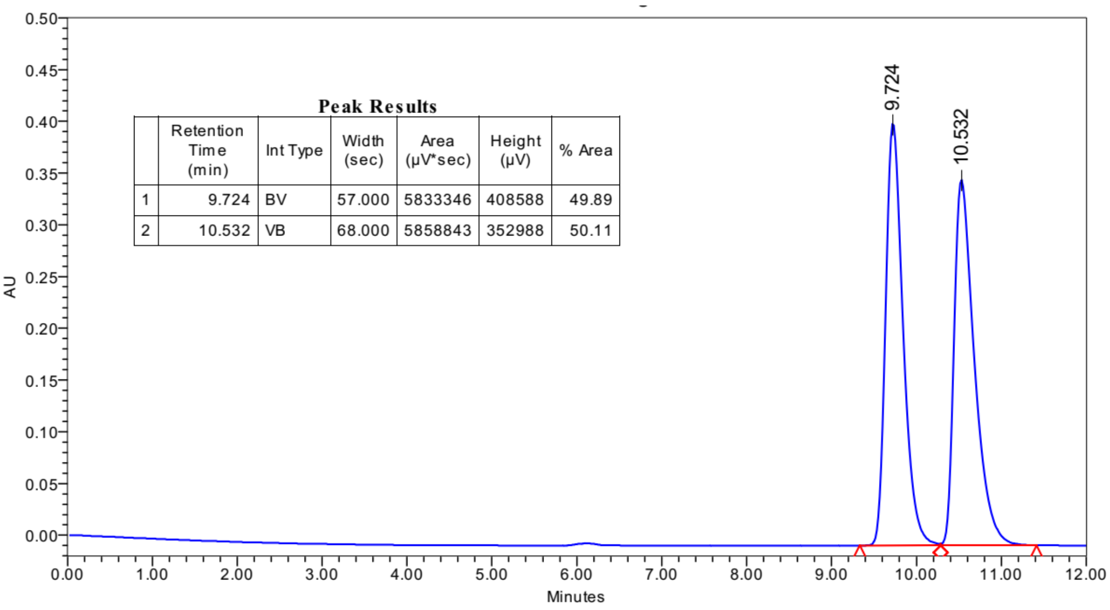

Peak Results

	Retention Time (min)	Int Type	Width (sec)	Area (µV*sec)	Height (µV)	% Area
1	9.724	BV	57.000	5833346	408588	49.89
2	10.532	VB	68.000	5858843	352988	50.11

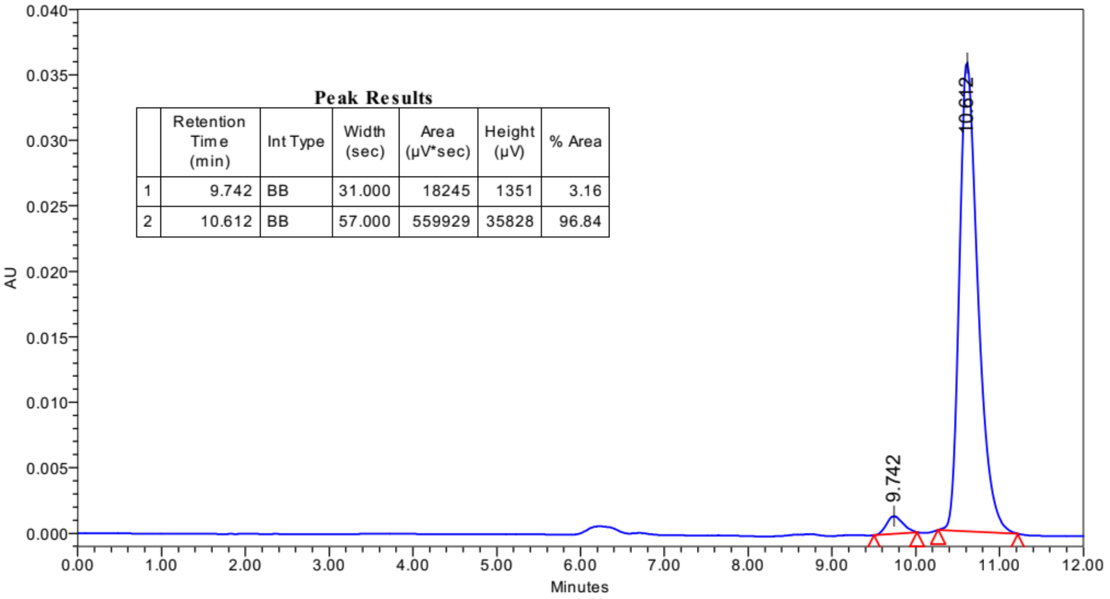

Peak Results

	Retention Time (min)	Int Type	Width (sec)	Area (µV*sec)	Height (µV)	% Area
1	9.742	BB	31.000	18245	1351	3.16
2	10.612	BB	57.000	559929	35828	96.84

(diazomethylene)dibenzene (2a)

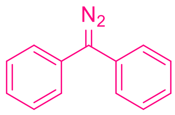

2a

Following the above procedure **C**, the product was obtained as a purple solid, yield: 90% (873 mg), R_f = 0.5 (silica gel, PE/Et_3N = 10:1), column chromatography (silica gel, PE/Et_3N = 20:1). Known compound.[1]

^{1}H NMR (500 MHz, $CDCl_3$) δ 7.40 (t, J = 7.5 Hz, 4H), 7.32 (d, J = 8.0 Hz, 4H), 7.20 (dd, J = 11.0, 4.1 Hz, 2H).

4,4'-(diazomethylene)bis(methoxybenzene)(2b)

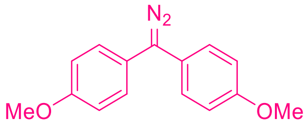

2b

Following the above procedure **C**, the product was obtained as a purple solid, yield: 58% (734 mg), R_f = 0.5 (silica gel, PE/Et_3N = 10:1), column chromatography (silica gel, PE/Et_3N = 20:1). Known compound.[1]

^{1}H NMR (500 MHz, $CDCl_3$) δ 7.22 – 7.16 (m, 4H), 6.98 – 6.92 (m, 4H), 3.83 (s, 6H).

4,4'-(diazomethylene)bis(fluorobenzene) (**2d**)

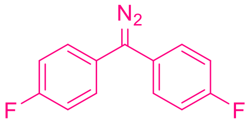

2d

Following the above procedure **C**, the product was obtained as a purple solid, yield: 65% (747 mg), R_f = 0.5 (silica gel, PE/Et_3N = 10:1), column chromatography (silica gel, PE/Et_3N = 20:1). Known compound.[1]

^{1}H NMR (500 MHz, $CDCl_3$) δ 7.24 – 7.19 (m, 4H), 7.13 – 7.07 (m, 4H).

3,3'-(diazomethylene)bis(methoxybenzene) (**2e**)

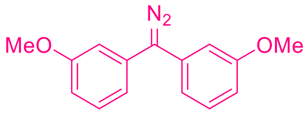

2e

Following the above procedure **C**, the product was obtained as a purple solid, yield: 62% (787 mg), R_f = 0.5 (Al_2O_3 base, PE/Et_3N = 10:1), column chromatography (Al_2O_3 base, PE/Et_3N = 20:1).

^{1}H NMR (500 MHz, $CDCl_3$) δ 7.31 (t, J = 8.0 Hz, 2H), 6.91 (dd, J = 7.1, 1.2 Hz, 2H), 6.86 (t, J = 1.8 Hz, 2H), 6.75 (dd, J = 8.3, 2.5 Hz, 2H), 3.81 (s, 6H).

^{13}C NMR (126 MHz, $CDCl_3$) δ 160.2, 131.0, 130.1, 117.8, 111.1, 110.8, 55.3.

3,3'-(diazomethylene)bis(methylbenzene)(2f)

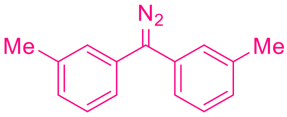

2f

Following the above procedure **C**, the product was obtained as a purple solid, yield: 46% (510 mg), R_f = 0.5 (silica gel, PE/Et_3N = 10:1), column chromatography (silica gel, PE/Et_3N = 20:1). Known compound.[1]

¹H NMR (500 MHz, $CDCl_3$) δ 7.32 – 7.27 (m, 2H), 7.13 (d, *J* = 6.6 Hz, 4H), 7.02 (d, *J* = 7.6 Hz, 2H), 2.38 (s, 6H).

¹³C NMR (126 MHz, $CDCl_3$) δ 138.8, 129.5, 128.9, 126.4, 125.7, 122.4, 21.5.

5,5'-(diazomethylene)bis(1,3-dimethoxybenzene) (2g)

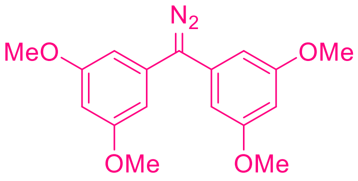

2g

Following the above procedure **C**, the product was obtained as a purple solid, yield: 51% (801 mg), R_f = 0.6 (silica gel, PE/Et_3N = 5:1), column chromatography (silica gel, PE/Et_3N = 10:1).

¹H NMR (400 MHz, $CDCl_3$) δ 6.46 (d, *J* = 2.1 Hz, 4H), 6.31 (t, *J* = 2.1 Hz, 2H), 3.78 (s, 12H).

¹³C NMR (126 MHz, $CDCl_3$) δ 161.3, 131.6, 103.5, 98.0, 55.4.

4-(diazo(phenyl)methyl)benzonitrile (2h)

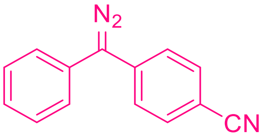

2h

Following the above procedure **C**, the product was obtained as a purple solid, yield: 58% (632 mg), R_f = 0.5 (silica gel, PE/Et_3N = 10:1), column chromatography (silica gel, PE/Et_3N = 20:1). Known compound.[2]

¹H NMR (500 MHz, $CDCl_3$) δ 7.62 – 7.57 (m, 2H), 7.48 – 7.43 (m, 2H), 7.38 – 7.34 (m, 2H), 7.33 – 7.28 (m, 1H), 7.28 – 7.24 (m, 2H).

¹³C NMR (126 MHz, $CDCl_3$) δ 136.2, 132.7, 129.5, 127.4, 127.3, 126.7, 123.4, 119.1, 107.3.

1-(diazo(phenyl)methyl)-4-nitrobenzene (2i)

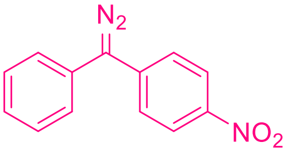

2i

Following the above procedure **C**, the product was obtained as a purple solid, yield: 42% (502 mg), R_f = 0.5 (silica gel, PE/Et_3N = 5:1), column chromatography (silica gel, PE/Et_3N = 10:1). Known compound.[2]

¹H NMR (500 MHz, $CDCl_3$) δ 8.21 – 8.16 (m, 2H), 7.50 – 7.44 (m, 2H), 7.40 – 7.36 (m, 2H), 7.33 (t, *J* = 7.4 Hz, 1H), 7.29 – 7.23 (m, 2H).

¹³C NMR (126 MHz, $CDCl_3$) δ 144.2, 138.8, 129.6, 127.6, 127.2, 127.1, 124.6, 122.9.

1-(diazo(phenyl)methyl)-4-methoxybenzene (2j)

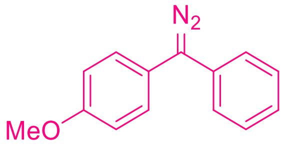

2j

Following the above procedure **C**, the product was obtained as a purple solid, yield: 77% (863 mg), R_f = 0.5 (silica gel, PE/Et_3N = 10:1), column chromatography (silica gel, PE/Et_3N = 10:1). Known compound.[2]

^{1}H NMR (500 MHz, $CDCl_3$) δ 7.40 – 7.35 (m, 2H), 7.31 – 7.27 (m, 2H), 7.22 (dd, *J* = 8.5, 1.1 Hz, 2H), 7.17 – 7.13 (m, 1H), 7.01 – 6.97 (m, 2H), 3.85 (s, 3H).

^{13}C NMR (126 MHz, $CDCl_3$) δ 158.2, 130.4, 129.0, 127.7, 124.9, 124.1, 120.7, 114.8, 55.4.

2-chloro-4-(diazo(4-methoxyphenyl)methyl)-1-fluorobenzene (2k)

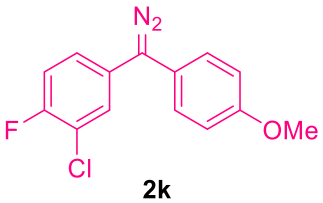

2k

Following the above procedure **C**, the product was obtained as a purple solid, yield: 51% (704 mg), R_f = 0.5 (silica gel, PE/Et_3N = 10:1), column chromatography (silica gel, PE/Et_3N = 20:1).

^{1}H NMR (500 MHz, $CDCl_3$) δ 7.35 – 7.30 (m, 1H), 7.27 – 7.23 (m, 2H), 7.01 – 6.96 (m, 2H), 6.92 (dd, *J* = 10.6, 2.2 Hz, 1H), 6.82 – 6.84 (m, 1H), 3.84 (s, 3H).

^{13}C NMR (126 MHz, $CDCl_3$) δ 158.6 (d, *J* = 246.6 Hz), 158.8, 132.1 (d, *J* = 7.9 Hz), 131.0, 128.3, 119.7 (d, *J* = 3.6 Hz), 119.4, 116.2 (d, *J* = 18.3 Hz), 115.0, 111.3 (d, *J* = 23.8 Hz), 55.4.

^{19}F NMR (471 MHz, $CDCl_3$) δ -114.6.

1,2-dichloro-4-(diazo(4-methoxyphenyl)methyl)benzene (2l)

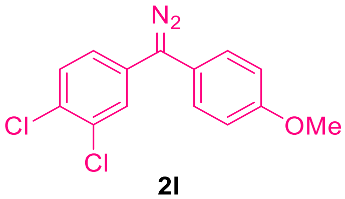

2l

Following the above procedure **C**, the product was obtained as a purple solid, yield: 37% (540 mg), R_f = 0.5 (silica gel, PE/Et_3N = 10:1), column chromatography (silica gel, PE/Et_3N = 20:1). Known compound.[2]

^{1}H NMR (500 MHz, $CDCl_3$) δ 7.27 – 7.24 (m, 2H), 7.05 (t, *J* = 1.8 Hz, 1H), 7.02 – 6.98 (m, 2H), 7.0 – 6.9(m, 2H), 3.84 (s, 3H).

^{13}**C NMR** (126 MHz, $CDCl_3$) δ 159.0, 135.6, 135.0, 128.7, 124.1, 121.1, 118.9, 115.1, 55.4.

1-(diazo(4-methoxyphenyl)methyl)-3,5-difluorobenzene (2m)

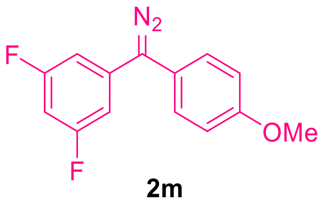

2m

Following the above procedure **C**, the product was obtained as a purple solid, yield: 38% (494 mg), R_f = 0.5 (silica gel, PE/Et_3N = 10:1), column chromatography (silica gel, PE/Et_3N = 20:1).

^{1}**H NMR** (500 MHz, $CDCl_3$) δ 7.30 – 7.27 (m, 2H), 7.02 – 6.98 (m, 2H), 6.67 – 6.60 (m, 2H), 6.52 (tt, J = 8.8, 2.3 Hz, 1H), 3.85 (s, 3H).

^{13}**C NMR** (126 MHz, $CDCl_3$) δ 164.6 (d, J = 13.6 Hz), 162.7 (d, J = 13.7 Hz), 159.0, 135.3 (t, J = 10.7 Hz), 128.8, 119.1, 115.0, 105.7 (dd, J = 21.2, 6.9 Hz), 99.5 (t, J = 25.8 Hz), 55.4.

^{19}**F NMR** (471 MHz, $CDCl_3$) δ -109.2.

1-(diazo(4-fluorophenyl)methyl)-4-methoxybenzene (2n)

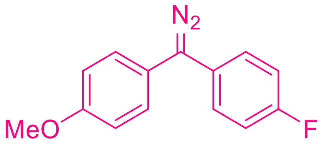

2n

Following the above procedure **C**, the product was obtained as a purple solid, yield: 80% (968 mg), R_f = 0.5 (silica gel, PE/Et_3N = 10:1), column chromatography (silica gel, PE/Et_3N = 20:1).

^{1}**H NMR** (500 MHz, $CDCl_3$) δ 7.23 – 7.20 (m, 2H), 7.20 – 7.15 (m, 2H), 7.11 – 7.05 (m, 2H), 6.99 – 6.95 (m, 2H), 3.84 (s, 3H).

^{13}**C NMR** (126 MHz, $CDCl_3$) δ 160.7 (d, J = 245.7 Hz), 158.1, 127.1, 126.1 (d, J = 7.8 Hz), 120.7, 116.2, 116.0, 114.9, 55.4.

^{19}**F NMR** (471 MHz, $CDCl_3$) δ -117.3.

1-bromo-4-(diazo(4-methoxyphenyl)methyl)benzene (2o)

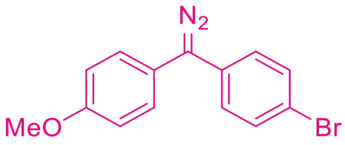

2o

Following the above procedure C, the product was obtained as a purple solid, yield: 56% (847 mg), R_f = 0.5 (silica gel, PE/Et_3N = 10:1), column chromatography (silica gel, PE/Et_3N = 20:1). Known compound.[2]

^{1}**H NMR** (500 MHz, $CDCl_3$) δ 7.48 – 7.43 (m, 2H), 7.27 – 7.22 (m, 2H), 7.07 – 7.02 (m, 2H), 7.00 – 6.95 (m, 2H), 3.84 (s, 3H).

^{13}**C NMR** (126 MHz, $CDCl_3$) δ 158.5, 132.1, 129.8, 127.9, 125.4, 120.1, 117.9, 114.9, 55.4.

4-(diazo(4-methoxyphenyl)methyl)benzonitrile (**2p**)

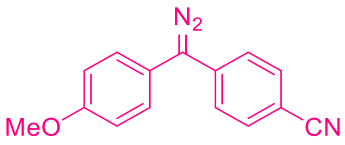

2p

Following the above procedure **C**, the product was obtained as a purple solid, yield: 56% (702 mg), R_f = 0.5 (silica gel, PE/Et_3N = 10:1), column chromatography (silica gel, PE/Et_3N = 20:1). Known compound.[2]

^{1}**H NMR** (400 MHz, $CDCl_3$) δ 7.55 (d, *J* = 8.6 Hz, 2H), 7.33 – 7.27 (m, 2H), 7.14 (d, *J* = 8.6 Hz, 2H), 7.04 – 6.96 (m, 2H), 3.85 (s, 3H).

^{13}**C NMR** (101 MHz, $CDCl_3$) δ 159.3, 137.2, 132.6, 129.2, 122.7, 119.2, 118.6, 115.1, 106.7, 55.4.

1-(diazo(4-(methylsulfonyl)phenyl)methyl)-4-methoxybenzene (**2q**)

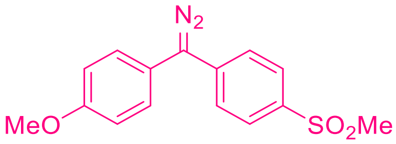

2q

Following the above procedure **C**, the product was obtained as a purple solid, yield: 14% (204 mg), R_f = 0.5 (silica gel, PE/Et_3N = 3:1), column chromatography (silica gel, PE/Et_3N = 5:1). Known compound.[2]

^{1}**H NMR** (500 MHz, $CDCl_3$) δ 7.85 – 7.82 (m, 2H), 7.33 – 7.29 (m, 2H), 7.25 – 7.21 (m, 2H), 7.03 – 6.99 (m, 2H), 3.85 (s, 3H), 3.05 (s, 3H).

^{13}**C NMR** (126 MHz, $CDCl_3$) δ 159.3, 138.4, 135.2, 129.2, 128.1, 122.8, 122.1, 118.7, 115.1, 55.4, 44.7.

ethyl 4-(diazo(4-methoxyphenyl)methyl)benzoate (**2r**)

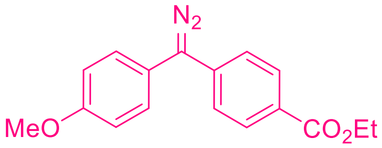

2r

Following the above procedure **C**, the product was obtained as a purple solid, yield: 16% (237 mg), R_f = 0.5 (silica gel, PE/Et_3N = 3:1), column chromatography (silica gel, PE/Et_3N = 5:1). Known compound.[2]

^{1}**H NMR** (500 MHz, $CDCl_3$) δ 8.00 – 7.96 (m, 2H), 7.33 – 7.28 (m, 2H), 7.18 – 7.14 (m,2H), 7.02 – 6.97 (m, 2H), 4.37 (q, *J* = 7.1 Hz, 2H), 3.84 (s, 3H), 1.39 (t, *J* = 7.1 Hz, 3H).

^{13}**C NMR** (126 MHz, $CDCl_3$) δ 166.3, 158.9, 136.4, 130.2, 128.8, 126.1, 122.5, 119.5, 114.9, 60.8, 55.4,

14.3.

4-(diazo(4-methoxyphenyl)methyl)-*N*,*N*-dimethylbenzamide (2s)

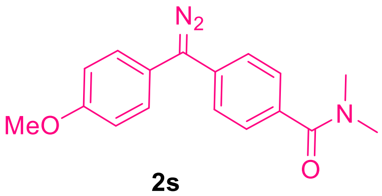

2s

Following the above procedure **C**, the product was obtained as a purple solid, yield: 10% (148 mg), R_f = 0.5 (silica gel, PE/Et_3N = 3:1), column chromatography (silica gel, PE/Et_3N = 5:1). Known compound.[2]

^{1}H NMR (500 MHz, $CDCl_3$) δ 7.42 – 7.38 (m, 2H), 7.29 – 7.24 (m, 2H), 7.18 – 7.14 (m, 2H), 6.98 – 6.94 (m, 2H), 3.81 (d, *J* = 1.2 Hz, 3H), 3.05 (d, *J* = 25.1 Hz, 6H).

^{13}C NMR (126 MHz, $CDCl_3$) δ 171.2, 158.6, 132.7, 132.1, 128.2, 128.1, 123.1, 119.9, 114.9, 55.3.

1-(diazo(4-(trifluoromethoxy)phenyl)methyl)-4-methoxybenzene (2t)

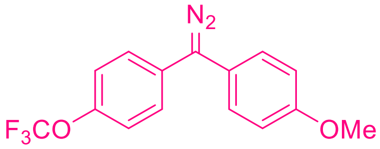

2t

Following the above procedure **C**, the product was obtained as a purple solid, yield: 58% (893 mg), R_f = 0.5 (silica gel, PE/Et_3N = 10:1), column chromatography (silica gel, PE/Et_3N = 15:1). Known compound.[2]

^{1}H NMR (500 MHz, $CDCl_3$) δ 7.28 – 7.24 (m, 2H), 7.23 – 7.17 (m, 4H), 7.01 – 6.96 (m, 2H), 3.84 (s, 3H).

^{13}C NMR (126 MHz, $CDCl_3$) δ 158.5, 146.3, 129.7, 127.8, 125.0, 121.8, 120.5 (d, *J* = 292.5 Hz,), 120.1, 119.5, 114.9, 55.4.

^{19}F NMR (471 MHz, $CDCl_3$) δ -59.1.

1-(diazo(4-(trifluoromethyl)phenyl)methyl)-4-methoxybenzene (2u)

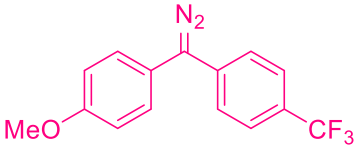

2u

Following the above procedure **C**, the product was obtained as a purple solid, yield: 60% (876 mg), R_f = 0.5 (silica gel, PE/Et_3N = 10:1), column chromatography (silica gel, PE/Et_3N = 20:1). Known compound.[2]

^{1}H NMR (500 MHz, $CDCl_3$) δ 7.56 (d, *J* = 8.3 Hz, 2H), 7.33 – 7.28 (m, 2H), 7.22 (d, *J* = 8.2 Hz, 2H),

7.04 – 6.98 (m, 2H), 3.85 (s, 3H).

^{13}C NMR (126 MHz, $CDCl_3$) δ 158.9, 135.4, 128.6, 125.9 (d, J = 3.8 Hz), 123.0, 119.4, 115.0, 55.4.

^{19}F NMR (471 MHz, $CDCl_3$) δ -62.1.

1-(diazo(4-(trifluoromethyl)phenyl)methyl)-3-methoxybenzene (2v)

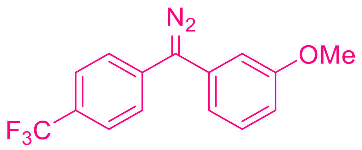

2v

Following the above procedure **C**, the product was obtained as a purple solid, yield: 43% (628 mg), R_f = 0.5 (silica gel, PE/Et_3N = 10:1), column chromatography (silica gel, PE/Et_3N = 20:1).

^{1}H NMR (500 MHz, $CDCl_3$) δ 7.64 – 7.59 (m, 2H), 7.35 (t, J = 8.0 Hz, 3H), 6.96 – 6.80 (m, 3H), 3.83 (s, 3H).

^{13}C NMR (126 MHz, $CDCl_3$) δ 160.4, 134.4, 130.4 (d, J = 7.1 Hz), 129.7, 127.3 (d, J = 15.5 Hz), 126.9 (d, J = 32.6 Hz), 126.0, 125.2, 124.1 (d, J = 3.6 Hz), 124.1, 123.1, 118.4 (d, J = 6.0 Hz), 111.9, 111.6, 55.3.

^{19}F NMR (471 MHz, $CDCl_3$) δ -62.2.

1-(benzyloxy)-4-(diazo(4-(trifluoromethyl)phenyl)methyl)benzene (2w)

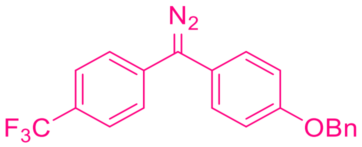

2w

Following the above procedure **C**, the product was obtained as a purple solid, yield: 52% (957 mg), R_f = 0.5 (silica gel, PE/Et_3N = 10:1), column chromatography (silica gel, PE/Et_3N = 20:1).

^{1}H NMR (500 MHz, $CDCl_3$) δ 7.57 (d, J = 8.4 Hz, 2H), 7.47 (d, J = 7.5 Hz, 2H), 7.42 (t, J = 7.5 Hz, 2H), 7.36 (t, J = 7.2 Hz, 1H), 7.32 – 7.28 (m, 2H), 7.23 (d, J = 8.4 Hz, 2H), 7.07 (t, J = 5.7 Hz, 2H), 5.12 (s, 2H).

^{13}C NMR (126 MHz, $CDCl_3$) δ 158.0, 136.6, 135.3 (d, J = 1.4 Hz), 128.6 (d, J = 12.6 Hz), 128.1, 127.5, 126.4, 126.1, 126.4, 126.1, 125.89 (dd, J = 8.0, 4.0 Hz), 123.1, 119.8, 115.9, 70.2.

^{19}F NMR (471 MHz, $CDCl_3$) δ -63.3.

1-(diazo(4-methoxyphenyl)methyl)-4-nitrobenzene (2x)

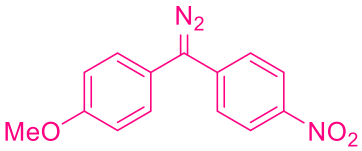

2x

Following the above procedure **C**, the product was obtained as a purple solid, yield: 59% (794 mg), R_f = 0.5 (silica gel, PE/Et_3N = 5:1), column chromatography (silica gel, PE/Et_3N = 10:1). Known compound.[2]

^{1}H NMR (500 MHz, $CDCl_3$) δ 8.15 (dd, J = 13.4, 9.0 Hz, 2H), 7.44 – 7.37 (m, 1H), 7.37 – 7.31 (m, 1H), 7.16 (d, J = 9.0 Hz, 1H), 7.11 – 7.00 (m, 3H), 3.86 (s, 3H).

^{13}C NMR (126 MHz, $CDCl_3$) δ 159.5, 143.8, 139.8, 130.9, 129.5, 124.6, 124.4, 122.2, 121.6, 118.4, 115.2, 55.4.

1-(diazo(4-methoxyphenyl)methyl)-3-nitrobenzene (**2y**)

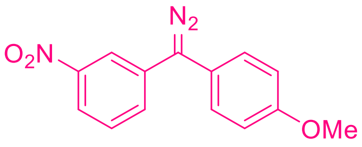

2y

Following the above procedure **C**, the product was obtained as a purple solid, yield: 24% (324 mg), R_f = 0.5 (silica gel, PE/Et_3N = 5:1), column chromatography (silica gel, PE/Et_3N = 10:1). Known compound.[2]

^{1}H NMR (400 MHz, $CDCl_3$) δ 7.97 (t, J = 1.9 Hz, 1H), 7.91 (dd, J = 8.0, 1.0 Hz, 1H), 7.49 (t, J = 8.0 Hz, 1H), 7.42 (d, J = 8.0 Hz, 1H), 7.29 (d, J = 8.8 Hz, 2H), 7.02 (t, J = 5.9 Hz, 2H), 3.86 (s, 3H).

1-(diazo(4-nitrophenyl)methyl)-4-methylbenzene (**2z**)

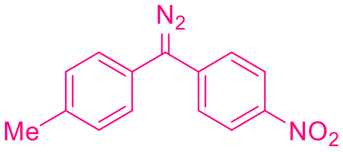

2z

Following the above procedure **C**, the product was obtained as a purple solid, yield: 46% (582 mg), R_f = 0.5 (silica gel, PE/Et_3N = 5:1), column chromatography (silica gel, PE/Et_3N = 5:1). Known compound.[2]

^{1}H NMR (500 MHz, $CDCl_3$) δ 8.22 – 8.14 (m, 2H), 7.29 (s, 4H), 7.25 – 7.19 (m, 2H), 2.41 (s, 3H).

^{13}C NMR (126 MHz, $CDCl_3$) δ 144.0, 139.3, 137.9, 130.3, 127.4, 124.6, 123.8, 122.6, 121.4, 21.2.

4-(benzo[d][1,3]dioxol-5-yl(diazo)methyl)benzonitrile (**2aa**)

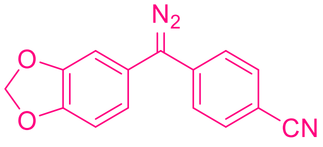

2aa

Following the above procedure **C**, the product was obtained as a purple solid, yield: 23% (303 mg), R_f = 0.5 (silica gel, PE/Et_3N = 5:1), column chromatography (silica gel, PE/Et_3N = 5:1).

^{1}H NMR (400 MHz, $CDCl_3$) δ 7.59 – 7.53 (m, 2H), 7.18 – 7.13 (m, 2H), 6.91 – 6.82 (m, 3H), 6.02 (s, 2H).

^{13}**C NMR** (101 MHz, $CDCl_3$) δ 148.7, 147.4, 136.8, 132.6, 122.9, 121.6, 120.3, 119.1, 109.3, 108.1, 106.9, 101.5.

1-benzhydryl-2-(tert-butyl)-2,3-dihydro-1H-benzo[c][1,2]azaborole (**3a**)

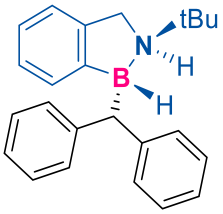

3a

Following the above procedure **D**, isolated yield: 90%, (30.7 mg), white solid (mp: 105.1 – 107.9 °C), R_f = 0.4 (silica gel, hexane/DCM = 5:1, v/v), column chromatography (silica gel, hexane/DCM = 3:1, v/v).

^{1}**H NMR** (500 MHz, $CDCl_3$) δ 7.37 – 7.32 (m, 2H), 7.29 – 7.25 (m, 2H), 7.25 – 7.20 (m, 2H), 7.15 (dd, *J* = 8.2, 1.4 Hz, 2H), 7.14 – 7.03 (m, 3H), 7.00 (d, *J* = 7.5 Hz, 1H), 6.95 (t, *J* = 7.2 Hz, 1H), 6.59 (d, *J* = 7.3 Hz, 1H), 4.13 (dd, *J* = 15.4, 3.3 Hz, 1H), 4.05 (dd, *J* = 15.4, 7.9 Hz, 1H), 3.53 (s, 1H), 3.46 (d, *J* = 6.4 Hz, 1H), 1.13 (s, 9H).

^{13}**C NMR** (126 MHz, $CDCl_3$) δ 148.5, 146.0, 139.5, 130.2, 129.8, 128.1, 128.0, 127.7, 126.2, 125.1, 124.8, 124.1, 120.1, 57.7, 52.1, 26.5.

^{11}**B NMR** (160 MHz, $CDCl_3$) δ -1.3.

HRMS (ESI) m/z: $[M-H]^-$ Calcd. for $C_{24}H_{27}BN$ 340.2242; Found: 340.2249.

Following the above procedure **F**, isolated yield: 45%, (30.6 mg).

HPLC analysis: DAICEL CHIRALCEL IG, hexane/isopropanol = 98/2, 0.5 mL/min, λ = 267 nm, t_1 = 9.542 min, t_2 = 10.618 min, 91% ee.

$[\alpha]^{25}_D$: +17.67 (*c* 0.3, $CHCl_3$).

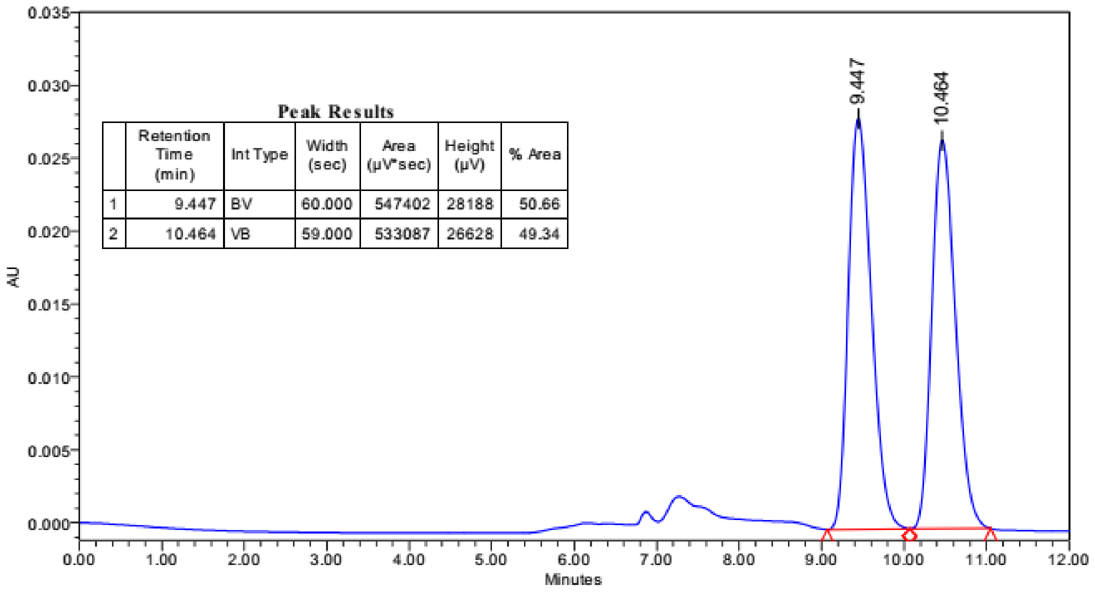

Peak Results

	Retention Time (min)	Int Type	Width (sec)	Area (μV*sec)	Height (μV)	% Area
1	9.447	BV	60.000	547402	28188	50.66
2	10.464	VB	59.000	533087	26628	49.34

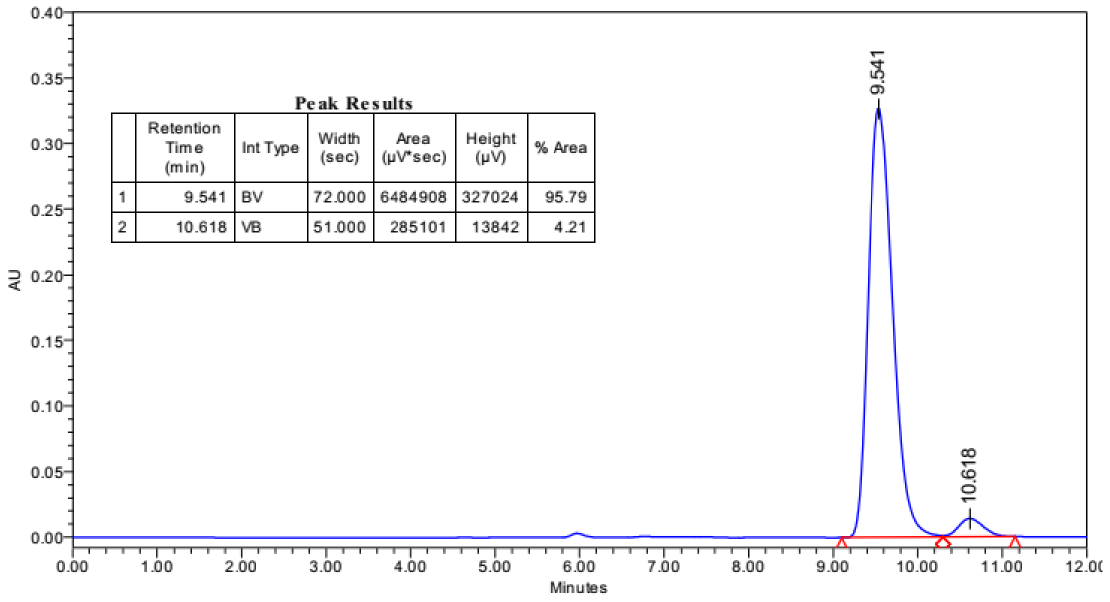

Peak Results

	Retention Time (min)	Int Type	Width (sec)	Area (μV*sec)	Height (μV)	% Area
1	9.541	BV	72.000	6484908	327024	95.79
2	10.618	VB	51.000	285101	13842	4.21

1-(bis(4-methoxyphenyl)methyl)-2-(tert-butyl)-2,3-dihydro-1H-benzo[c][1,2]azaborole (3b)

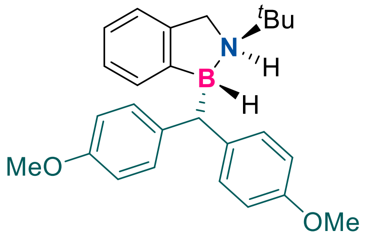

3b

Following the above procedure **D**, isolated yield: 75%, (30.1 mg), white solid (mp: 99.8 – 101.7 °C), R_f = 0.3 (silica gel, hexane/DCM = 3:1, v/v), column chromatography (silica gel, hexane/DCM = 2:1, v/v).

^{1}H NMR (400 MHz, Acetone-d_6) δ 7.29 (d, J = 8.1 Hz, 2H), 7.10 (d, J = 7.9 Hz, 2H), 7.06 – 6.96 (m, 2H), 6.85 (dd, J = 13.2, 7.9 Hz, 3H), 6.76 (d, J = 8.5 Hz, 2H), 6.67 (d, J = 7.1 Hz, 1H), 4.35 (d, J = 7.2 Hz, 2H), 4.06 (s, 1H), 3.77 (s, 3H), 3.75 (s, 3H), 3.28 (d, J = 6.5 Hz, 1H), 1.15 (s, 9H).

^{13}C NMR (101 MHz, Acetone-d_6) δ 157.4, 142.3, 141.3, 140.0, 131.4, 130.2, 129.5, 126.1, 125.3, 120.8, 113.7, 113.8, 58.2, 55.2, 55.0, 52.6, 26.4.

^{11}B NMR (128 MHz, Acetone-d_6) δ -0.0.

HRMS (ESI) m/z: $[M+H]^+$ Calcd. for $C_{26}H_{33}BNO_2$ 402.2599; Found: 402.2593.

HPLC analysis: DAICEL CHIRALCEL IG, hexane/isopropanol = 80/20, 0.5 mL/min, λ = 267 nm, t_1 = 11.466 min, t_2 = 13.399 min, 87% ee.

$[\alpha]^{25}_{D}$: +1.82 (c 0.33, $CHCl_3$).

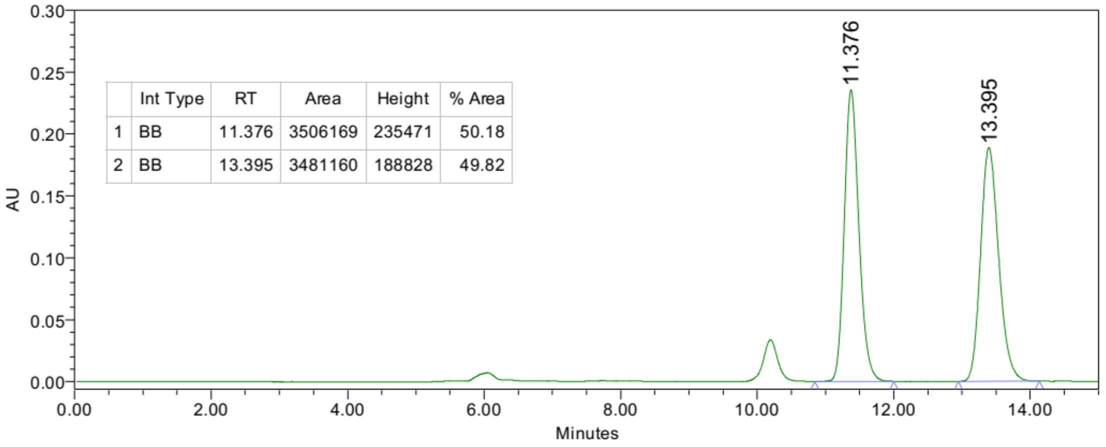

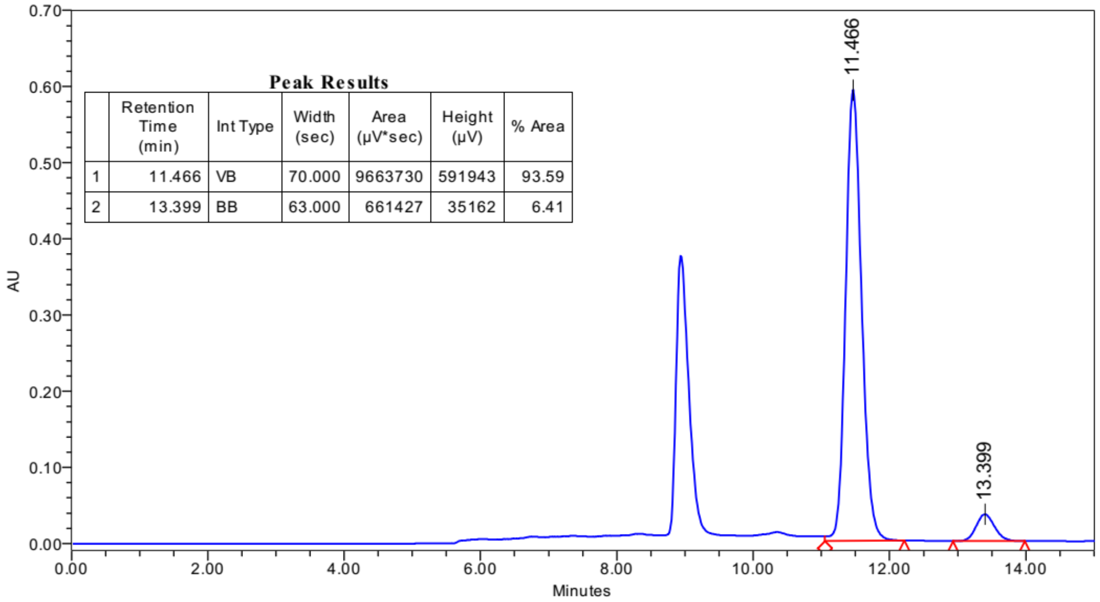

2-(tert-butyl)-1-(di-p-tolylmethyl)-2,3-dihydro-1H-benzo[c][1,2]azaborole (3c)

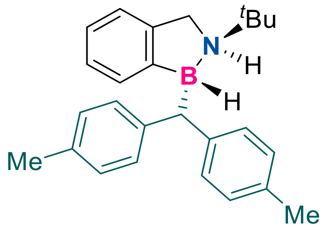

3c

Following the above procedure **D**, isolated yield: 90%, (33.2 mg), white solid (mp: 144.8 – 146.4 °C), R_f = 0.4 (silica gel, hexane/DCM = 5:1, v/v), column chromatography (silica gel, hexane/DCM = 3:1, v/v).

^{1}H NMR (400 MHz, Acetone-d_6) δ 7.27 (d, J = 7.8 Hz, 2H), 7.13 – 6.95 (m, 8H), 6.85 (t, J = 7.2 Hz, 1H), 6.67 (d, J = 7.2 Hz, 1H), 4.34 (d, J = 4.2 Hz, 2H), 4.05 (s, 1H), 3.30 (d, J = 6.9 Hz, 1H), 2.29 (s, 3H), 2.27 (s, 3H), 1.13 (s, 9H).

^{13}C NMR (126 MHz, $CDCl_3$) δ 145.5, 143.0, 139.5, 133.8, 133.1,130.0, 129.9, 128.7, 127.7, 126.2, 123.0, 120.1, 57.6, 52.1, 26.6, 21.0, 20.9.

^{11}B NMR (160 MHz, $CDCl_3$) δ -2.0.

HRMS (ESI) m/z: $[M+Cl]^-$ Calcd. for $C_{26}H_{32}BNCl$ 404.2322; Found: 404.2333.

HPLC analysis: DAICEL CHIRALCEL AD-H, hexane/isopropanol = 98/2, 0.5 mL/min, λ = 267 nm, t_1 = 8.554 min, t_2 = 10.820 min, 90% ee.

$[\alpha]^{25}_D$: +23.03 (c 0.33, $CHCl_3$).

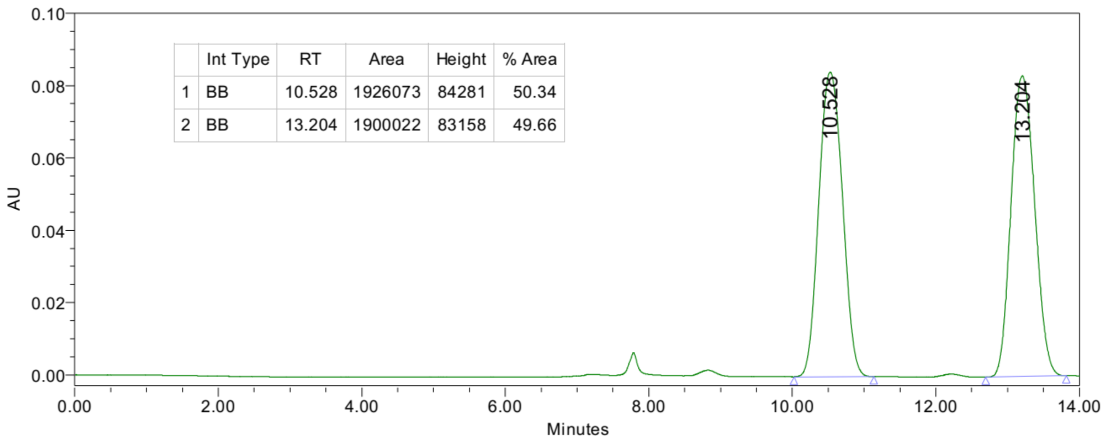

	Int Type	RT	Area	Height	% Area
1	BB	10.528	1926073	84281	50.34
2	BB	13.204	1900022	83158	49.66

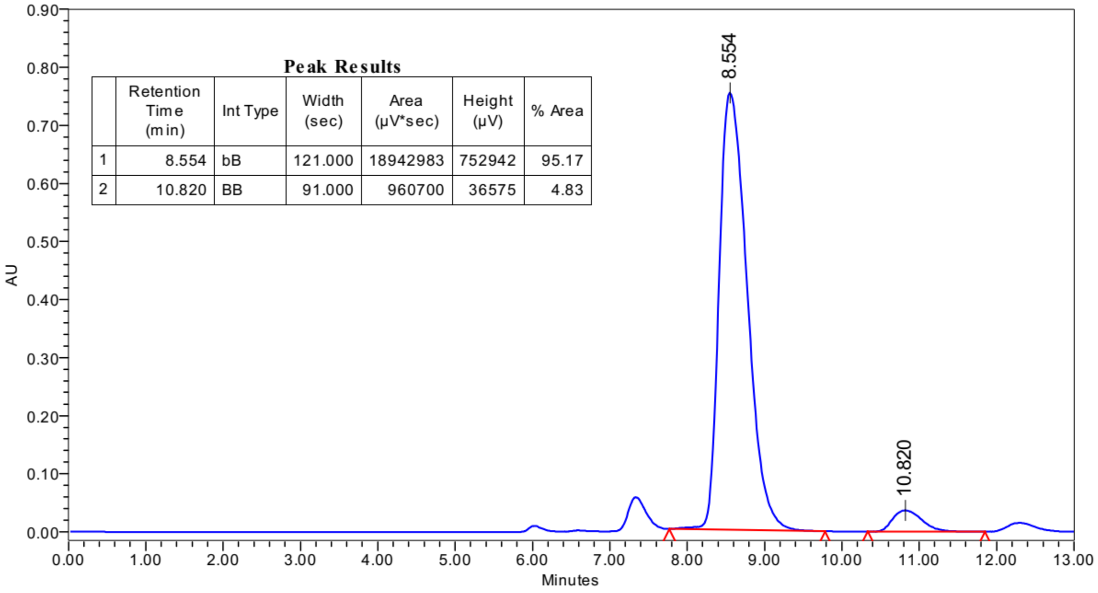

1-(bis(4-fluorophenyl)methyl)-2-(tert-butyl)-2,3-dihydro-1H-benzo[c][1,2]azaborole (**3d**)

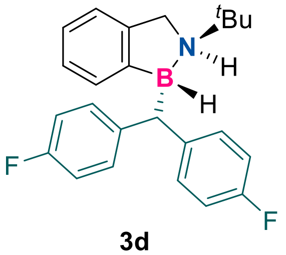

3d

Following the above procedure **D**, isolated yield: 76%, (28.7 mg), white solid (mp: 125.8 – 128.1 ºC), R_f = 0.4 (silica gel, hexane/DCM = 5:1, v/v), column chromatography (silica gel, hexane/DCM = 3:1, v/v).

^{1}H NMR (500 MHz, $CDCl_3$) δ 7.28 – 7.22 (m, 2H), 7.10 – 7.03 (m, 3H), 7.02 – 6.94 (m, 4H), 6.94 – 6.87 (m, 2H), 6.58 (d, J = 7.2 Hz, 1H), 4.15 (dd, J = 15.4, 2.8 Hz, 1H), 4.05 (dd, J = 15.5, 7.9 Hz, 1H), 3.40 (d, J = 5.9 Hz, 2H), 1.15 (s, 9H).

^{13}C NMR (126 MHz, $CDCl_3$) δ 161.5 (d, J = 49.6 Hz), 159.6 (d, J = 48.6 Hz), 143.8, 141.5, 139.4, 131.2 (d, J = 7.5 Hz), 129.7, 128.9 (d, J = 7.4 Hz), 126.4, 125.4, 120.3, 114.8 (dd, J = 20.6, 12.1 Hz), 57.9, 52.2, 26.6.

^{11}B NMR (160 MHz, $CDCl_3$) δ -1.6.

^{19}F NMR (471 MHz, $CDCl_3$) δ -118.7, -119.9.

HRMS (ESI) m/z: $[M+H]^+$ Calcd. for $C_{24}H_{27}BF_2N$ 378.2199; Found: 378.2201.

HPLC analysis: DAICEL CHIRALCEL AD-H, hexane/isopropanol = 98/2, 0.5 mL/min, λ = 267 nm, t_1 = 9.455 min, t_2 = 10.568 min, 78% ee.

$[\alpha]^{25}_D$: +18.13 (c 0.32, $CHCl_3$).

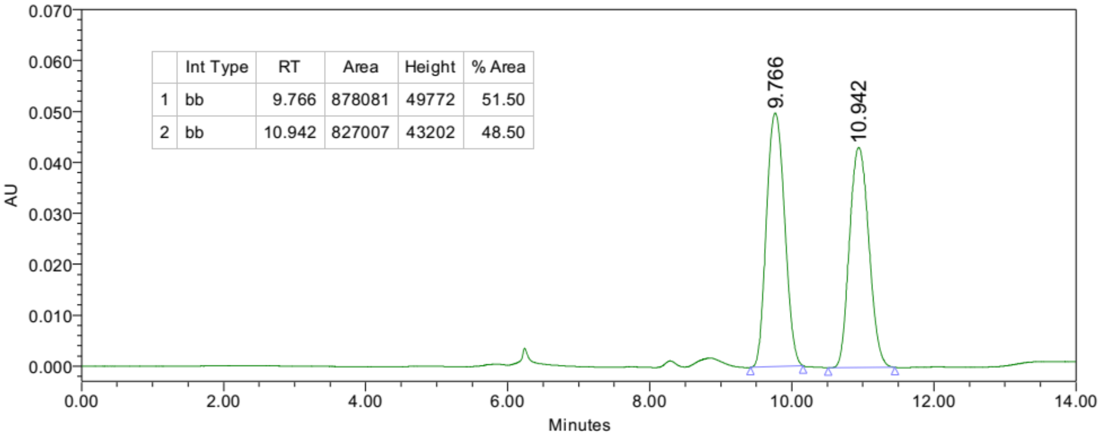

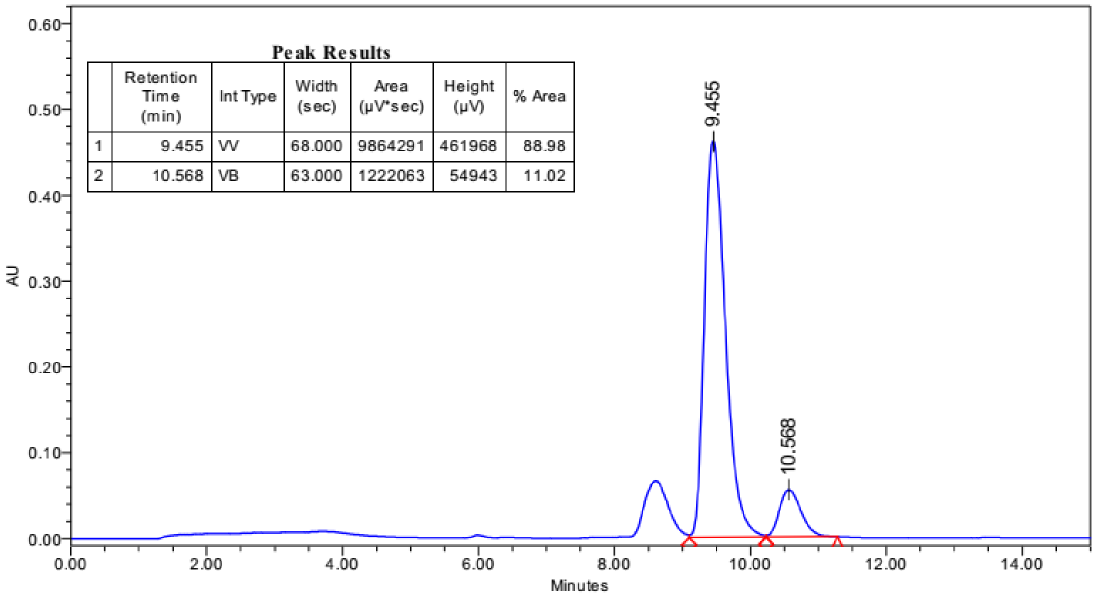

1-(bis(3-methoxyphenyl)methyl)-2-(tert-butyl)-2,3-dihydro-1H-benzo[c][1,2]azaborole (**3e**)

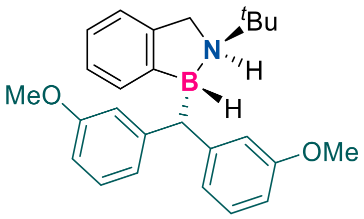

3e

Following the above procedure **D**, isolated yield: 85%, (34.1 mg), white solid (mp: 156.5 – 158.2 °C), R_f = 0.3 (silica gel, hexane/EtOAc = 3:1, v/v), column chromatography (silica gel, hexane/EtOAc = 2:1, v/v).

^{1}H NMR (500 MHz, Acetone-d_6) δ 7.17 (t, J = 8.1 Hz, 1H), 7.11 (t, J = 7.8 Hz, 1H), 7.05 (d, J = 7.4 Hz, 1H), 7.02 – 6.98 (m, 3H), 6.87 (t, J = 7.2 Hz, 1H), 6.83 (d, J = 7.5 Hz, 1H), 6.73 – 6.64 (m, 4H), 4.33 (d, J = 5.1 Hz, 2H), 4.13 (s, 1H), 3.77 (s, 3H), 3.61 (s, 3H), 3.41 (d, J = 6.5 Hz, 1H), 1.18 (s, 9H).

^{13}C NMR (126 MHz, Acetone-d_6) δ 160.3, 159.9, 151.2, 148.9, 141.3, 130.1, 129.1, 129.0, 126.1, 125.4,

123.1, 121.2, 120.8, 115.9, 114.7, 110.9, 109.6, 58.2, 54.9, 54.7, 52.6, 26.2.

^{11}B NMR (160 MHz, Acetone-d_6) δ -1.7.

HRMS (ESI) m/z: $[M+H]^+$ Calcd. for $C_{26}H_{33}BNO_2$ 402.2599; Found: 402.2593.

HPLC analysis: DAICEL CHIRALCEL IG, hexane/isopropanol = 95/5, 0.5 mL/min, λ = 267 nm, t_1 = 20.309 min, t_2 = 21.711 min, 84% ee.

[α]^{25}$_D$: +5.14 (*c* 0.35, $CHCl_3$).

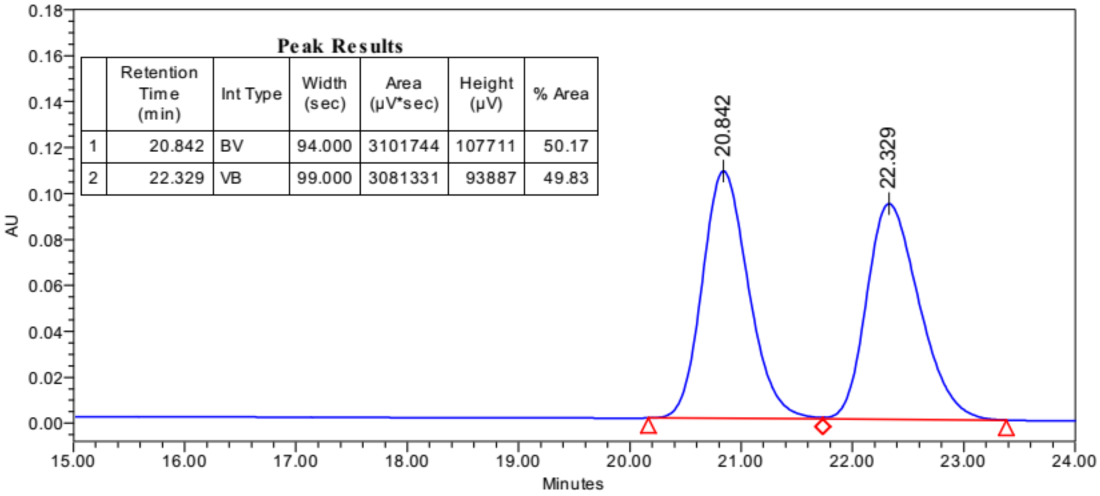

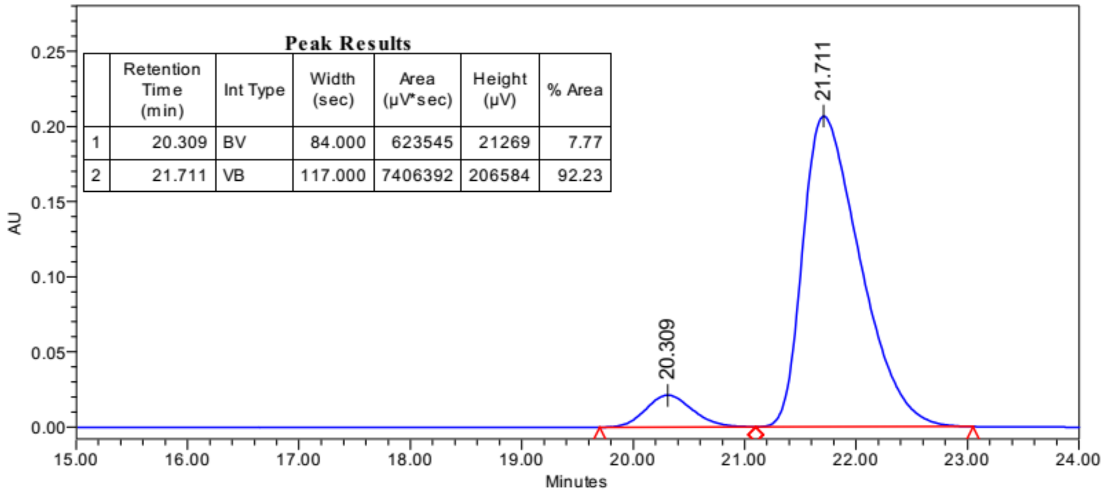

2-(tert-butyl)-1-(di-m-tolylmethyl)-2,3-dihydro-1H-benzo[c][1,2]azaborole (3f)

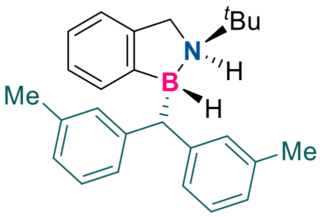

3f

Following the above procedure **D**, isolated yield: 80%, (29.6 mg), white solid (mp: 120.1 – 122.5 ºC), R_f = 0.4 (silica gel, hexane/EtOAc = 5:1, v/v), column chromatography (silica gel, hexane/EtOAc = 3:1, v/v).

^{1}H NMR (500 MHz, Acetone-d_6) δ 7.26 (s, 1H), 7.20 (d, J = 7.7 Hz, 1H), 7.13 (dd, J = 13.7, 6.1 Hz, 1H), 7.10 – 7.07 (m, 2H), 7.06 – 6.97 (m, 3H), 6.91 (d, J = 7.0 Hz, 2H), 6.84 (t, J = 7.2 Hz, 1H), 6.66 (d, J = 7.3 Hz, 1H), 4.41 – 4.31 (m, 2H), 4.07 (s, 1H), 3.28 (d, J = 7.2 Hz, 1H), 2.31 (s, 3H), 2.24 (s, 3H), 1.11 (s, 9H).

^{13}C NMR (126 MHz, Acetone-d_6) δ 149.9, 147.5, 141.3, 137.5, 137.1, 131.6, 130.1, 129.5, 128.3, 128.0, 127.7, 126.0, 125.8, 125.6, 125.3, 125.1, 120.7, 58.1, 52.4, 26.2, 21.4, 21.3.

^{11}B NMR (160 MHz, Acetone-d_6) δ -2.3.

HRMS (ESI) m/z: $[M+H]^+$ Calcd. for $C_{26}H_{33}BN$ 370.2701; Found: 370.2706.

HPLC analysis: DAICEL CHIRALCEL IB N-3, hexane/isopropanol = 98/2, 0.5 mL/min, λ = 267 nm, t_1 = 9.386 min, t_2 = 10.208 min, 86% ee.

$[\alpha]^{25}_{D}$: +3.64 (c 0.33, $CHCl_3$).

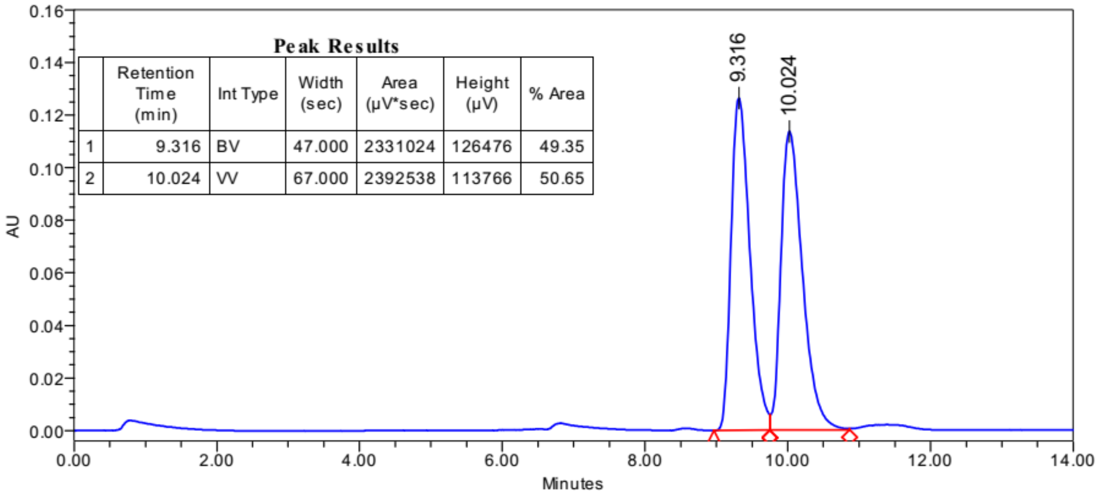

	Retention Time (min)	Int Type	Width (sec)	Area (µV*sec)	Height (µV)	% Area
1	9.316	BV	47.000	2331024	126476	49.35
2	10.024	VV	67.000	2392538	113766	50.65

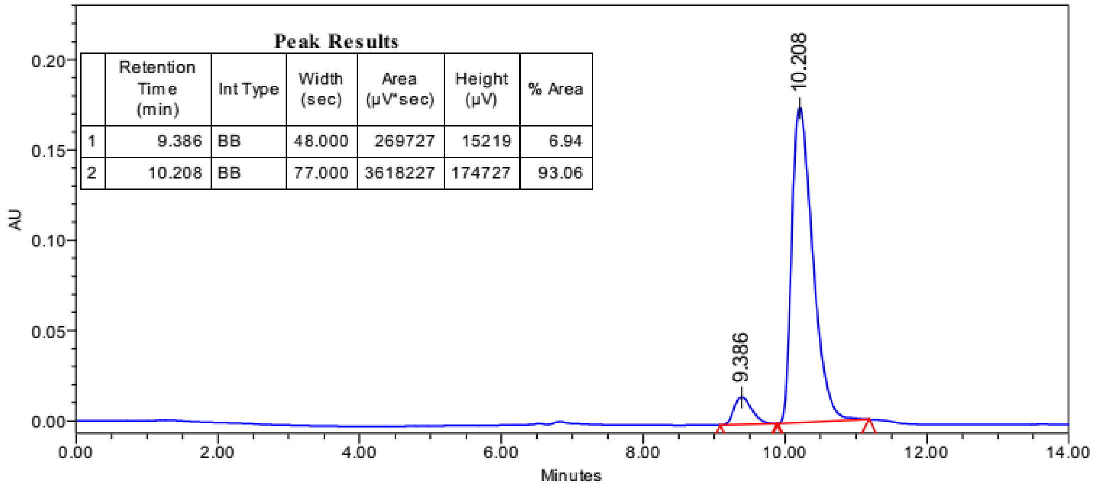

	Retention Time (min)	Int Type	Width (sec)	Area (µV*sec)	Height (µV)	% Area
1	9.386	BB	48.000	269727	15219	6.94
2	10.208	BB	77.000	3618227	174727	93.06

1-(bis(3,5-dimethoxyphenyl)methyl)-2-(tert-butyl)-2,3-dihydro-1*H*-benzo[c][1,2]azaborole (3g)

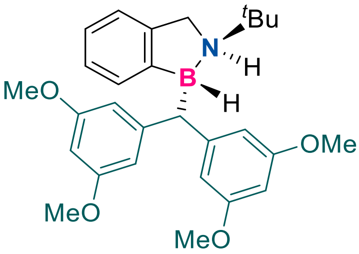

3g

Following the above procedure **D**, isolated yield: 76%, (35.1 mg), colorless liquid, R_f = 0.3 (silica gel, hexane/EtOAc = 2:1, v/v), column chromatography (silica gel, hexane/EtOAc = 1:1, v/v).

^{1}H NMR (500 MHz, Acetone-d_6) δ 7.05 (d, J = 7.4 Hz, 1H), 7.01 (td, J = 7.3, 1.1 Hz, 1H), 6.91 (t, J = 7.2 Hz, 1H), 6.76 (d, J = 7.2 Hz, 1H), 6.61 (d, J = 2.3 Hz, 2H), 6.34 (d, J = 2.3 Hz, 2H), 6.27 (t, J = 2.3 Hz, 1H), 6.23 (t, J = 2.3 Hz, 1H), 4.37 – 4.27 (m, 2H), 4.18 (s, 1H), 3.77 (s, 6H), 3.62 (s, 6H), 3.38 (d, J = 6.5 Hz, 1H), 1.22 (s, 9H).

^{13}C NMR (101 MHz, Acetone-d_6) δ161.2, 160.9, 151.7, 149.4, 130.2, 126.1, 125.4, 120.8, 108.5, 107.0, 97.6, 96.3, 58.2, 55.0, 54.8, 52.8, 26.3.

^{11}B NMR (160 MHz, Acetone-d_6) δ -1.8.

HRMS (ESI) m/z: $[M+H]^+$ Calcd. for $C_{28}H_{37}BNO_4$ 462.281; Found: 462.2799.

HPLC analysis: DAICEL CHIRALCEL AD-H, hexane/isopropanol = 90/10, 0.5 mL/min, λ = 267 nm, t_1 = 11.569 min, t_2 = 18.557 min, 80% ee.

$[\alpha]^{25}_D$: +0.88 (c 0.34, $CHCl_3$).

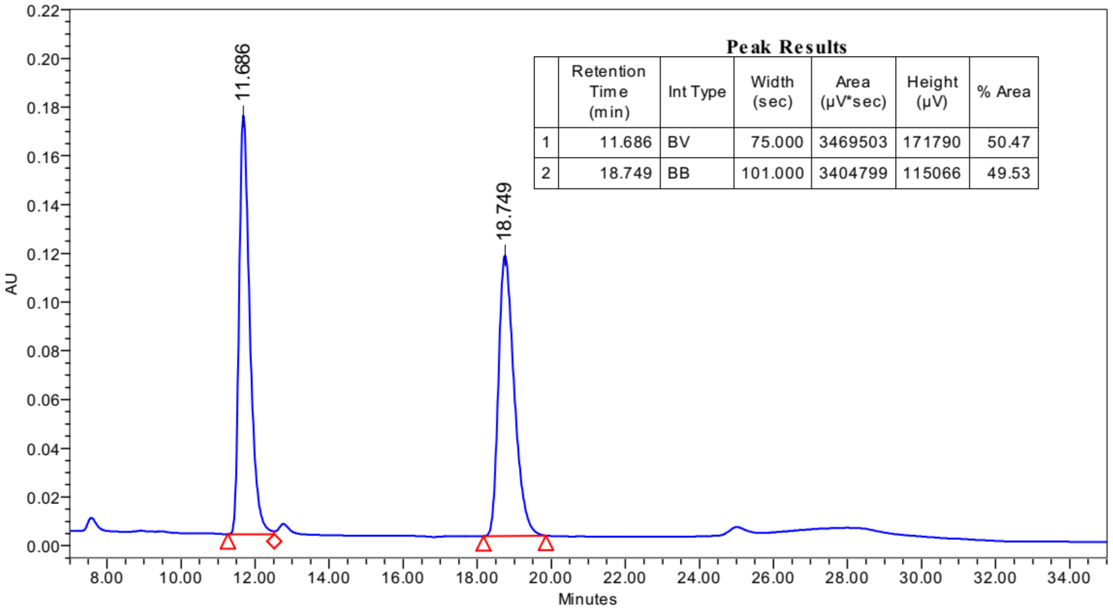

Peak Results

	Retention Time (min)	Int Type	Width (sec)	Area (μV*sec)	Height (μV)	% Area
1	11.686	BV	75.000	3469503	171790	50.47
2	18.749	BB	101.000	3404799	115066	49.53

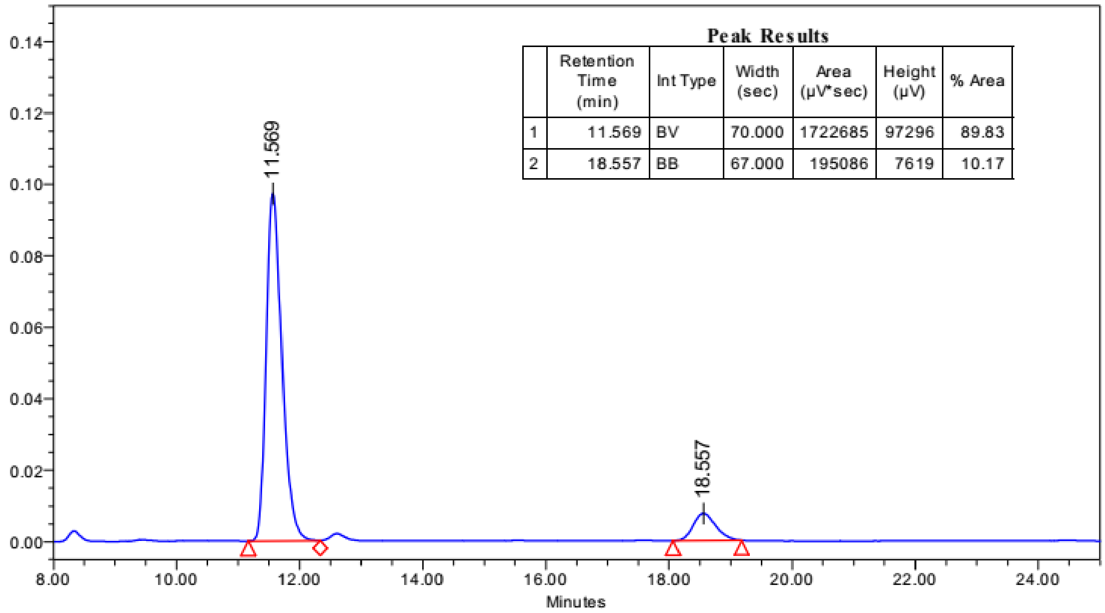

(R)-4-((2-(tert-butyl)-2,3-dihydro-1*H*-benzo[c][1,2]azaborol-1-yl)(phenyl)methyl)benzonitrile (3h)

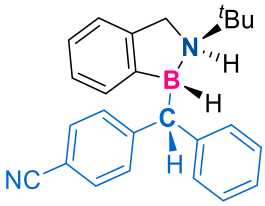

3h

Following the above procedure **E**, isolated yield: 80%, (24.5 mg), white solid (mp: 78.3 – 80.6 °C), R_f = 0.3 (silica gel, hexane/DCM = 3:1, v/v), column chromatography (silica gel, hexane/DCM = 3:1, v/v).

^{1}H NMR (500 MHz, $CDCl_3$) δ 7.55 – 7.51 (m, 2H), 7.40 (d, *J* = 8.3 Hz, 2H), 7.22 (t, *J* = 7.3 Hz, 2H), 7.19 – 7.15 (m, 1H), 7.11 – 7.05 (m, 1H), 7.00 – 6.91 (m, 4H), 6.33 (d, *J* = 7.3 Hz, 1H), 4.09 (dd, *J* = 15.5, 4.6 Hz, 1H), 3.85 – 3.76 (m, 2H), 3.45 (s, 1H), 1.26 (s, 9H).

^{13}C NMR (126 MHz, $CDCl_3$) δ 154.0, 143.8, 139.5, 131.6, 130.0, 129.5, 128.6, 128.3, 126.4, 125.6, 125.5, 120.4, 107.0, 58.0, 52.7, 26.6.

^{11}B NMR (160 MHz, $CDCl_3$) δ -2.0.

HRMS (ESI) m/z: $[M+H]^+$ Calcd. for $C_{25}H_{28}BN_2$ 367.234; Found: 367.2335.

HPLC analysis: DAICEL CHIRALCEL AD-H, hexane/isopropanol = 98/2, 0.5 mL/min, λ = 267 nm, t_1 = 18.946 min, t_2 = 24.565 min, t_3 = 26.027 min, t_4 = 37.825 min, 92% ee, dr = 21.7:1.

$[\alpha]^{25}_D$: +9.67 (*c* 0.3, $CHCl_3$).

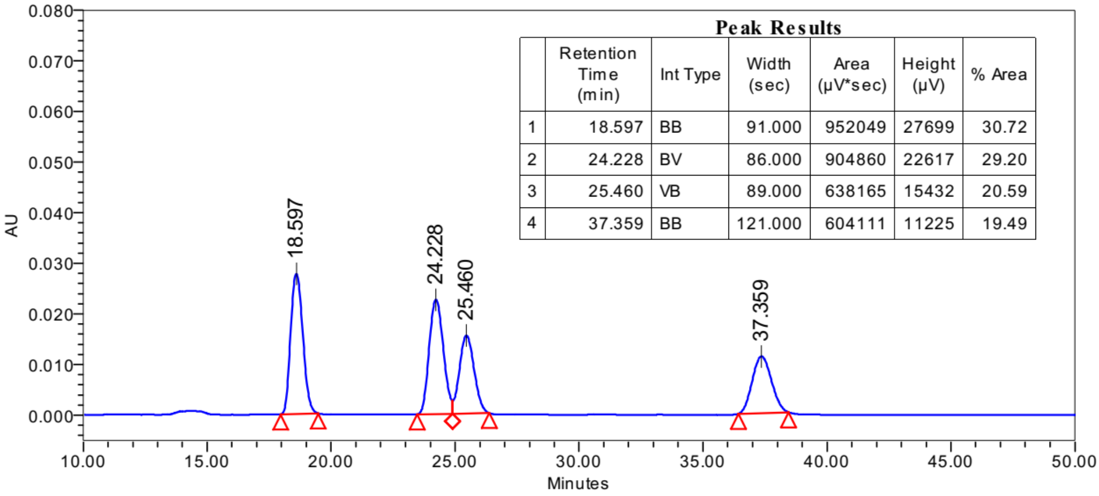

	Retention Time (min)	Int Type	Width (sec)	Area (μV*sec)	Height (μV)	% Area
1	18.597	BB	91.000	952049	27699	30.72
2	24.228	BV	86.000	904860	22617	29.20
3	25.460	VB	89.000	638165	15432	20.59
4	37.359	BB	121.000	604111	11225	19.49

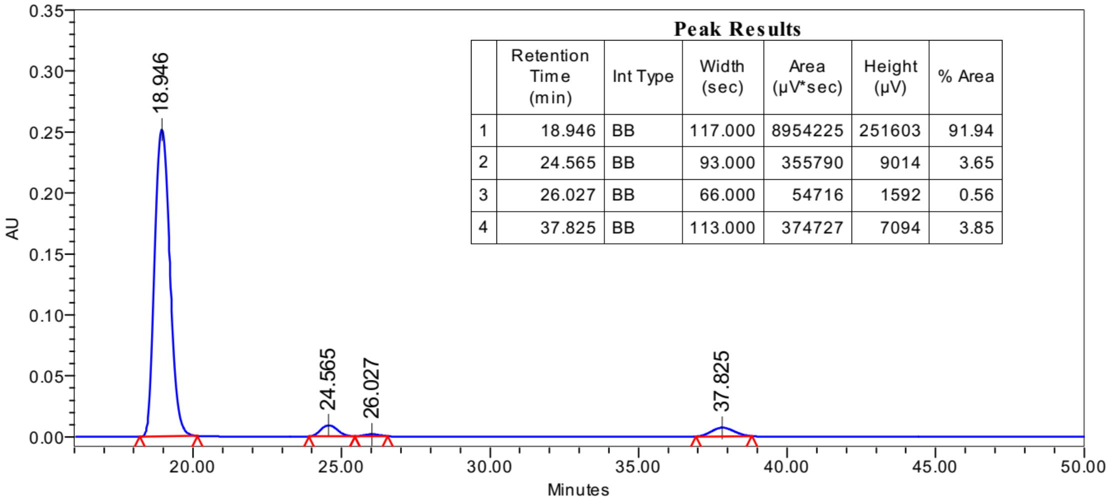

	Retention Time (min)	Int Type	Width (sec)	Area (μV*sec)	Height (μV)	% Area
1	18.946	BB	117.000	8954225	251603	91.94
2	24.565	BB	93.000	355790	9014	3.65
3	26.027	BB	66.000	54716	1592	0.56
4	37.825	BB	113.000	374727	7094	3.85

(R)-2-(tert-butyl)-1-((4-nitrophenyl)(phenyl)methyl)-2,3-dihydro-1H-benzo[c][1,2]azaborole (3i)

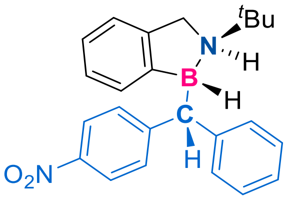

3i

Following the above procedure **E**, isolated yield: 70%, (27.0 mg), white solid (mp: 117.9 – 119.6 °C), R_f = 0.3 (silica gel, hexane/EtOAc = 2:1, v/v), column chromatography (silica gel, hexane/EtOAc = 1:1, v/v).

^{1}H NMR (400 MHz, $CDCl_3$) δ 8.11 (d, *J* = 8.8 Hz, 2H), 7.42 (d, *J* = 8.7 Hz, 2H), 7.24 – 7.15 (m, 3H), 7.09 (t, *J* = 7.4 Hz, 1H), 7.00 – 6.87 (m, 4H), 6.32 (d, *J* = 7.3 Hz, 1H), 4.08 (dd, *J* = 15.5, 4.6 Hz, 1H), 3.89 – 3.74 (m, 2H), 3.46 (s, 1H), 1.27 (s, 9H).

^{13}C NMR (101 MHz, $CDCl_3$) δ 156.6, 144.6, 143.7, 139.5, 130.0, 129.5, 128.8, 128.2, 126.5, 125.8, 125.7, 123.1, 120.4, 58.2, 52.8, 26.6.

^{11}B NMR (128 MHz, CDCl$_3$) δ -0.7.

HRMS (ESI) m/z: $[M+H]^+$ Calcd. for $C_{24}H_{28}BN_2O_2$ 387.2239; Found: 387.2233.

HPLC analysis: DAICEL CHIRALCELAD-H, hexane/isopropanol = 95/5, 0.5 mL/min, λ = 267 nm, t_1 = 12.606 min, t_2 = 13.903 min, t_3 = 16.760 min, 18.917 min, 92% ee, dr = 10.2:1.

[α]^{25}$_D$: +3.24 (*c* 0.34, CHCl$_3$).

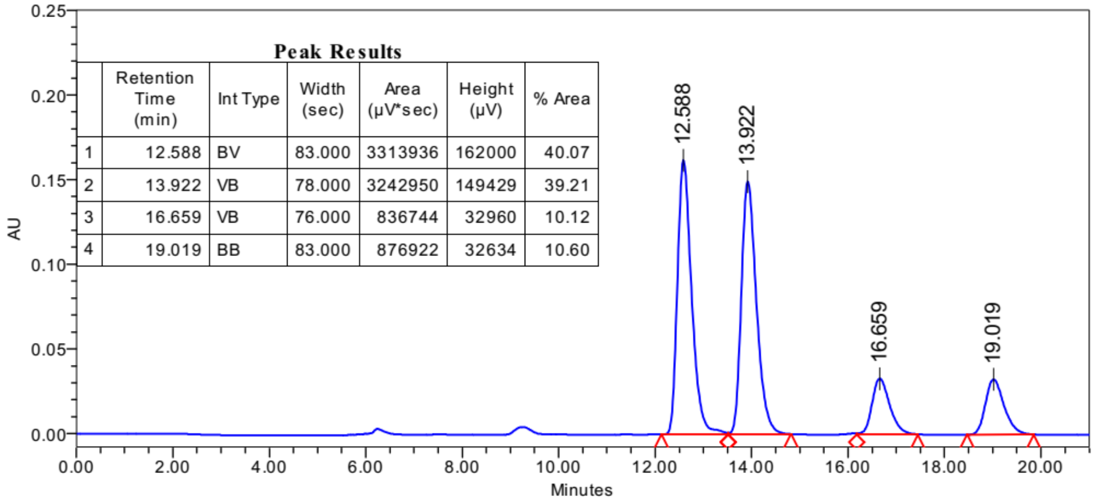

Peak Results

	Retention Time (min)	Int Type	Width (sec)	Area (μV*sec)	Height (μV)	% Area
1	12.588	BV	83.000	3313936	162000	40.07
2	13.922	VB	78.000	3242950	149429	39.21
3	16.659	VB	76.000	836744	32960	10.12
4	19.019	BB	83.000	876922	32634	10.60

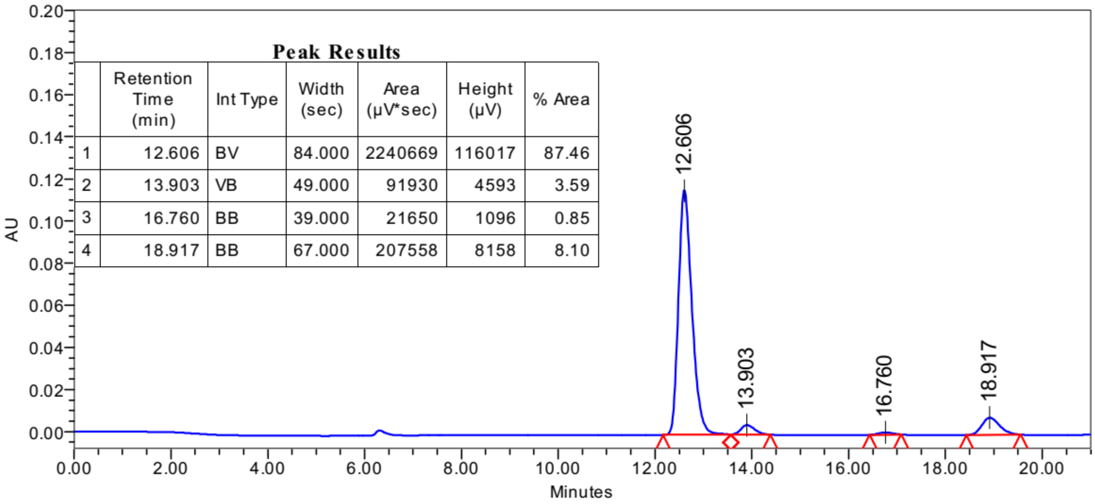

Peak Results

	Retention Time (min)	Int Type	Width (sec)	Area (μV*sec)	Height (μV)	% Area
1	12.606	BV	84.000	2240669	116017	87.46
2	13.903	VB	49.000	91930	4593	3.59
3	16.760	BB	39.000	21650	1096	0.85
4	18.917	BB	67.000	207558	8158	8.10

(S)-2-(tert-butyl)-1-((4-methoxyphenyl)(phenyl)methyl)-2,3-dihydro-1H-benzo[c][1,2]azaborole (3j)

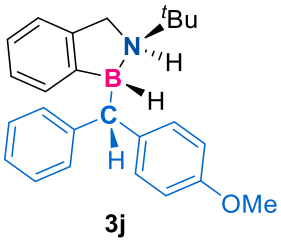

3j

Following the above procedure **E**, isolated yield: 90%, (33.4 mg), white solid (mp: 52.5 – 55.7 ºC), R_f = 0.4 (silica gel, hexane/DCM = 3:1, v/v), column chromatography (silica gel, hexane/DCM = 3:1, v/v).

^{1}H NMR (500 MHz, $CDCl_3$) δ 7.37 – 7.34 (m, 2H), 7.27 – 7.22 (m, 2H), 7.16 – 7.12 (m, 2H), 7.10 – 7.03 (m, 2H), 7.01 – 6.97(m, 1H), 6.85 (dd, *J* = 9.4, 6.2 Hz, 1H), 6.80 – 6.75 (m, 2H), 6.64 (d, *J* = 7.2 Hz, 1H), 4.43 – 4.32 (m, 2H), 4.08 (s, 1H), 3.76 (s, 3H), 3.36 (d, *J* = 7.0 Hz, 1H), 1.13 (s, 9H).

^{13}C NMR (126 MHz, $CDCl_3$) δ 157.0, 148.9, 139.5, 137.8, 131.1, 129.8, 128.7, 128.1, 127.6, 126.2, 125.1, 124.6, 123.9, 120.1, 113.5, 57.6, 55.1, 52.1, 26.5.

^{11}B NMR (160 MHz, $CDCl_3$) δ -1.7.

HRMS (ESI) m/z: $[M+H]^+$ Calcd. for $C_{25}H_{31}BNO$ 372.2493; Found: 372.2492.

HPLC analysis: DAICEL CHIRALCEL IG, hexane/isopropanol = 98/2, 0.5 mL/min, λ = 267 nm, t_1 = 14.297 min, t_2 = 15.135 min, t_3 = 17.629 min, t_4 = 18.669 min, 95% ee, dr = 5:1.

$[\alpha]^{25}_{D}$: +25.00 (*c* 0.3, $CHCl_3$).

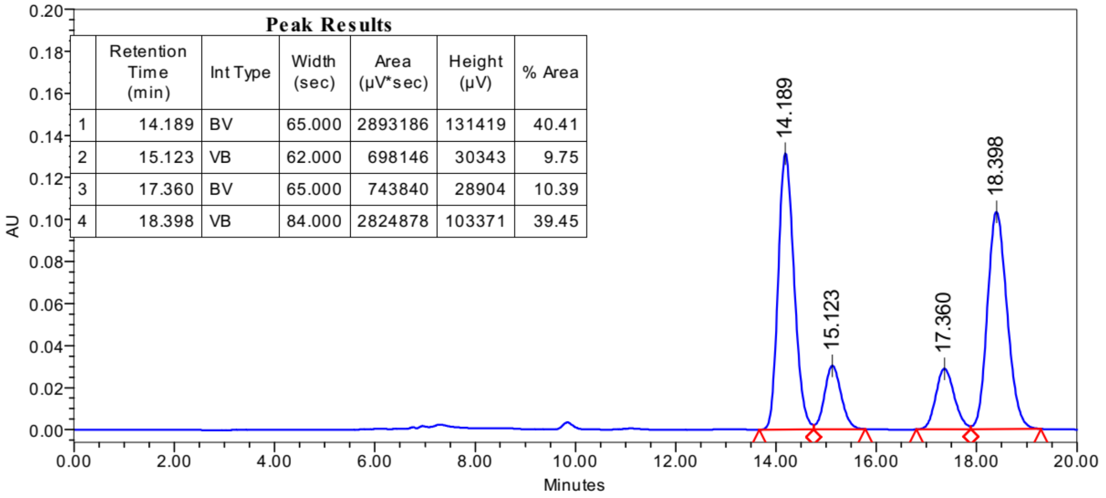

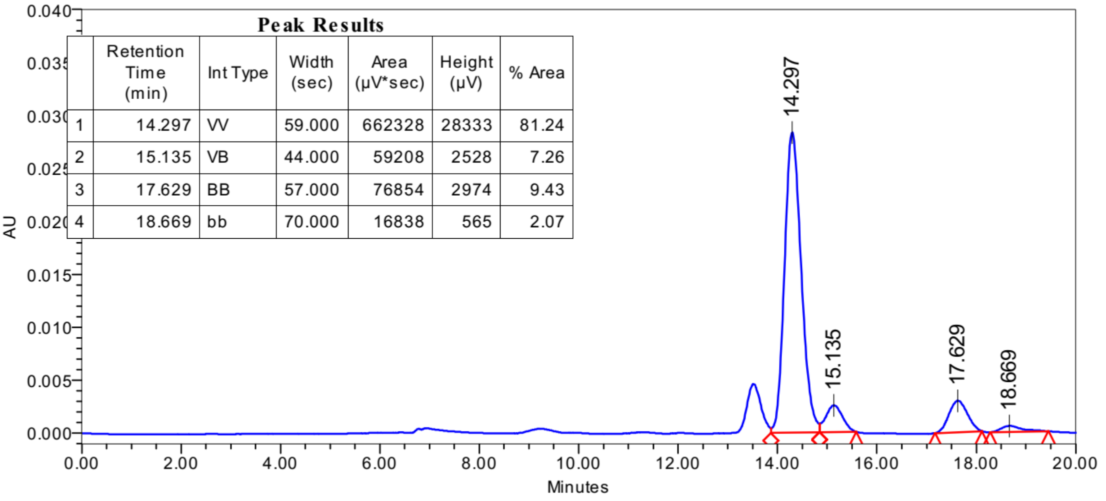

(R)-2-(tert-butyl)-1-((3-chloro-4-fluorophenyl)(4-methoxyphenyl)methyl)-2,3-dihydro-1*H*-benzo[c][1,2]azaborole (3k)

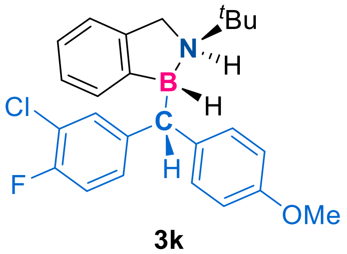

3k

Following the above procedure **E**, isolated yield: 51%, (21.6 mg), white solid (mp: 127.5 – 129.4 ºC), R_f = 0.3 (silica gel, hexane/EtOAc = 3:1, v/v), column chromatography (silica gel, hexane/EtOAc = 3:1, v/v).

^{1}H NMR (500 MHz, $CDCl_3$) δ 7.23 (t, *J* = 8.1 Hz, 1H), 7.10 – 7.00 (m, 3H), 6.99 – 6.95 (m, 2H), 6.84 – 6.73 (m, 4H), 6.50 – 6.46 (m, 1H), 4.09 (dd, *J* = 15.4, 4.7 Hz, 1H), 3.86 (dd, *J* = 15.4, 8.3 Hz, 1H), 3.77 (s, 3H), 3.58 (d, *J* = 5.9 Hz, 1H), 3.47 (s, 1H), 1.25 (s, 9H).

^{13}C NMR (126 MHz, $CDCl_3$) δ 157.8 (d, *J* = 247.1 Hz), 157.4, 149.85 (d, *J* = 6.3 Hz), 139.4, 136.3, 130.9, 129.7, 129.4, 128.7, 126.4, 125.4, 124.2 (d, *J* = 3.4 Hz), 120.3, 115.8 (d, *J* = 20.7 Hz), 115.4 (d, *J* = 17.8 Hz), 114.0, 57.9, 55.1, 52.6, 26.6.

^{11}B NMR (160 MHz, $CDCl_3$) δ -2.1.

^{19}F NMR (471 MHz, $CDCl_3$) δ -117.1.

HRMS (ESI) m/z: $[M+H]^+$ Calcd. for $C_{25}H_{29}BClNO$ 424.201; Found: 424.2021.

HPLC analysis: DAICEL CHIRALCEL OZ-3, hexane/isopropanol = 99/1, 0.5 mL/min, λ = 267 nm, t_1 = 12.857 min, t_2 = 14.168 min, t_3 = 15.211 min, t_4 = 18.875 min, 95% ee, dr = 11.7:1.

$[\alpha]^{25}_D$: -16.33 (*c* 0.3, $CHCl_3$).

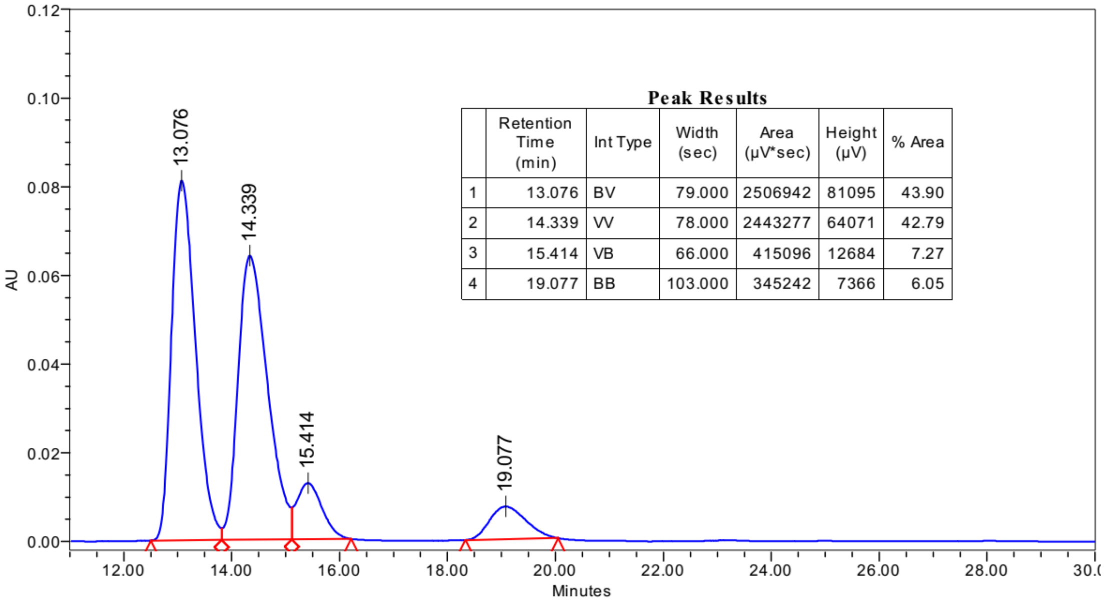

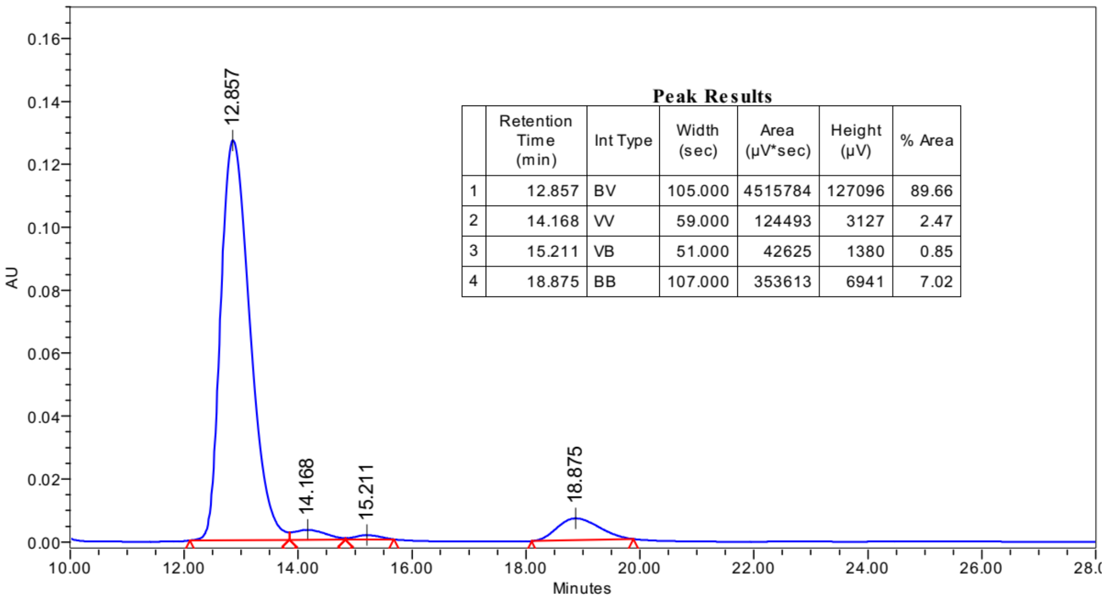

(R)-2-(tert-butyl)-1-((3,4-dichlorophenyl)(4-methoxyphenyl)methyl)-2,3-dihydro-1H-benzo[c][1,2]azaborole (**3l**)

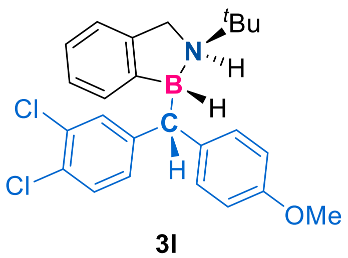

3l

Following the above procedure **E**, isolated yield: 82%, (36.0 mg), white solid (mp: 140.1 – 142.8 ºC), R_f = 0.3 (silica gel, hexane/EtOAc = 3:1, v/v), column chromatography (silica gel, hexane/EtOAc = 3:1, v/v).

^{1}H NMR (500 MHz, Acetone-d_6) δ 7.31 (s, 2H), 7.17 (d, J = 1.8 Hz, 1H), 7.08 – 6.97 (m, 4H), 6.90 (t, J = 7.0 Hz, 1H), 6.80 – 6.75 (m, 2H), 6.55 (d, J = 7.3 Hz, 1H), 4.37 – 4.26 (m, 3H), 3.76 (s, 3H), 3.55 (d, J = 5.8 Hz, 1H), 1.26 (s, 9H).

^{13}C NMR (126 MHz, Acetone-d_6) δ 158.0, 154.7, 141.3, 137.4, 134.2, 131.3, 129.9, 127.3, 126.2, 125.6, 123.7, 120.9, 114.0, 58.5, 54.9, 53.0, 26.2.

^{11}B NMR (160 MHz, Acetone-d_6) δ -1.7.

HRMS (ESI) m/z: [M+Cl]$^-$ Calcd. for $C_{25}H_{28}BCl_3NO$ 474.1335; Found: 474.1348.

HPLC analysis: DAICEL CHIRALCEL AD-H, hexane/isopropanol = 98/2, 0.5 mL/min, λ = 267 nm, t_1 = 9.284 min, t_2 = 10.135 min, t_3 = 10.690 min, t_4 = 13.296 min, 89% ee.

[α]$^{25}_{D}$: +10.56 (c 0.36, $CHCl_3$).

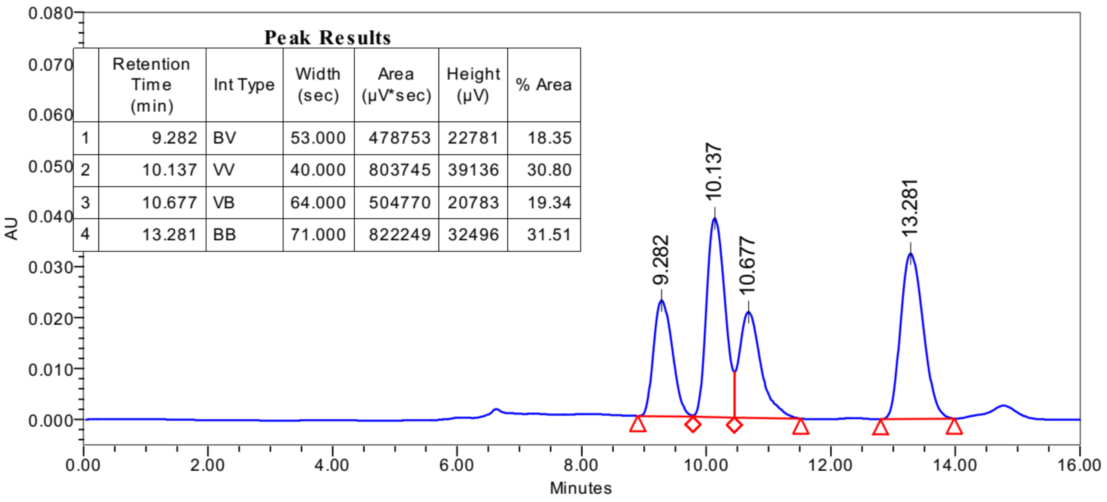

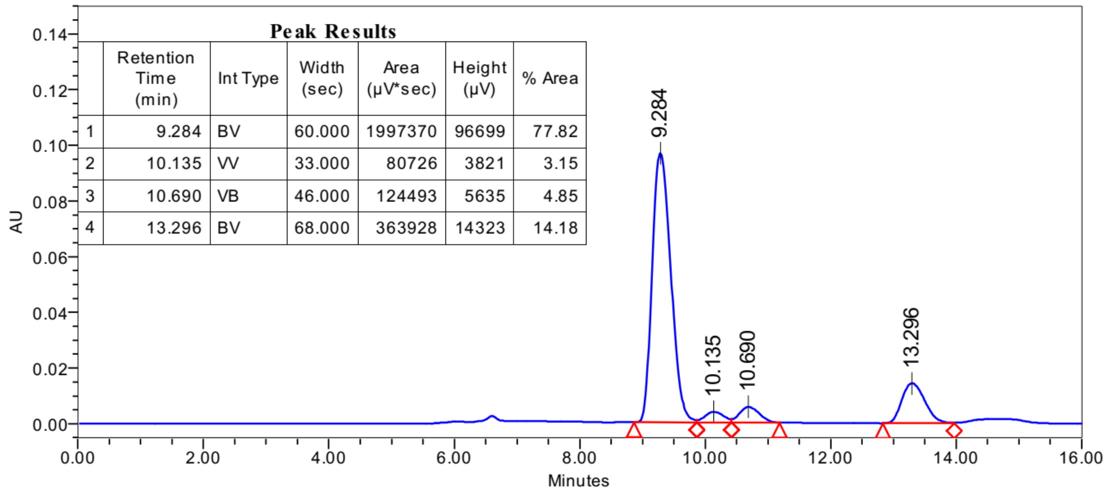

(R)-2-(tert-butyl)-1-((3,5-difluorophenyl)(4-methoxyphenyl)methyl)-2,3-dihydro-1*H*-benzo[c][1,2]azaborole (3m)

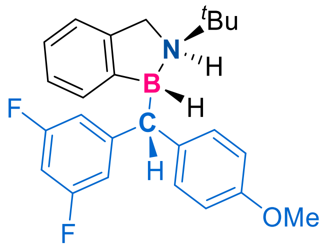

3m

Following the above procedure **E**, isolated yield: 64%, (26.1 mg), white solid (mp: 134.6 – 137.5 °C), R_f = 0.3 (silica gel, hexane/EtOAc = 3:1, v/v), column chromatography (silica gel, hexane/EtOAc = 3:1, v/v).

^{1}H NMR (500 MHz, $CDCl_3$) δ 7.10 – 7.05 (m, 1H), 7.00 – 6.95 (m, 2H), 6.87 – 6.82 (m, 2H), 6.82 – 6.78 (m, 2H), 6.78 – 6.74 (m, 2H), 6.55 – 6.50 (m, 1H), 6.49 (d, *J* = 7.8 Hz, 1H), 4.10 (dd, *J* = 15.4, 4.5 Hz, 1H), 3.88 (dd, *J* = 15.4, 8.2 Hz, 1H), 3.77 (s, 3H), 3.59 (d, *J* = 5.9 Hz, 1H), 3.48 (s, 1H), 1.24 (s, 9H).

^{13}C NMR (126 MHz, $CDCl_3$) δ 163.8 (d, *J* = 13.4 Hz), 161.9 (d, *J* = 13.5 Hz), 157.4, 153.0 (t, *J* = 8.5

Hz), 139.4, 136.1, 131.0, 129.7, 126.5, 125.4, 120.3, 114.0, 110.2 (dd, J = 19.0, 5.4 Hz), 98.9 (t, J = 25.8 Hz), 57.9, 55.1, 52.6, 26.6.

^{11}B NMR (160 MHz, $CDCl_3$) δ -3.1.

^{19}F NMR (471 MHz, $CDCl_3$) δ -111.7.

HRMS (ESI) m/z: $[M+H]^+$ Calcd. for $C_{25}H_{29}BFNO$ 408.2305; Found: 408.2311.

HPLC analysis: DAICEL CHIRALCEL AD-H, hexane/isopropanol = 98/2, 0.5 mL/min, λ = 267 nm, t_1 = 9.742 min, t_2 = 10.911 min, t_3 = 11.628 min, t_4 = 13.278 min, 88% ee, dr = 5.3:1.

[α]$^{25}_{D}$: +19.6 (*c* 0.31, $CHCl_3$).

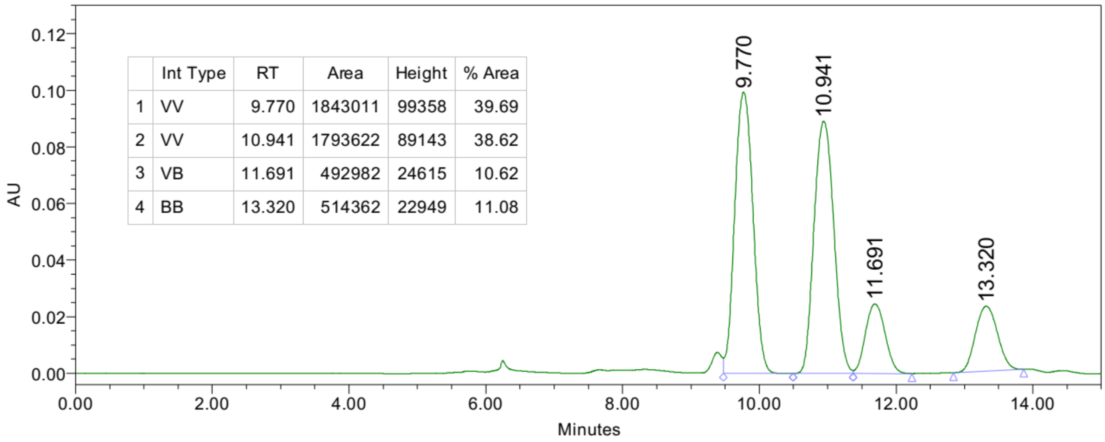

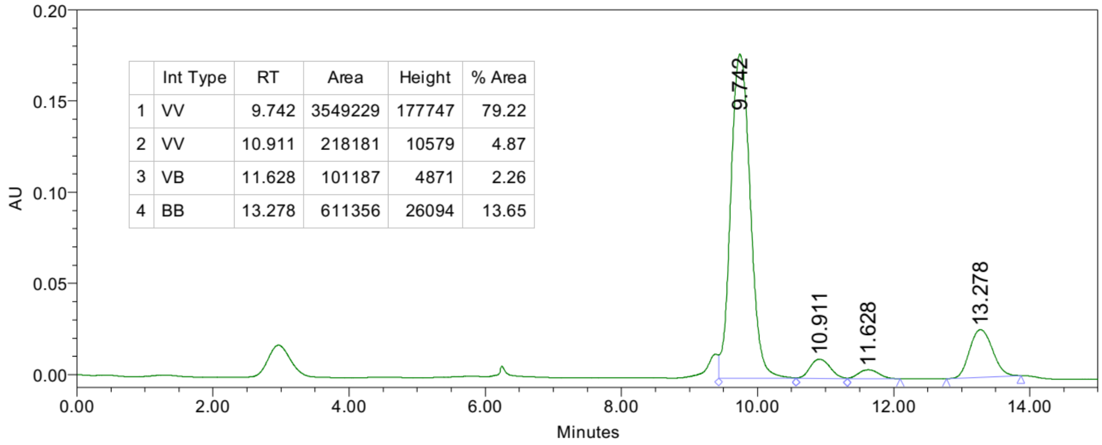

(R)-2-(tert-butyl)-1-((4-fluorophenyl)(4-methoxyphenyl)methyl)-2,3-dihydro-1H-benzo[c][1,2]azaborole (3n)

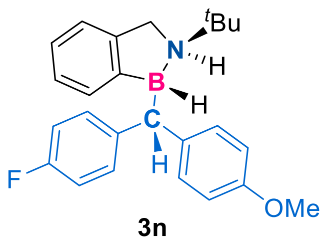

3n

Following the above procedure **E**, isolated yield: 88%, (34.3 mg), white solid (mp: 117.4 – 119.8 °C), R_f

= 0.4 (silica gel, hexane/DCM = 3:1, v/v), column chromatography (silica gel, hexane/DCM = 3:1, v/v).

^{1}H NMR (500 MHz, $CDCl_3$) δ 7.25 – 7.21 (m, 2H), 7.08 – 7.05 (m, 1H), 7.00 – 6.95 (m, 2H), 6.95 – 6.91 (m, 4H), 6.76 (d, *J* = 8.7 Hz, 2H), 6.52 (d, *J* = 7.3 Hz, 1H), 4.11 (dd, *J* = 15.4, 4.1 Hz, 1H), 3.96 (dd, *J* = 15.4, 8.1 Hz, 1H), 3.77 (s, 3H), 3.49 (d, *J* = 6.3 Hz, 2H), 1.20 (s, 9H).

^{13}C NMR (126 MHz, $CDCl_3$) δ 160.2 (d, *J* = 241.5 Hz), 157.1, 144.1 (d, *J* = 3.4 Hz), 139.5, 137.6, 131.0, 129.8, 128.8 (d, *J* = 7.5 Hz), 126.3, 125.2, 120.2, 114.5 (d, *J* = 20.7 Hz), 113.7, 57.8, 55.1, 52.4, 26.6.

^{11}B NMR (160 MHz, $CDCl_3$) δ -1.9.

^{19}F NMR (471 MHz, $CDCl_3$) δ -120.6.

HRMS (ESI) m/z: $[M+H]^+$ Calcd. for $C_{25}H_{30}BFNO$ 390.2399; Found: 390.2400.

HPLC analysis: DAICEL CHIRALCEL IG, hexane/isopropanol = 98/2, 1mL/min, λ = 267 nm, t_1 = 14.214 min, t_2 = 15.290 min, t_3 = 16.936 min, t_4 = 18.374 min, 95% ee, dr =9.2:1.

[α]$^{25}_{D}$: +42.571 (*c* 0.35, $CHCl_3$).

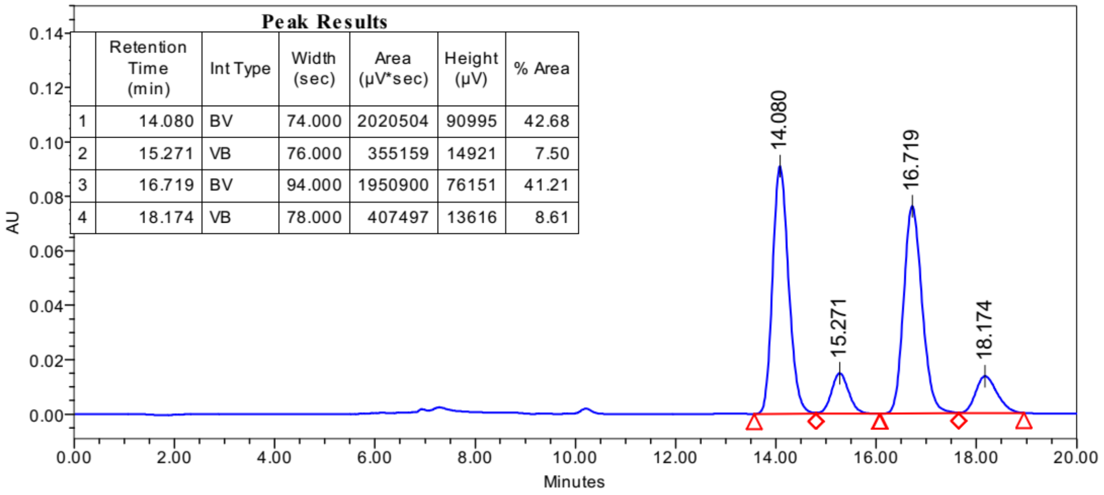

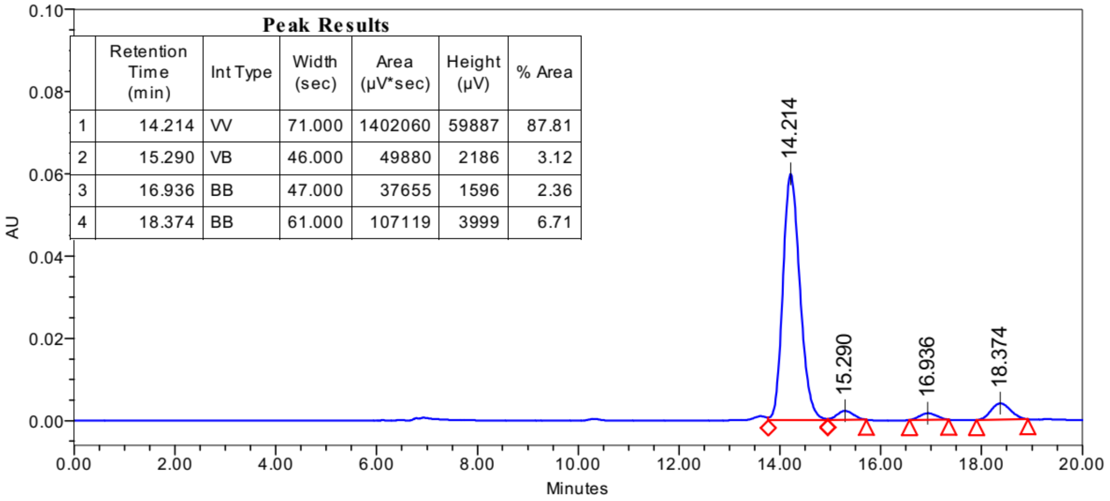

(R)-1-((4-bromophenyl)(4-methoxyphenyl)methyl)-2-(tert-butyl)-2,3-dihydro-1*H*-benzo[c][1,2]azaborole (3o)

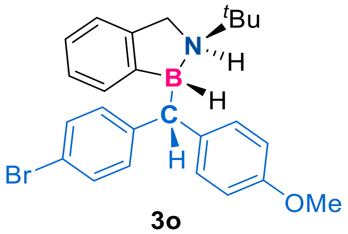

3o

Following the above procedure **E**, isolated yield: 88%, (39.5 mg), white solid (mp: 105.1 – 107.4 ºC), R_f = 0.3 (silica gel, hexane/DCM = 3:1, v/v), column chromatography (silica gel, hexane/DCM = 3:1, v/v).

^{1}H NMR (500 MHz, $CDCl_3$) δ 7.38 – 7.35 (m, 2H), 7.17 (d, *J* = 8.4 Hz, 2H), 7.10 – 7.06 (m, 1H), 7.01 – 6.95 (m, 2H), 6.93 – 6.89 (m, 2H), 6.79 – 6.75 (m, 2H), 6.53 (d, *J* = 7.3 Hz, 1H), 4.11 (dd, *J* = 15.4, 4.3 Hz, 1H), 3.93 (dd, *J* = 15.4, 8.2 Hz, 1H), 3.78 (s, 3H), 3.52 (d, *J* = 6.2 Hz, 1H), 3.49 (s, 1H), 1.22 (s, 9H).

^{13}C NMR (126 MHz, $CDCl_3$) δ 157.2, 147.5, 139.4, 137.0, 130.9, 130.7, 129.8, 129.6, 126.3, 125.3, 120.2, 117.2, 113.8, 57.8, 55.1, 52.4, 47.7, 26.6.

^{11}B NMR (160 MHz, $CDCl_3$) δ -1.6.

HRMS (ESI) m/z: $[M-H]^-$ Calcd. for $C_{25}H_{28}BBrNO$ 448.1453; Found: 448.1443.

HPLC analysis: DAICEL CHIRALCEL AD-H, hexane/isopropanol = 98/2, 0.5 mL/min, λ = 267 nm, t_1 = 13.128 min, t_2 = 17.846 min, t_3 = 19.295 min, t_4 = 21.524 min, 98% ee, dr = 21.5:1.

$[\alpha]^{25}_D$: -18.71 (*c* 0.31, $CHCl_3$).

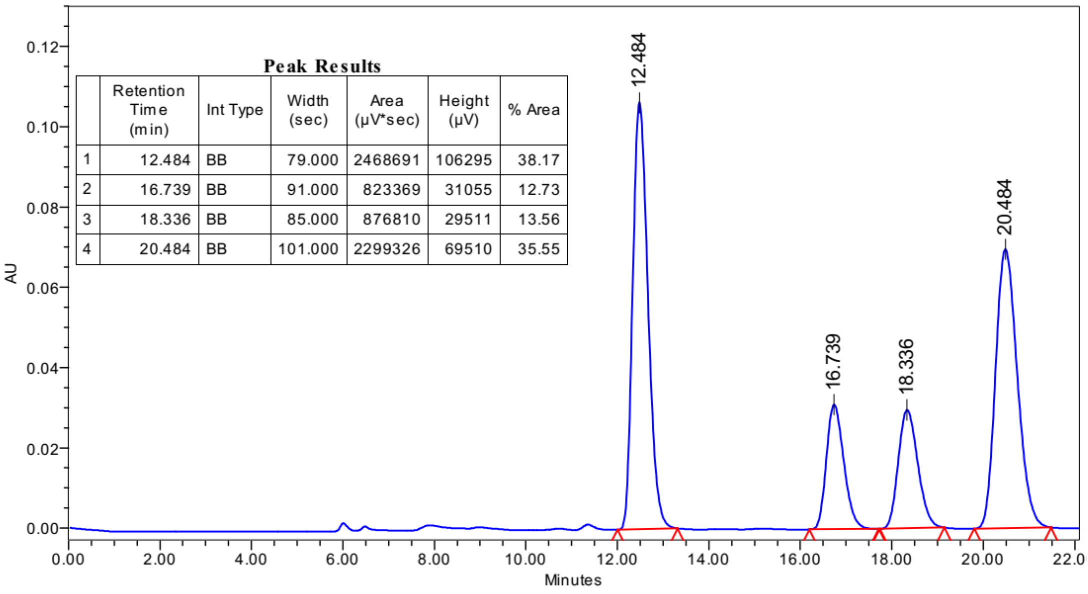

Peak Results

	Retention Time (min)	Int Type	Width (sec)	Area (μV*sec)	Height (μV)	% Area
1	12.484	BB	79.000	2468691	106295	38.17
2	16.739	BB	91.000	823369	31055	12.73
3	18.336	BB	85.000	876810	29511	13.56
4	20.484	BB	101.000	2299326	69510	35.55

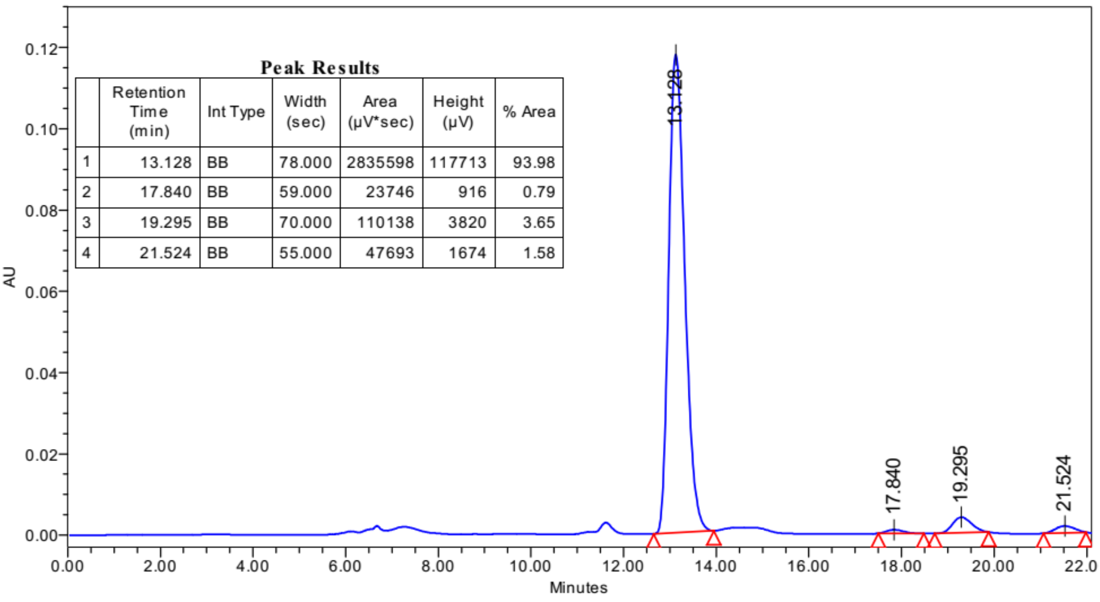

	Retention Time (min)	Int Type	Width (sec)	Area (µV*sec)	Height (µV)	% Area
1	13.128	BB	78.000	2835598	117713	93.98
2	17.840	BB	59.000	23746	916	0.79
3	19.295	BB	70.000	110138	3820	3.65
4	21.524	BB	55.000	47693	1674	1.58

(S)-4-((2-(tert-butyl)-2,3-dihydro-1*H*-benzo[c][1,2]azaborol-1-yl)(4-methoxyphenyl)methyl)benzonitrile (**3p**)

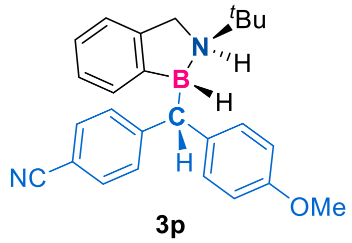

3p

Following the above procedure **E**, isolated yield: 76%, (24.7 mg), white solid (mp: 66.8 – 69.3 ºC), R_f = 0.3 (silica gel, hexane/DCM = 3:1, v/v), column chromatography (silica gel, hexane/DCM = 3:1, v/v).

^{1}H NMR (500 MHz, $CDCl_3$) δ 7.53 – 7.49 (m, 2H), 7.34 (d, *J* = 8.2 Hz, 2H), 7.09 – 7.06 (m, 1H), 7.00 – 6.92 (m, 2H), 6.84 – 6.74 (m, 4H), 6.28 (d, *J* = 7.3 Hz, 2H), 4.09 (dd, *J* = 15.7, 4.6 Hz, 1H), 3.86 (dd, *J* = 15.4, 8.3 Hz, 1H), 3.77 (s, 3H), 3.74 (d, *J* = 5.7 Hz, 1H), 3.49 (s, 1H), 1.26 (s, 9H).

^{13}C NMR (126 MHz, $CDCl_3$) δ 157.5, 154.5, 139.4, 135.6, 131.6, 131.1, 129.5, 128.3, 126.4, 125.5, 120.4, 120.0, 114.1, 106.9, 58.0, 55.1, 52.8, 26.6.

^{11}B NMR (160 MHz, $CDCl_3$) δ -1.8.

HRMS (ESI) m/z: $[M+H]^+$ Calcd. for $C_{26}H_{30}BNO$ 397.2446; Found: 397.2444.

HPLC analysis: DAICEL CHIRALCEL AD-H, hexane/isopropanol = 90/10, 0.5 mL/min, λ = 267 nm, t_1 = 12.479 min, t_2 = 16.990 min, t_3 = 17.935 min, t_4 = 18.823 min, 93% ee, dr > 30:1.

$[\alpha]^{25}_D$: -39.00 (*c* 0.3, $CHCl_3$).

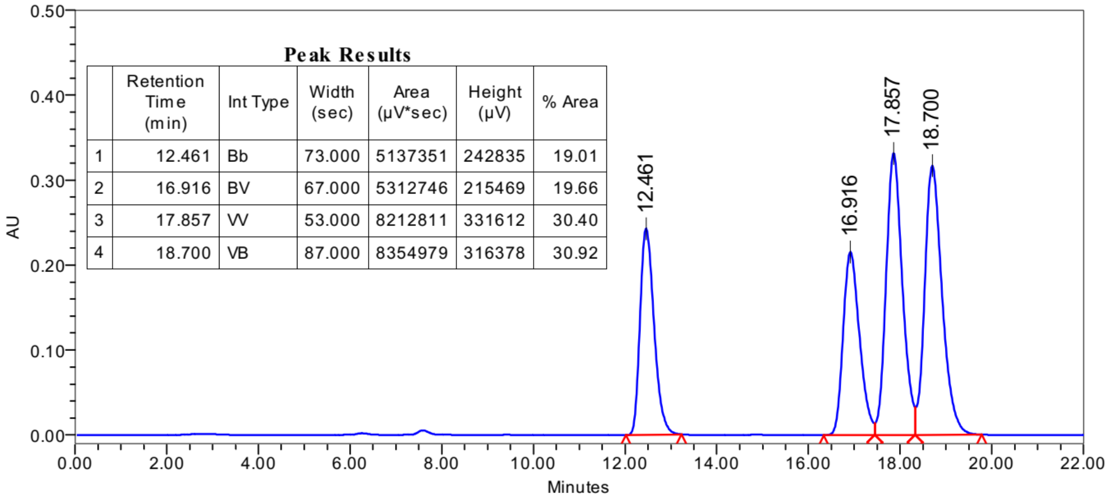

Peak Results

	Retention Time (min)	Int Type	Width (sec)	Area (µV*sec)	Height (µV)	% Area
1	12.461	Bb	73.000	5137351	242835	19.01
2	16.916	BV	67.000	5312746	215469	19.66
3	17.857	VV	53.000	8212811	331612	30.40
4	18.700	VB	87.000	8354979	316378	30.92

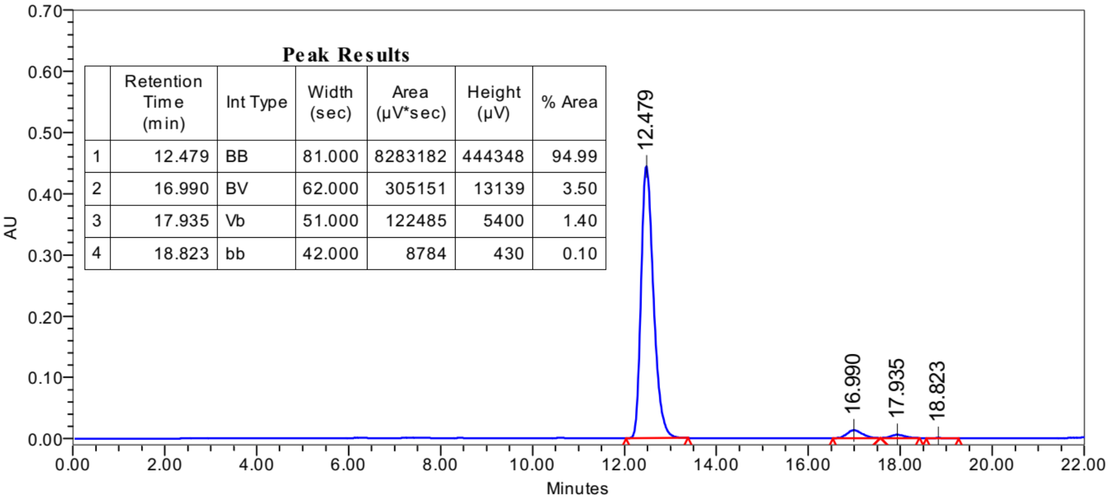

Peak Results

	Retention Time (min)	Int Type	Width (sec)	Area (µV*sec)	Height (µV)	% Area
1	12.479	BB	81.000	8283182	444348	94.99
2	16.990	BV	62.000	305151	13139	3.50
3	17.935	Vb	51.000	122485	5400	1.40
4	18.823	bb	42.000	8784	430	0.10

(R)-2-(tert-butyl)-1-((4-methoxyphenyl)(4-(methylsulfonyl)phenyl)methyl)-2,3-dihydro-1H-benzo[c][1,2]azaborole (**3q**)

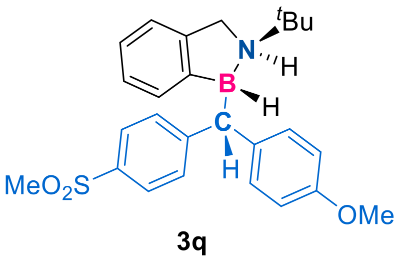

3q

Following the above procedure **E**, isolated yield: 68%, (30.3 mg), white solid (mp: 82.7 – 84.6 °C), R_f = 0.3 (silica gel, hexane/DCM = 3:1, v/v), column chromatography (silica gel, hexane/DCM = 3:1, v/v).

^{1}H NMR (400 MHz, $CDCl_3$) δ 7.79 (d, *J* = 8.3 Hz, 2H), 7.43 (d, *J* = 8.3 Hz, 2H), 7.07 (t, *J* = 7.3 Hz, 1H), 7.00 – 6.90 (m, 2H), 6.81 (dd, *J* = 34.1, 8.6 Hz, 4H), 6.33 (d, *J* = 7.3 Hz, 1H), 4.10 (dd, *J* = 15.4, 4.5 Hz, 1H), 3.88 (dd, *J* = 15.4, 8.3 Hz, 1H), 3.77 (s, 3H), 3.74 (d, *J* = 5.6 Hz, 1H), 3.48 (s, 1H), 3.03 (s, 3H), 1.25 (s, 9H).

^{13}C NMR (101 MHz, $CDCl_3$) δ 157.5, 155.6, 139.4, 135.8, 135.5, 131.1, 129.6, 128.4, 126.9, 126.5, 125.6, 120.4, 114.1, 58.1, 55.2, 52.8, 44.8, 26.7.

^{11}B NMR (160 MHz, $CDCl_3$) δ -1.7.

HRMS (ESI) m/z: $[M+H]^+$ Calcd. for $C_{26}H_{33}BNO_3S$ 450.2269; Found: 450.2274.

HPLC analysis: DAICEL CHIRALCEL IB N-3, hexane/isopropanol = 80/20, 1mL/min, λ = 267 nm, t_1 = 28.310 min, t_2 = 31.376 min, t_3 = 32.973 min, t_4 = 36.842 min, 90% ee, dr = 5.9:1.

$[\alpha]^{25}_D$: -19.00 (*c* 0.3, $CHCl_3$).

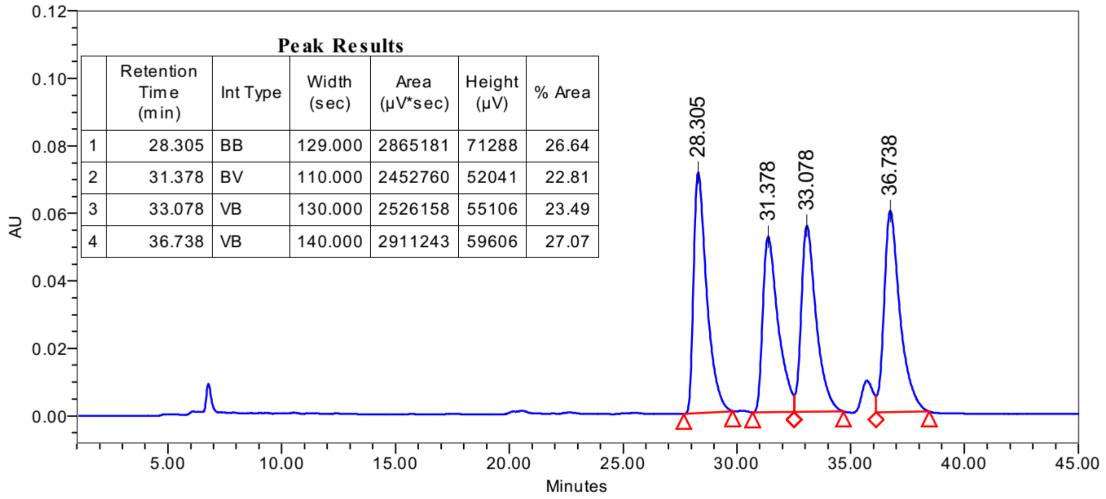

	Retention Time (min)	Int Type	Width (sec)	Area (μV*sec)	Height (μV)	% Area
1	28.305	BB	129.000	2865181	71288	26.64
2	31.378	BV	110.000	2452760	52041	22.81
3	33.078	VB	130.000	2526158	55106	23.49
4	36.738	VB	140.000	2911243	59606	27.07

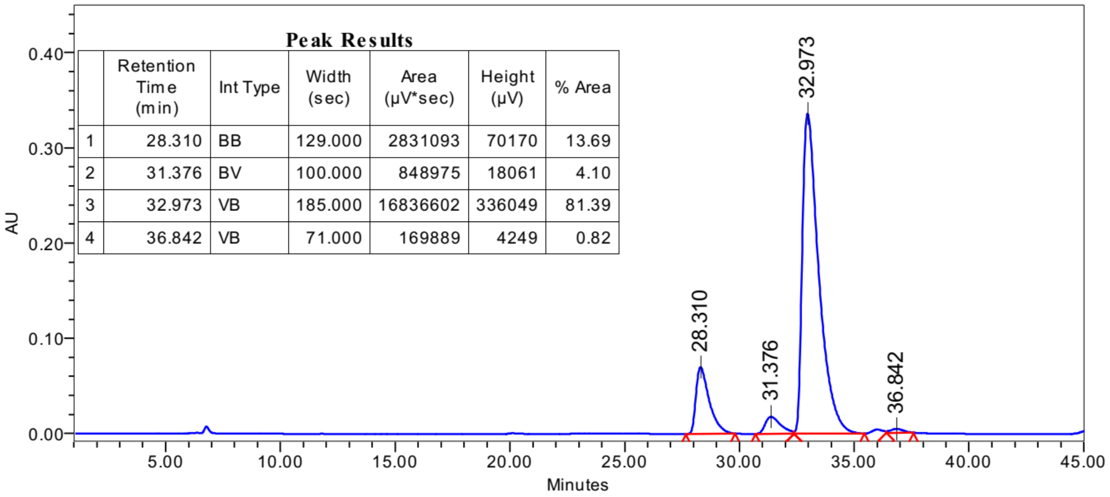

	Retention Time (min)	Int Type	Width (sec)	Area (μV*sec)	Height (μV)	% Area
1	28.310	BB	129.000	2831093	70170	13.69
2	31.376	BV	100.000	848975	18061	4.10
3	32.973	VB	185.000	16836602	336049	81.39
4	36.842	VB	71.000	169889	4249	0.82

ethyl (S)-4-((2-(tert-butyl)-2,3-dihydro-1H-benzo[c][1,2]azaborol-1-yl)(4-methoxyphenyl)methyl)benzoate (3r)

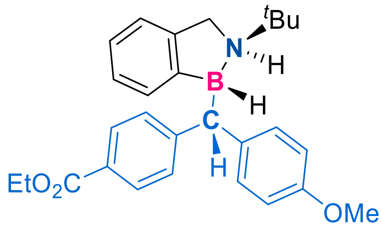

3r

Following the above procedure **E**, isolated yield: 70%, (30.3 mg), white solid (mp: 62.4 – 65.8 °C), R_f = 0.3 (silica gel, hexane/DCM = 3:1, v/v), column chromatography (silica gel, hexane/DCM = 3:1, v/v).

^{1}H NMR (400 MHz, $CDCl_3$) δ 7.92 (d, *J* = 8.1 Hz, 2H), 7.31 (d, *J* = 8.0 Hz, 2H), 7.04 (t, *J* = 7.3 Hz, 1H), 6.99 – 6.89 (m, 4H), 6.76 (d, *J* = 8.5 Hz, 2H), 6.43 (d, *J* = 7.3 Hz, 1H), 4.35 (q, *J* = 7.1 Hz, 2H), 4.09 (dd, *J* = 15.3, 4.0 Hz, 1H), 3.94 (dd, *J* = 15.4, 8.1 Hz, 1H), 3.76 (s, 3H), 3.62 (d, *J* = 5.1 Hz, 1H), 3.50 (s, 1H), 1.38 (t, *J* = 7.1 Hz, 3H), 1.18 (s, 9H).

^{13}C NMR (101 MHz, $CDCl_3$) δ 167.1, 157.3, 154.6, 139.4, 136.6, 131.1, 129.7, 129.3, 127.5, 126.3, 125.9, 125.3, 120.2, 113.8, 60.5, 57.8, 55.1, 52.5, 26.6, 14.3.

^{11}B NMR (128 MHz, $CDCl_3$) δ 0.5.

HRMS (ESI) m/z: $[M+H]^+$ Calcd. for $C_{28}H_{35}BNO_3$ 444.2705; Found: 444.2702.

HPLC analysis: DAICEL CHIRALCEL AD-H, hexane/isopropanol = 90/10, 0.5 mL/min, λ = 267 nm, t_1 = 13.199 min, t_2 = 18.173 min, t_3 = 20.743 min, t_4 = 24.021 min, 96% ee, dr = 13.1:1.

[α]$^{25}_D$: -15.16 (*c* 0.31, $CHCl_3$).

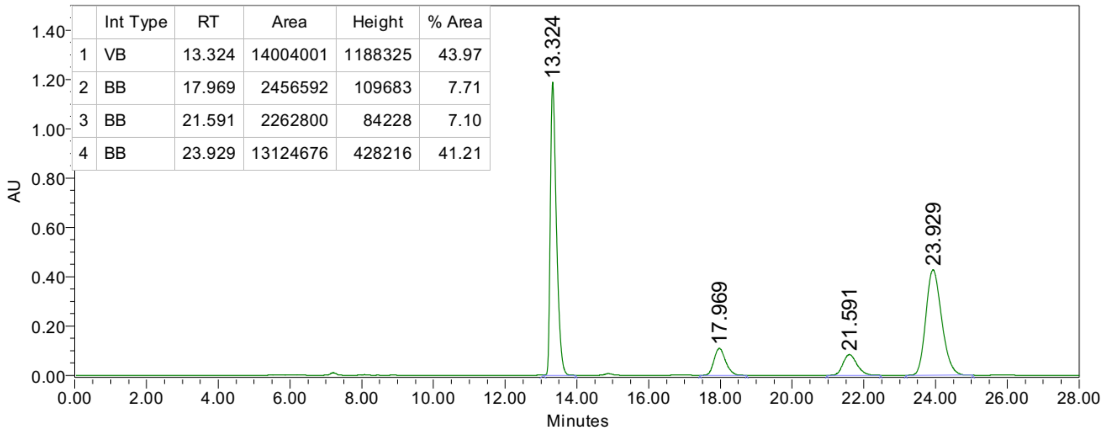

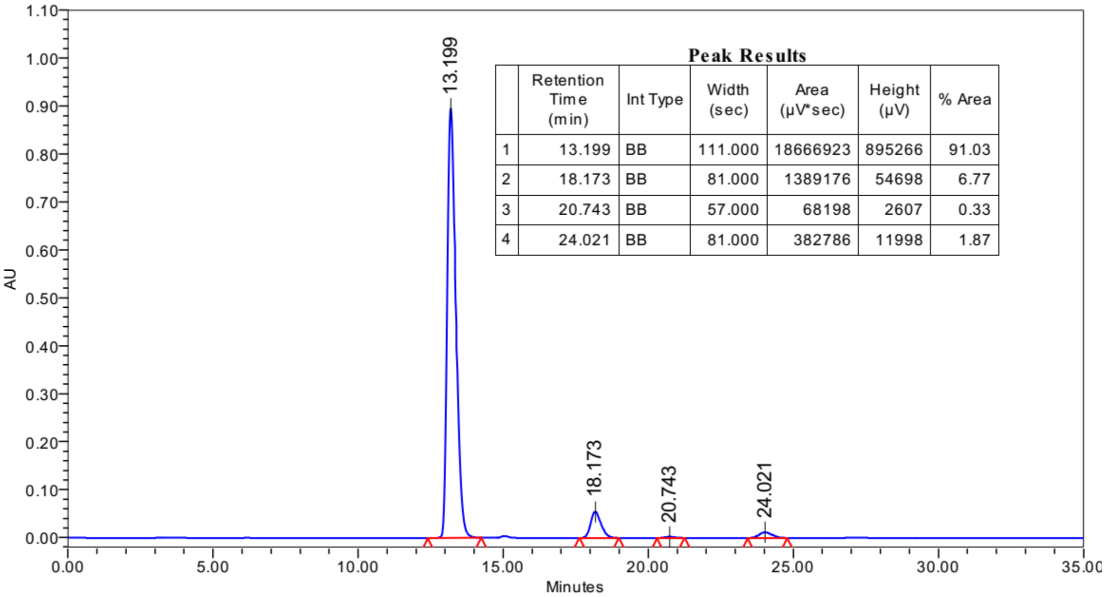

(S)-4-((2-(tert-butyl)-2,3-dihydro-1H-benzo[c][1,2]azaborol-1-yl)(4-methoxyphenyl)methyl)-N,N-dimethylbenzamide (**3s**)

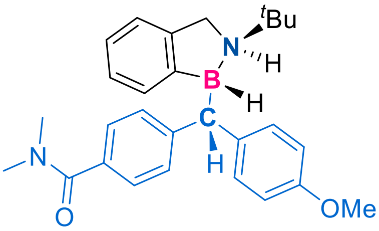

3s

Following the above procedure **E**, isolated yield: 82%, (36.3 mg), white solid (mp: 71.7 – 74.3 °C), R_f = 0.3 (silica gel, hexane/DCM = 3:1, v/v), column chromatography (silica gel, hexane/DCM = 3:1, v/v).

^{1}H NMR (400 MHz, $CDCl_3$) δ 7.24 (dd, *J* = 7.3, 3.7 Hz, 4H), 7.12 (d, *J* = 7.9 Hz, 2H), 7.03 (t, *J* = 7.2 Hz, 1H), 7.00 – 6.89 (m, 2H), 6.83 (d, *J* = 8.4 Hz, 2H), 6.62 (d, *J* = 7.1 Hz, 1H), 4.16 – 4.03 (m, 2H), 3.78 (s, 3H), 3.43 (s, 1H), 3.34 (d, *J* = 6.2 Hz, 1H), 3.07 (s, 3H), 2.97 (s, 3H), 1.12 (s, 9H).

^{13}C NMR (126 MHz, $CDCl_3$) δ 172.2, 157.1, 150.6, 139.4, 137.1, 131.5, 131.0, 129.7, 127.4, 127.1, 126.2, 125.2, 120.2, 113.7, 57.8, 55.1, 52.4, 26.6.

^{11}B NMR (128 MHz, $CDCl_3$) δ -0.9.

HRMS (ESI) m/z: $[M+H]^+$ Calcd. for $C_{28}H_{36}BN_2O_2$ 443.2865; Found: 443.2861.

HPLC analysis: DAICEL CHIRALCEL IC, hexane/isopropanol = 50/50, 0.5 mL/min, λ = 267 nm, t_1= 25.924 min, t_2 = 29.441 min, t_3 = 31.483 min, t_4 = 53.421 min, 98% ee, dr > 30:1.

$[\alpha]^{25}_D$: -11.88 (*c* 0.32, $CHCl_3$).

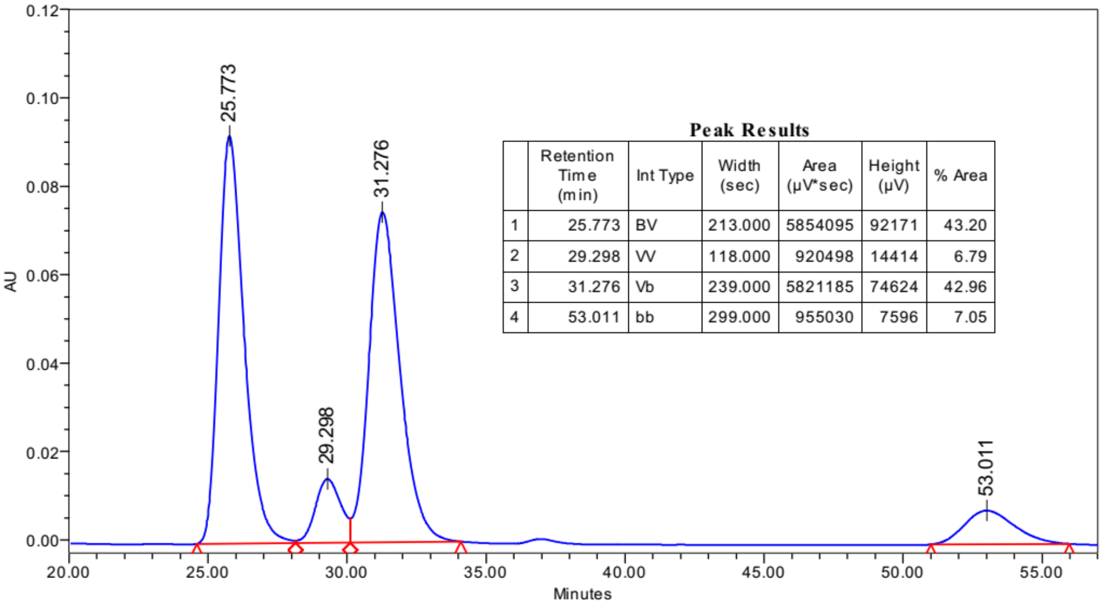

	Retention Time (min)	Int Type	Width (sec)	Area (μV*sec)	Height (μV)	% Area
1	25.773	BV	213.000	5854095	92171	43.20
2	29.298	VV	118.000	920498	14414	6.79
3	31.276	Vb	239.000	5821185	74624	42.96
4	53.011	bb	299.000	955030	7596	7.05

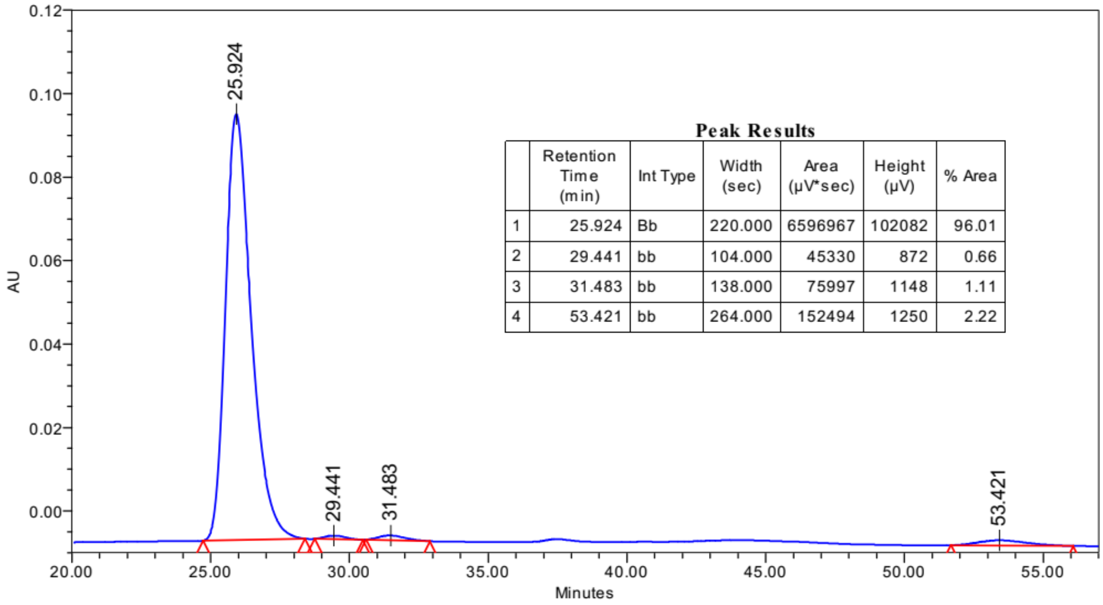

Peak Results

	Retention Time (min)	Int Type	Width (sec)	Area (μV*sec)	Height (μV)	% Area
1	25.924	Bb	220.000	6596967	102082	96.01
2	29.441	bb	104.000	45330	872	0.66
3	31.483	bb	138.000	75997	1148	1.11
4	53.421	bb	264.000	152494	1250	2.22

(R)-2-(tert-butyl)-1-((4-methoxyphenyl)(4-(trifluoromethoxy)phenyl)methyl)-2,3-dihydro-1H-benzo[c][1,2]azaborole (3t)

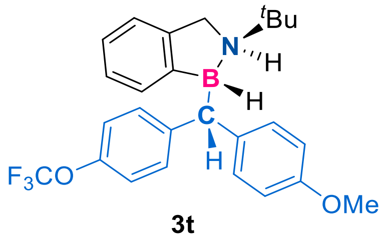

3t

Following the above procedure **E**, isolated yield: 50%, (22.8 mg), white solid (mp: 179.7 – 182.1 ºC), R_f = 0.3 (silica gel, hexane/DCM = 3:1, v/v), column chromatography (silica gel, hexane/DCM = 3:1, v/v).

^{1}H NMR (500 MHz, $CDCl_3$) δ 7.29 (d, *J* = 8.6 Hz, 2H), 7.14 – 7.06 (m, 3H), 7.01 – 6.91 (m, 4H), 6.80 – 6.76 (m, 2H), 6.47 (d, *J* = 7.3 Hz, 1H), 4.12 (dd, *J* = 15.4, 4.2 Hz, 1H), 3.95 (dd, *J* = 15.6, 8.3 Hz, 1H), 3.78 (s, 3H), 3.57 (d, *J* = 6.1 Hz, 1H), 3.49 (s, 1H), 1.21 (s, 9H).

^{13}C NMR (126 MHz, $CDCl_3$) δ 157.2, 147.4, 145.9, 139.5, 137.1, 131.0, 129.7, 128.7, 126.3, 125.3, 120.6 (dd, *J* = 256.3 Hz), 120.5, 120.2, 113.8, 57.8, 55.1, 52.5, 26.6.

^{11}B NMR (160 MHz, $CDCl_3$) δ -1.7.

^{19}F NMR (471 MHz, $CDCl_3$) δ -57.8.

HRMS (ESI) m/z: $[M+H]^+$ Calcd. for $C_{26}H_{30}BF_3NO_2$ 456.2316; Found: 456.2325.

HPLC analysis: DAICEL CHIRALCEL AD-H, hexane/isopropanol = 98/2, 0.5 mL/min, λ = 267 nm, t_1 = 9.377 min, t_2 = 10.482 min, t_3 = 11.369 min, t_4 = 13.852 min, 90% ee, dr = 9.9:1.

$[\alpha]^{25}{}_D$: +12.79 (*c* 0.43, $CHCl_3$).

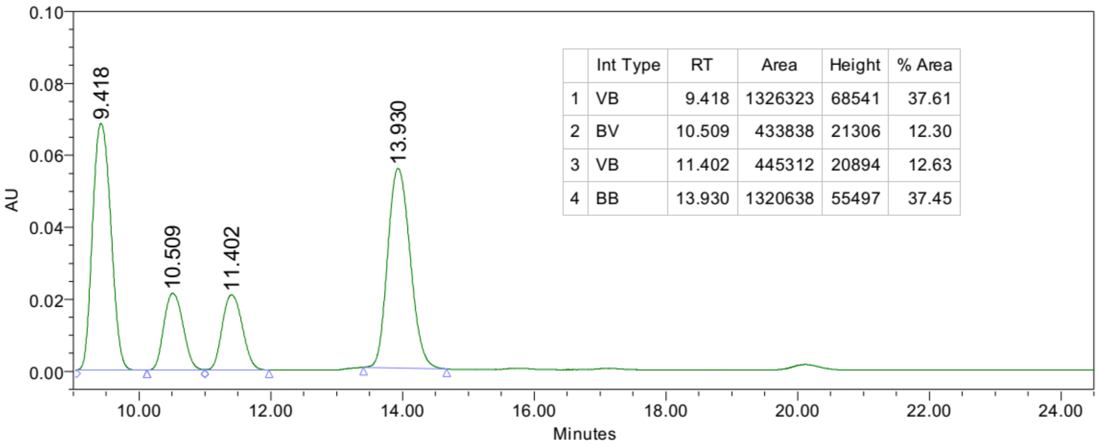

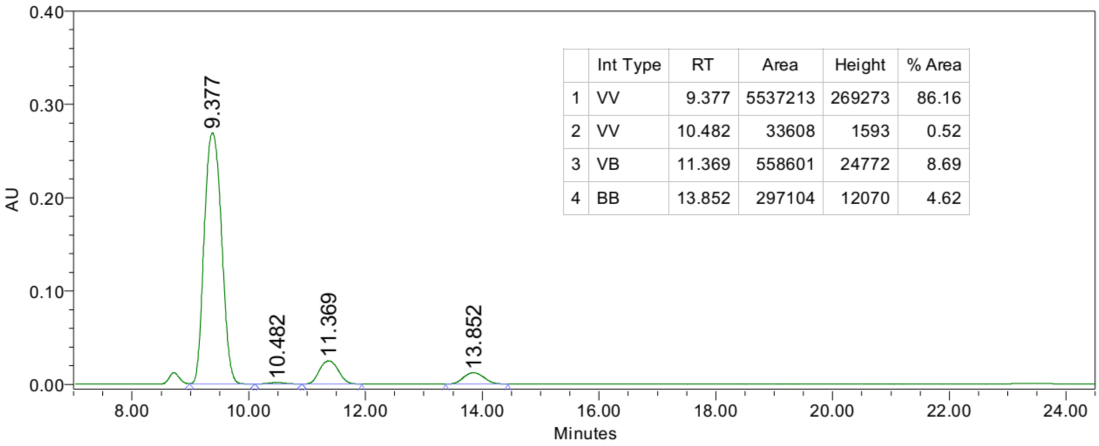

(S)-2-(tert-butyl)-1-((4-methoxyphenyl)(4-(trifluoromethyl)phenyl)methyl)-2,3-dihydro-1H-benzo[c][1,2]azaborole (**3u**)

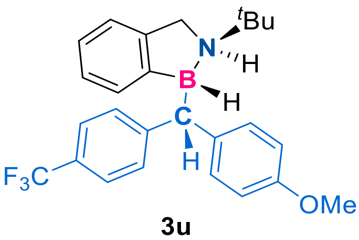

3u

Following the above procedure **E**, isolated yield: 85%, (37.3 mg), white solid (mp: 102.7 – 105.3 °C), R_f = 0.3 (silica gel, hexane/DCM = 3:1, v/v), column chromatography (silica gel, hexane/DCM = 3:1, v/v).

^{1}H NMR (500 MHz, Acetone-d_6) δ 7.55 (q, J = 8.4 Hz, 4H), 7.07 – 7.00 (m, 4H), 6.87 (t, J = 7.1 Hz, 1H), 6.80 – 6.75 (m, 2H), 6.54 (d, J = 7.3 Hz, 1H), 4.36 (dd, J = 15.8, 3.5 Hz, 1H), 4.30 (dd, J = 15.7, 7.4 Hz, 1H), 4.24 (s, 3H), 3.76 (s, 1H), 3.61 (d, J = 6.2 Hz, 1H), 1.22 (s, 9H).

^{13}C NMR (126 MHz, Acetone-d_6) δ157.8, 155.1 (d, J = 1.7 Hz), 141.2, 137.9, 131.4, 130.3 (d, J = 10.8 Hz), 129.9, 129.9 (d, J = 33.5 Hz), 128.9, 126.0, 125.5, 124.8 (dd, J = 8.1, 4.1 Hz), 120.8, 113.8, 58.3, 54.9, 52.8, 26.0.

^{11}B NMR (160 MHz, Acetone-d_6) δ -1.8.

^{19}F NMR (471 MHz, Acetone-d_6) δ -62.1.

HRMS (ESI) m/z: $[M+Cl]^-$ Calcd. for $C_{26}H_{29}BF_3ClNO$ 474.1988; Found: 474.1993.

HPLC analysis: DAICEL CHIRALCEL AD-H, hexane/isopropanol = 98/2, 0.5 mL/min, λ = 267 nm, t_1 = 12.107 min, t_2 = 13.647 min, t_3 = 14.965 min, 87% ee, dr > 30:1. (**Note:** The less abundant diastereomers were not separated, but the diastereoselectivity and the enantioselectivity of the main product could be determined.)

$[\alpha]^{25}_D$: +6.98 (c 0.43, $CHCl_3$).

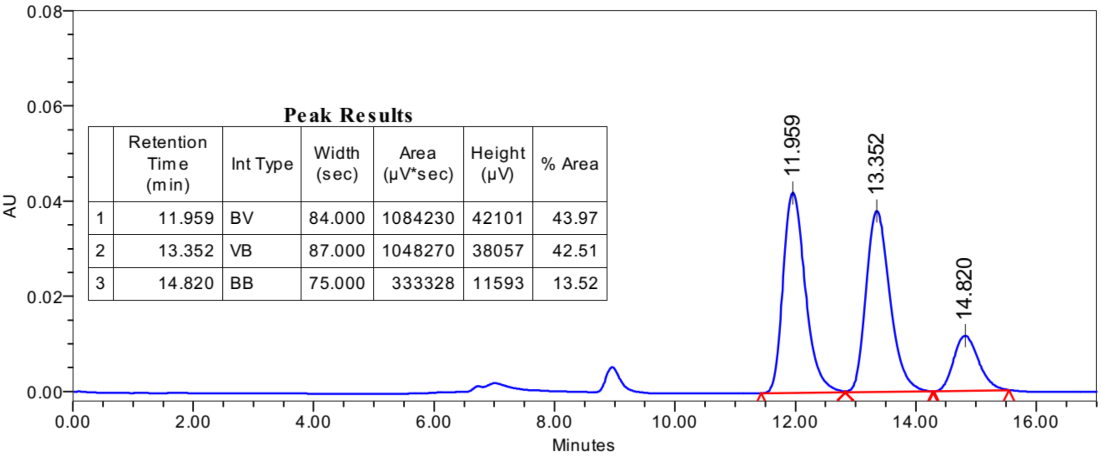

Peak Results

	Retention Time (min)	Int Type	Width (sec)	Area (μV*sec)	Height (μV)	% Area
1	11.959	BV	84.000	1084230	42101	43.97
2	13.352	VB	87.000	1048270	38057	42.51
3	14.820	BB	75.000	333328	11593	13.52

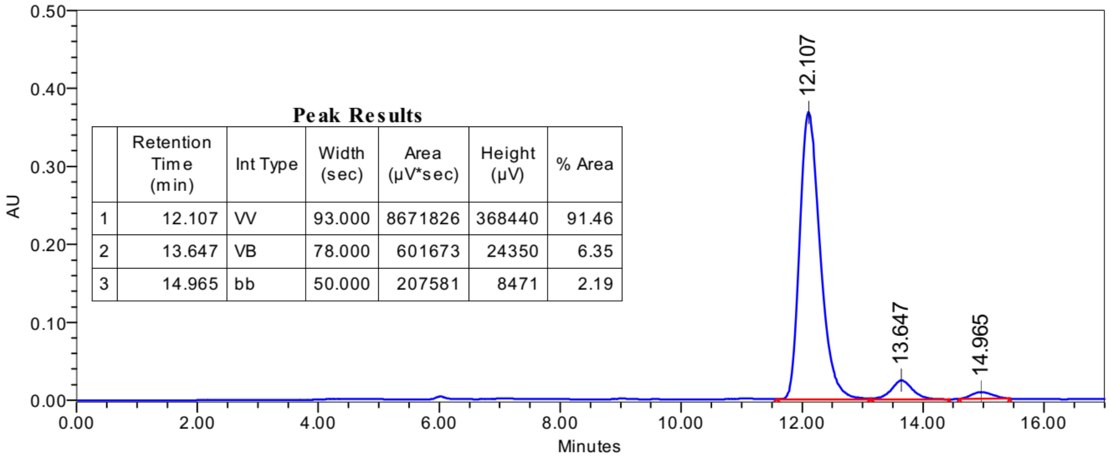

Peak Results

	Retention Time (min)	Int Type	Width (sec)	Area (μV*sec)	Height (μV)	% Area
1	12.107	VV	93.000	8671826	368440	91.46
2	13.647	VB	78.000	601673	24350	6.35
3	14.965	bb	50.000	207581	8471	2.19

(S)-2-(tert-butyl)-1-((3-methoxyphenyl)(4-(trifluoromethyl)phenyl)methyl)-2,3-dihydro-1H-benzo[c][1,2]azaborole (**3v**)

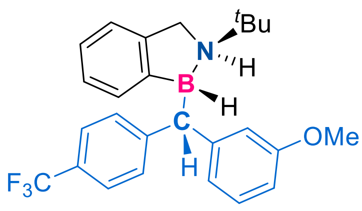

3v

Following the above procedure **E**, isolated yield: 77%, (33.8 mg), white solid (mp: 105.3 – 107.9 ºC), R_f = 0.3 (silica gel, hexane/DCM = 3:1, v/v), column chromatography (silica gel, hexane/DCM = 3:1, v/v).

^{1}H NMR (500 MHz, Acetone-d_6) δ 7.57 (s, 4H), 7.11 (t, *J* = 7.9 Hz, 1H), 7.07 – 7.00 (m, 2H), 6.91 – 6.87 (m, 1H), 6.74 (dd, *J* = 7.6, 0.9 Hz, 1H), 6.69 – 6.67 (m, 1H), 6.61 – 6.58 (m, 1H), 6.55 (d, *J* = 7.3 Hz, 1H), 4.35 – 4.19 (m, 3H), 3.69 (d, *J* = 6.0 Hz, 1H), 3.56 (s, 3H), 1.27 (s, 9H).

^{13}C NMR (126 MHz, Acetone-d_6) δ 159.9, 154.3, 147.6, 141.4, 129.9, 129.3, 129.1, 126.1, 125.8 (dd, *J* = 113.5, 81.6 Hz), 125.5, 124.8 (d, *J* = 3.5 Hz), 122.9, 120.9, 115.5, 111.4, 58.4, 54.5, 52.9, 26.1.

^{11}B NMR (160 MHz, Acetone-d_6) δ -1.4.

^{19}F NMR (471 MHz, Acetone-d_6) δ -62.1.

HRMS (ESI) m/z: $[M+H]^+$ Calcd. for $C_{26}H_{30}BF_3NO$ 440.2367; Found: 440.2371.

HPLC analysis: DAICEL CHIRALCEL OD-H, hexane/isopropanol = 98/2, 0.5 mL/min, λ = 267 nm, t_1 = 11.360 min, t_2 = 13.039 min, t_3 = 13.684 min, t_2 = 14.320 min, 87% ee, dr = 9.1:1.

[α]$^{25}_{D}$: -6.88 (*c* 0.37, $CHCl_3$).

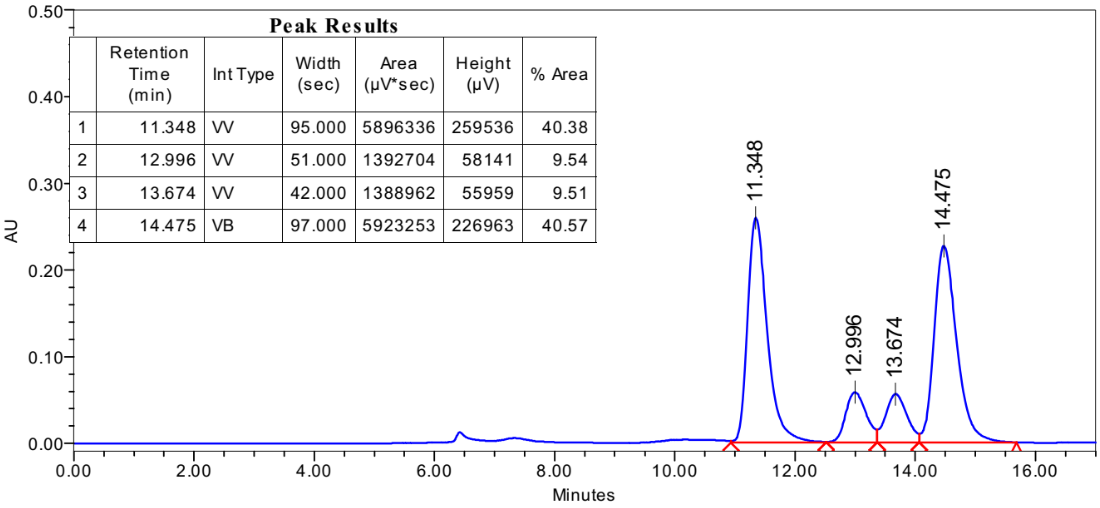

	Retention Time (min)	Int Type	Width (sec)	Area (μV*sec)	Height (μV)	% Area
1	11.348	VV	95.000	5896336	259536	40.38
2	12.996	VV	51.000	1392704	58141	9.54
3	13.674	VV	42.000	1388962	55959	9.51
4	14.475	VB	97.000	5923253	226963	40.57

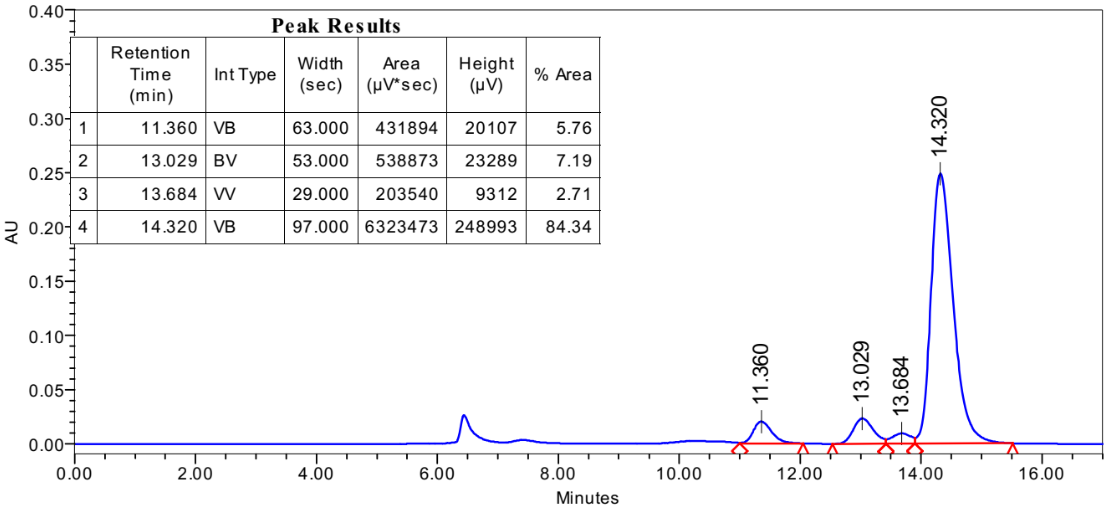

	Retention Time (min)	Int Type	Width (sec)	Area (μV*sec)	Height (μV)	% Area
1	11.360	VB	63.000	431894	20107	5.76
2	13.029	BV	53.000	538873	23289	7.19
3	13.684	VV	29.000	203540	9312	2.71
4	14.320	VB	97.000	6323473	248993	84.34

(S)-1-((4-(benzyloxy)phenyl)(4-(trifluoromethyl)phenyl)methyl)-2-(tert-butyl)-2,3-dihydro-1H-benzo[c][1,2]azaborole (3w)

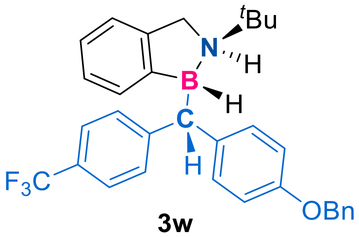

3w

Following the above procedure **E**, isolated yield: 69%, (35.6 mg), white solid (mp: 47.8 – 51.3 °C), R_f = 0.3 (silica gel, hexane/DCM = 3:1, v/v), column chromatography (silica gel, hexane/DCM = 3:1, v/v).

^{1}H NMR (500 MHz, Acetone-d_6) δ 7.49 (q, J = 8.4 Hz, 4H), 7.43 (d, J = 7.6 Hz, 2H), 7.35 (t, J = 7.6 Hz, 2H), 7.28 (t, J = 7.0 Hz, 1H), 6.97 (dd, J = 20.2, 8.1 Hz, 4H), 6.82 – 6.79 (m, 3H), 6.48 (d, J = 6.1 Hz, 1H), 5.01 (s, 2H), 4.29 – 4.17 (m, 3H), 3.55 (d, J = 5.1 Hz, 2H), 1.15 (s, 9H).

^{13}C NMR (126 MHz, Acetone-d_6) δ 157.0, 155.0, 141.3, 138.3 (d, J = 11.4 Hz), 131.5, 129.9, 129.0, 128.8, 128.1, 128.1, 126.1,125.8 (dd, J = 180.8, 148.9 Hz) 125.5, 124.9 (d, J = 3.6 Hz), 120.8, 114.9, 70.0, 58.4, 52.8, 26.1.

^{11}B NMR (160 MHz, Acetone-d_6) δ -1.5.

^{19}F NMR (471 MHz, Acetone-d_6) δ -62.1.

HRMS (ESI) m/z: $[M+H]^+$ Calcd. for $C_{32}H_{34}BF_3NO$ 516.268; Found: 516.2676.

HPLC analysis: DAICEL CHIRALCEL AD-H, hexane/isopropanol = 98/2, 0.5 mL/min, λ = 267 nm, t_1 = 16.872 min, t_2 = 20.869 min, t_3 = 22.863 min, t_4 = 27.151 min, 93% ee, dr = 6.7:1.

[α]^{25}$_D$: +11.75 (c 0.4, $CHCl_3$).

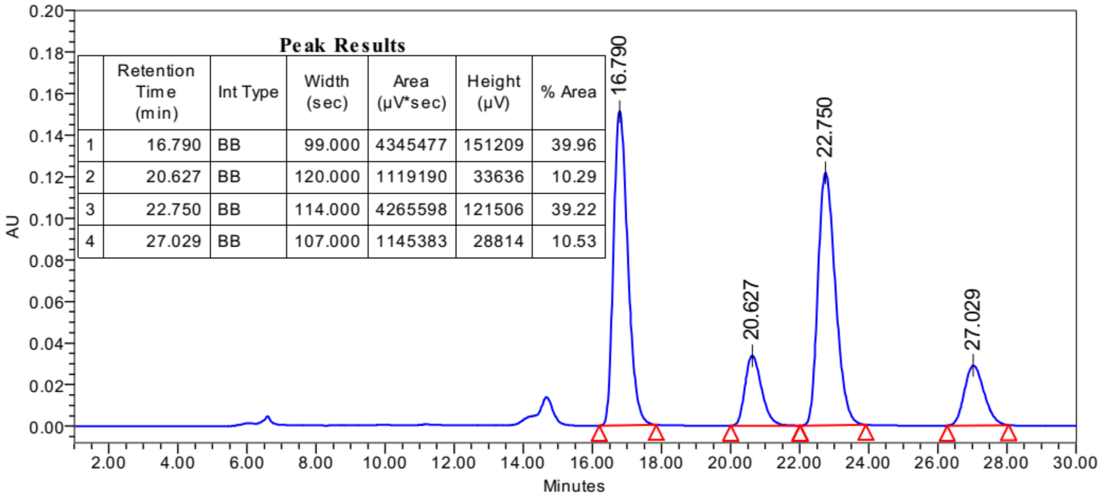

Peak Results

	Retention Time (min)	Int Type	Width (sec)	Area (μV*sec)	Height (μV)	% Area
1	16.790	BB	99.000	4345477	151209	39.96
2	20.627	BB	120.000	1119190	33636	10.29
3	22.750	BB	114.000	4265598	121506	39.22
4	27.029	BB	107.000	1145383	28814	10.53

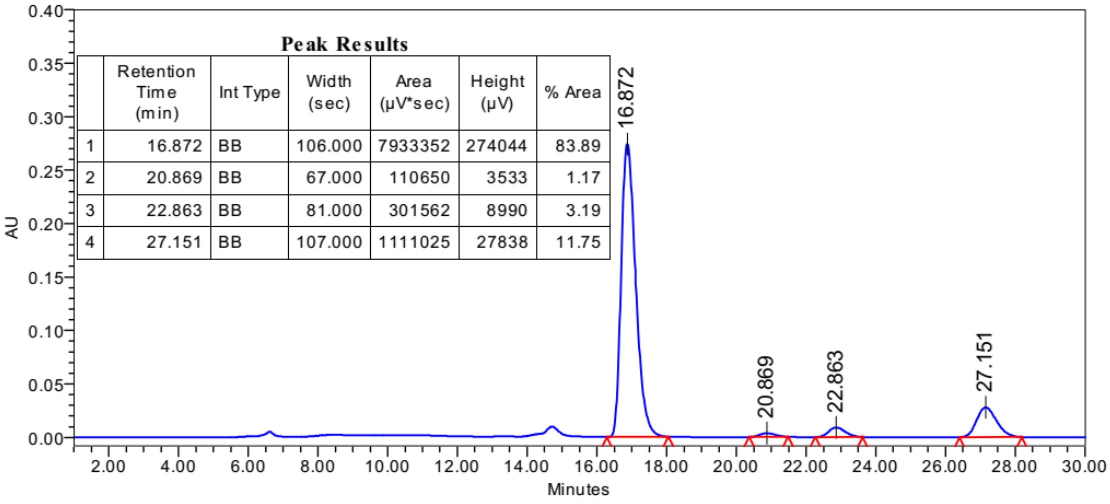

	Retention Time (min)	Int Type	Width (sec)	Area (µV*sec)	Height (µV)	% Area
1	16.872	BB	106.000	7933352	274044	83.89
2	20.869	BB	67.000	110650	3533	1.17
3	22.863	BB	81.000	301562	8990	3.19
4	27.151	BB	107.000	1111025	27838	11.75

(S)-2-(tert-butyl)-1-((4-methoxyphenyl)(4-nitrophenyl)methyl)-2,3-dihydro-1H-benzo[c][1,2]azaborole (**3x**)

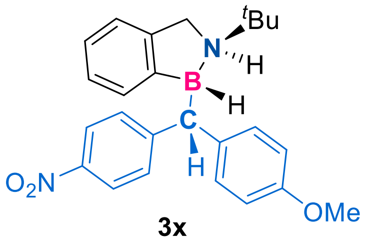

3x

Following the above procedure **E**, isolated yield: 48%, (20.0 mg), white solid (mp: 123.1 – 125.4 ºC), R_f = 0.3 (silica gel, hexane/DCM = 3:1, v/v), column chromatography (silica gel, hexane/DCM = 2:1, v/v).

^{1}H NMR (500 MHz, Acetone-*d*$_6$) δ 8.17 – 8.12 (m, 2H), 7.55 (d, *J* = 8.8 Hz, 2H), 7.08 – 6.99 (m, 4H), 6.91 – 6.86 (m, 1H), 6.81 – 6.76 (m, 2H), 6.49 (d, *J* = 7.3 Hz, 1H), 4.39 – 4.23 (m, 3H), 3.77 (s, 3H), 3.75 (s, 1H), 1.26 (s, 9H).

^{13}C NMR (126 MHz, Acetone-*d*$_6$) δ 159.2, 158.1, 145.1, 141.3, 137.1, 131.5, 129.9, 129.1, 126.2, 125.7, 123.3, 120.9, 114.0, 58.6, 54.9, 53.0, 26.1.

^{11}B NMR (160 MHz, Acetone-*d*$_6$) δ -1.5.

HRMS (ESI) m/z: $[M-H]^-$ Calcd. for $C_{25}H_{28}BN_2O_3$ 415.2198; Found: 415.2123.

HPLC analysis: DAICEL CHIRALCEL AD-H, hexane/isopropanol = 90/10, 1mL/min, λ = 254 nm, t_R (minor) = 12.445 min, t_2 = 16.694 min, t_3 = 17.426 min, t_4 = 19.765 min, 95% ee, dr = 20.5:1.

$[\alpha]^{25}_D$: +2.42 (*c* 0.33, $CHCl_3$).

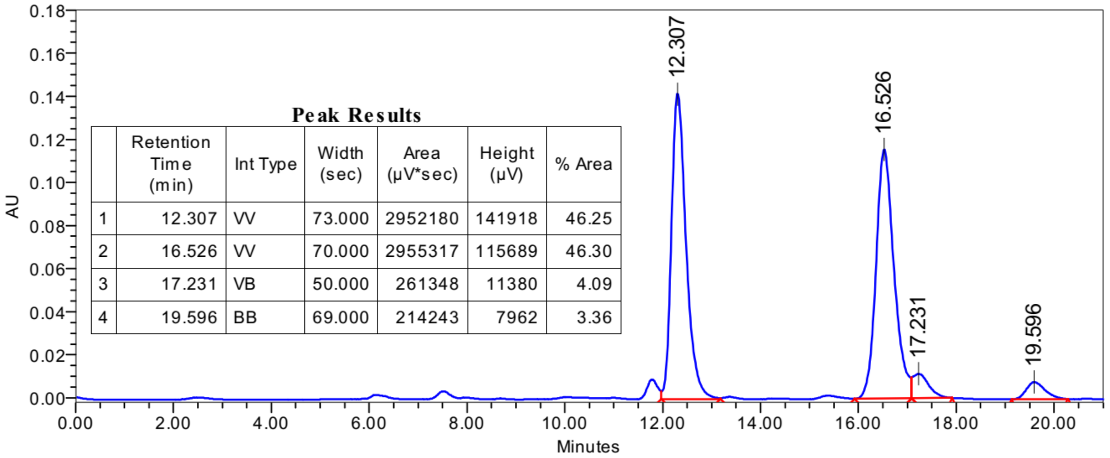

Peak Results

	Retention Time (min)	Int Type	Width (sec)	Area (μV*sec)	Height (μV)	% Area
1	12.307	VV	73.000	2952180	141918	46.25
2	16.526	VV	70.000	2955317	115689	46.30
3	17.231	VB	50.000	261348	11380	4.09
4	19.596	BB	69.000	214243	7962	3.36

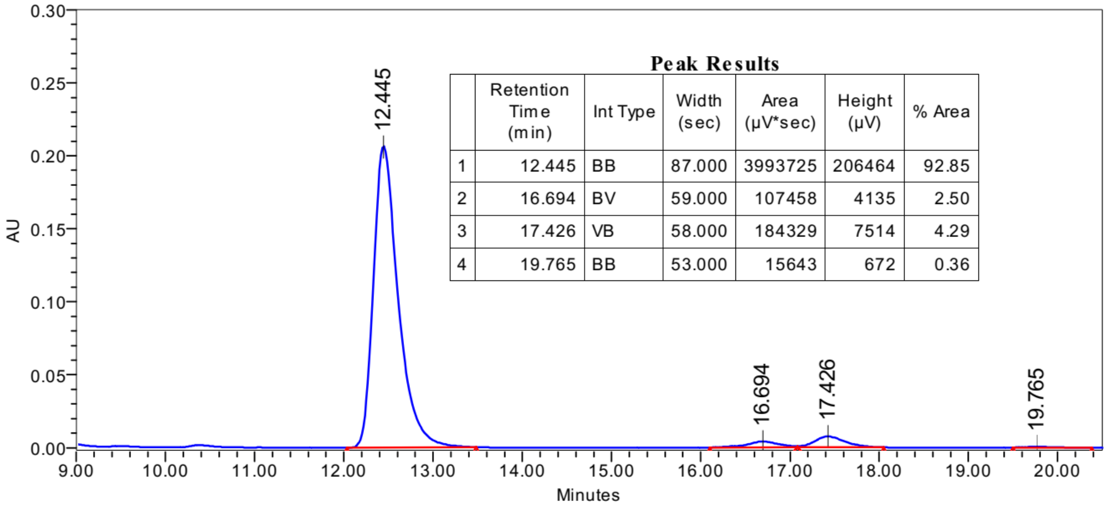

Peak Results

	Retention Time (min)	Int Type	Width (sec)	Area (μV*sec)	Height (μV)	% Area
1	12.445	BB	87.000	3993725	206464	92.85
2	16.694	BV	59.000	107458	4135	2.50
3	17.426	VB	58.000	184329	7514	4.29
4	19.765	BB	53.000	15643	672	0.36

(R)-2-(tert-butyl)-1-((4-methoxyphenyl)(3-nitrophenyl)methyl)-2,3-dihydro-1H-benzo[c][1,2]azaborole (**3y**)

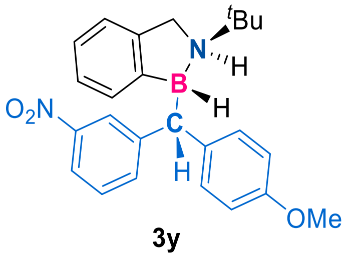

3y

Following the above procedure **E**, isolated yield: 51%, (21.2 mg), white solid (mp: 43.5 – 46.7 °C), R_f = 0.3 (silica gel, hexane/DCM = 3:1, v/v), column chromatography (silica gel, hexane/DCM = 2:1, v/v).

^{1}H NMR (500 MHz, Acetone-d_6) δ 8.20 (s, 1H), 7.97 (dd, J = 8.1, 2.0 Hz, 1H), 7.80 (d, J = 7.8 Hz, 1H), 7.52 (t, J = 7.9 Hz, 1H), 7.09 – 7.01 (m, 4H), 6.87 (t, J = 7.1 Hz, 1H), 6.78 (d, J = 8.4 Hz, 2H), 6.49 (d, J = 7.3 Hz, 1H), 4.45 – 4.24 (m, 3H), 3.76 (s, 3H), 3.72 (d, J = 5.6 Hz, 1H), 1.26 (s, 9H).

^{13}C NMR (126 MHz, Acetone-d_6) δ 158.1, 152.6, 148.7, 141.4, 137.7, 135.5, 131.4, 130.3, 130.0, 129.1, 126.2, 125.7, 123.0, 121.0, 119.2, 114.1, 58.6, 55.0, 53.1, 26.2.

^{11}B NMR (160 MHz, Acetone-d_6) δ -1.8.

HRMS (ESI) m/z: [M+H]$^+$ Calcd. for $C_{25}H_{30}BN_2O_3$ 417.2344; Found: 417.2343.

HPLC analysis: DAICEL CHIRALCEL IG, hexane/isopropanol = 90/10, 0.5 mL/min, λ = 267 nm, t_1 = 22.235 min, t_2 = 25.148 min, t_3 = 27.014 min, t_4 = 28.437 min, 90% ee, dr = 12.9:1.

[α]^{25}D: +19.77 (*c* 0.44, $CHCl_3$).

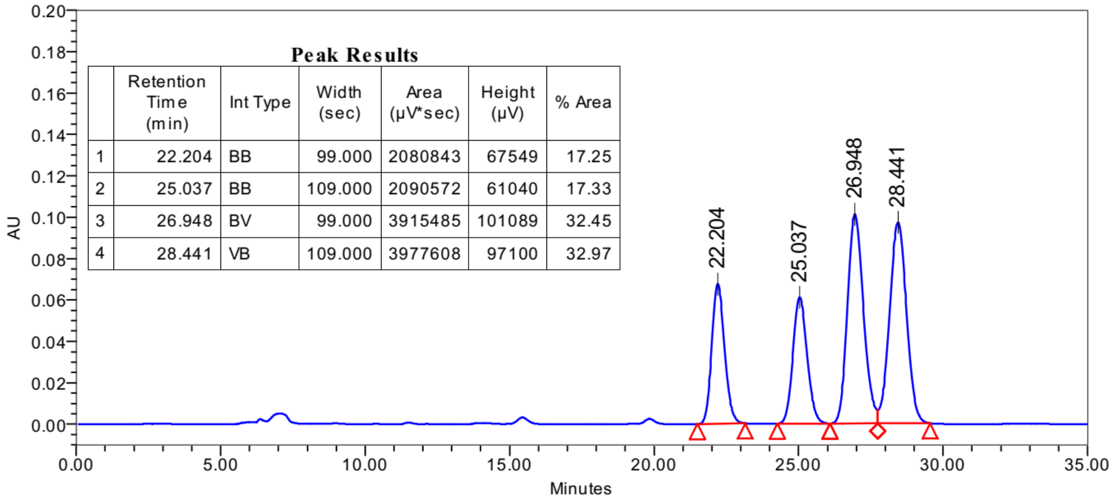

Peak Results

	Retention Time (min)	Int Type	Width (sec)	Area (µV*sec)	Height (µV)	% Area
1	22.204	BB	99.000	2080843	67549	17.25
2	25.037	BB	109.000	2090572	61040	17.33
3	26.948	BV	99.000	3915485	101089	32.45
4	28.441	VB	109.000	3977608	97100	32.97

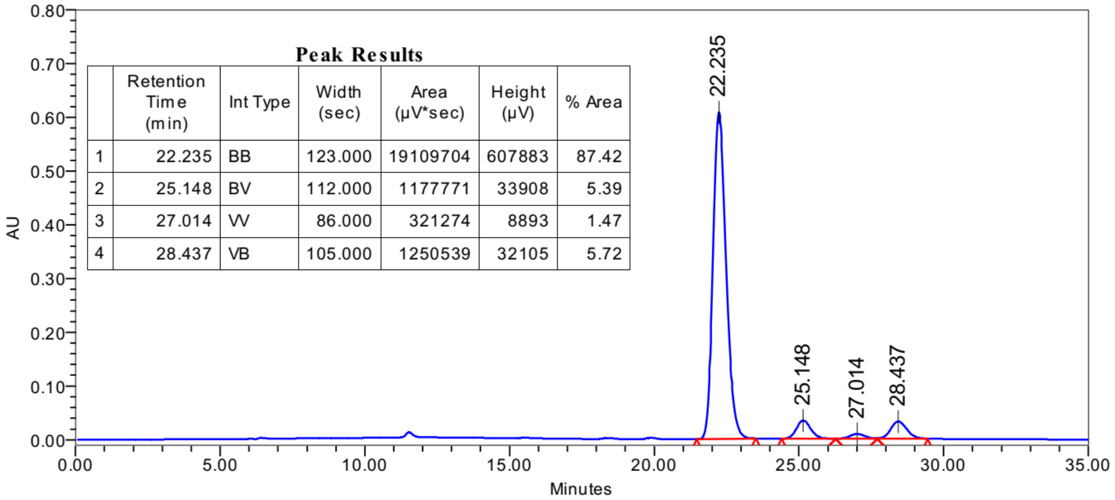

Peak Results

	Retention Time (min)	Int Type	Width (sec)	Area (µV*sec)	Height (µV)	% Area
1	22.235	BB	123.000	19109704	607883	87.42
2	25.148	BV	112.000	1177771	33908	5.39
3	27.014	VV	86.000	321274	8893	1.47
4	28.437	VB	105.000	1250539	32105	5.72

(R)-2-(tert-butyl)-1-((4-nitrophenyl)(p-tolyl)methyl)-2,3-dihydro-1H-benzo[c][1,2]azaborole (3z)

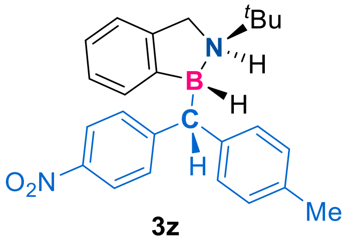

3z

Following the above procedure **E**, isolated yield: 70%, (28.0 mg), white solid (mp: 65.9 – 68.4 °C), R_f = 0.3 (silica gel, hexane/DCM = 3:1, v/v), column chromatography (silica gel, hexane/DCM = 2:1, v/v).

^{1}H NMR (500 MHz, $CDCl_3$) δ 8.10 (d, *J* = 8.7 Hz, 2H), 7.41 (d, *J* = 8.6 Hz, 2H), 7.08 (t, *J* = 7.3 Hz, 1H), 7.03 (d, *J* = 7.7 Hz, 2H), 6.98 (d, *J* = 7.5 Hz, 1H), 6.94 (t, *J* = 7.3 Hz, 1H), 6.78 (d, *J* = 7.8 Hz, 2H), 6.30 (d, *J* = 7.2 Hz, 1H), 4.08 (dd, *J* = 15.4, 4.7 Hz, 1H), 3.89 – 3.78 (m, 2H), 3.51 (s, 1H), 2.30 (s, 3H),

1.28 (s, 9H).

^{13}C NMR (126 MHz, $CDCl_3$) δ 156.9, 144.5, 140.3, 139.5, 135.2, 129.9, 129.5, 129.5, 128.1, 126.4, 125.6, 123.1, 120.4, 58.1, 52.8, 26.6, 20.9.

^{11}B NMR (160 MHz, $CDCl_3$) δ -1.7.

HRMS (ESI) m/z: $[M-H]^-$ Calcd. for $C_{25}H_{28}BN_2O_2$ 399.2249; Found: 399.2238.

HPLC analysis: DAICEL CHIRALCEL AD-H, hexane/isopropanol = 90/10, 0.5 mL/min, λ = 267 nm, t_1 = 9.634 min, t_2 = 10.873 min, t_3 = 12.483 min, t_4 = 13.304 min, 84% ee, dr = 5.5:1.

$[\alpha]^{25}_D$: -81.88 (*c* 0.32, $CHCl_3$).

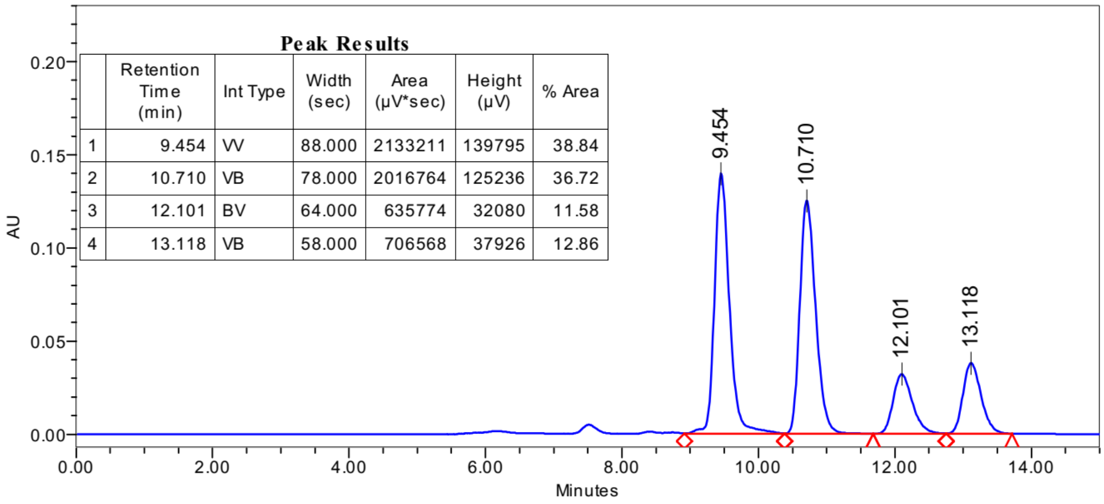

	Retention Time (min)	Int Type	Width (sec)	Area (μV*sec)	Height (μV)	% Area
1	9.454	VV	88.000	2133211	139795	38.84
2	10.710	VB	78.000	2016764	125236	36.72
3	12.101	BV	64.000	635774	32080	11.58
4	13.118	VB	58.000	706568	37926	12.86

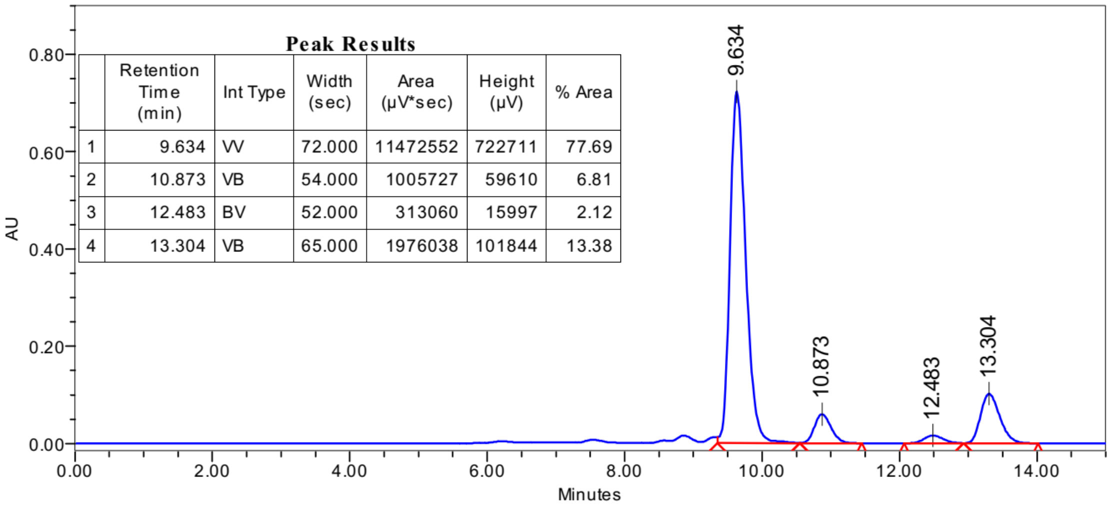

	Retention Time (min)	Int Type	Width (sec)	Area (μV*sec)	Height (μV)	% Area
1	9.634	VV	72.000	11472552	722711	77.69
2	10.873	VB	54.000	1005727	59610	6.81
3	12.483	BV	52.000	313060	15997	2.12
4	13.304	VB	65.000	1976038	101844	13.38

(S)-4-(benzo[d][1,3]dioxol-5-yl(2-(tert-butyl)-2,3-dihydro-1H-benzo[c][1,2]azaborol-1-yl)methyl)benzonitrile (3aa)

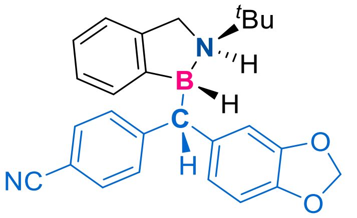

3aa

Following the above procedure **E**, isolated yield: 76%, (31.2 mg), white solid (mp: 61.6 – 63.4 °C), R_f = 0.3 (silica gel, hexane/DCM = 3:1, v/v), column chromatography (silica gel, hexane/DCM = 2:1, v/v).

^{1}H NMR (500 MHz, $CDCl_3$) δ 7.52 (d, *J* = 8.2 Hz, 2H), 7.34 (d, *J* = 8.2 Hz, 2H), 7.08 (t, *J* = 7.3 Hz, 1H), 6.99 (d, *J* = 7.5 Hz, 1H), 6.94 (t, *J* = 7.2 Hz, 1H), 6.69 (d, *J* = 7.9 Hz, 1H), 6.47 – 6.42 (m, 1H), 6.35 (d, *J* = 0.9 Hz, 1H), 6.31 (d, *J* = 7.3 Hz, 1H), 5.90 (d, *J* = 1.9 Hz, 2H), 4.13 (dd, *J* = 15.4, 4.5 Hz, 1H), 3.95 (dd, *J* = 15.4, 8.2 Hz, 1H), 3.69 (d, *J* = 5.4 Hz, 1H), 3.53 (s, 1H), 1.26 (s, 9H).

^{13}C NMR (126 MHz, $CDCl_3$) δ 154.3, 147.8, 145.4, 139.3, 137.6, 131.6, 129.5, 128.3, 126.5, 125.7, 122.9, 120.5, 119.9, 110.5, 108.4, 107.1, 100.7, 58.1, 52.8, 26.6.

^{11}B NMR (128 MHz, $CDCl_3$) δ -0.9.

HRMS (ESI) m/z: $[M+H]^+$ Calcd. for $C_{26}H_{28}BN_2O_2$ 411.2239; Found: 411.2234.

HPLC analysis: DAICEL CHIRALCEL IG, hexane/isopropanol = 90/10, 0.5 mL/min, λ = 267 nm, t_1 = 24.098 min, t_2 = 30.207 min, t_3 = 39.818 min, t_4 = 41.642 min, 96% ee, dr = 5.6:1.

$[\alpha]^{25}_D$: -23.75 (*c* 0.32, $CHCl_3$).

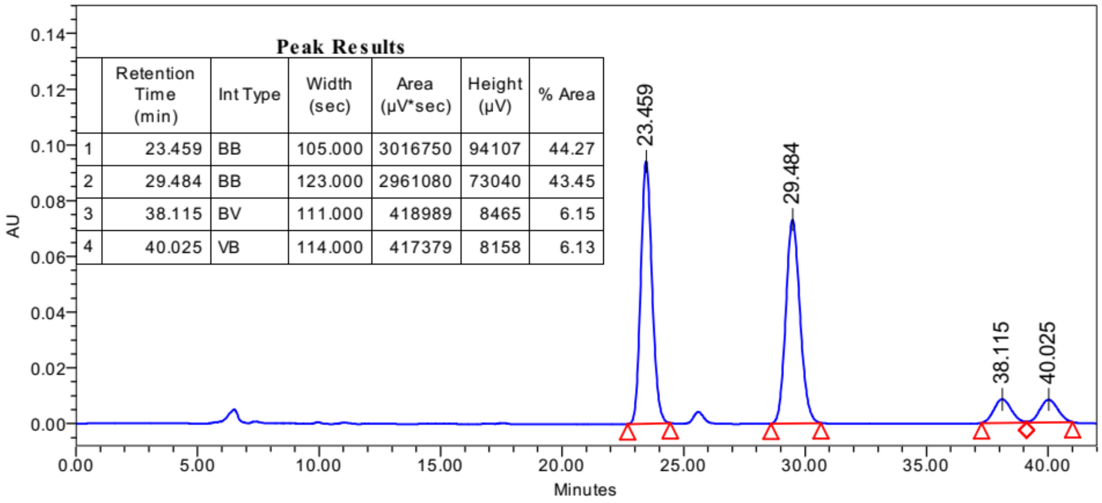

	Retention Time (min)	Int Type	Width (sec)	Area (μV*sec)	Height (μV)	% Area
1	23.459	BB	105.000	3016750	94107	44.27
2	29.484	BB	123.000	2961080	73040	43.45
3	38.115	BV	111.000	418989	8465	6.15
4	40.025	VB	114.000	417379	8158	6.13

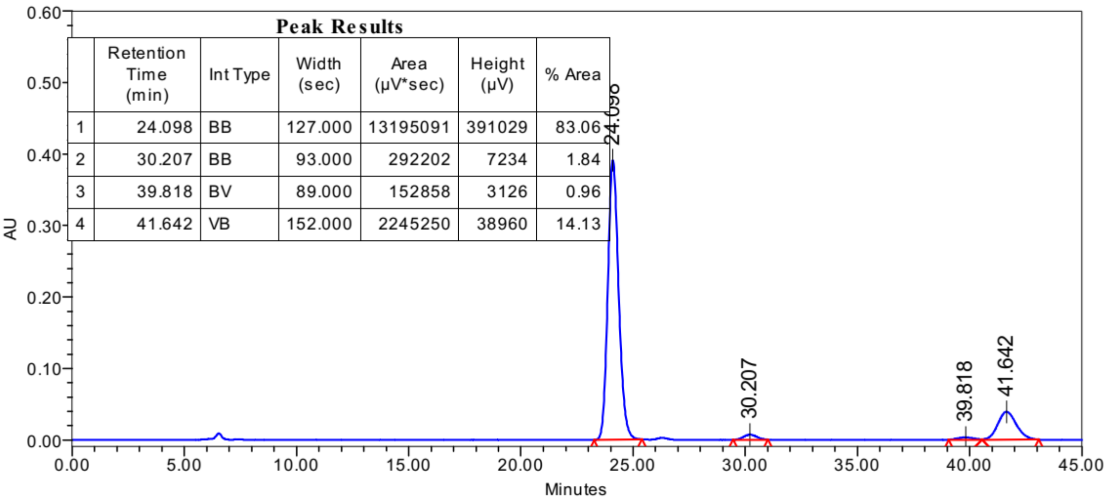

	Retention Time (min)	Int Type	Width (sec)	Area (µV*sec)	Height (µV)	% Area
1	24.098	BB	127.000	13195091	391029	83.06
2	30.207	BB	93.000	292202	7234	1.84
3	39.818	BV	89.000	152858	3126	0.96
4	41.642	VB	152.000	2245250	38960	14.13

(S)-4-((2-(tert-butyl)-5-methoxy-2,3-dihydro-1H-benzo[c][1,2]azaborol-1-yl)(4-methoxyphenyl)methyl)benzonitrile (**3ab**)

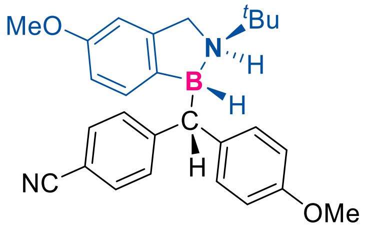

3ab

Following the above procedure **E**, isolated yield: 74%, (31.5 mg), white solid (mp: 92.2 – 93.9 ºC), R_f = 0.3 (silica gel, hexane/EtOAc = 3:1, v/v), column chromatography (silica gel, hexane/EtOAc = 2:1, v/v).

^{1}H NMR (400 MHz, $CDCl_3$) δ 7.50 (d, *J* = 8.3 Hz, 2H), 7.32 (d, *J* = 8.2 Hz, 2H), 6.80 (dd, *J* = 23.7, 8.7 Hz, 4H), 6.55 (d, *J* = 7.2 Hz, 2H), 6.16 (d, *J* = 8.5 Hz, 1H), 4.04 (dd, *J* = 15.5, 4.9 Hz, 1H), 3.81 (d, *J* = 8.6 Hz, 1H), 3.77 (s, 3H), 3.74 (s, 3H), 3.72 (d, *J* = 5.7 Hz, 1H), 3.49 (s, 1H), 1.25 (s, 9H).

^{13}C NMR (126 MHz, $CDCl_3$) δ 158.4, 157.5, 154.6, 140.8, 135.6, 131.5, 131.0, 130.3, 128.2, 120.0, 114.1, 112.5, 106.8, 106.2, 58.0, 55.1, 55.0, 52.7, 26.6.

^{11}B NMR (128 MHz, $CDCl_3$) δ -0.6.

HRMS (ESI) m/z: $[M+H]^+$ Calcd. for $C_{27}H_{31}BN_2O_2$ 426.2473; Found: 426.2440.

HPLC analysis: DAICEL CHIRALCEL OD-3, hexane/isopropanol = 90/10, 0.5 mL/min, λ = 267 nm, t_1 = 20.004 min, t_2 = 23.633 min, t_3 = 30.062 min, t_4 = 40.330 min, 87% ee, dr = 12.1:1.

$[\alpha]^{25}_D$: -44.84 (*c* 0.31, $CHCl_3$).

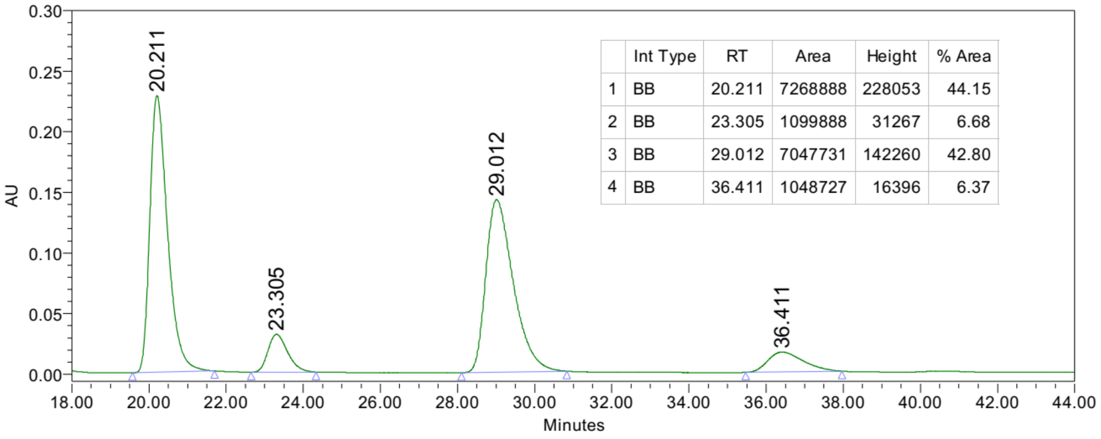

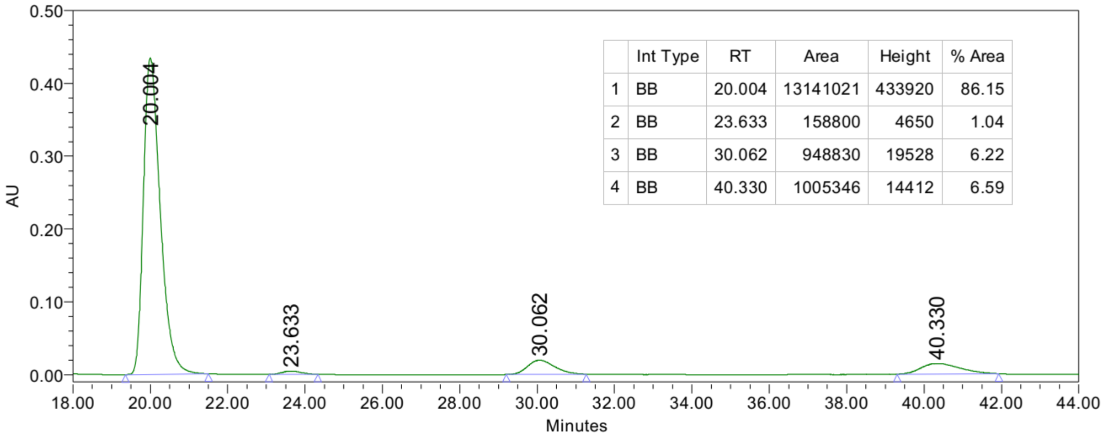

(S)-4-((2-(tert-butyl)-5-methyl-2,3-dihydro-1H-benzo[c][1,2]azaborol-1-yl)(4-methoxyphenyl)methyl)benzonitrile (3ac)

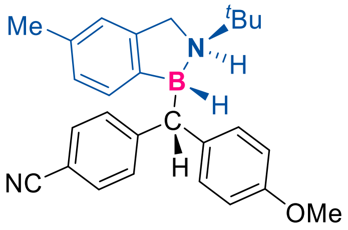

3ac

Following the above procedure **E**, isolated yield: 76%, (31.1 mg), white solid (mp: 79.7 – 82.1 °C), R_f = 0.3 (silica gel, hexane/EtOAc = 3:1, v/v), column chromatography (silica gel, hexane/EtOAc = 2:1, v/v).

^{1}H NMR (400 MHz, $CDCl_3$) δ 7.50 (d, J = 8.2 Hz, 2H), 7.32 (d, J = 8.2 Hz, 2H), 6.84 (d, J = 8.6 Hz, 2H), 6.78 (t, J = 8.3 Hz, 4H), 6.16 (d, J = 7.4 Hz, 1H), 4.05 (dd, J = 15.5, 4.7 Hz, 1H), 3.87 – 3.78 (m, 1H), 3.77 (s, 3H), 3.73 (d, J = 5.6 Hz, 1H), 3.47 (s, 1H), 2.27 (s, 3H), 1.24 (s, 9H).

^{13}C NMR (101 MHz, $CDCl_3$) δ 157.5, 154.6, 139.7, 135.7, 135.0, 131.5, 131.1, 129.4, 128.2, 127.5, 121.0, 120.0, 114.1, 106.8, 57.9, 55.1, 52.7, 26.6, 21.2.

^{11}B NMR (128 MHz, $CDCl_3$) δ -0.6.

HRMS (ESI) m/z: $[M+Cl]^-$ Calcd. for $C_{27}H_{30}BClN_2O$ 444.2145; Found: 444.2105.

HPLC analysis: DAICEL CHIRALCEL IG, hexane/isopropanol = 90/10, 0.5 mL/min, λ = 267 nm, t_1 = 20.834 min, t_2 = 26.024 min, t_3 = 35.478 min, t_4 = 39.639 min, 84% ee, dr = 5.5:1.

[α]^{25}$_D$: -39.39 (*c* 0.31, $CHCl_3$).

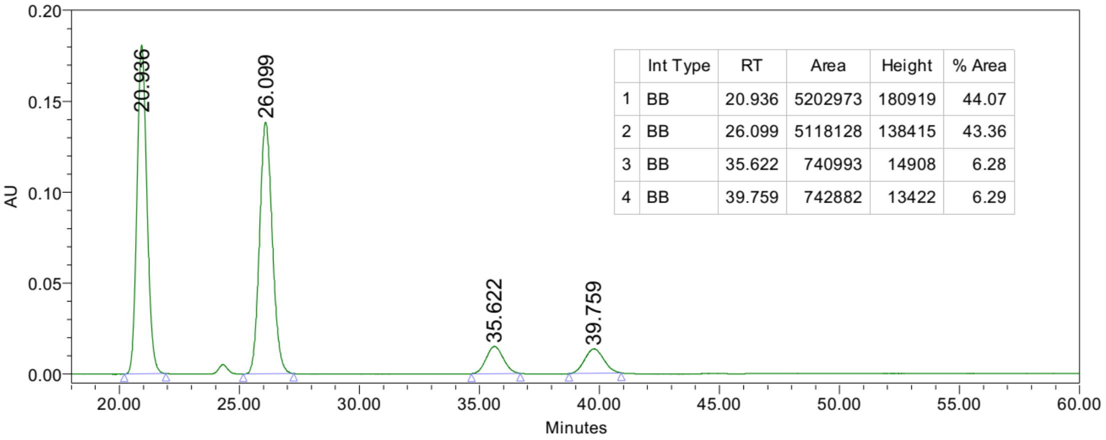

	Int Type	RT	Area	Height	% Area
1	BB	20.936	5202973	180919	44.07
2	BB	26.099	5118128	138415	43.36
3	BB	35.622	740993	14908	6.28
4	BB	39.759	742882	13422	6.29

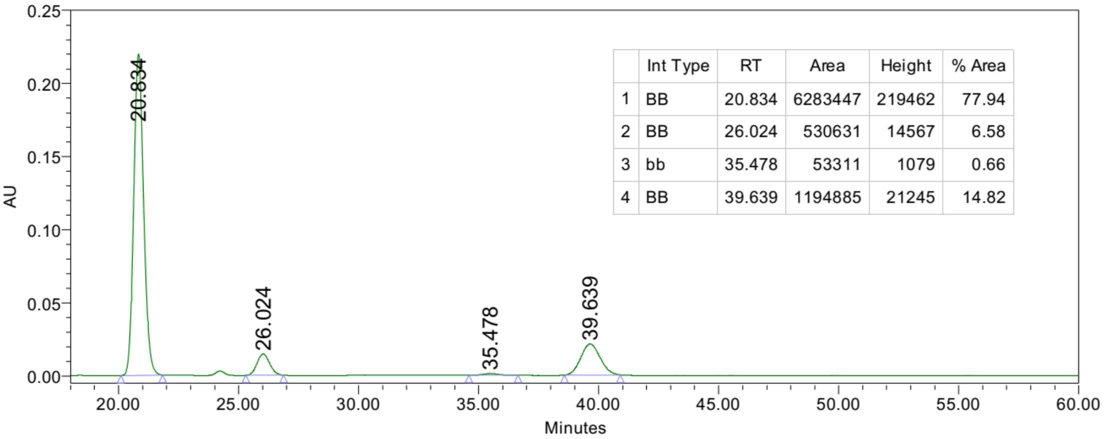

	Int Type	RT	Area	Height	% Area
1	BB	20.834	6283447	219462	77.94
2	BB	26.024	530631	14567	6.58
3	bb	35.478	53311	1079	0.66
4	BB	39.639	1194885	21245	14.82

(S)-4-((2-(tert-butyl)-5-chloro-2,3-dihydro-1H-benzo[c][1,2]azaborol-1-yl)(4-methoxyphenyl)methyl)benzonitrile (3ad)

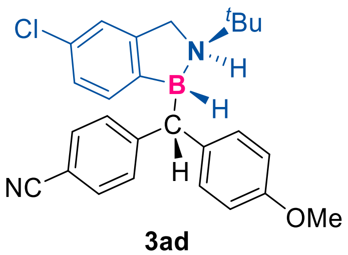

3ad

Following the above procedure **E**, isolated yield: 82%, (35.2 mg), white solid (mp: 67.3 – 69.1 °C), R_f = 0.3 (silica gel, hexane/DCM = 3:1, v/v), column chromatography (silica gel, hexane/DCM = 2:1, v/v).

^{1}H NMR (400 MHz, $CDCl_3$) δ 7.51 (d, *J* = 8.2 Hz, 2H), 7.32 (d, *J* = 8.2 Hz, 2H), 6.96 (s, 1H), 6.91 (d, *J* = 7.9 Hz, 1H), 6.80 (q, *J* = 8.7 Hz, 4H), 6.19 (d, *J* = 7.9 Hz, 1H), 4.04 (dd, *J* = 15.7, 4.5 Hz, 1H), 3.83 (d, *J* = 8.3 Hz, 1H), 3.77 (s, 3H), 3.70 (d, *J* = 5.6 Hz, 1H), 3.51 (s, 1H), 1.24 (s, 9H).

^{13}C NMR (101 MHz, $CDCl_3$) δ 157.6, 154.1, 141.2, 135.3, 131.6, 131.4, 130.9, 130.7, 128.2, 126.7,

120.6, 119.8, 114.2, 107.1, 58.2, 55.1, 52.2, 26.5.

^{11}B NMR (128 MHz, $CDCl_3$) δ -1.0.

HRMS (ESI) m/z: $[M+H]^+$ Calcd. for $C_{26}H_{28}BClN_2O$ 430.1978; Found: 430.1967.

HPLC analysis: DAICEL CHIRALCEL AD-H, hexane/isopropanol = 90/10, 0.5 mL/min, λ = 267 nm, t_1 = 13.812 min, t_2 = 19.555 min, t_3 = 21.817 min, t_4 = 24.218 min, 87% ee, dr = 4.2:1.

$[\alpha]^{25}_D$: -47.74 (*c* 0.31, $CHCl_3$).

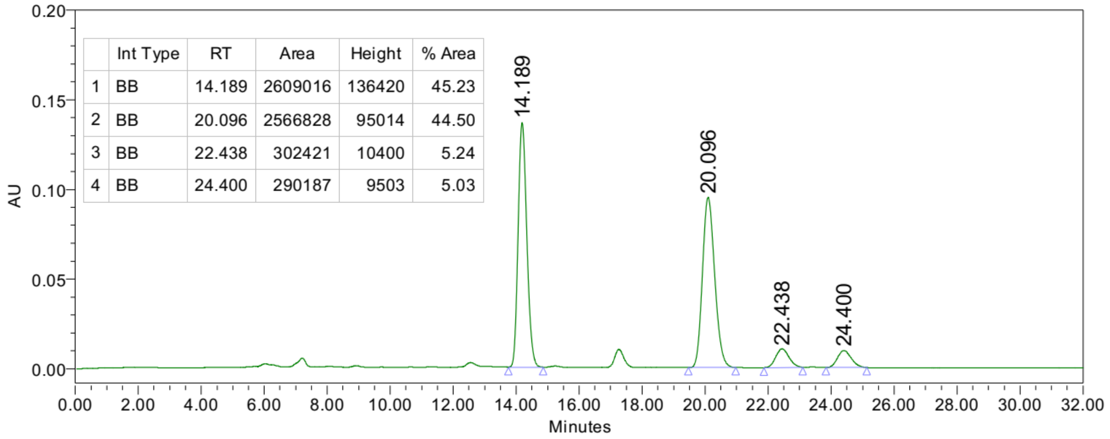

	Int Type	RT	Area	Height	% Area
1	BB	14.189	2609016	136420	45.23
2	BB	20.096	2566828	95014	44.50
3	BB	22.438	302421	10400	5.24
4	BB	24.400	290187	9503	5.03

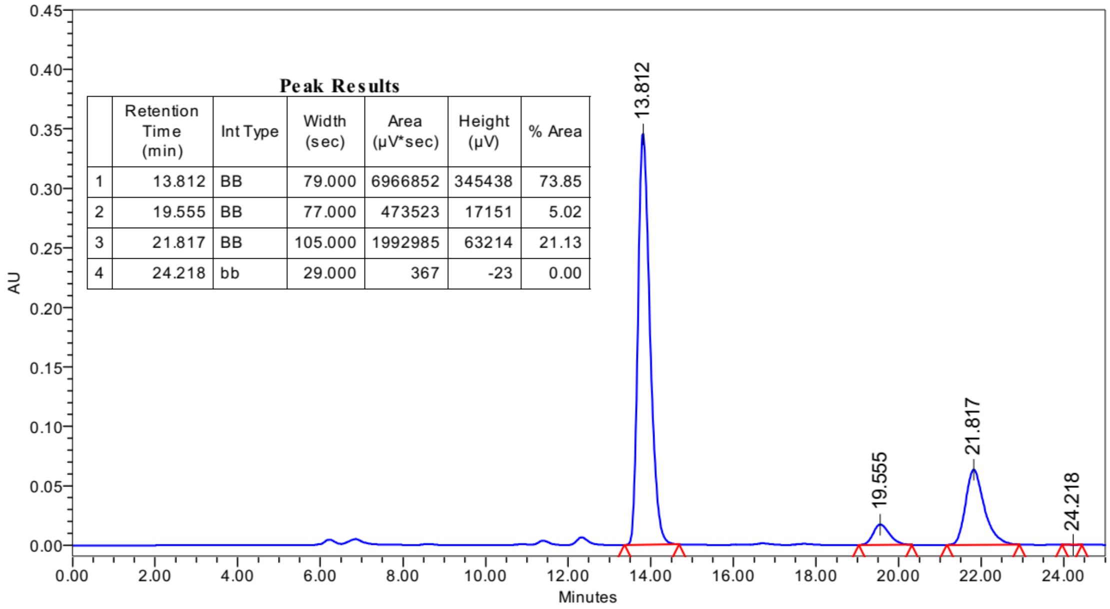

Peak Results

	Retention Time (min)	Int Type	Width (sec)	Area (μV*sec)	Height (μV)	% Area
1	13.812	BB	79.000	6966852	345438	73.85
2	19.555	BB	77.000	473523	17151	5.02
3	21.817	BB	105.000	1992985	63214	21.13
4	24.218	bb	29.000	367	-23	0.00

(S)-4-((2-(tert-butyl)-6-fluoro-2,3-dihydro-1H-benzo[c][1,2]azaborol-1-yl)(4-methoxyphenyl)methyl)benzonitrile (3ae)

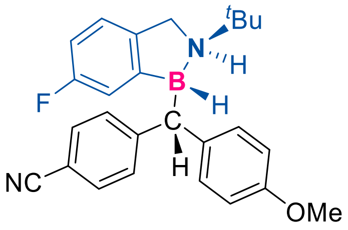

3ae

Following the above procedure **E**, isolated yield: 78%, (32.2 mg), white solid (mp: 149.6 – 152.4 °C), R_f = 0.3 (silica gel, hexane/EtOAc = 3:1, v/v), column chromatography (silica gel, hexane/EtOAc = 2:1, v/v).

^{1}H NMR (500 MHz, $CDCl_3$) δ 7.54 – 7.50 (m, 2H), 7.34 (d, *J* = 8.3 Hz, 2H), 6.91 (dd, *J* = 8.2, 4.8 Hz, 1H), 6.85 – 6.76 (m, 4H), 6.76 – 6.71 (m, 1H), 5.96 (dd, *J* = 9.4, 2.5 Hz, 1H), 4.05 (dd, *J* = 15.2, 4.6 Hz, 1H), 3.86 – 3.80 (m, 1H), 3.77 (s, 3H), 3.70 (d, *J* = 5.9 Hz, 1H), 3.53 (s, 1H), 1.24 (s, 9H).

^{13}C NMR (126 MHz, $CDCl_3$) δ 162.17 (d, *J* = 244.6 Hz), 157.6, 154.1, 135.3, 134.7, 131.6, 131.0, 129.7 (d, *J* = 53.3 Hz), 128.3, 121.58 (d, *J* = 8.0 Hz), 119.8, 115.5 (d, *J* = 19.2 Hz), 114.2, 112.7 (d, *J* = 23.3 Hz), 107.1, 58.2, 55.1, 52.2, 26.5.

^{11}B NMR (160 MHz, $CDCl_3$) δ -2.1.

^{19}F NMR (471 MHz, $CDCl_3$) δ -117.0.

HRMS (ESI) m/z: $[M+H]^+$ Calcd. for $C_{26}H_{28}BFN_2O$ 414.2273; Found: 414.2260.

HPLC analysis: DAICEL CHIRALCEL AD-H, hexane/isopropanol = 95/5, 0.5 mL/min, λ = 267 nm, t_1 = 23.261 min, t_2 = 30.476 min, t_3 = 31.854 min, t_4 = 34.919 min, 88% ee, dr = 3.6:1.

$[\alpha]^{25}_D$: -50.97 (*c* 0.31, $CHCl_3$).

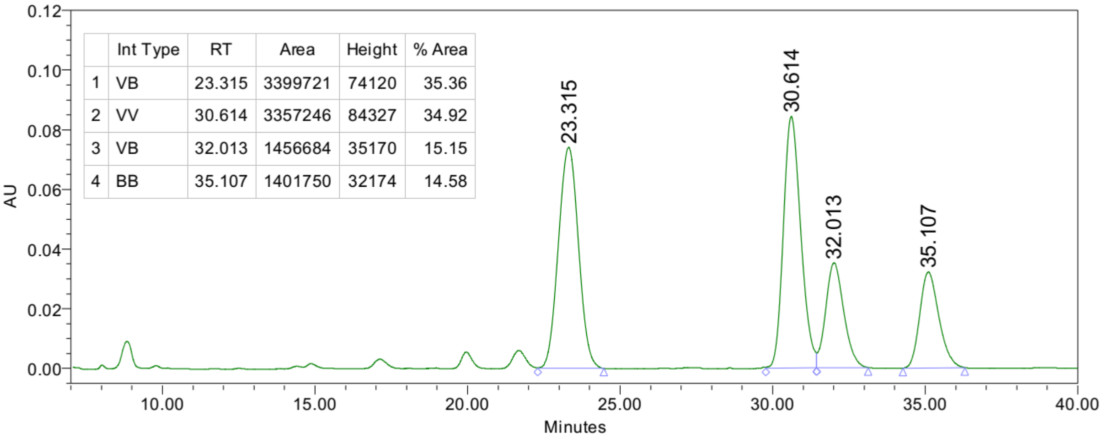

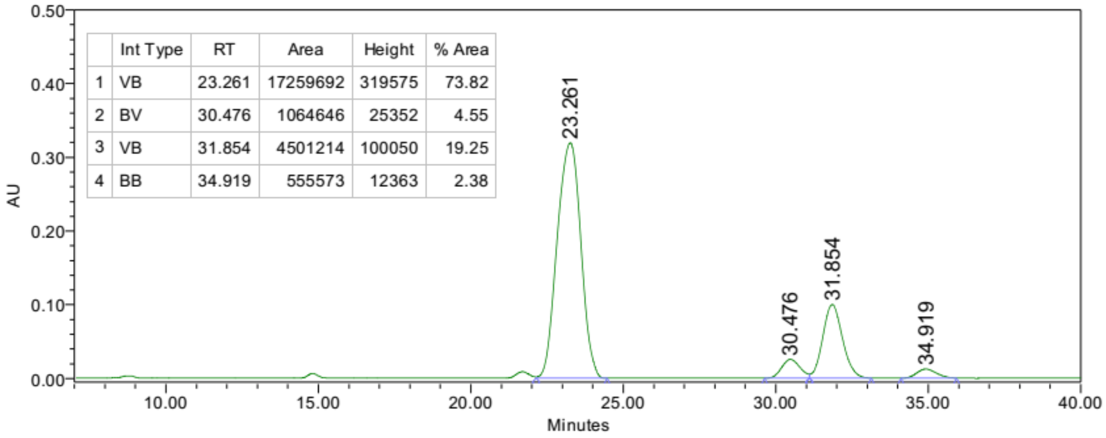

(S)-4-((4-methoxyphenyl)(2-(tert-pentyl)-2,3-dihydro-1H-benzo[c][1,2]azaborol-1-yl)methyl)benzonitrile (3af)

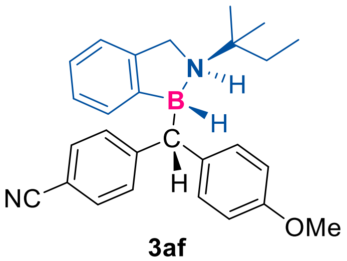

3af

Following the above procedure **E**, isolated yield: 80%, (32.7 mg), white solid (mp: 50.6 – 53.4 °C), R_f = 0.3 (silica gel, hexane/EtOAc = 3:1, v/v), column chromatography (silica gel, hexane/EtOAc = 2:1, v/v).

^{1}H NMR (500 MHz, $CDCl_3$) δ 7.53 – 7.49 (m, 2H), 7.34 (d, *J* = 8.3 Hz, 2H), 7.09 – 7.06 (m, 1H), 6.99 – 6.92 (m, 2H), 6.84 – 6.74 (m, 4H), 6.27 (d, *J* = 7.3 Hz, 1H), 4.05 (dd, *J* = 14.5, 3.7 Hz, 1H), 3.76 (s, 3H), 3.76 – 3.70 (m, 3H), 1.67 – 1.60 (m, 1H), 1.58 – 1.51 (m, 1H), 1.28 (s, 3H), 1.16 (s, 3H), 0.72 (t, *J* = 7.5 Hz, 3H).

^{13}C NMR (126 MHz, $CDCl_3$) δ 157.6, 154.4, 139.5, 135.6, 131.5, 131.1, 129.5, 128.3, 126.3, 125.5, 120.3, 120.0, 114.1, 106.8, 60.5, 55.1, 52.0, 31.9, 23.8, 22.8, 7.6.

^{11}B NMR (160 MHz, $CDCl_3$) δ -2.1.

HRMS (ESI) m/z: $[M+H]^+$ Calcd. for $C_{27}H_{31}BN_2O$ 410.2524; Found: 410.2501.

HPLC analysis: DAICEL CHIRALCEL AD-H, hexane/isopropanol = 90/10, 0.5 mL/min, λ = 267 nm, t_1 = 11.901 min, t_2 = 16.855 min, t_3 = 18.090 min, t_4 = 23.213 min, 90% ee, dr = 7.3:1.

$[\alpha]^{25}_{D}$: -62.67 (*c* 0.31, $CHCl_3$).

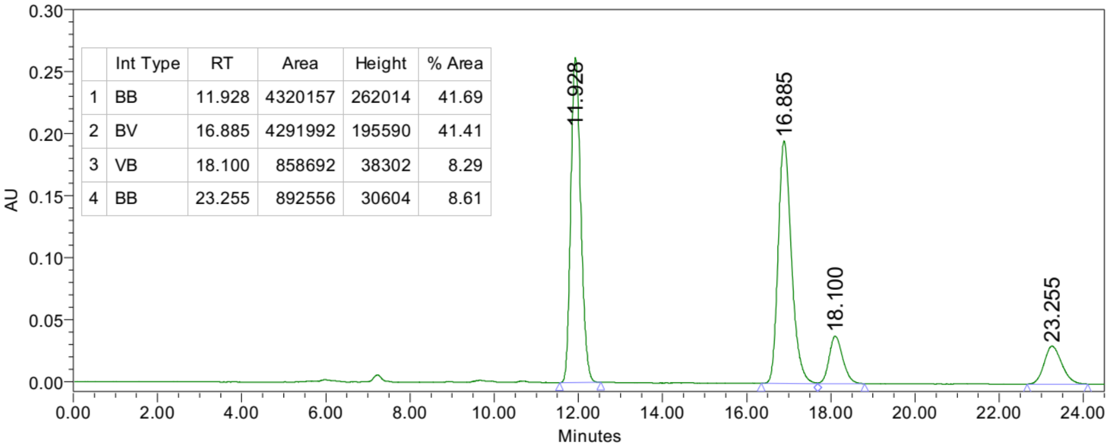

	Int Type	RT	Area	Height	% Area
1	BB	11.928	4320157	262014	41.69
2	BV	16.885	4291992	195590	41.41
3	VB	18.100	858692	38302	8.29
4	BB	23.255	892556	30604	8.61

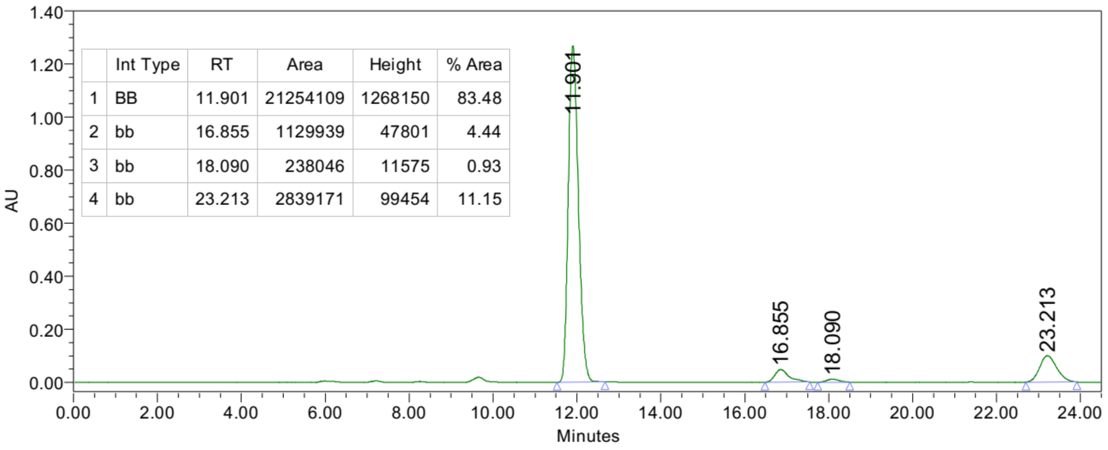

(S)-4-((4-methoxyphenyl)(2-(2,4,4-trimethylpentan-2-yl)-2,3-dihydro-1H-benzo[c][1,2]azaborol-1-yl)methyl)benzonitrile (3ag)

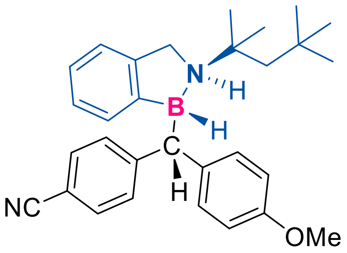

3ag

Following the above procedure **E**, isolated yield: 82%, (37.0 mg), white solid (mp: 71.1 – 74.3 ºC), R_f = 0.3 (silica gel, hexane/DCM = 3:1, v/v), column chromatography (silica gel, hexane/DCM = 2:1, v/v).

^{1}H NMR (400 MHz, $CDCl_3$) δ 7.51 (d, *J* = 8.3 Hz, 2H), 7.31 (d, *J* = 8.2 Hz, 2H), 7.08 (t, *J* = 7.3 Hz, 1H), 6.99 (d, *J* = 7.4 Hz, 1H), 6.93 (t, *J* = 7.2 Hz, 1H), 6.78 (dd, *J* = 21.7, 8.7 Hz, 4H), 6.17 (d, *J* = 7.3 Hz, 1H), 4.12 (q, *J* = 8.7 Hz, 1H), 3.86 (s, 1H), 3.81 (dd, *J* = 13.3, 7.2 Hz, 2H), 3.76 (s, 3H), 1.58 (s, 2H), 1.49 (s, 3H), 1.25 (s, 3H), 0.88 (s, 9H).

^{13}C NMR (101 MHz, $CDCl_3$) δ 157.7, 154.5, 139.5, 135.8, 131.5, 131.2, 129.6, 128.3, 126.4, 125.5, 120.3, 120.0, 114.3, 106.9, 62.1, 55.2, 52.7, 50.5, 31.3, 26.5, 25.4.

^{11}B NMR (128 MHz, $CDCl_3$) δ -0.4.

HRMS (ESI) m/z: $[M+H]^+$ Calcd. for $C_{30}H_{37}BN_2O$ 452.2994; Found: 452.2975.

HPLC analysis: DAICEL CHIRALCEL AD-H, hexane/isopropanol = 95/5, 0.5 mL/min, λ = 267 nm, t_1 = 11.280 min, t_2 = 15.452 min, t_3 = 17.217 min, t_4 = 19.483 min, 88% ee, dr = 5.7:1.

$[\alpha]^{25}_D$: -40.67 (*c* 0.3, $CHCl_3$).

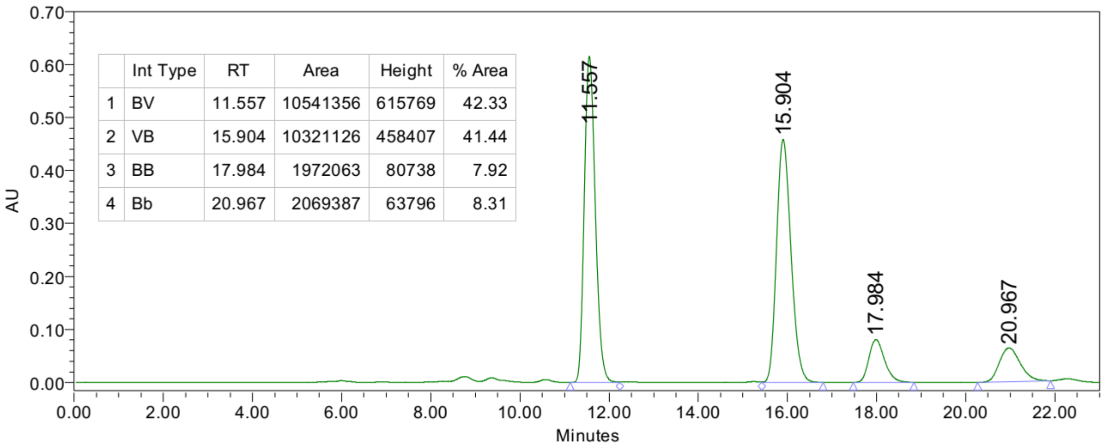

	Int Type	RT	Area	Height	% Area
1	BV	11.557	10541356	615769	42.33
2	VB	15.904	10321126	458407	41.44
3	BB	17.984	1972063	80738	7.92
4	Bb	20.967	2069387	63796	8.31

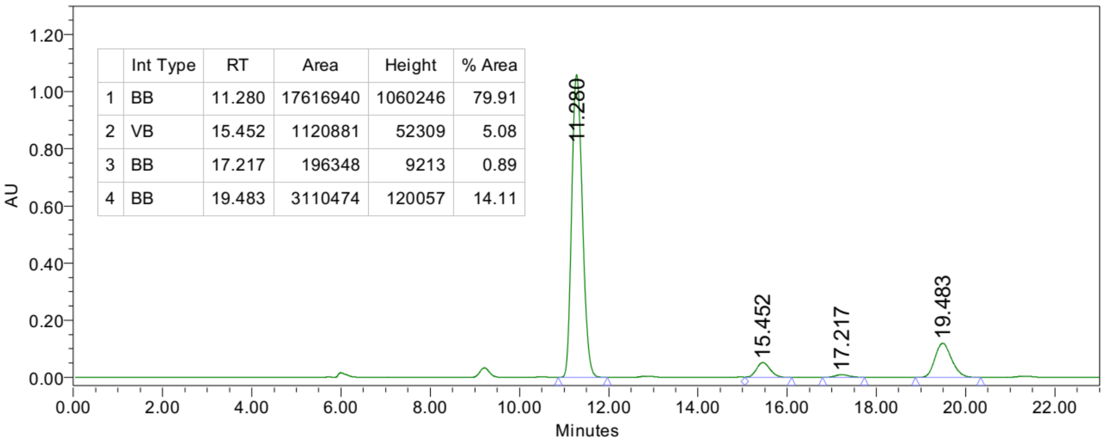

	Int Type	RT	Area	Height	% Area
1	BB	11.280	17616940	1060246	79.91
2	VB	15.452	1120881	52309	5.08
3	BB	17.217	196348	9213	0.89
4	BB	19.483	3110474	120057	14.11

(S)-4-((4-methoxyphenyl)(2-(2-phenylpropan-2-yl)-2,3-dihydro-1H-benzo[c][1,2]azaborol-1-yl)methyl)benzonitrile (**3ah**)

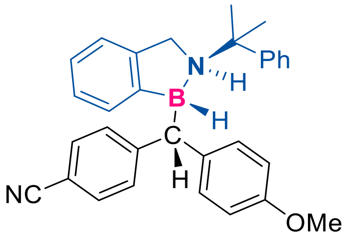

3ah

Following the above procedure **E**, isolated yield: 72%, (32.9 mg), white solid (mp: 81.3 – 84.9 °C), R_f = 0.3 (silica gel, hexane/DCM = 3:1, v/v), column chromatography (silica gel, hexane/DCM = 2:1, v/v).

^{1}H NMR (500 MHz, $CDCl_3$) δ 7.55 (d, *J* = 8.3 Hz, 2H), 7.39 – 7.36 (m, 5H), 7.10 – 7.06 (m, 2H), 7.05 – 7.02 (m, 1H), 7.00 – 6.92 (m, 3H), 6.84 – 6.82(m, 3H), 6.41 (d, *J* = 7.3 Hz, 1H), 4.35 (d, *J* = 4.0 Hz, 1H), 3.80 (s, 3H), 3.67 – 3.61 (m, 2H), 3.52 (dd, *J* = 15.8, 8.1 Hz, 1H), 1.56 (s, 3H), 1.54 (s, 3H).

^{13}C NMR (101 MHz, $CDCl_3$) δ 157.7, 154.8, 143.9, 139.6, 136.0, 131.8, 131.1, 129.7, 129.2, 128.2, 128.1, 126.4, 125.6, 124.8, 120.4, 119.9, 114.1, 107.2, 62.6, 55.2, 54.4, 28.3, 20.2.

^{11}B NMR (128 MHz, $CDCl_3$) δ -0.9.

HRMS (ESI) m/z: $[M+H]^+$ Calcd. for $C_{31}H_{31}BN_2O$ 458.2524; Found: 458.2517.

HPLC analysis: DAICEL CHIRALCEL AD-H, hexane/isopropanol = 90/10, 0.5 mL/min, λ = 267 nm, t_1 = 12.277 min, t_2 = 15.807 min, t_3 = 18.464 min, t_4 = 20.661 min, 89% ee, dr = 5.2:1.

$[\alpha]^{25}_{D}$: -34.19 (*c* 0.31, $CHCl_3$).

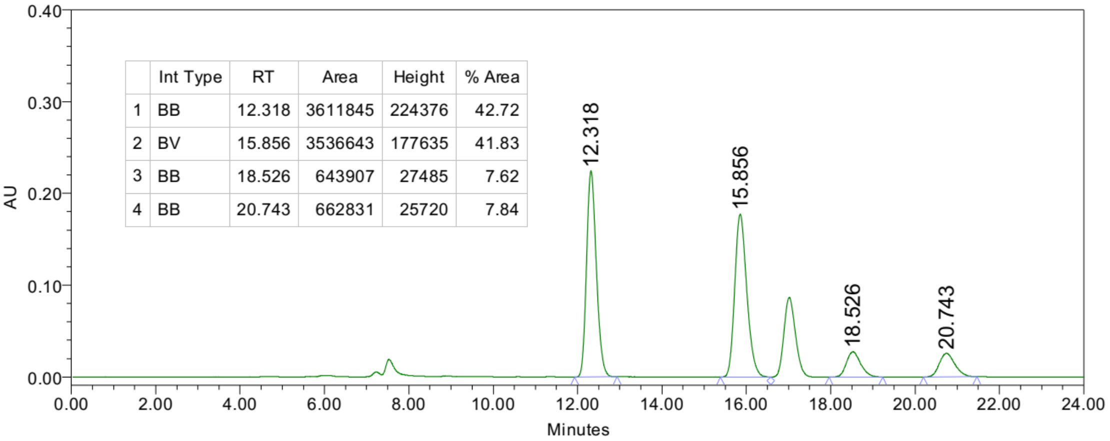

	Int Type	RT	Area	Height	% Area
1	BB	12.318	3611845	224376	42.72
2	BV	15.856	3536643	177635	41.83
3	BB	18.526	643907	27485	7.62
4	BB	20.743	662831	25720	7.84

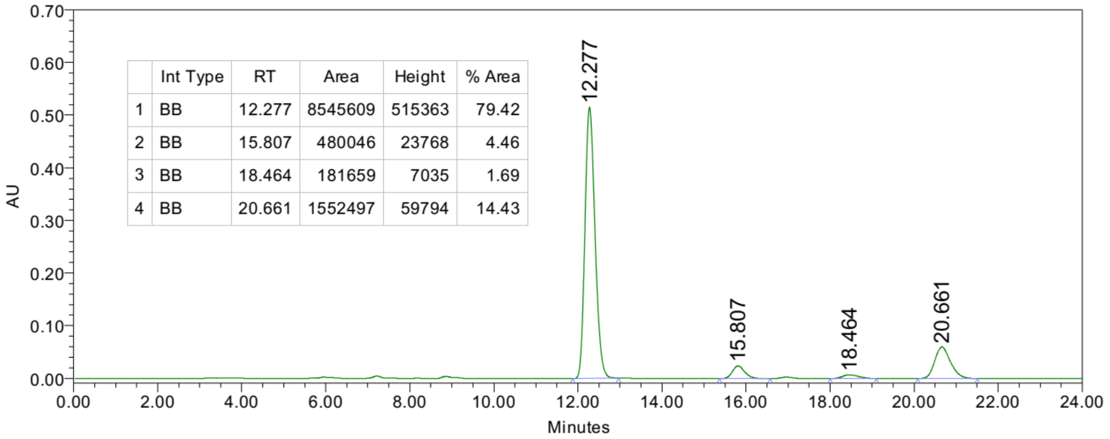

	Int Type	RT	Area	Height	% Area
1	BB	12.277	8545609	515363	79.42
2	BB	15.807	480046	23768	4.46
3	BB	18.464	181659	7035	1.69
4	BB	20.661	1552497	59794	14.43

(S)-4-((2-(1-hydroxy-2-methylpropan-2-yl)-2,3-dihydro-1H-benzo[c][1,2]azaborol-1-yl)(4-methoxyphenyl)methyl)benzonitrile (**3ai**)

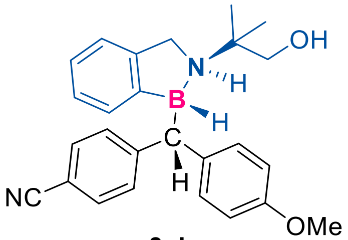

3ai

Following the above procedure **E**, isolated yield: 56%, (23.0 mg), white solid (mp: 64.6 – 67.9 ºC), R_f = 0.3 (silica gel, hexane/DCM = 3:1, v/v), column chromatography (silica gel, hexane/DCM = 2:1, v/v).

^{1}H NMR (400 MHz, $CDCl_3$) δ 7.43 (d, *J* = 8.1 Hz, 2H), 7.34 (d, *J* = 8.4 Hz, 2H), 7.25 (d, *J* = 8.0 Hz, 2H), 7.03 (q, *J* = 7.4 Hz, 2H), 6.92 (t, *J* = 6.9 Hz, 1H), 6.87 (d, *J* = 8.4 Hz, 2H), 6.59 (d, *J* = 7.2 Hz, 1H), 4.31 – 4.21 (m, 2H), 4.13 (d, *J* = 13.4 Hz, 1H), 3.77 (s, 3H), 3.73 (d, *J* = 10.4 Hz, 1H), 3.33 (d, *J* = 11.2 Hz, 1H), 3.22 (d, *J* = 6.8 Hz, 1H), 1.06 (s, 3H), 0.93 (s, 3H).

^{13}C NMR (101 MHz, $CDCl_3$) δ 157.3, 153.6, 139.6, 139.5, 131.5, 130.0, 129.6, 129.1, 126.3, 125.5, 120.4, 119.8, 114.1, 107.6, 67.1, 60.7, 55.3, 51.3, 21.2, 20.8.

^{11}B NMR (128 MHz, $CDCl_3$) δ -0.8.

HRMS (ESI) m/z: $[M+Cl]^-$ Calcd. for $C_{26}H_{28}BClN_2O_2$ 446.1938; Found: 446.1923.

HPLC analysis: DAICEL CHIRALCEL AD-H, hexane/isopropanol = 90/10, 0.5 mL/min, λ = 267 nm, t_1 = 20.322 min, t_2 = 24.468 min, t_3 = 25.647 min, t_4 = 36.064 min, 87% ee, dr =3.8:1.

$[\alpha]^{25}_D$: -13.75 (*c* 0.32, $CHCl_3$).

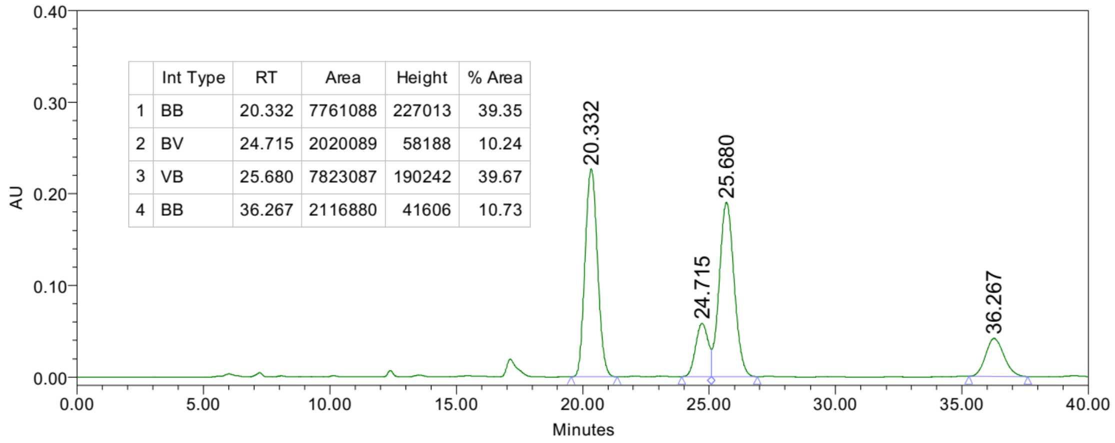

	Int Type	RT	Area	Height	% Area
1	BB	20.332	7761088	227013	39.35
2	BV	24.715	2020089	58188	10.24
3	VB	25.680	7823087	190242	39.67
4	BB	36.267	2116880	41606	10.73

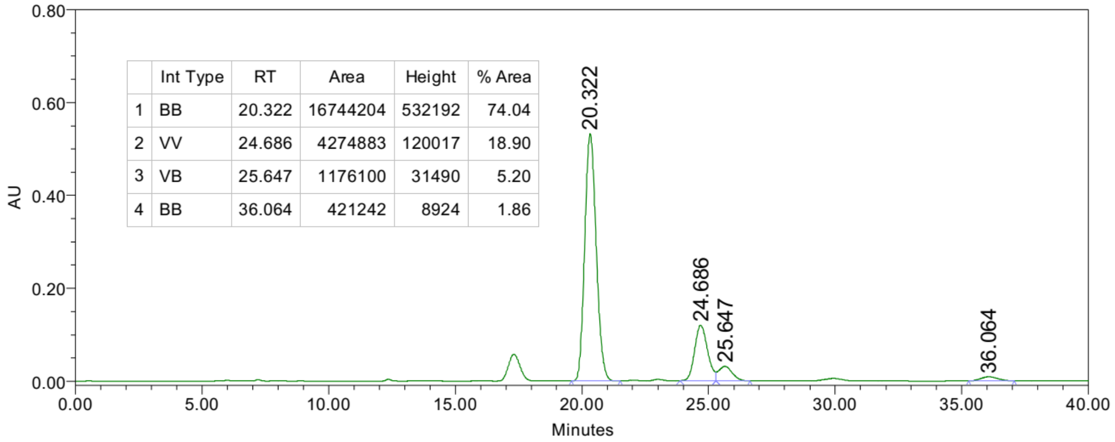

	Int Type	RT	Area	Height	% Area
1	BB	20.322	16744204	532192	74.04
2	VV	24.686	4274883	120017	18.90
3	VB	25.647	1176100	31490	5.20
4	BB	36.064	421242	8924	1.86

(S)-4-((4-methoxyphenyl)(2-(3-methylbenzyl)-2,3-dihydro-1H-benzo[c][1,2]azaborol-1-yl)methyl)benzonitrile (3aj)

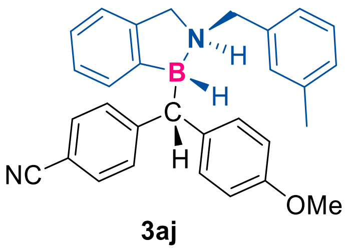

3aj

Following the above procedure **E**, isolated yield: 83%, (36.8 mg), white solid (mp: 65.7 – 67.8 ºC), R_f = 0.3 (silica gel, hexane/DCM = 3:1, v/v), column chromatography (silica gel, hexane/DCM = 2:1, v/v).

^{1}H NMR (400 MHz, $CDCl_3$) δ 7.53 (d, *J* = 8.2 Hz, 2H), 7.44 (d, *J* = 8.1 Hz, 2H), 7.26 – 7.20 (m, 1H), 7.16 (d, *J* = 7.6 Hz, 1H), 7.07 – 6.94 (m, 5H), 6.83 (d, *J* = 8.4 Hz, 2H), 6.77 (d, *J* = 7.2 Hz, 1H), 6.63 (s, 1H), 6.46 (d, *J* = 7.0 Hz, 1H), 4.09 (d, *J* = 11.3 Hz, 1H), 3.93 – 3.81 (m, 3H), 3.78 (s, 3H), 3.75 – 3.66 (m, 2H), 2.31 (s, 3H).

^{13}C NMR (126 MHz, $CDCl_3$) δ 157.7, 154.7, 139.2, 138.3, 135.9, 134.1, 131.8, 131.0, 129.7, 129.6, 129.3, 129.1, 128.4, 127.2, 125.8, 125.6, 121.4, 119.8, 114.3, 107.3, 56.6, 56.2, 55.2, 21.2.

^{11}B NMR (160 MHz, $CDCl_3$) δ -1.1.

HRMS (ESI) m/z: $[M+Cl]^-$ Calcd. for $C_{30}H_{29}BN_2O$ 444.2368; Found: 444.2325.

HPLC analysis: DAICEL CHIRALCEL AD-H, hexane/isopropanol = 95/5, 0.5 mL/min, λ = 267 nm, t_1 = 31.487 min, t_2 = 33.304 min, t_3 = 44.126 min, t_4 = 49.178 min, 88% ee, dr = 1.3:1.

$[\alpha]^{25}_{D}$: +25.63 (*c* 0.32, $CHCl_3$).

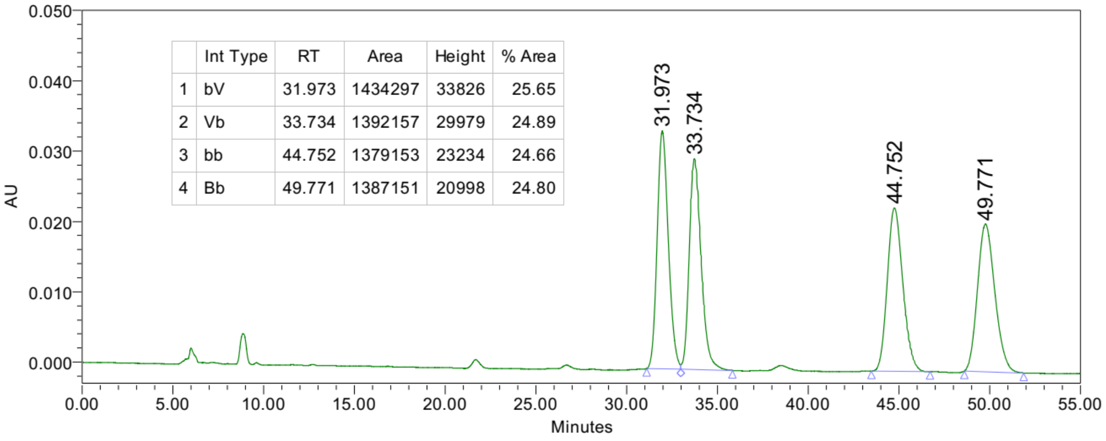

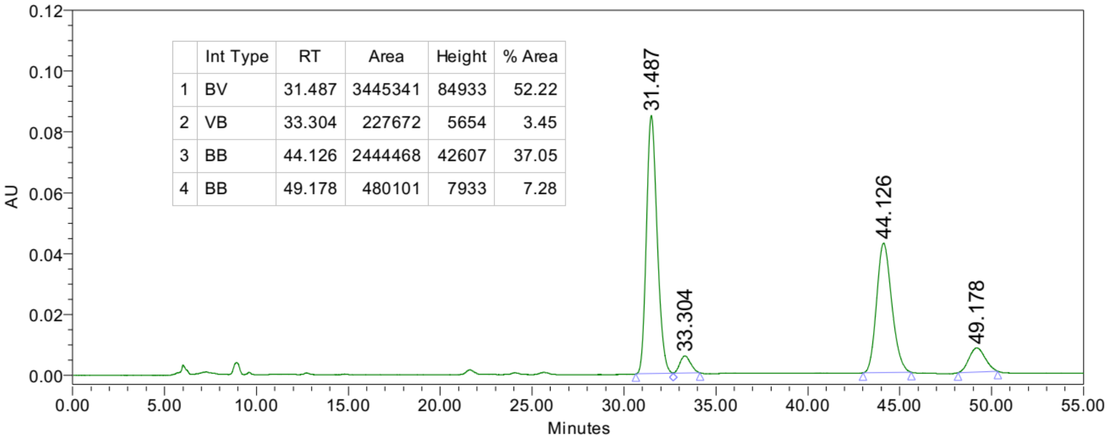

1-benzhydryl-2-(tert-butyl)-1,2,3,4-tetrahydrobenzo[c][1,2]azaborinine (3ak)

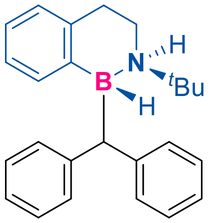

3ak

Following the above procedure **E**, isolated yield: 61%, (21.7 mg), white solid (mp: 91.2 – 94.0 ºC), R_f = 0.3 (silica gel, hexane/DCM = 5:1, v/v), column chromatography (silica gel, hexane/DCM = 3:1, v/v).

^{1}H NMR (500 MHz, $CDCl_3$) δ 7.43 (dd, *J* = 8.1, 1.1 Hz, 2H), 7.29 – 7.25 (m, 2H), 7.25 – 7.22 (m, 2H), 7.18 – 7.14 (m, 2H), 7.12 – 7.08 (m, 1H), 7.08 – 7.04 (m, 1H),6.97 – 6.92(m, 2H), 6.80 – 6.77 (m, 1H), 6.61 (d, *J* = 7.5 Hz, 1H), 3.40 – 3.34 (m, 1H), 3.29 (d, *J* = 7.1 Hz, 1H), 3.16 (s, 1H), 2.99 – 2.93(m, 1H), 2.92 – 2.86 (m, 1H), 2.83 – 2.76 (m, 1H), 1.16 (s, 9H).

^{13}C NMR (126 MHz, $CDCl_3$) δ 148.1, 147.1, 137.5, 133.9, 129.9, 128.5, 128.4, 127.6, 125.9, 124.7, 124.5, 124.3, 124.3, 58.7, 43.0, 29.3, 27.4.

^{11}B NMR (160 MHz, $CDCl_3$) δ -6.9.

HRMS (ESI) m/z: $[M-H]^-$ Calcd. for $C_{25}H_{29}BN$ 354.2398; Found: 354.2387.

HPLC analysis: DAICEL CHIRALCEL IG, hexane/isopropanol = 98/2, 0.5 mL/min, λ = 267 nm, t_1= 11.708 min, t_2 = 12.529 min, 42% ee.

$[\alpha]^{25}_D$: -2.58 (*c* 0.31, $CHCl_3$).

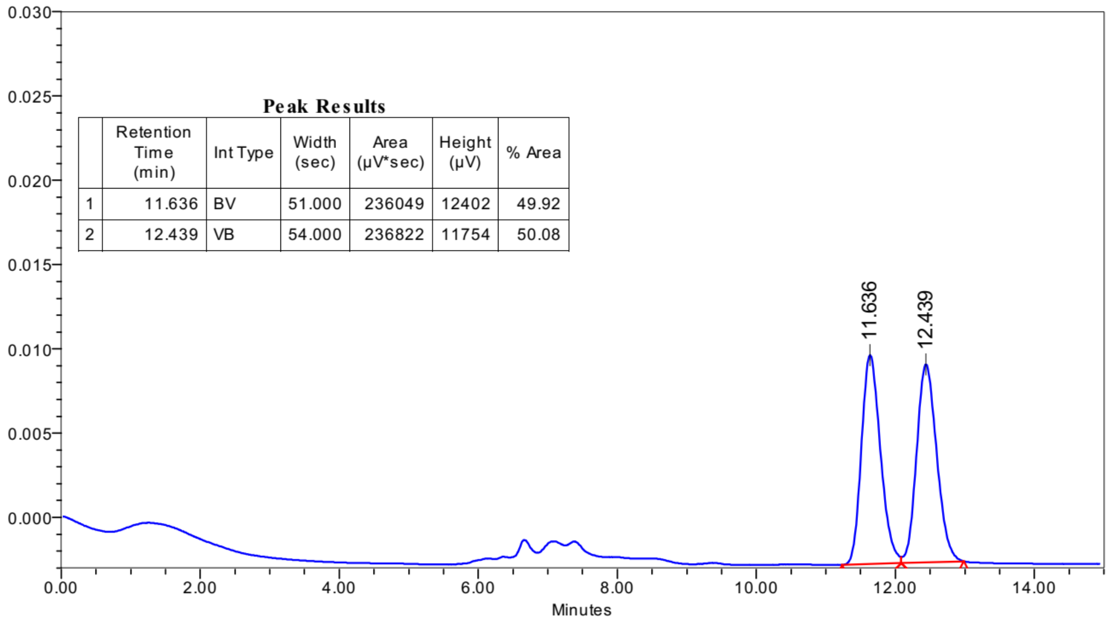

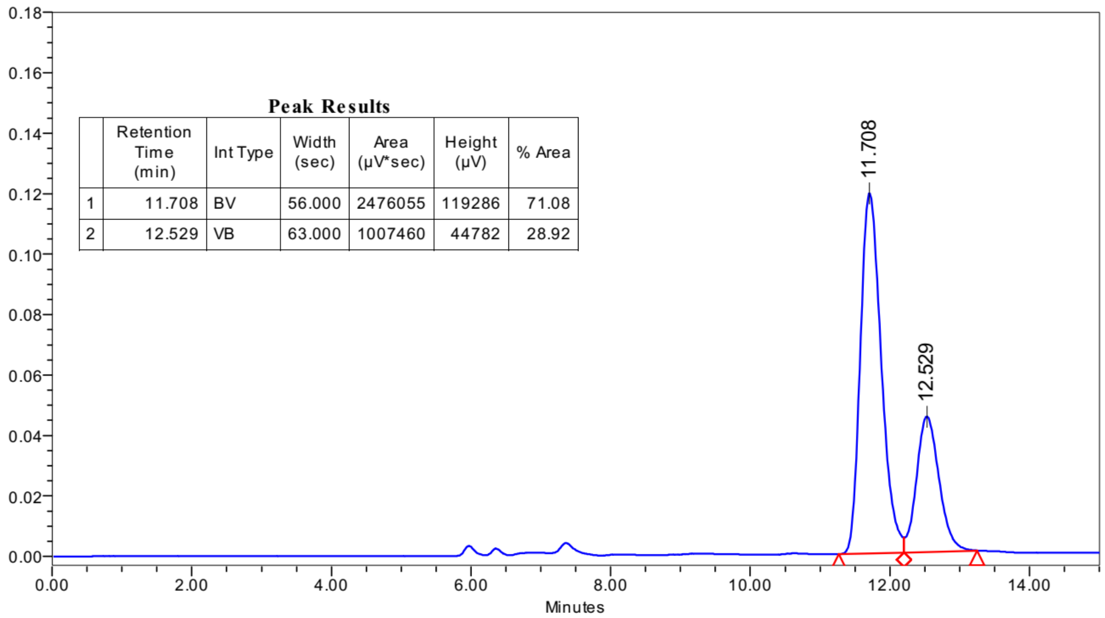

	Retention Time (min)	Int Type	Width (sec)	Area (µV*sec)	Height (µV)	% Area
1	11.708	BV	56.000	2476055	119286	71.08
2	12.529	VB	63.000	1007460	44782	28.92

1-benzhydryl-2-(tert-butyl)-5,6-dimethoxy-2,3-dihydro-1H-benzo[c][1,2]azaborole (**3al**)

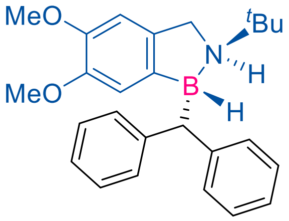

3al

Following the above procedure **F**, isolated yield: 40%, (32.1 mg), white solid (mp: 153.2 – 155.8 ºC), R_f = 0.4 (silica gel, hexane/DCM = 3:1, v/v), column chromatography (silica gel, hexane/DCM = 2:1, v/v).

^{1}H NMR (500 MHz, $CDCl_3$) δ 7.30 (d, *J* = 7.2 Hz, 2H), 7.26 – 7.17 (m, 4H), 7.13 – 7.04 (m, 2H), 7.03 (dd, *J* = 5.1, 3.2 Hz, 2H), 6.52 (s, 1H), 5.81 (s, 1H), 4.03 (dd, *J* = 15.0, 4.3 Hz, 1H), 3.89 (dd, *J* = 15.0, 8.0 Hz, 1H), 3.80 (s, 3H), 3.56 (d, *J* = 6.3 Hz, 1H), 3.49 (s, 1H), 3.48 (s, 3H), 1.16 (s, 9H).

^{13}C NMR (126 MHz, $CDCl_3$) δ 147.9, 147.6, 147.3, 145.6, 130.5, 130.2, 128.2, 127.9, 127.7, 124.8, 123.8, 111.9, 103.7, 57.7, 55.7, 55.1, 52.4, 26.6.

^{11}B NMR (160 MHz, $CDCl_3$) δ -1.7.

HRMS (ESI) m/z: $[M+Cl]^-$ Calcd. for $C_{26}H_{32}BClNO_2$ 436.222; Found: 436.2231.

HPLC analysis: DAICEL CHIRALCEL IG, hexane/isopropanol = 90/10, 0.5 mL/min, λ = 267 nm, t_1 = 17.098 min, t_2 = 20.892 min, 90% ee.

$[\alpha]^{25}_D$: +4.55 (*c* 0.33, $CHCl_3$).

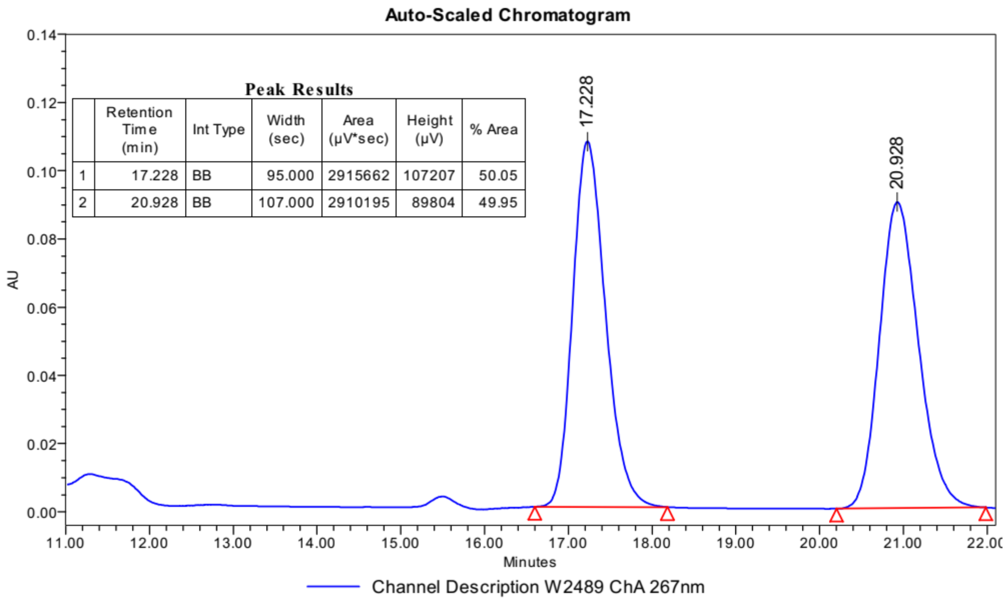

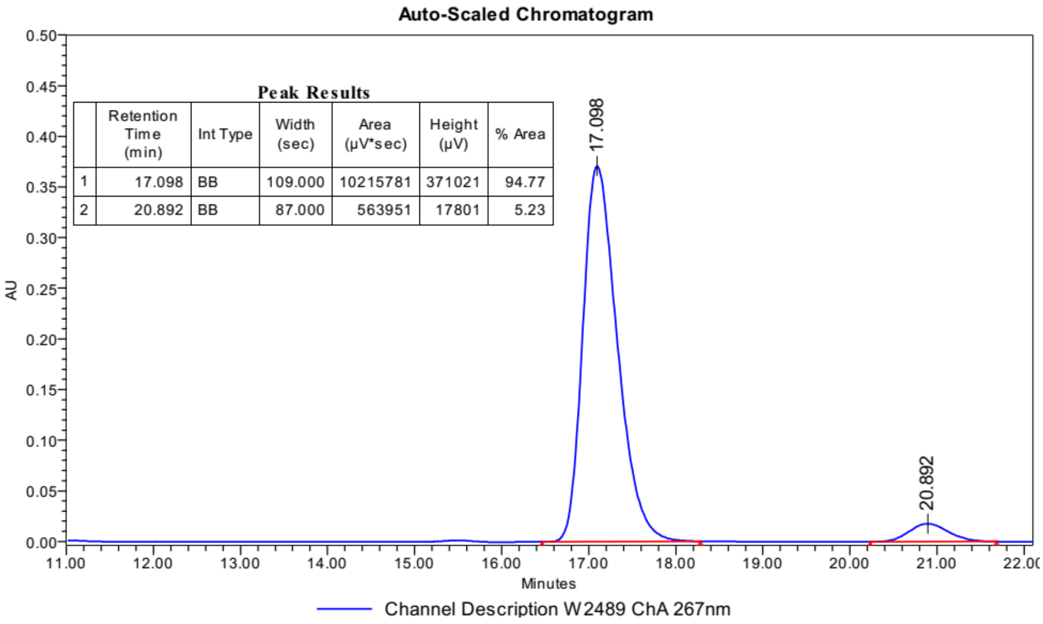

1-benzhydryl-2-(tert-butyl)-6-methoxy-2,3-dihydro-1H-benzo[c][1,2]azaborole (**3am**)

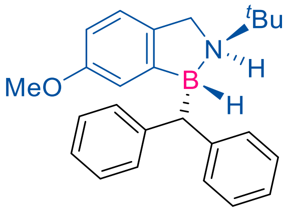

3am

Following the above procedure **F**, isolated yield: 40%, (29.7 mg), white solid (mp: 69.3 – 71.5 °C), R_f = 0.4 (silica gel, hexane/DCM = 4:1, v/v), column chromatography (silica gel, hexane/DCM = 3:1, v/v). **^{1}H NMR** (500 MHz, Acetone-d_6) δ 7.43 (d, J = 7.9 Hz, 2H), 7.28 – 7.20 (m, 6H), 7.12 – 7.08 (m, 2H), 6.95 (d, J = 8.2 Hz, 1H), 6.56 (dd, J = 8.2, 2.4 Hz, 1H), 6.09 (s, 1H), 4.36 – 4.26 (m, 2H), 4.11 (s, 1H),

3.49 (s, 3H), 3.41 (d, J = 7.0 Hz, 1H), 1.14 (s, 9H).

^{13}C NMR (126 MHz, Acetone-d_6) δ 158.7, 149.7, 147.6, 133.2, 130.7, 128.7, 128.4, 128.2, 125.0, 124.5, 121.4, 114.3, 112.1, 58.2, 54.5, 52.2, 26.2.

^{11}B NMR (128 MHz, Acetone-d_6) δ -0.63 (d, J = 78.2 Hz).

HRMS (ESI) m/z: $[M+H]^+$ Calcd. for $C_{25}H_{31}BNO$ 372.2493; Found: 372.2502.

HPLC analysis: DAICEL CHIRALCEL IG, hexane/isopropanol = 98/2, 0.5 mL/min, λ = 267 nm, t_1 = 13.541 min, t_2 = 15.765 min, 87% ee.

$[\alpha]^{25}_D$: +43.90 (c 0.41, $CHCl_3$).

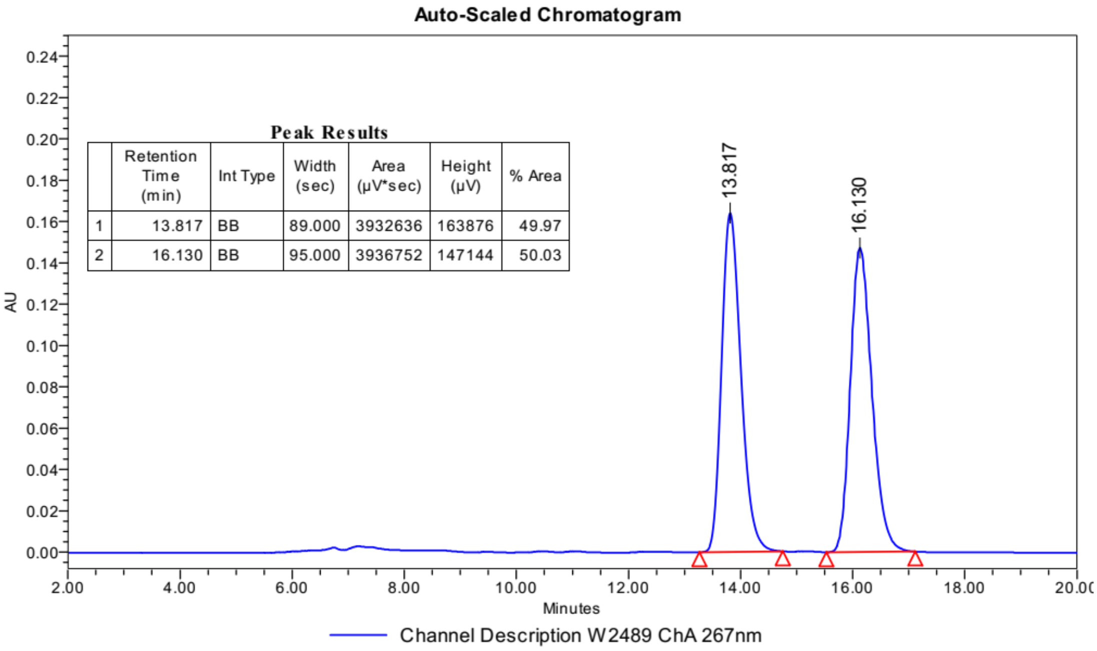

Peak Results

	Retention Time (min)	Int Type	Width (sec)	Area (μV*sec)	Height (μV)	% Area
1	13.817	BB	89.000	3932636	163876	49.97
2	16.130	BB	95.000	3936752	147144	50.03

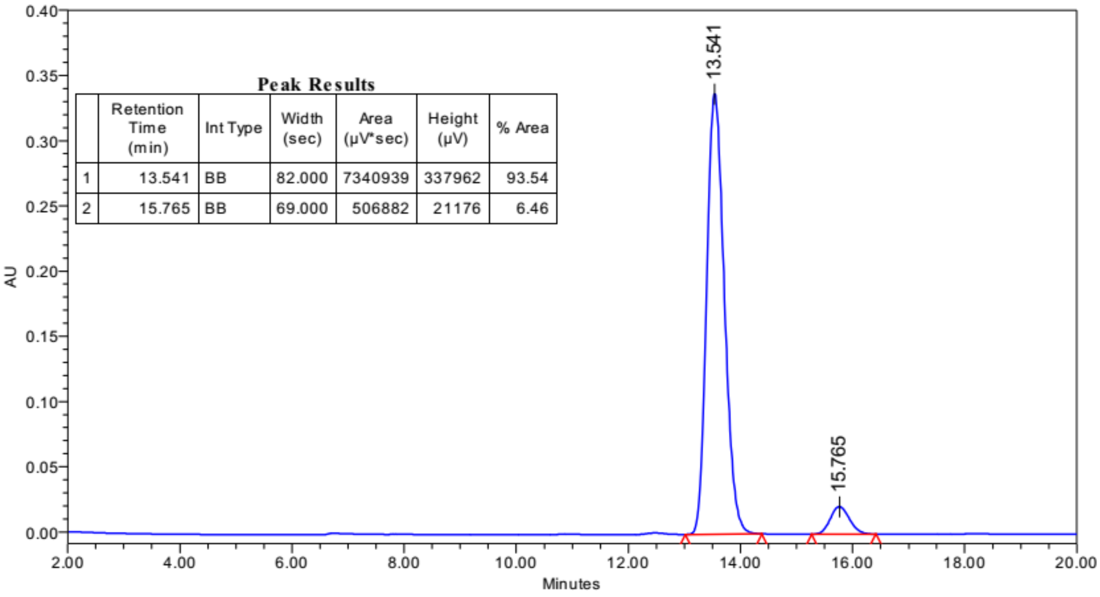

Peak Results

	Retention Time (min)	Int Type	Width (sec)	Area (μV*sec)	Height (μV)	% Area
1	13.541	BB	82.000	7340939	337962	93.54
2	15.765	BB	69.000	506882	21176	6.46

1-benzhydryl-2-(tert-butyl)-5-fluoro-2,3-dihydro-1H-benzo[c][1,2]azaborole (**3an**)

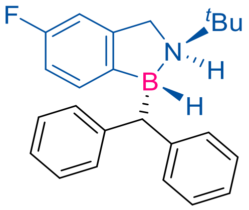

3an

Following the above procedure **F**, isolated yield: 45%, (32.3 mg), white solid (mp: 129.8 – 132.4 ºC), R_f = 0.4 (silica gel, hexane/DCM = 5:1, v/v), column chromatography (silica gel, hexane/DCM = 3:1, v/v).

^{1}H NMR (500 MHz, $CDCl_3$) δ 7.33 – 7.30 (m, 2H), 7.28 – 7.19 (m, 4H), 7.15 – 7.07 (m, 4H), 6.68 (dd, *J* = 9.5, 2.1 Hz, 1H), 6.66 – 6.61 (m, 1H), 6.46 (dd, *J* = 8.0, 6.3 Hz, 1H), 4.06 (dd, *J* = 15.7, 3.4 Hz, 1H), 3.96 (dd, *J* = 15.7, 8.0 Hz, 1H), 3.55 (s, 1H), 3.42 (d, *J* = 6.8 Hz, 1H), 1.11 (s, 9H).

^{13}C NMR (126 MHz, $CDCl_3$) δ 161.9 (d, *J* = 241.2 Hz), 148.2, 145.8, 141.0 (d, *J* = 7.3 Hz), 130.8 (d, *J* = 7.6 Hz), 130.1, 128.14 (d, *J* = 3.7 Hz), 127.7, 124.9, 124.2, 113.3 (d, *J* = 19.8 Hz), 107.1 (d, *J* = 21.42 Hz), 57.9, 51.7 (d, *J* = 3.7 Hz), 26.4.

^{19}F NMR (471 MHz, $CDCl_3$) δ -119.0.

^{11}B NMR (160 MHz, $CDCl_3$) δ -2.1.

HRMS (ESI) m/z: $[M+H]^+$ Calcd. for $C_{24}H_{27}BFN$ 360.2294; Found: 360.2303.

HPLC analysis: DAICEL CHIRALCEL AS-H, hexane/isopropanol = 98/2, 0.5 mL/min, λ = 267 nm, t_1 = 10.588 min, t_2 = 12.675 min, 77% ee.

$[\alpha]^{25}_D$: +0.33 (*c* 0.3, $CHCl_3$).

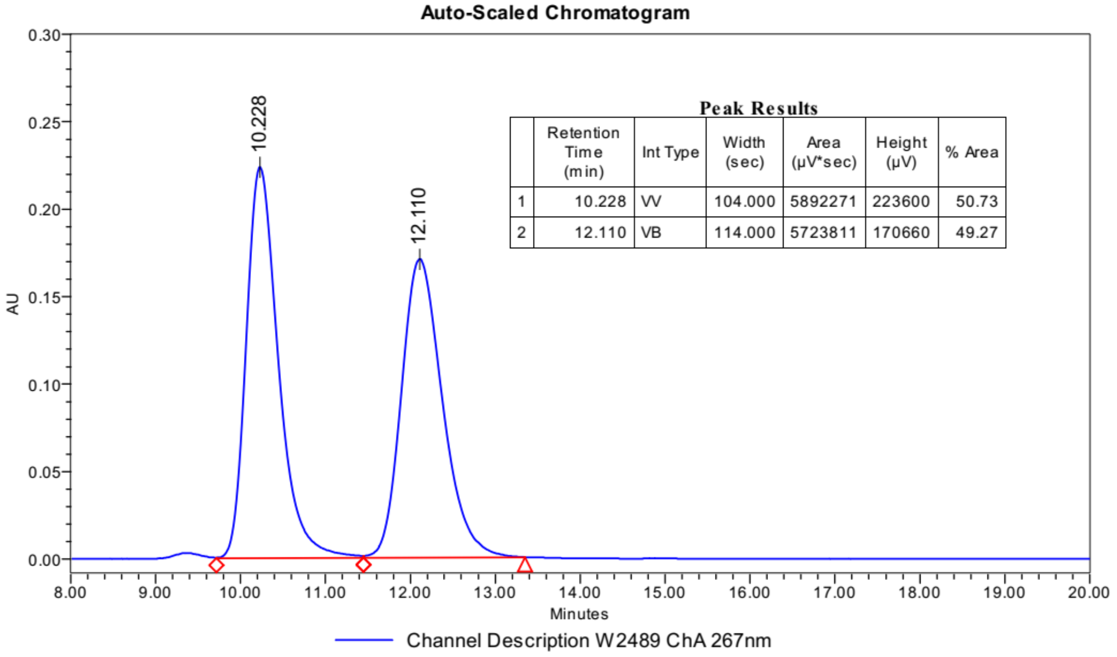

Peak Results

	Retention Time (min)	Int Type	Width (sec)	Area (µV*sec)	Height (µV)	% Area
1	10.228	VV	104.000	5892271	223600	50.73
2	12.110	VB	114.000	5723811	170660	49.27

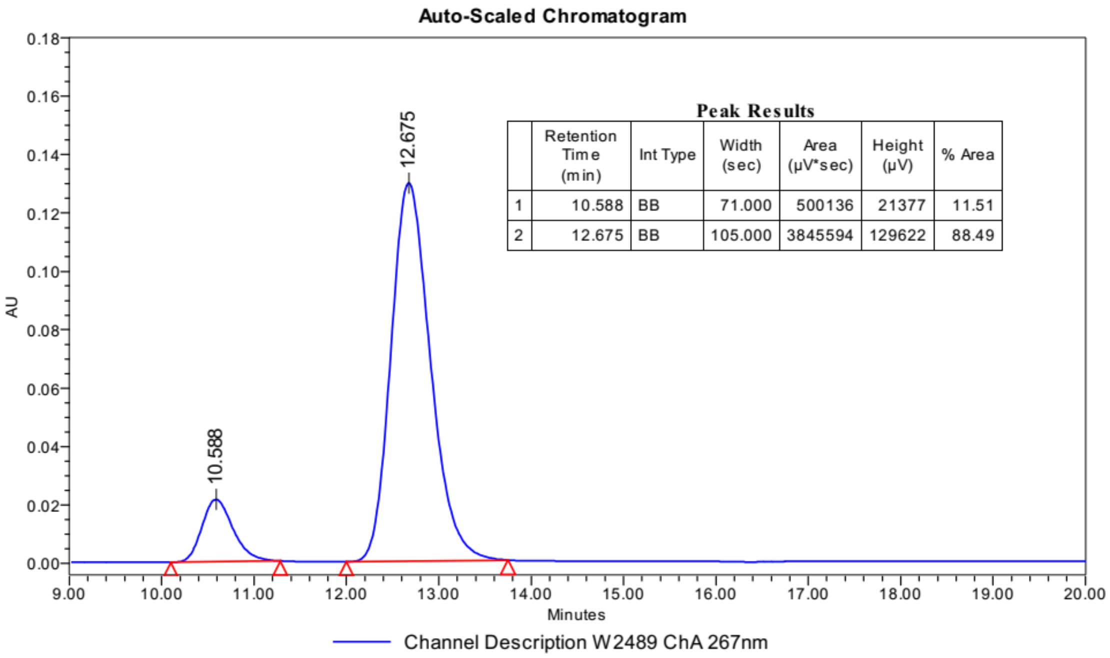

	Retention Time (min)	Int Type	Width (sec)	Area (μV*sec)	Height (μV)	% Area
1	10.588	BB	71.000	500136	21377	11.51
2	12.675	BB	105.000	3845594	129622	88.49

1-benzhydryl-2-benzyl-2-methyl-2,3-dihydro-1H-2l4-benzo[c][1,2]azaborole (3ao)

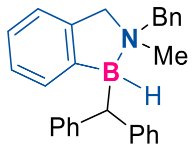

3ao

Following the above procedure **E**, isolated yield: 70%, (27.2 mg), colorless oil, R_f = 0.5 (silica gel, hexane/DCM = 5:1, v/v), column chromatography (silica gel, hexane/DCM = 3:1, v/v).

^{1}H NMR (500 MHz, $CDCl_3$) δ 7.61 (d, *J* = 7.1 Hz, 2H), 7.55 (d, *J* = 7.1 Hz, 2H), 7.40 – 7.34 (m, 3H), 7.32 – 7.26 (m, 4H), 7.18 – 7.08 (m, 4H), 7.03 (dd, *J* = 7.7, 1.3 Hz, 2H), 7.01 – 6.96 (m, 1H), 6.38 (d, *J* = 7.2 Hz, 1H), 3.99 (d, *J* = 13.4 Hz, 1H), 3.87 (dd, *J* = 13.4, 9.3 Hz, 2H), 3.67 (d, *J* = 13.5 Hz, 1H), 3.59 (d, *J* = 8.0 Hz, 1H), 2.19 (s, 3H).

^{13}C NMR (126 MHz, $CDCl_3$) δ 149.1, 148.2, 138.5, 132.1, 130.8, 130.5, 129.7, 129.0, 128.5, 128.3, 128.1, 127.0, 125.4, 124.8, 124.6, 121.3, 62.7, 56.5, 46.4.

^{11}B NMR (160 MHz, $CDCl_3$) δ 4.3.

HRMS (ESI) m/z: $[M+K]^+$ Calcd. for $C_{28}H_{28}BNK$ 428.1946; Found: 428.1940.

HPLC analysis: DAICEL CHIRALCEL ID-3, hexane/isopropanol = 98/2, 1.0 mL/min, λ = 267 nm, t_1 = 6.226 min, t_2 = 6.786 min, 4% ee.

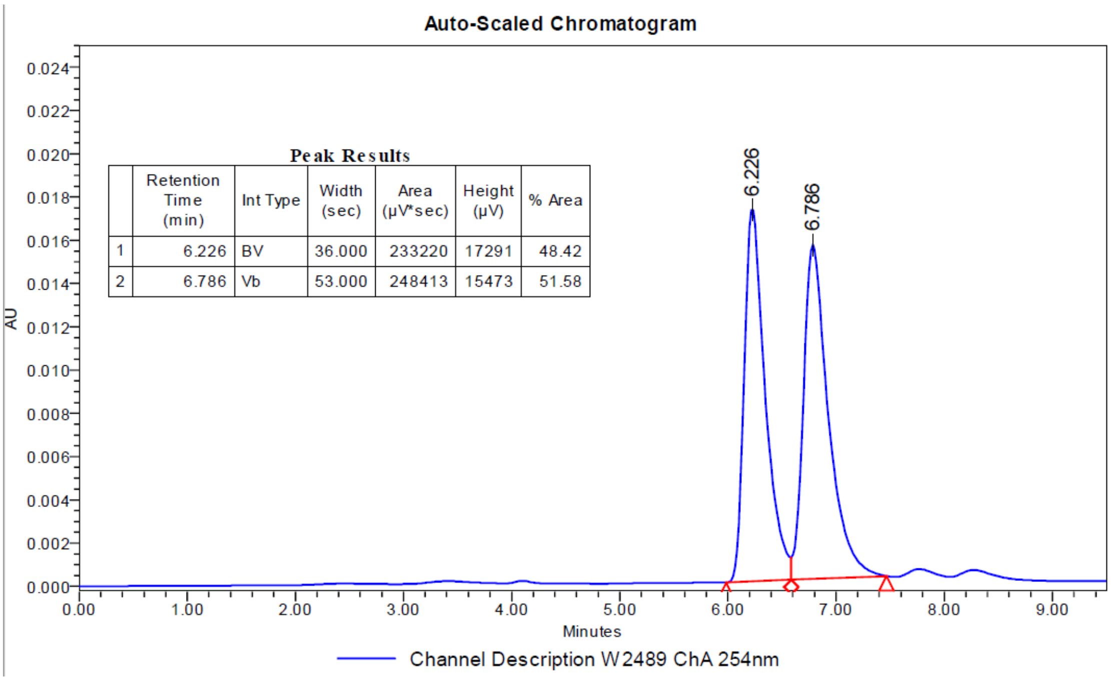

Peak Results

	Retention Time (min)	Int Type	Width (sec)	Area (µV*sec)	Height (µV)	% Area
1	6.226	BV	36.000	233220	17291	48.42
2	6.786	Vb	53.000	248413	15473	51.58

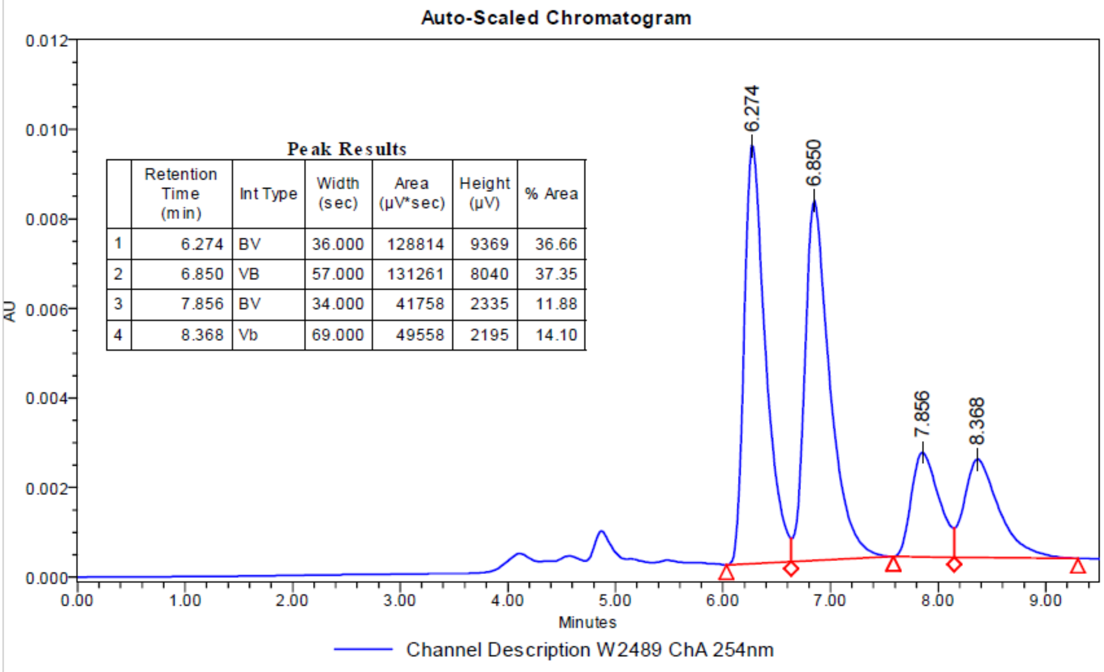

Peak Results

	Retention Time (min)	Int Type	Width (sec)	Area (µV*sec)	Height (µV)	% Area
1	6.274	BV	36.000	128814	9369	36.66
2	6.850	VB	57.000	131261	8040	37.35
3	7.856	BV	34.000	41758	2335	11.88
4	8.368	Vb	69.000	49558	2195	14.10

1-(5-((2-(tert-butyl)-2,3-dihydro-1H-benzo[c][1,2]azaborol-1-yl)(phenyl)methyl)-2-methylfuran-3-yl)ethan-1-ol (3ap)

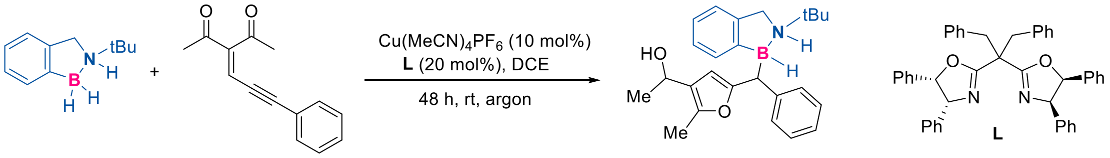

3ap, 31% yield, dr = 1.12:1

In air, a 25 mL schlenk tube was charged with **1a** (0.3 mmol, 3 equiv), ene-yne-ketone (0.1 mmol, 1

equiv), $Cu(MeCN)_4PF_6$ (10 mol%), **L** (20 mol%). The tube was evacuated and filled with argon for three cycles. Then, 1 mL of 1,2-dichloroethane was added under argon. The reaction was allowed to stir at rt for 48 hours. Upon completion, proper amount of silica gel was added to the reaction mixture. After removal of the solvent, the crude reaction mixture was purified on silica gel to afford the desired product **3ap**. Isolated yield: 31%, (12.0 mg), colorless oil, dr = 1.12:1, R_f = 0.4 (silica gel, PE/acetone = 5:1, v/v).

^{1}H NMR (500 MHz, $CDCl_3$) δ 7.23 – 7.19 (m, 2H), 7.15 – 7.10 (m, 3H), 7.05 – 7.01 (m, 1H), 6.99 – 6.96 (m, 1H), 6.95 – 6.90 (m, 1H), 6.54 – 6.47 (m, 1H), 5.81 (s, 1H), 4.79 – 4.72 (m, 1H), 4.34 – 4.24 (m, 1H), 4.21 – 6.90 (m, 2H), 3.23 – 3.15 (m, 2H), 2.29 (s, 0.53*3H), 2.29 (s, 0.47*3H), 1.39 (d, J = 6.4 Hz, 0.47*3H), 1.37 (d, J = 6.4 Hz, 0.53*3H), 1.14 (s, 0.53*3H), 1.14 (s, 0.47*3H).

^{13}C NMR (126 MHz, $CDCl_3$) δ 160.8, 160.7, 144.4, 144.2, 144.1, 144.1, 139.5, 129.7, 129.7, 129.3, 129.2, 127.9, 127.8, 126.1, 125.2, 124.6, 124.2, 120.0, 101.7, 101.5, 62.9, 57.9, 52.2, 52.2, 26.3, 23.9, 11.9.

^{11}B NMR (160 MHz, $CDCl_3$) δ -1.39.

HRMS (ESI) m/z: $[M+Na]^+$ Calcd. for $C_{25}H_{32}BNO_2Na$ 412.2418; Found: 412.2408.

3 Mechanistic studies

A The enantioselectivities of **3a** and **1a-1** vs a function of time

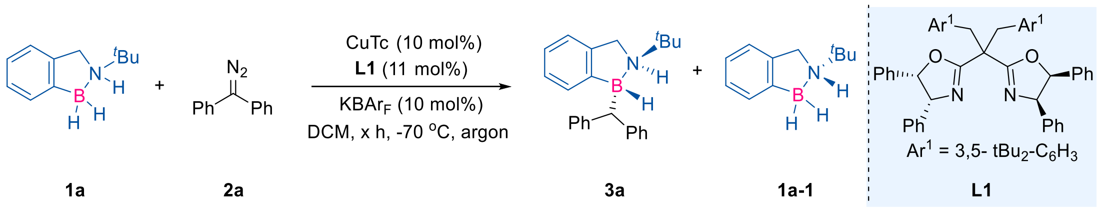

In air, a 25 mL schlenk tube was charged with **1a** (0.2 mmol, 2 equiv), **2a** (0.1 mmol, 1 equiv), CuTc (10 mol%), **L1** (11 mol%), additive (10 mol%). The tube was evacuated and filled with argon for three cycles. Then, 1 mL of dichloromethane was added under argon. The reaction was allowed to stir at -70 °C. Diazo compound(**2a**) is very reactive, sampling in the middle of the reaction leads to inaccurate results. This reaction was balanced 10 times, quenched one reaction at every interval time. The reaction was quenched with 3 M of HCl, and immediately separated.

Time/h	3a	1a-1
3	90	26
9	90	31
14	90	35
19	90	42
24	90	56

29	91	69
34	90	72
39	91	85
42	90	90
48	91	90

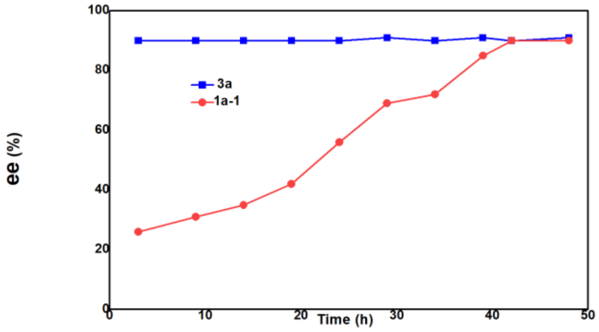

Figure S1. The enantioselectivities of **3a** and **1a-1** vs a function of time

B The effect of the ratio of **1a** to **2a** on the reaction

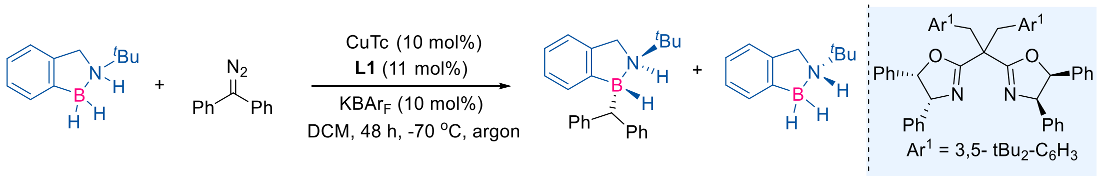

1a (1 quive) **2a** (x quive) **3a** **1a-1** **L1**

In air, a 25 mL schlenk tube was charged with **1a** (0.2 mmol, 1 equiv), **2a** (x mmol, x equiv), CuTc (10 mol%), **L1** (11 mol%), additive (10 mol%). The tube was evacuated and filled with argon for three cycles. Then, 1 mL of dichloromethane was added under argon. The reaction was allowed to stir at -70 °C for 48 h. Upon completion, proper amount of silica gel was added to the reaction mixture. After removal of the solvent, the crude reaction mixture was purified on silica gel (petroleum ether and ethyl acetate) to afford the desired products.

Table S6. The effect of the ratio of 1a to 2a on the reaction

entry	**1a**:**2a**	**3a** ee (%)	**3a** Yield (%)	**1a-1** ee (%)	**1a-1** Yield (%)
1	1:0.6	69	60	94	32
2	1:0.55	83	49	92	47
3	1:0.5	90	46	90	49
4	1:0.47	90	45	81	54
5	1:0.45	91	40	73	53

Isolated yields.

C The deuterium-labeling experiment

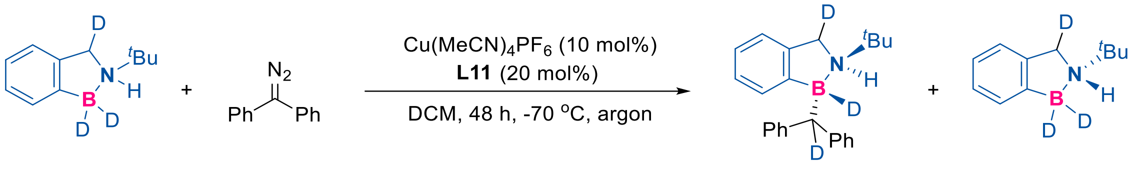

In air, a 25 mL schlenk tube was charged with **1a-*d*₃** (0.2 mmol, 2 equiv), **2a** (0.1 mmol, 1 equiv), $Cu(MeCN)_4PF_6$ (10 mol%), **L1** (20 mol%). The tube was evacuated and filled with argon for three cycles. Then, 1 mL of Dichloromethane was added under argon. The reaction was allowed to stir at -70 °C for 48 hours. Upon completion, proper amount of silica gel was added to the reaction mixture. After removal of the solvent, the crude reaction mixture was purified on silica gel (petroleum ether and ethyl acetate) to afford the desired products, isolated yield: 32%, (11.2 mg), white solid (mp: 109.8 – 112.4 °C), R_f = 0.4 (silica gel, hexane/DCM = 4:1, v/v), column chromatography (silica gel, hexane/DCM = 3:1, v/v).

^{1}H NMR (500 MHz, $CDCl_3$) δ 7.36 – 7.32 (m, 2H), 7.29 – 7.26(m, 2H), 7.24 – 7.21 (m, 2H), 7.17 – 7.10 (m, 4H), 7.08 – 7.03 (m, 1H), 7.00 (d, *J* = 7.4 Hz, 1H), 6.95 (t, *J* = 7.2 Hz, 1H), 6.61 – 6.56 (m, 1H), 4.15 – 4.01 (m, 1H), 3.50 (s, 1H), 1.13 (s, 9H).

^{13}C NMR (126 MHz, $CDCl_3$) δ 148.4, 145.9, 139.5, 130.2, 129.8, 128.1, 128.0, 127.7, 126.2, 125.1, 124.8, 124.1, 120.1, 120.1, 57.7, 26.5.

^{11}B NMR (160 MHz, $CDCl_3$) δ -1.7.

HRMS (ESI) m/z: $[M+H]^+$ Calcd. for $C_{24}H_{26}D_3BN$ 345.2576; Found: 345.2579.

HPLC analysis: DAICEL CHIRALCEL IG, hexane/isopropanol = 98/2, 0.5 mL/min, λ = 267 nm, t_1 = 9.732 min, t_2 = 10.773 min, 91% ee.

$[\alpha]^{25}_D$: -22.0 (*c* 0.3, $CHCl_3$).

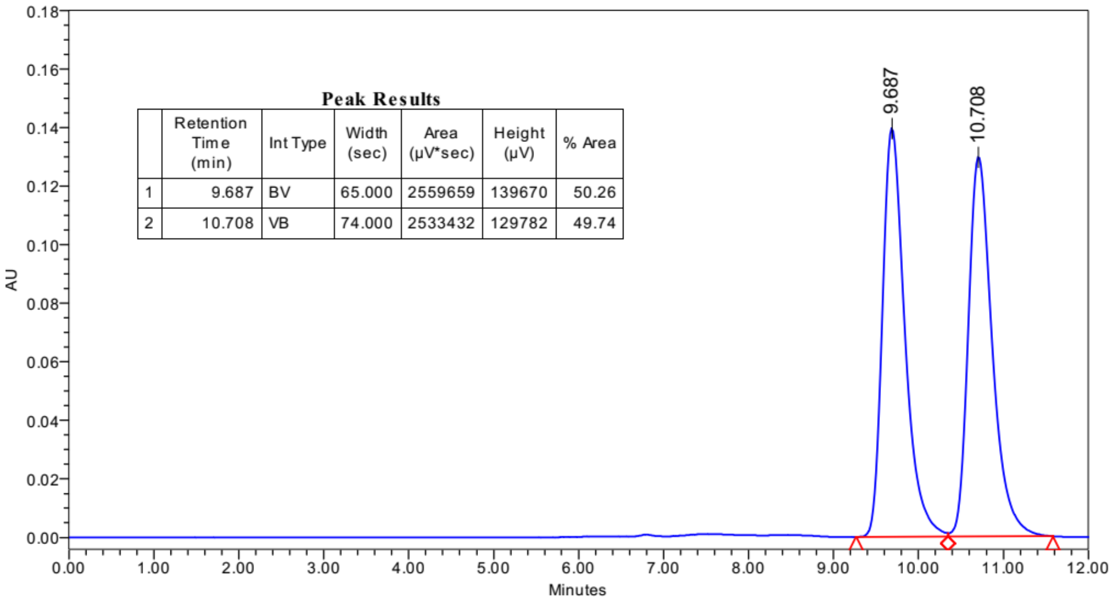

	Retention Time (min)	Int Type	Width (sec)	Area (μV*sec)	Height (μV)	% Area
1	9.687	BV	65.000	2559659	139670	50.26
2	10.708	VB	74.000	2533432	129782	49.74

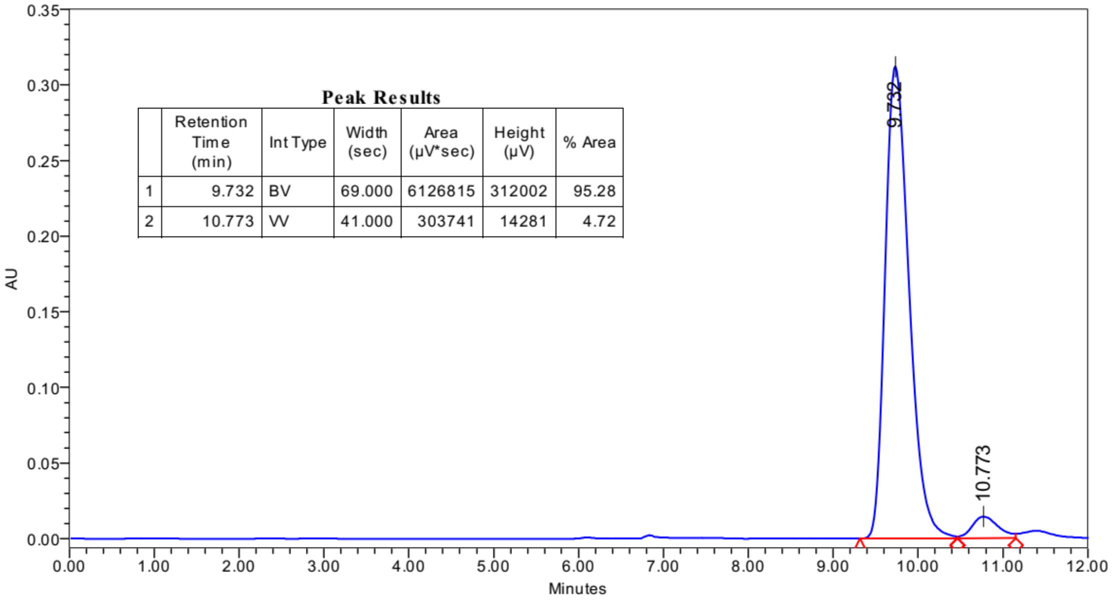

	Retention Time (min)	Int Type	Width (sec)	Area (μV*sec)	Height (μV)	% Area
1	9.732	BV	69.000	6126815	312002	95.28
2	10.773	VV	41.000	303741	14281	4.72

4 The application of the chiral amine borane

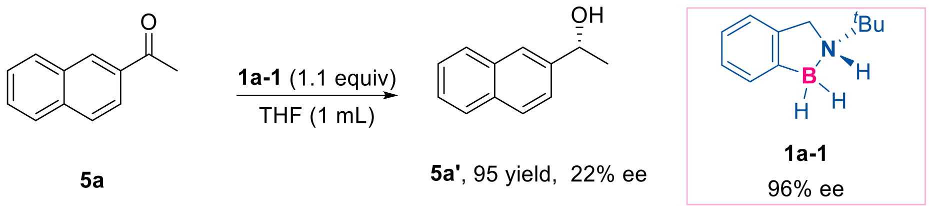

A Schlenk bottle was charged with chiral amine borane (**1a-1**) (19.3 mg, 0.11 mmol, 1.1 equiv), the 2-acetonaphthone (**5a**) (17 mg, 0.1 mmol, 1 equiv), and THF (1 mL) were added. The reaction was monitored by TLC using petroleum ether: ethyl acetate = 10: 1 as eluent. Upon completion, proper amount of silica gel was added to the reaction mixture. After removal of the solvent, the crude reaction

mixture was purified on silica gel (petroleum ether and ethyl acetate) to afford the desired product **5a**.

(*R*)-1-(naphthalen-2-yl)ethan-1-ol. **^{1}H NMR** (500 MHz, $CDCl_3$) δ 7.86 (dd, *J* = 8.3, 5.3 Hz, 3H), 7.81 (s, 1H), 7.55 – 7.48 (m, 3H), 5.05 (dd, *J* = 12.2, 6.0 Hz, 1H), 2.34 (dd, *J* = 51.0, 20.9 Hz, 1H), 1.60 (dd, *J* = 6.5, 1.0 Hz, 3H). **^{13}C NMR** (126 MHz, $CDCl_3$) δ 143.14 (d, *J* = 2.3 Hz), 133.2, 132.8, 128.2, 127.9, 127.6, 126.1, 125.7, 123.8, 123.7, 99.8, 25.1.[3]

HPLC analysis: DAICEL CHIRALCEL OJ-H, hexane/isopropanol = 80/20, 1 mL/min, λ = 254 nm, t_1 = 13.503 min, t_2 = 16.509 min, 22% ee.

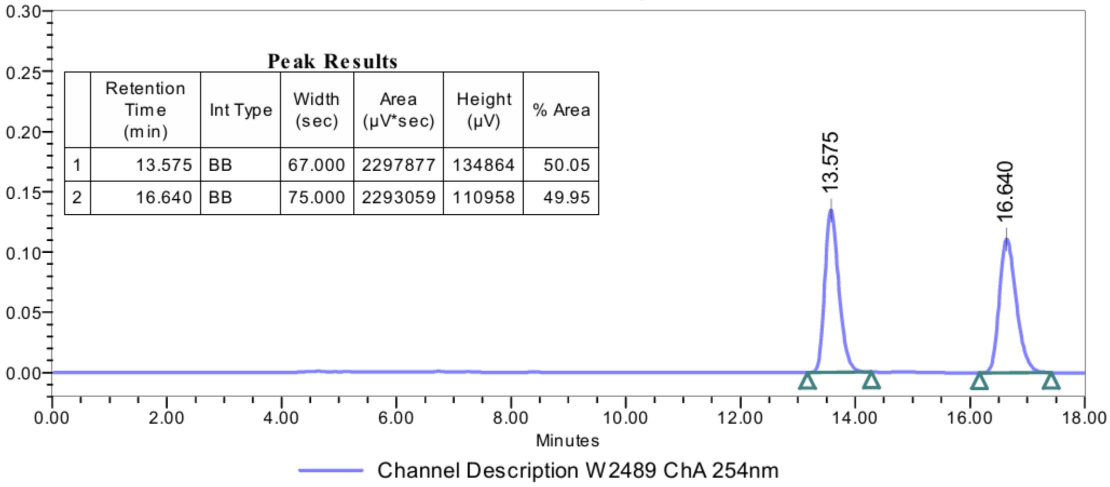

Peak Results

	Retention Time (min)	Int Type	Width (sec)	Area (μV*sec)	Height (μV)	% Area
1	13.575	BB	67.000	2297877	134864	50.05
2	16.640	BB	75.000	2293059	110958	49.95

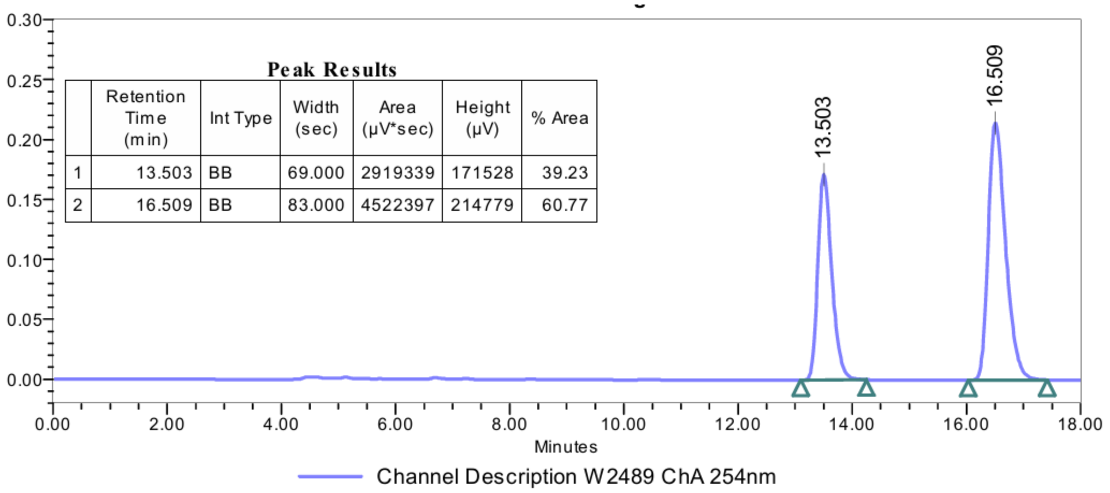

Peak Results

	Retention Time (min)	Int Type	Width (sec)	Area (μV*sec)	Height (μV)	% Area
1	13.503	BB	69.000	2919339	171528	39.23
2	16.509	BB	83.000	4522397	214779	60.77

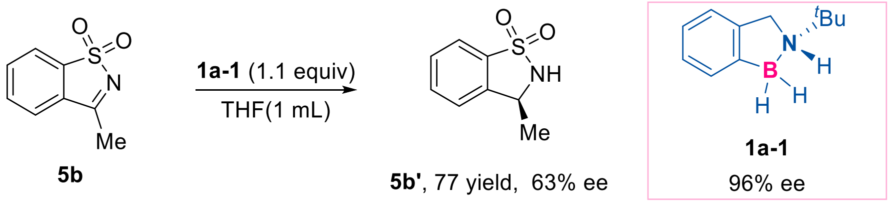

A Schlenk bottle was charged withchiral amine borane (**1a-1**) (19.3 mg, 0.11 mmol, 1.1 equiv), 3-methylbenzo[d]isothiazole 1,1-dioxide (**5b**) (18 mg, 0.1 mmol, 1 equiv), and THF (1 mL) were added. The reaction was monitored by TLC using petroleum ether: ethyl acetate = 5:1 as eluent. Upon completion, proper amount of silica gel was added to the reaction mixture. After removal of the solvent,

the crude reaction mixture was purified on silica gel (petroleum ether and ethyl acetate) to afford the desired product **5b**. **(*S*)-3-methyl-2,3-dihydrobenzo[d]isothiazole 1,1-dioxide**. **^{1}H NMR** (500 MHz, $CDCl_3$) δ 7.76 – 7.73 (m, 1H), 7.63 – 7.60 (m, 1H), 7.53 – 7.48 (m, 1H), 7.38 (dd, *J* = 7.8, 0.7 Hz, 1H), 5.06 (s, 1H), 4.78 (q, *J* = 6.7 Hz, 1H), 1.59 (d, *J* = 6.7 Hz, 3H).[4]

HPLC analysis: DAICEL CHIRALCEL OD-H, hexane/isopropanol = 80/20, 0.8 mL/min, λ = 210 nm, t_1 = 22.433 min, t_2 = 28.618 min, 63% ee.

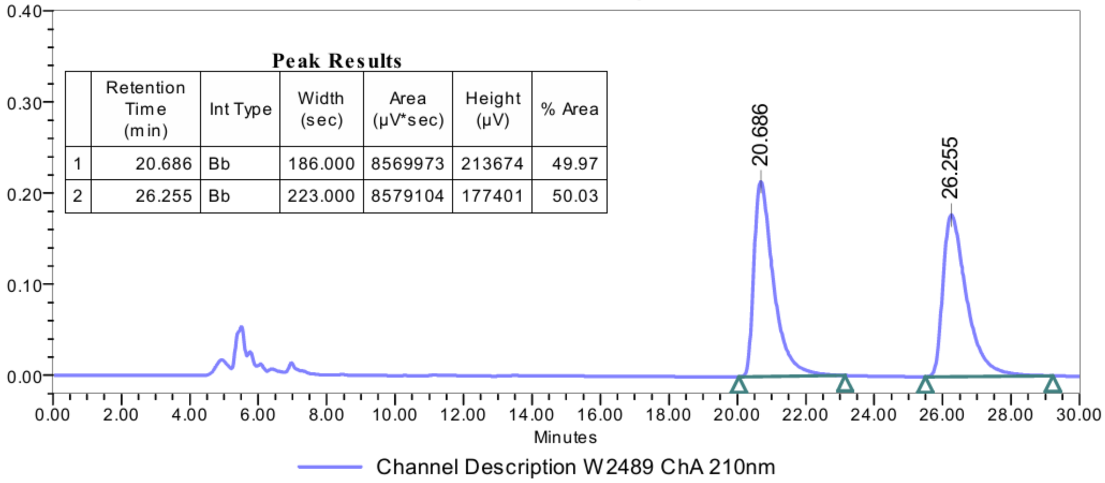

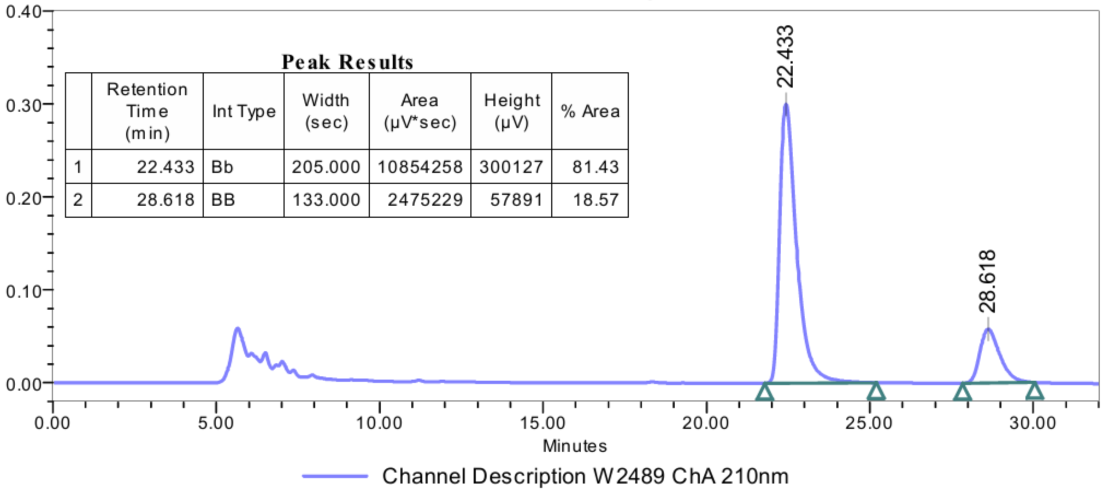

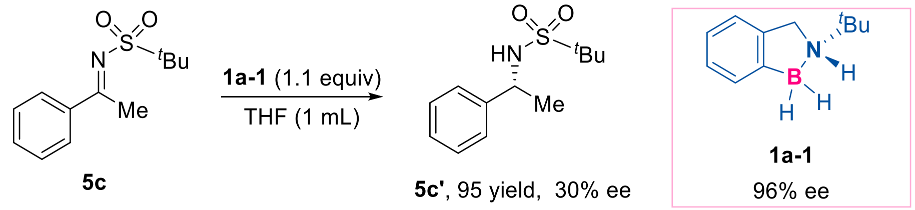

A Schlenk bottle was charged withchiral amine borane (**1a-1**) (19.3 mg, 0.11 mmol, 1.1 equiv), (*E*)-2-methyl-N-(1-phenylethylidene)propane-2-sulfonamide (**5c**) (24 mg, 0.1 mmol, 1 equiv), and THF (1 mL) were added. The reaction was monitored by TLC using petroleum ether: ethyl acetate = 10: 1 as eluent. Upon completion, proper amount of silica gel was added to the reaction mixture. After removal of the solvent, the crude reaction mixture was purified on silica gel (petroleum ether and ethyl acetate) to afford

the desired products. **(*R*)-2-methyl-N-(1-phenylethyl)propane-2-sulfonamide**. **^{1}H NMR** (500 MHz, $CDCl_3$) δ 7.37 – 7.30 (m,4H), 7.29 – 7.24 (m, 1H), 4.73 (d, J = 8.9 Hz), 4.69 – 4.63 (m, 1H), 1.56 (d, J = 6.8 Hz, 3H), 1.31 (s, 9H). [4]

HPLC analysis: DAICEL CHIRALCEL OD-H, hexane/isopropanol = 95/5, 1 mL/min, λ = 210 nm, t_1 = 11.224 min, t_2 = 14.280 min, 30% ee.

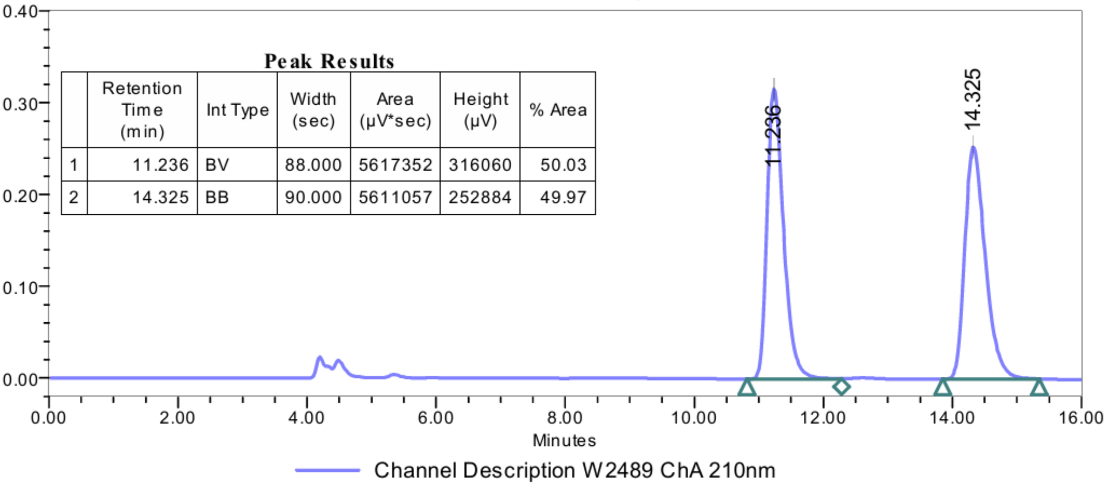

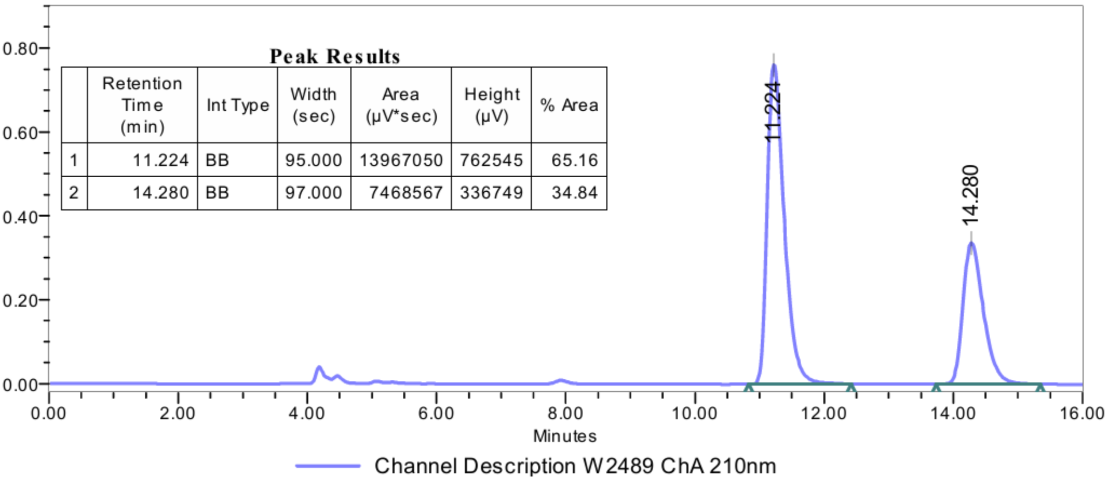

5 The synthetic tranformations of the amine borane product

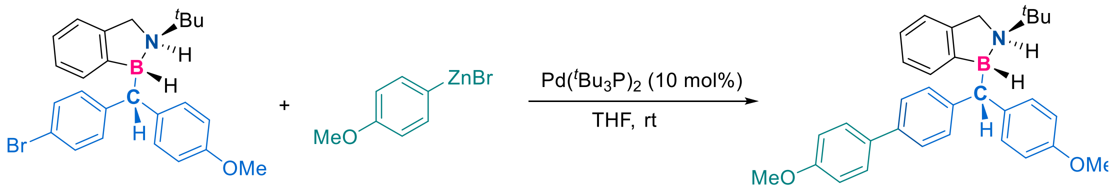

3o, 95% ee — **6**, 63% yield, 95% ee

In a glove box, a 25 mL Schlenk tube was charged with zinc chloride (1 M in THF, 0.2 mmol, 2.0 equiv,) and 4-methoxyphenylmagnesium bromide (1 M in THF, 0.15 mmol, 1.5 equiv). The mixture was stirred for 30 minutes. Then, the mixture was charged with **3o** (0.1 mmol, 1.0 equiv), bis(tri-tert-butylphosphine)palladium (0.01 mmol, 10 mol%), 1 mL of THF. The tube was removed from glove box, and stirred for 12 h at room temperature. Upon completion, the solvent was concentrated under reduced

pressure and rapidly purified by column chromatography to obtain product **6**.

White solid, mp: 64-65 °C, yield: 63% (30 mg), R_f= 0.4 (PE: DCM=2:1)

^{1}H NMR (500 MHz, $CDCl_3$) δ 7.55 (d, *J* = 8.8 Hz, 2H), 7.47 (d, *J* = 8.2 Hz, 2H), 7.33 (d, *J* = 8.2 Hz, 2H), 7.09 – 7.02 (m, 3H), 7.01 – 6.93 (m, 4H), 6.79 (d, *J* = 8.7 Hz, 2H), 6.63 (d, *J* = 7.3 Hz, 1H), 4.14 (dd, *J* = 15.4, 3.6 Hz, 1H), 4.05 (dd, *J* = 15.4, 7.9 Hz, 1H), 3.85 (s, 3H), 3.78 (s, 3H), 3.57 (d, *J* = 4.2 Hz, 1H), 3.49 (d, *J* = 6.4 Hz, 1H), 1.17 (s, 9H).

^{13}C NMR (126 MHz, $CDCl_3$) δ 158.7, 157.0, 147.4, 139.5, 137.8, 136.3, 134.0, 131.1, 129.9, 128.0, 127.7, 126.3, 125.1, 120.2, 114.1, 113.6, 57.7, 55.3, 55.1, 52.2, 26.6.

^{11}B NMR (160 MHz, $CDCl_3$) δ -0.77.

HRMS (ESI) m/z: $[M+Na]^+$ Calcd for $C_{32}H_{36}BNO_2Na$ 500.2731, found: 500.2723.

HPLC analysis: DALCEL CHIRALCEL ASH, hexane/isopropanol = 85/15, 10 mL/min, λ = 267nm, t_R(major) =10.893 min, t_R(minor) = 8.470 min, 95% ee. **$[\alpha]^{25}_D$**: -52.000 (*c* 0.3, $CHCl_3$)

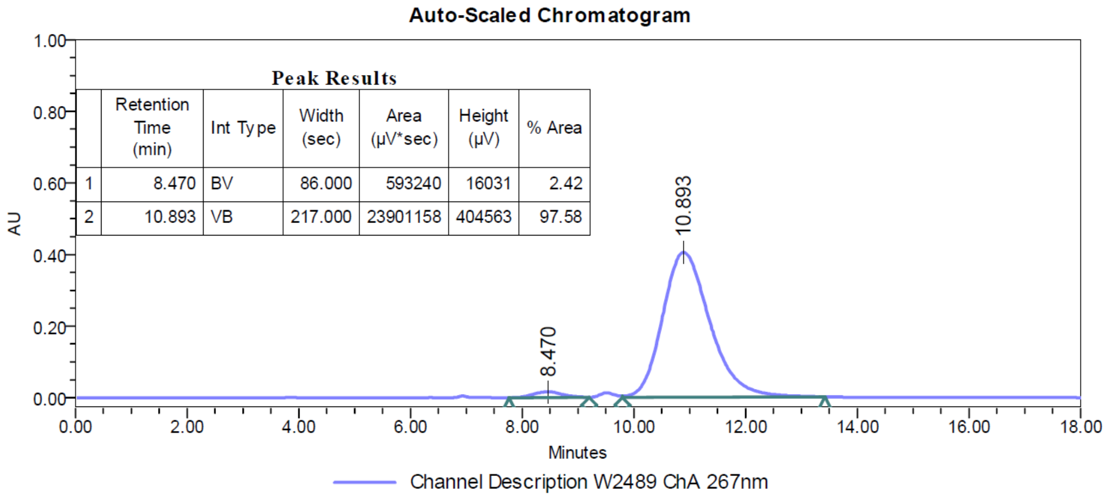

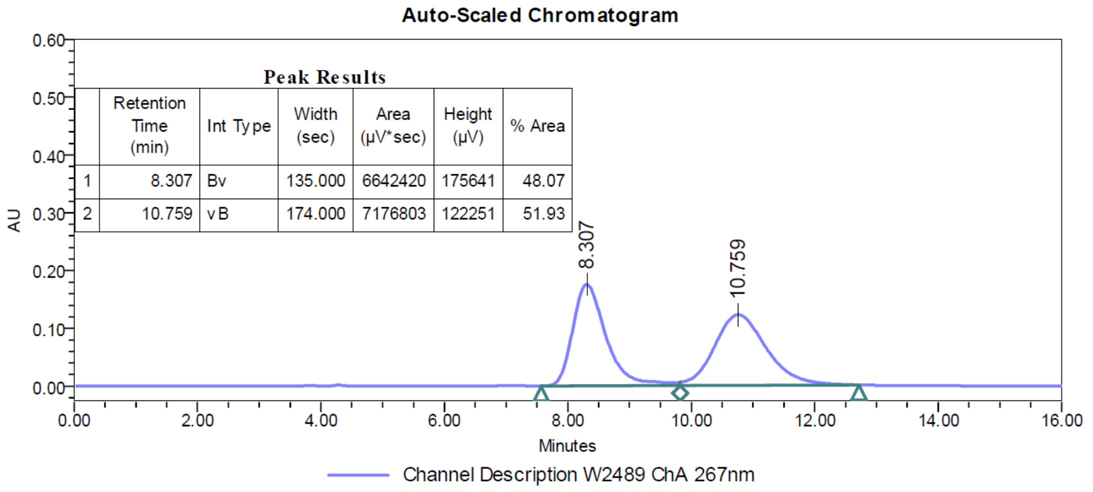

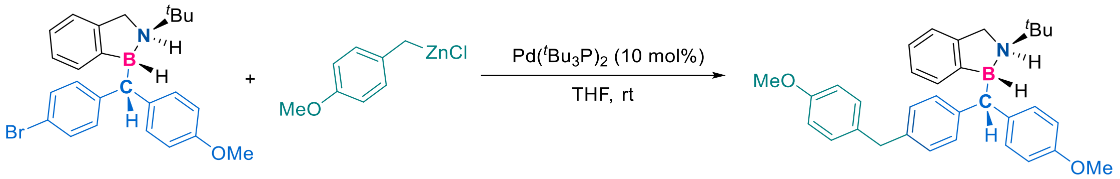

3o, 95% ee　　　　**7**, 87% yield, 96% ee

In a glove box, a 25 mL Schlenk tube was charged with zinc chloride (1 M in THF, 0.3 mmol, 3.0 equiv,) and 4-methoxybenzylmagnesium chloride (0.25 M in THF, 0.3 mmol, 3.0 equiv). The mixture was stirred for 30 minutes. Then, the mixture was charged with **3o** (0.1 mmol, 1.0 equiv), bis(tri-tert-butylphosphine)palladium (0.01 mmol, 10 mol%), 1 mL of THF. The tube was removed from glove box, and stirred for 12 h at room temperature. Upon completion, the solvent was concentrated under reduced pressure and rapidly purified by column chromatography to obtain product **7**.

White solid, 137 -138 ºC, yield: 87% (42.9 mg), R_f = 0.5 (PE: EA=5:1).

^{1}H NMR (500 MHz, $CDCl_3$) δ 7.19 (d, *J* = 8.0 Hz, 2H), 7.10 (d, *J* = 8.7 Hz, 2H), 7.07 – 7.02 (m, 5H), 6.98 (d, *J* = 7.5 Hz, 1H), 6.93 (t, *J* = 7.2 Hz, 1H), 6.81 (d, *J* = 8.7 Hz, 2H), 6.76 (d, *J* = 8.7 Hz, 2H), 6.58 (d, *J* = 7.2 Hz, 1H), 4.11 (dd, *J* = 15.4, 3.6 Hz, 1H), 4.08 – 4.01 (m, 1H), 3.89 (s, 2H), 3.78 (s, 3H), 3.76 (s, 3H), 3.53 (d, *J* = 3.9 Hz, 1H), 3.37 (d, *J* = 6.7 Hz, 1H), 1.11 (s, 9H).

^{13}C NMR (126 MHz, $CDCl_3$) δ 157.7, 156.9, 146.5, 139.5, 138.0, 136.9, 134.0, 131.0, 129.8, 129.7, 128.6, 127.6, 126.2, 125.0, 120.1, 113.7, 113.5, 57.6, 55.2, 55.1, 52.1, 40.5, 26.5.

HRMS (ESI) m/z: $[M+Na]^+$calcd for $C_{33}H_{38}BNO_2Na$ 514.2888, found: 514.2886.

HPLC analysis: DALCEL CHIRALCEL ASH, hexane/isopropanol = 95/05, 10 mL/min, λ = 267 nm, t_R(major) =17.395 min, t_R(minor) = 10.784 min, 96% ee.

$[\alpha]^{25}_D$: -3.000 (*c* 0.3, $CHCl_3$).

Auto-Scaled Chromatogram

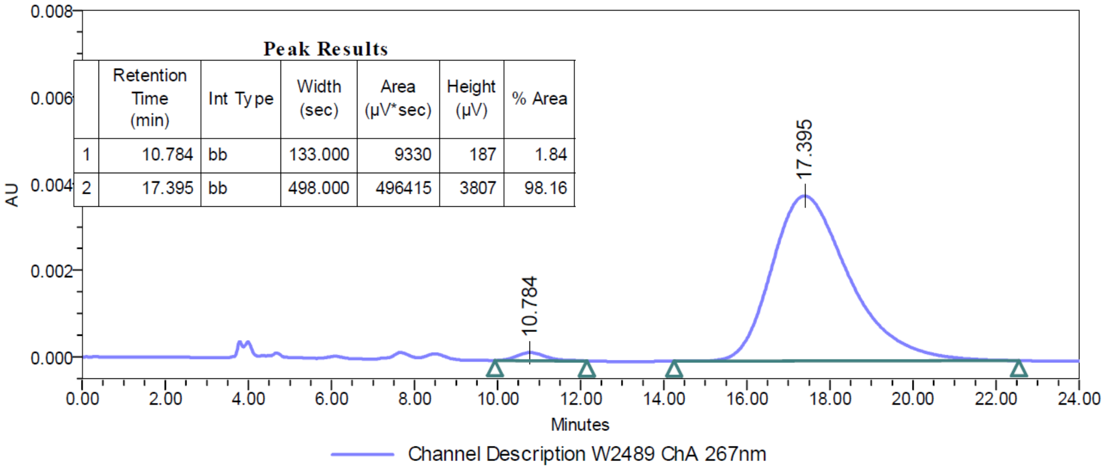

Peak Results

	Retention Time (min)	Int Type	Width (sec)	Area (µV*sec)	Height (µV)	% Area
1	10.784	bb	133.000	9330	187	1.84
2	17.395	bb	498.000	496415	3807	98.16

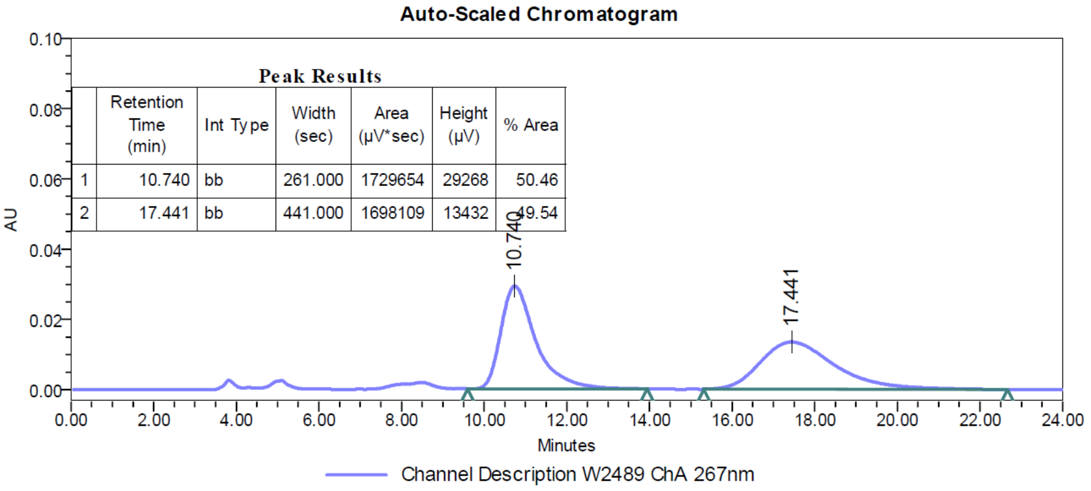

Peak Results

	Retention Time (min)	Int Type	Width (sec)	Area (µV*sec)	Height (µV)	% Area
1	10.740	bb	261.000	1729654	29268	50.46
2	17.441	bb	441.000	1698109	13432	49.54

6 Crystal structure of compound 3o

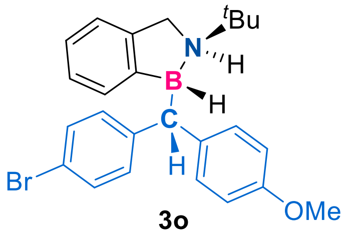

3o

CCDC 2353804

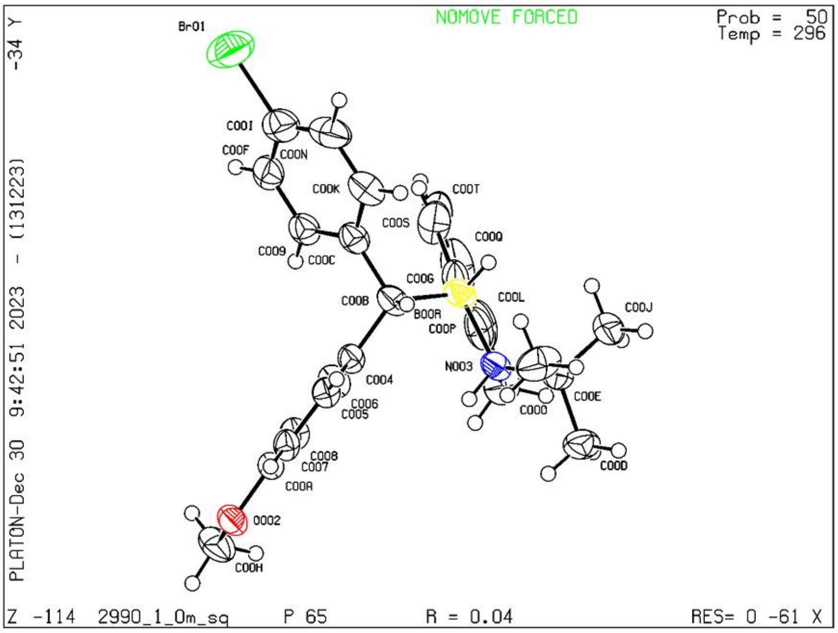

Bond precision:	C-C = 0.0072 A		Wavelength=0.71073
Cell:	a=19.7753(11)	b=19.7753(11)	c=11.5911(7)
	alpha=90	beta=90	gamma=120
Temperature:	296 K		

	Calculated	Reported
Volume	3925.6(5)	3925.6(5)

Space group	P 65	P 65
Hall group	P 65	P 65
Moiety formula	C25 H29 B Br N O [+ solvent]	C25 H29 B Br N O
Sum formula	C25 H29 B Br N O [+ solvent]	C25 H29 B Br N O
Mr	450.20	450.21
Dx,g cm-3	1.143	1.143
Z	6	6
Mu (mm-1)	1.584	1.584
F000	1404.0	1404.0
F000'	1402.73	
h,k,lmax	23,23,13	23,23,13
Nref	4669[2461]	4634
Tmin,Tmax		
Tmin'		

Correction method= Not given

Data completeness= 1.88/0.99 Theta(max)= 25.100

R(reflections)= 0.0360(3858) wR2(reflections)= 0.0987(4634)

S = 1.034 Npar= 271

7 Computational study

7.1 Computational methods

All DFT calculations were carried out with the *Gaussian 16* program.[5] The B3LYP functional[6,7] with Grimme's D3(BJ) dispersion correction[8] was used to optimize the molecular geometries. All atoms were described using the def2-SVP[9] basis set. The quasi-RRHO model proposed by Truhlar is used to handle the low-frequency vibrational modes.[10] Frequency calculations and thermal corrections (203.15 K and 1 atm) were carried out at the same level in the SMD solvation model. [11,12] Intrinsic reaction coordinates (IRC) were carried out to confirm all transition states. Multiple conformations have been considered during the calculations and the lowest-energy ones are reported. The conformational search was carried by using Grimme's program xTB 6.3 and CREST 2.10.2.[13,14] Single-point energy calculations were performed at the PBE0-D3(BJ) functional with def2-TZVP basis set using the SMD solvation model. The IGMH analysis was carried by using Multiwfn 3.8(dev).[15] All 3D structures were generated using CYLView.[16]

7.2 Computational Details

To explain the origin of stereoselectivity in the reaction and the excellent selectivity of ligand **L1**, we summarized the common structural features of ligands **L1**-**L6**, constructed a model ligand **L3**, and

studied the asymmetric hydrogen transfer steps of both **L3** and **L1** in the reaction shown at the top of Figure S2 through DFT calculations.

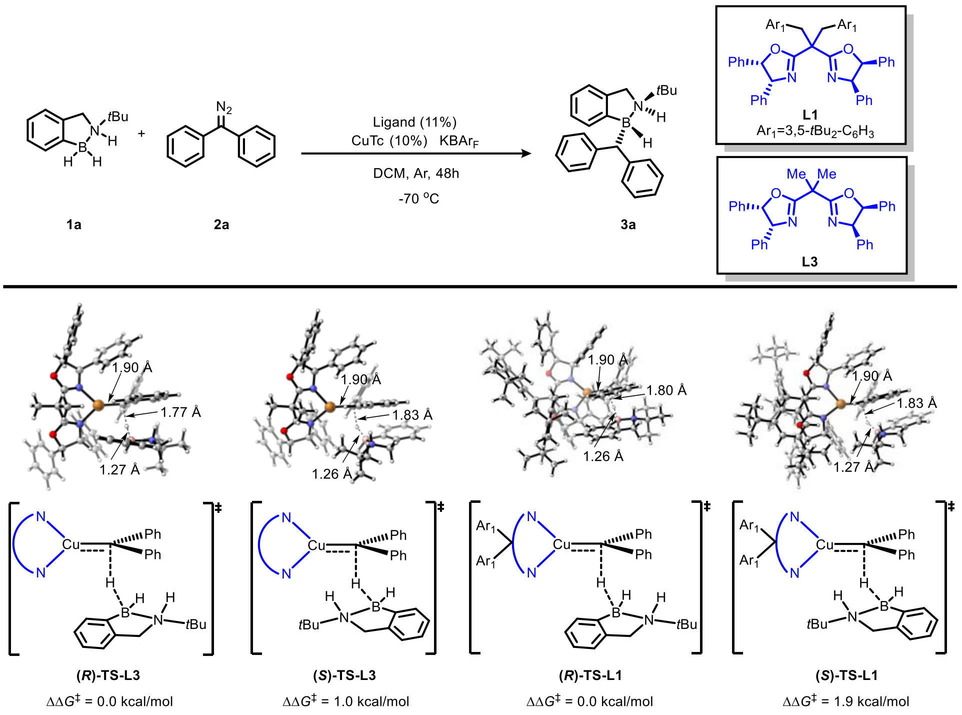

Figure S2. DFT-calculated key transition state structures for the enantio-determining hydride transfer step with 1a and 2a as substrates.

The corresponding pair of transition states, **(*R*)-TS-L3** and **(*S*)-TS-L3**, are shown in the bottom of Figure S2. Energy calculations indicate that the pathway through **(*R*)-TS-L3** has a lower activation free energy barrier, with **(*S*)-TS-L3** being 1.0 kcal/mol higher (at 203.15K). Furthermore, we investigated both transition states through IGMH analysis to visualize how non-covalent interactions influence reaction selectivity, as illustrated in Figure S3. The IGMH analysis results show that one of the aromatic groups of the symmetric diazo compound **2a** in **(*R*)-TS-L3** effectively overlaps with the aromatic group of amine borane **1a**, forming distinct π-π interaction, which we postulate as one source of the reaction's stereoselectivity.

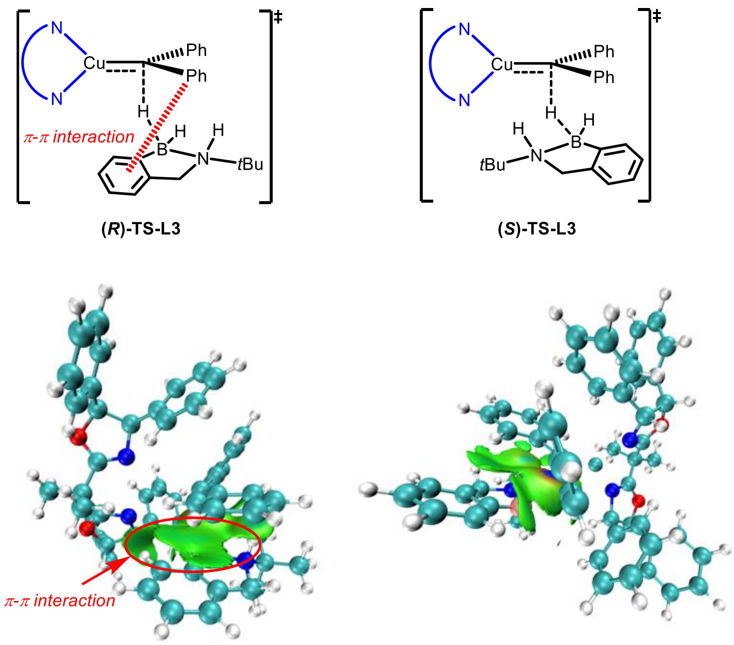

Figure S3. The IGMH analysis for the transition states (*R*)-TS-L3 and (*S*)-TS-L3

To better quantify the stereoselectivity factors in the transition states, we conducted Distortion-Interaction analysis on both transition states involving the **L3** ligand, with results presented in Table S7. The analysis reveals that while the **(*R*)-TS-L3** transition state structure shows a slight disadvantage in distortion energy (+0.3 kcal/mol) compared to **(*S*)-TS-L3**. It demonstrates a significant advantage in interaction energy (-0.7 kcal/mol). This DI analysis further corroborates the results of IGMH analysis, confirming that π-π interaction between substrates is crucial in favoring ***R***-configured product formation. Subsequently, building upon the **L3** ligand catalytic model, we further investigated the reaction transition states involving ligand **L1** to provide a rational explanation for its relatively higher stereoselectivity and guide future ligand optimization. As shown in Figure S2, the asymmetric hydrogen transfer transition states for ligand **L1** are **(*R*)-TS-L1** and **(*S*)-TS-L1**, with a calculated energy difference of 1.9 kcal/mol, slightly higher than the corresponding experimental value of 1.3 kcal/mol (91% *ee* at 203K). Considering that the main structural difference between **L1** and **L3** lies in the two outward-facing aromatic substituents, we reasoned that their effect on π-π interaction energy would be minimal. Therefore, we directly employed DI analysis for further investigation, with results shown in Table S7. The calculations reveal that, compared to transition states involving ligand **L3**, the main difference lies in the significantly increased distortion of the diazo compound in **(*S*)-TS-L1** relative to **(*R*)-TS-L1**. This results in the ***R***-configured transition state of **L1**-catalyzed hydride transfer having both substantial advantages in interaction energy (-1.6 kcal/mol) and distortion energy (-1.5 kcal/mol).

Table S7. The DI analysis results of the transition states mediated by L3 and L1.

Energy/ kcal mol^{-1}	(*R*)-TS-L3	(*S*)-TS-L3	(*R*)-TS-L1	(*S*)-TS-L1
ΔE_{dist}(carbene)	2.6	2.5	2.6	4.0
ΔE_{dist}(borane)	1.3	1.1	1.1	1.3
ΔE_{dist}(total)	3.9	3.6	3.8	5.3
ΔE_{int}	-12.4	-11.7	-14.9	-13.3

Comparing the transition state structures and DI analysis results of **L3** and **L1** ligands, we propose that the weak interactions between the aromatic group in the symmetric diazo compound and the aromatic group in amine borane are one of the key factors leading to stereoselectivity tendency in the reaction. Meanwhile, the excellent selectivity of ligand **L1** may be due to the large steric hindrance between its bulky substituents and the substituent in amine borane, which leads to further distortion of the copper carbene fragment in the transition state, thereby increasing the activation energy difference between transition states.

To investigate the origin of diastereoselectivity in this reaction, we selected unsymmetrical para-cyano-substituted diaryldiazomethane as the substrate and employed **L3** as the model ligand in searching for the four stereoisomeric transition states. The results The results suggest that with unsymmetrical diaryldiazomethanes, the previously lowest-energy ***R***-configured transition state underwent a conformational shift, rendering the **TS-*R*-*R*** (boron stereochemistry first, then carbon) configuration energetically most favorable due to an N-H···π interaction between the N-H bond of substrate 1a and the phenyl ring, alongside a π···π interaction between the additional cyano-substituted phenyl ring and the catalyst's pendant phenyl ring (Figure S4). Although both weak interactions were present in **TS-*R*-*S***, the N-H bond of **1a** preferentially engaged with the electron-rich aromatic ring, elucidating why larger electronic differences between the two aryl rings in asymmetric diaryldiazomethanes lead to higher dr values, such as **3o**.

To better quantify the stereoselectivity in the transition states, we performed distortion-interaction analysis on the aforementioned four transition states, with the results summarized in Table S8; **TS-*R*-*R*** exhibited the largest interaction energy, validating the strongest non-covalent interactions present, while the higher distortion energy of **TS-*R*-*S*** relative to **TS-*R*-*R*** indicated more pronounced steric repulsion leading to its elevated energy, which corresponds precisely with the experimental findings (**3af-3aj**) showing that reducing the steric bulk of the N-substituent significantly decreases the dr value; this DI analysis further confirms that both weak interactions between substrates and spatial positioning effects are crucial for favoring the formation of the ***R,R***-configured product.

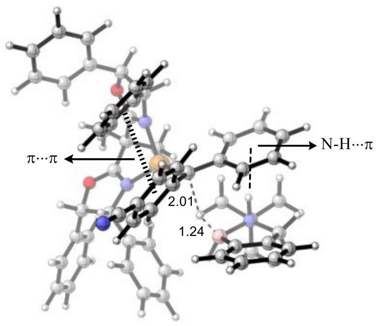

TS-*R*-*R* (Major product)
ΔΔ*G* = +0.0 kcal/mol

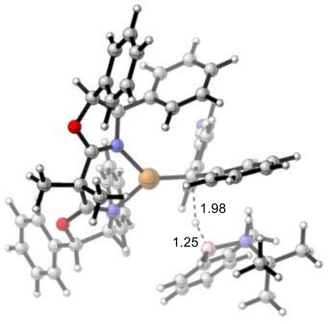

TS-*S*-*S* (Enantiomer)
ΔΔ*G* = +1.6 kcal/mol

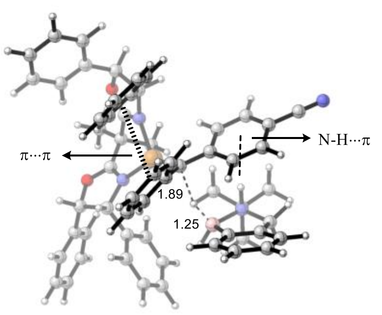

TS-*R*-*S* (Diastereomer)
ΔΔ*G* = +1.6 kcal/mol

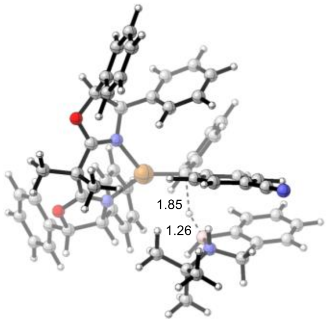

TS-*S*-*R* (Diastereomer)
ΔΔ*G* = +0.6 kcal/mol

Figure S4. DFT-calculated key transition state structures for asymmetric diaryldiazomethanes.

Table S8. The DI analysis results of the four transition states.

Energy/ kcal mol^{-1}	TS-*R*-*R*	TS-*S*-*S*	TS-*R*-*S*	TS-*S*-*R*
ΔE_{dist}(carbene)	5.3	1.9	6.6	2.7
ΔE_{dist}(borane)	0.5	0.5	0.7	1.1
ΔE_{dist}(total)	5.8	2.4	7.3	3.8
ΔE_{int}	-12.0	-10.4	-11.8	-11.8

8 NMR spectra

2-(tert-butyl)-2,3-dihydro-1H-benzo[c][1,2]azaborole (1a)

^{1}H NMR (500 MHz, $CDCl_3$)

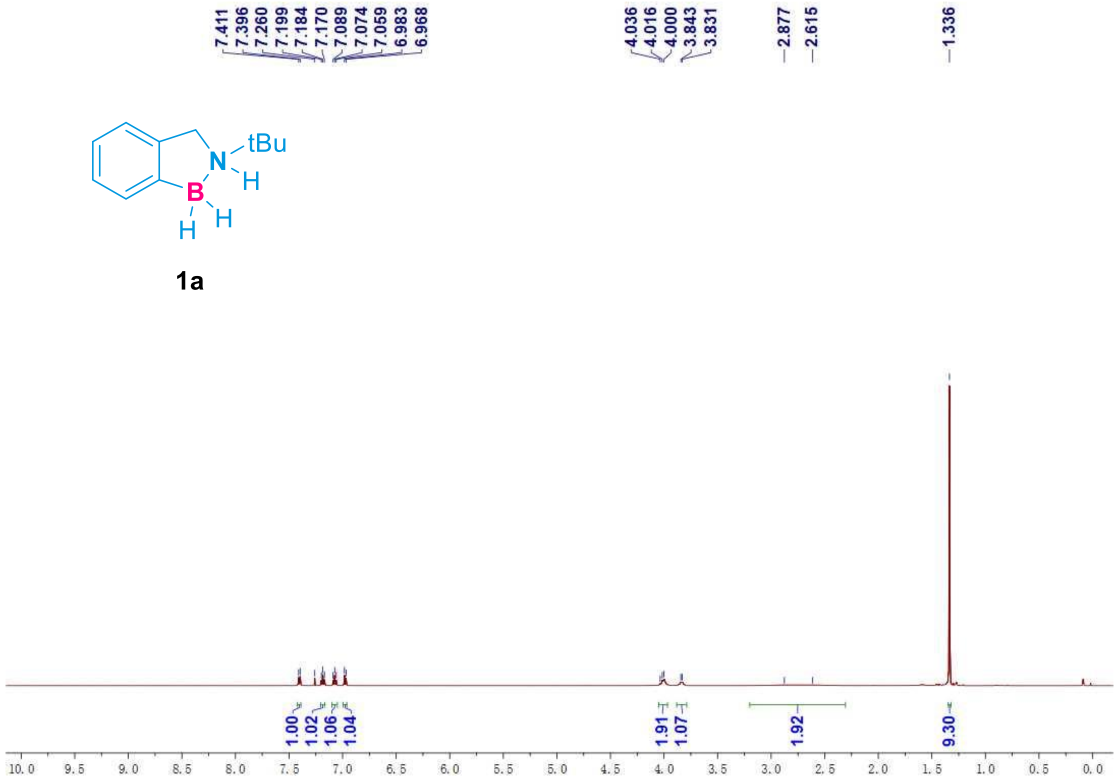

^{13}C NMR (126 MHz, $CDCl_3$)

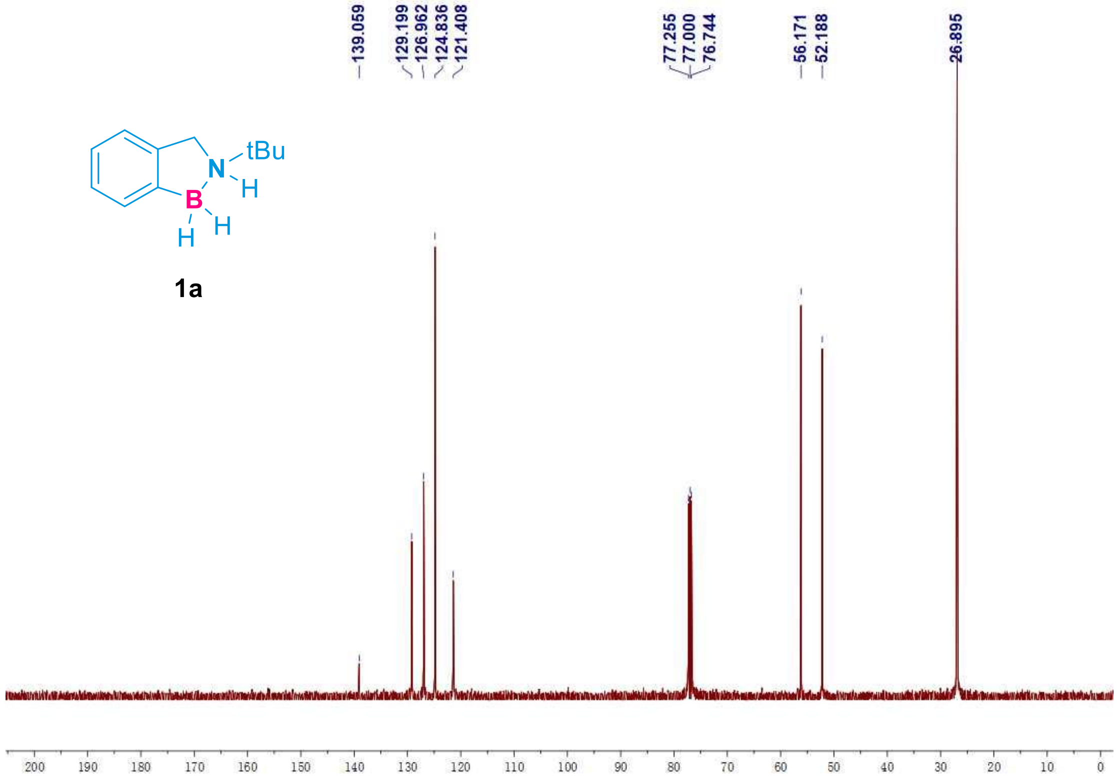

^{11}B NMR (160 MHz, $CDCl_3$)

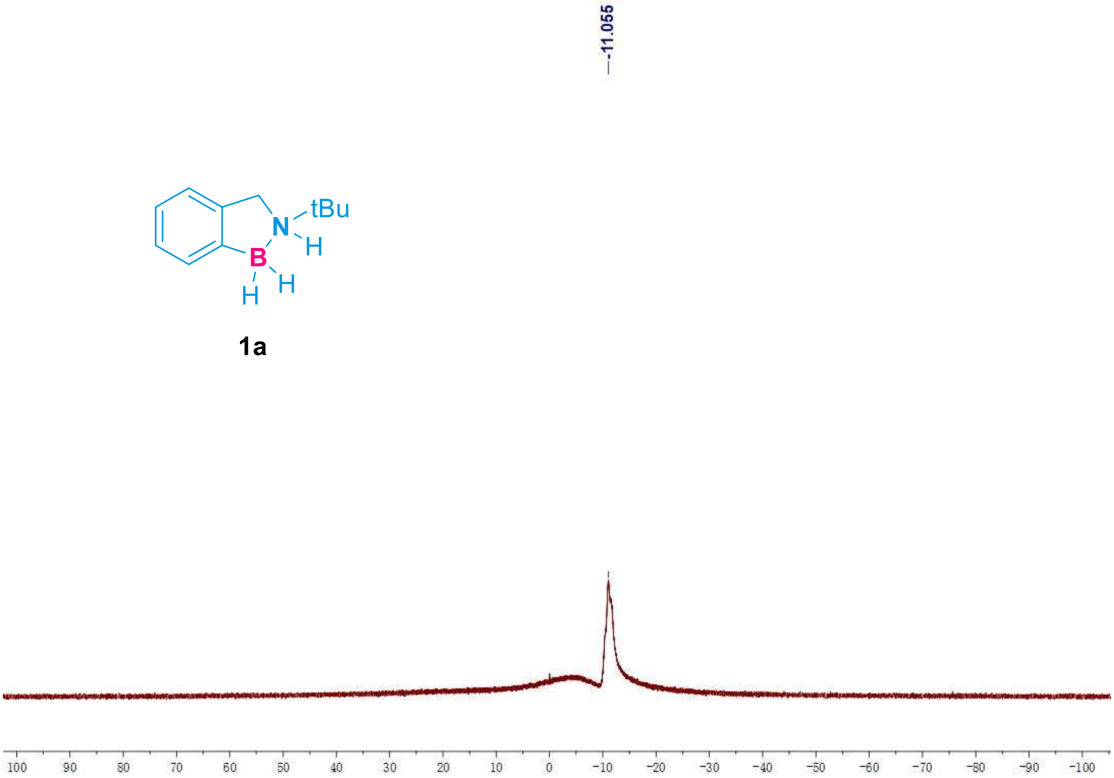

2-(tert-butyl)-5-methoxy-2,3-dihydro-1H-benzo[c][1,2]azaborole (1b)

^{1}H NMR (500 MHz, $CDCl_3$)

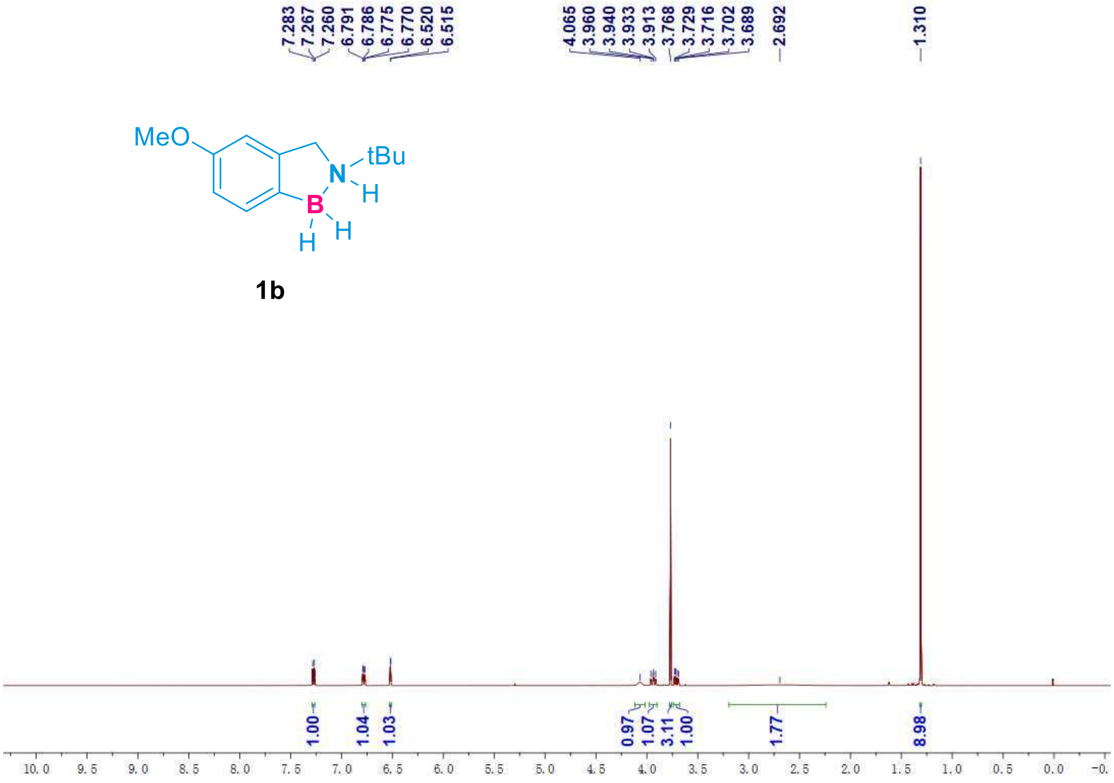

^{13}C NMR (126 MHz, $CDCl_3$)

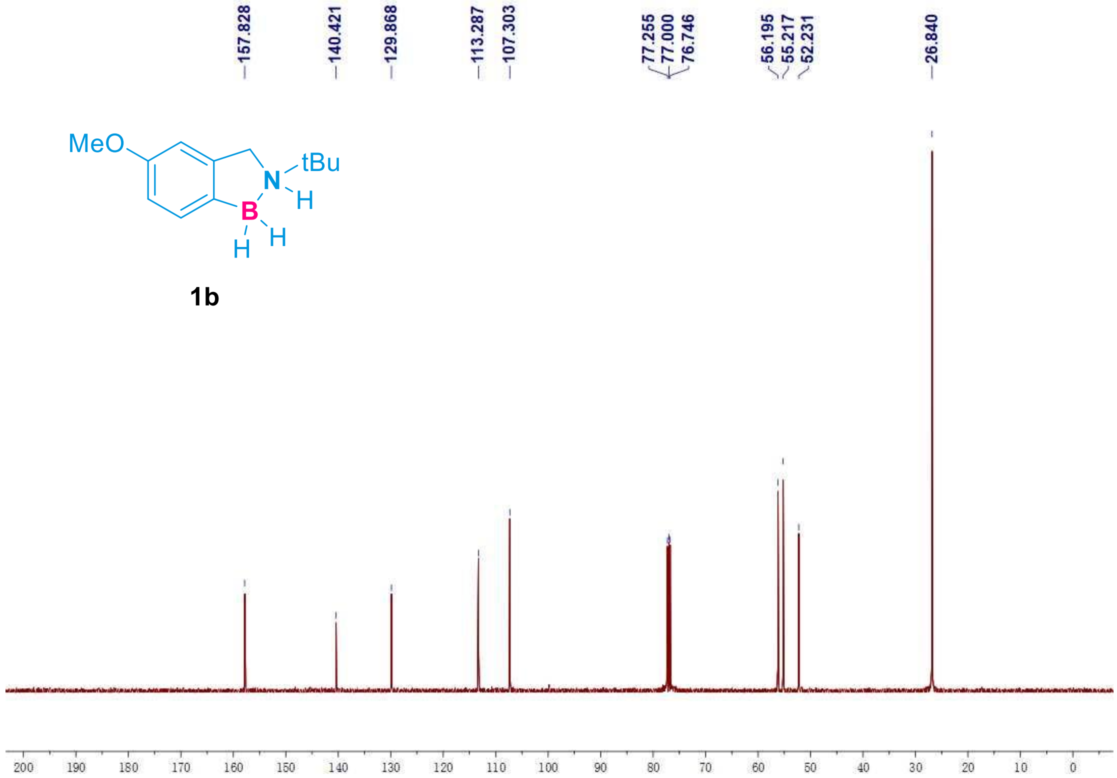

^{11}B NMR (160 MHz, $CDCl_3$)

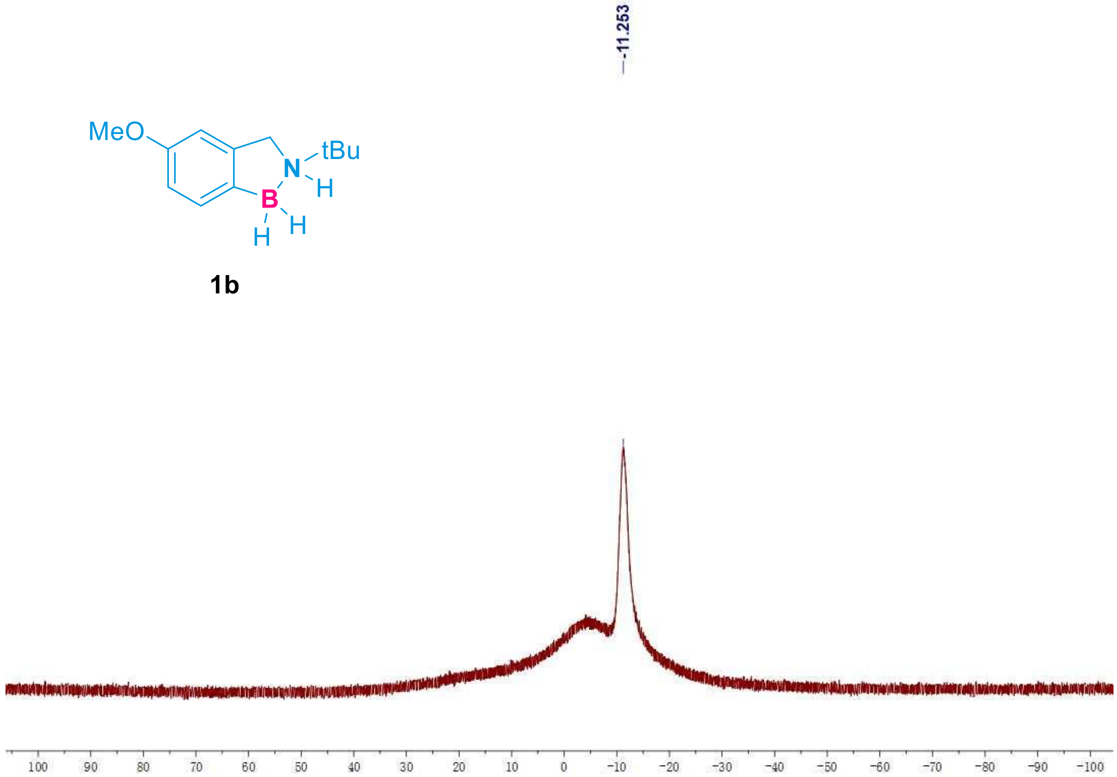

2-(tert-butyl)-5-methyl-2,3-dihydro-1H-benzo[c][1,2]azaborole (1c)

^{1}H NMR (500 MHz, $CDCl_3$)

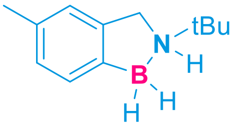

1c

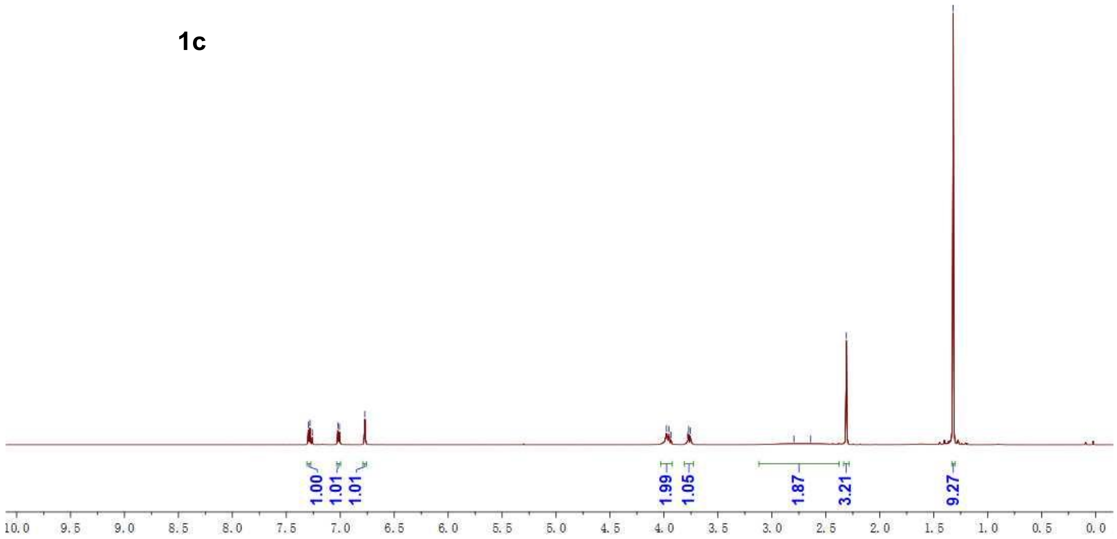

^{13}C NMR (126 MHz, $CDCl_3$)

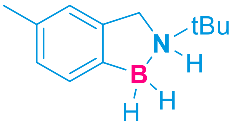

1c

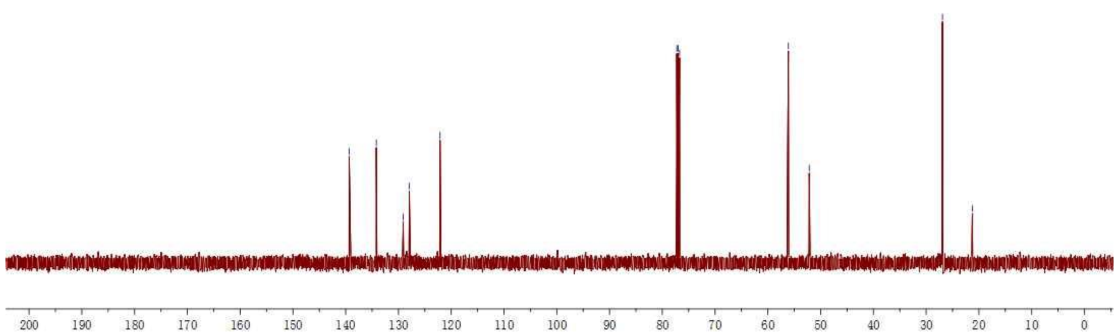

[11]**B NMR** (128 MHz, $CDCl_3$)

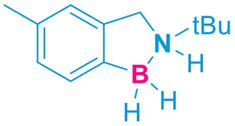

1c

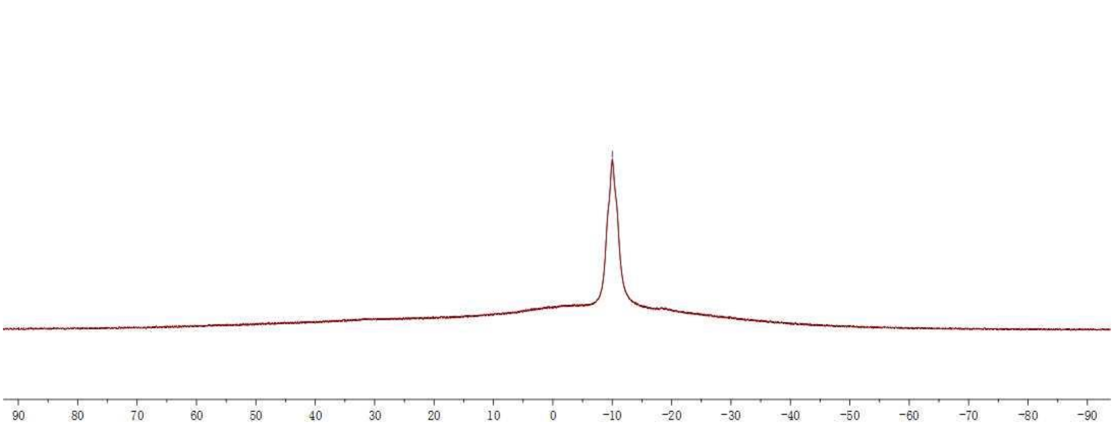

2-(tert-butyl)-5-chloro-2,3-dihydro-1H-benzo[c][1,2]azaborole (1d)

[1]**H NMR** (500 MHz, $CDCl_3$)

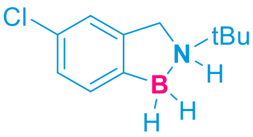

1d

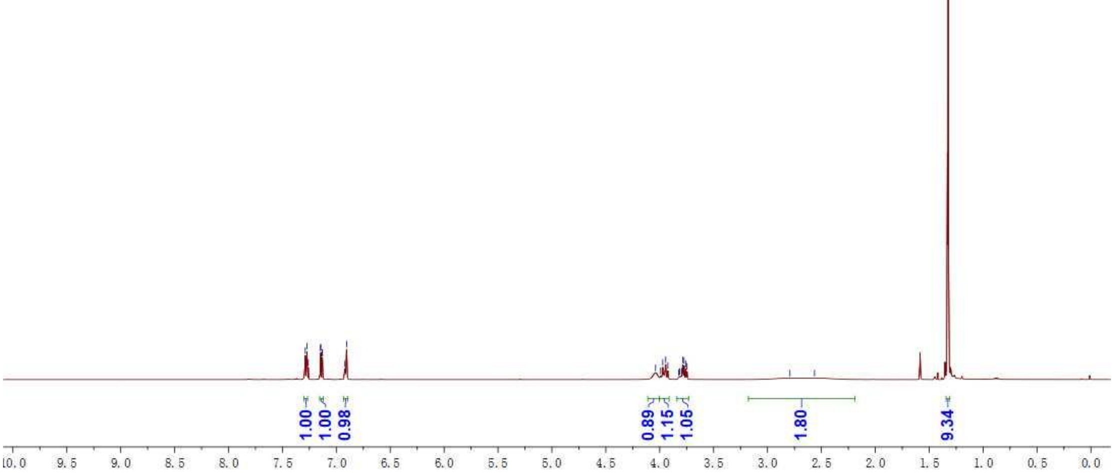

^{13}C NMR (126 MHz, $CDCl_3$)

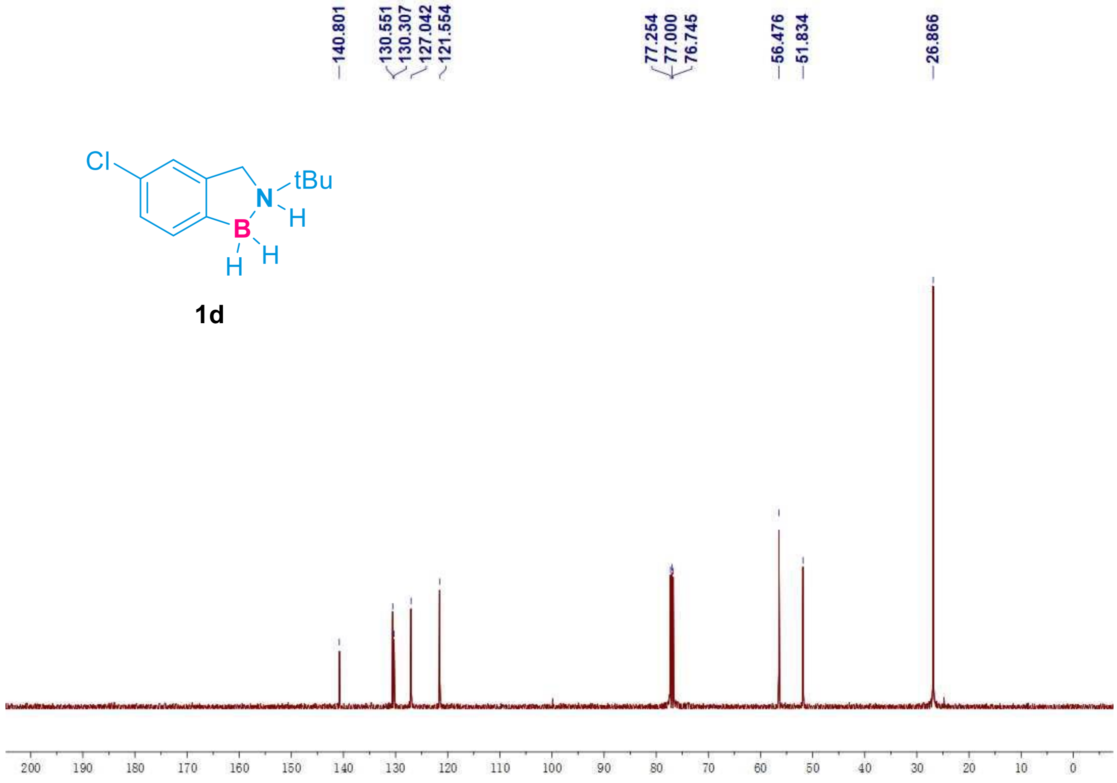

^{11}B NMR (160 MHz, $CDCl_3$)

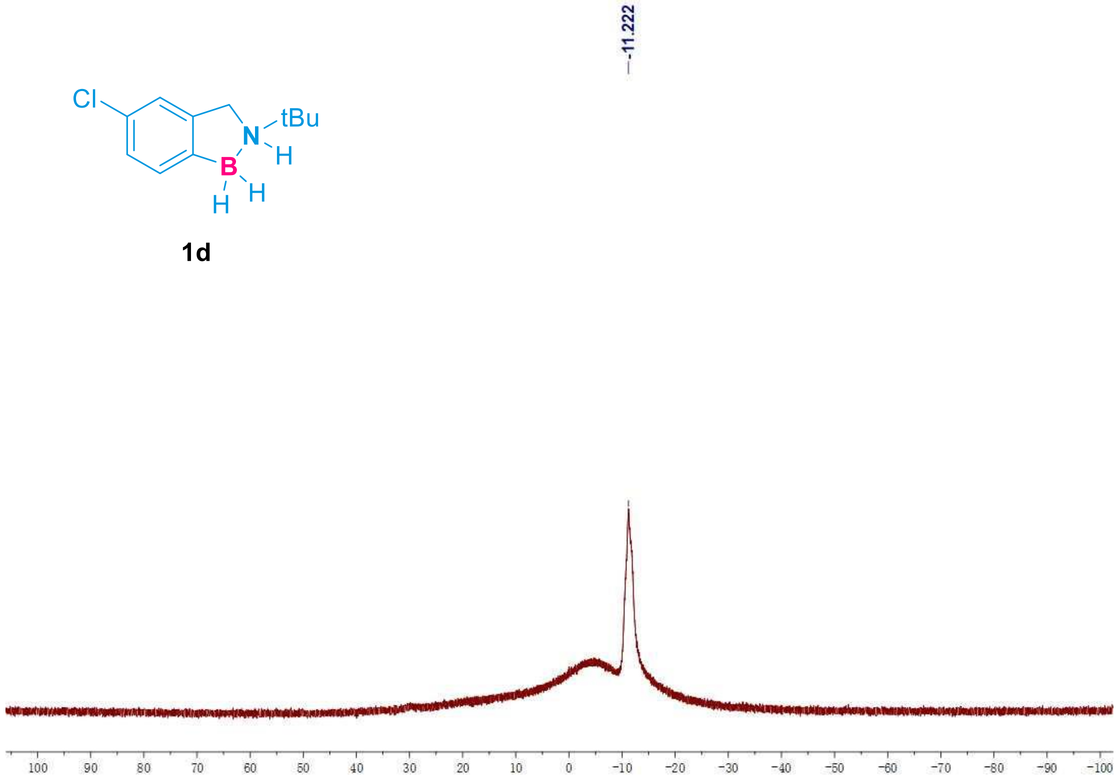

2-(tert-butyl)-6-fluoro-2,3-dihydro-1H-benzo[c][1,2]azaborole (1e)

^{1}H NMR (500 MHz, $CDCl_3$)

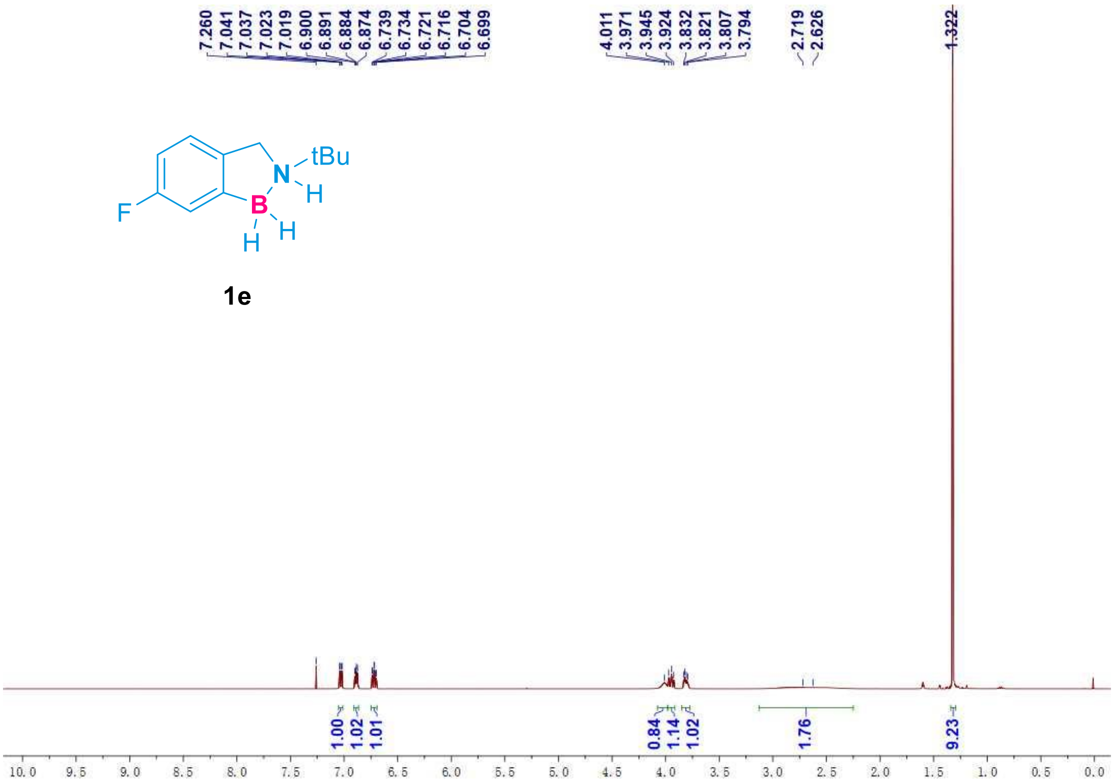

^{13}C NMR (101 MHz, $CDCl_3$)

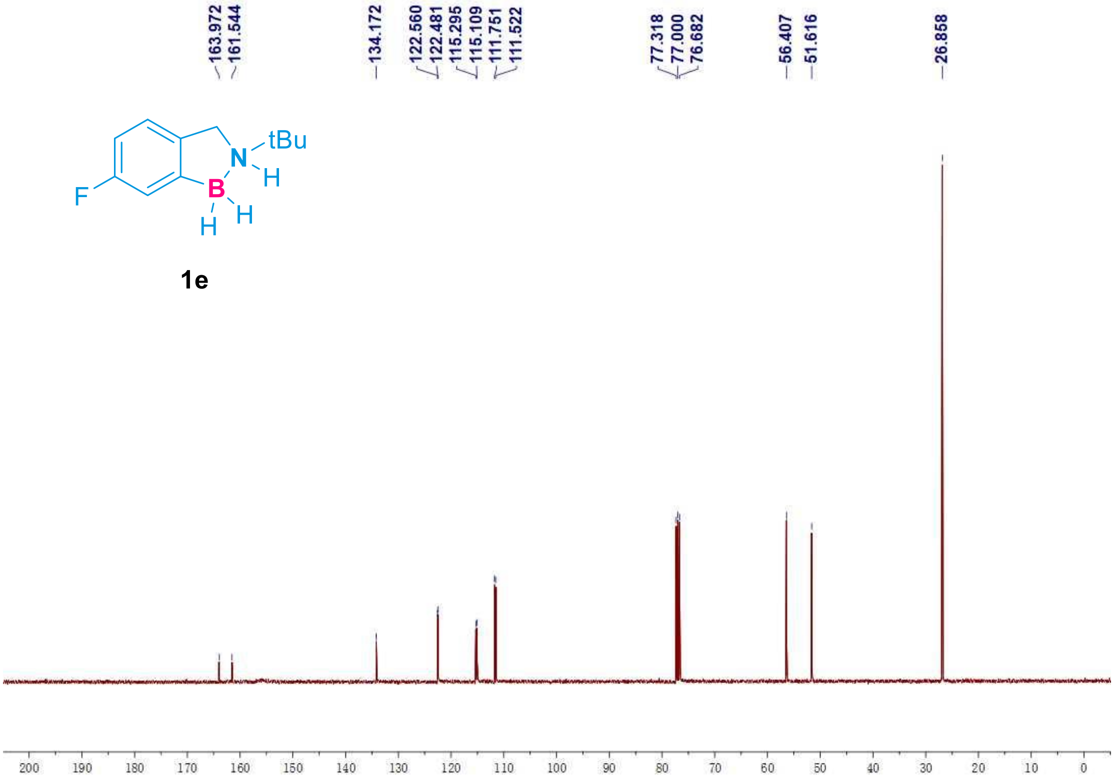

^{11}**B NMR** (128 MHz, CDCl$_3$)

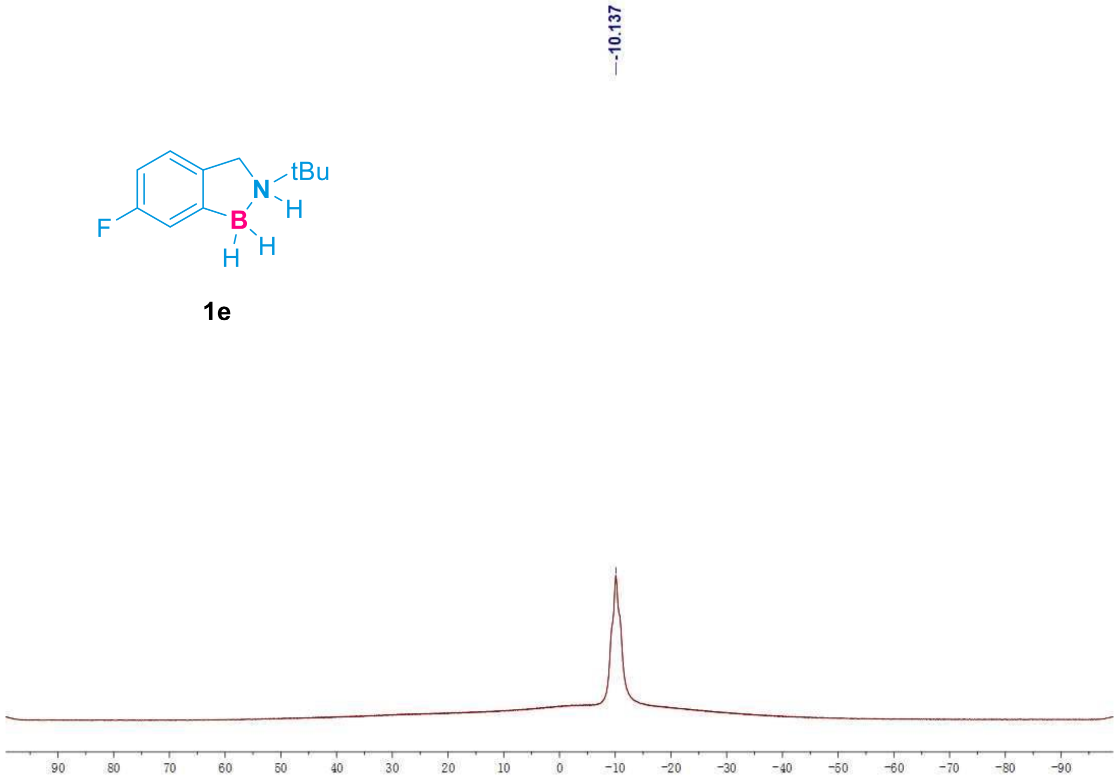

^{19}**F NMR** (376 MHz, CDCl$_3$)

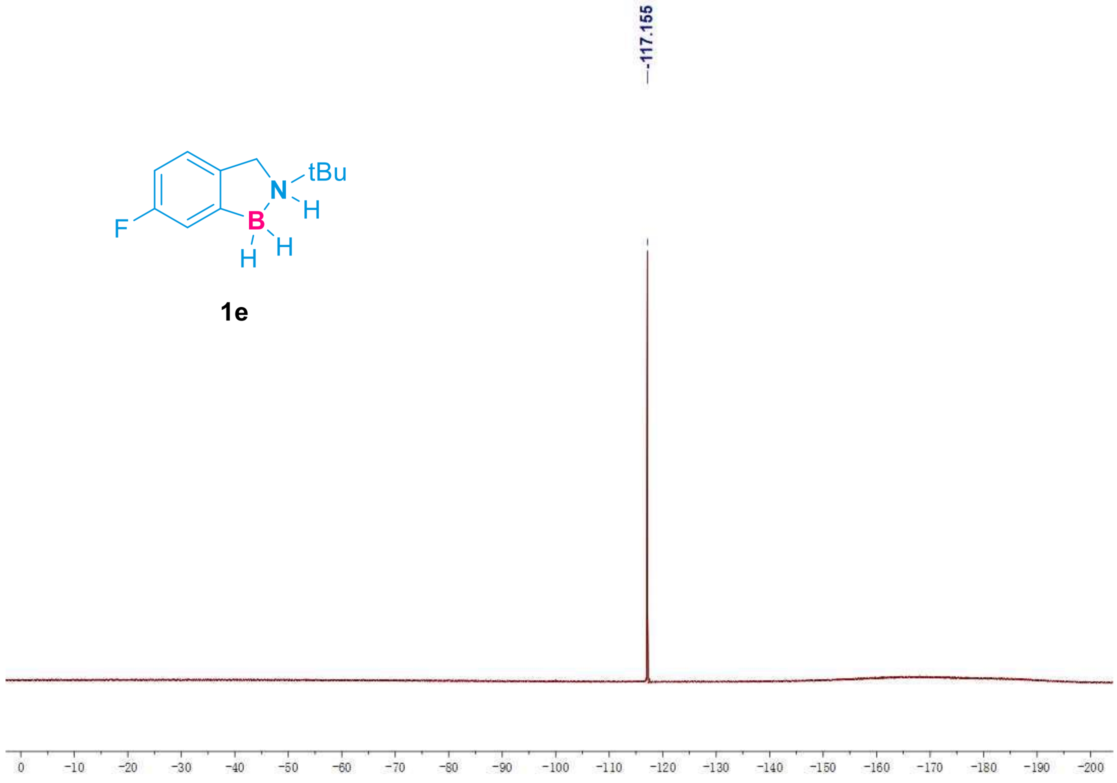

2-(tert-pentyl)-2,3-dihydro-1H-benzo[c][1,2]azaborole (1f)

^{1}H NMR (500 MHz, $CDCl_3$)

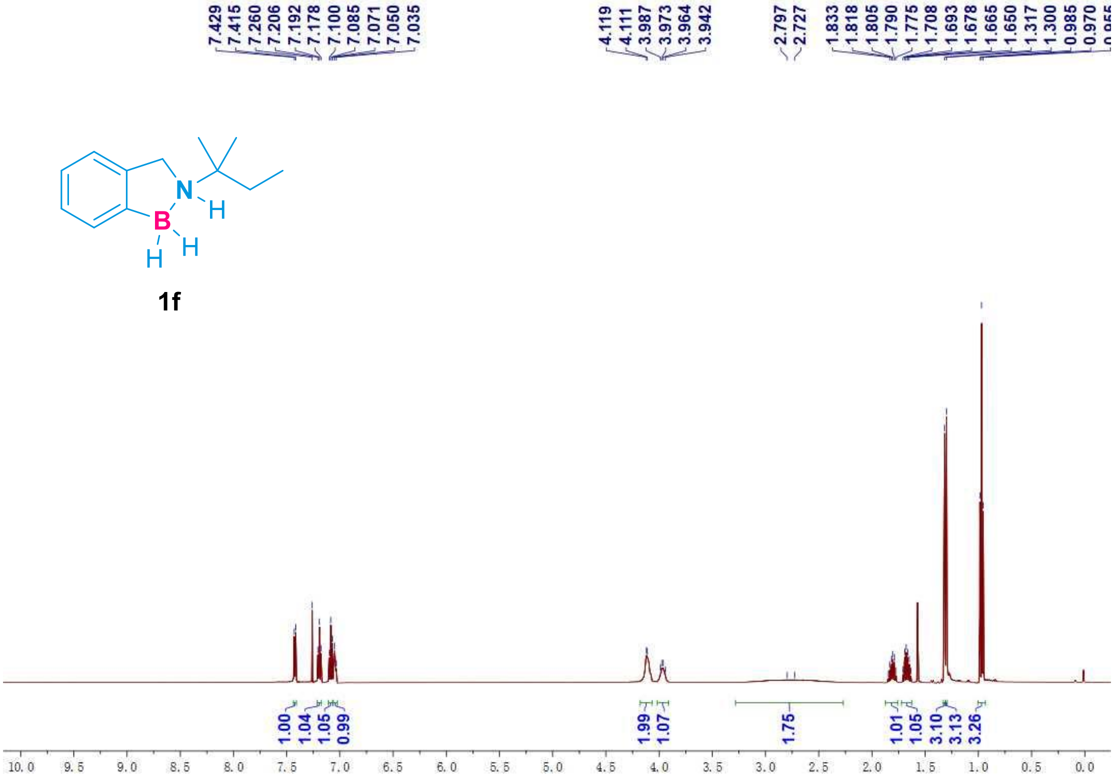

^{13}C NMR (101 MHz, $CDCl_3$)

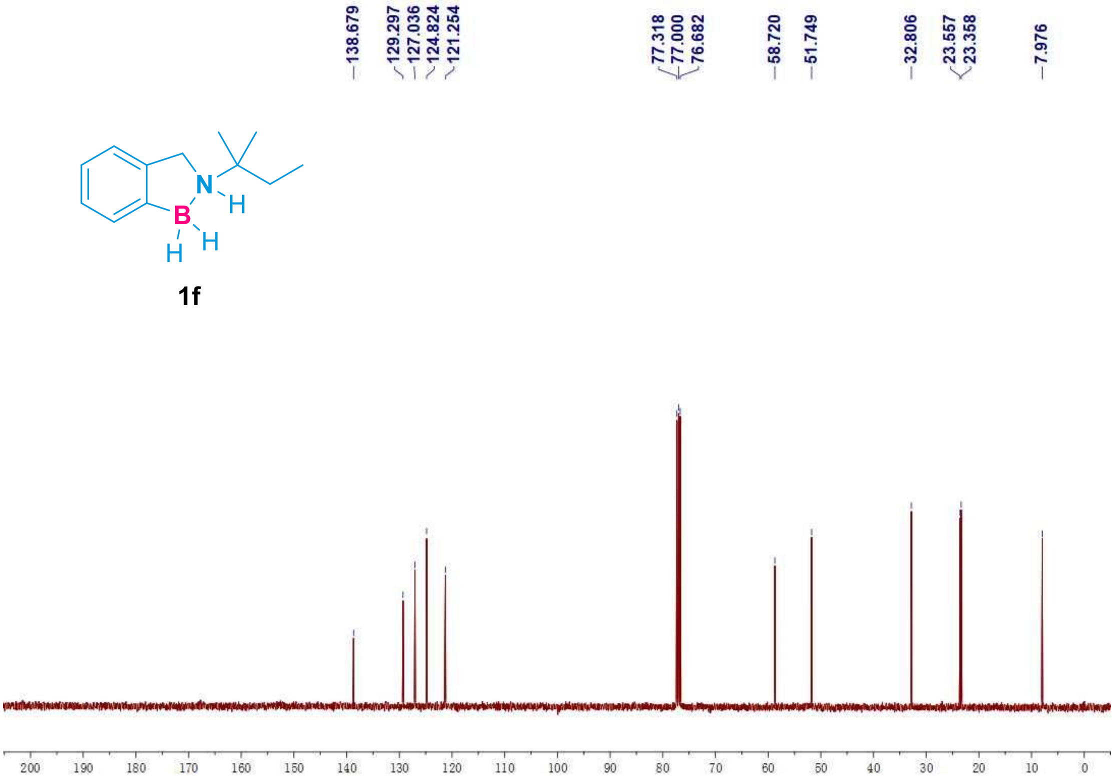

[11]**B NMR** (128 MHz, $CDCl_3$)

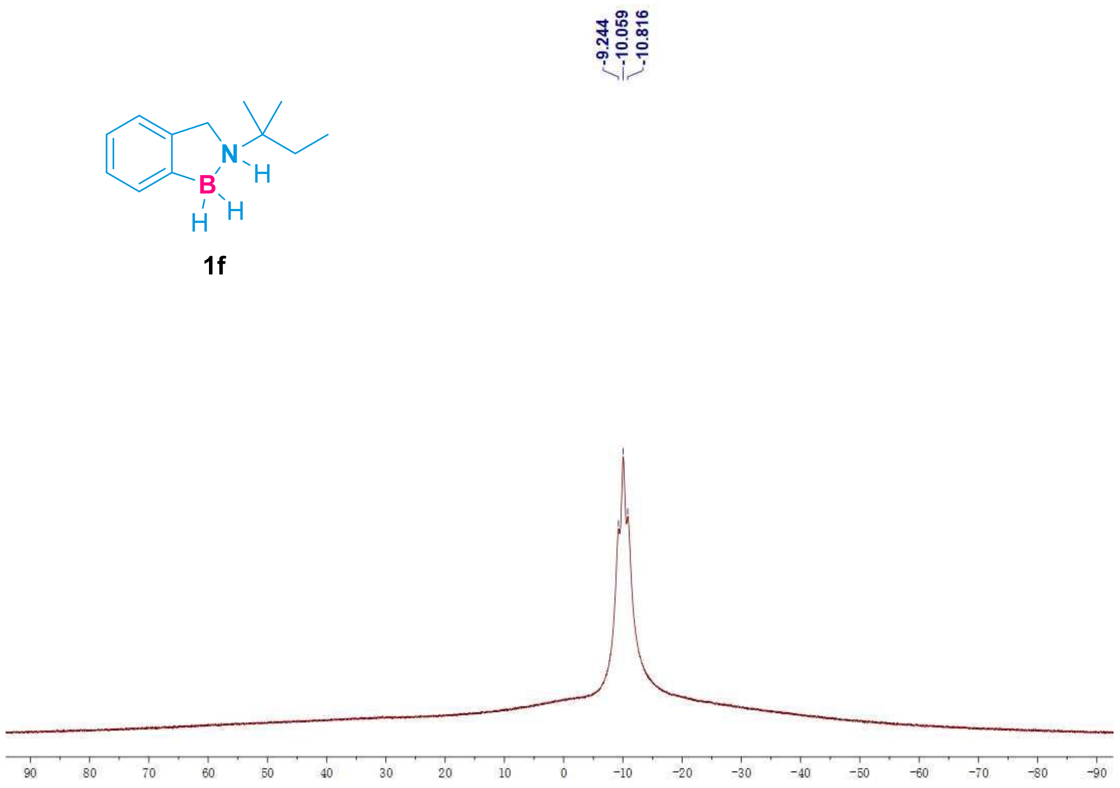

2-(2,4,4-trimethylpentan-2-yl)-2,3-dihydro-1H-benzo[c][1,2]azaborole (1g)

[1]**H NMR** (500 MHz, $CDCl_3$)

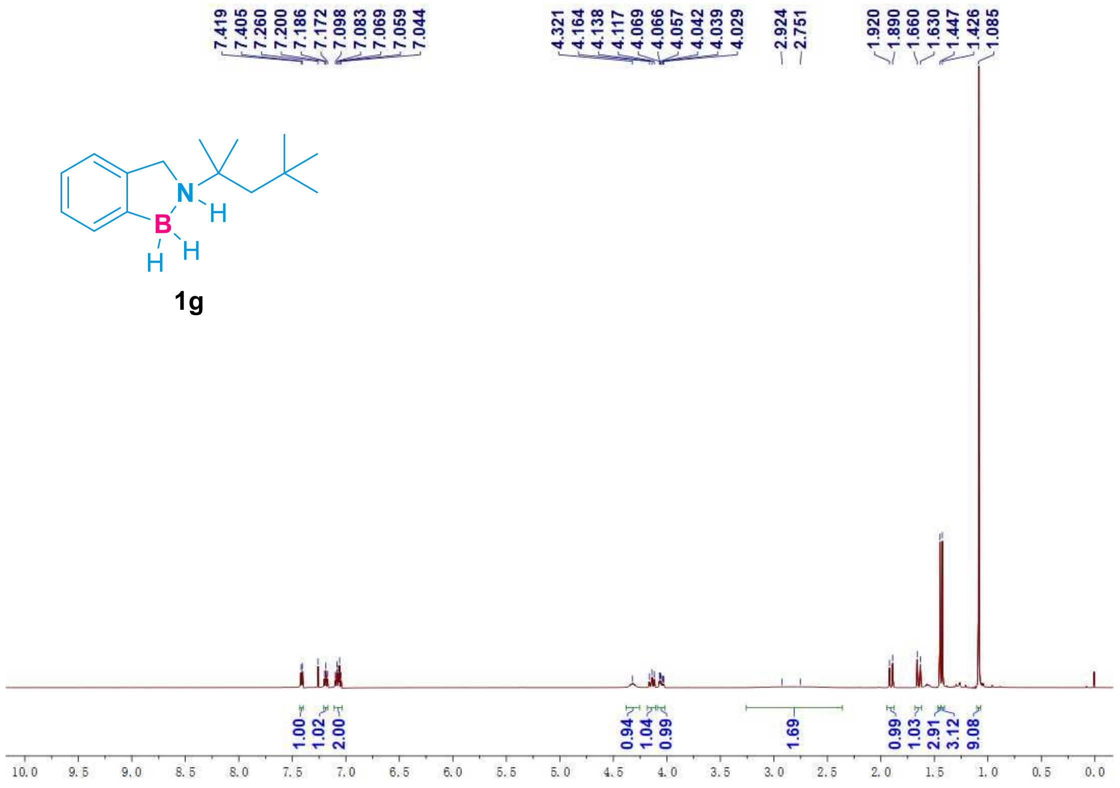

^{13}**C NMR** (126 MHz, $CDCl_3$)

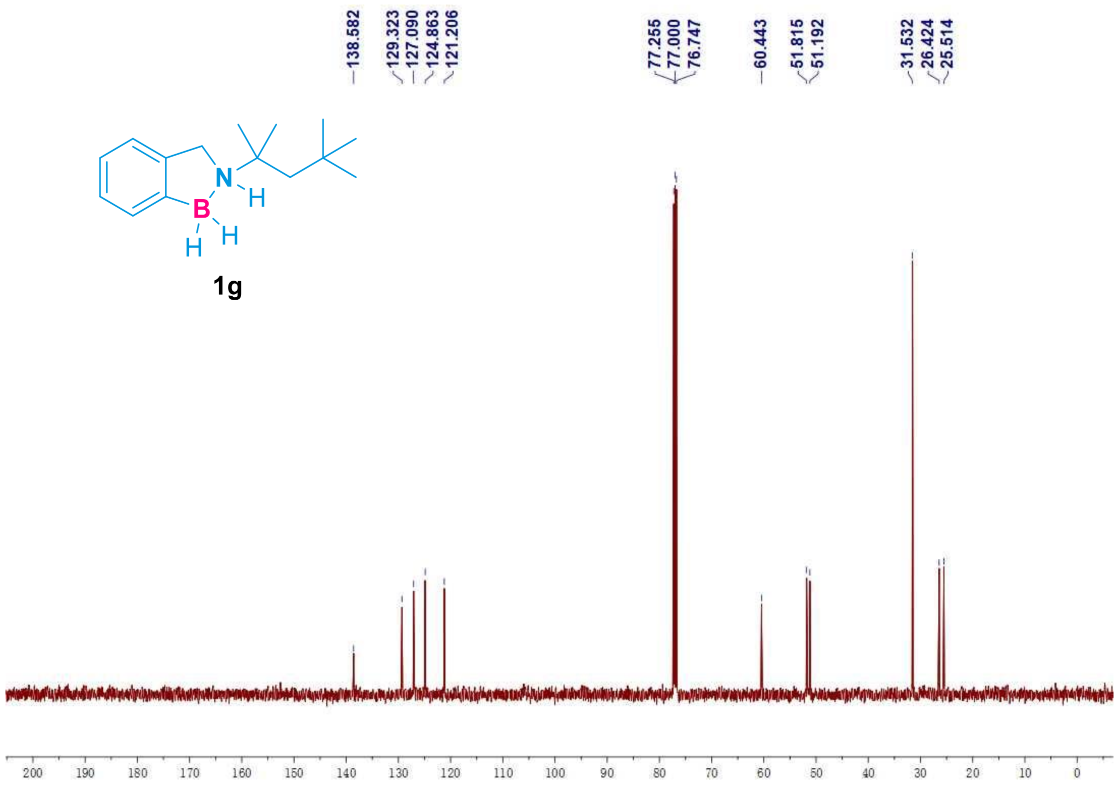

^{11}**B NMR** (160 MHz, $CDCl_3$)

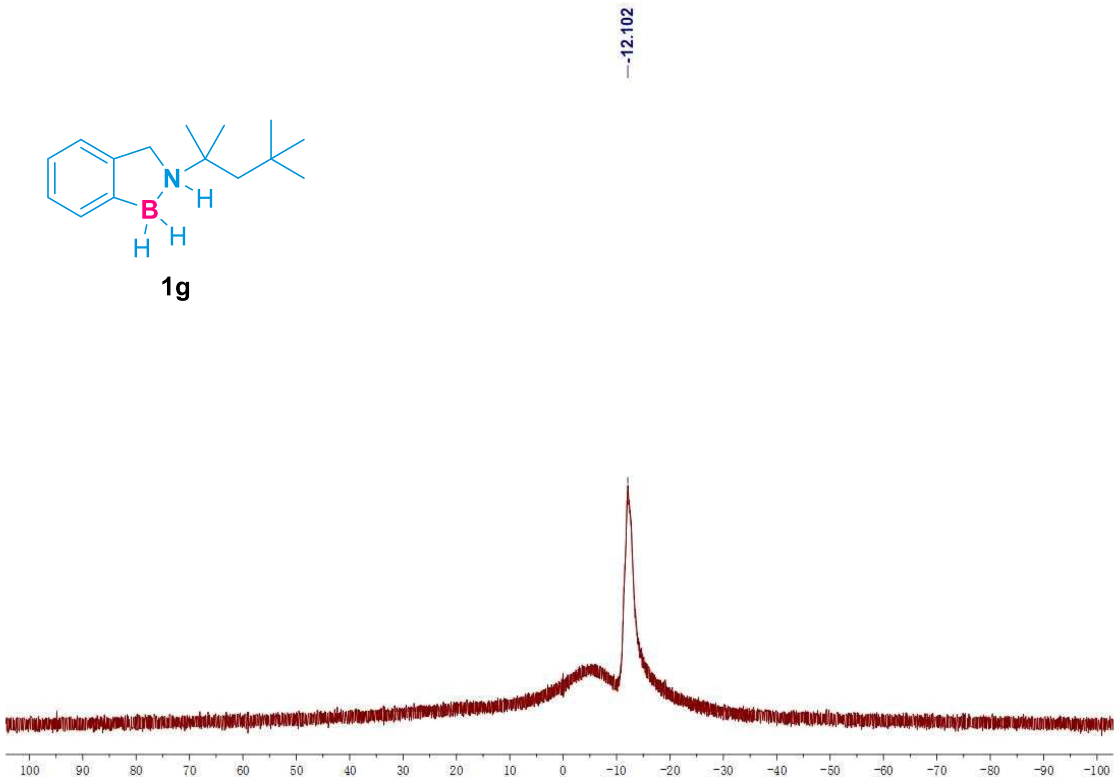

2-(2-phenylpropan-2-yl)-2,3-dihydro-1H-benzo[c][1,2]azaborole (1h)

^{1}H NMR (500 MHz, $CDCl_3$)

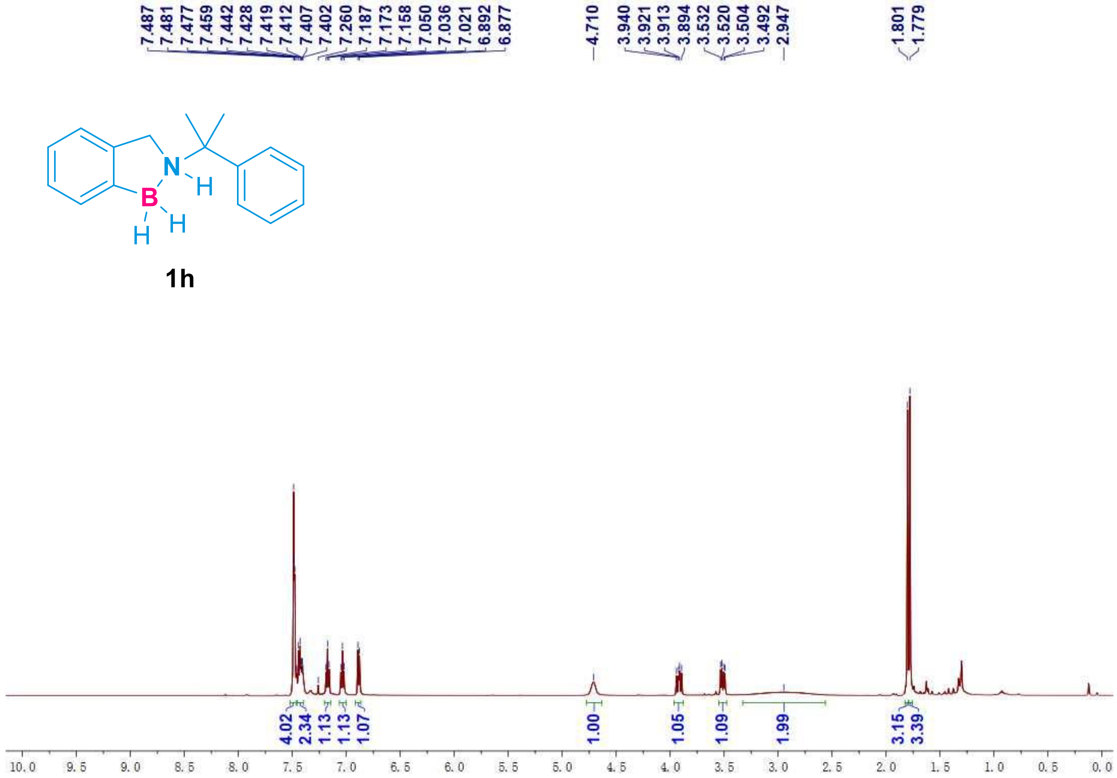

^{13}C NMR (126 MHz, $CDCl_3$)

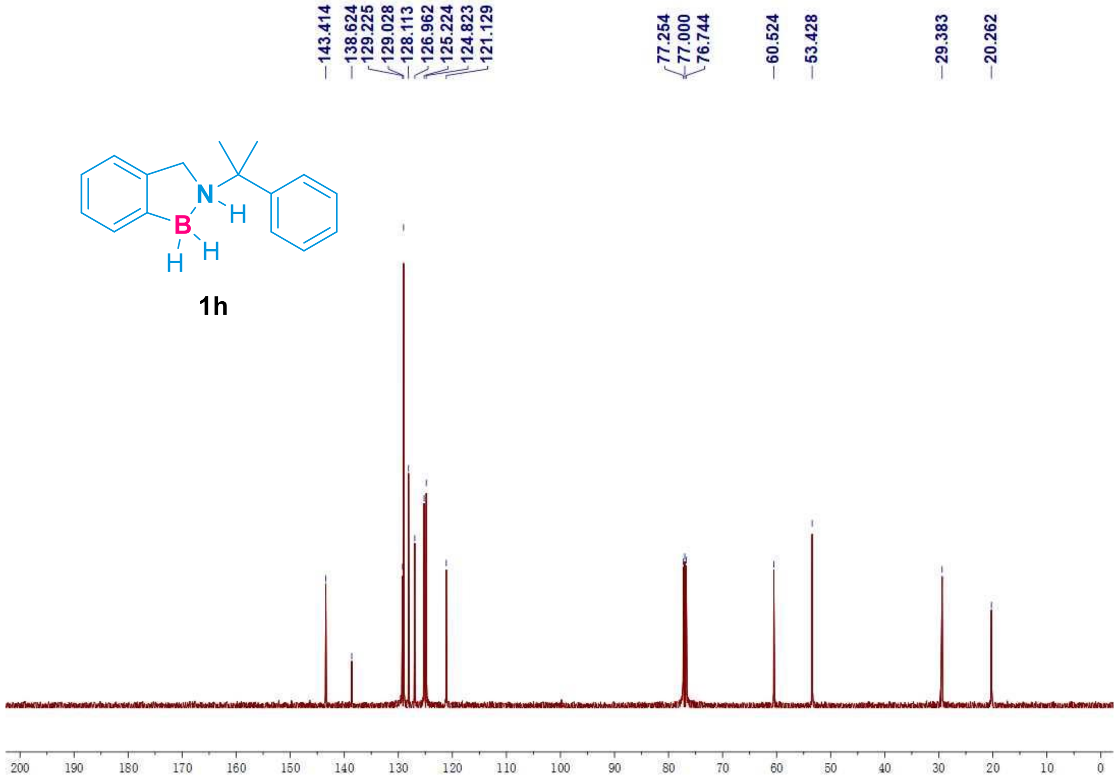

^{11}B NMR (160 MHz, CDCl$_3$)

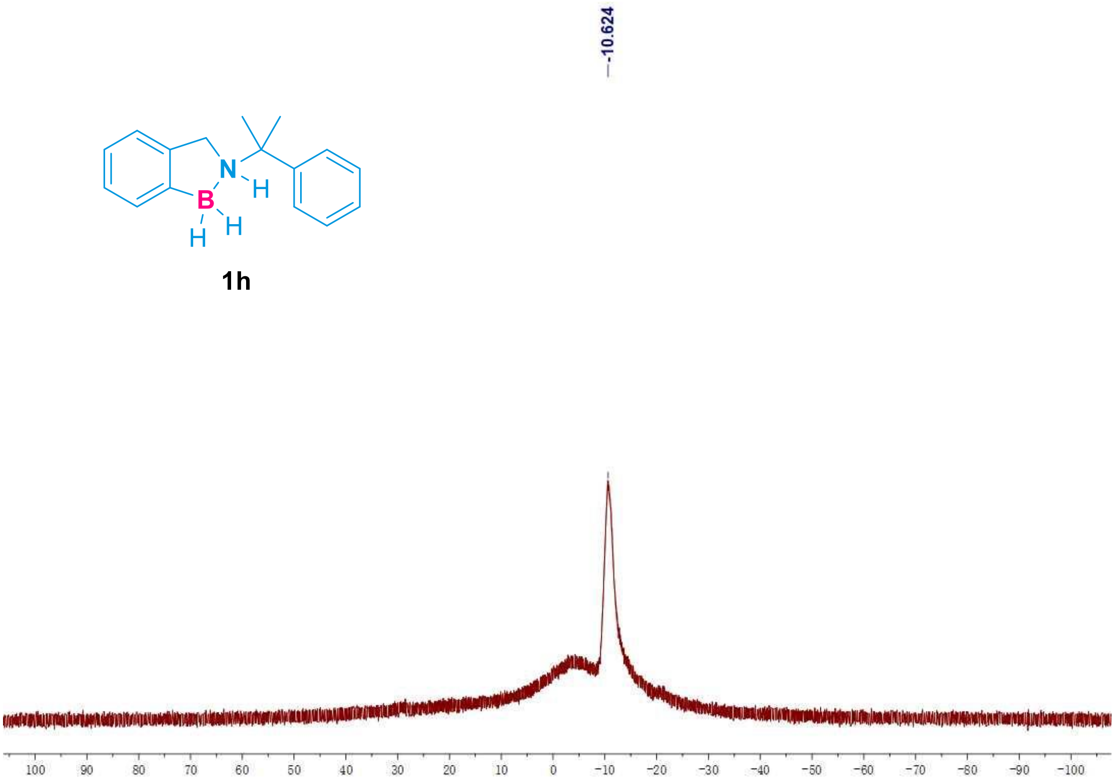

2-(1,3-dihydro-2H-benzo[c][1,2]azaborol-2-yl)-2-methylpropan-1-ol (1i)

^{1}H NMR (500 MHz, CDCl$_3$)

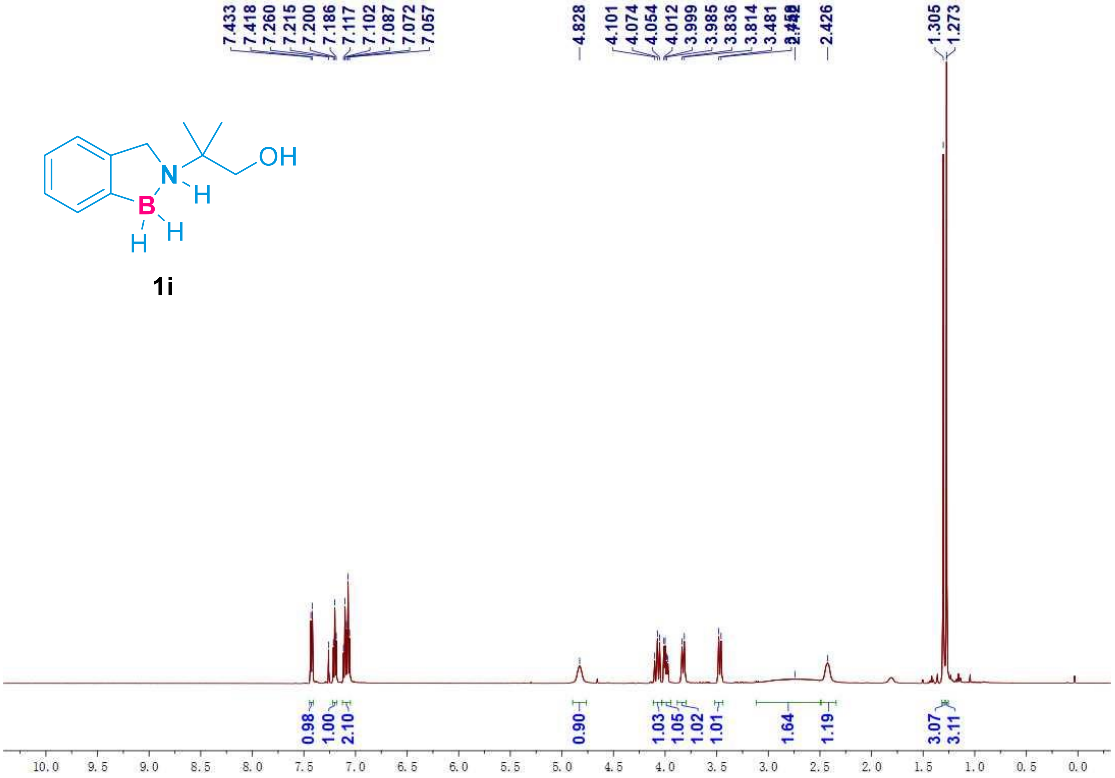

^{13}C NMR (126 MHz, $CDCl_3$)

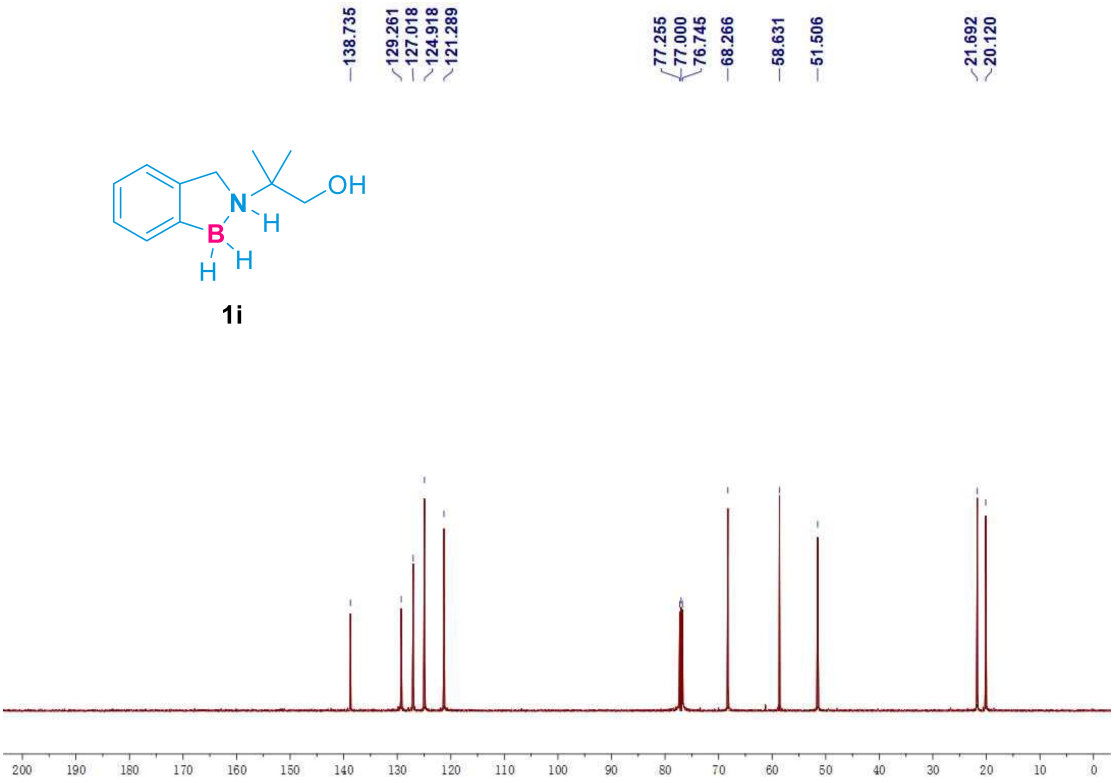

^{11}B NMR (160 MHz, $CDCl_3$)

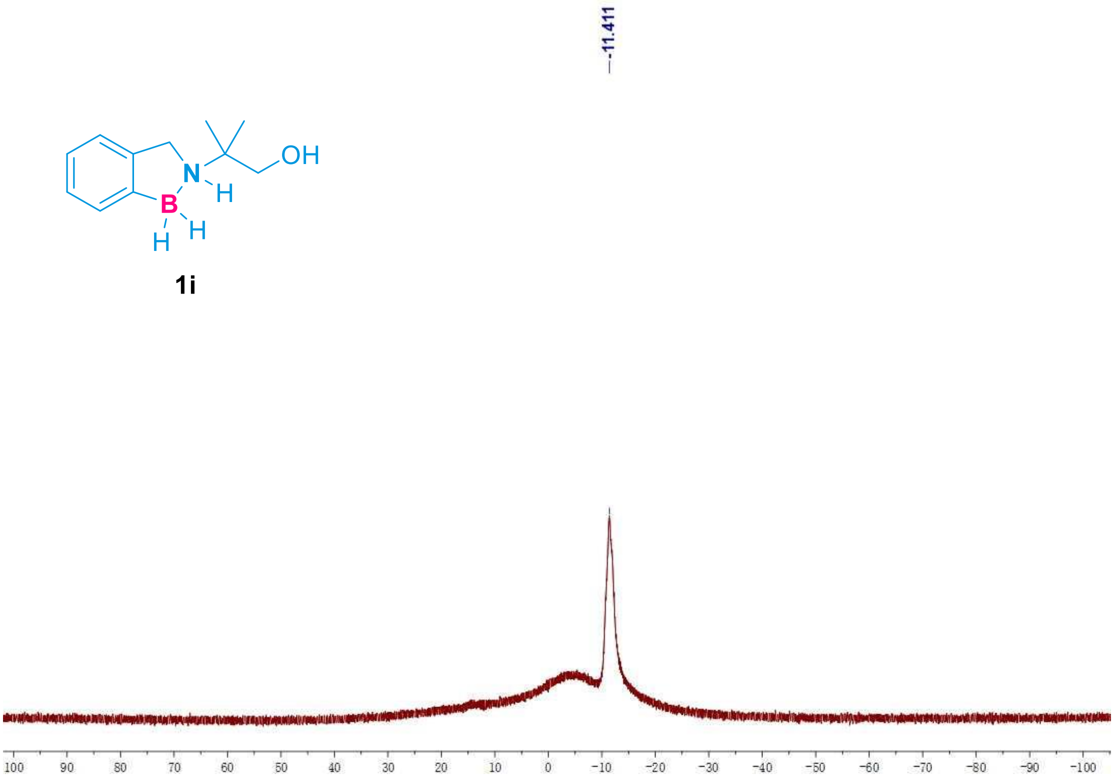

2-(3-methylbenzyl)-2,3-dihydro-1H-benzo[c][1,2]azaborole (1j)

^{1}H NMR (500 MHz, $CDCl_3$)

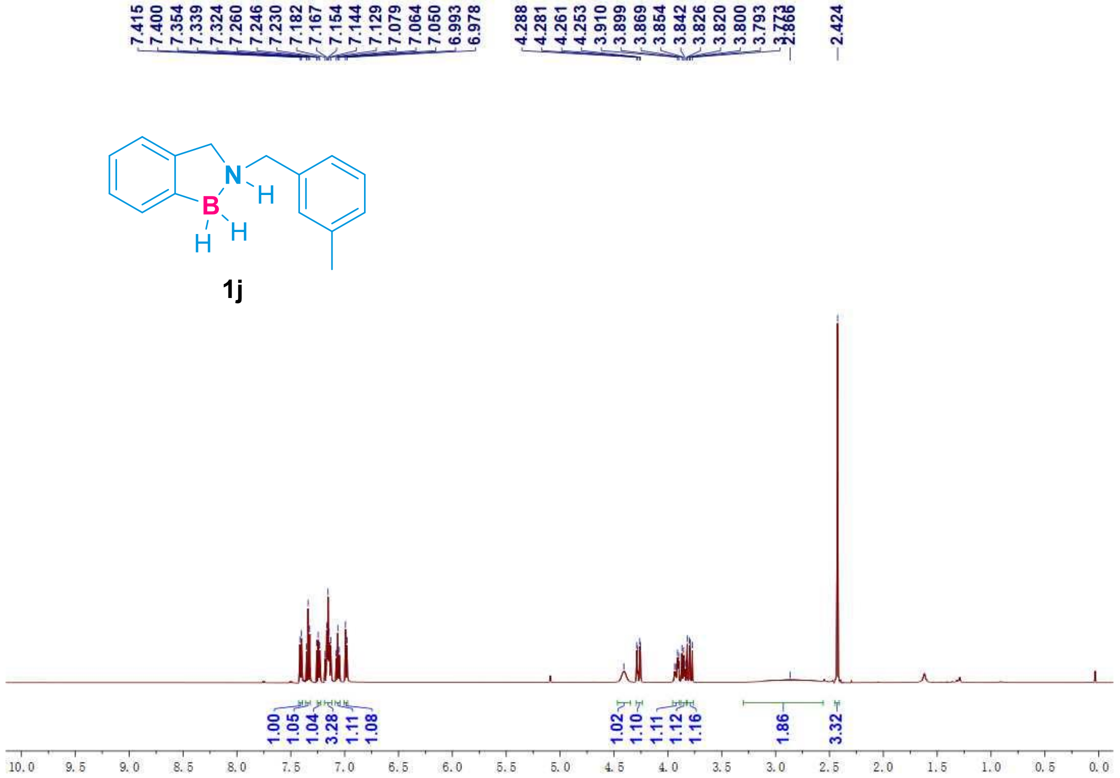

^{13}C NMR (126 MHz, $CDCl_3$)

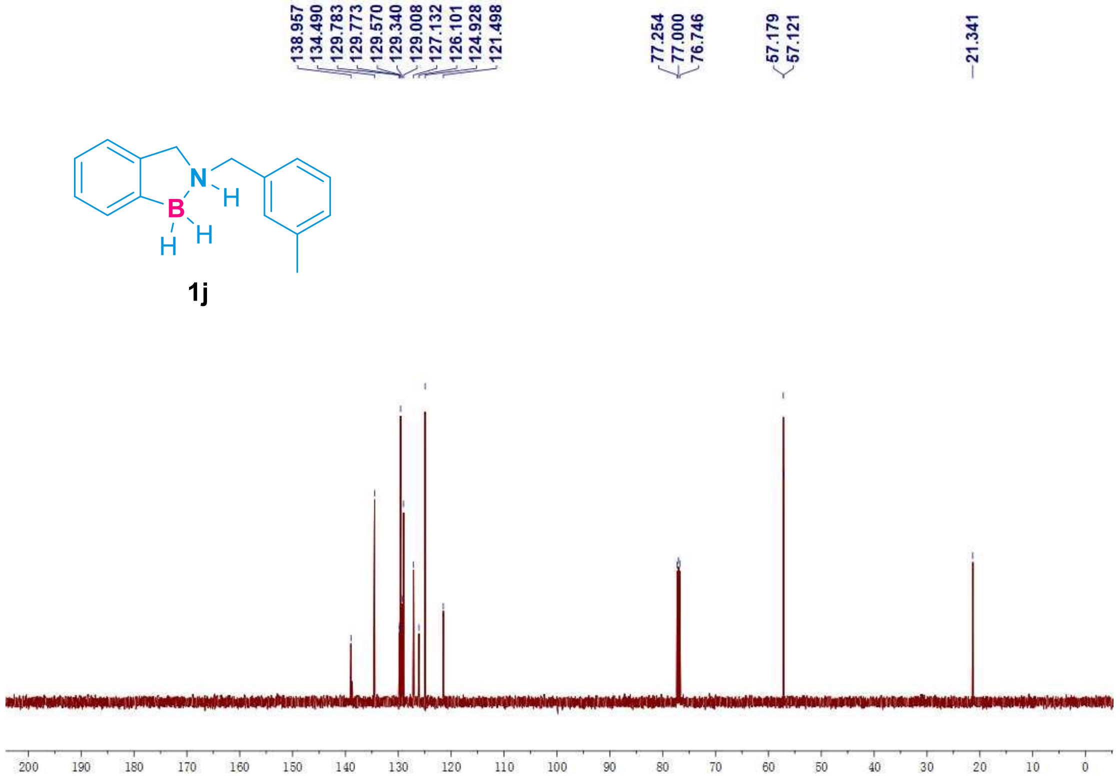

^{11}B NMR (160 MHz, $CDCl_3$)

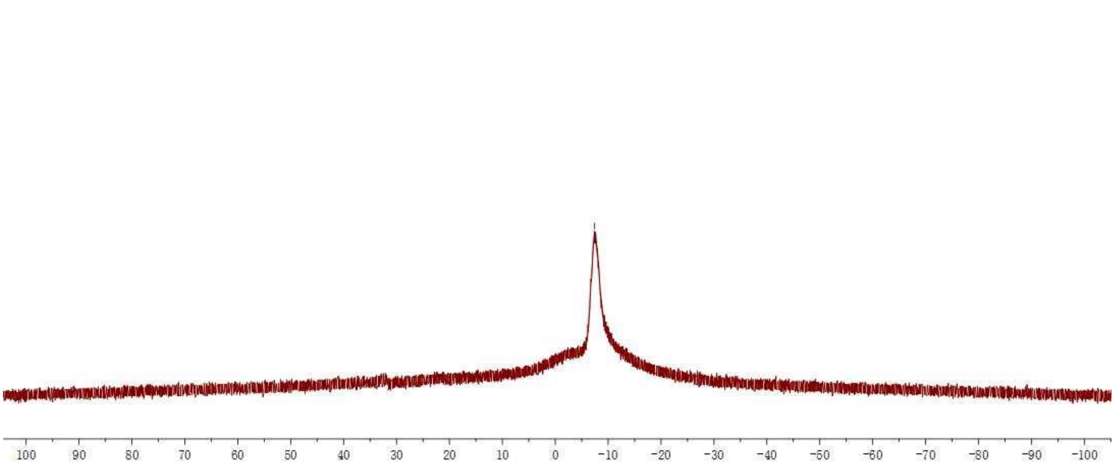

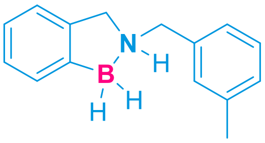

1j

2-(tert-butyl)-1,2,3,4-tetrahydrobenzo[c][1,2]azaborinine (**1k**)

^{1}H NMR (500 MHz, $CDCl_3$)

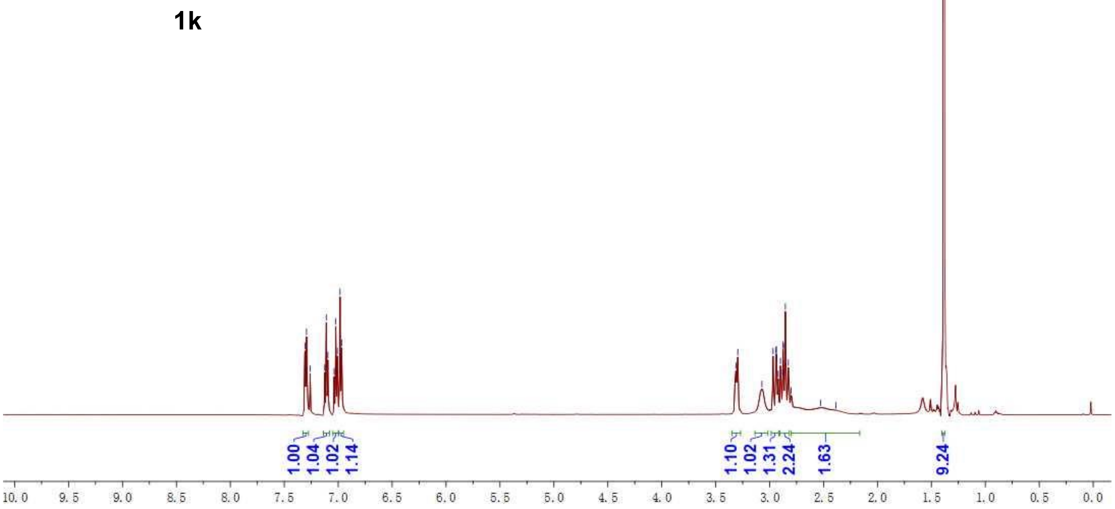

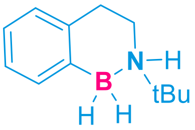

1k

^{13}C NMR (126 MHz, $CDCl_3$)

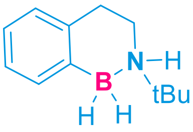

1k

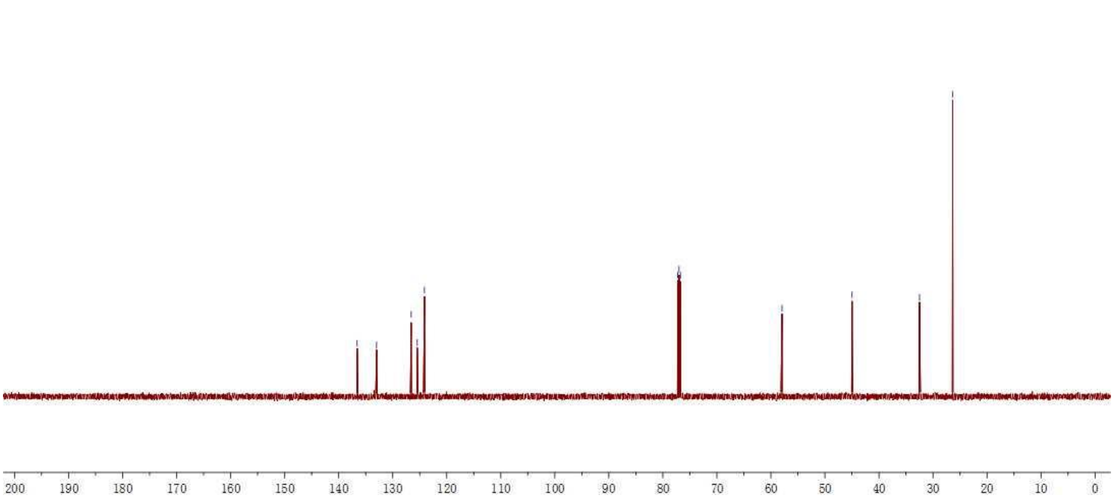

^{11}B NMR (400 MHz, $CDCl_3$)

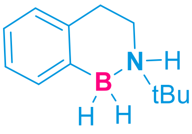

1k

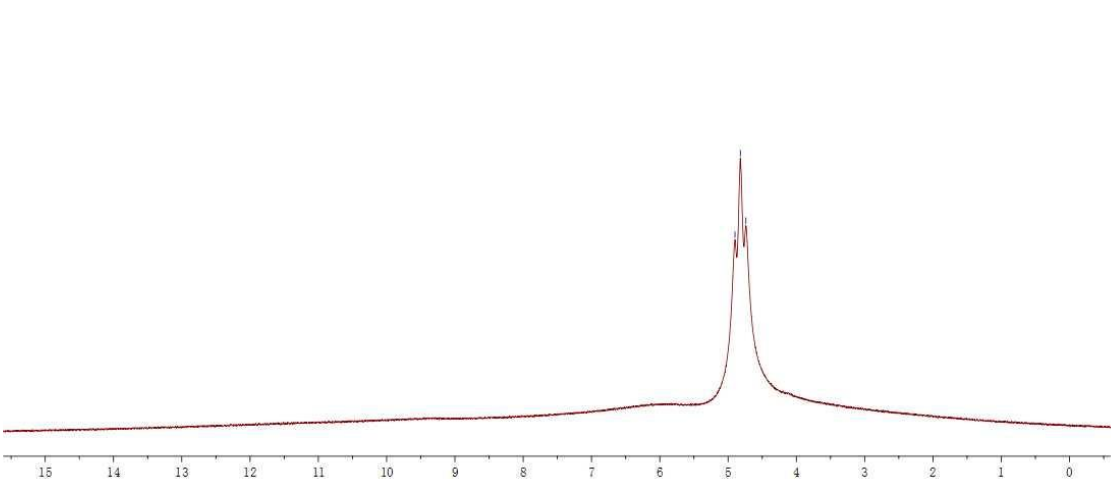

2-(tert-butyl)-5,6-dimethoxy-2,3-dihydro-1H-1λ^{4},2λ^{4}-benzo[c][1,2]azaborole (1l)

^{1}H NMR (500 MHz, $CDCl_3$)

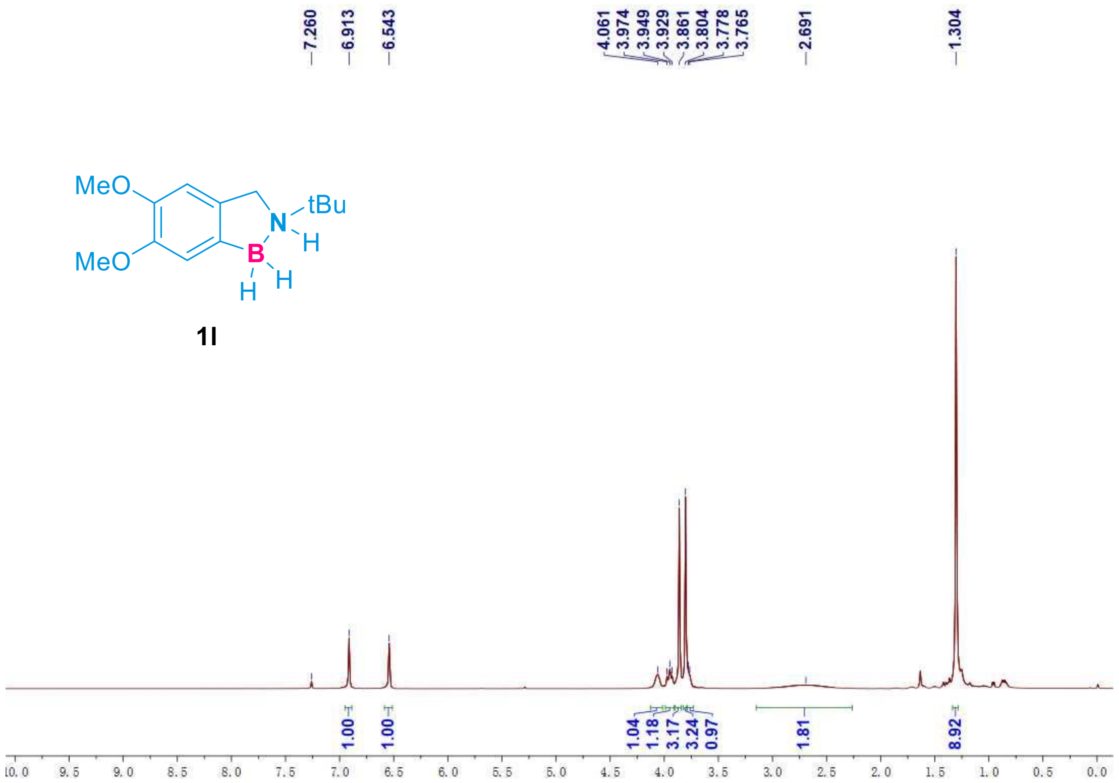

^{13}C NMR (126 MHz, $CDCl_3$)

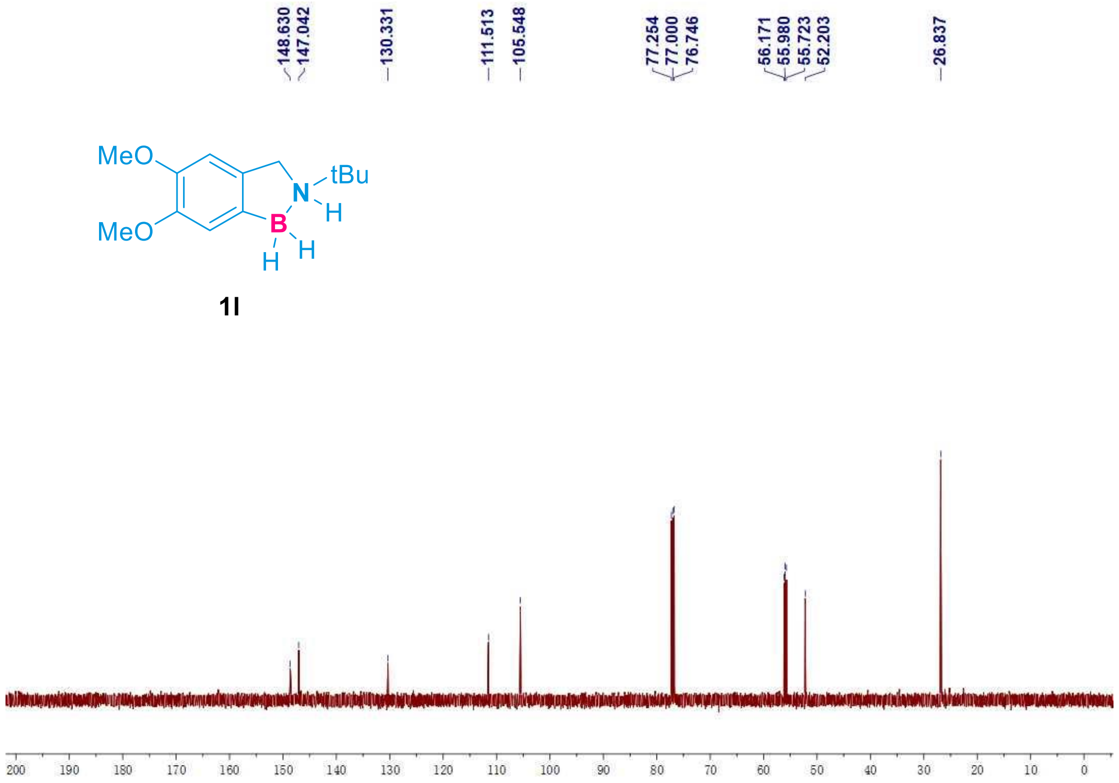

^{11}B NMR (160 MHz, $CDCl_3$)

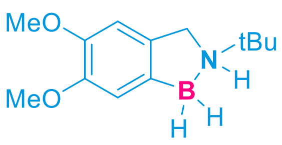

1l

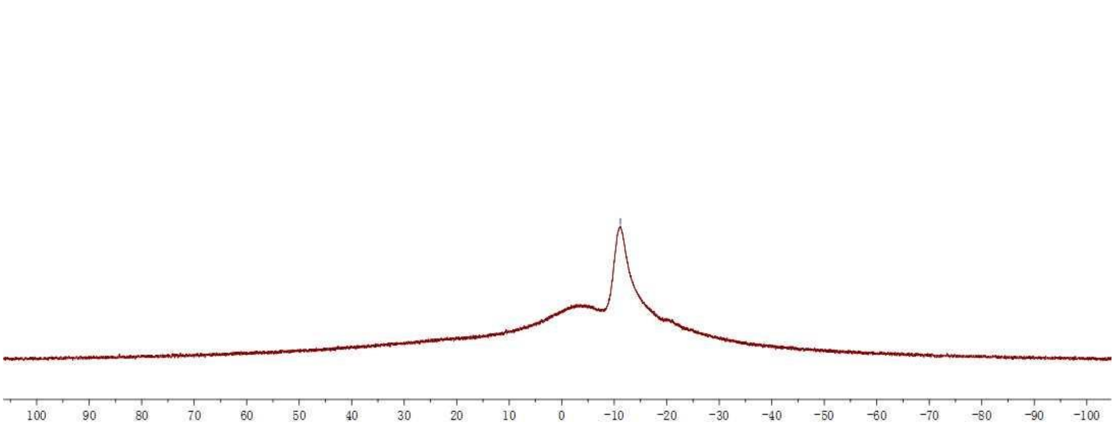

2-(tert-butyl)-6-methoxy-2,3-dihydro-1H-1λ^4,2λ^4-benzo[c][1,2]azaborole (1m)

^{1}H NMR (500 MHz, $CDCl_3$)

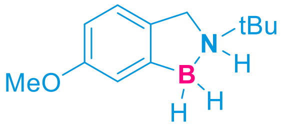

1m

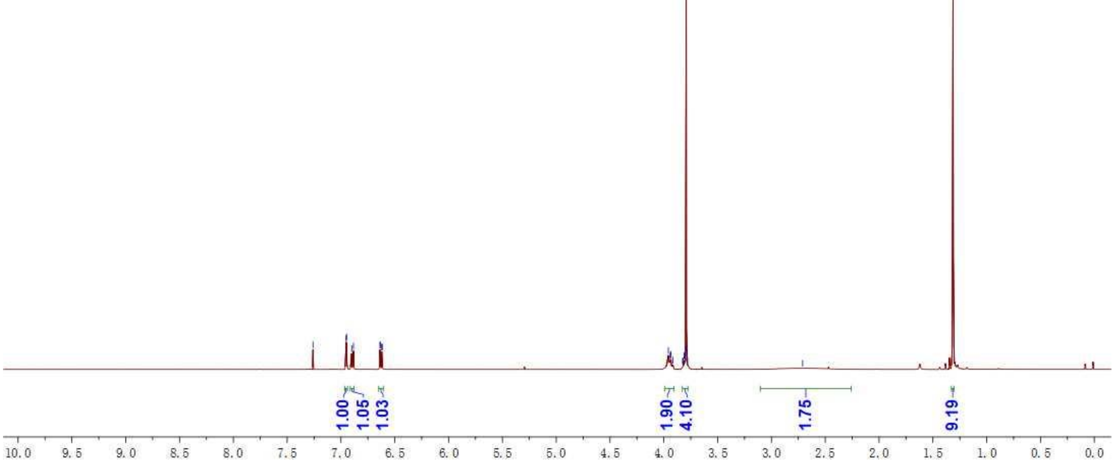

^{13}C NMR (126 MHz, $CDCl_3$)

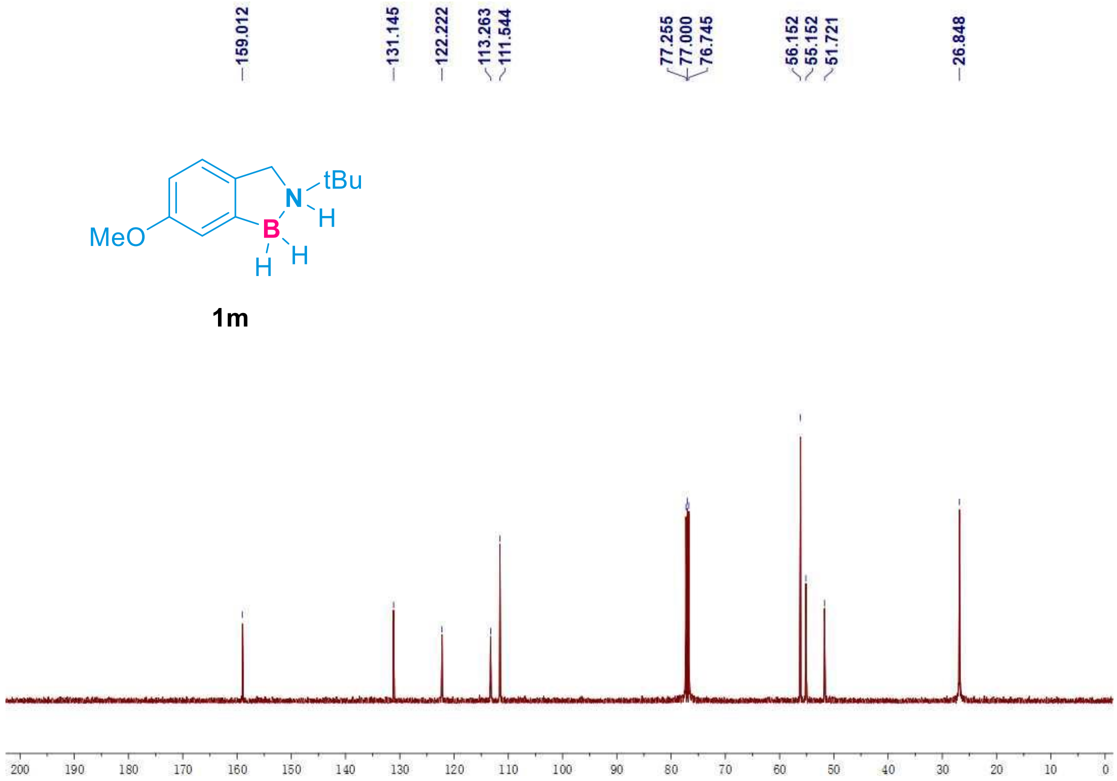

^{11}B NMR (128 MHz, $CDCl_3$)

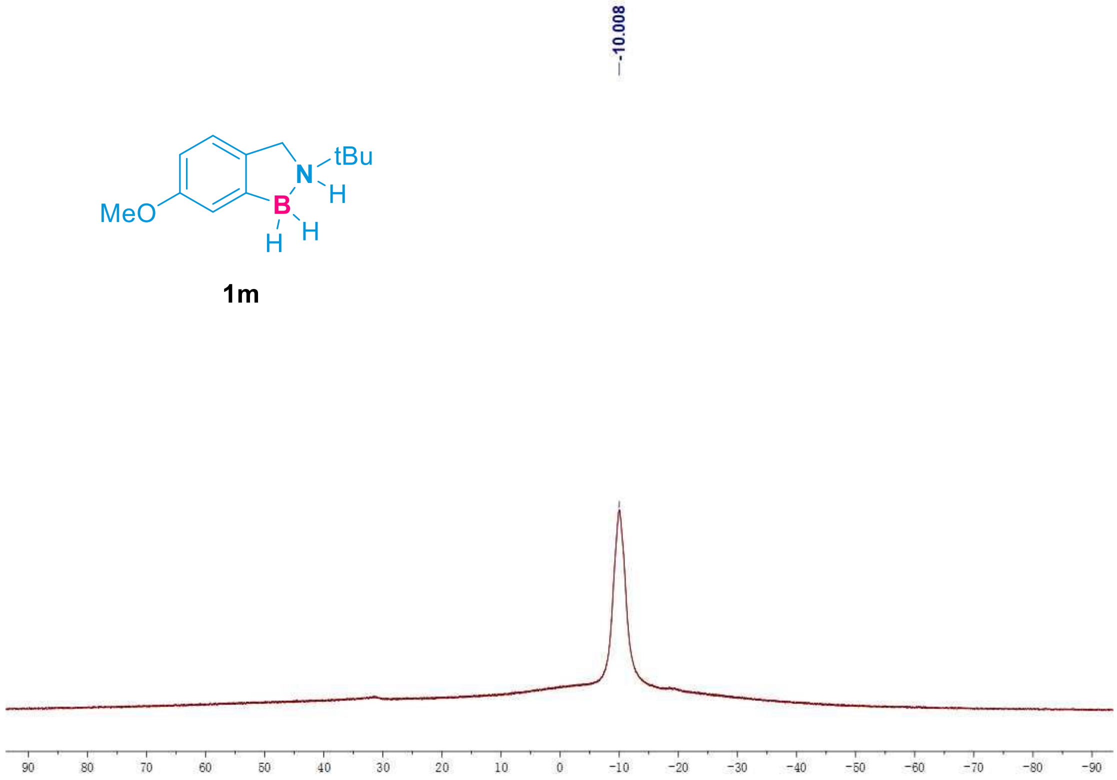

2-(tert-butyl)-5-fluoro-2,3-dihydro-1H-1λ[4],2λ[4]-benzo[c][1,2]azaborole (1n)

^{1}H NMR (500 MHz, $CDCl_3$)

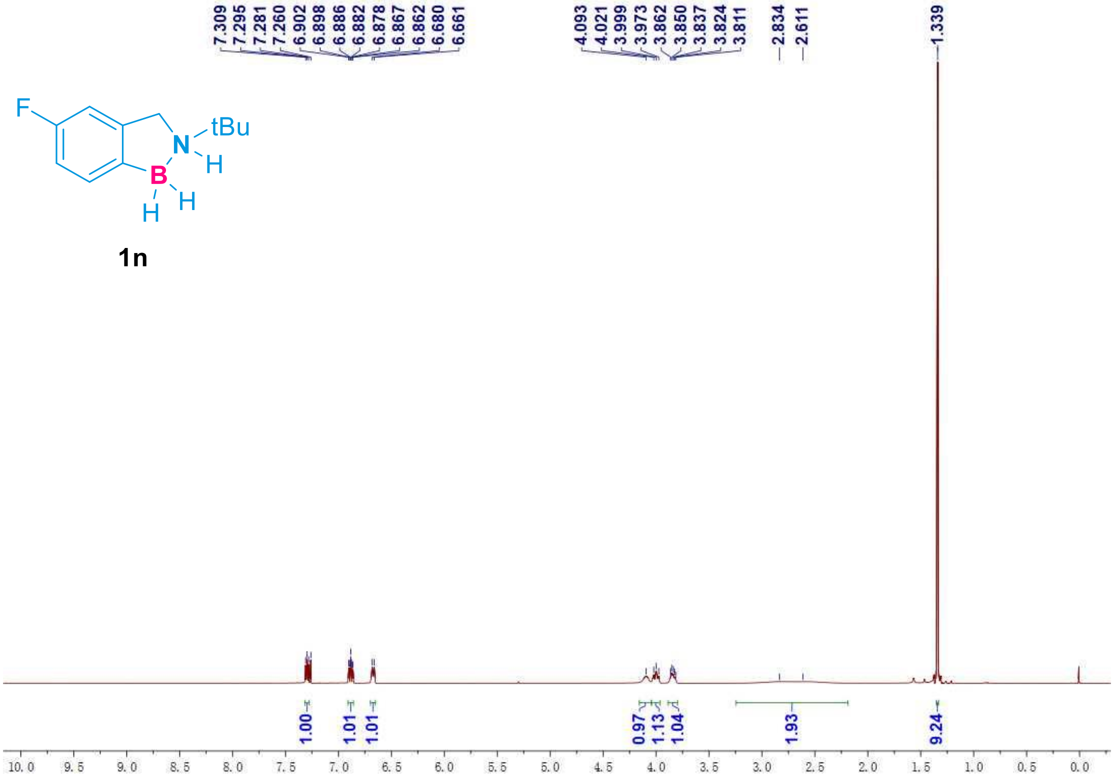

^{13}C NMR (101 MHz, $CDCl_3$)

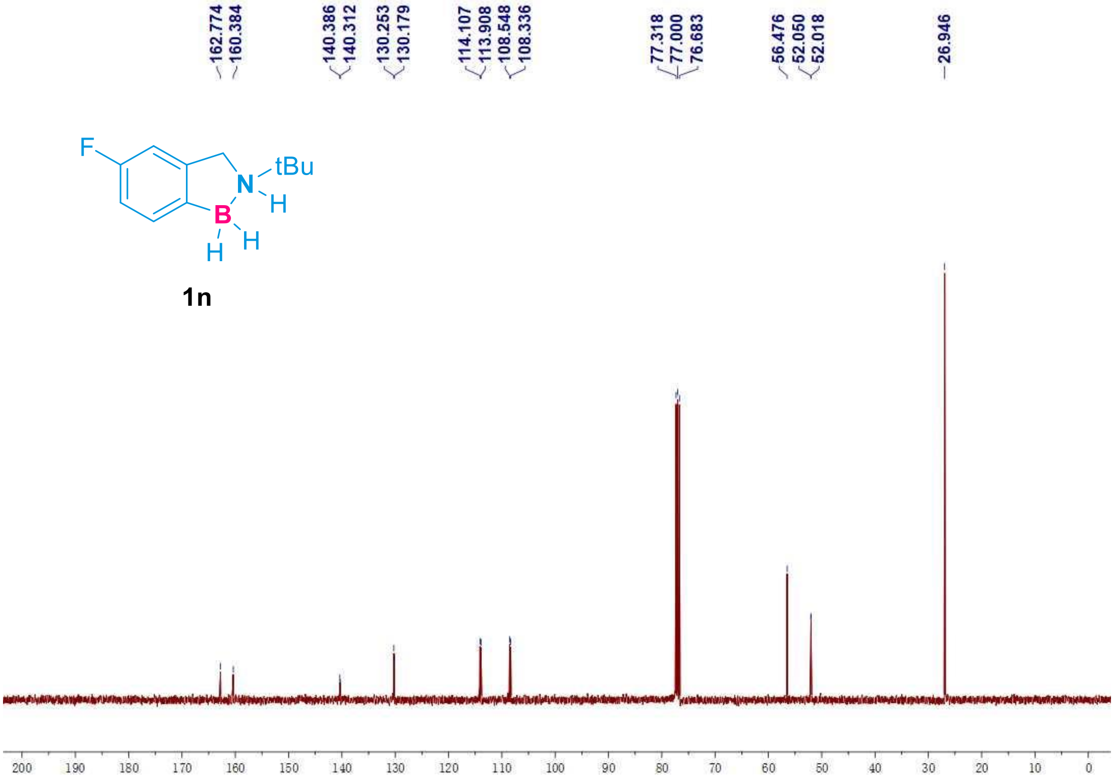

^{19}F NMR (471 MHz, $CDCl_3$)

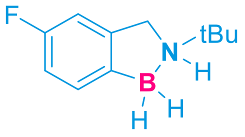

1n

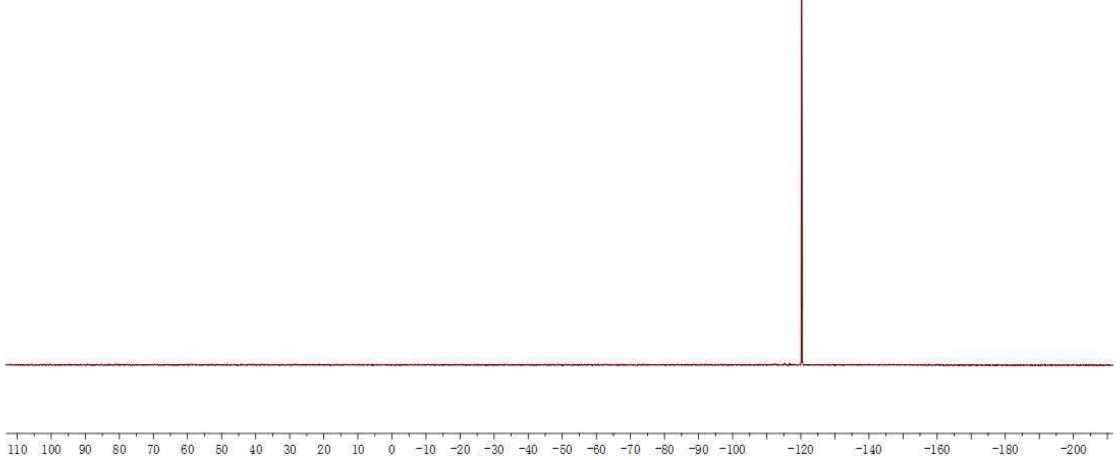

^{11}B NMR (128 MHz, $CDCl_3$)

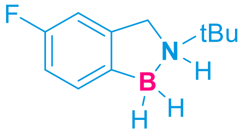

1n

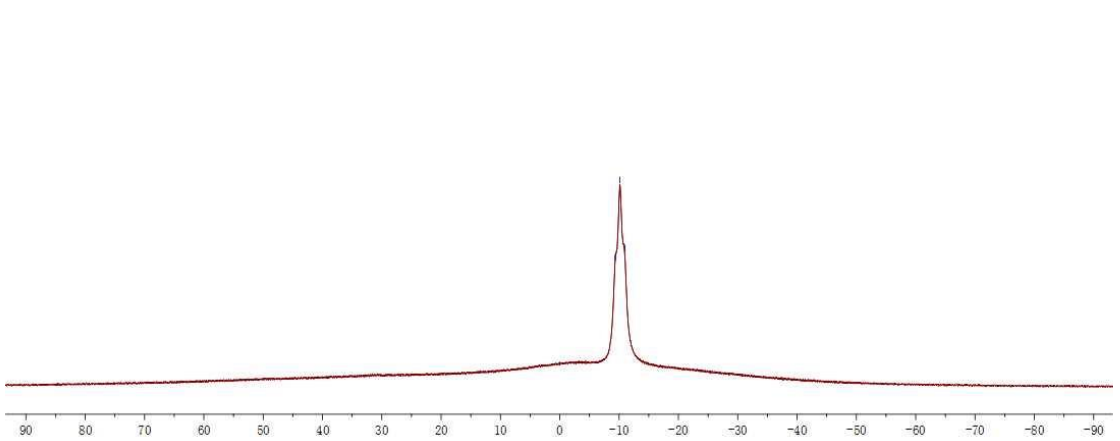

2-(tert-butyl)-2,3-dihydro-1H-1λ[4]-benzo[c][1,2]azaborole-1,1,3-d3 (1a-*d₃*)

^{1}H NMR (400 MHz, $CDCl_3$)

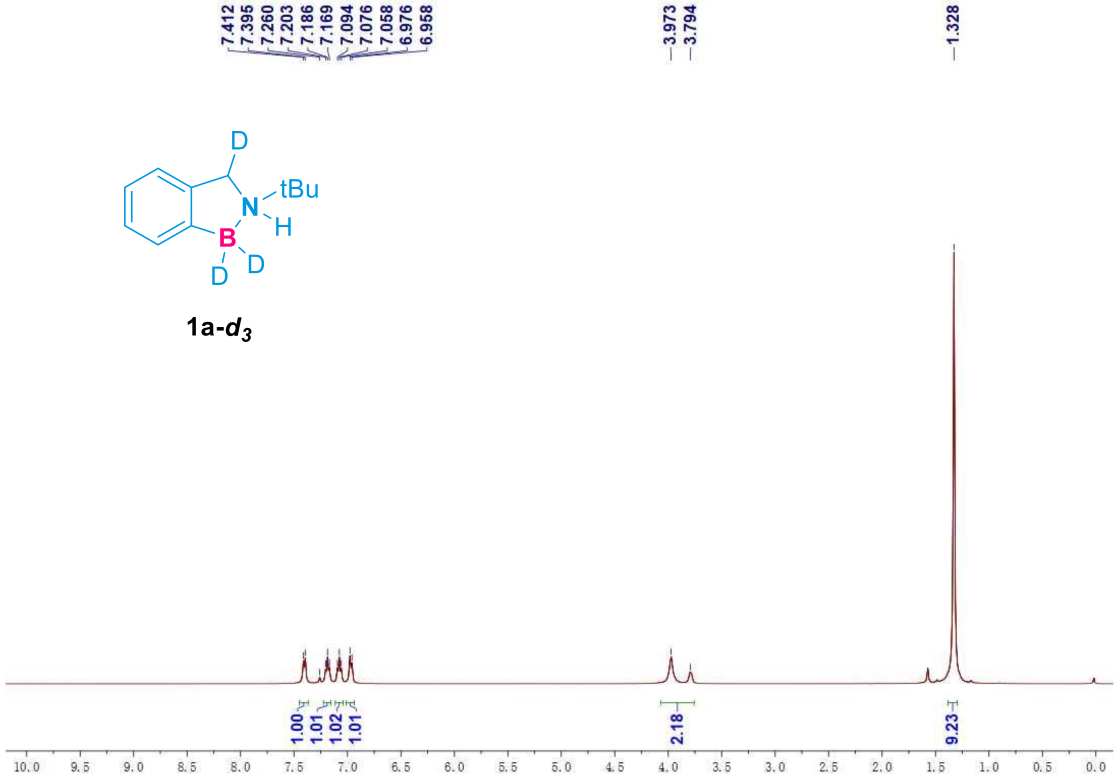

^{13}C NMR (101 MHz, $CDCl_3$)

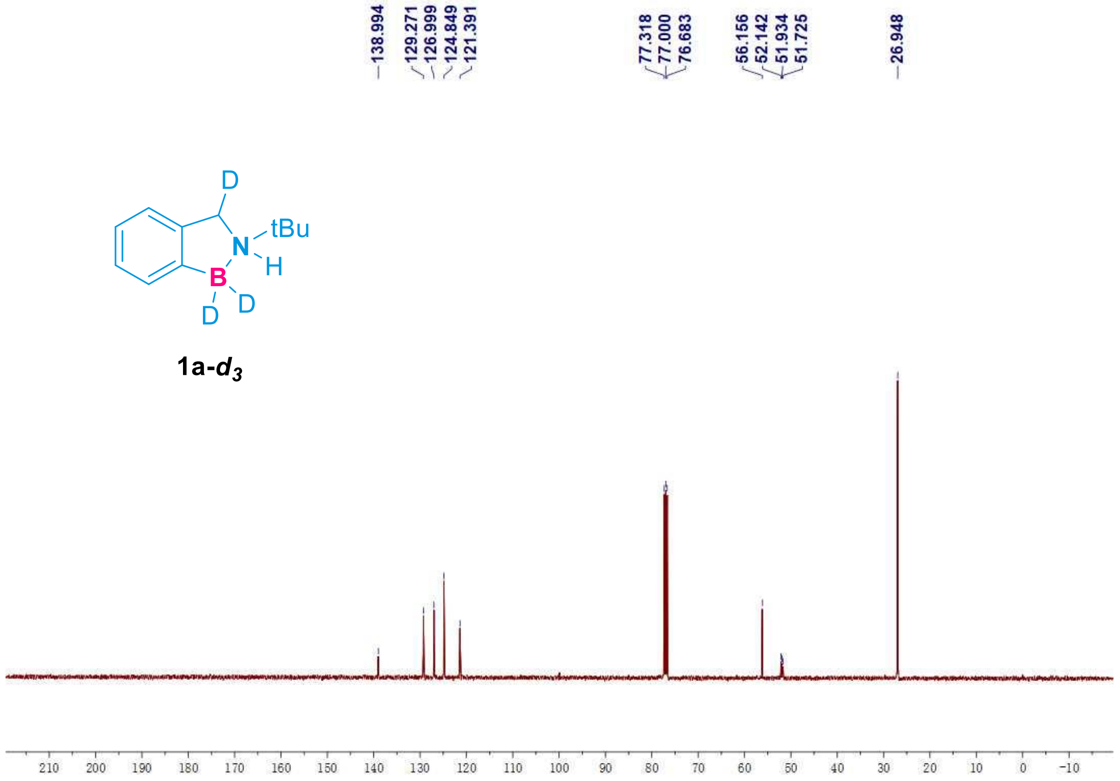

^{11}B NMR (128 MHz, $CDCl_3$)

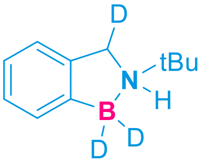

1a-*d*[3]

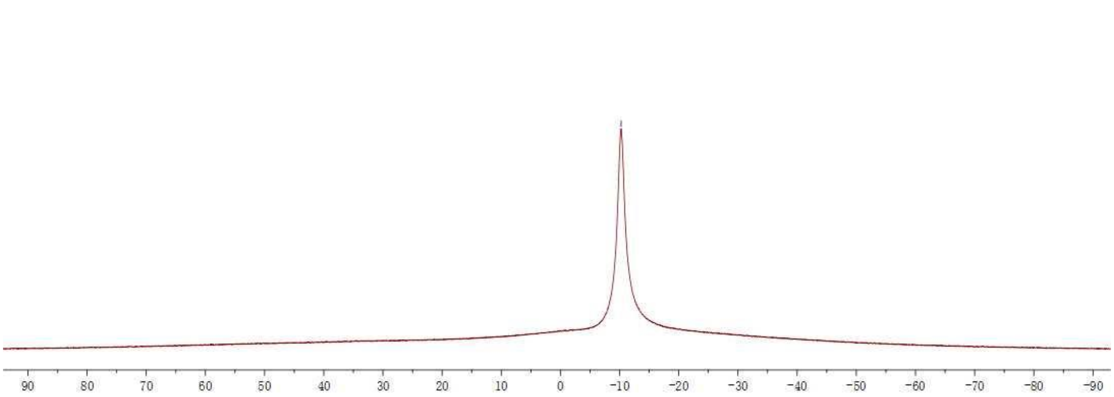

(diazomethylene)dibenzene (2a)

^{1}H NMR (500 MHz, $CDCl_3$)

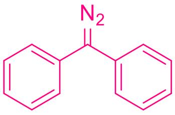

2a

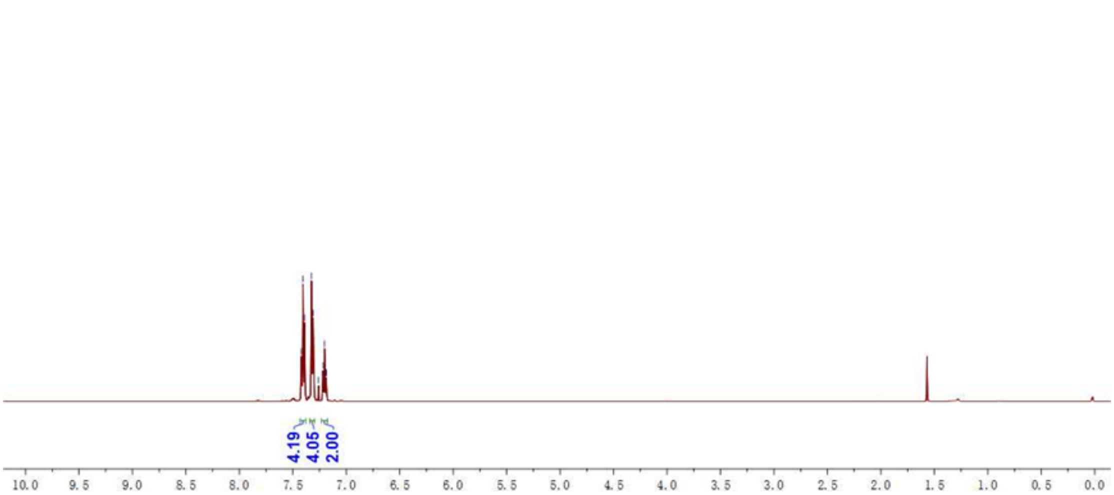

4,4'-(diazomethylene)bis(methoxybenzene)(2b)

^{1}H NMR (500 MHz, CDCl3)

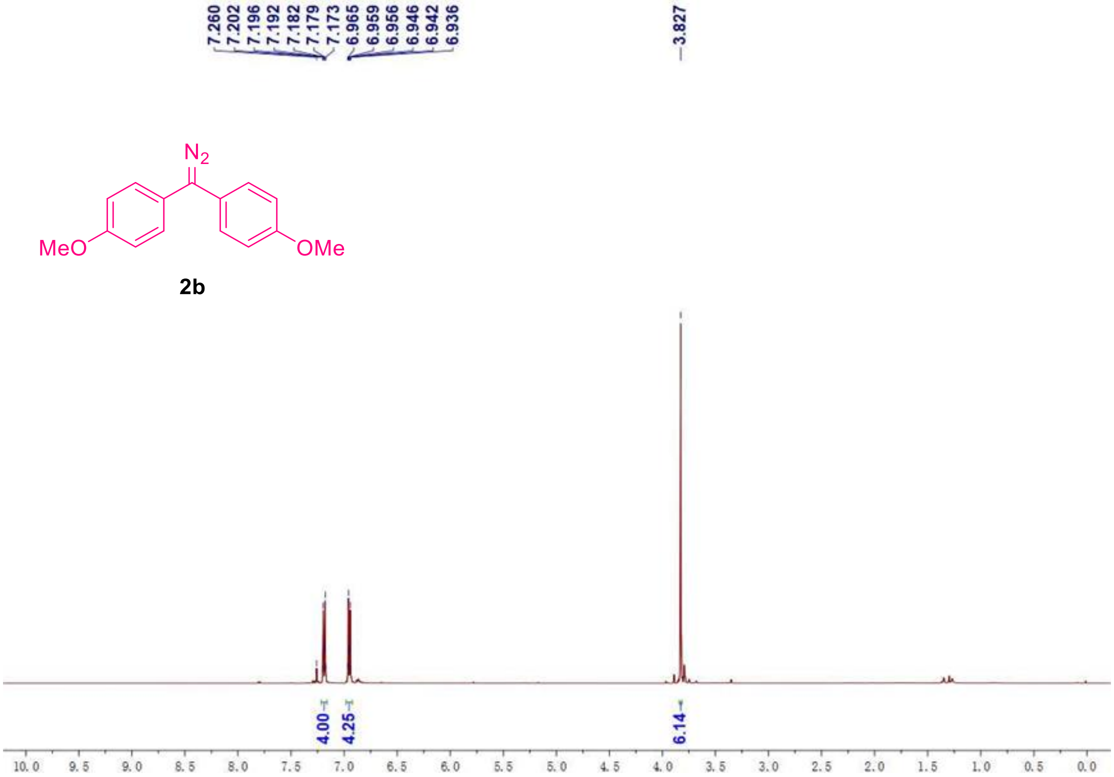

2b

4,4'-(diazomethylene)bis(fluorobenzene) (2d)

^{1}H NMR (500 MHz, CDCl3)

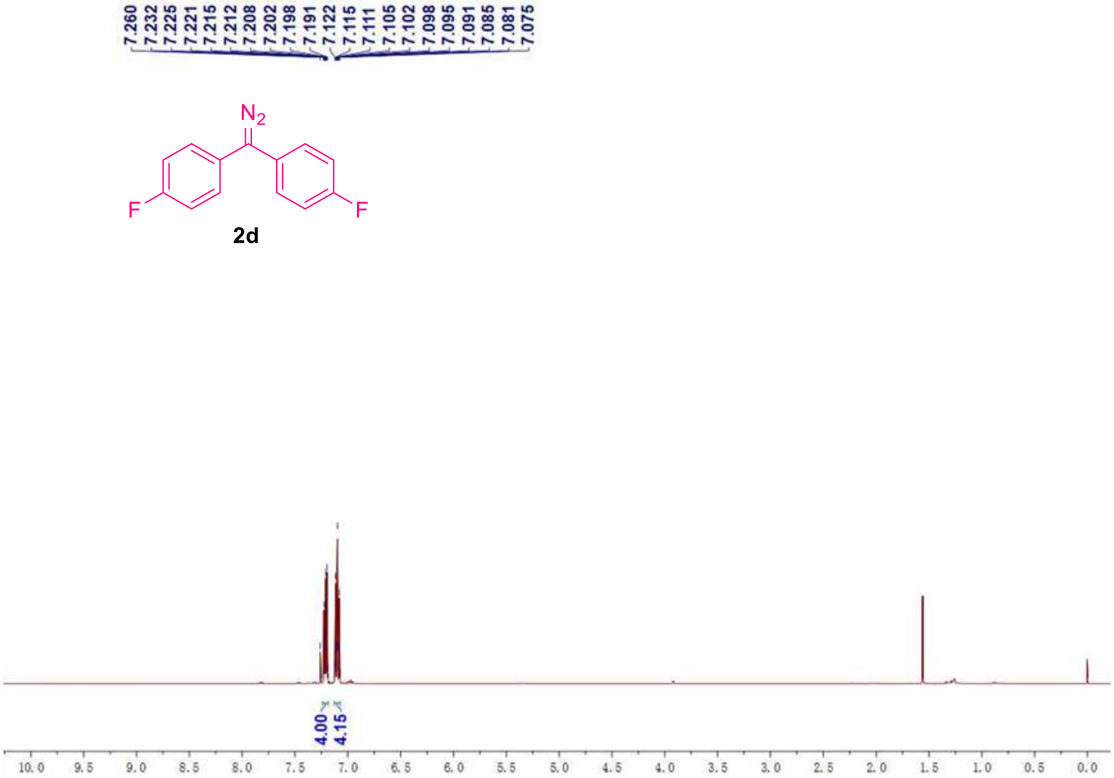

2d

3,3'-(diazomethylene)bis(methoxybenzene) (2e)

^{1}H NMR (500 MHz, $CDCl_3$)

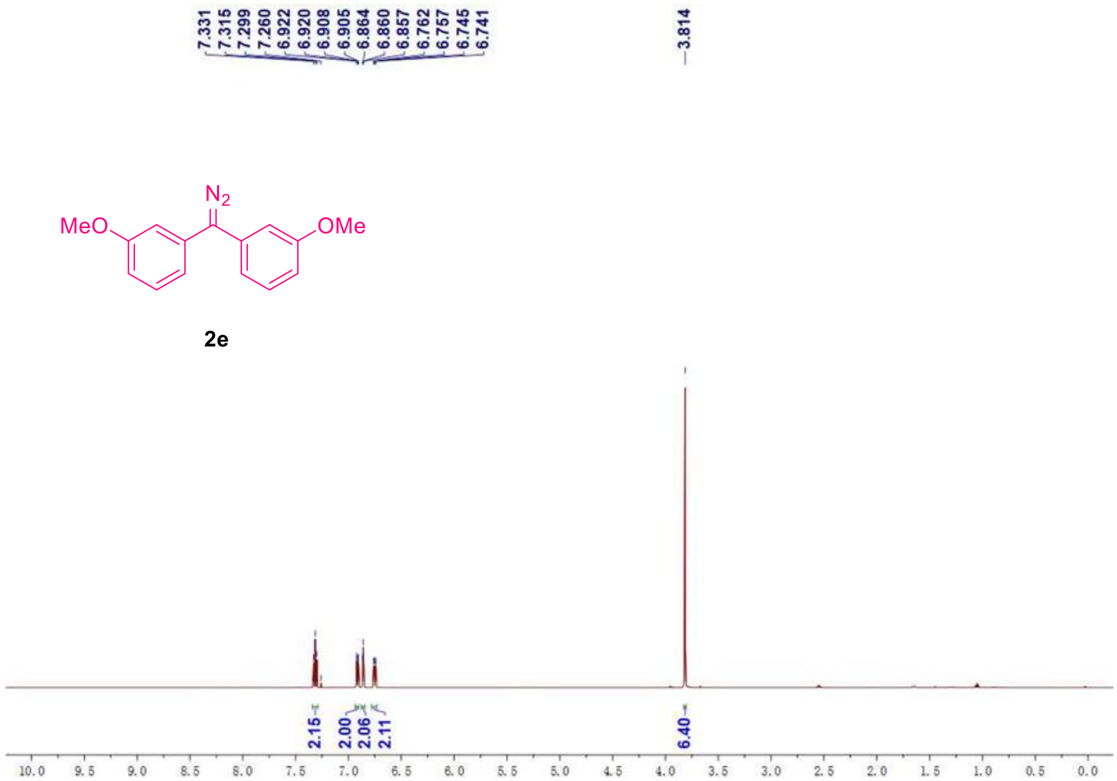

^{13}C NMR (126 MHz, $CDCl_3$)

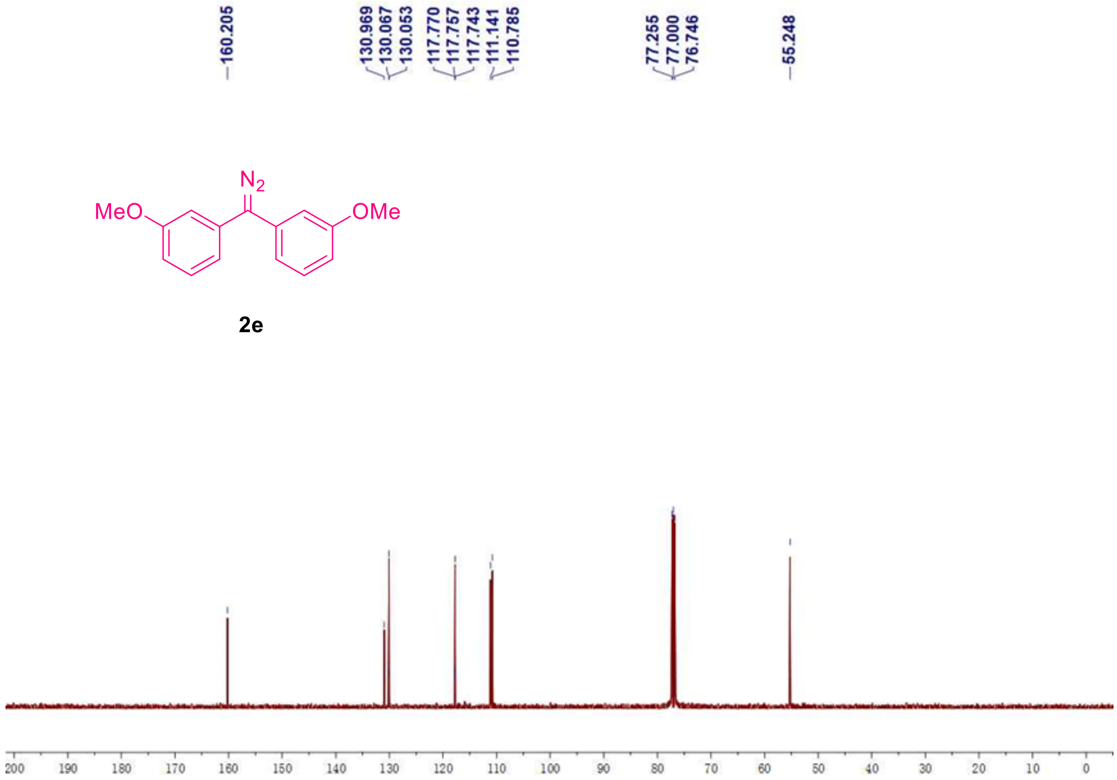

3,3'-(diazomethylene)bis(methylbenzene)(2f)

^{1}H NMR (500 MHz, $CDCl_3$)

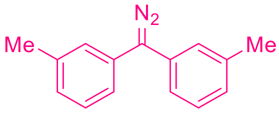

2f

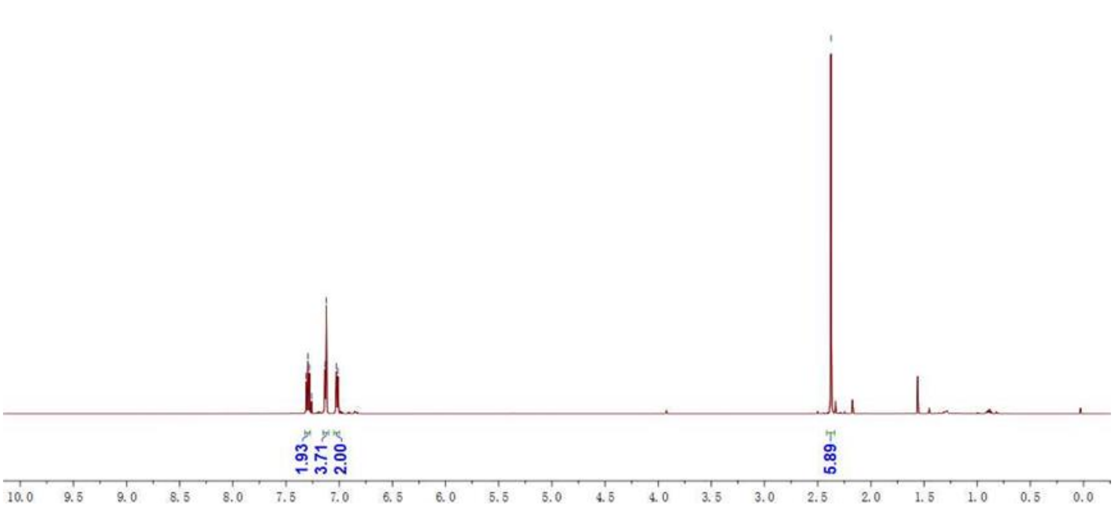

^{13}C NMR (126 MHz, $CDCl_3$)

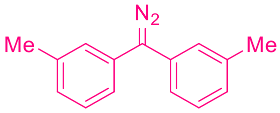

2f

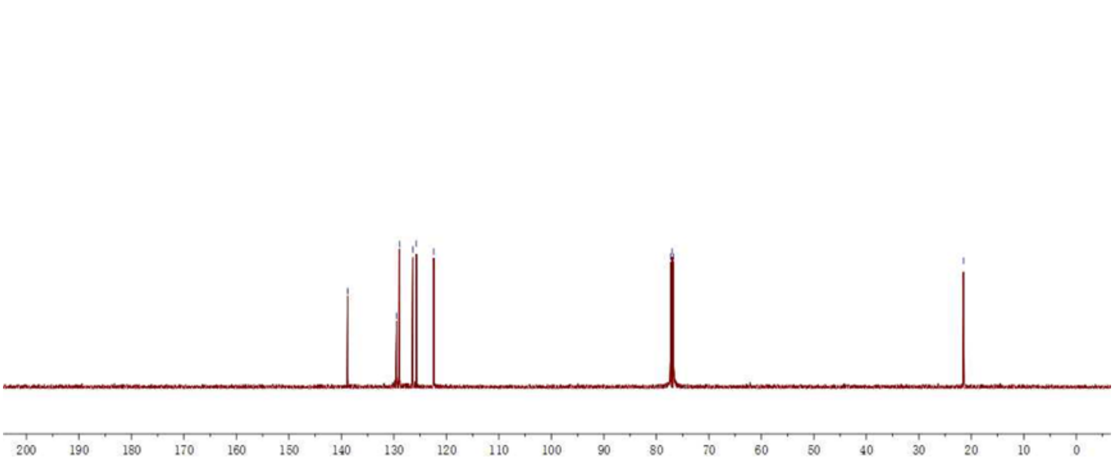

5,5'-(diazomethylene)bis(1,3-dimethoxybenzene) (2g)

^{1}H NMR (400 MHz, $CDCl_3$)

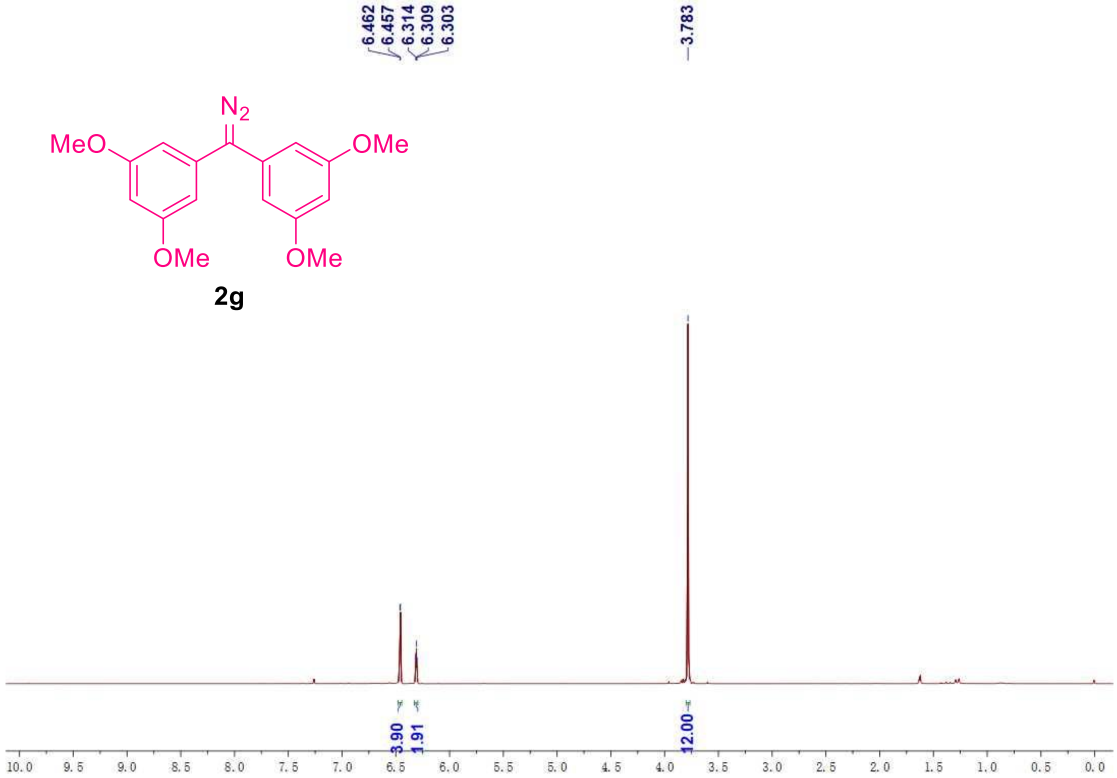

^{13}C NMR (126 MHz, $CDCl_3$)

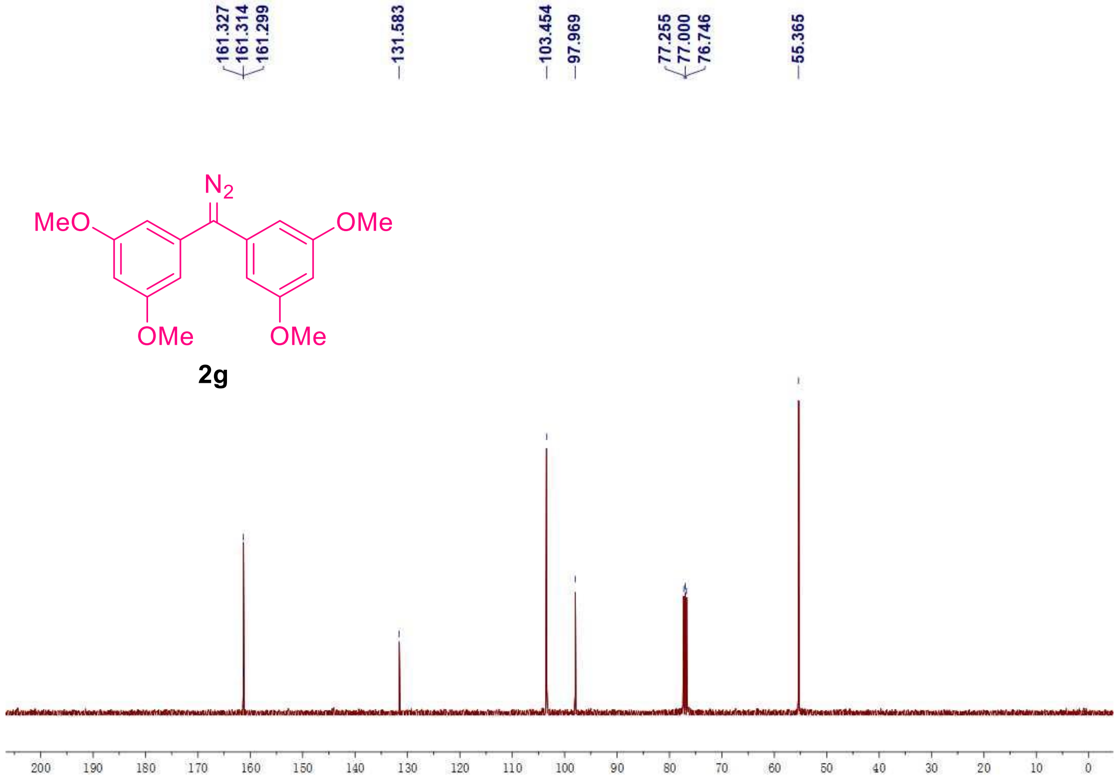

4-(diazo(phenyl)methyl)benzonitrile (2h)

^{1}H NMR (500 MHz, $CDCl_3$)

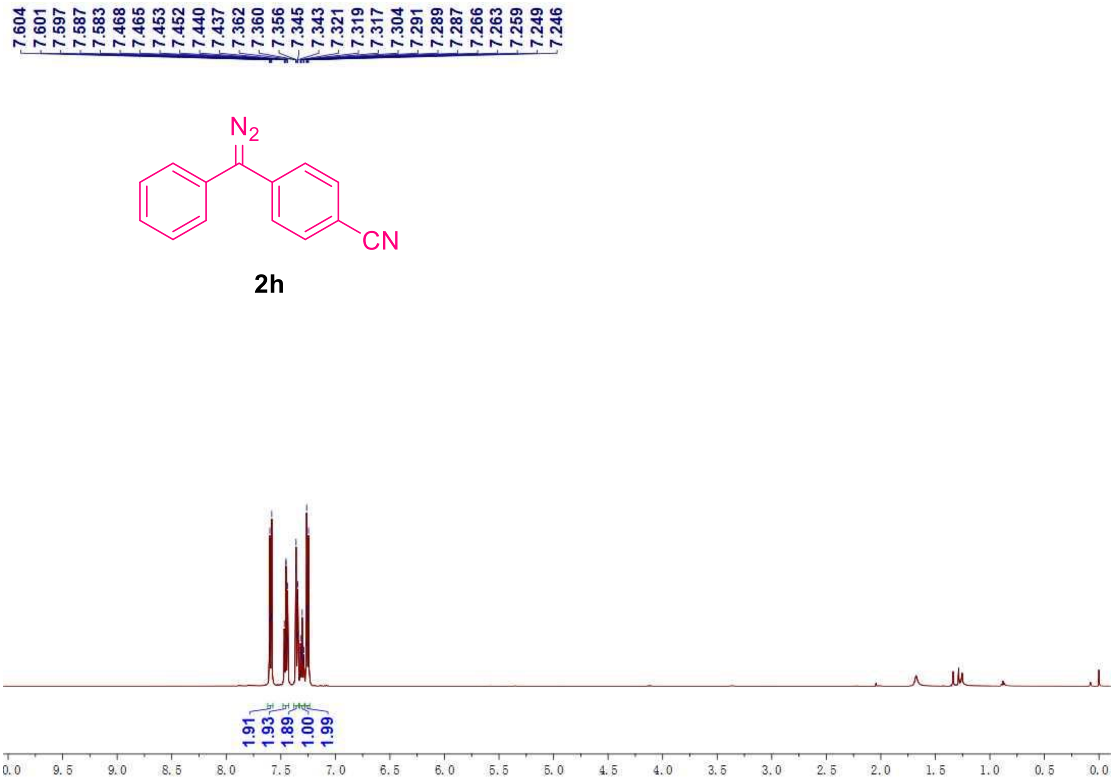

^{13}C NMR (126 MHz, $CDCl_3$)

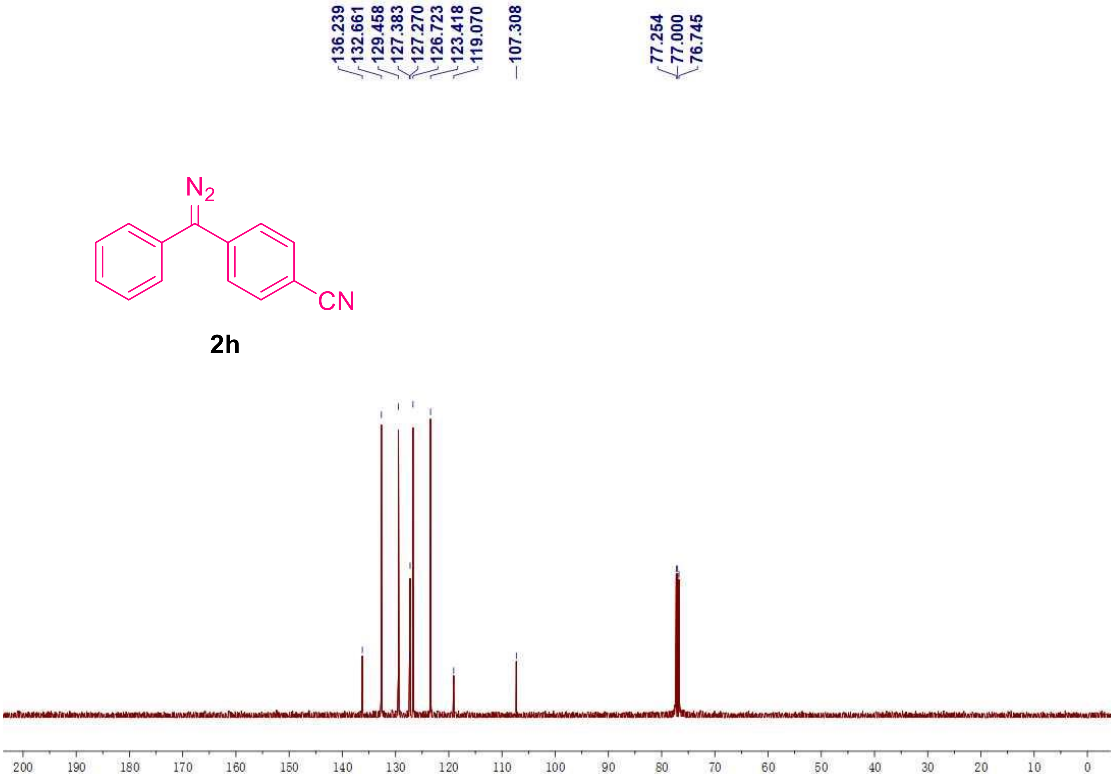

1-(diazo(phenyl)methyl)-4-nitrobenzene (**2i**)

[1]**H NMR** (500 MHz, $CDCl_3$)

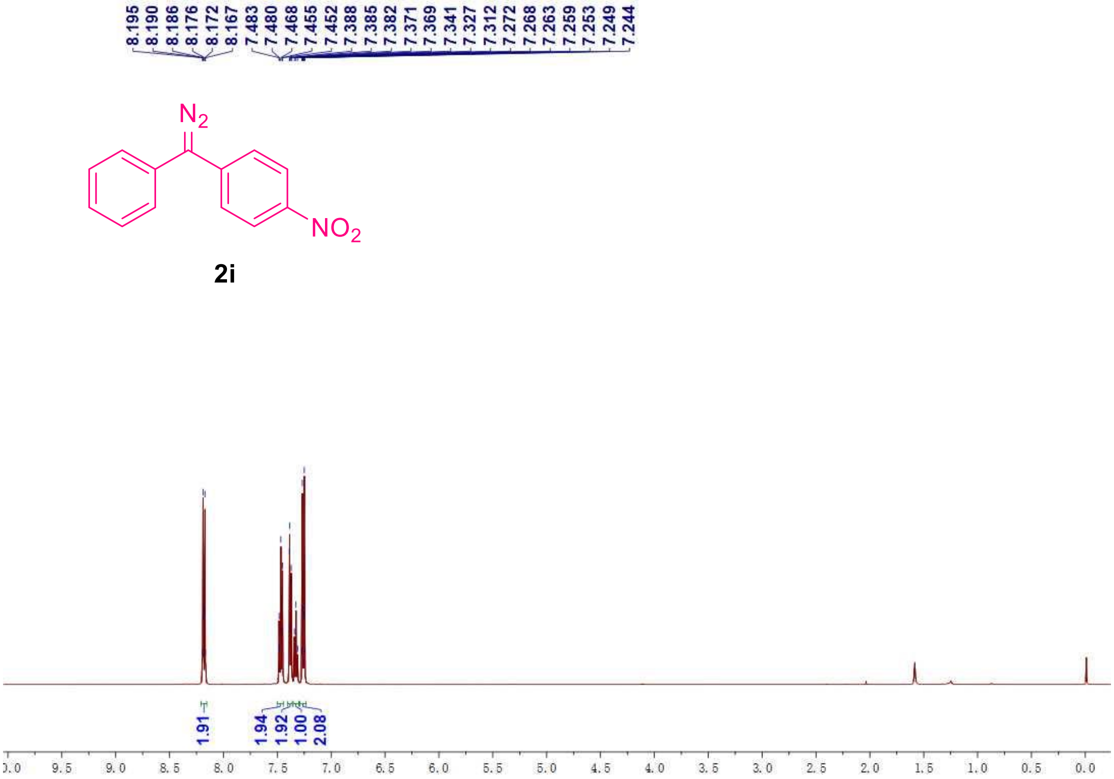

[13]**C NMR** (126 MHz, $CDCl_3$)

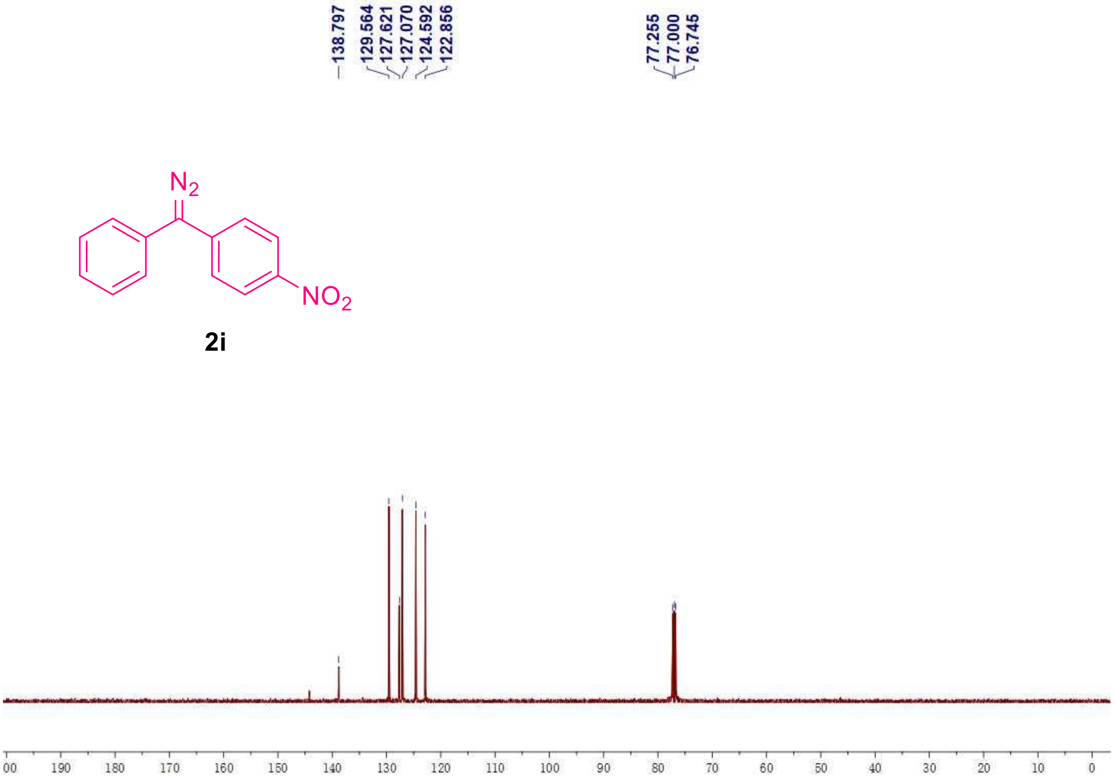

1-(diazo(phenyl)methyl)-4-methoxybenzene (2j)

^{1}H NMR (500 MHz, $CDCl_3$)

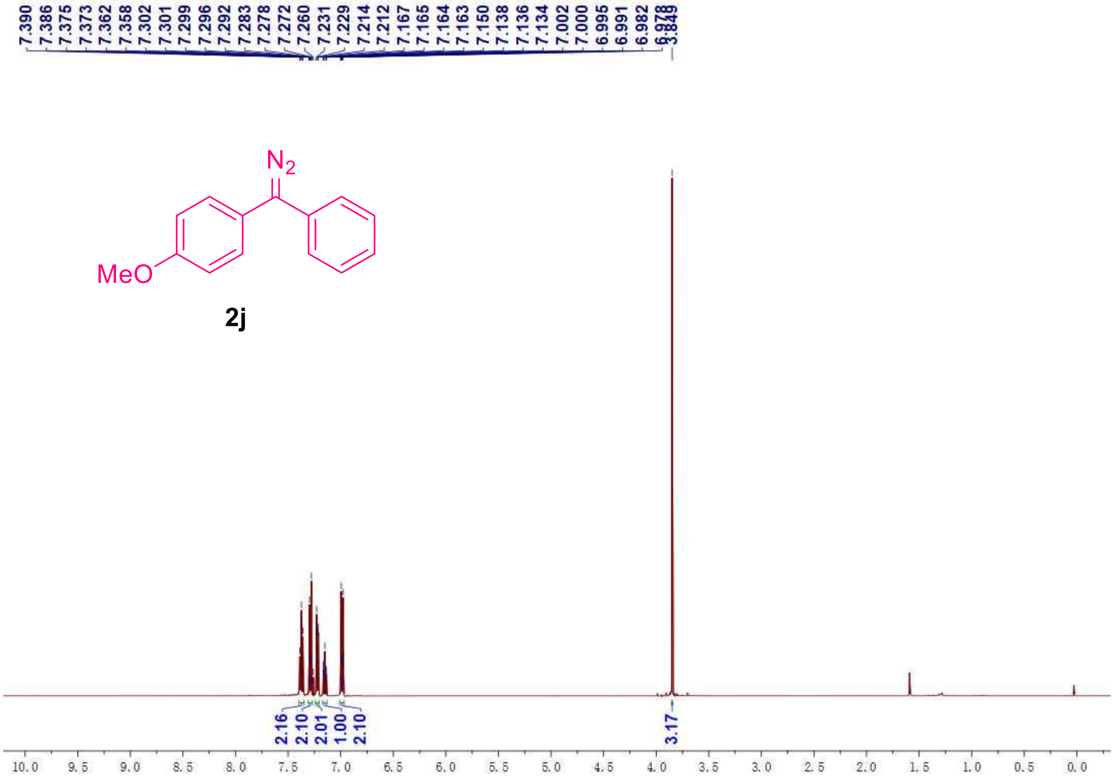

^{13}C NMR (126 MHz, $CDCl_3$)

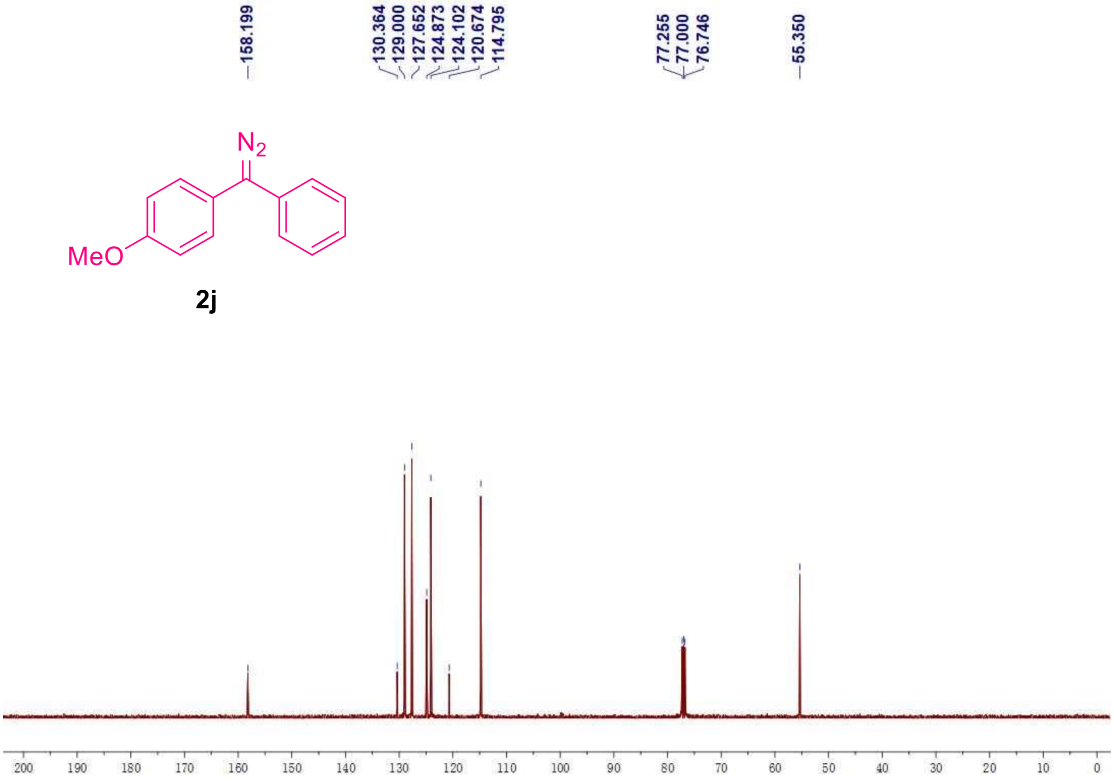

2-chloro-4-(diazo(4-methoxyphenyl)methyl)-1-fluorobenzene (2k)

^{1}H NMR (500 MHz, $CDCl_3$)

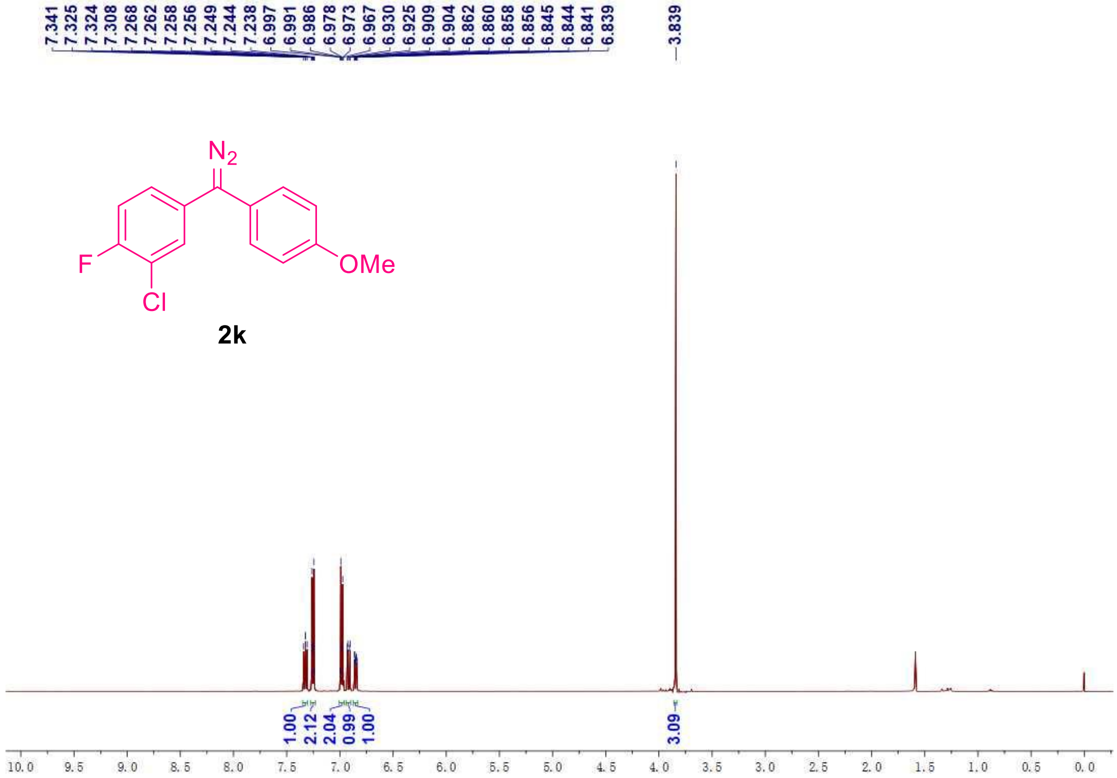

^{13}C NMR (126 MHz, $CDCl_3$)

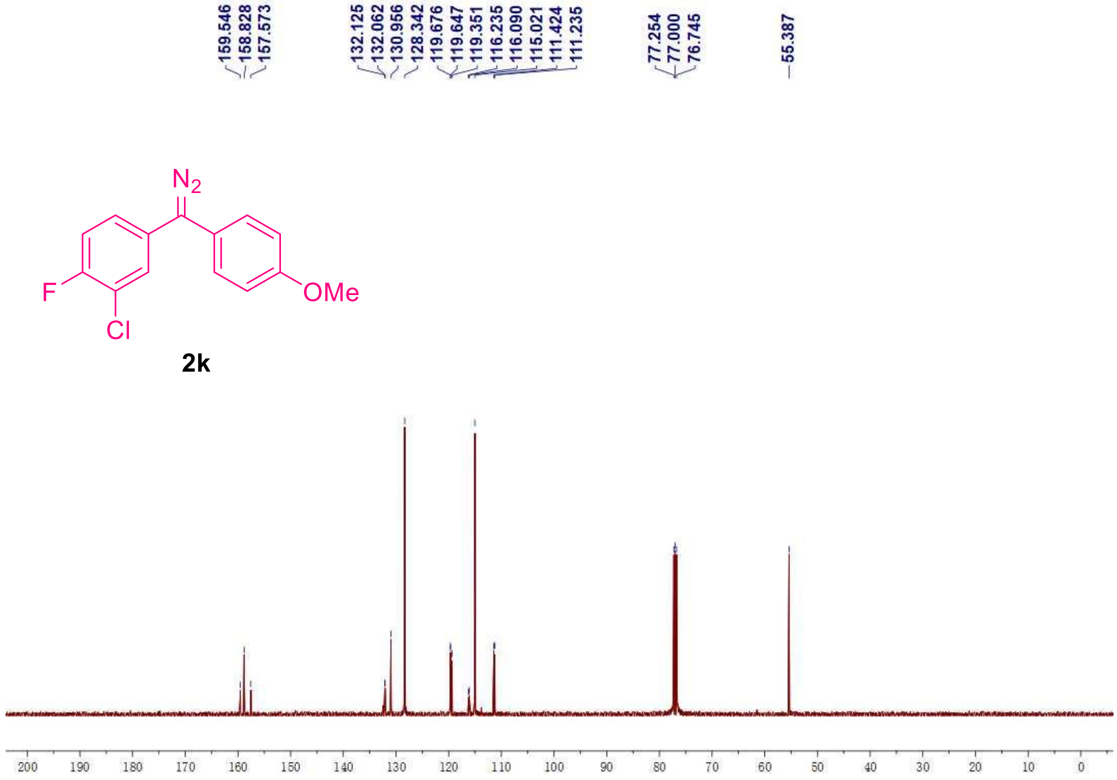

^{19}F NMR (471 MHz, $CDCl_3$)

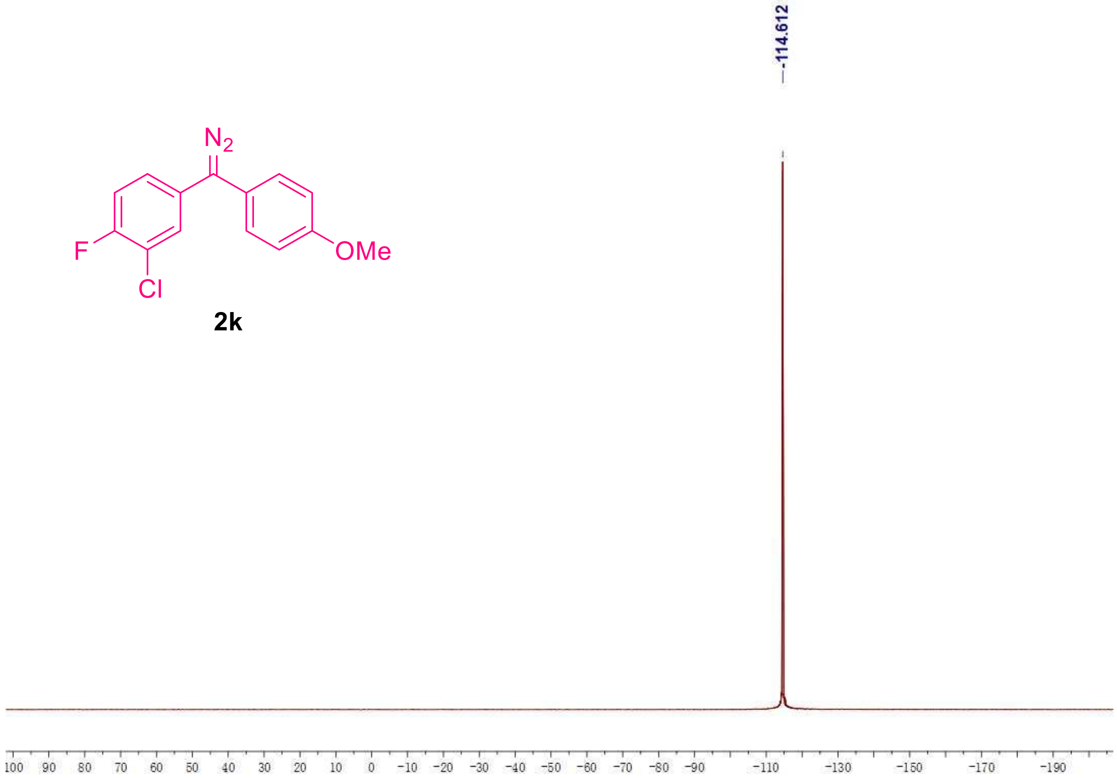

1,2-dichloro-4-(diazo(4-methoxyphenyl)methyl)benzene (2l)

^{1}H NMR (500 MHz, $CDCl_3$)

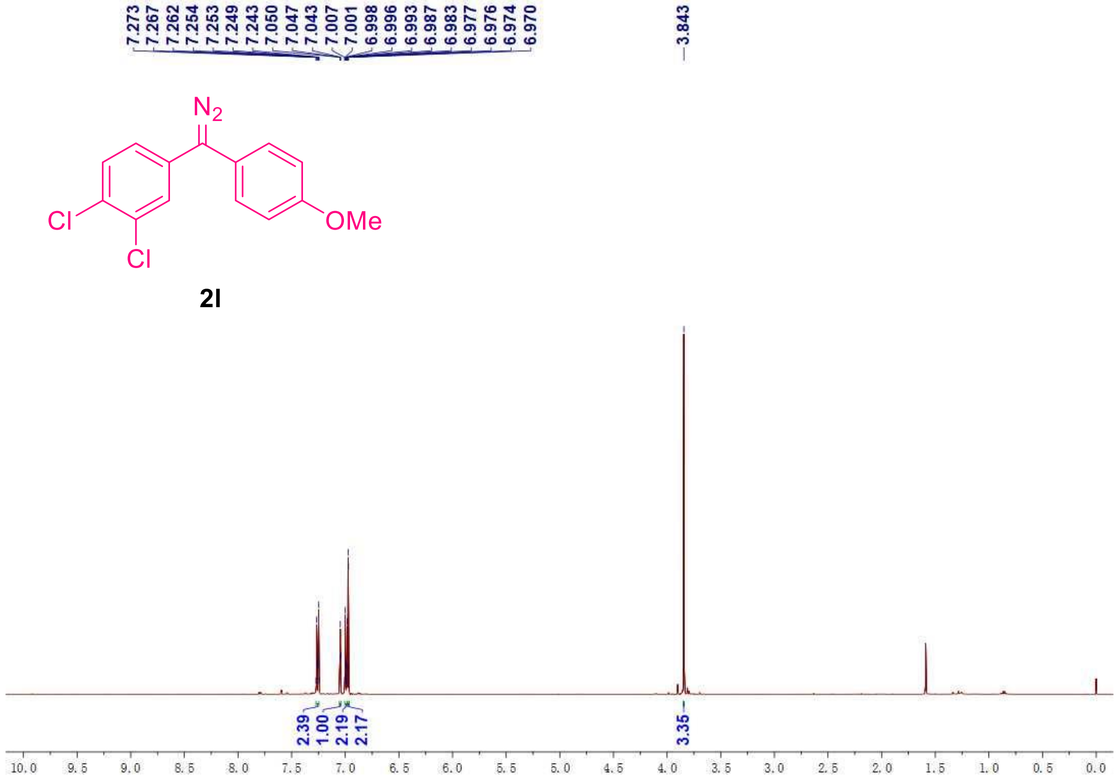

^{13}C NMR (126 MHz, $CDCl_3$)

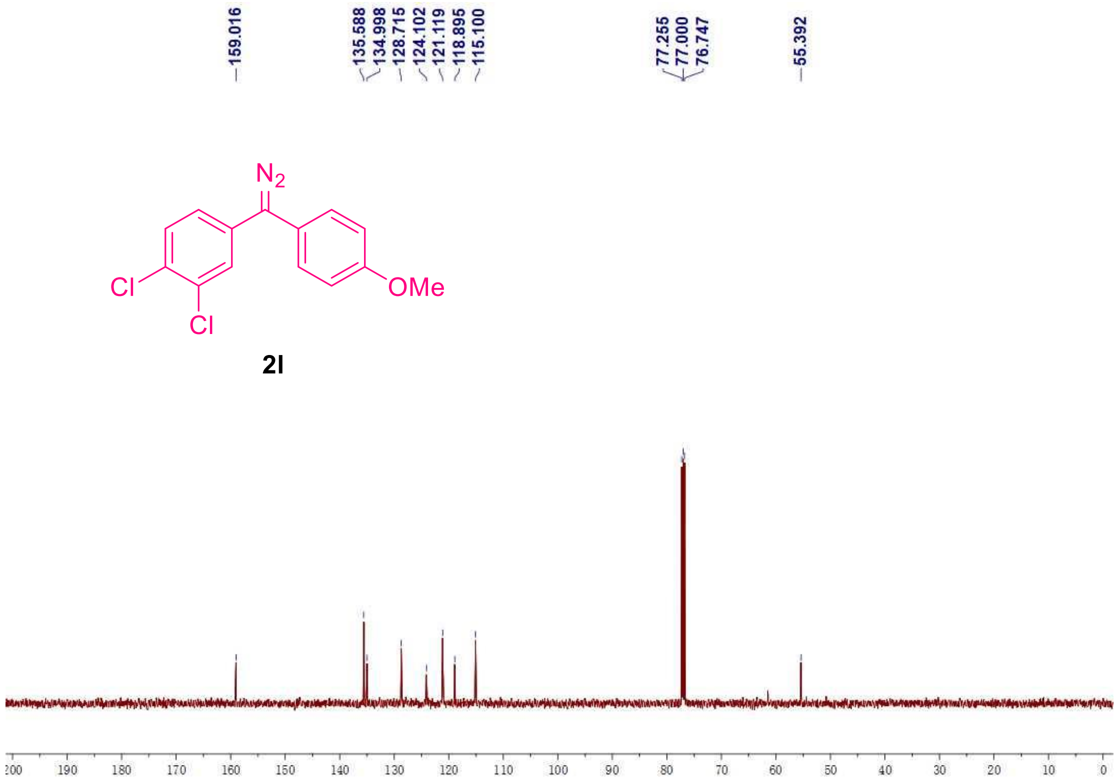

2l

1-(diazo(4-methoxyphenyl)methyl)-3,5-difluorobenzene (**2m**)

^{1}H NMR (500 MHz, $CDCl_3$)

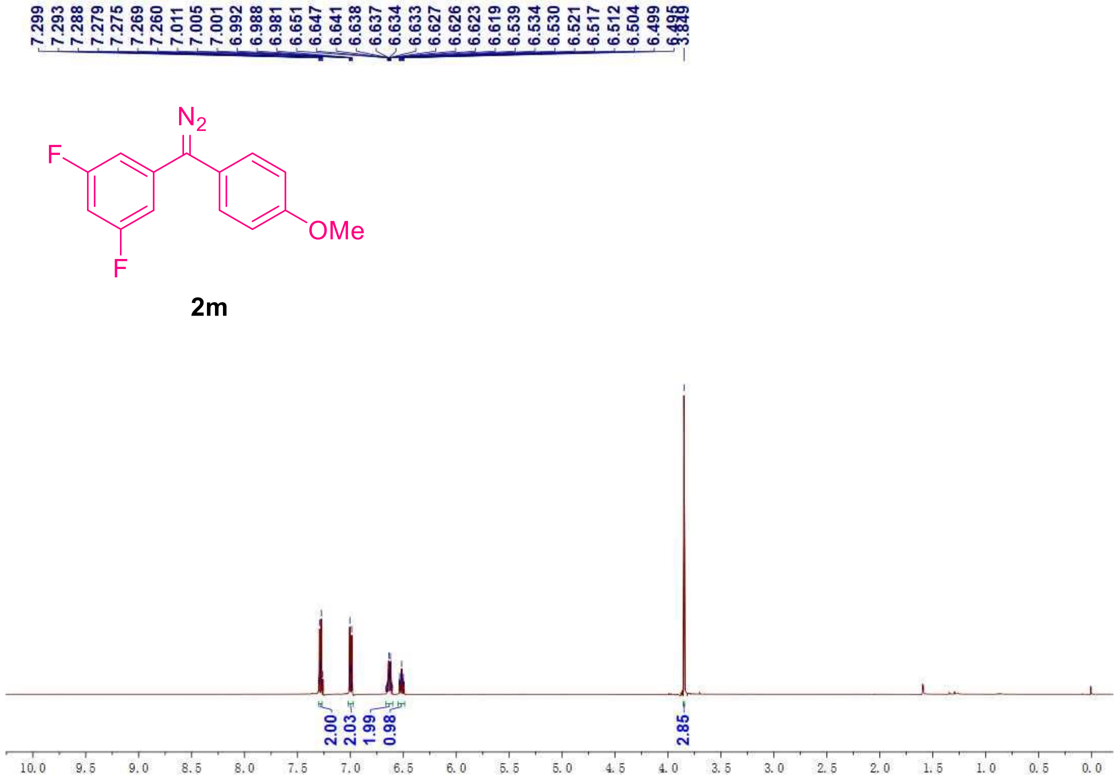

2m

^{13}**C NMR** (126 MHz, $CDCl_3$)

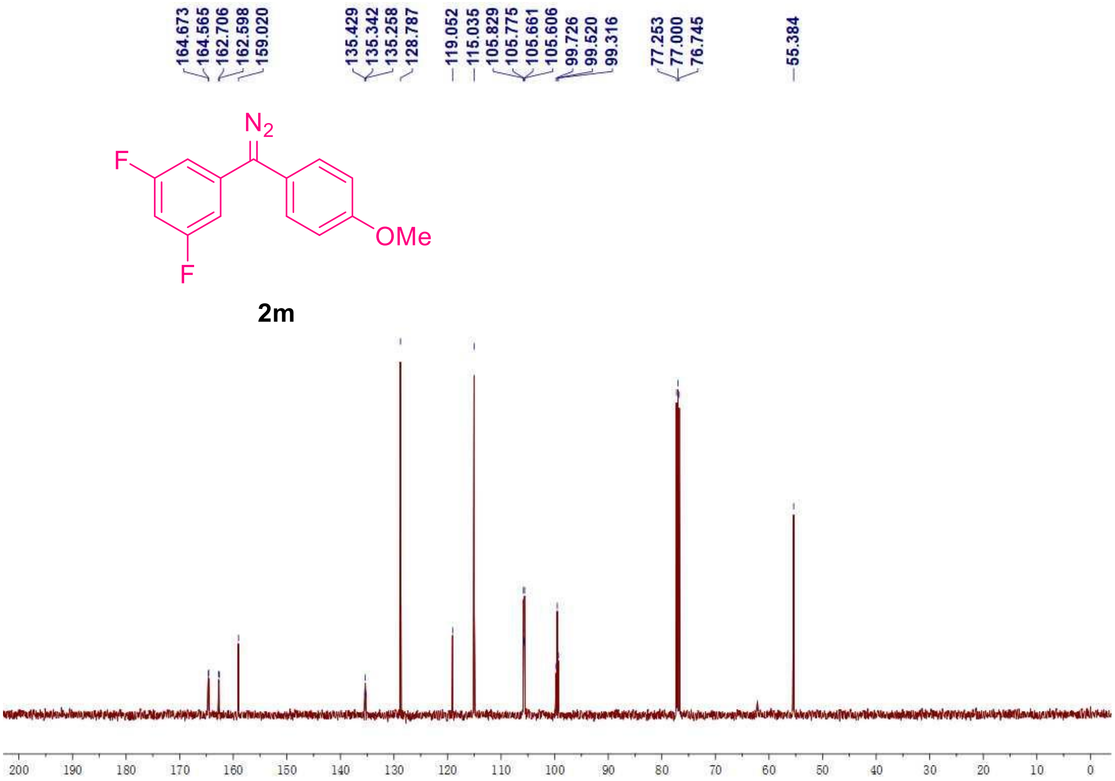

^{19}**F NMR** (471 MHz, $CDCl_3$)

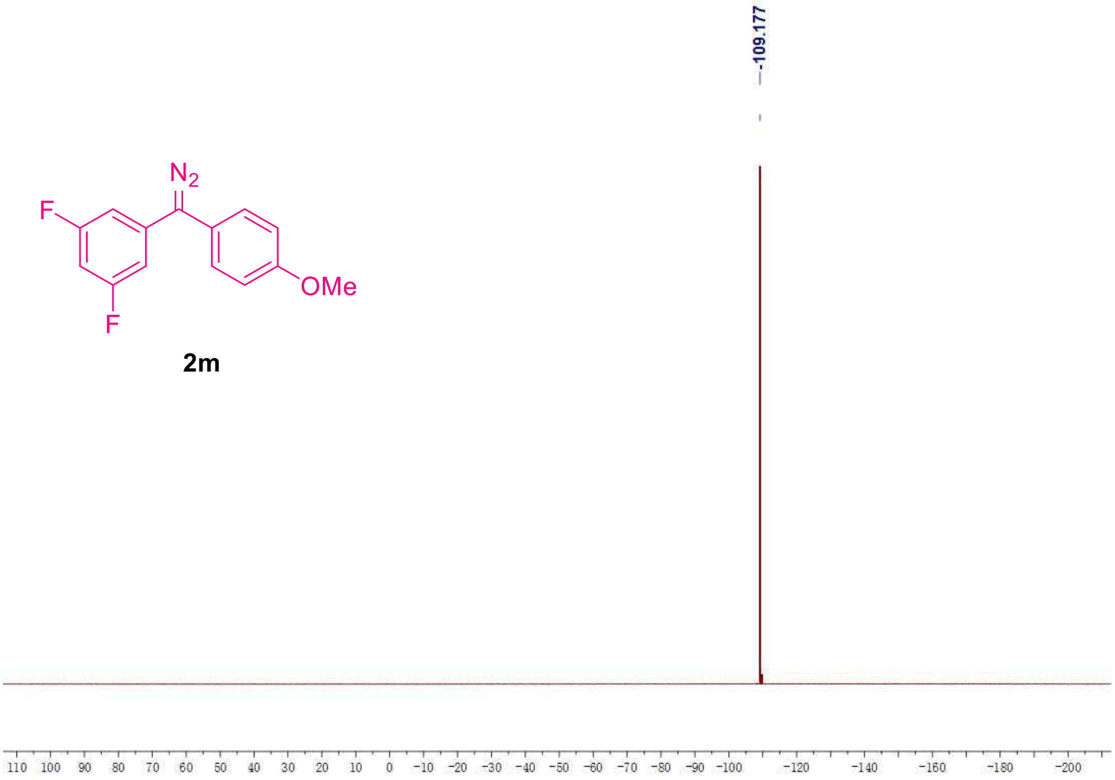

1-(diazo(4-fluorophenyl)methyl)-4-methoxybenzene (2n)

^{1}**H NMR** (500 MHz, $CDCl_3$)

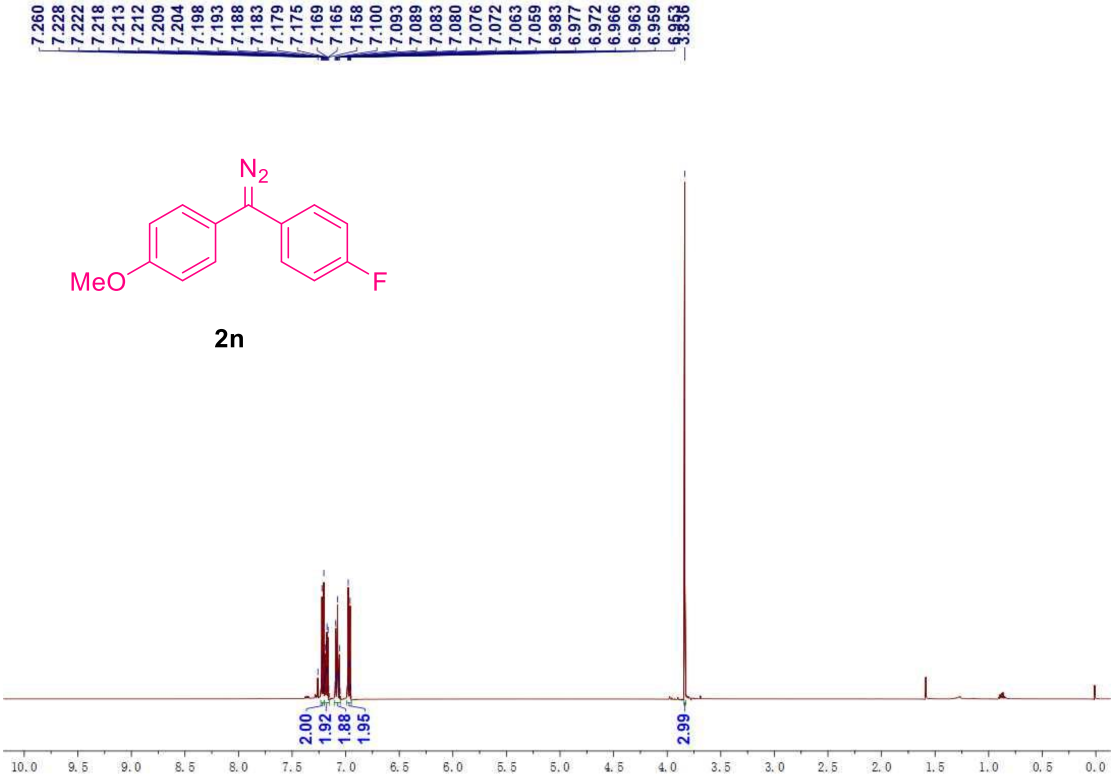

^{13}**C NMR** (126 MHz, $CDCl_3$)

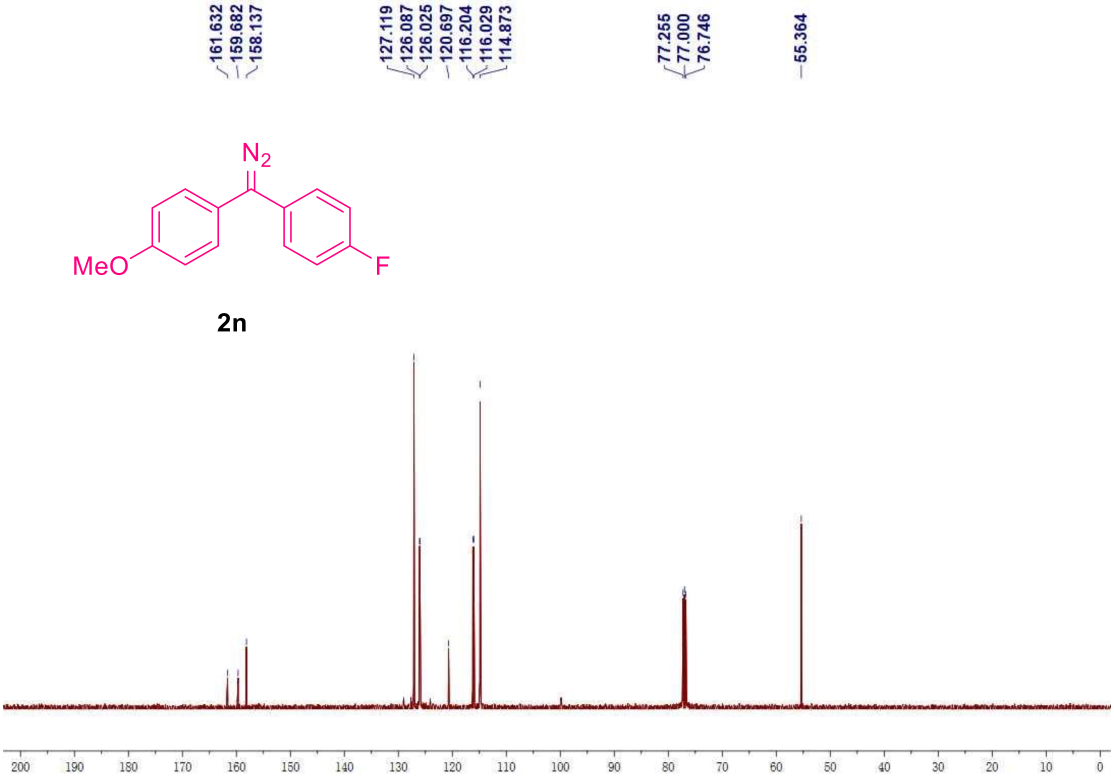

^{19}F NMR (471 MHz, $CDCl_3$)

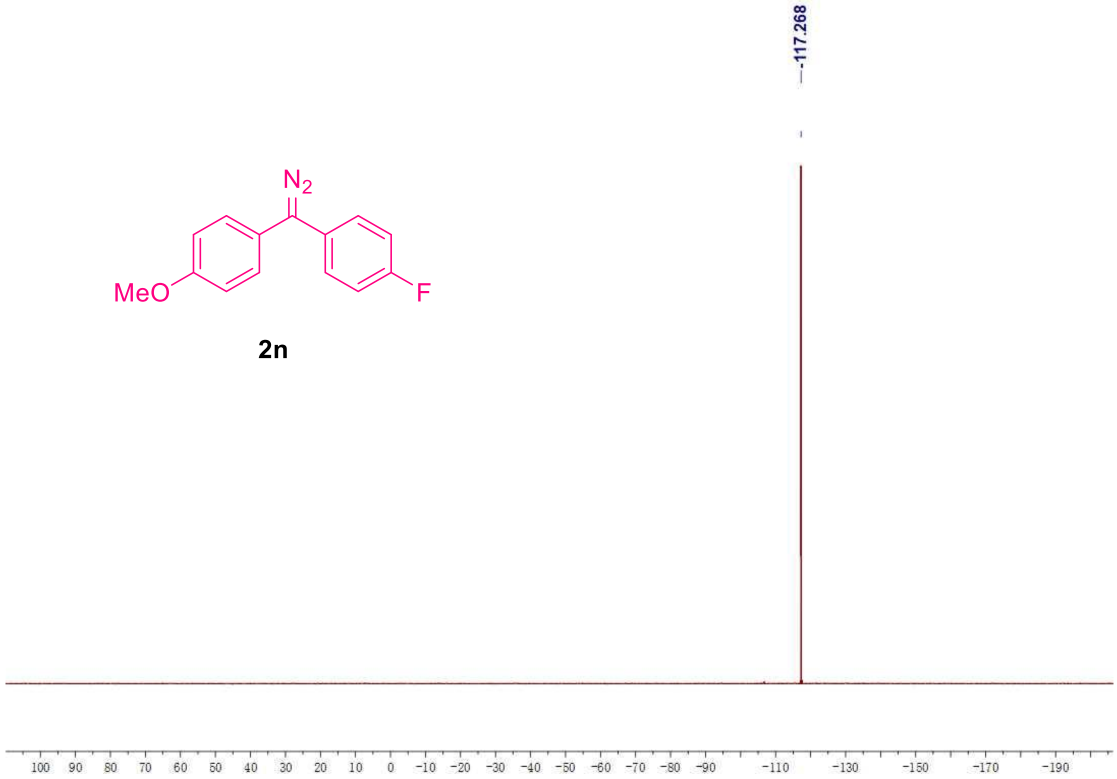

1-bromo-4-(diazo(4-methoxyphenyl)methyl)benzene (**2o**)

^{1}H NMR (500 MHz, $CDCl_3$)

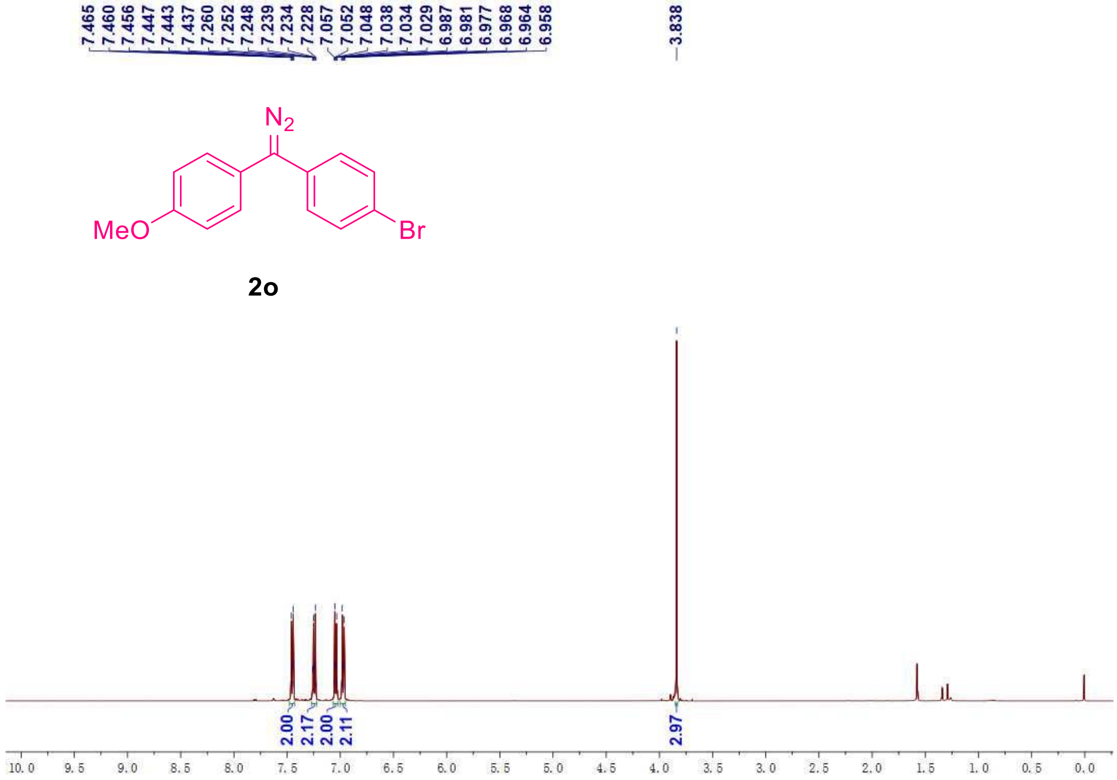

^{13}C NMR (126 MHz, $CDCl_3$)

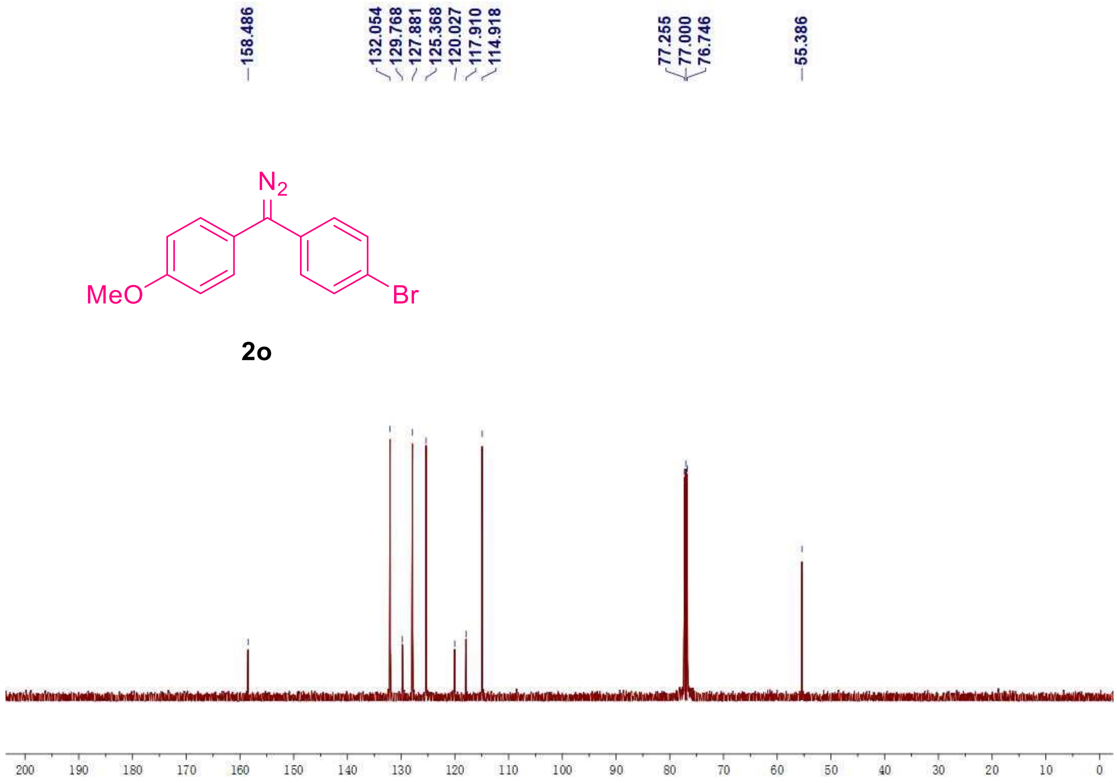

4-(diazo(4-methoxyphenyl)methyl)benzonitrile (2p)

^{1}H NMR (400 MHz, $CDCl_3$)

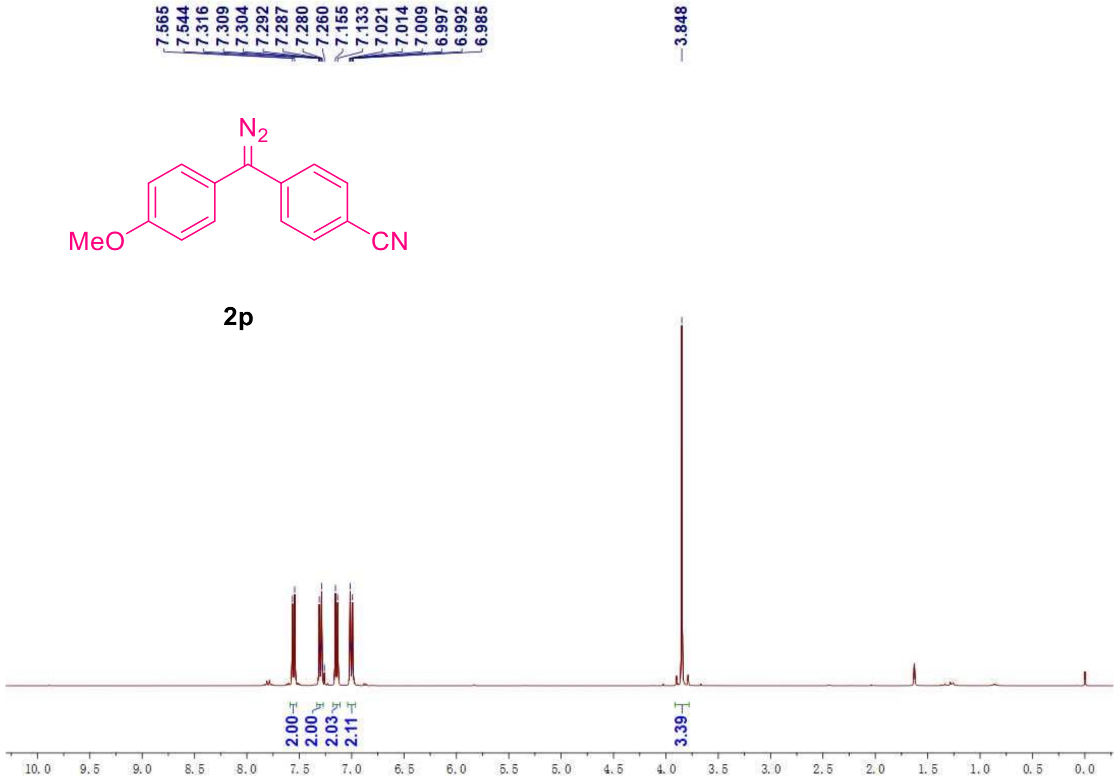

^{13}C NMR (101 MHz, $CDCl_3$)

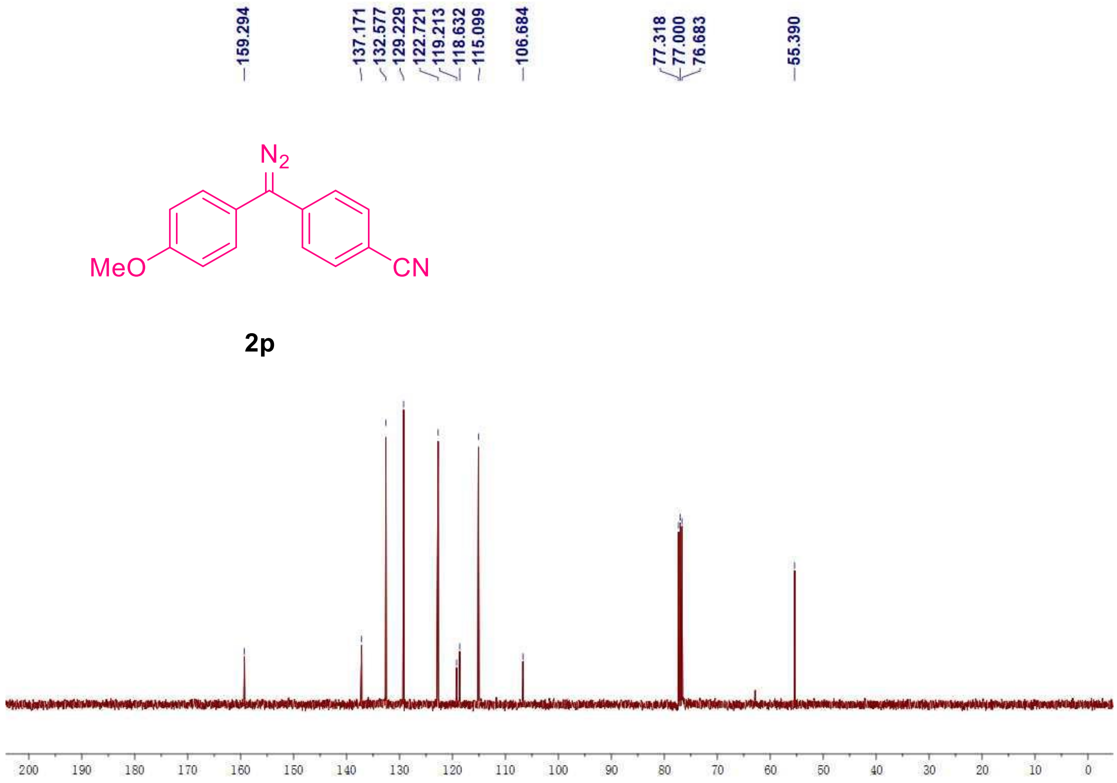

1-(diazo(4-(methylsulfonyl)phenyl)methyl)-4-methoxybenzene (3q)

^{1}H NMR (500 MHz, $CDCl_3$)

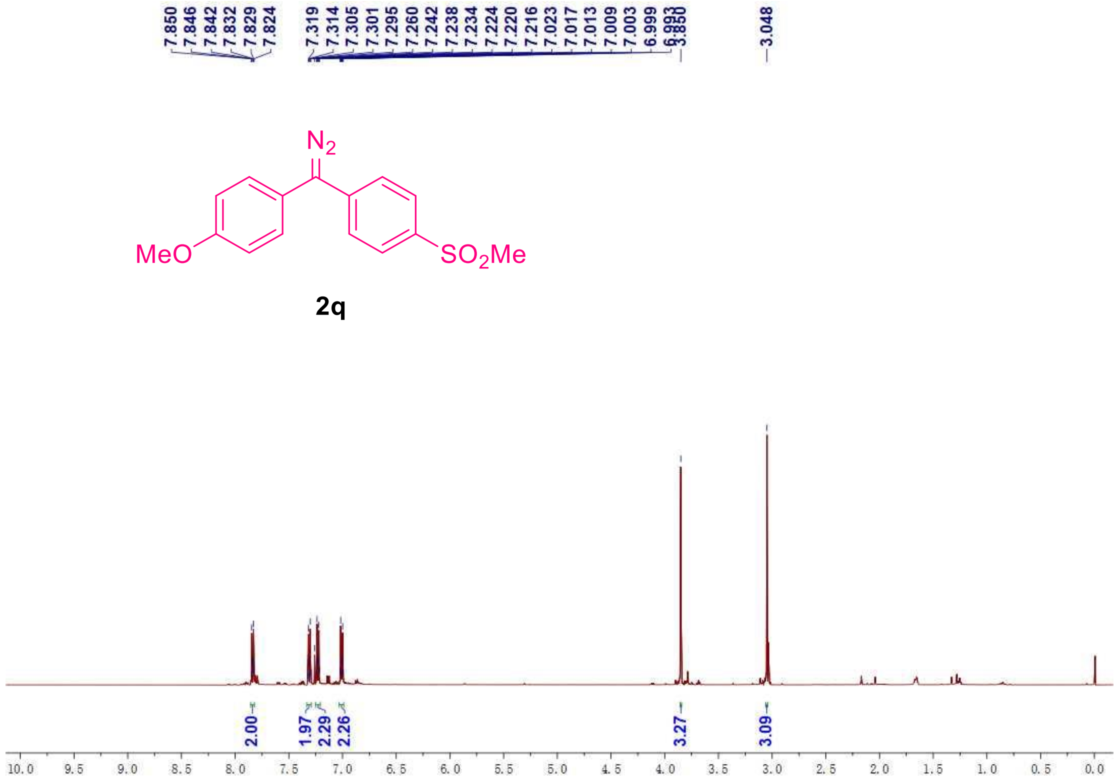

^{13}C NMR (126 MHz, $CDCl_3$)

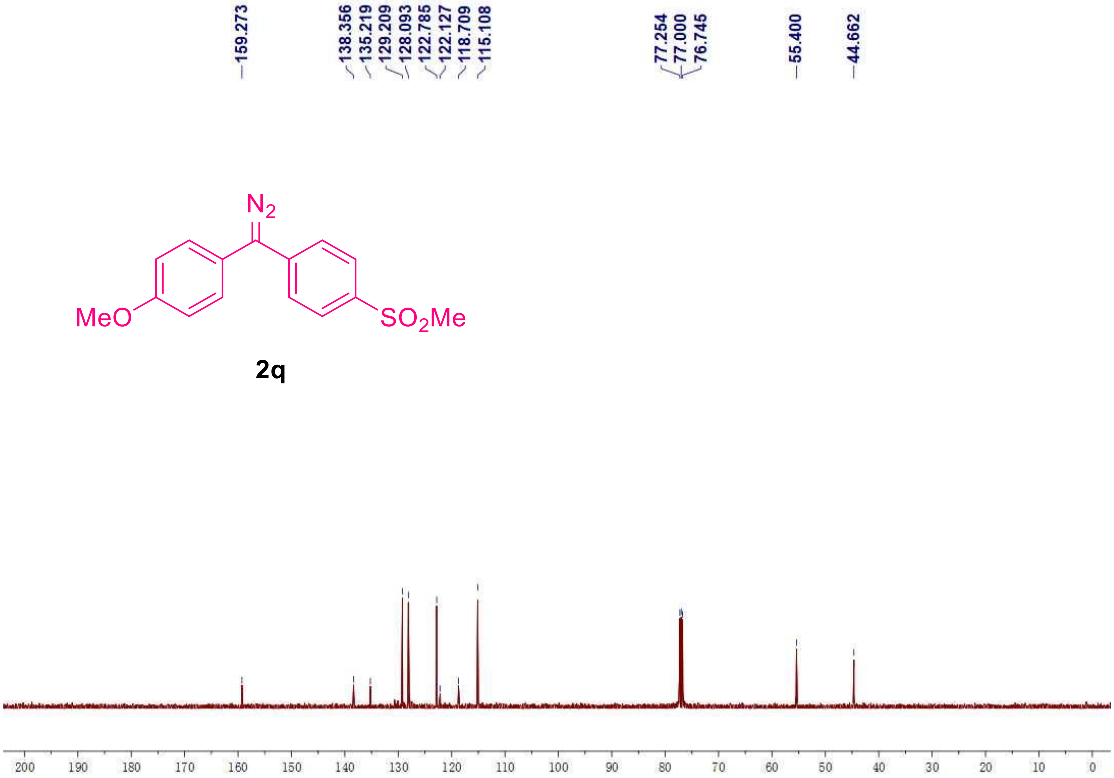

ethyl 4-(diazo(4-methoxyphenyl)methyl)benzoate (**2r**)

^{1}H NMR (500 MHz, $CDCl_3$)

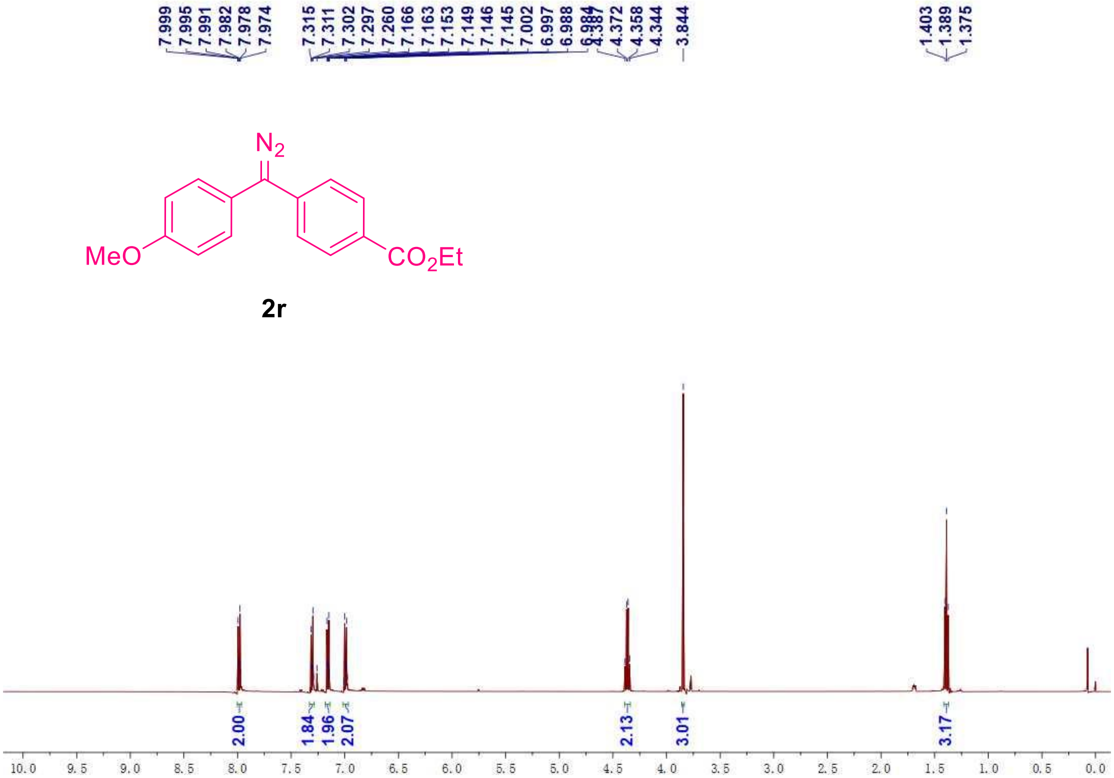

^{13}C NMR (126 MHz, $CDCl_3$)

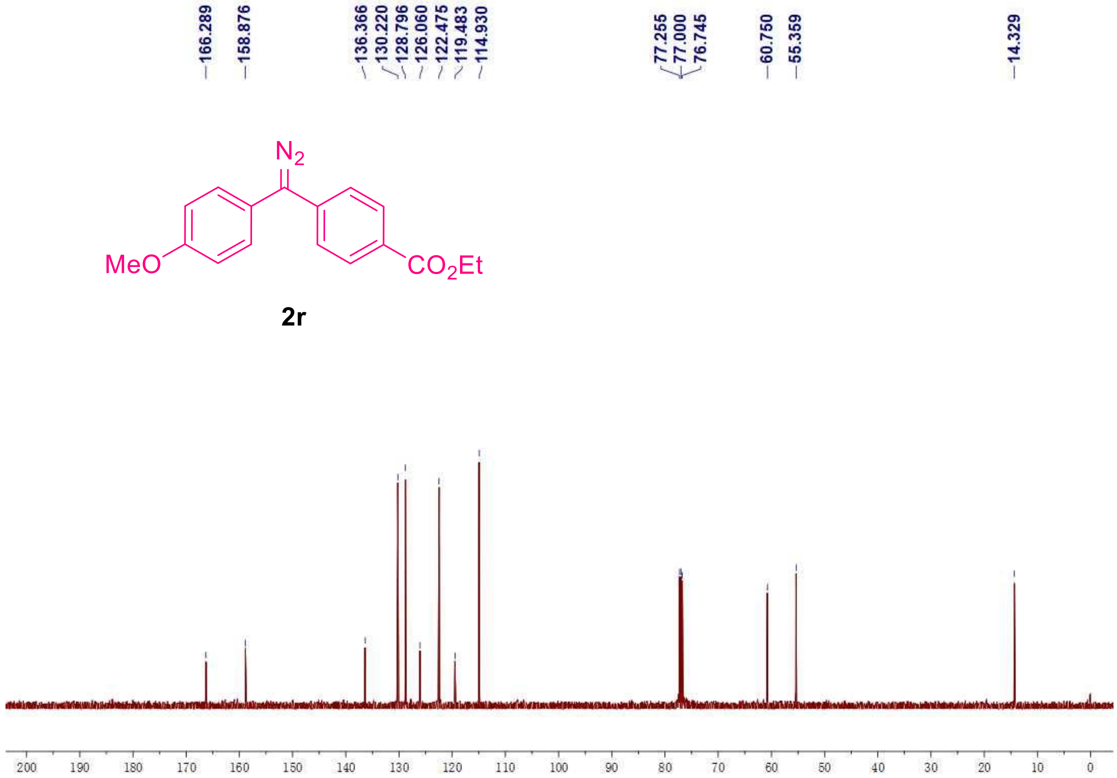

4-(diazo(4-methoxyphenyl)methyl)-*N*,*N*-dimethylbenzamide (2s)

^{1}H NMR (500 MHz, $CDCl_3$)

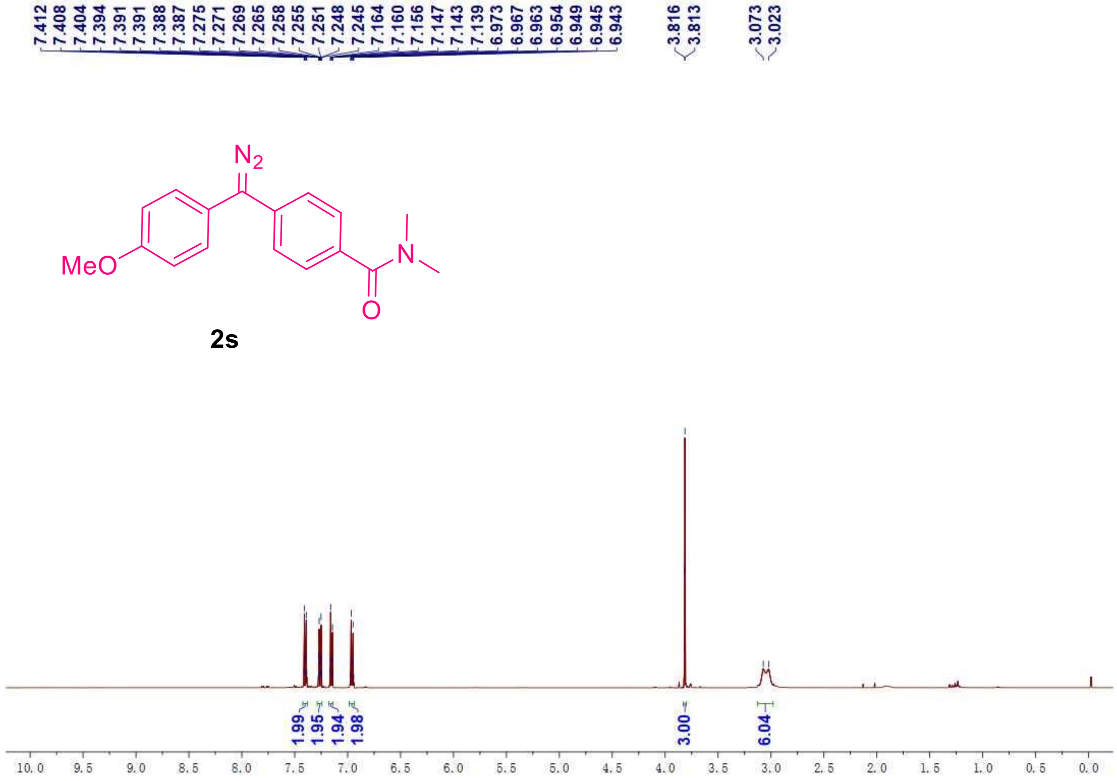

^{13}**C NMR** (126 MHz, $CDCl_3$)

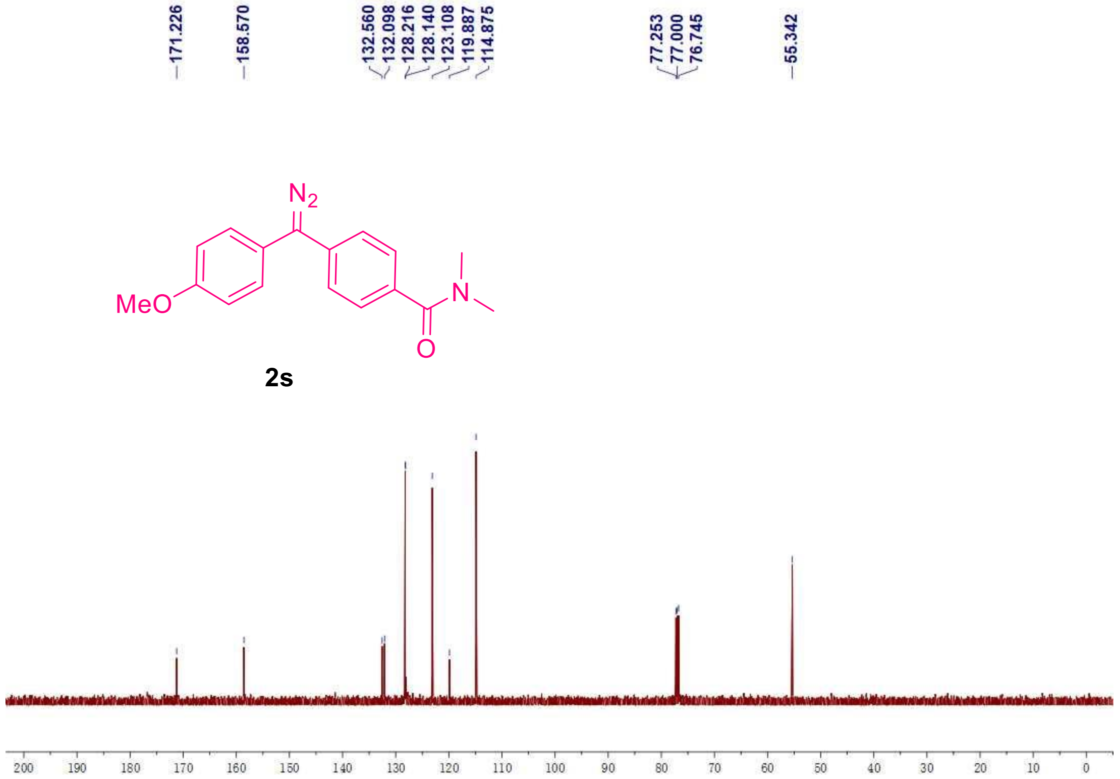

1-(diazo(4-(trifluoromethoxy)phenyl)methyl)-4-methoxybenzene (2t)

^{1}**H NMR** (500 MHz, $CDCl_3$)

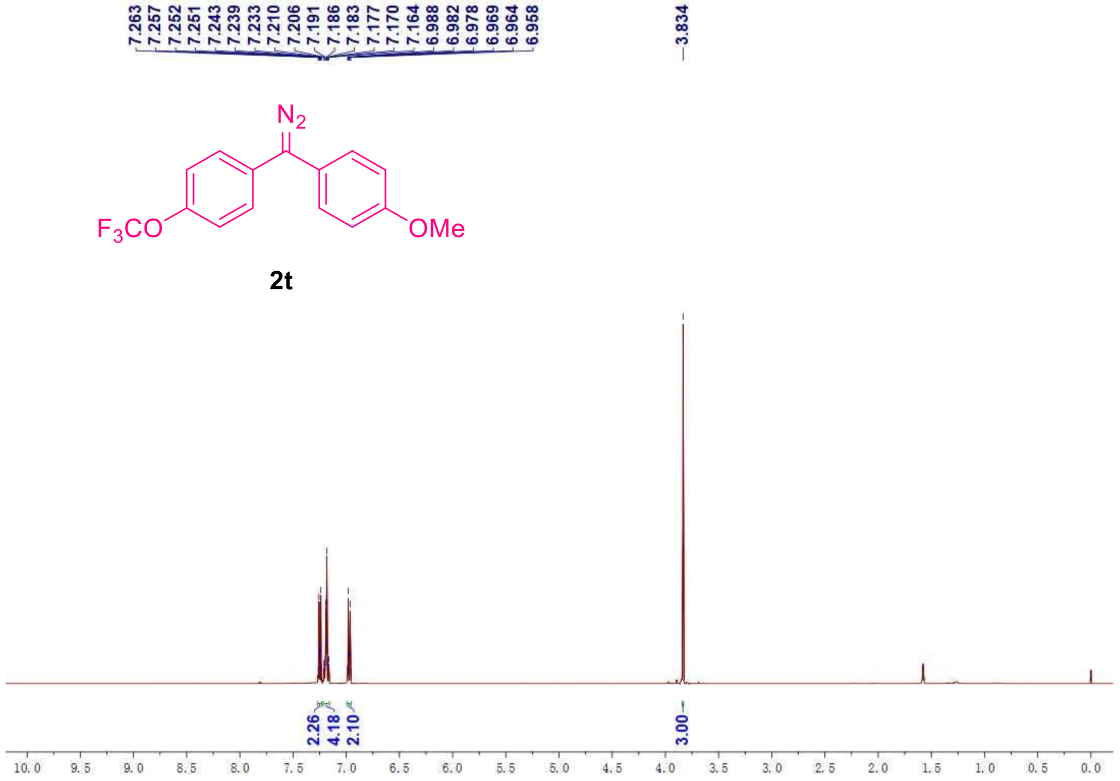

^{13}**C NMR** (126 MHz, $CDCl_3$)

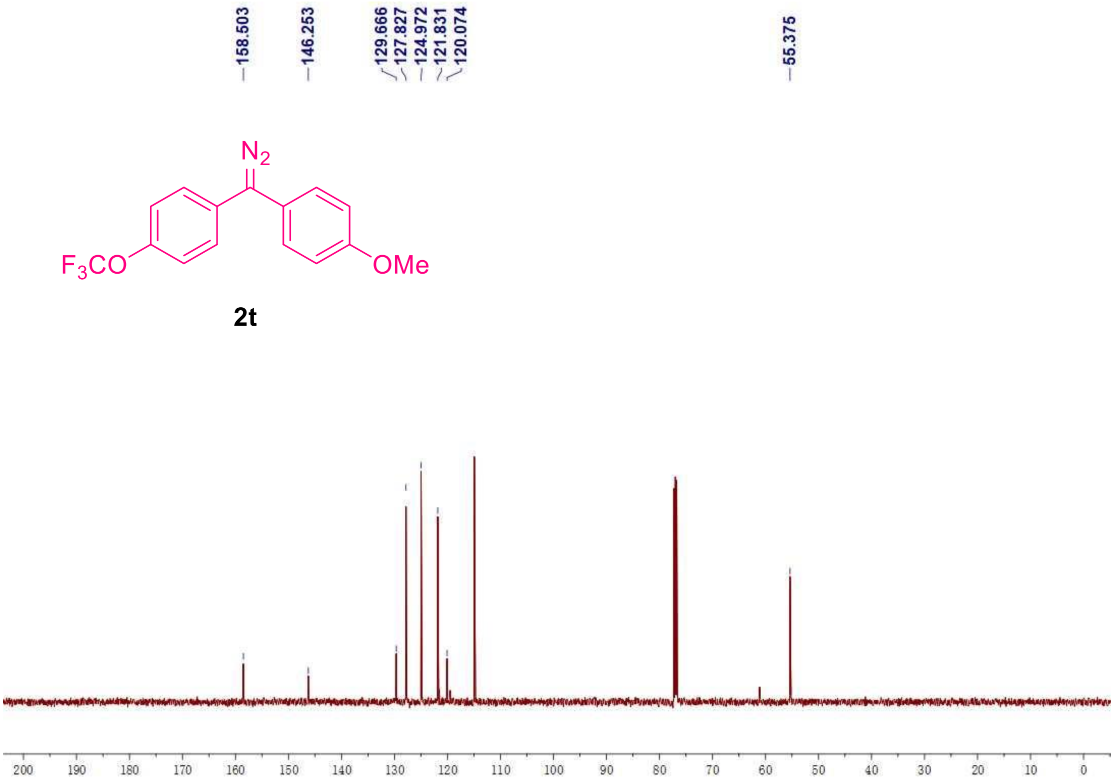

^{19}**F NMR** (471 MHz, $CDCl_3$)

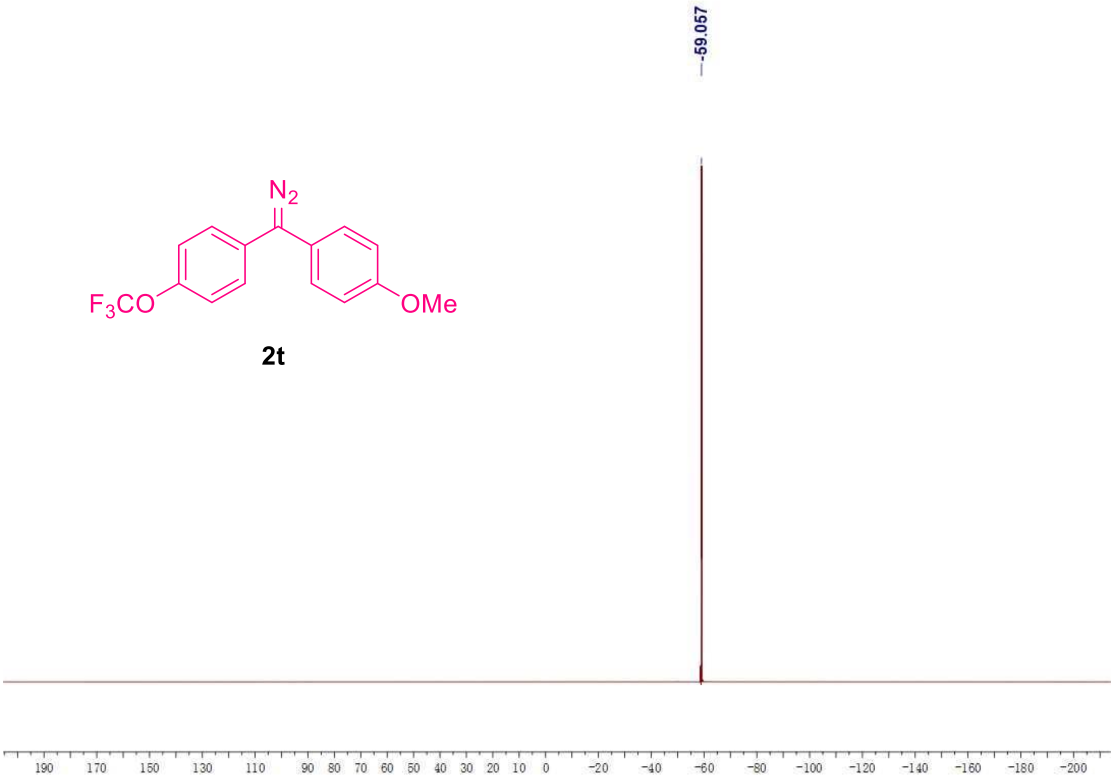

1-(diazo(4-(trifluoromethyl)phenyl)methyl)-4-methoxybenzene (2u)

^{1}H NMR (500 MHz, CDCl$_3$)

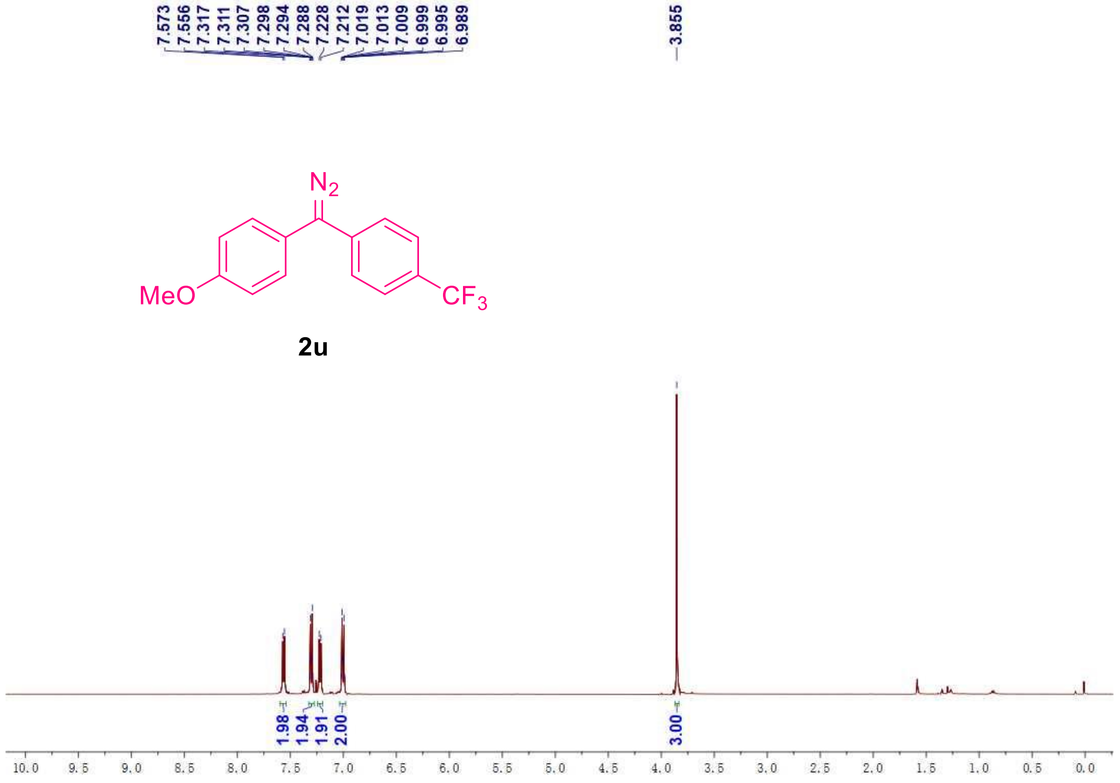

^{13}C NMR (126 MHz, CDCl$_3$)

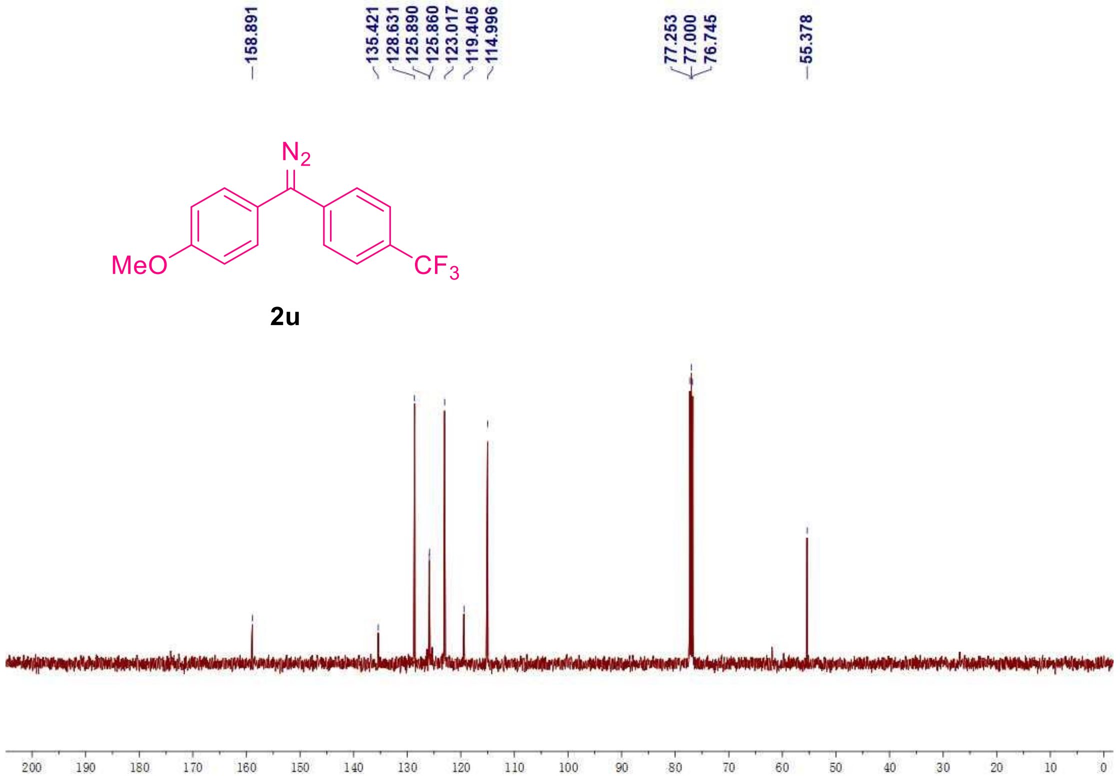

^{19}F NMR (471 MHz, CDCl$_3$)

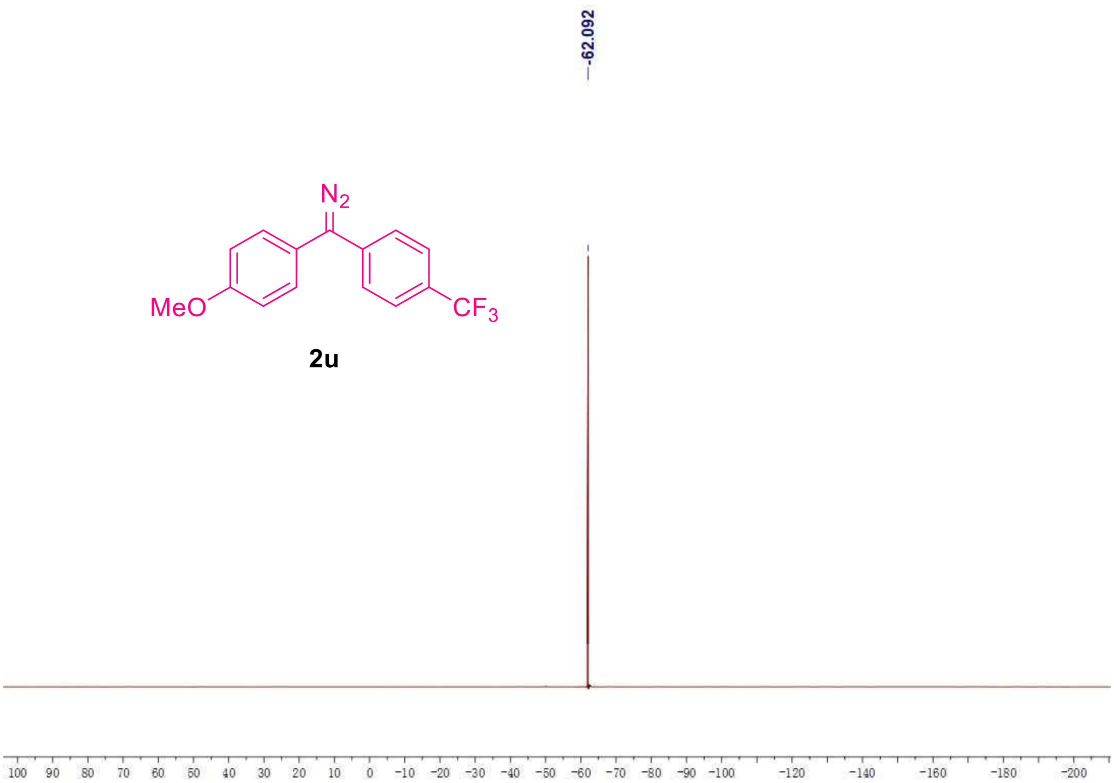

1-(diazo(4-(trifluoromethyl)phenyl)methyl)-3-methoxybenzene (2v)

^{1}H NMR (500 MHz, $CDCl_3$)

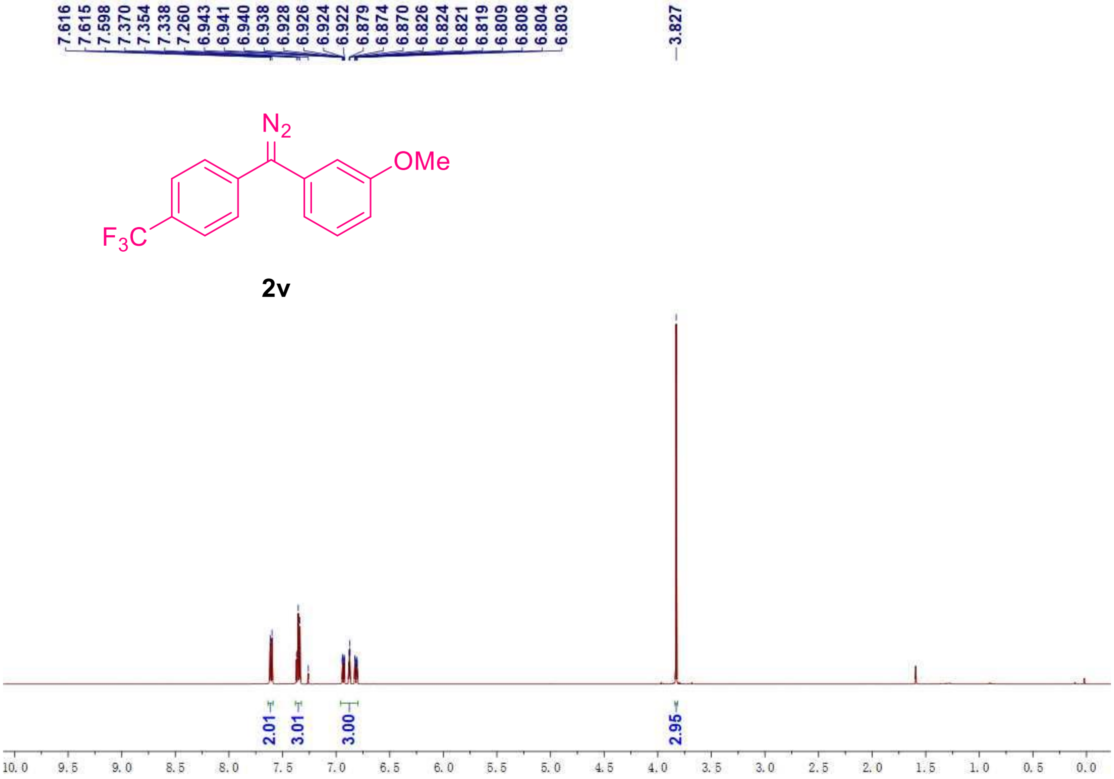

^{13}C NMR (126 MHz, $CDCl_3$)

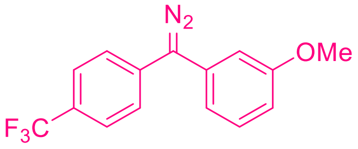

2v

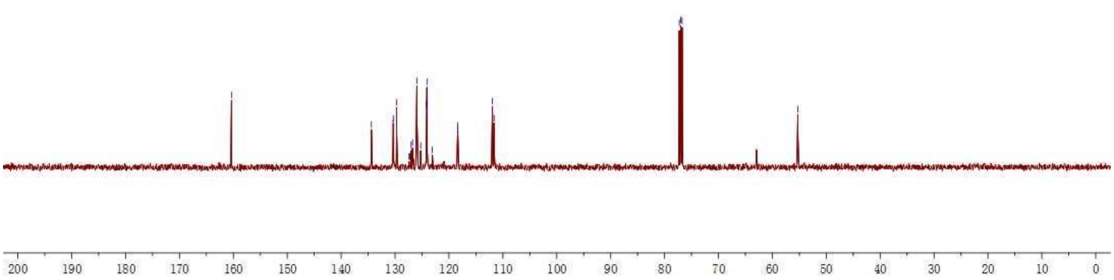

^{19}F NMR (471 MHz, $CDCl_3$)

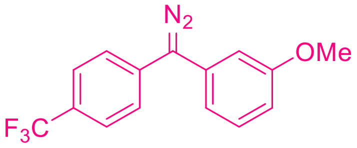

2v

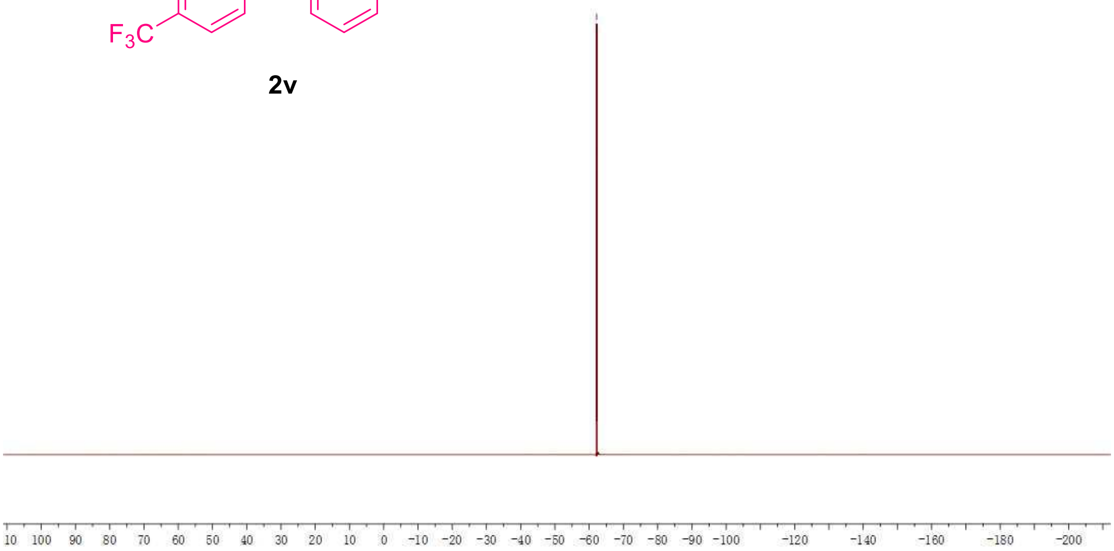

1-(benzyloxy)-4-(diazo(4-(trifluoromethyl)phenyl)methyl)benzene (2w)

^{1}H NMR (500 MHz, $CDCl_3$)

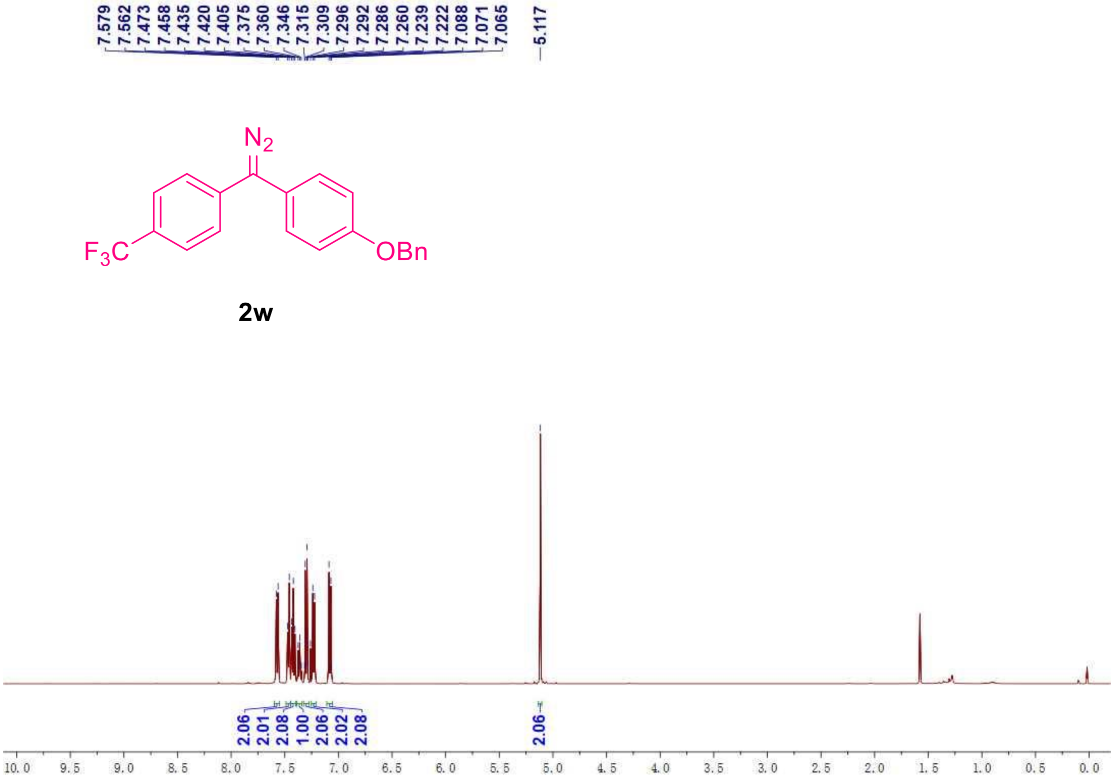

^{13}C NMR (126 MHz, $CDCl_3$)

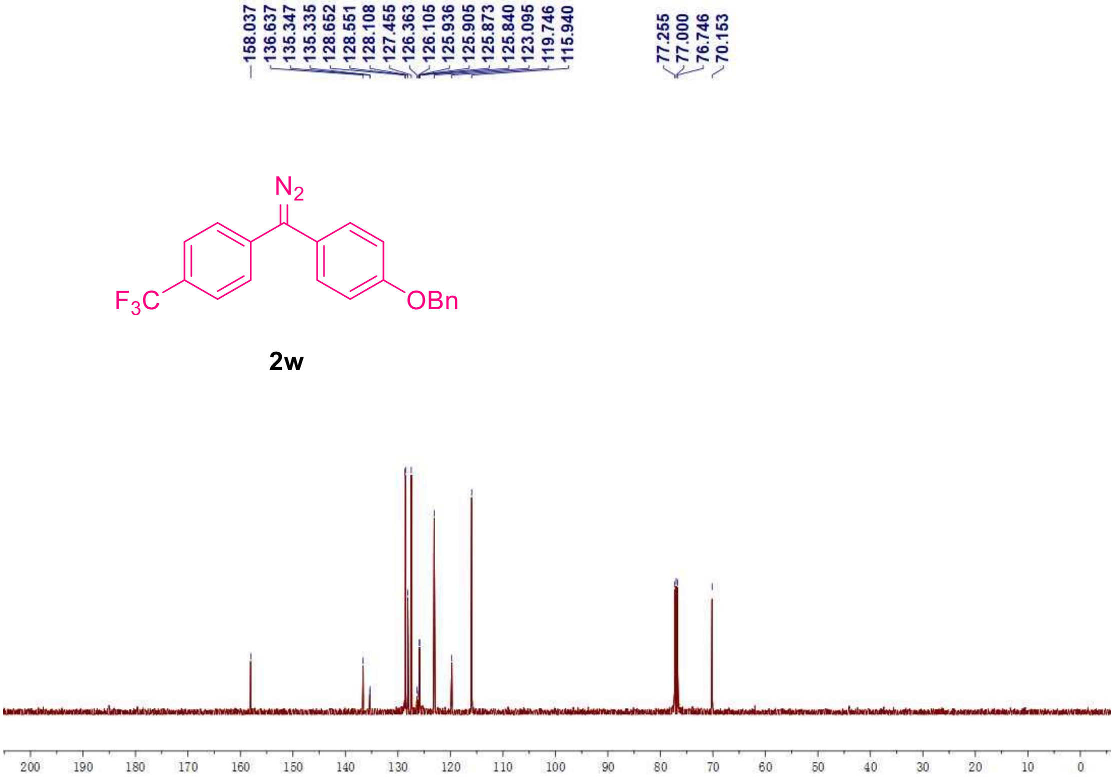

^{19}F NMR (471 MHz, $CDCl_3$)

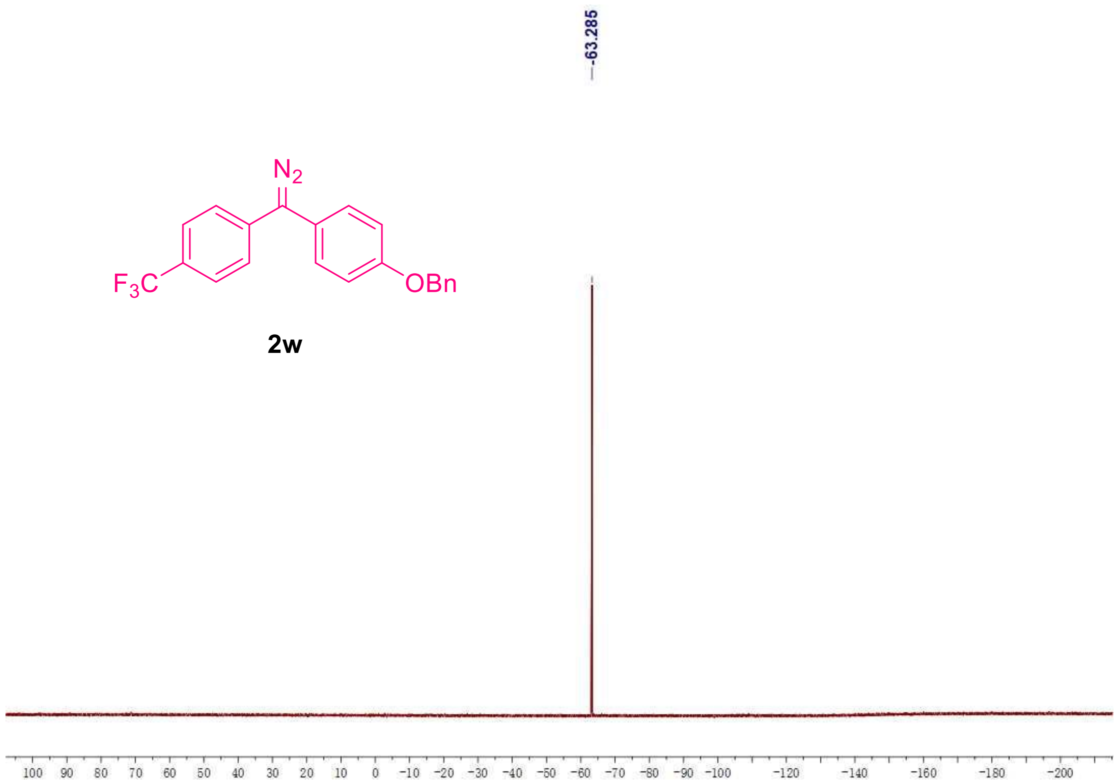

1-(diazo(4-methoxyphenyl)methyl)-4-nitrobenzene (2x)

^{1}H NMR (500 MHz, $CDCl_3$)

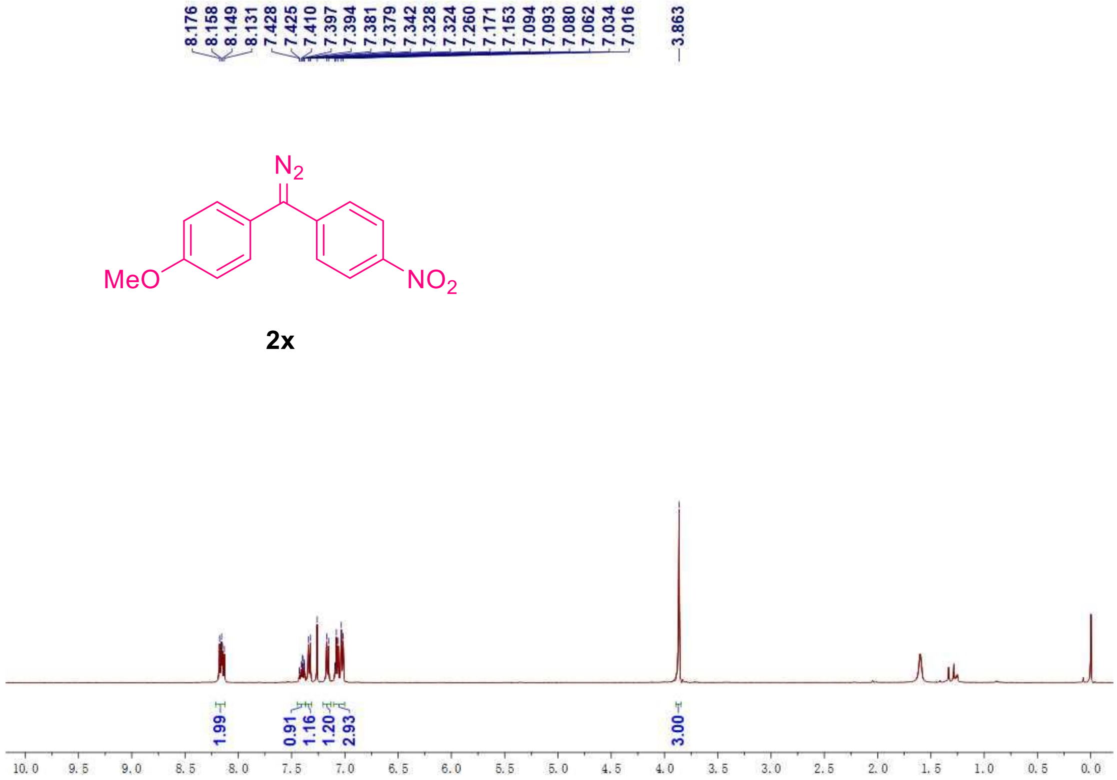

^{13}C NMR (126 MHz, $CDCl_3$)

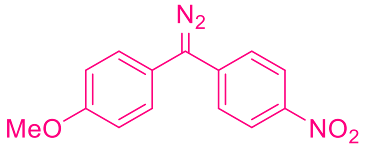

2x

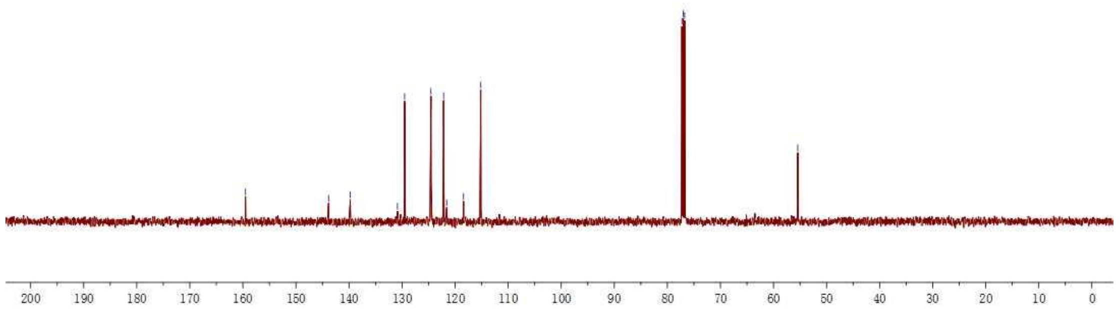

1-(diazo(4-methoxyphenyl)methyl)-3-nitrobenzene (**2y**)

^{1}H NMR (400 MHz, $CDCl_3$)

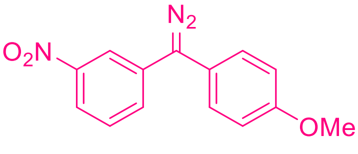

2y

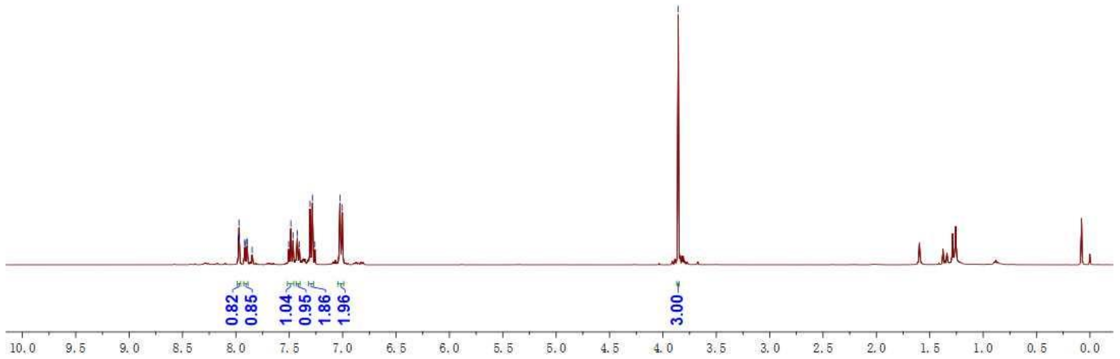

1-(diazo(4-nitrophenyl)methyl)-4-methylbenzene (2z)

[1]**H NMR** (500 MHz, $CDCl_3$)

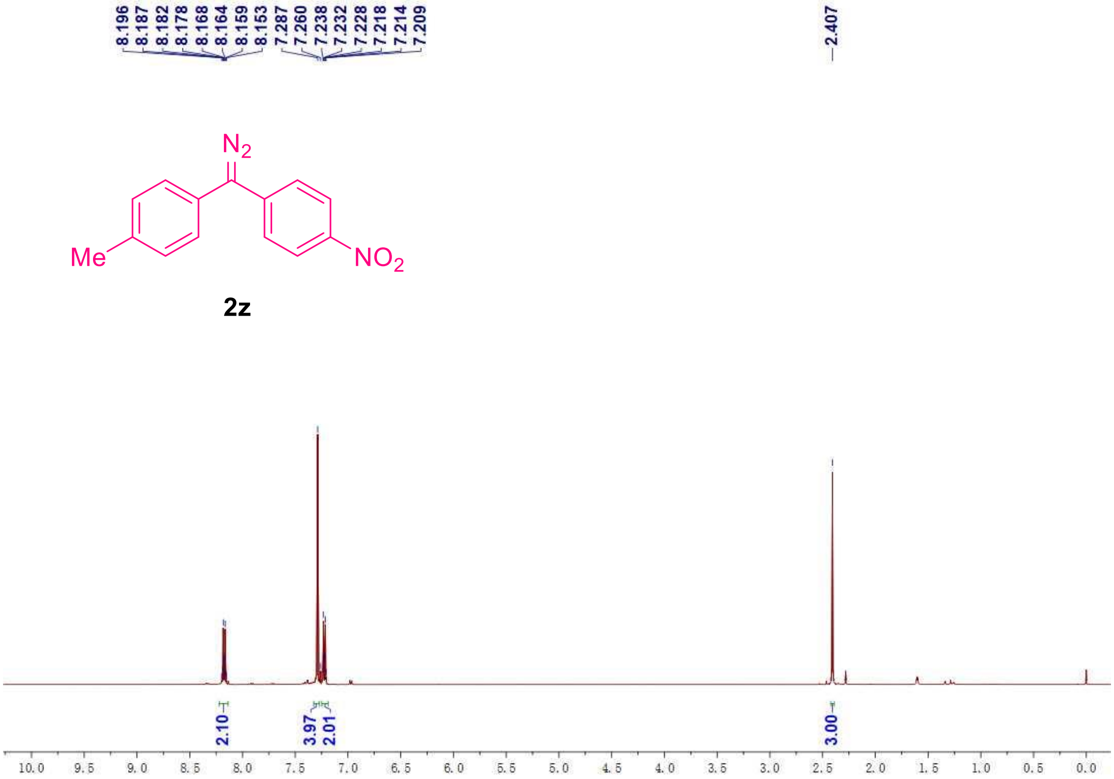

[13]**C NMR** (126 MHz, $CDCl_3$)

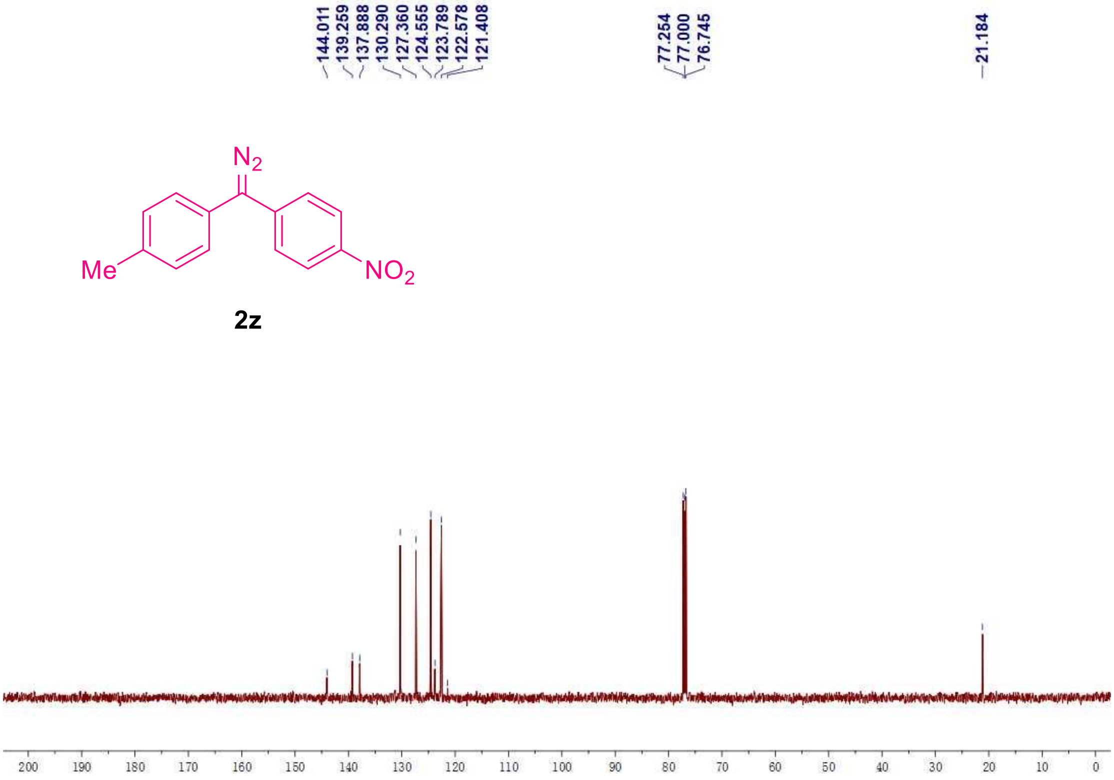

4-(benzo[d][1,3]dioxol-5-yl(diazo)methyl)benzonitrile (2aa)

^{1}H NMR (400 MHz, $CDCl_3$)

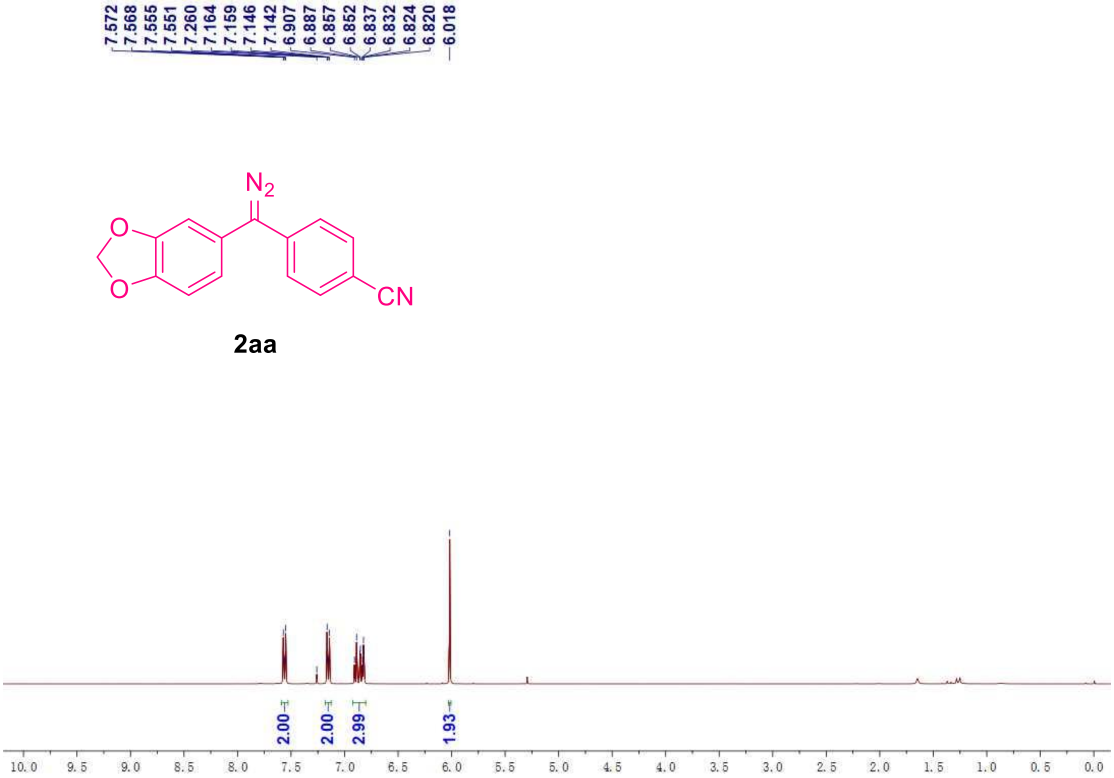

^{13}C NMR (101 MHz, $CDCl_3$)

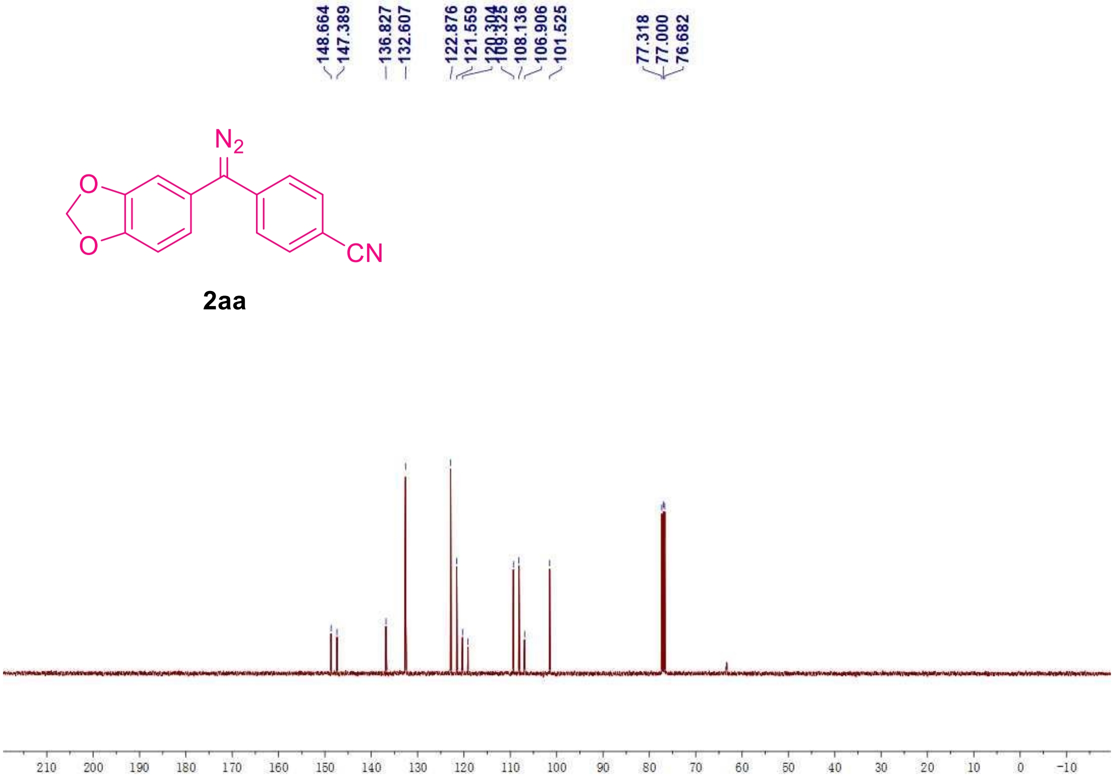

1-benzhydryl-2-(tert-butyl)-2,3-dihydro-1H-benzo[c][1,2]azaborole (3a)

^{1}H NMR (500 MHz, $CDCl_3$)

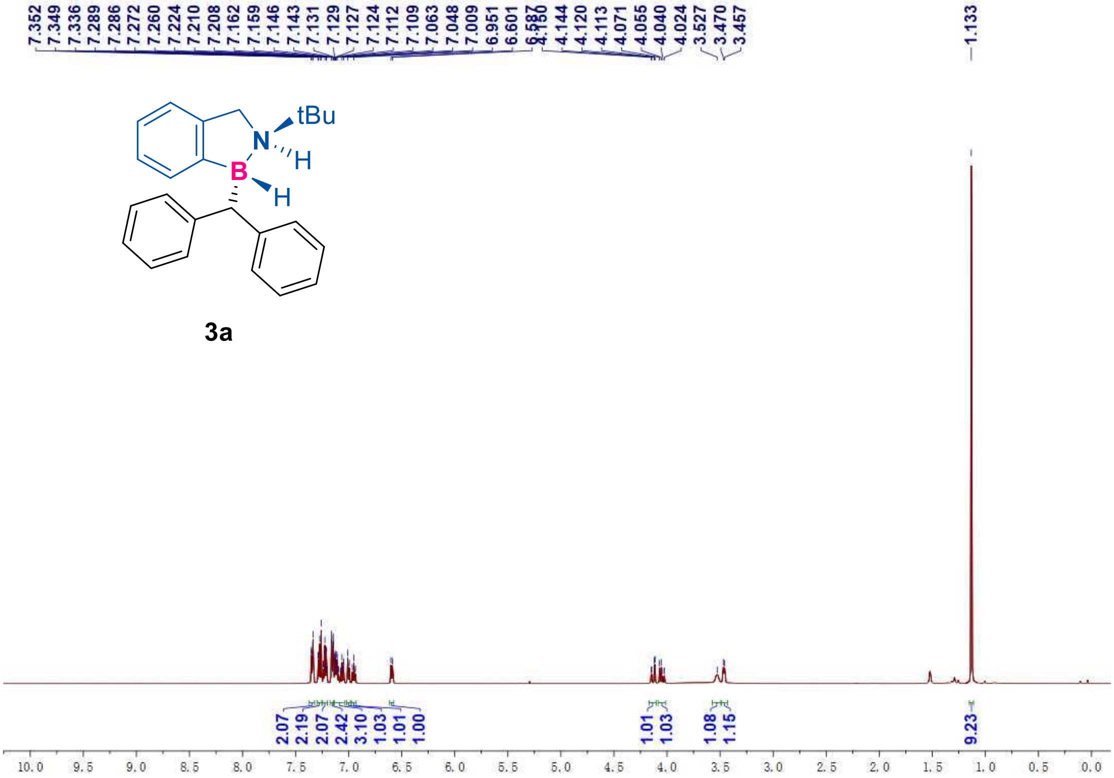

^{13}C NMR (126 MHz, $CDCl_3$)

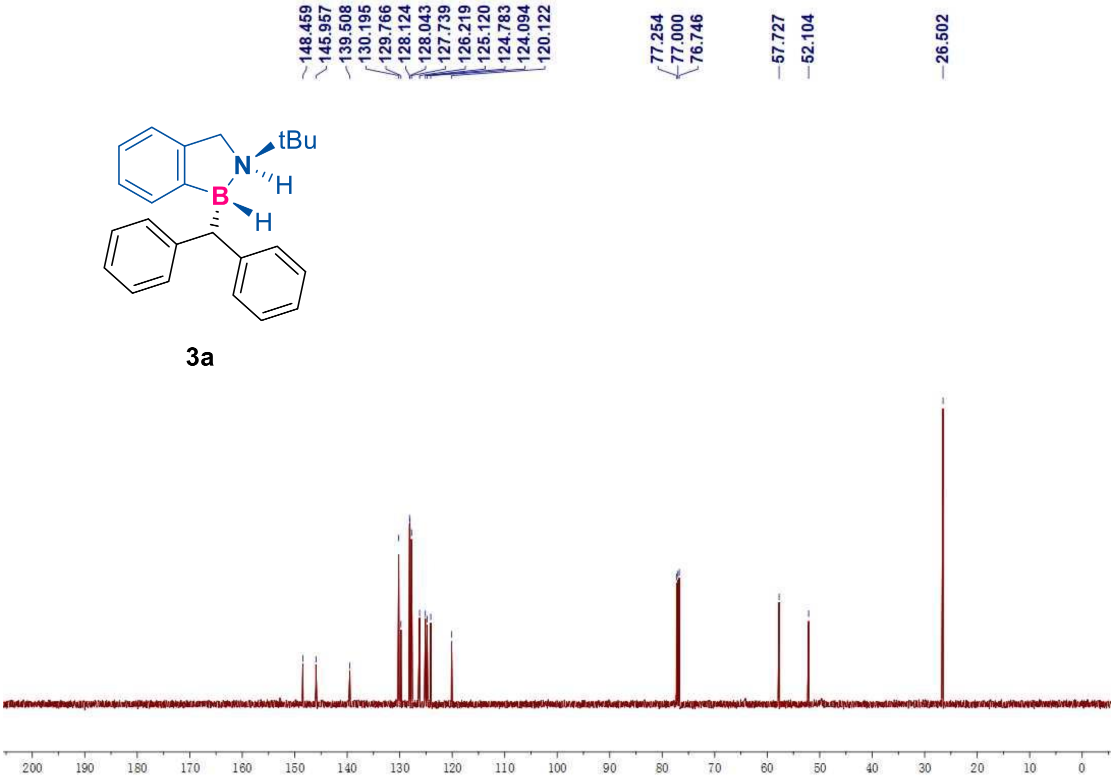

[11]**B NMR** (160 MHz, $CDCl_3$)

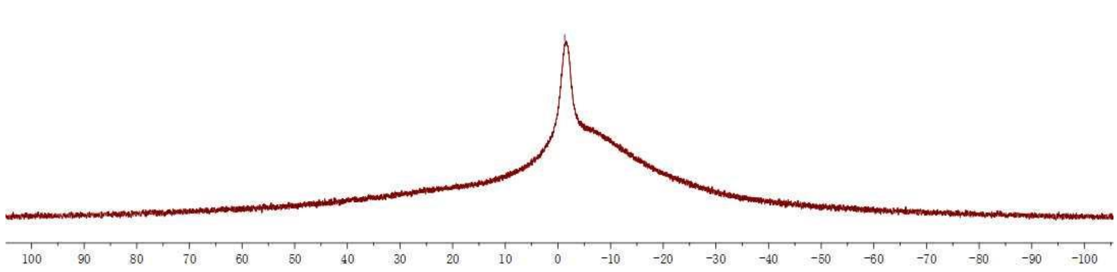

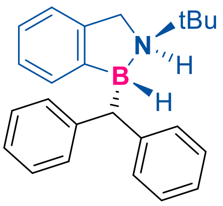

3a

1-(bis(4-methoxyphenyl)methyl)-2-(tert-butyl)-2,3-dihydro-1H-benzo[c][1,2]azaborole (**3b**)

[1]**H NMR** (400 MHz, Acetone-d_6)

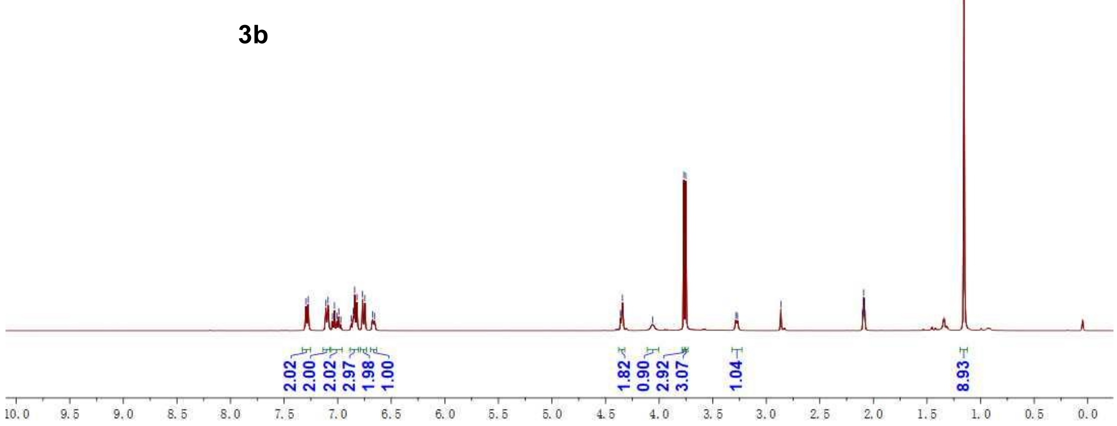

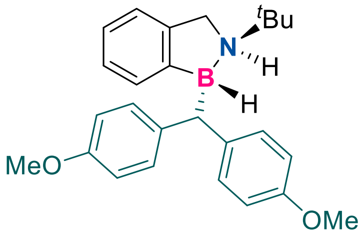

3b

^{13}**C NMR** (100 MHz, Acetone-d_6)

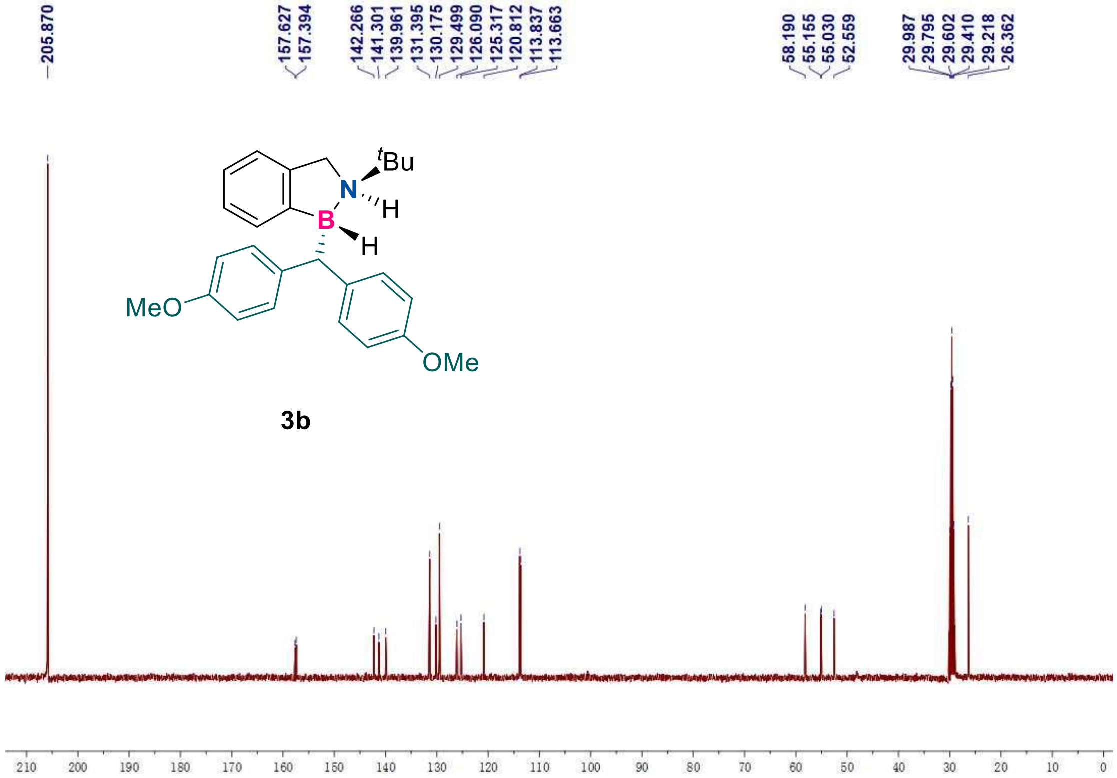

^{11}**B NMR** (128 MHz, Acetone-d_6)

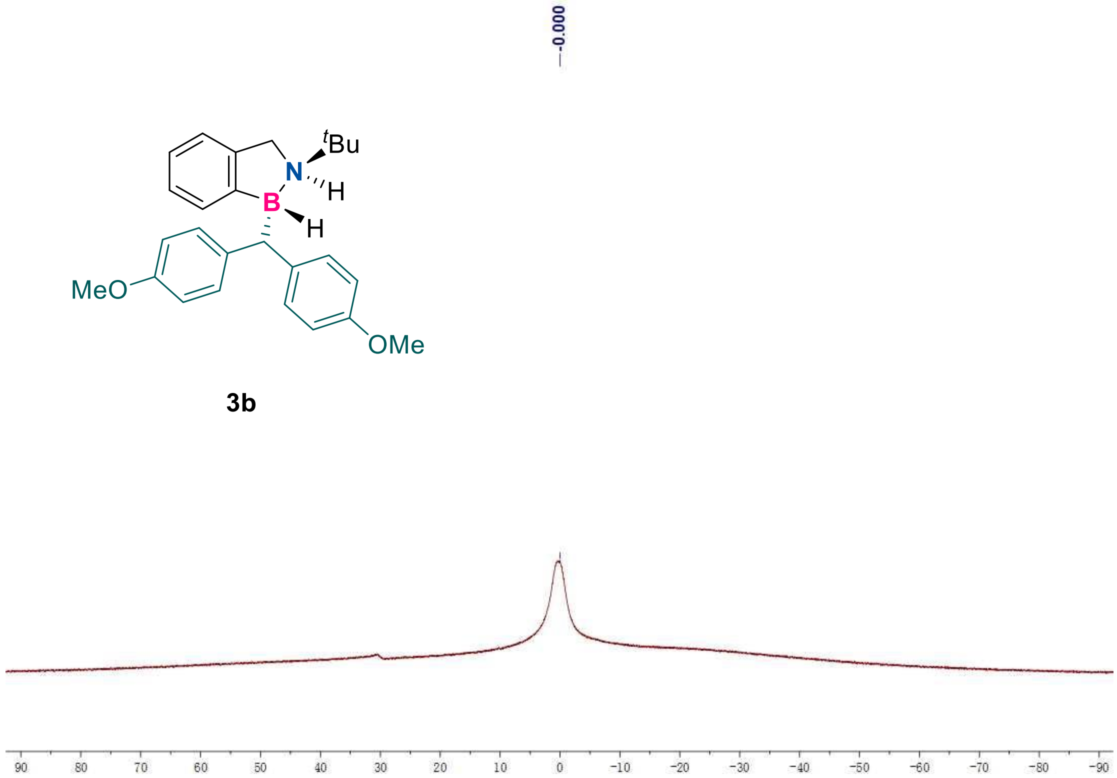

2-(tert-butyl)-1-(di-p-tolylmethyl)-2,3-dihydro-1H-benzo[c][1,2]azaborole (3c)

^{1}H NMR (400 MHz, Acetone-d_6)

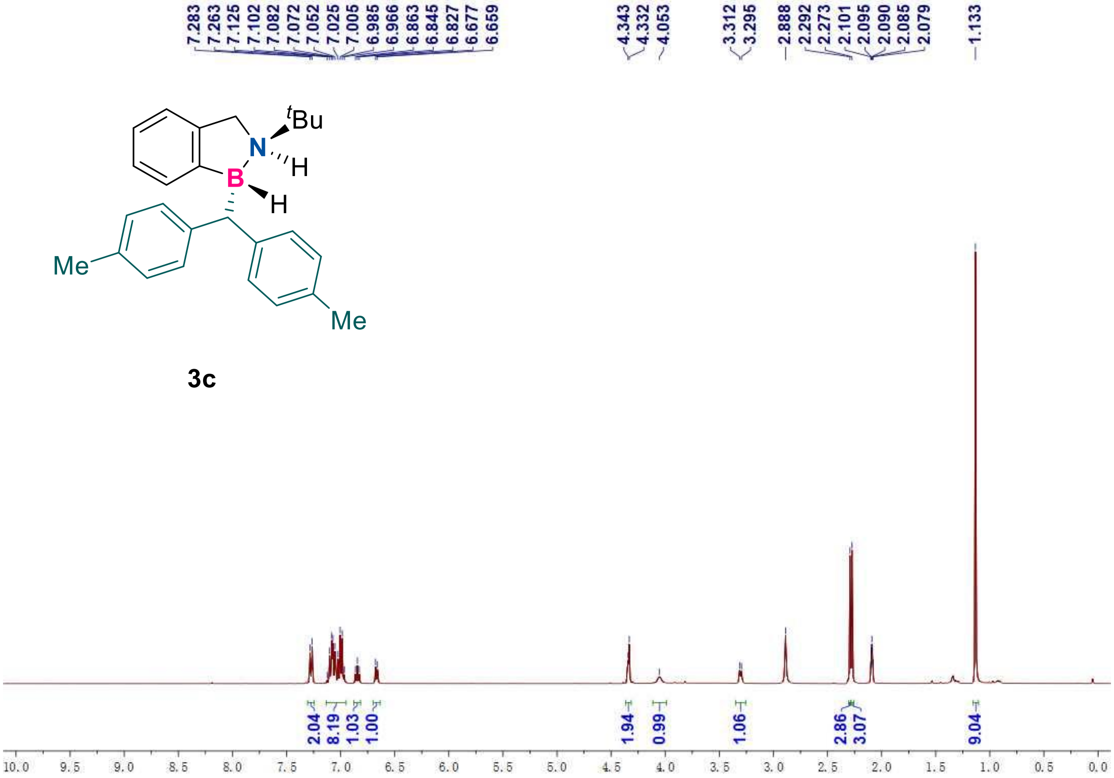

^{13}C NMR (126 MHz, $CDCl_3$)

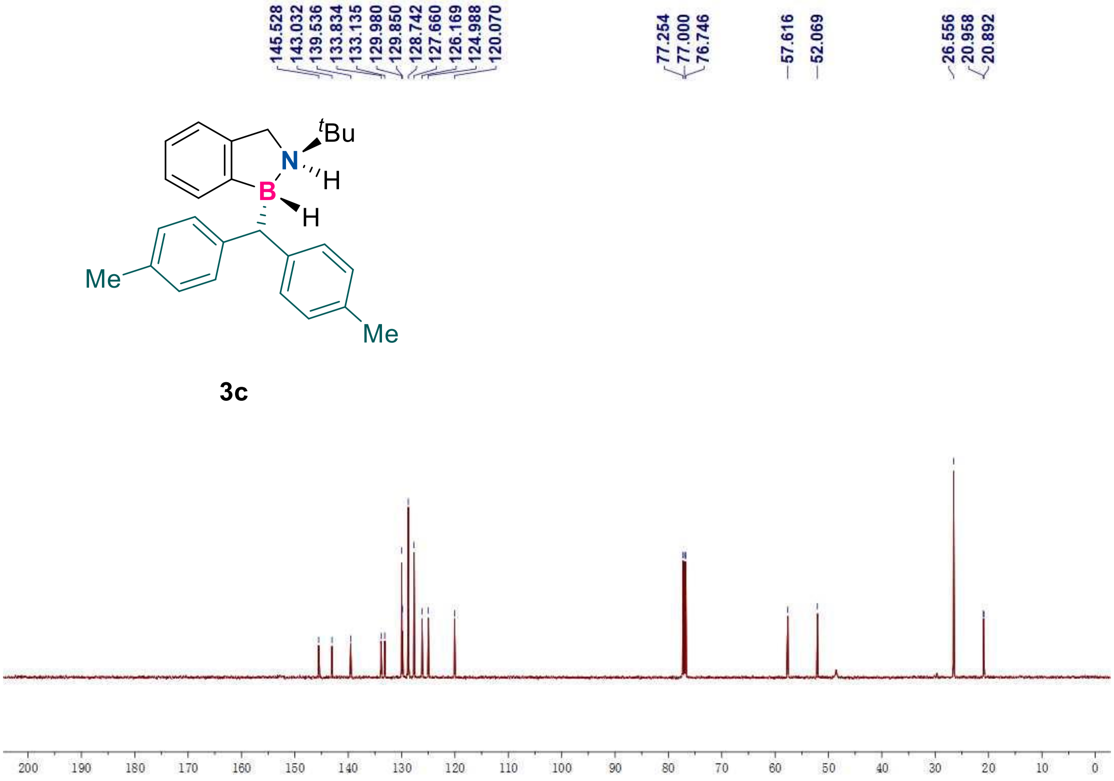

^{11}B NMR (160 MHz, $CDCl_3$)

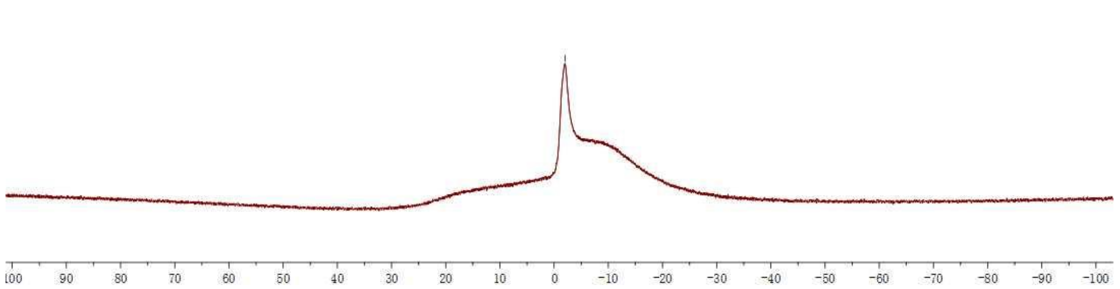

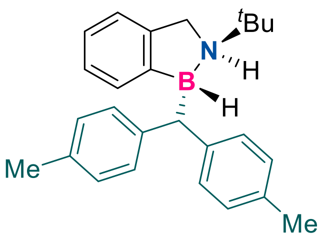

3c

1-(bis(4-fluorophenyl)methyl)-2-(tert-butyl)-2,3-dihydro-1H-benzo[c][1,2]azaborole (3d)

^{1}H NMR (500 MHz, $CDCl_3$)

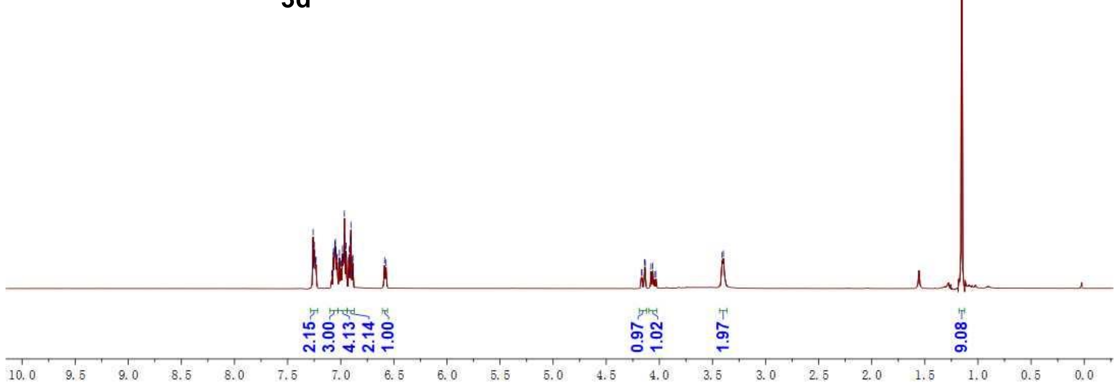

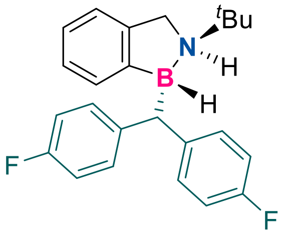

3d

^{13}C NMR (126 MHz, $CDCl_3$)

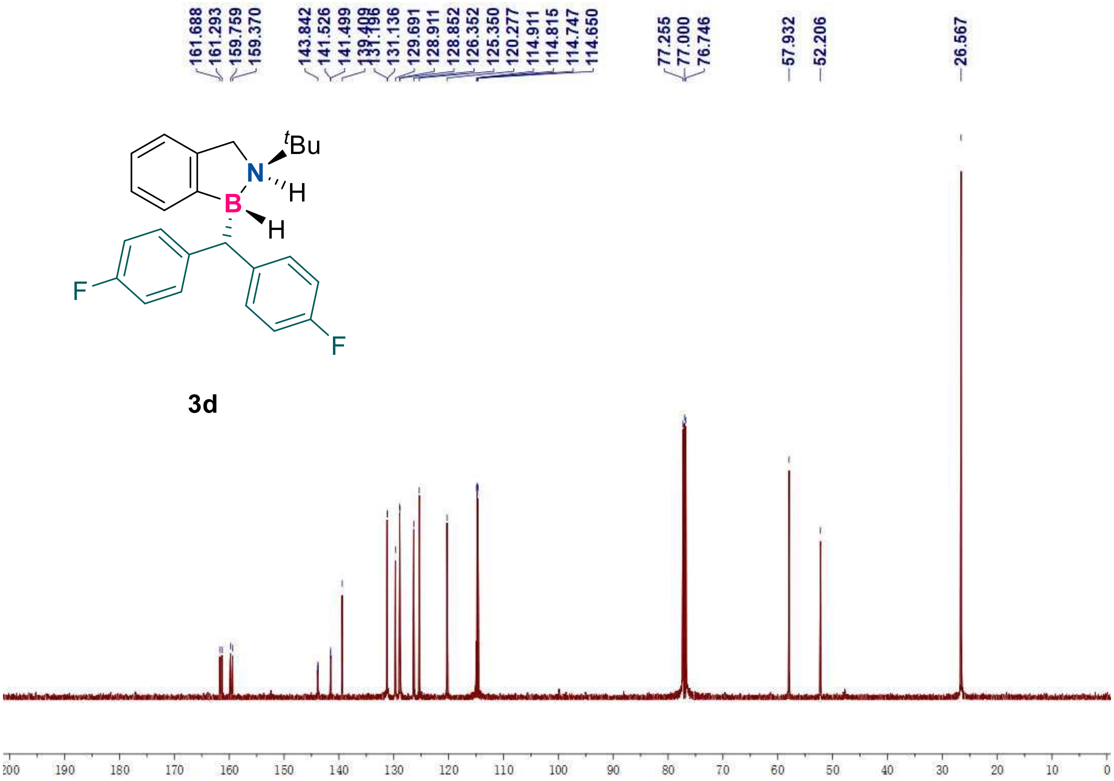

^{11}B NMR (160 MHz, $CDCl_3$)

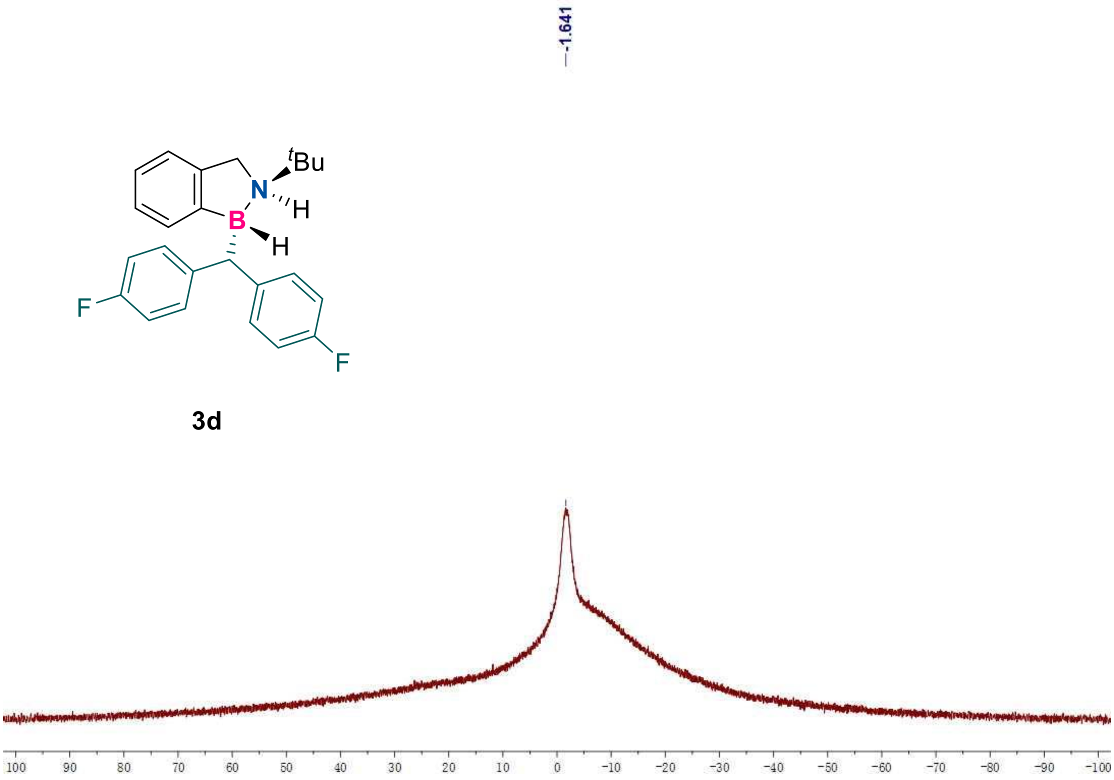

^{19}F NMR (471 MHz, $CDCl_3$)

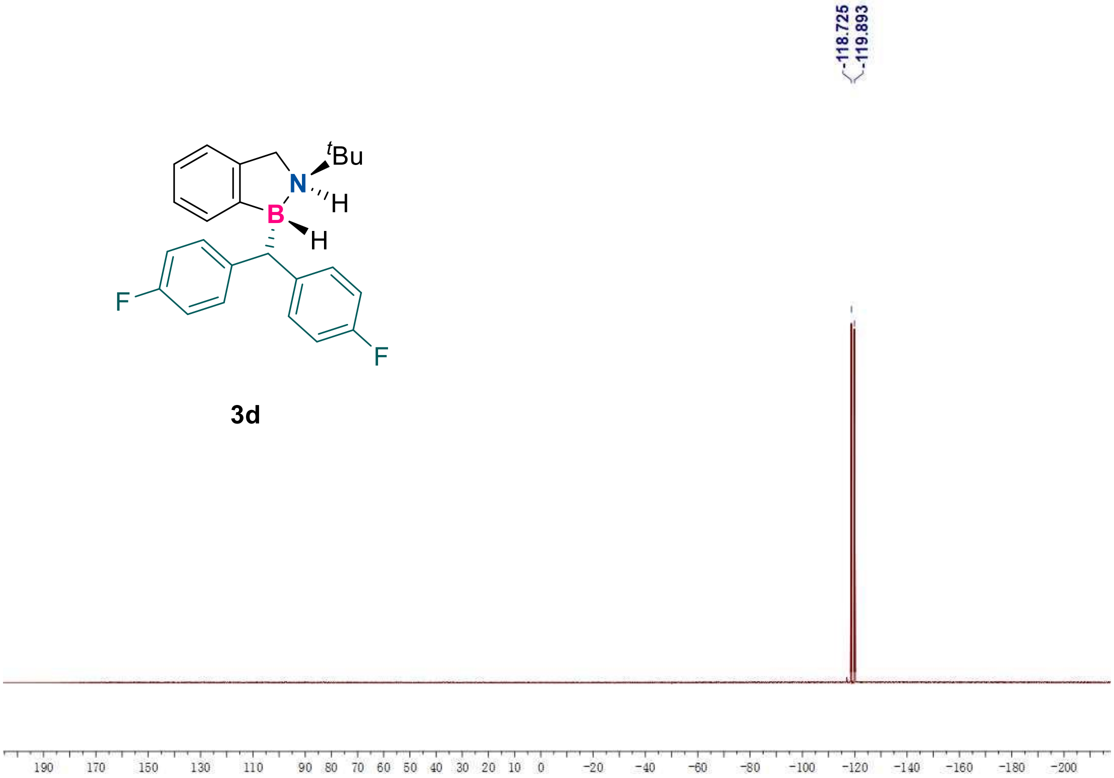

1-(bis(3-methoxyphenyl)methyl)-2-(tert-butyl)-2,3-dihydro-1H-benzo[c][1,2]azaborole (3e)

^{1}H NMR (500 MHz, Acetone-d_6)

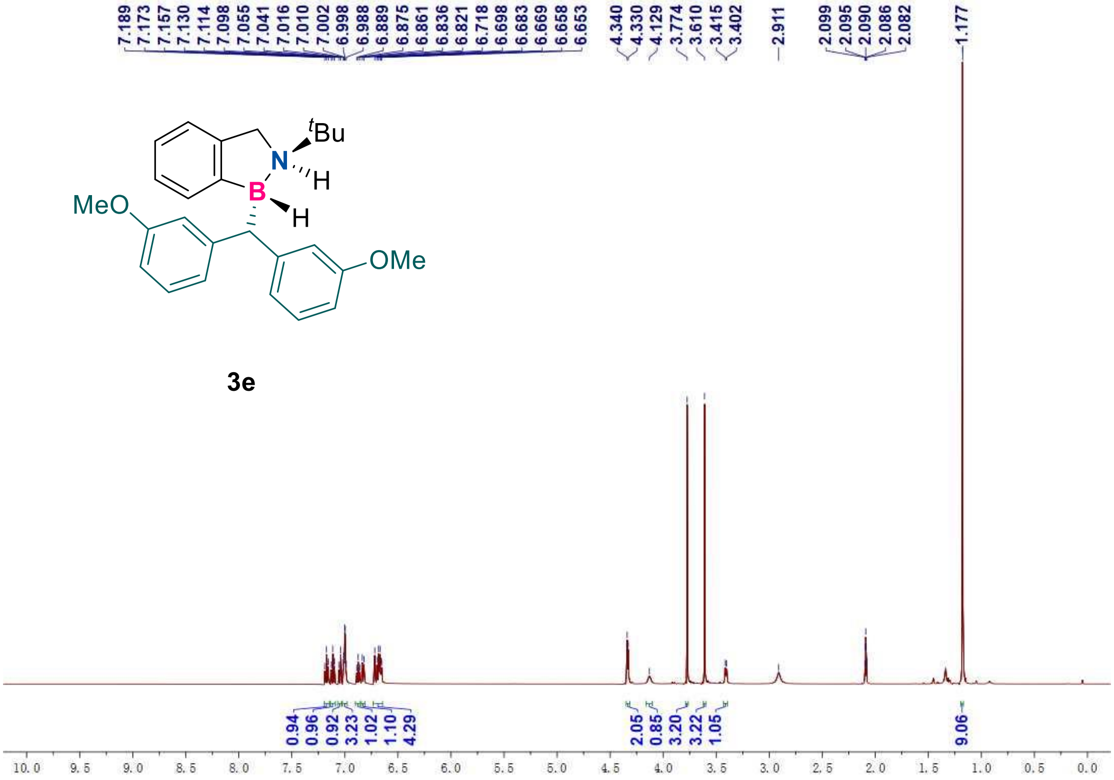

^{13}C NMR (126 MHz, Acetone-d_6)

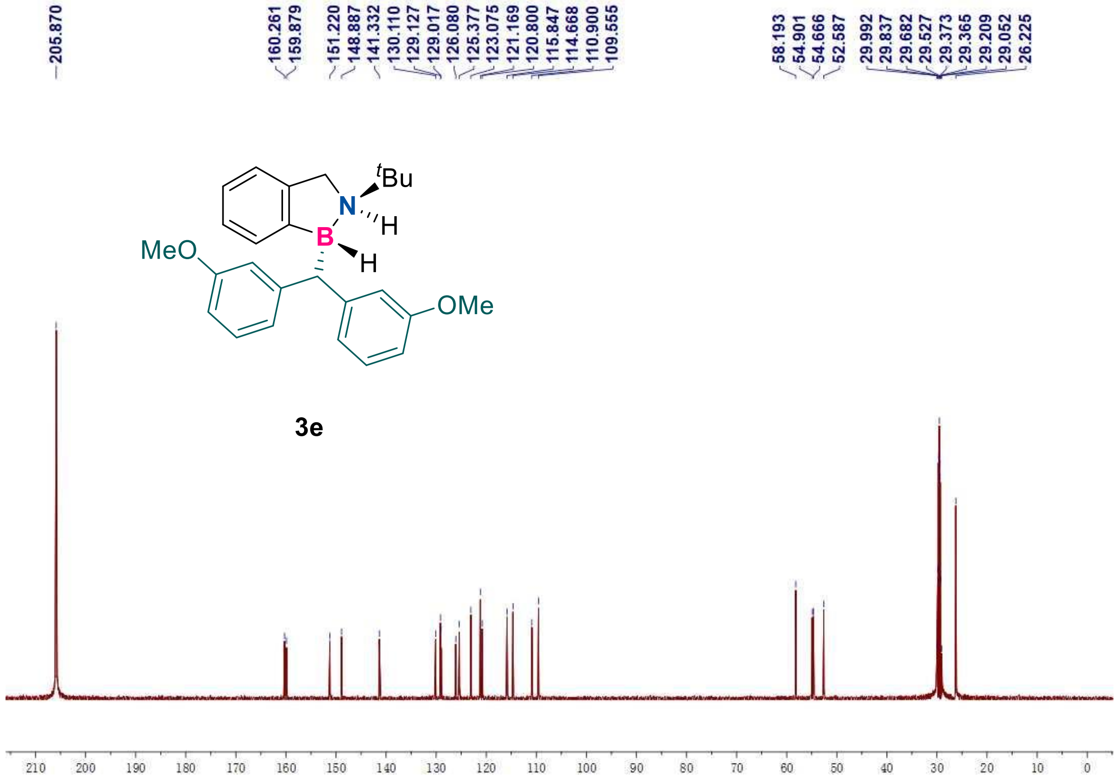

3e

^{11}B NMR (160 MHz, Acetone-d_6)

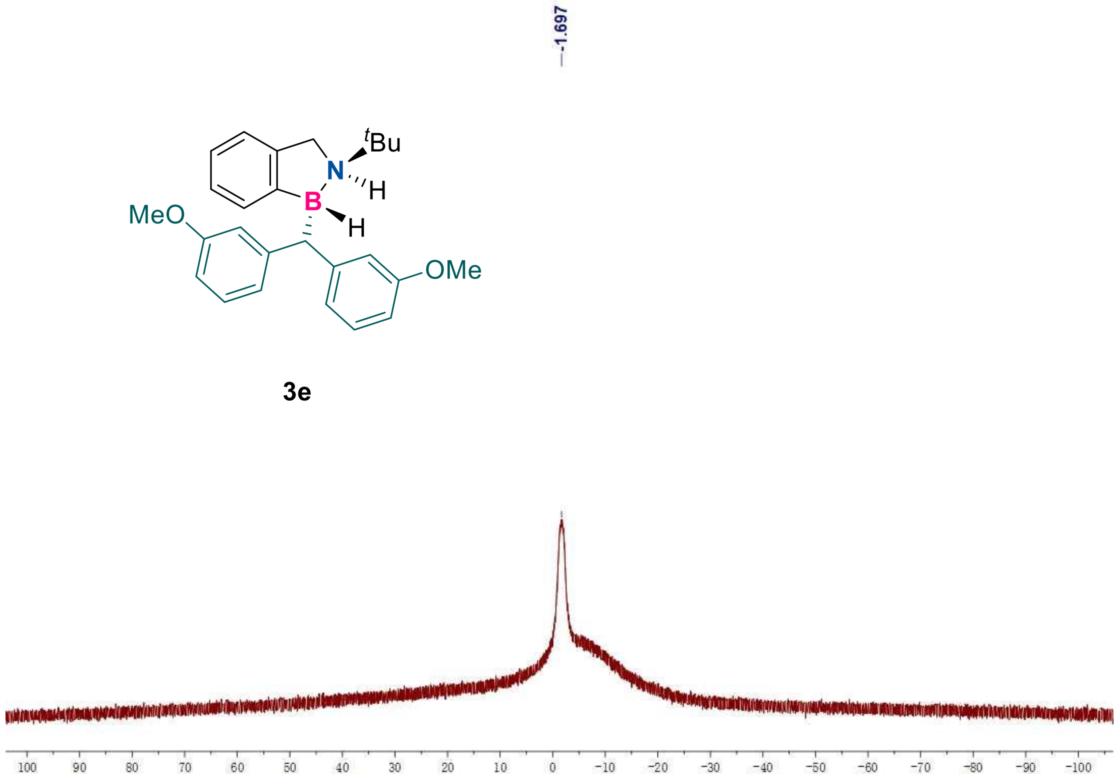

3e

2-(tert-butyl)-1-(di-m-tolylmethyl)-2,3-dihydro-1H-benzo[c][1,2]azaborole (3f)

^{1}H NMR (500 MHz, Acetone-d_6)

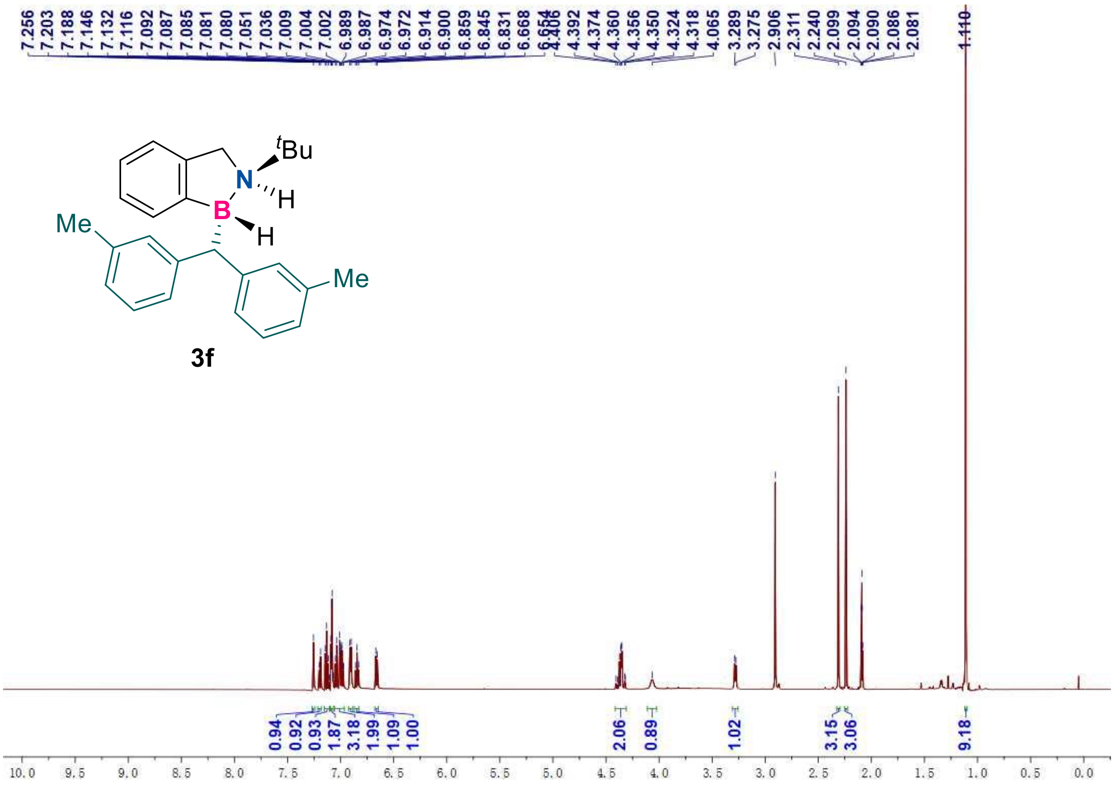

^{13}C NMR (126 MHz, Acetone-d_6)

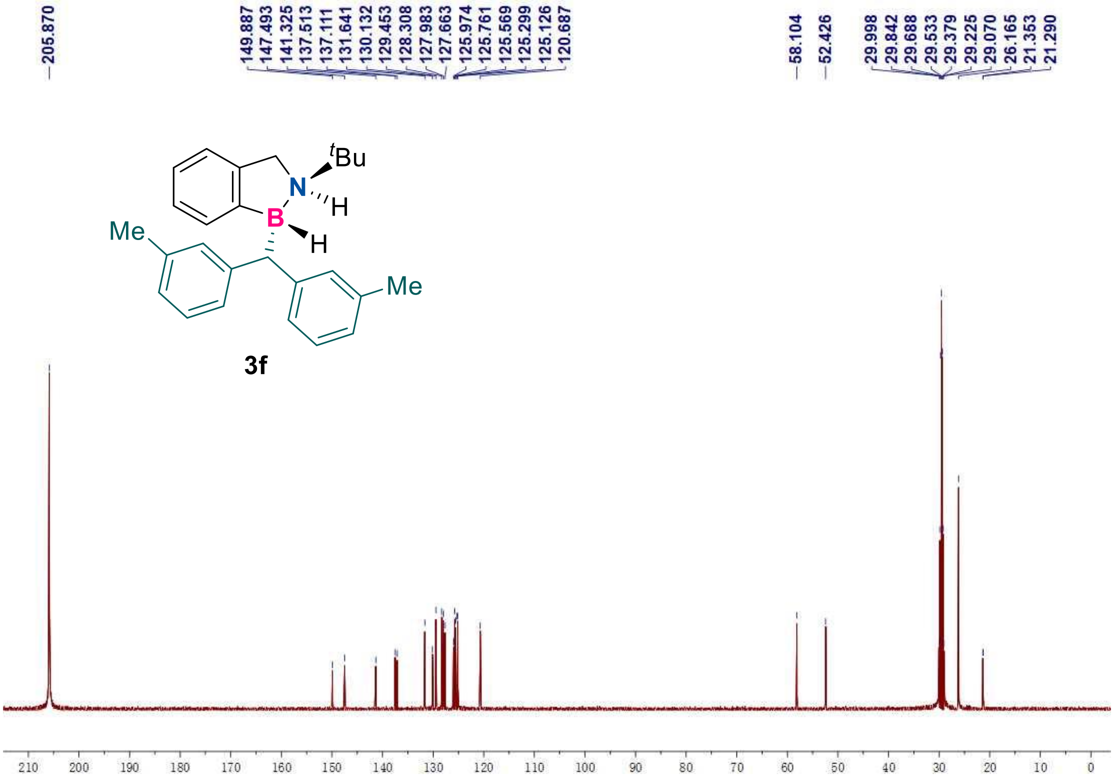

^{11}B NMR (160 MHz, Acetone-d_6)

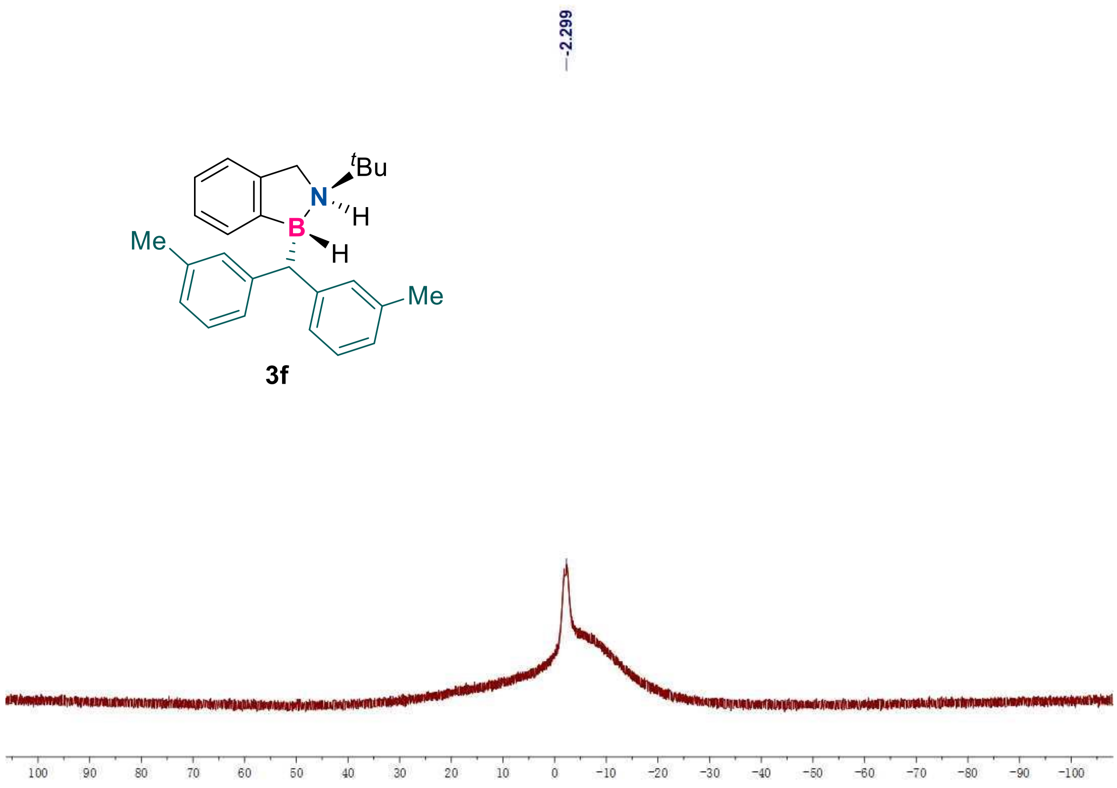

1-(bis(3,5-dimethoxyphenyl)methyl)-2-(tert-butyl)-2,3-dihydro-1*H*-benzo[c][1,2]azaborole (3g)

^{1}H NMR (500 MHz, Acetone-d_6)

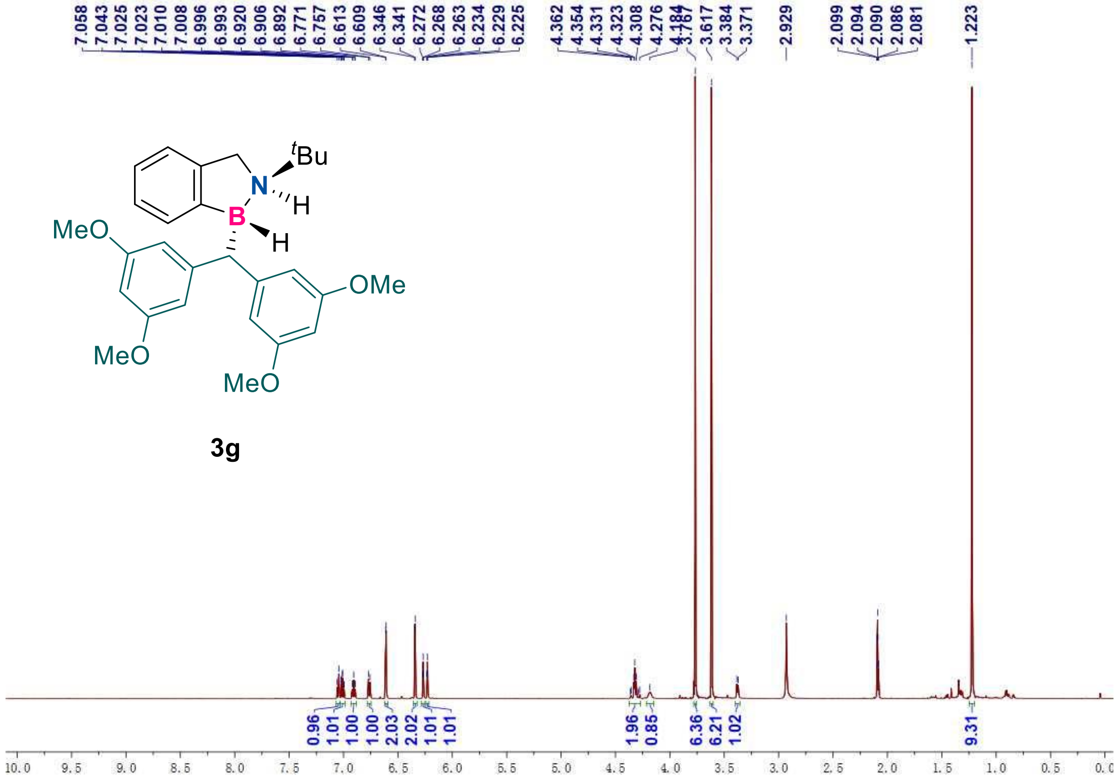

^{13}**C NMR** (101 MHz, Acetone-d_6)

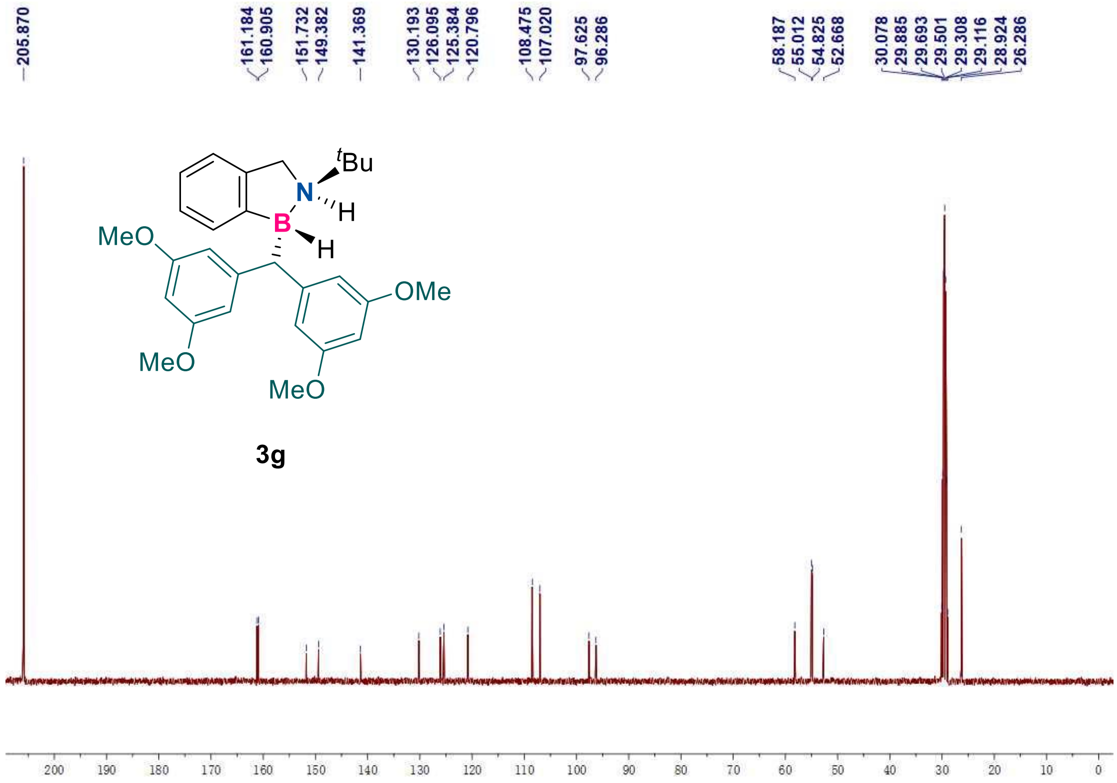

^{11}**B NMR** (160 MHz, Acetone-d_6)

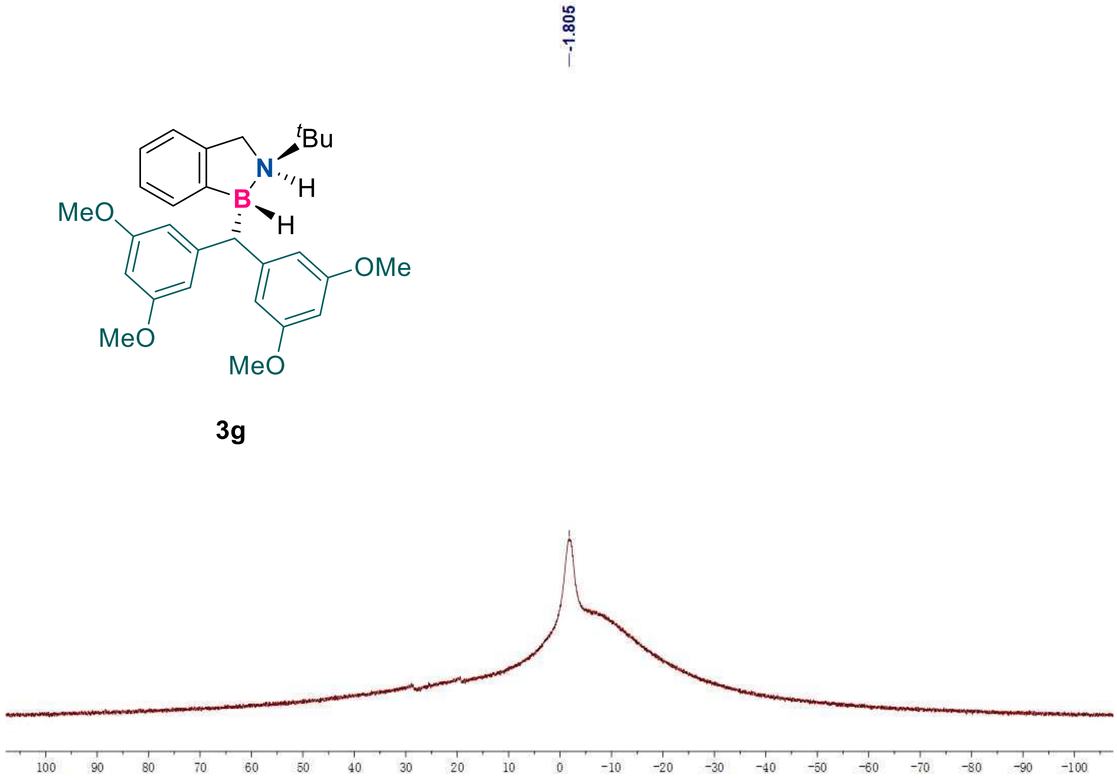

(R)-4-((2-(tert-butyl)-2,3-dihydro-1*H*-benzo[c][1,2]azaborol-1-yl)(phenyl)methyl)benzonitrile

(**3h**)

^{1}H NMR (500 MHz, $CDCl_3$)

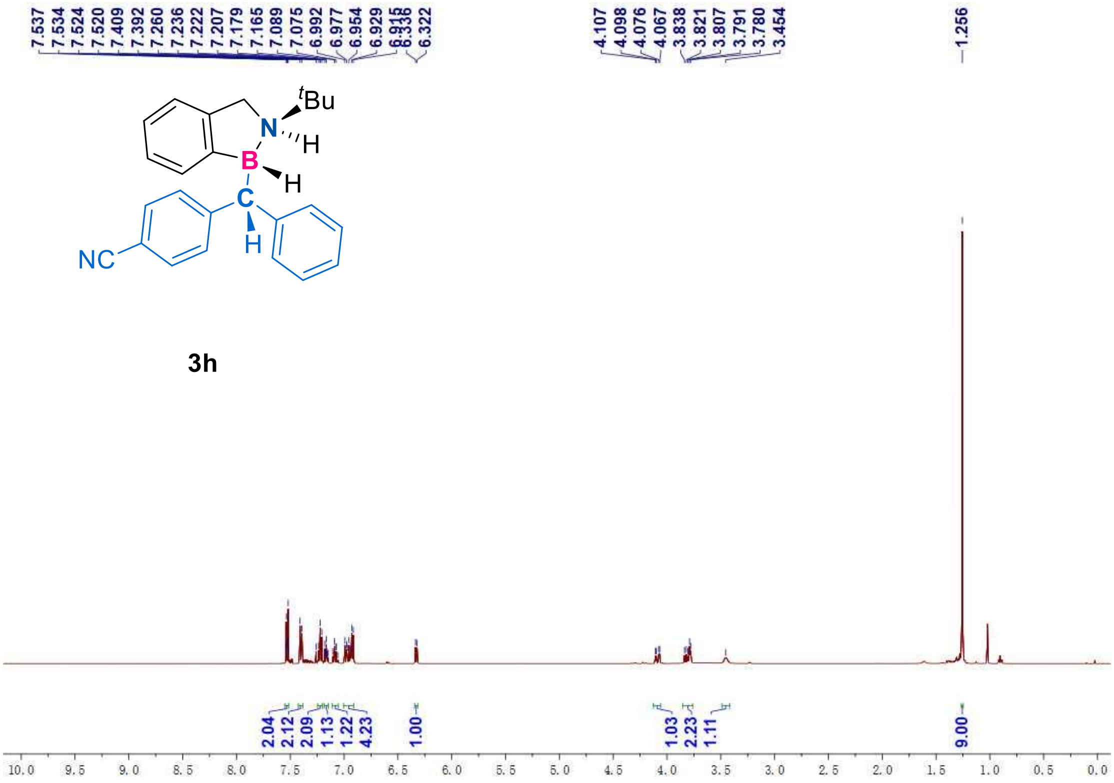

^{13}C NMR (126 MHz, $CDCl_3$)

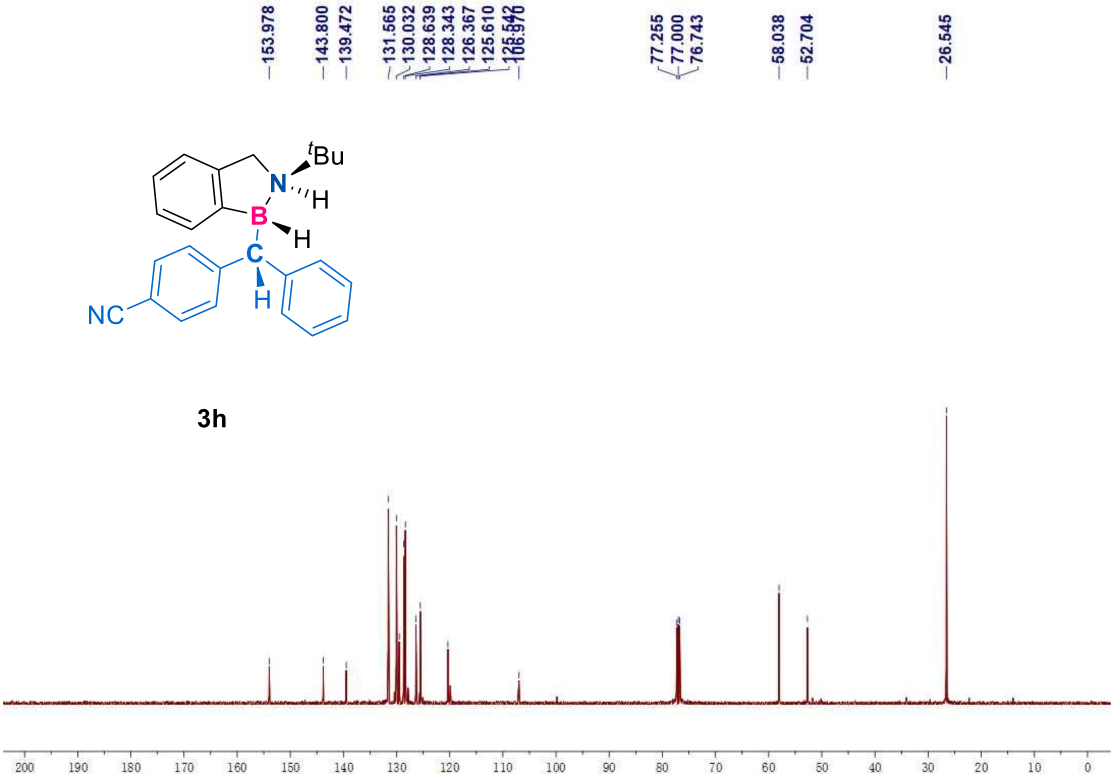

^{11}B NMR (160 MHz, $CDCl_3$)

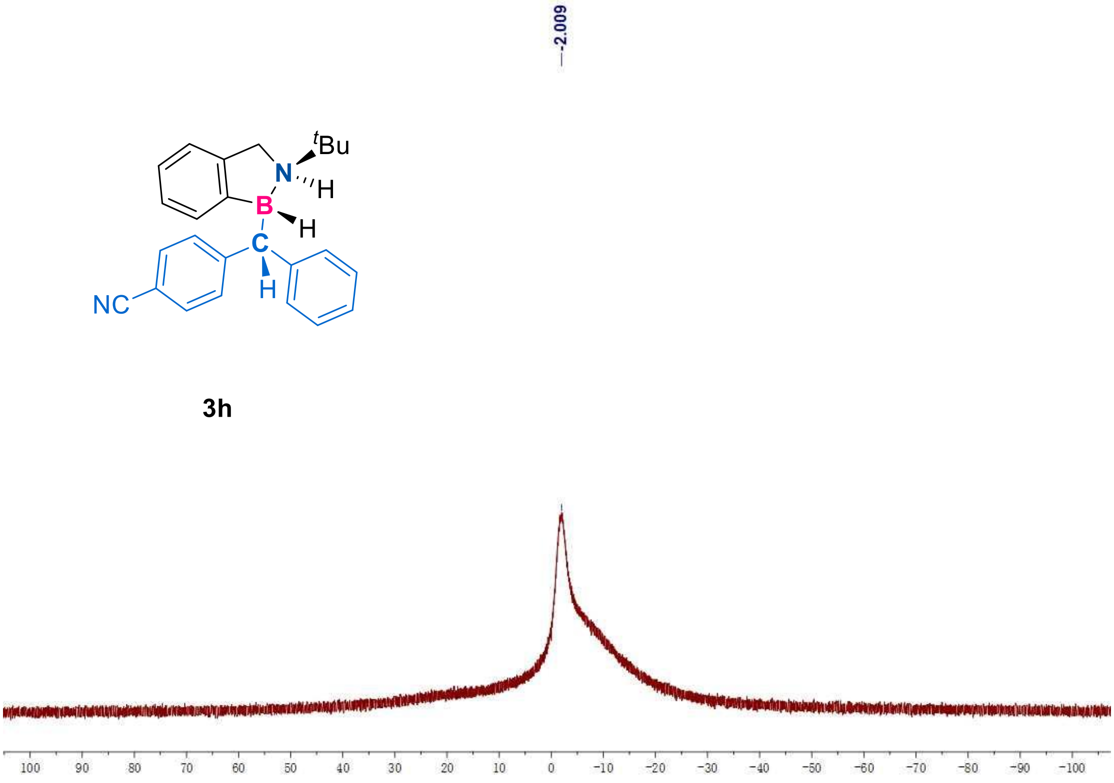

(R)-2-(tert-butyl)-1-((4-nitrophenyl)(phenyl)methyl)-2,3-dihydro-1H-benzo[c][1,2]azaborole (3i)

^{1}H NMR (400 MHz, $CDCl_3$)

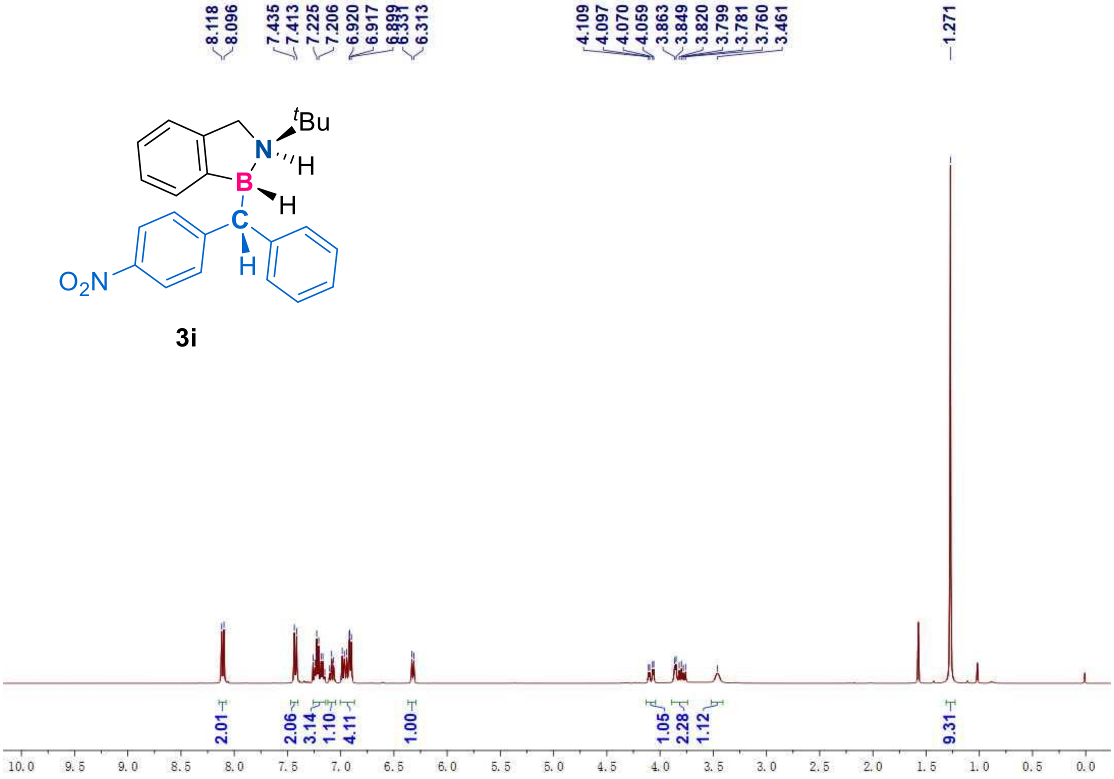

^{13}C NMR (101 MHz, $CDCl_3$)

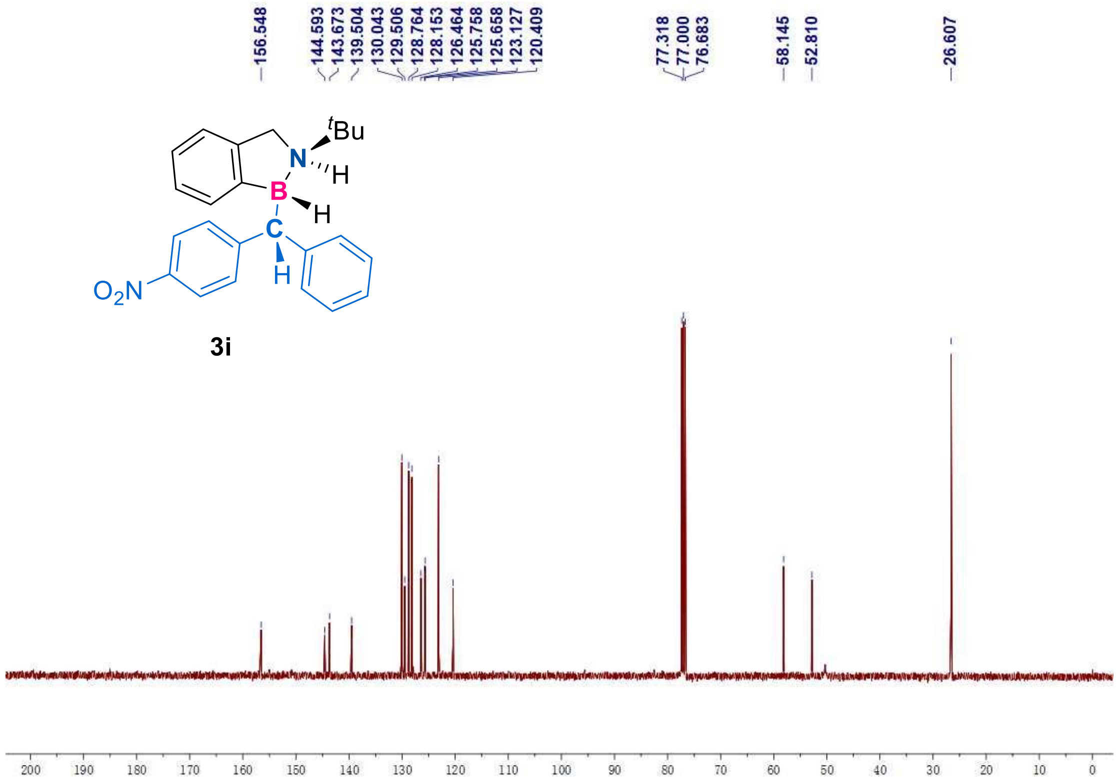

^{11}B NMR (128 MHz, $CDCl_3$)

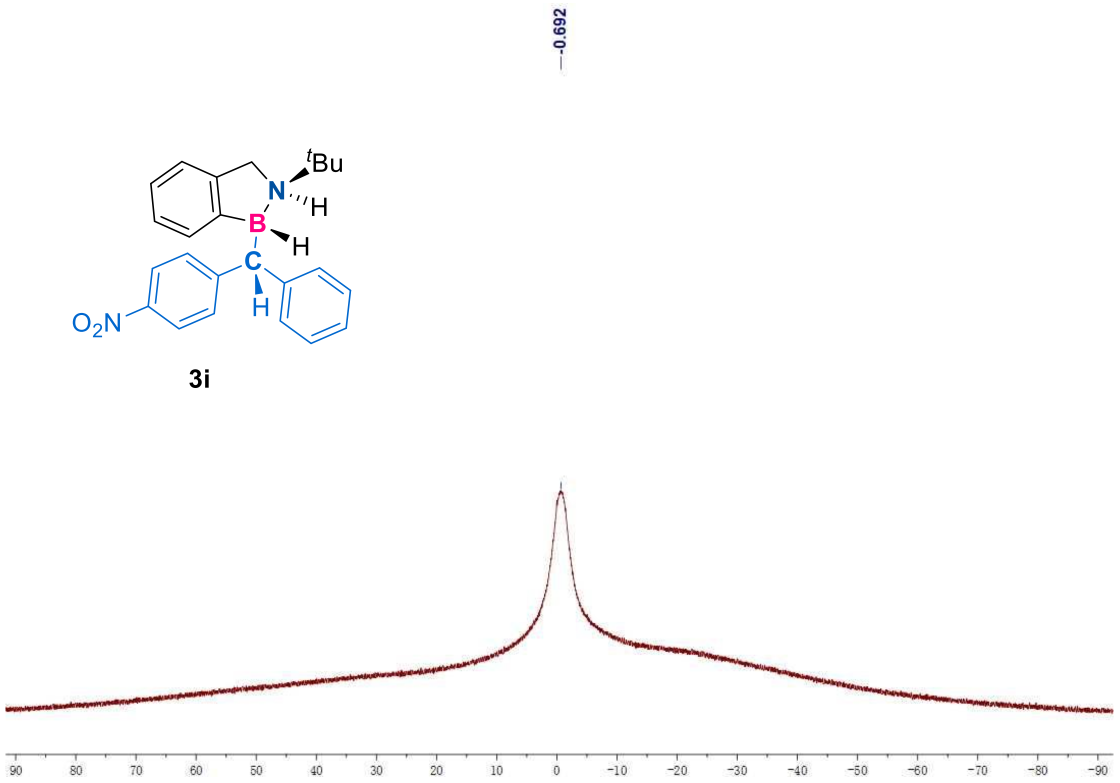

(S)-2-(tert-butyl)-1-((4-methoxyphenyl)(phenyl)methyl)-2,3-dihydro-1H-benzo[c][1,2]azaborole (3j)

^{1}H NMR (500 MHz, $CDCl_3$)

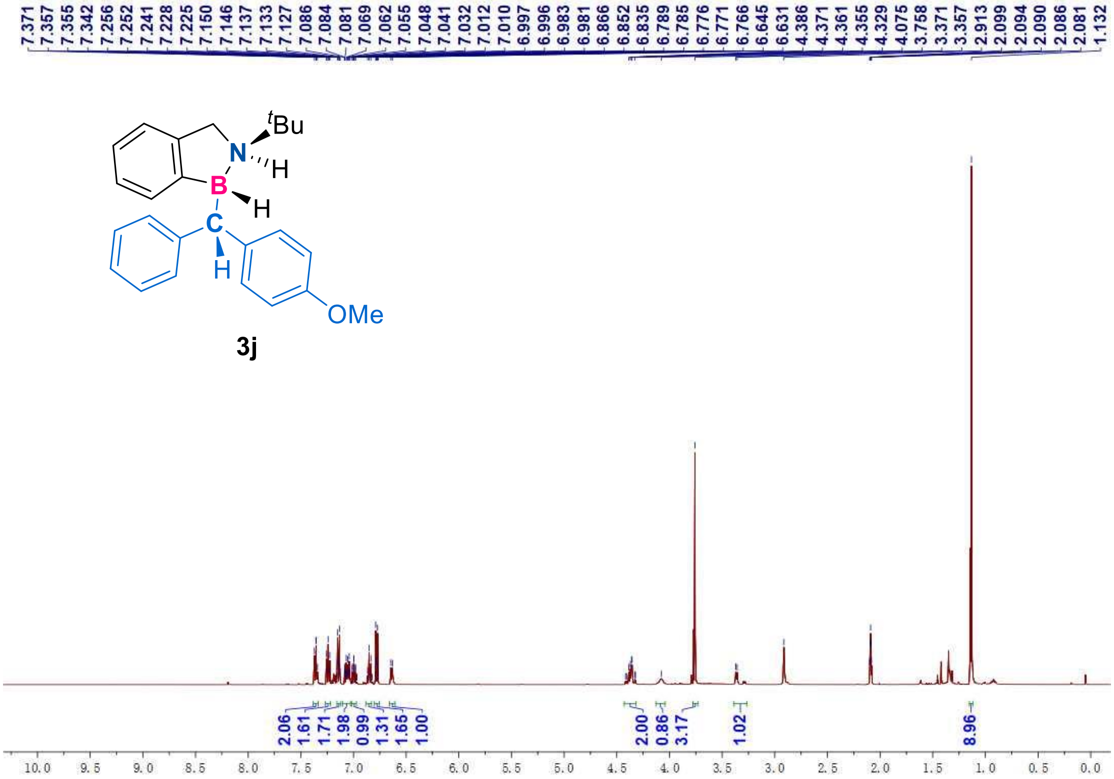

^{13}C NMR (126 MHz, $CDCl_3$)

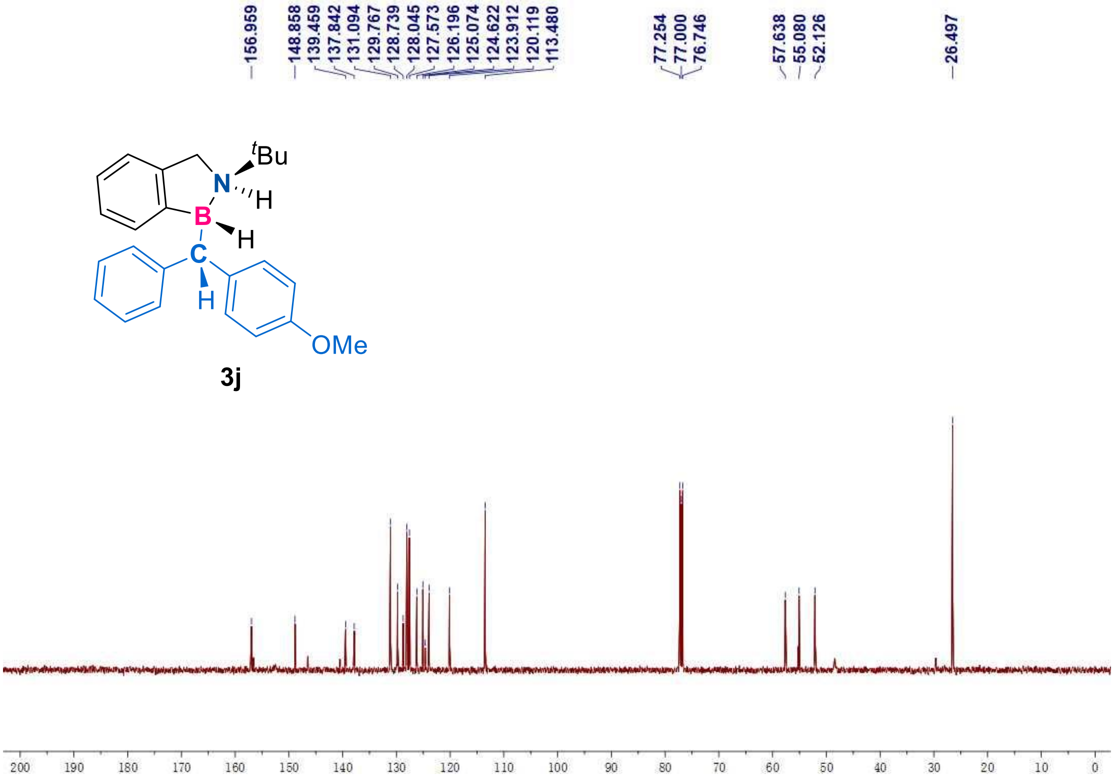

^{11}B NMR (160 MHz, $CDCl_3$)

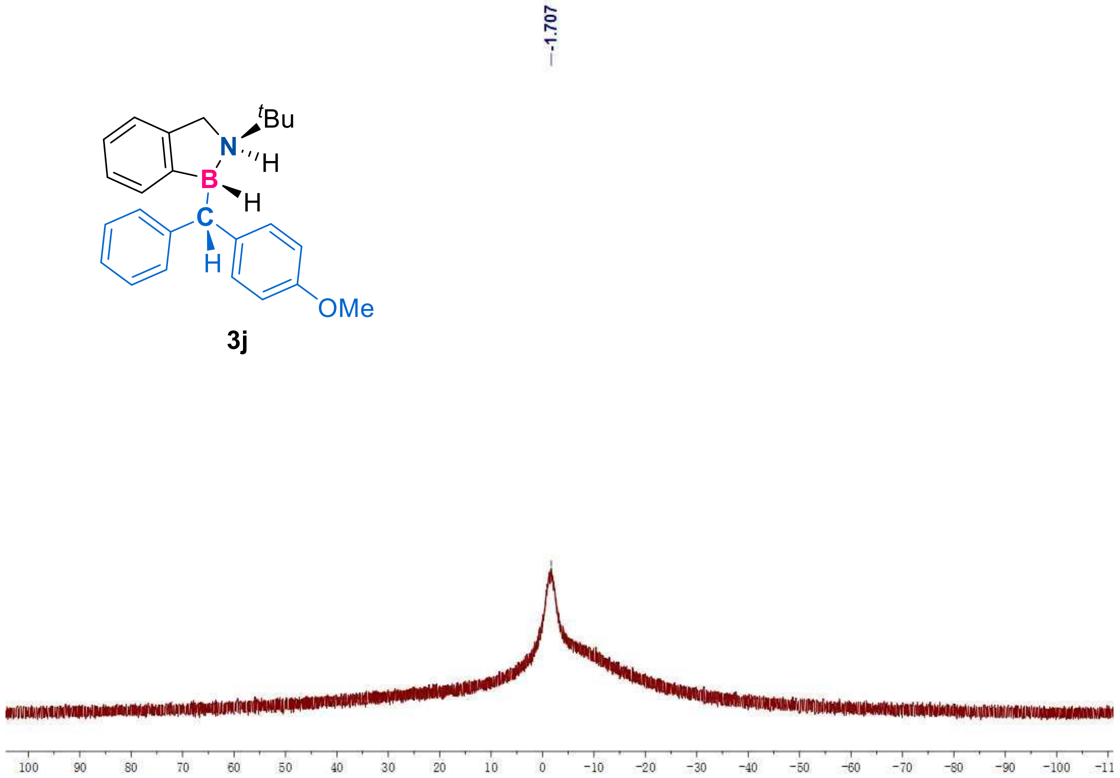

(R)-2-(tert-butyl)-1-((3-chloro-4-fluorophenyl)(4-methoxyphenyl)methyl)-2,3-dihydro-1*H*-benzo[c][1,2]azaborole (**3k**)

^{1}H NMR (500 MHz, $CDCl_3$)

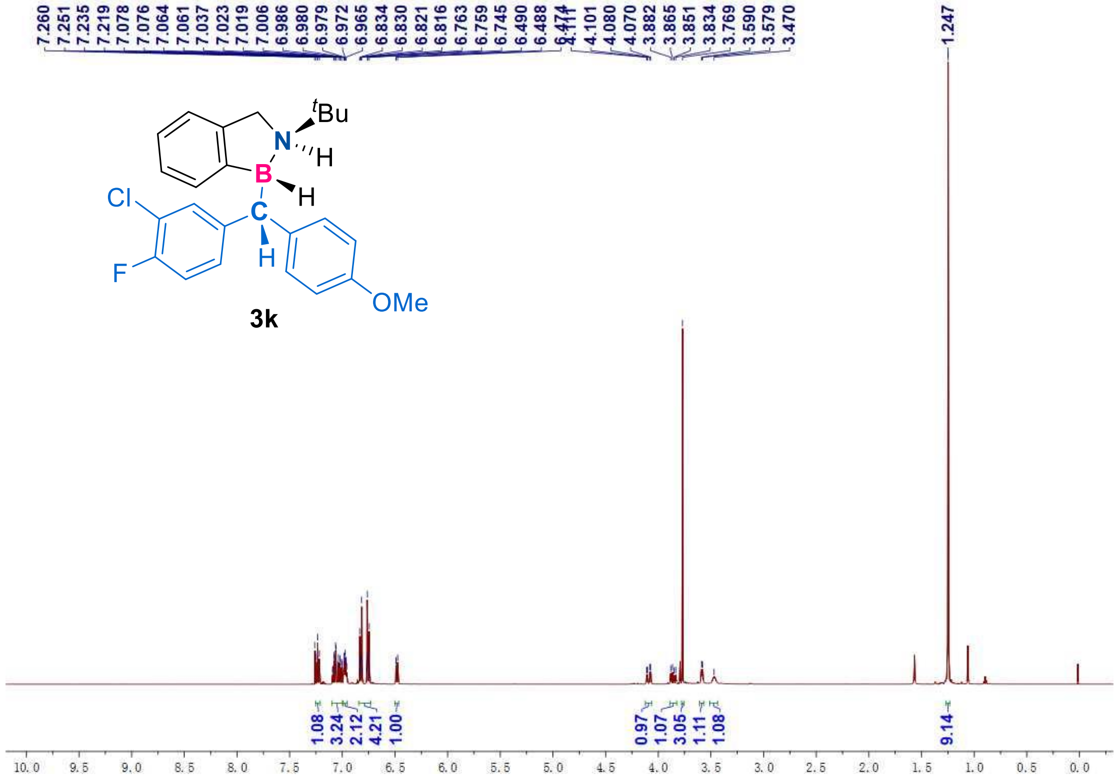

^{13}C NMR (126 MHz, $CDCl_3$)

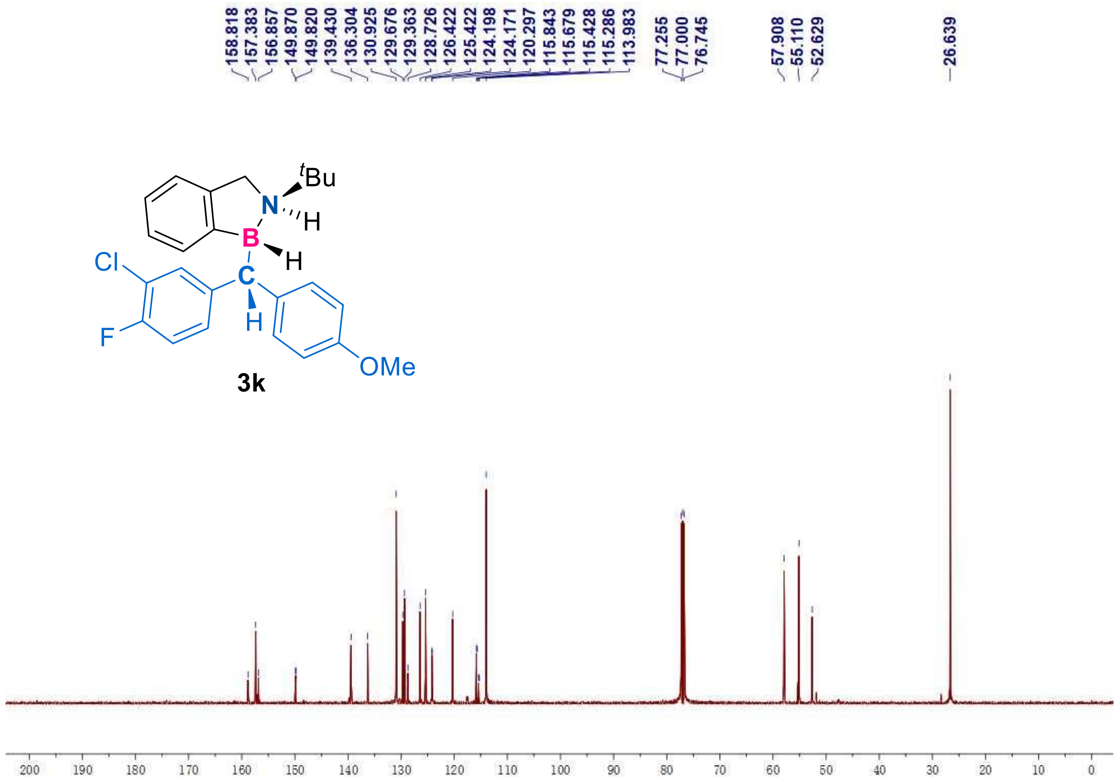

^{11}B NMR (160 MHz, $CDCl_3$)

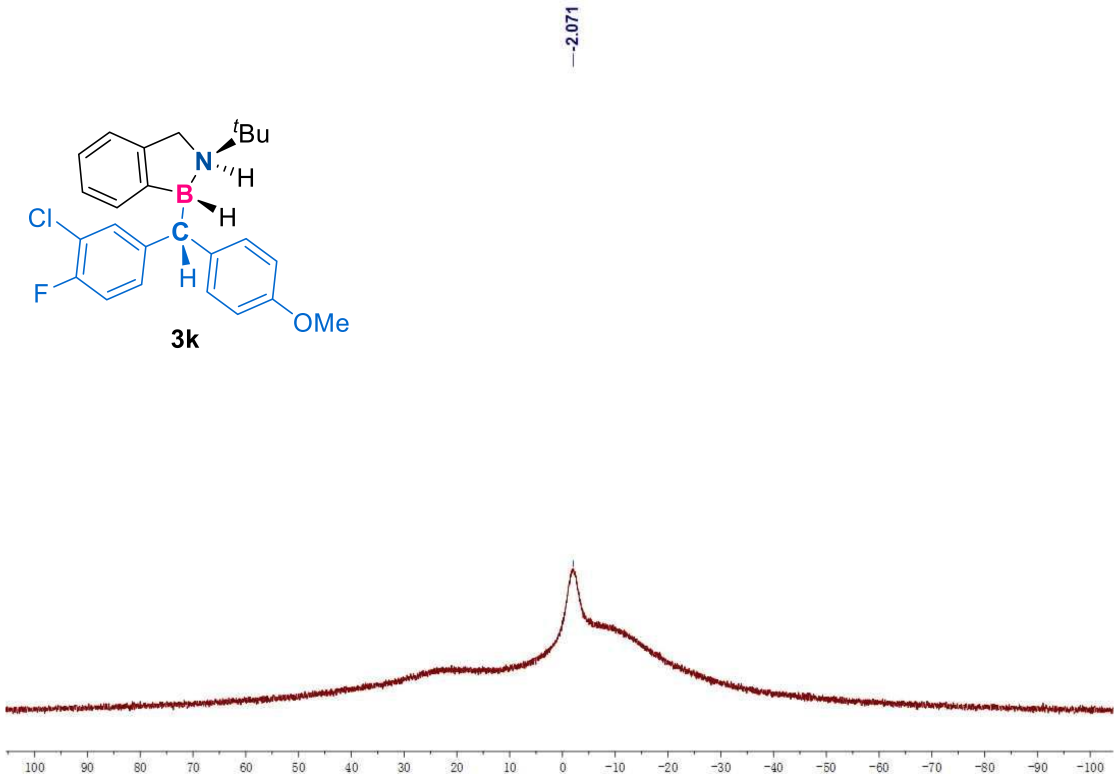

^{19}F NMR (471 MHz, $CDCl_3$)

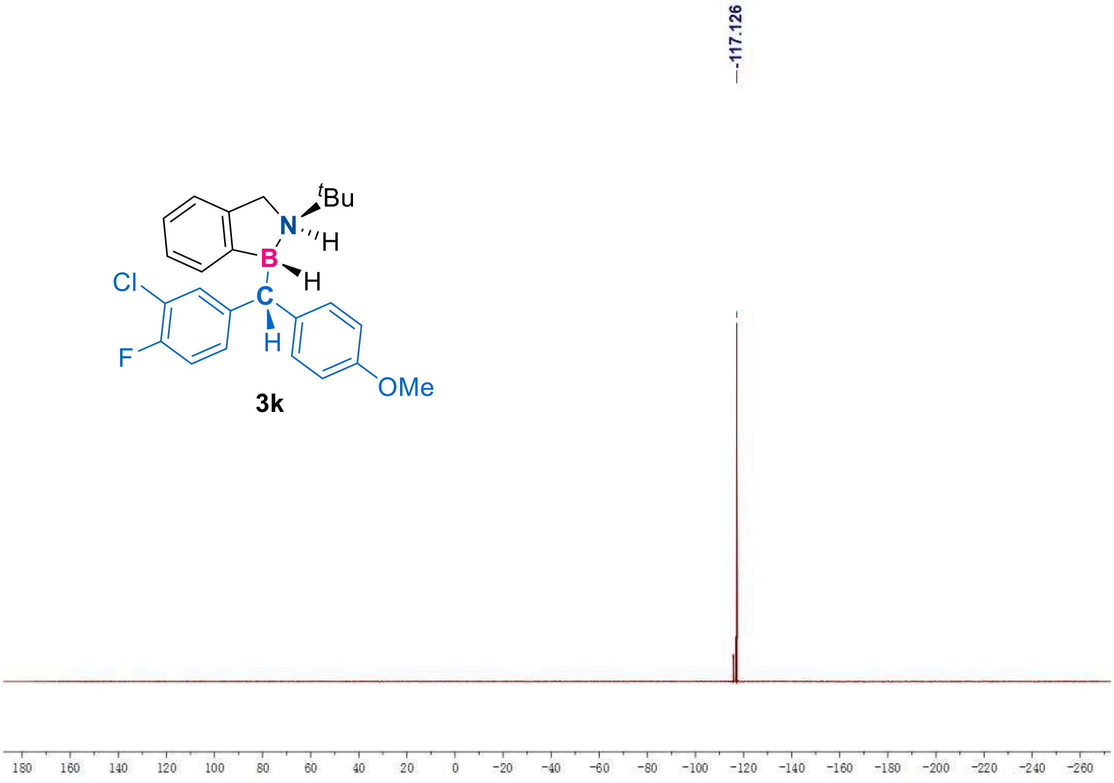

(R)-2-(tert-butyl)-1-((3,4-dichlorophenyl)(4-methoxyphenyl)methyl)-2,3-dihydro-1H-benzo[c][1,2]azaborole (3l)

^{1}H NMR (500 MHz, Acetone-d_6)

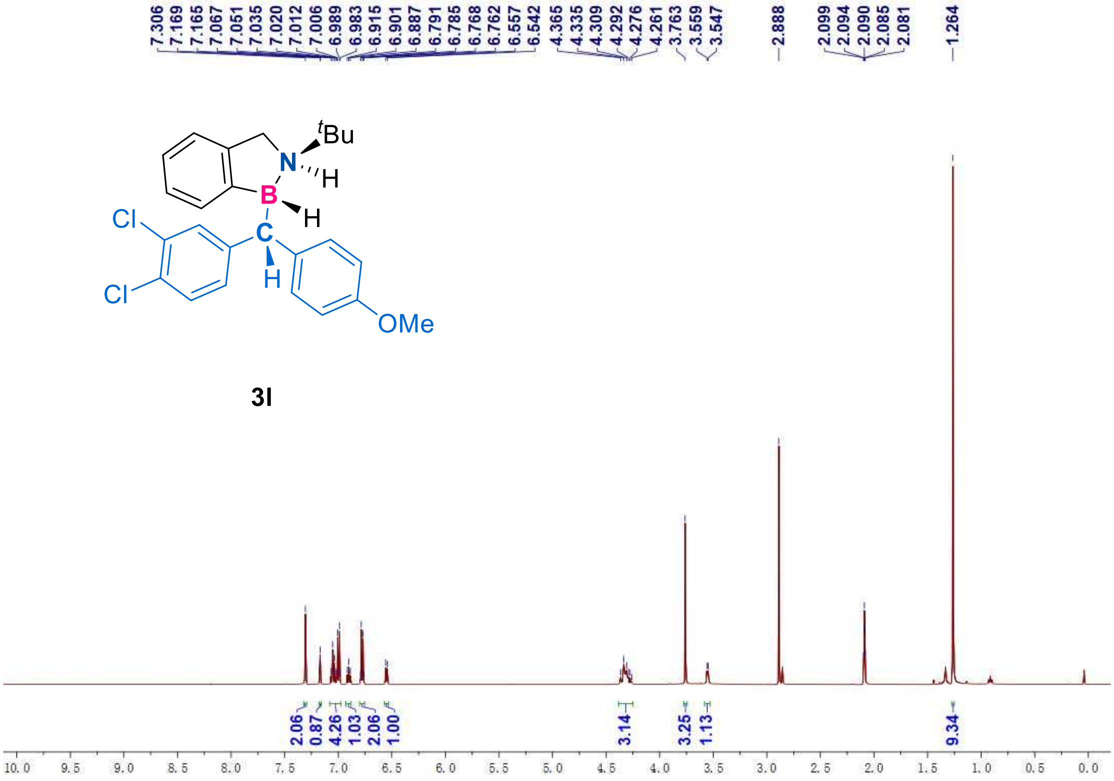

^{13}C NMR (126 MHz, Acetone-d_6)

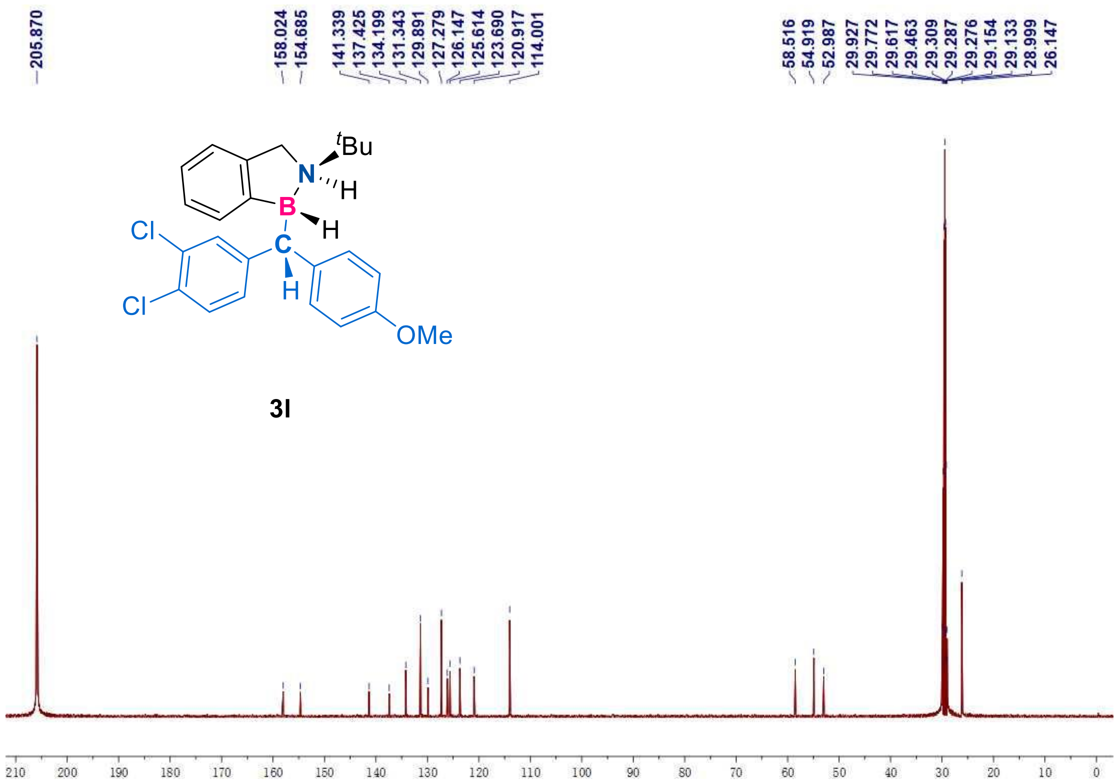

^{11}B NMR (160 MHz, Acetone-d_6)

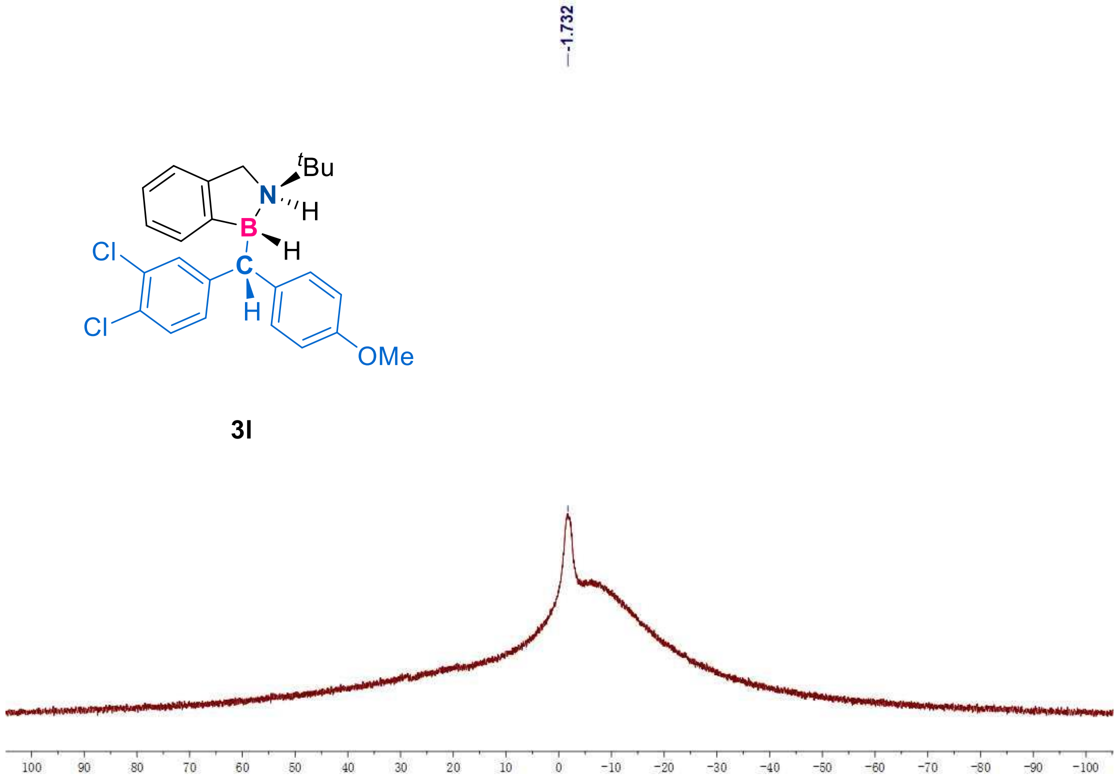

(R)-2-(tert-butyl)-1-((3,5-difluorophenyl)(4-methoxyphenyl)methyl)-2,3-dihydro-1*H*-

benzo[c][1,2]azaborole (3m)

^{1}H NMR (500 MHz, $CDCl_3$)

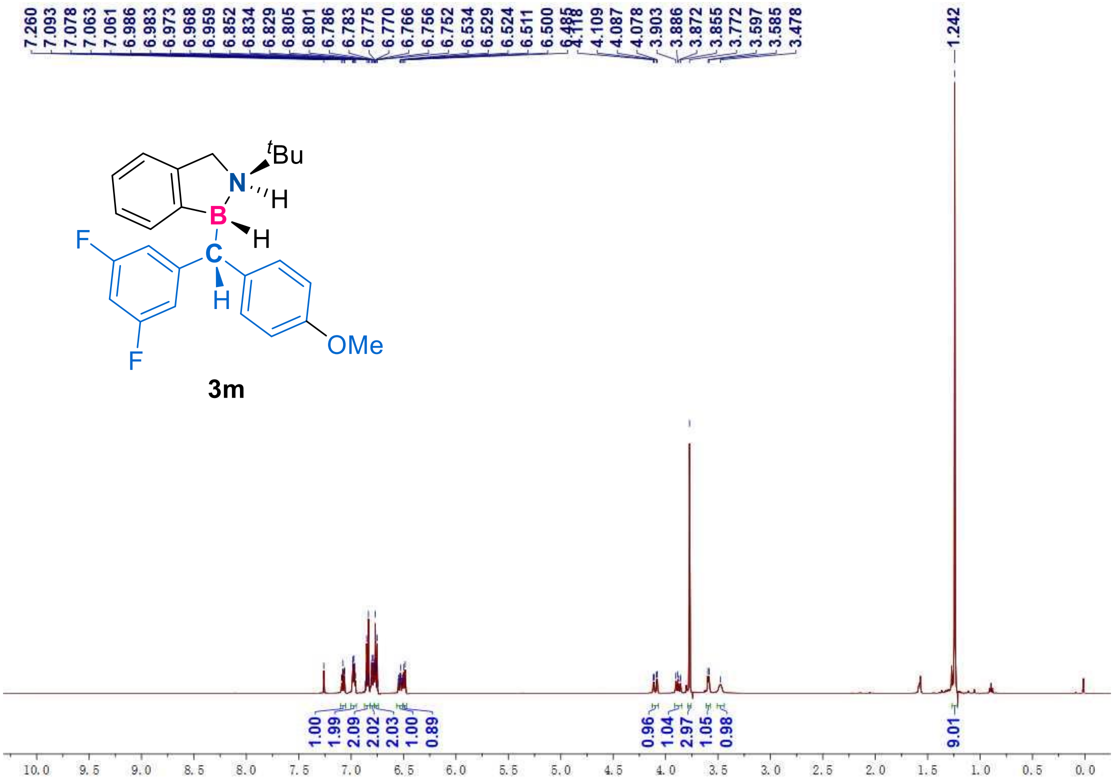

^{13}C NMR (126 MHz, $CDCl_3$)

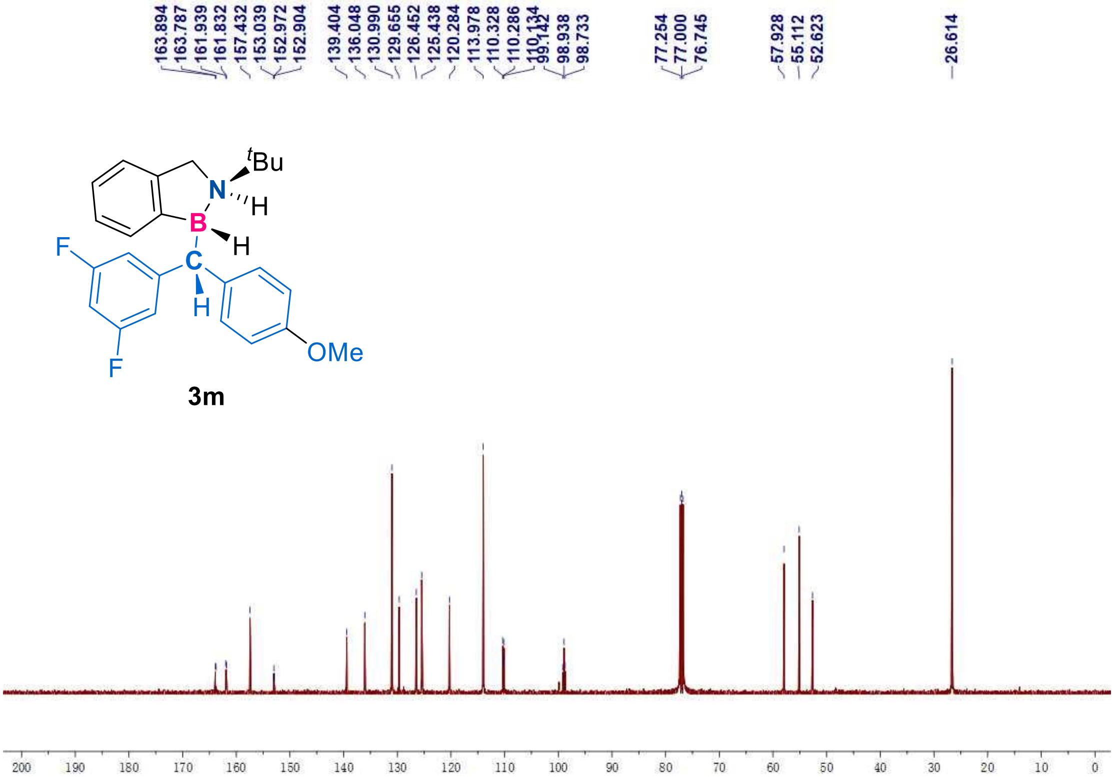

^{11}B NMR (160 MHz, CDCl$_3$)

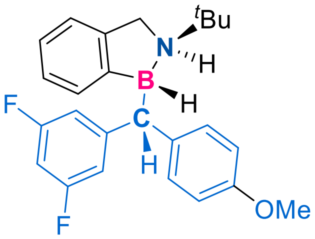

3m

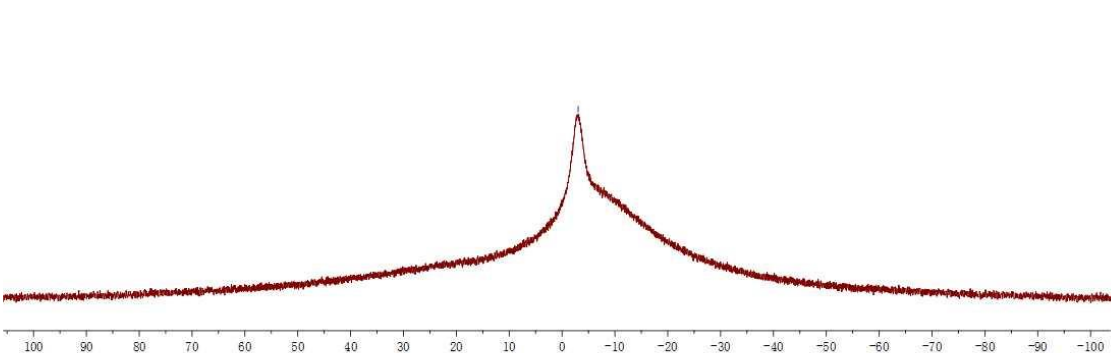

^{19}F NMR (471 MHz, CDCl$_3$)

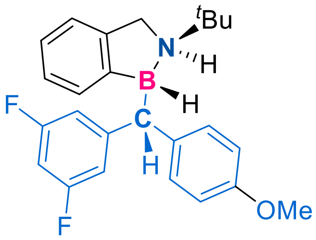

3m

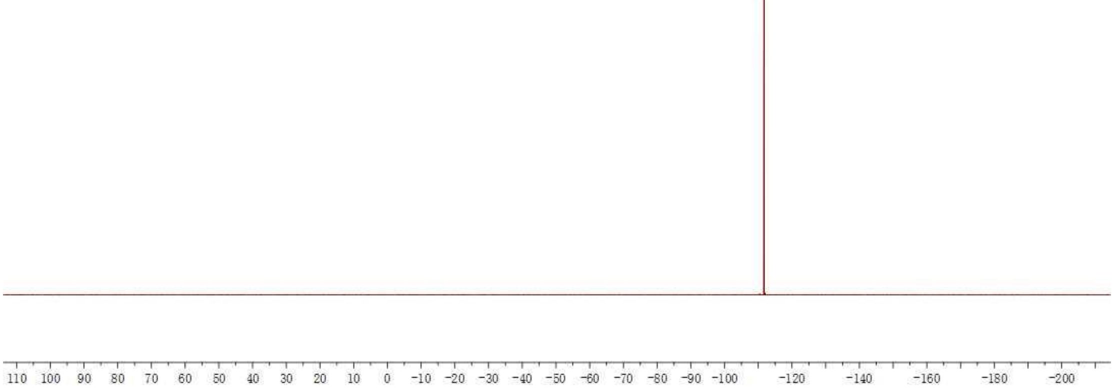

(R)-2-(tert-butyl)-1-((4-fluorophenyl)(4-methoxyphenyl)methyl)-2,3-dihydro-1H-benzo[c][1,2]azaborole (**3n**)

^{1}**H NMR** (500 MHz, CDCl$_3$)

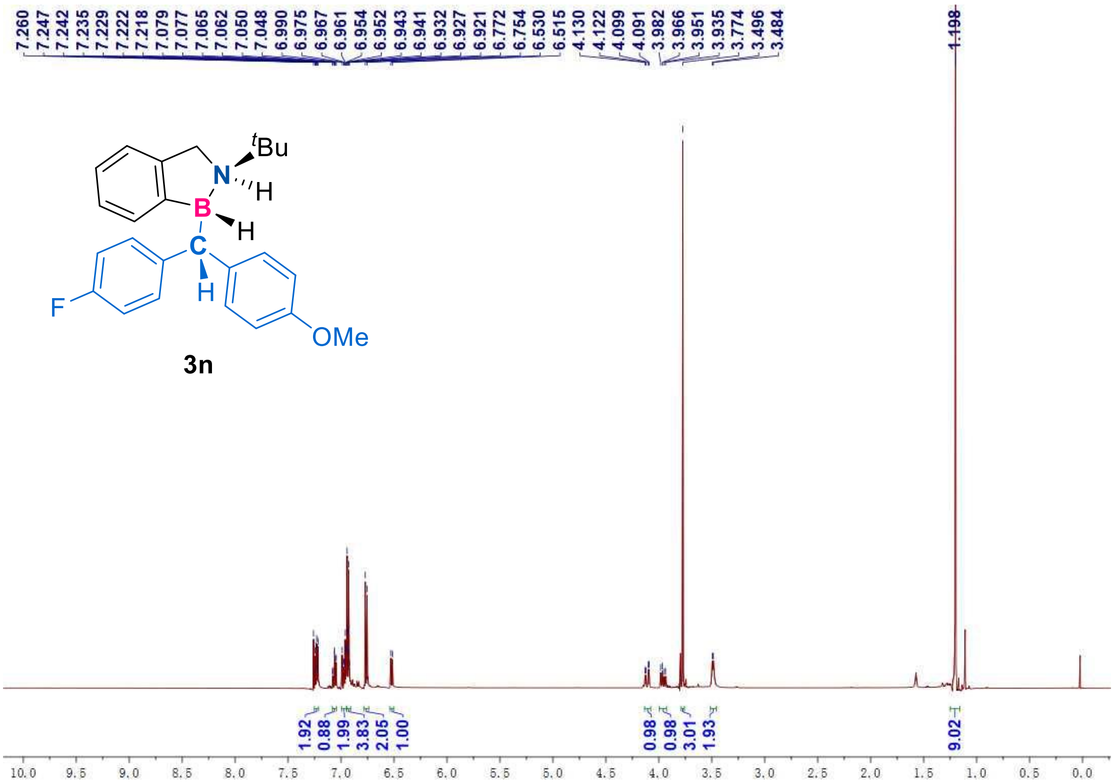

^{13}**C NMR** (126 MHz, CDCl$_3$)

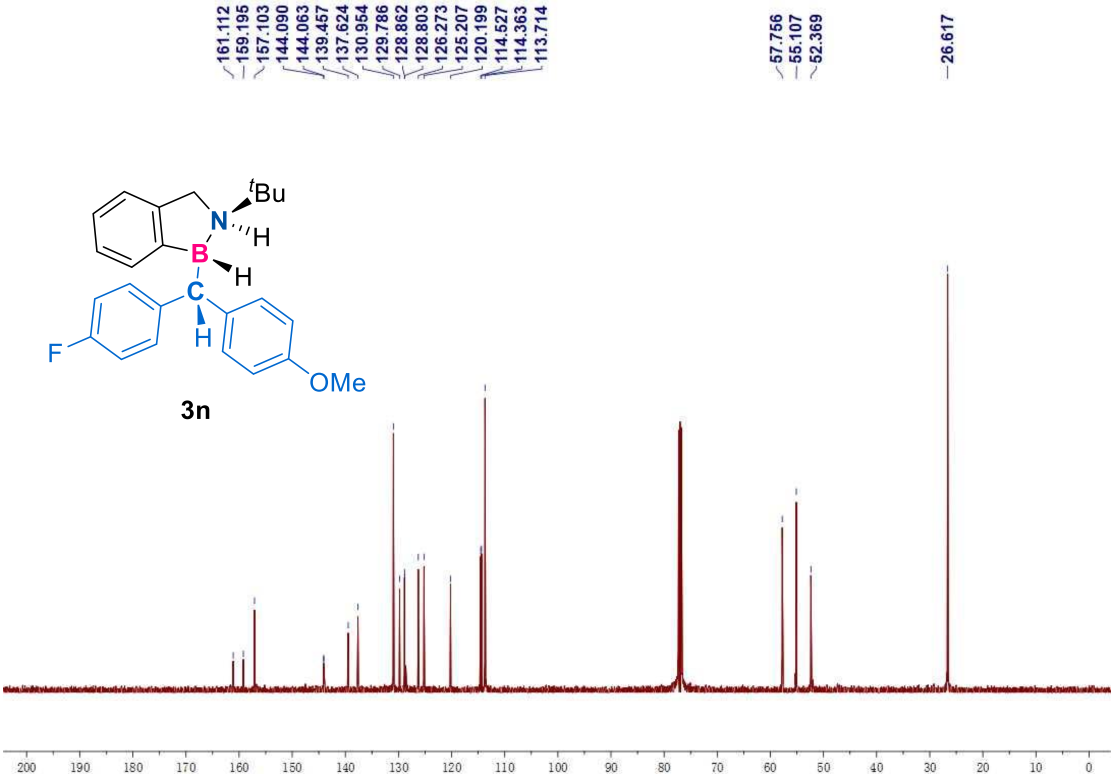

^{11}B NMR (160 MHz, $CDCl_3$)

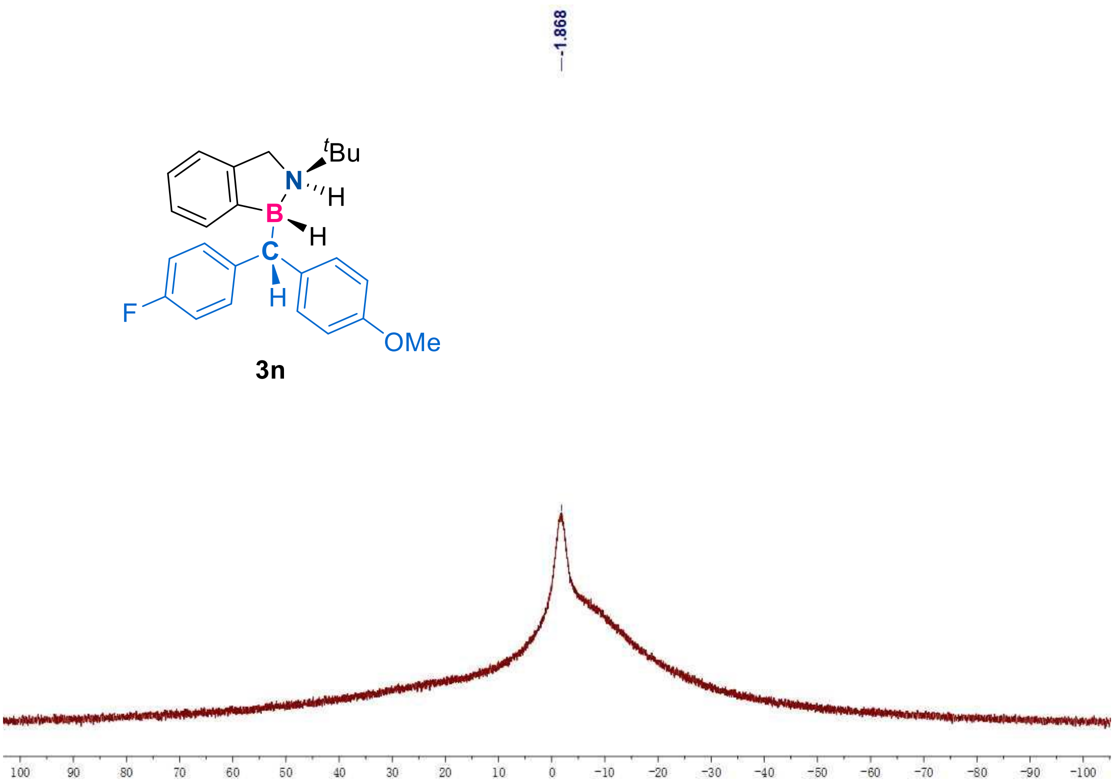

^{19}F NMR (471 MHz, $CDCl_3$)

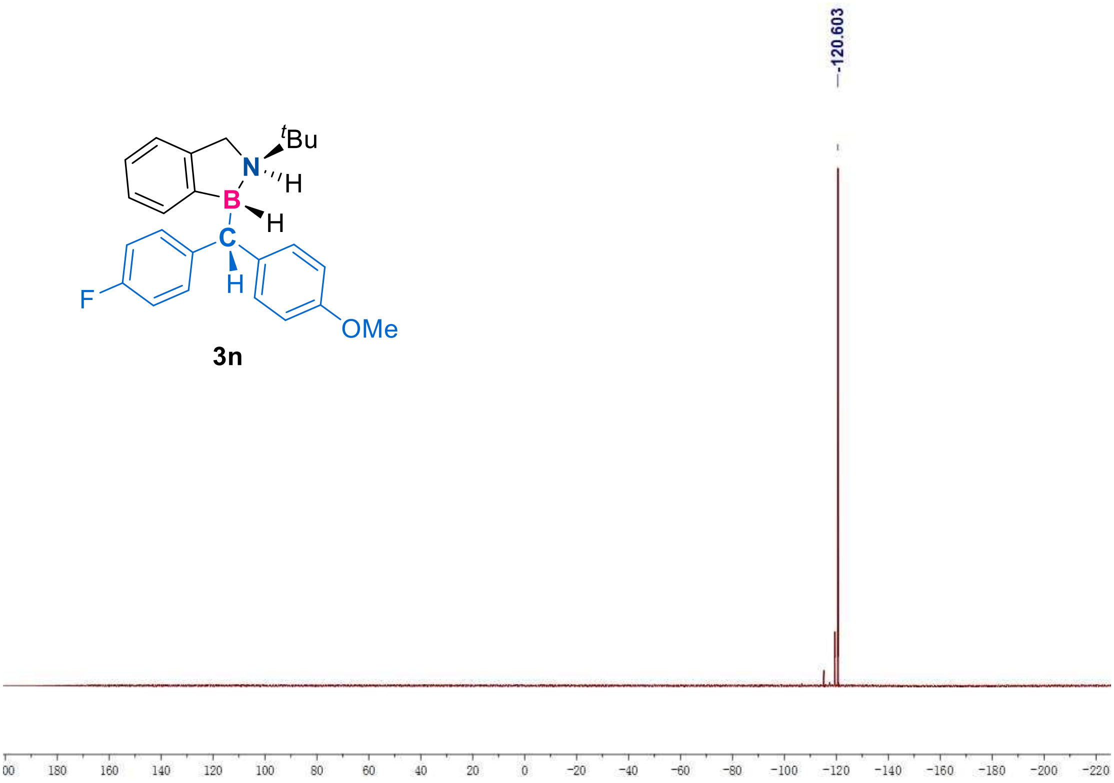

(R)-1-((4-bromophenyl)(4-methoxyphenyl)methyl)-2-(tert-butyl)-2,3-dihydro-1*H*-benzo[c][1,2]azaborole (3o)

^{1}H NMR (500 MHz, $CDCl_3$)

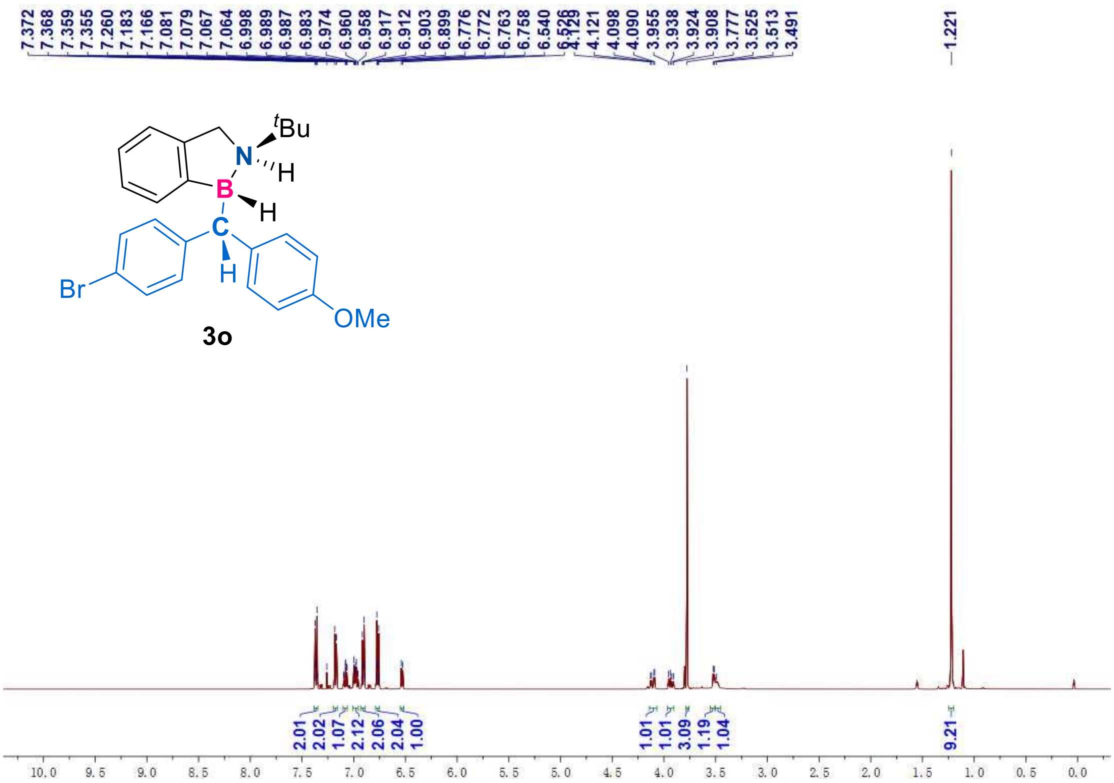

^{13}C NMR (126 MHz, $CDCl_3$)

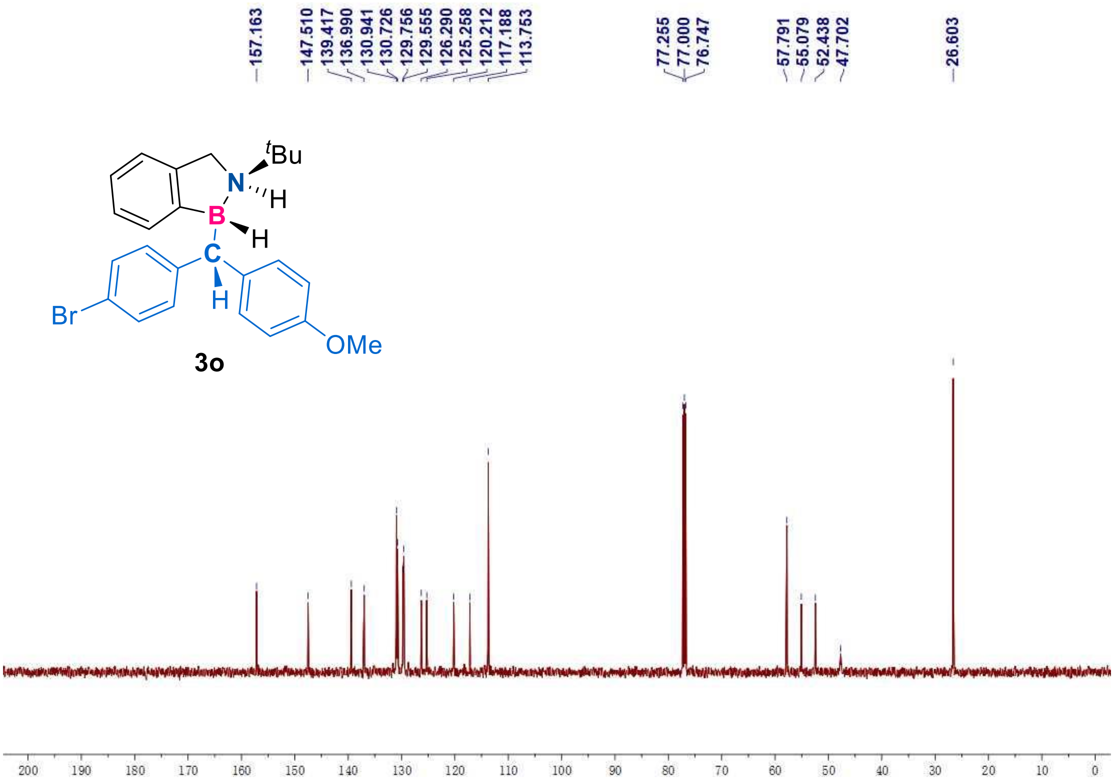

^{11}B NMR (160 MHz, $CDCl_3$)

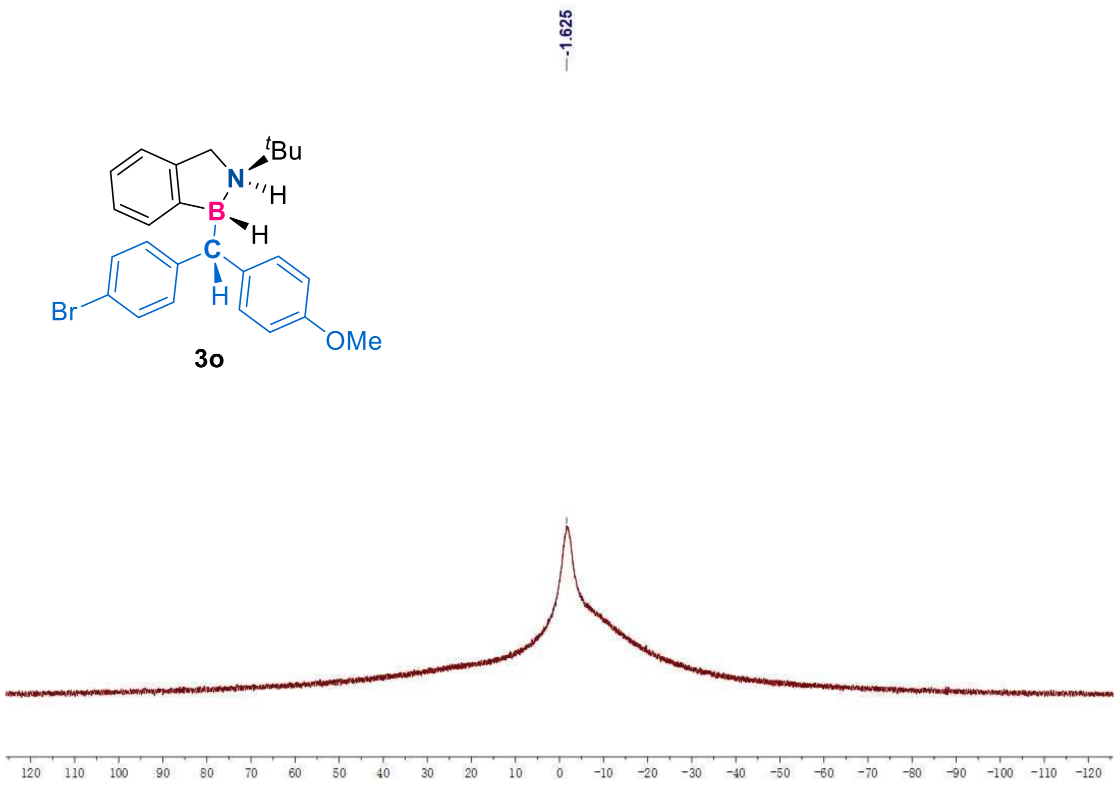

(S)-4-((2-(tert-butyl)-2,3-dihydro-1*H*-benzo[c][1,2]azaborol-1-yl)(4-methoxyphenyl)methyl)benzonitrile (**3p**)

^{1}H NMR (500 MHz, $CDCl_3$)

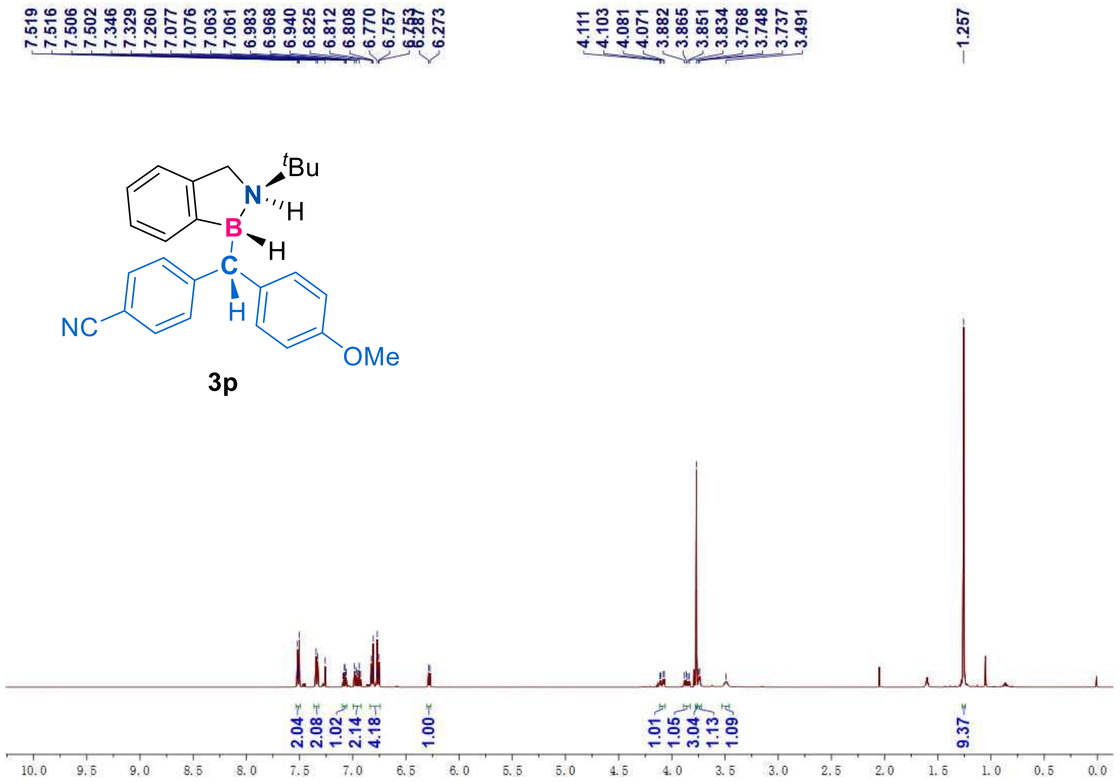

^{13}C NMR (126 MHz, $CDCl_3$)

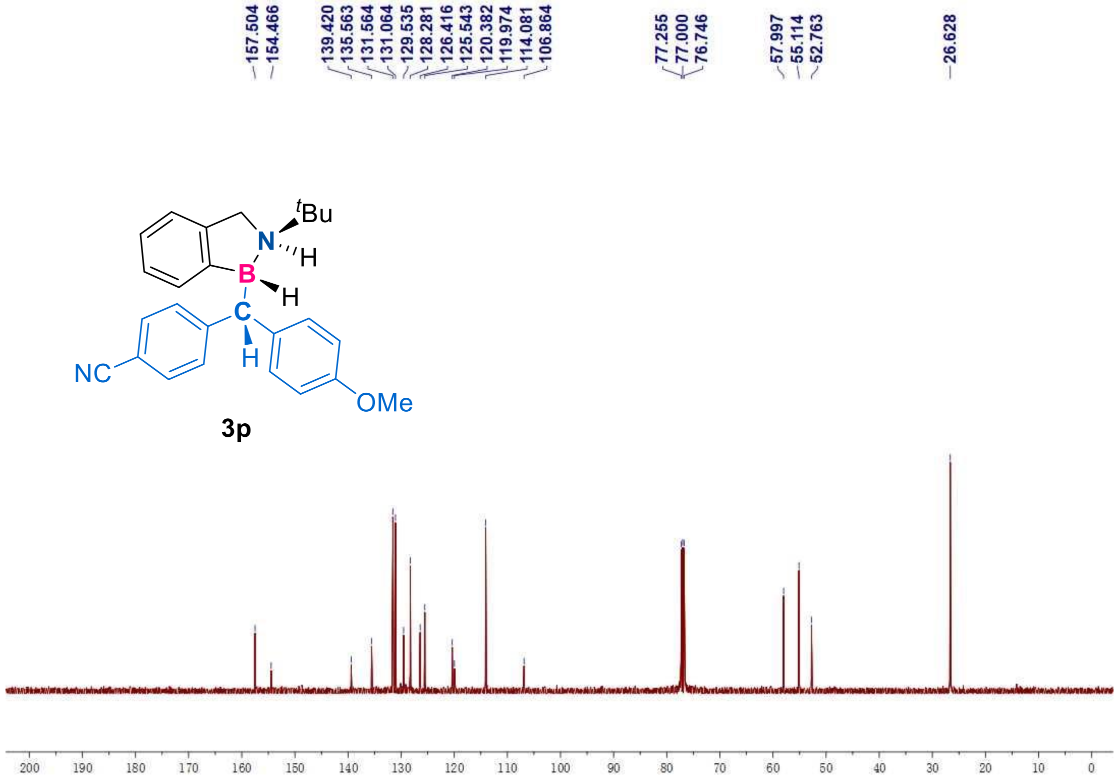

^{11}B NMR (160 MHz, $CDCl_3$)

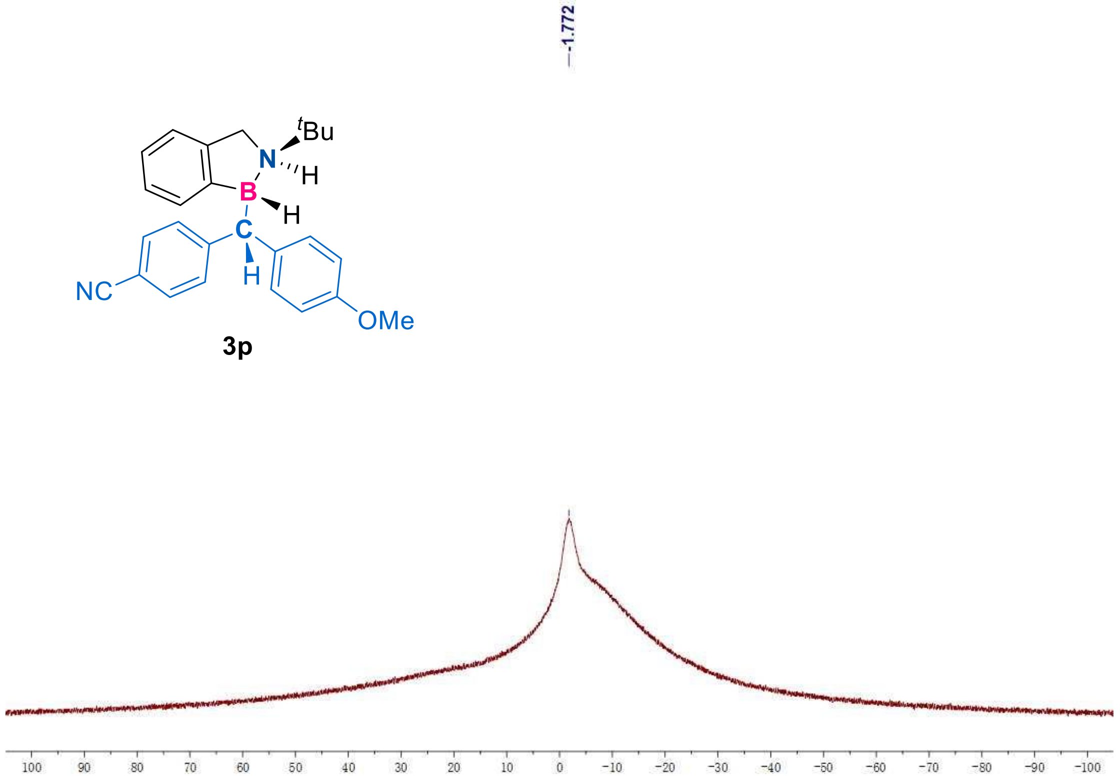

(R)-2-(tert-butyl)-1-((4-methoxyphenyl)(4-(methylsulfonyl)phenyl)methyl)-2,3-dihydro-1H-benzo[c][1,2]azaborole (**3q**)

^{1}H NMR (400 MHz, $CDCl_3$)

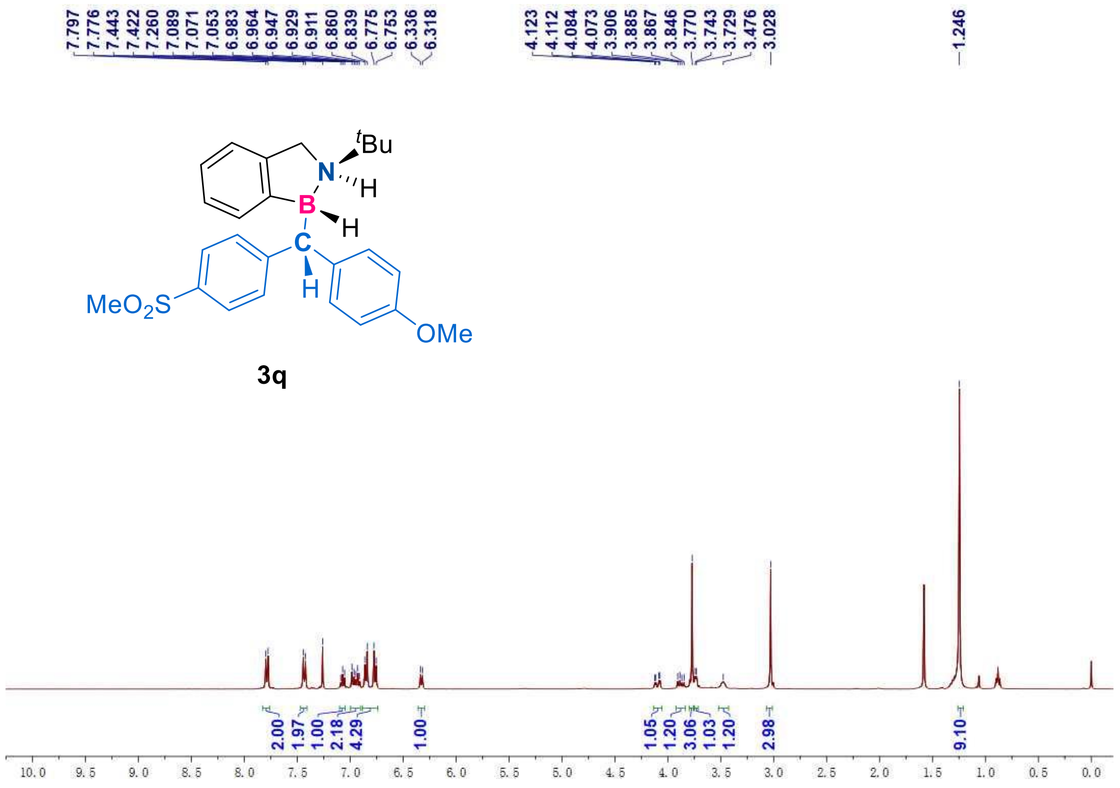

^{13}C NMR (101 MHz, $CDCl_3$)

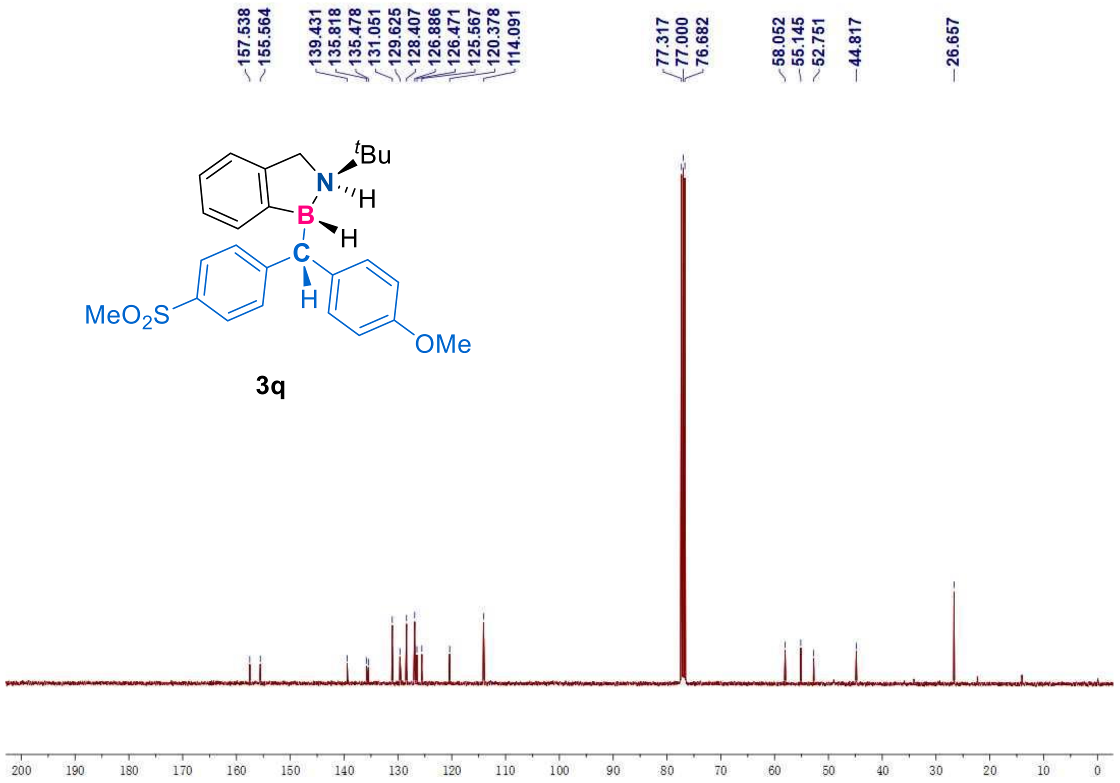

^{11}B NMR (160 MHz, $CDCl_3$)

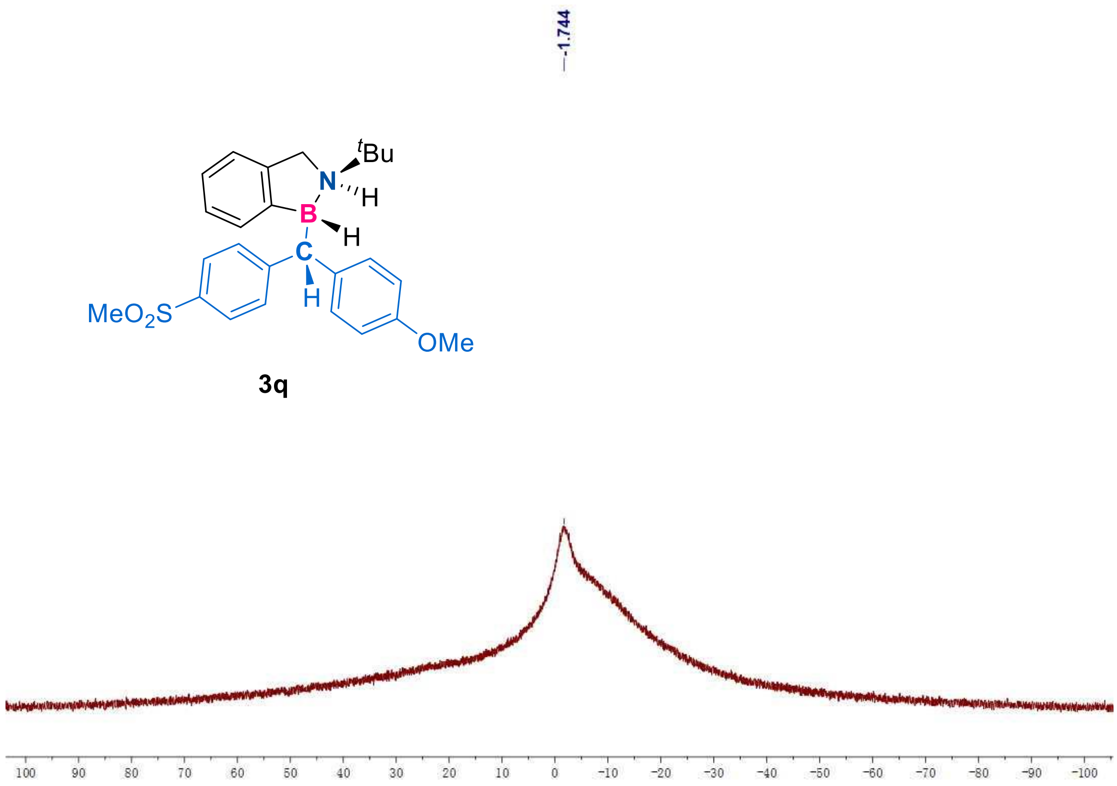

ethyl (S)-4-((2-(tert-butyl)-2,3-dihydro-1H-benzo[c][1,2]azaborol-1-yl)(4-methoxyphenyl)methyl)benzoate (**3r**)

^{1}H NMR (400 MHz, $CDCl_3$)

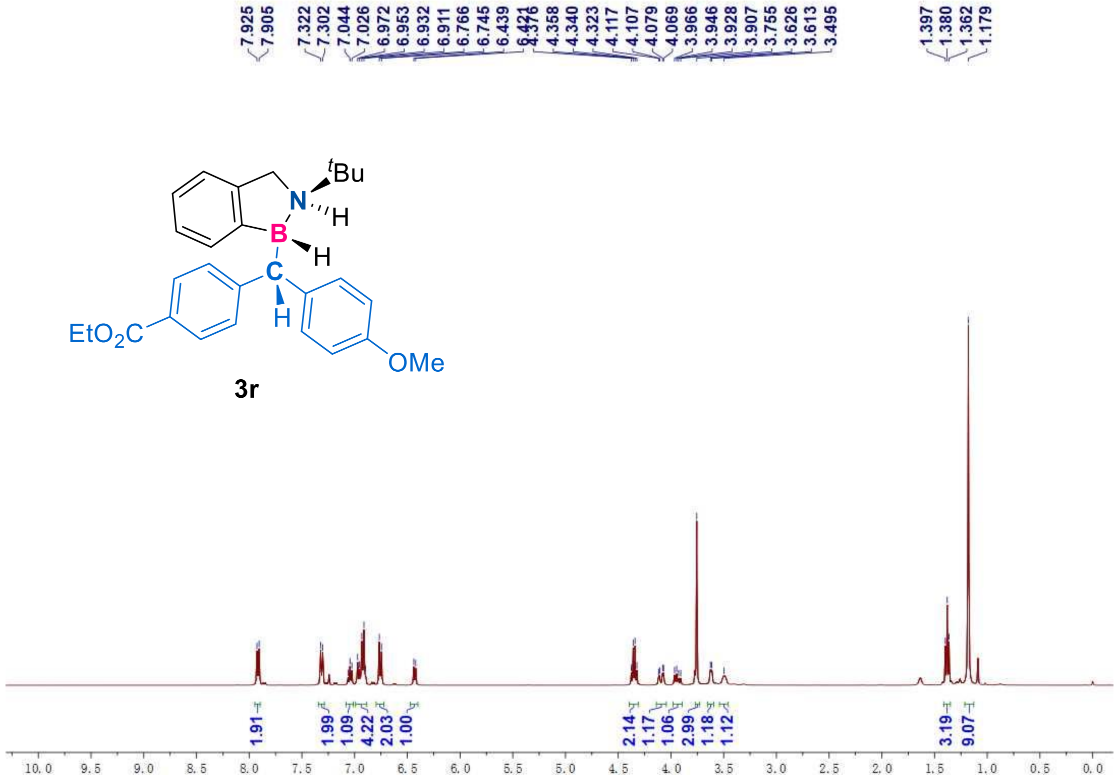

^{13}C NMR (101 MHz, $CDCl_3$)

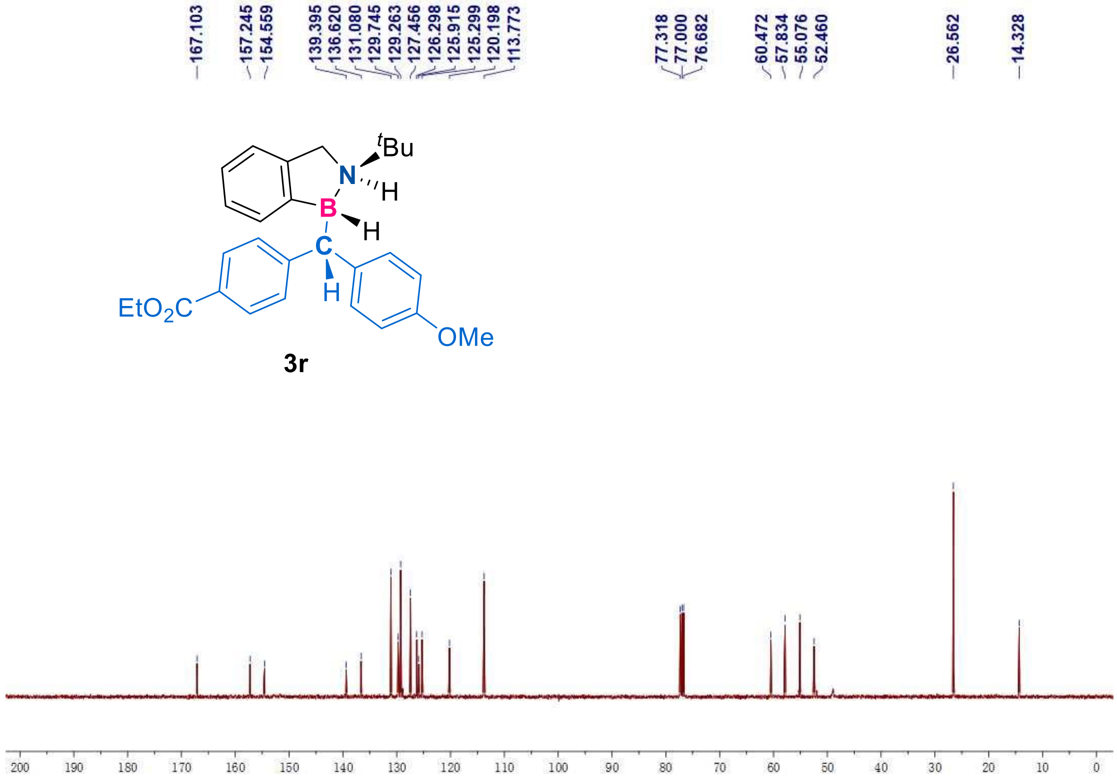

^{11}B NMR (128 MHz, $CDCl_3$)

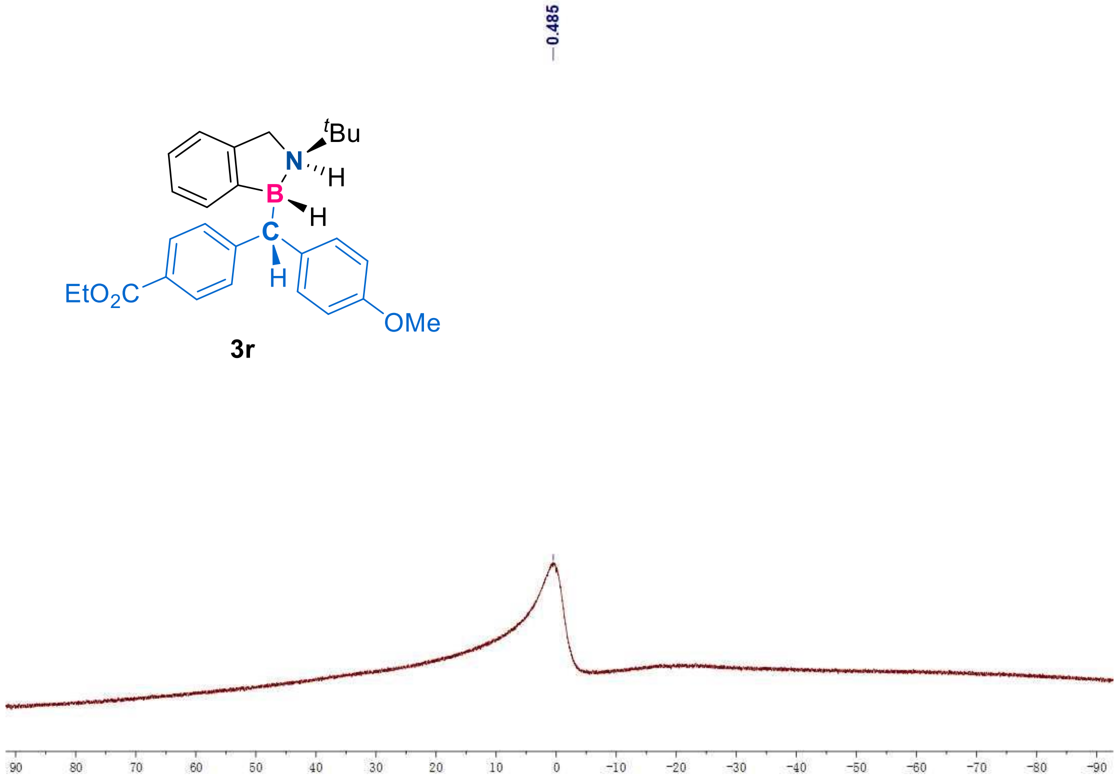

(S)-4-((2-(tert-butyl)-2,3-dihydro-1H-benzo[c][1,2]azaborol-1-yl)(4-methoxyphenyl)methyl)-N,N-dimethylbenzamide (3s)

^{1}H NMR (400 MHz, $CDCl_3$)

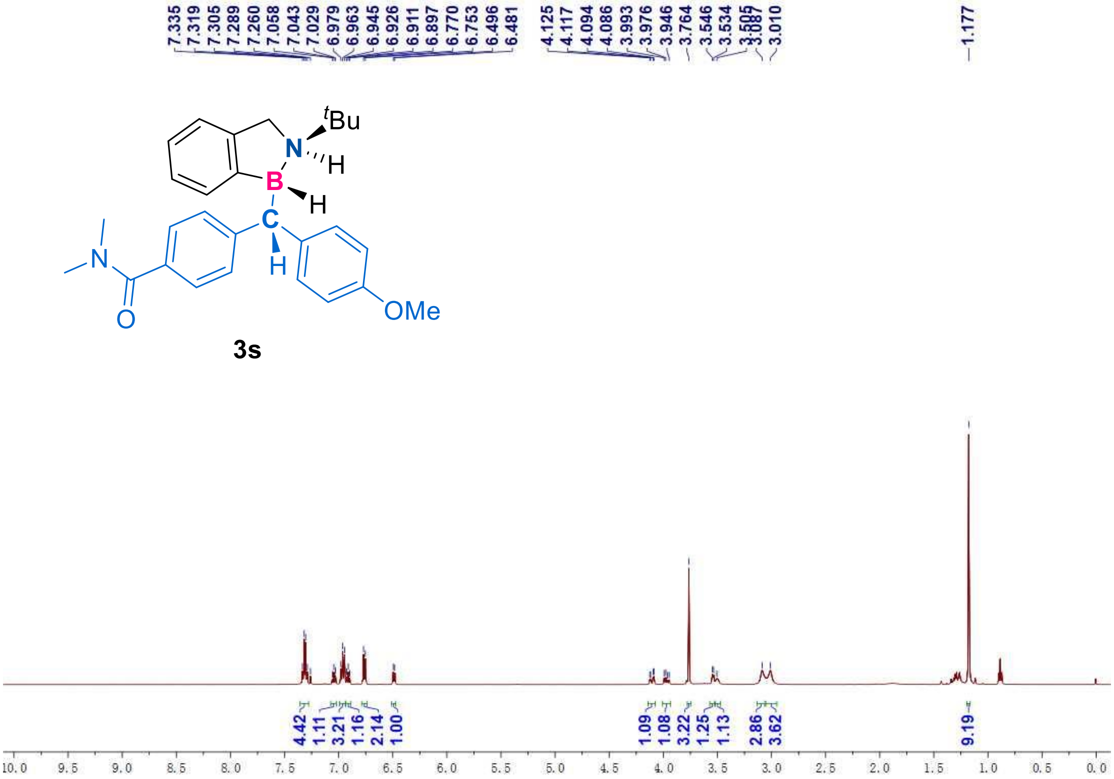

^{13}C NMR (126 MHz, $CDCl_3$)

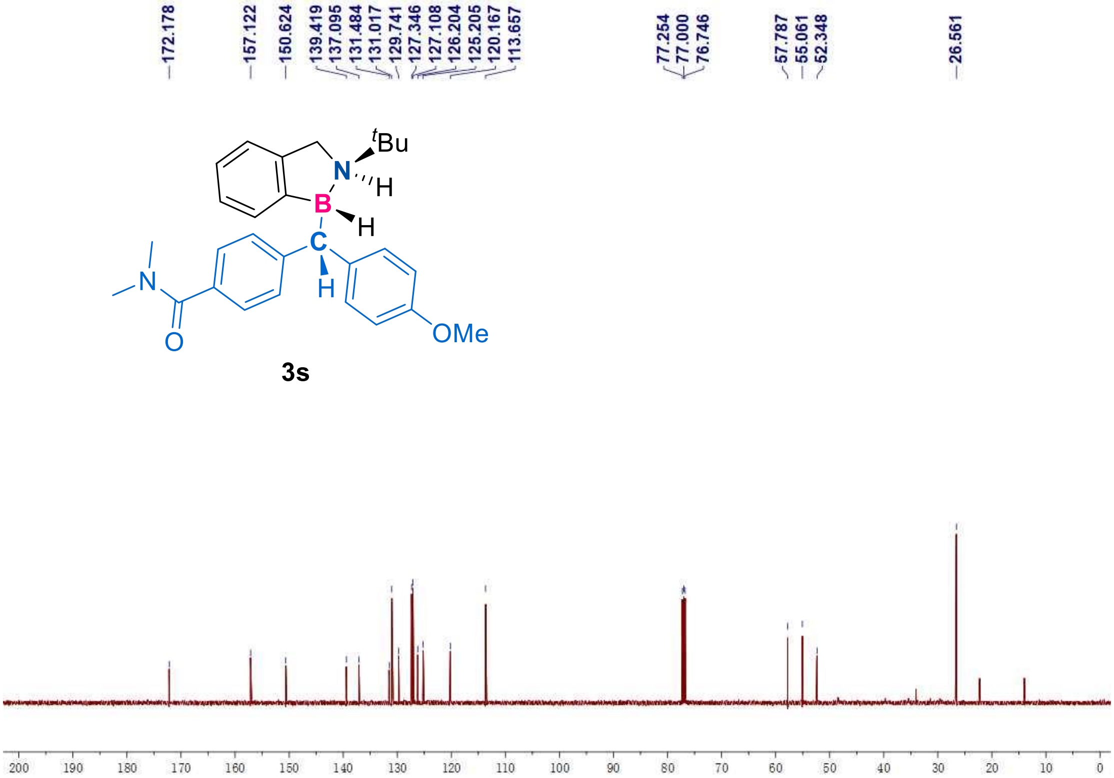

^{11}B NMR (128 MHz, $CDCl_3$)

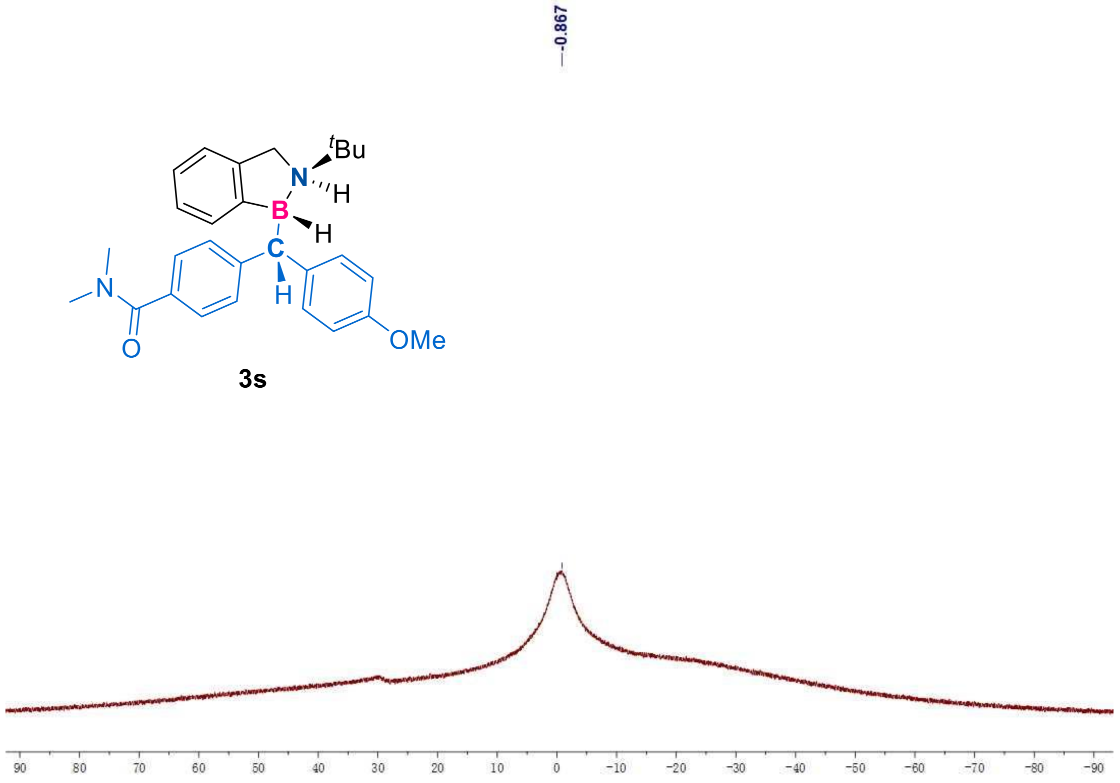

(R)-2-(tert-butyl)-1-((4-methoxyphenyl)(4-(trifluoromethoxy)phenyl)methyl)-2,3-dihydro-1H-benzo[c][1,2]azaborole (3t)

^{1}H NMR (500 MHz, $CDCl_3$)

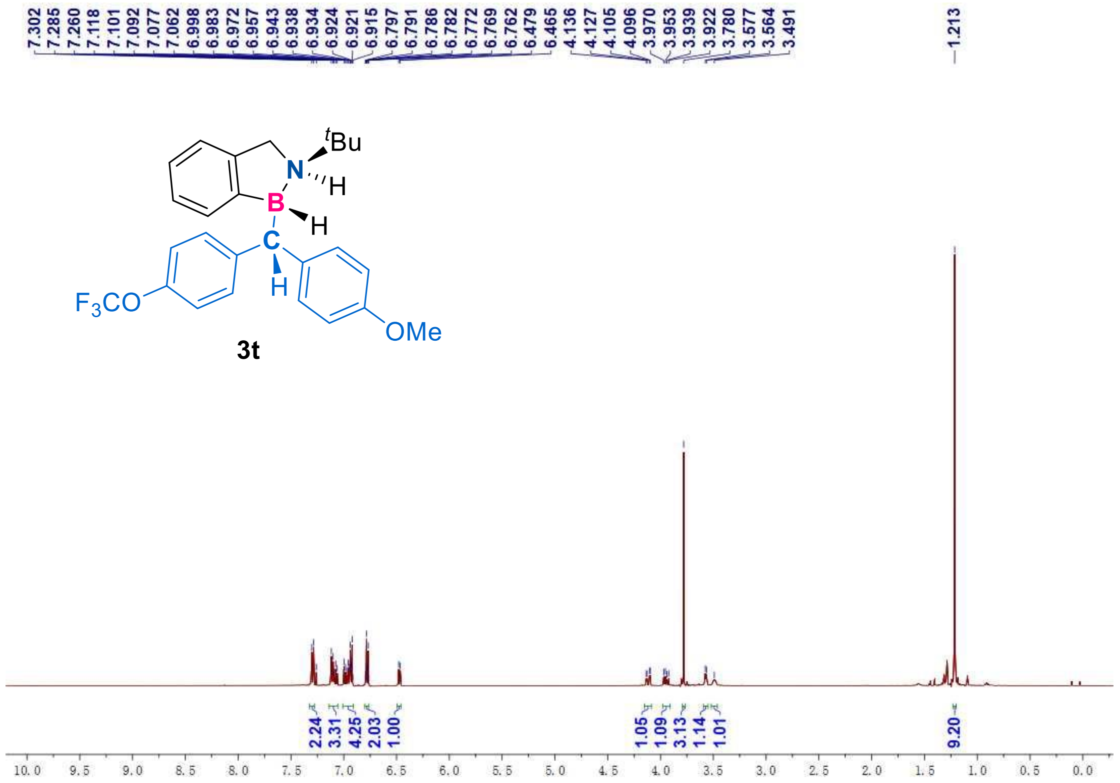

^{13}C NMR (126 MHz, $CDCl_3$)

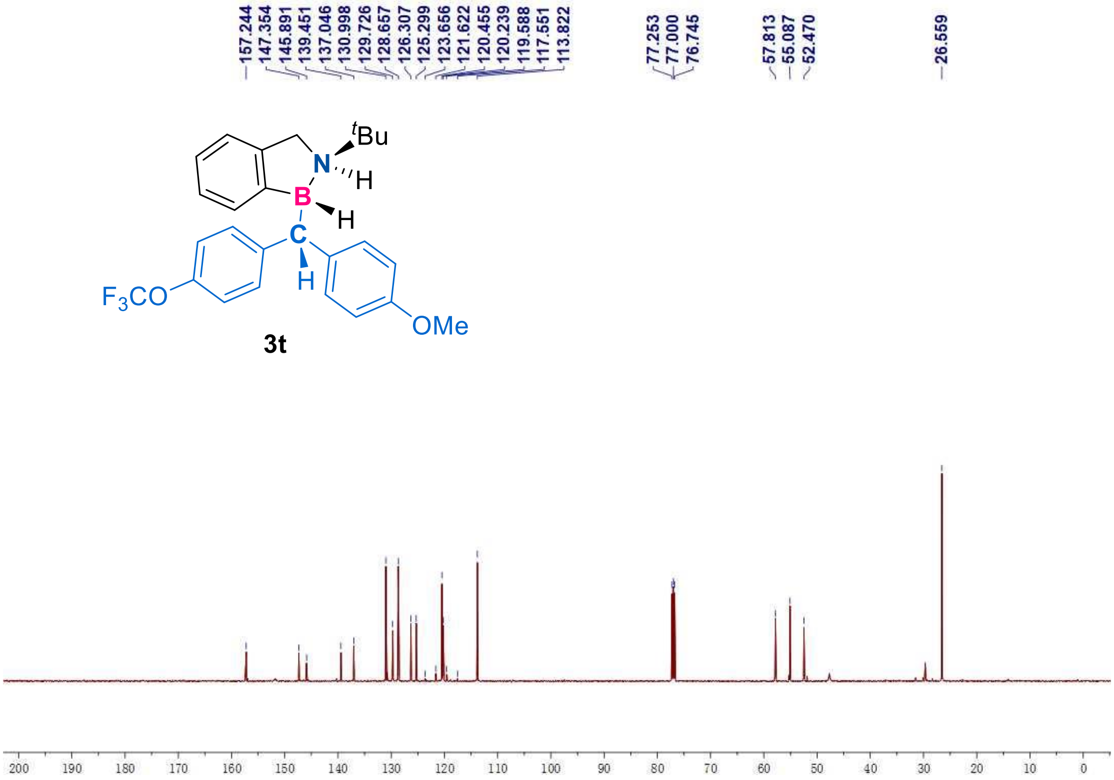

^{11}B NMR (160 MHz, $CDCl_3$)

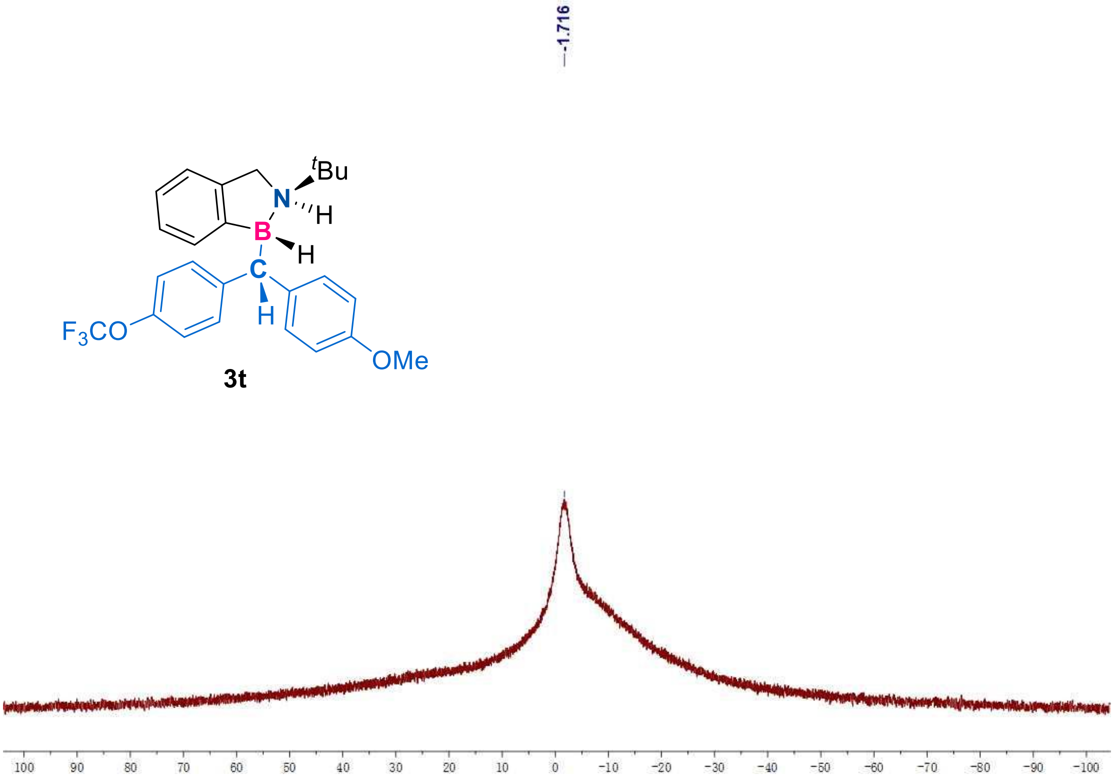

¹⁹F NMR (471 MHz, $CDCl_3$)

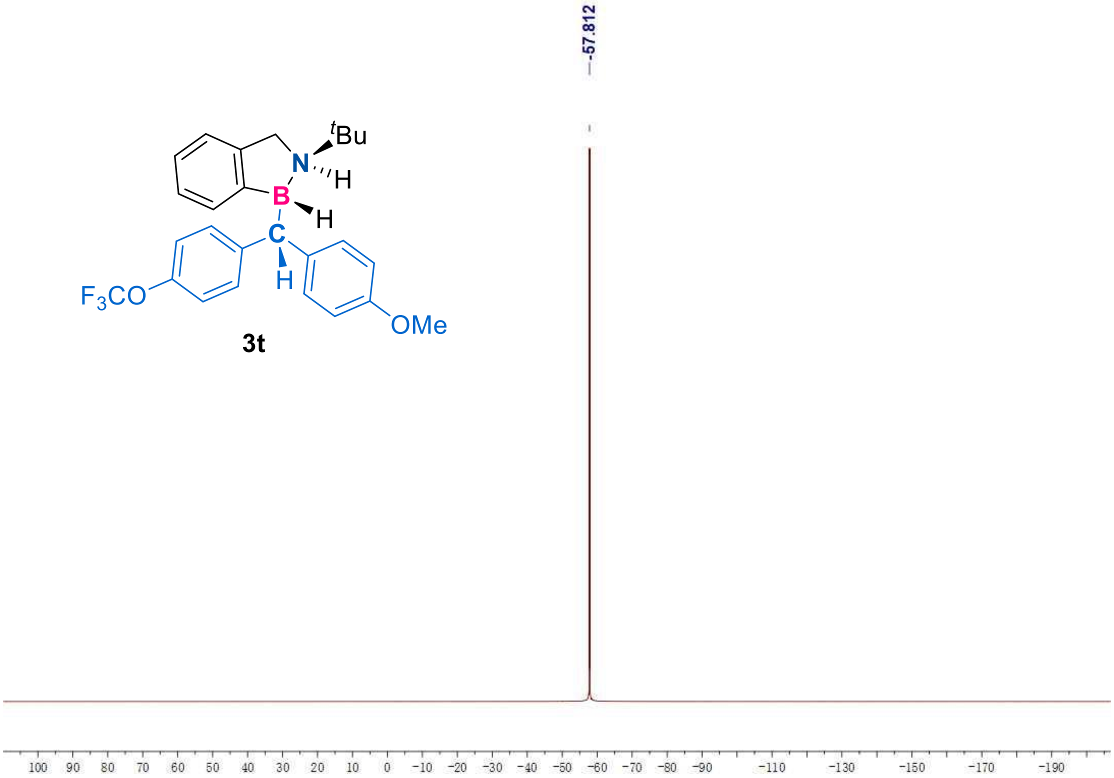

(S)-2-(tert-butyl)-1-((4-methoxyphenyl)(4-(trifluoromethyl)phenyl)methyl)-2,3-dihydro-1H-benzo[c][1,2]azaborole (**3u**)

¹H NMR (500 MHz, Acetone-d_6)

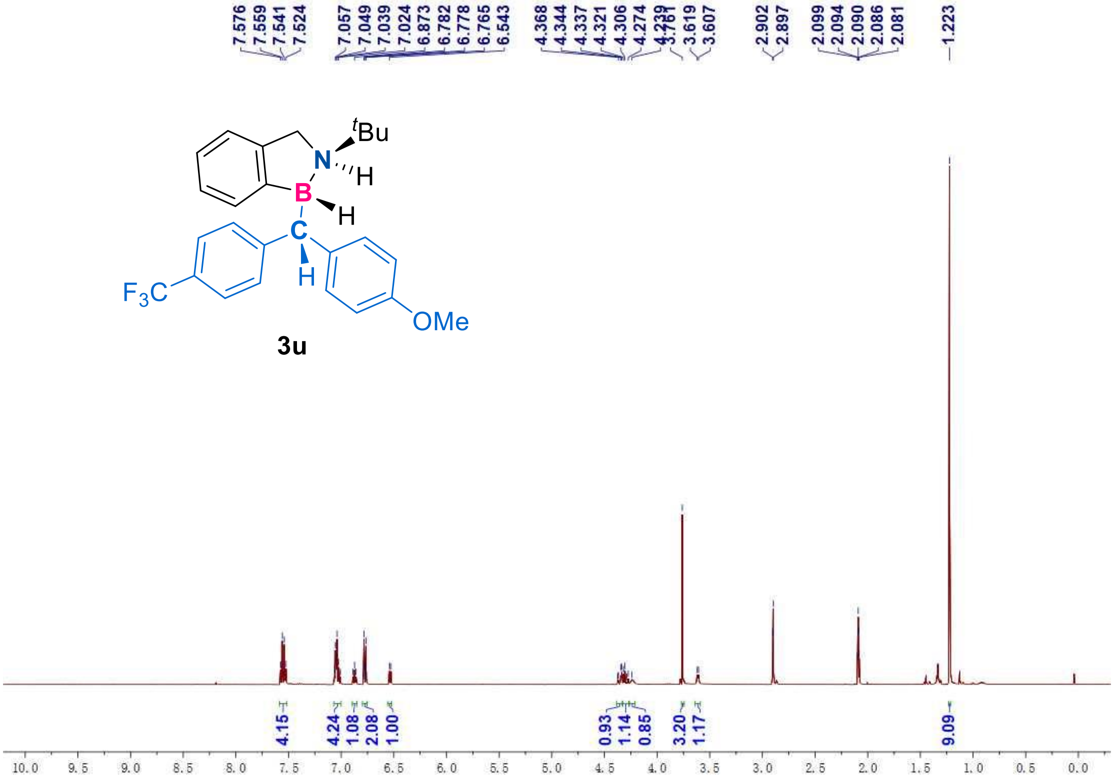

^{13}**C NMR** (126 MHz, Acetone-d_6)

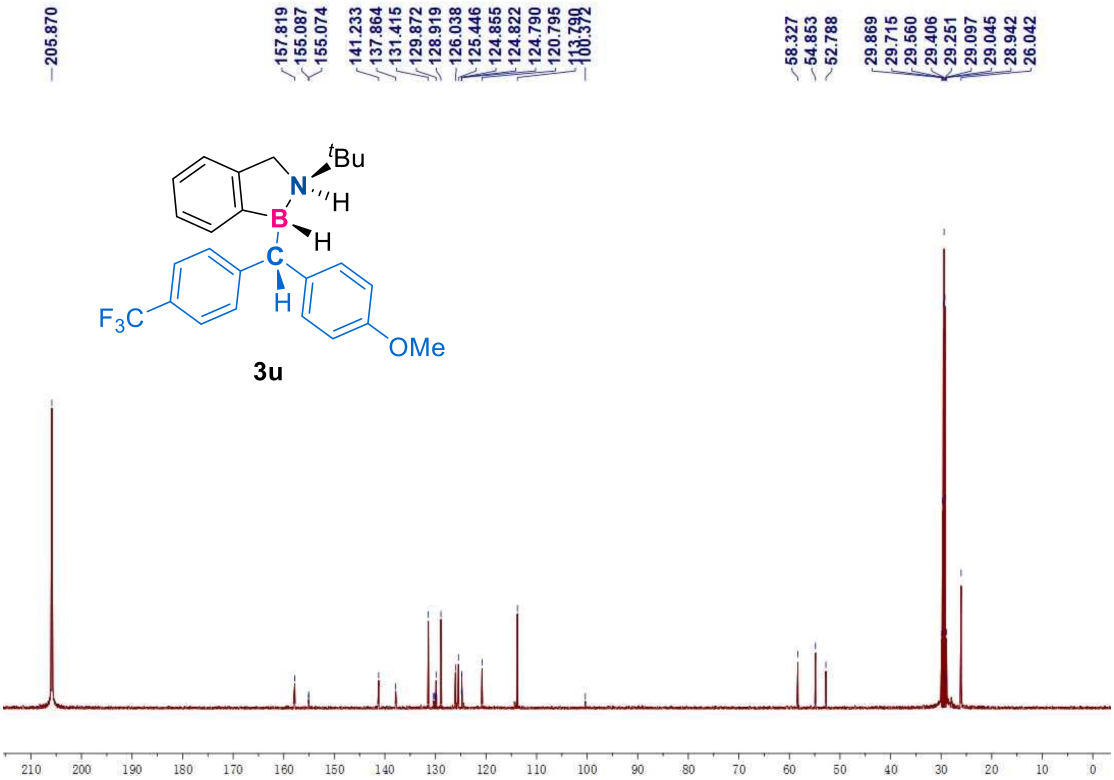

^{11}**B NMR** (160 MHz, Acetone-d_6)

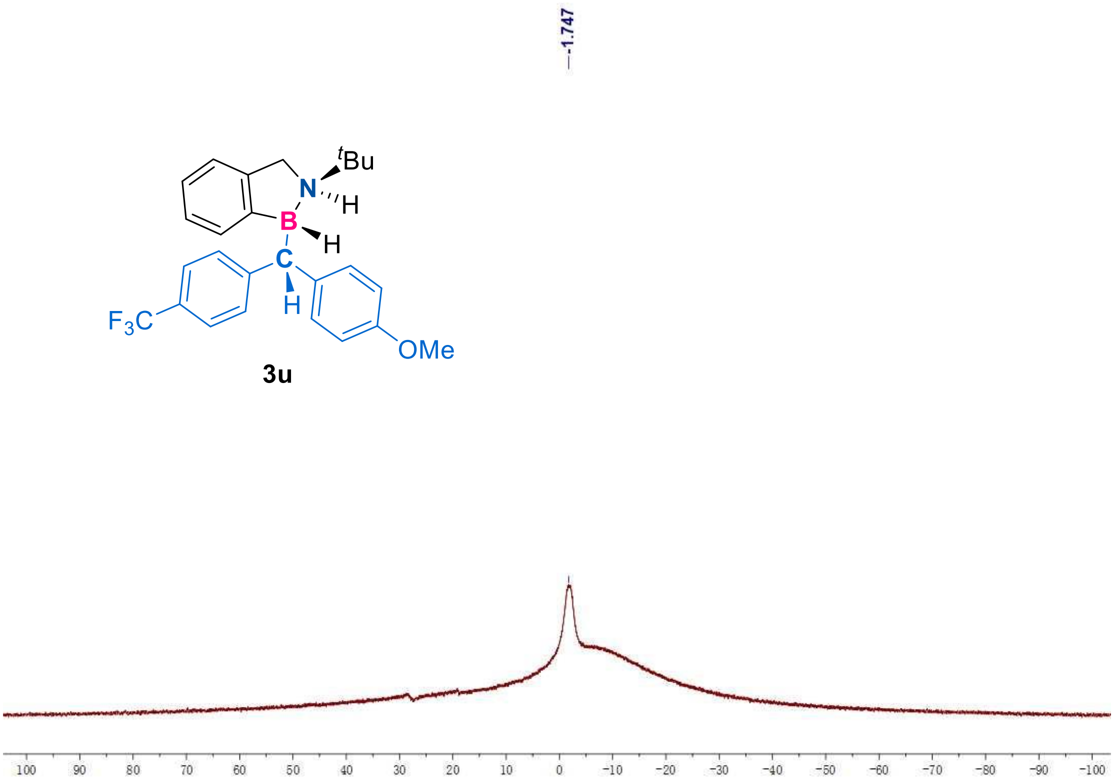

[19]**F NMR** (471 MHz, Acetone-d_6)

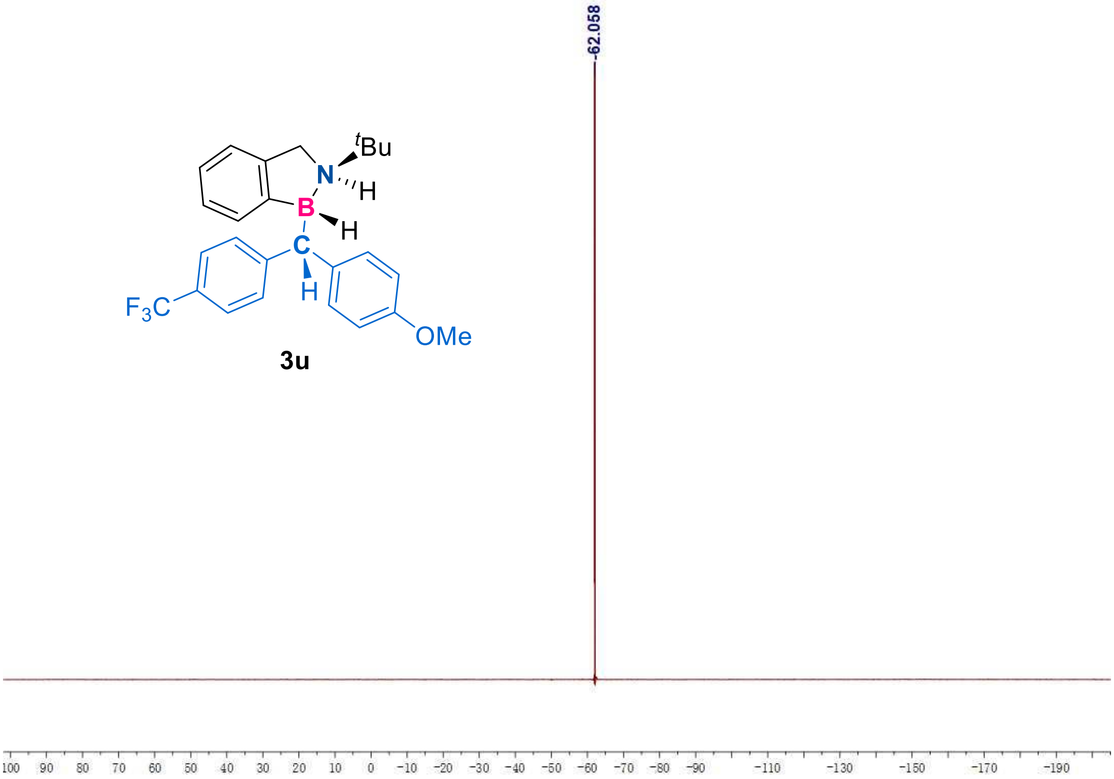

(S)-2-(tert-butyl)-1-((3-methoxyphenyl)(4-(trifluoromethyl)phenyl)methyl)-2,3-dihydro-1H-benzo[c][1,2]azaborole (3v)

[1]**H NMR** (500 MHz, Acetone-d_6)

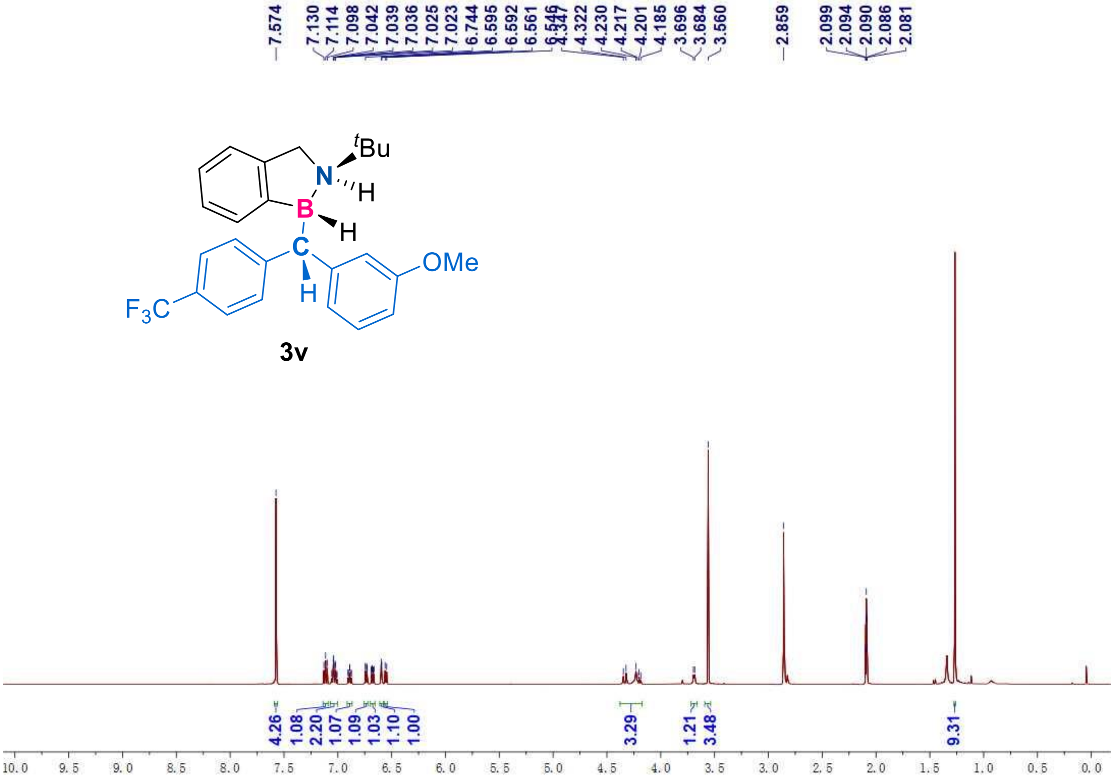

^{13}C NMR (126 MHz, Acetone-d_6)

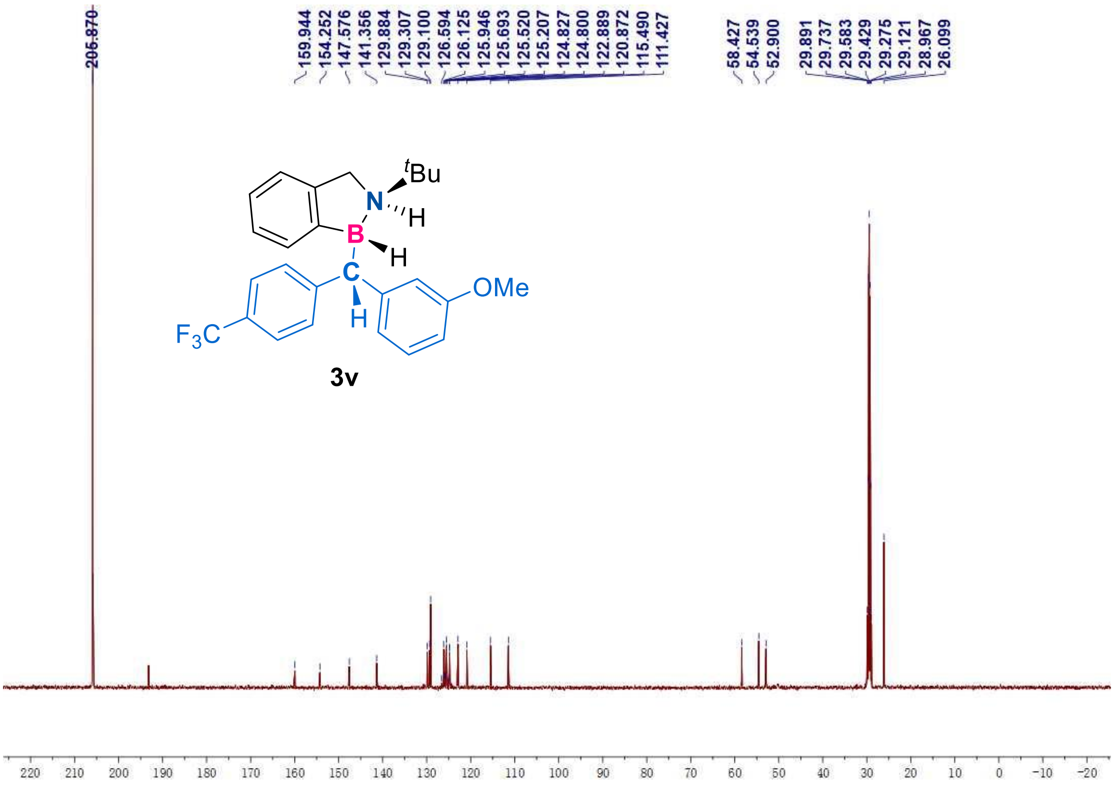

^{11}B NMR (160 MHz, Acetone-d_6)

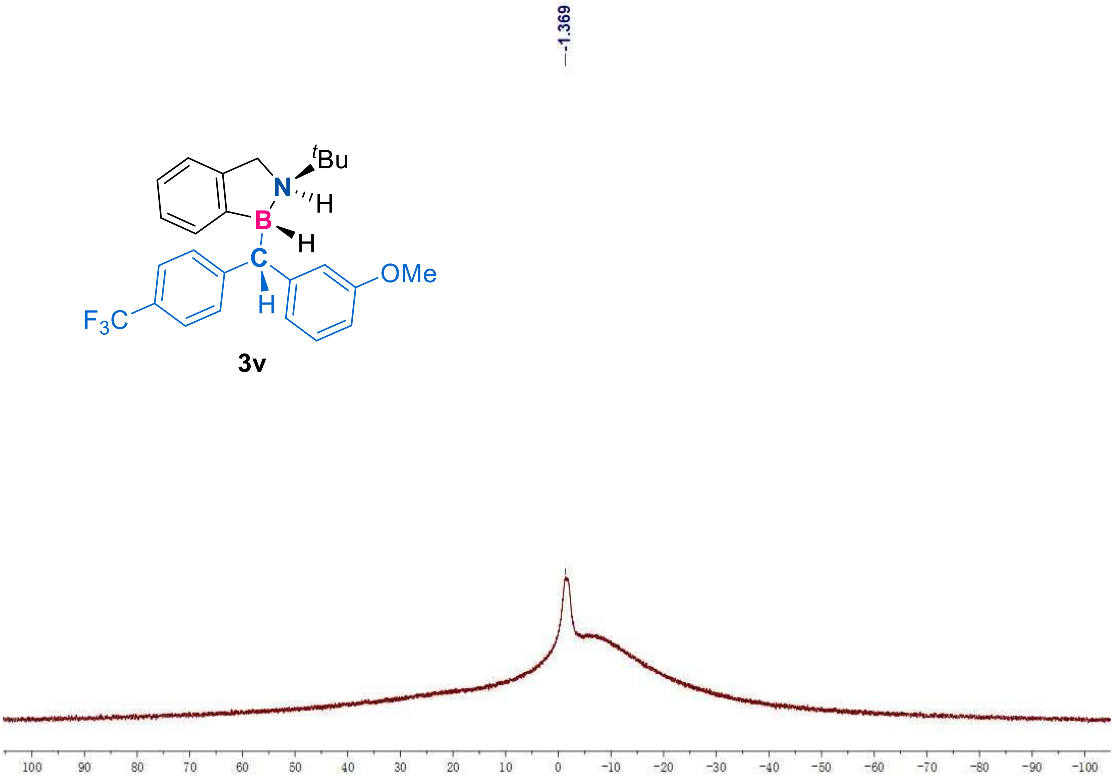

^{19}F NMR (471 MHz, Acetone-d_6)

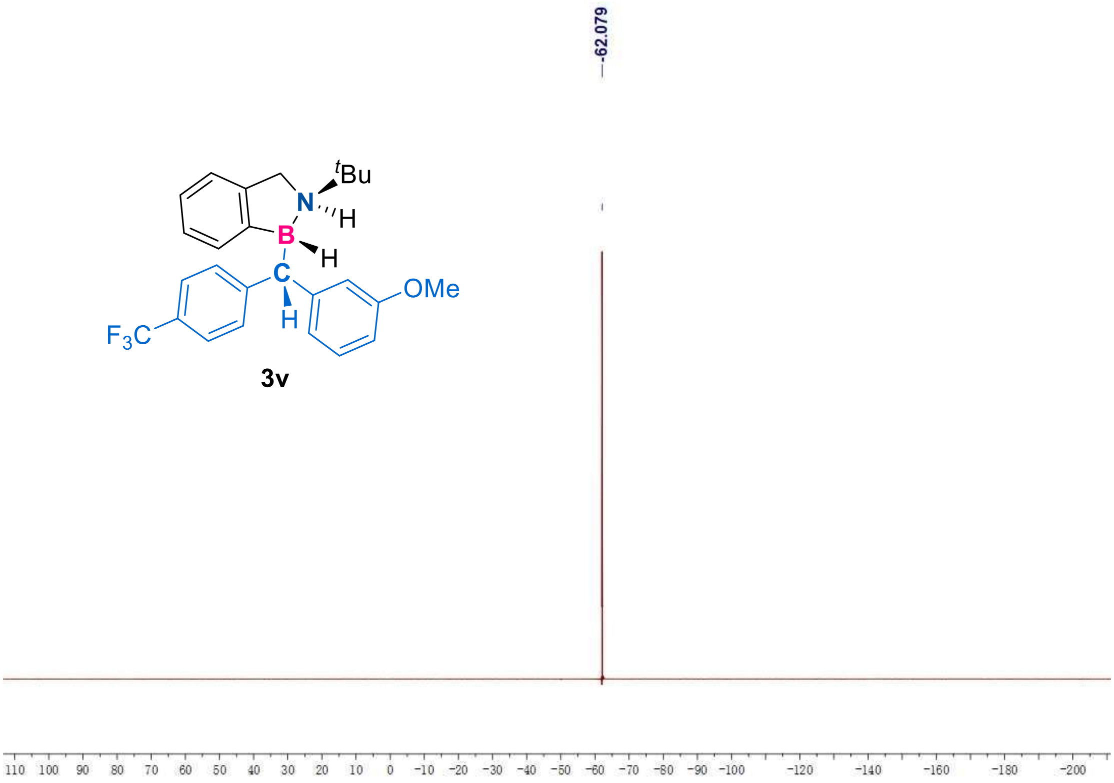

(S)-1-((4-(benzyloxy)phenyl)(4-(trifluoromethyl)phenyl)methyl)-2-(tert-butyl)-2,3-dihydro-1H-benzo[c][1,2]azaborole (3w)

^{1}H NMR (500 MHz, Acetone-d_6)

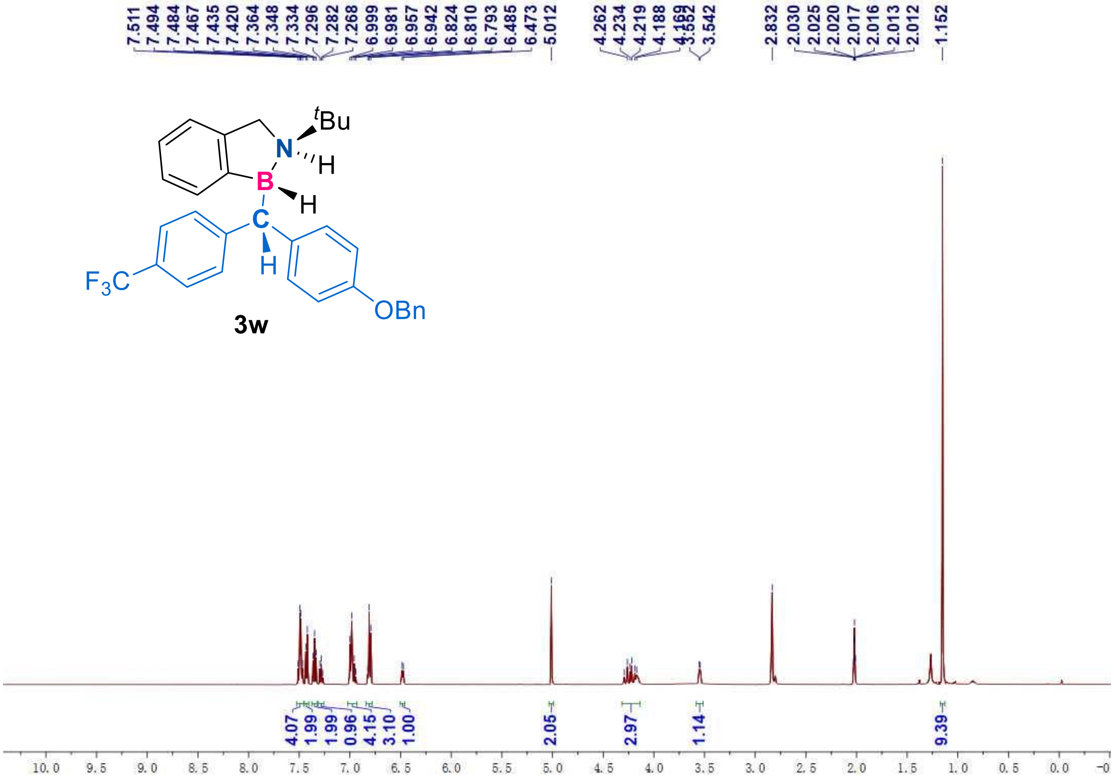

^{13}C NMR (126 MHz, Acetone-d_6)

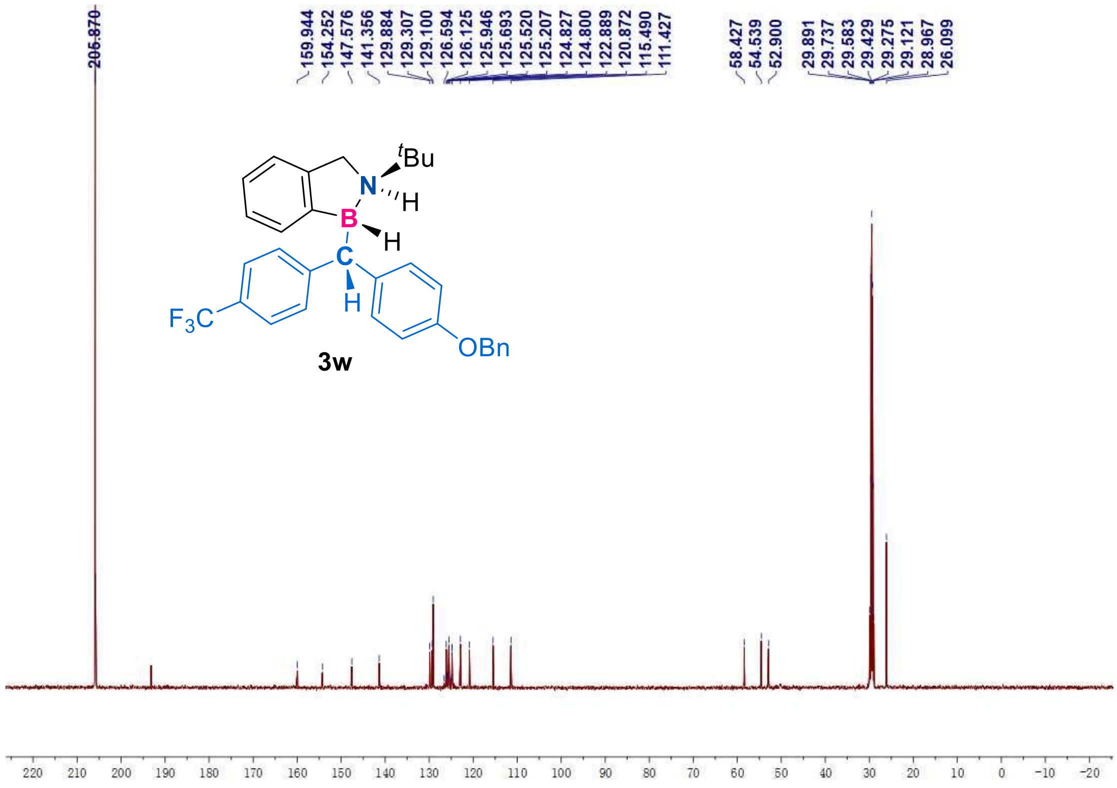

^{11}B NMR (160 MHz, Acetone-d_6)

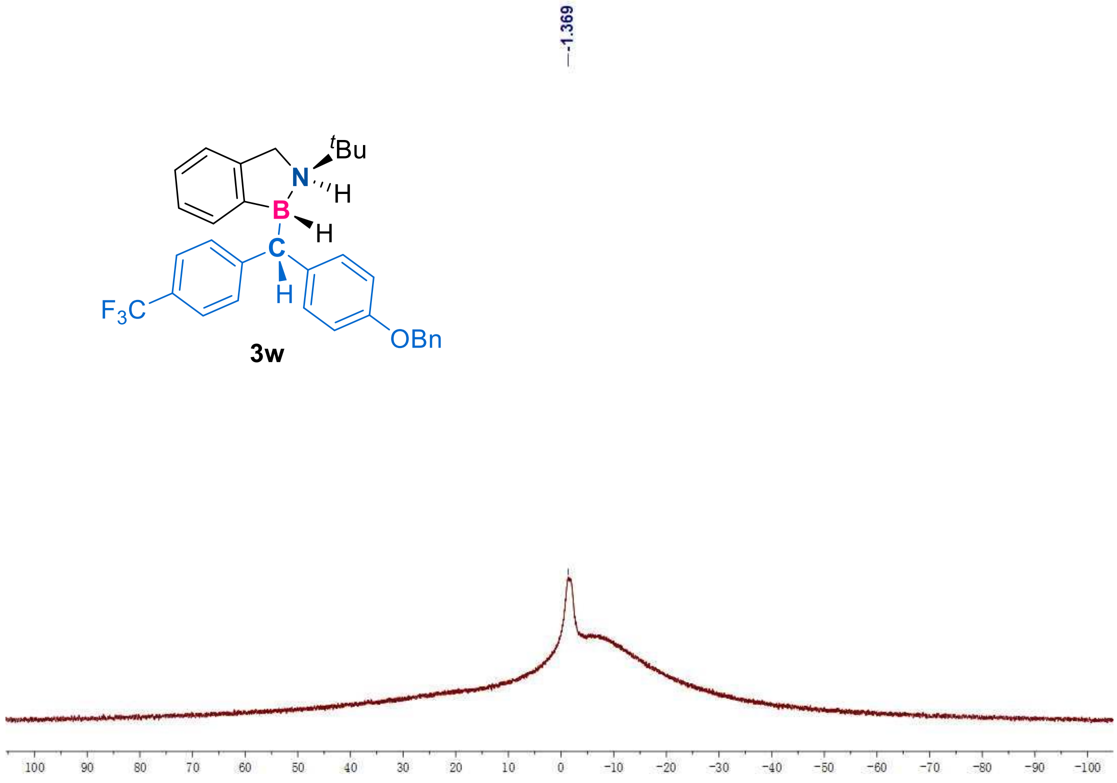

^{19}**F NMR** (471 MHz, Acetone-d_6)

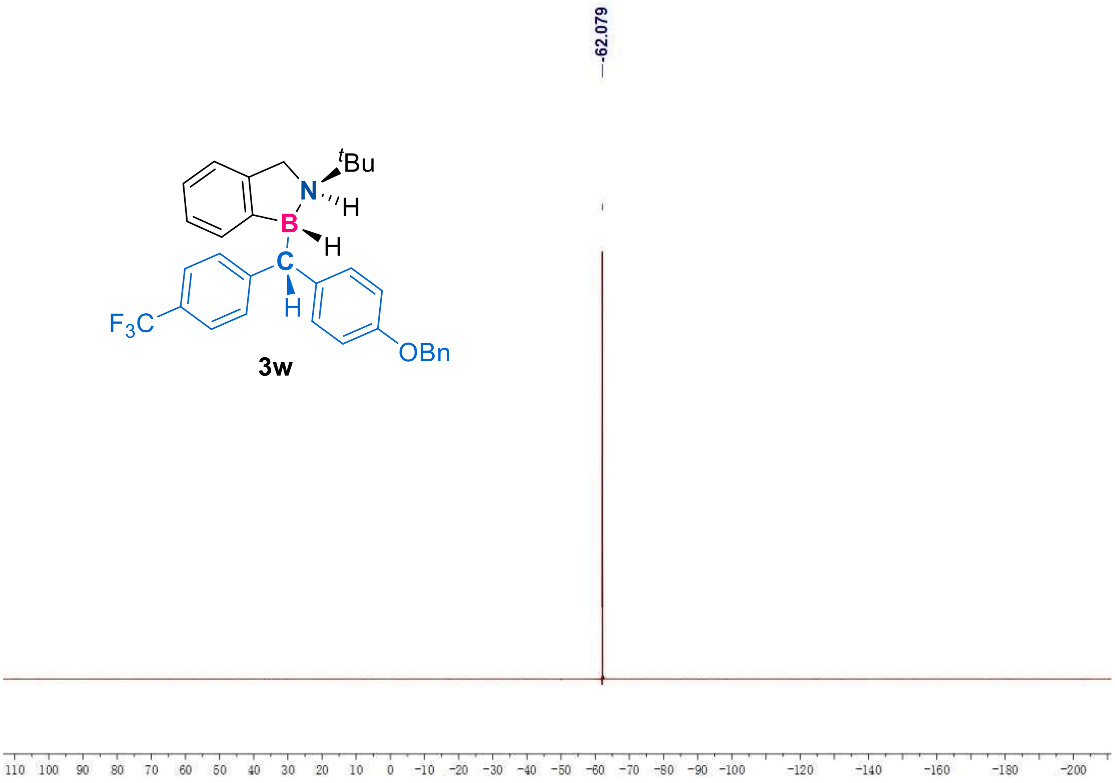

(S)-2-(tert-butyl)-1-((4-methoxyphenyl)(4-nitrophenyl)methyl)-2,3-dihydro-1H-benzo[c][1,2]azaborole (**3x**)

^{1}**H NMR** (500 MHz, Acetone-d_6)

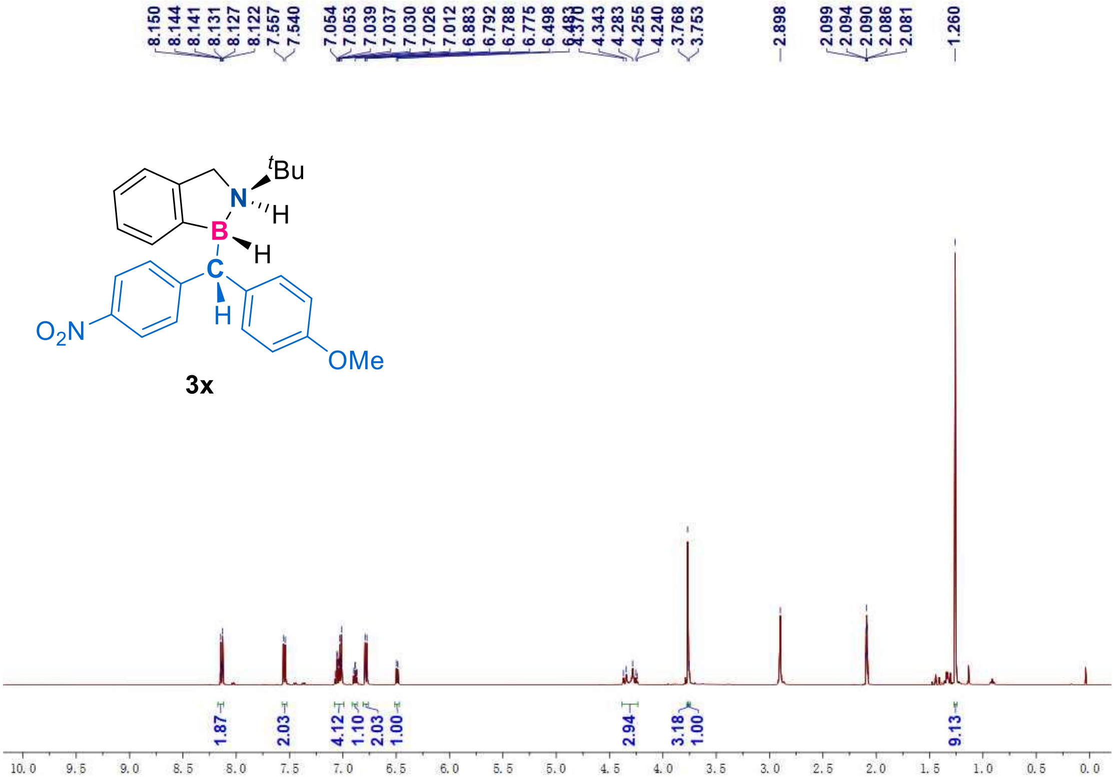

^{13}C NMR (126 MHz, Acetone-d_6)

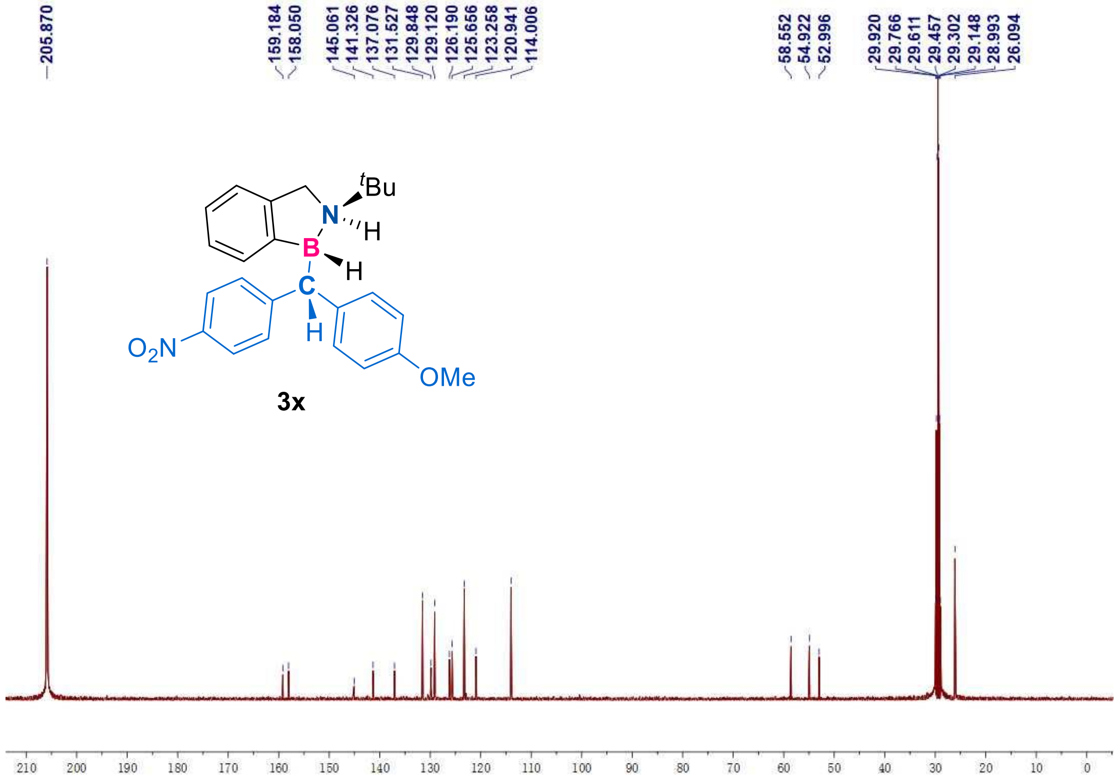

^{11}B NMR (160 MHz, Acetone-d_6)

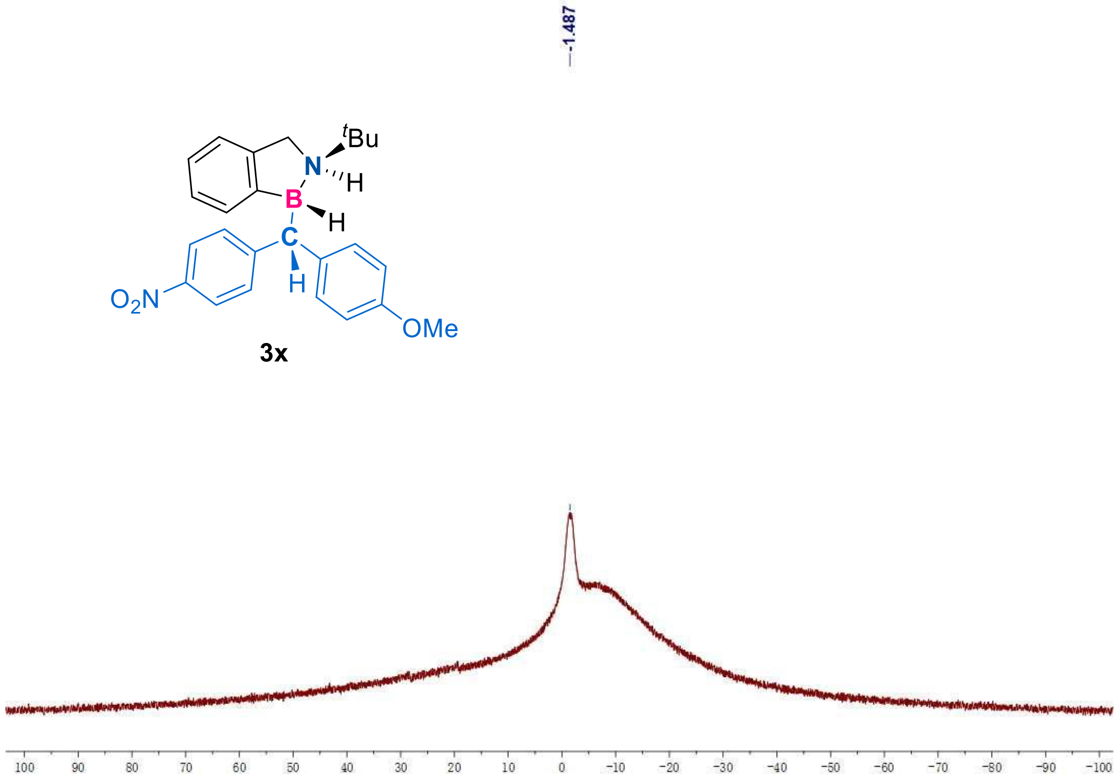

(R)-2-(tert-butyl)-1-((4-methoxyphenyl)(3-nitrophenyl)methyl)-2,3-dihydro-1H-benzo[c][1,2]azaborole (**3y**)

^{1}H NMR (500 MHz, Acetone-d_6)

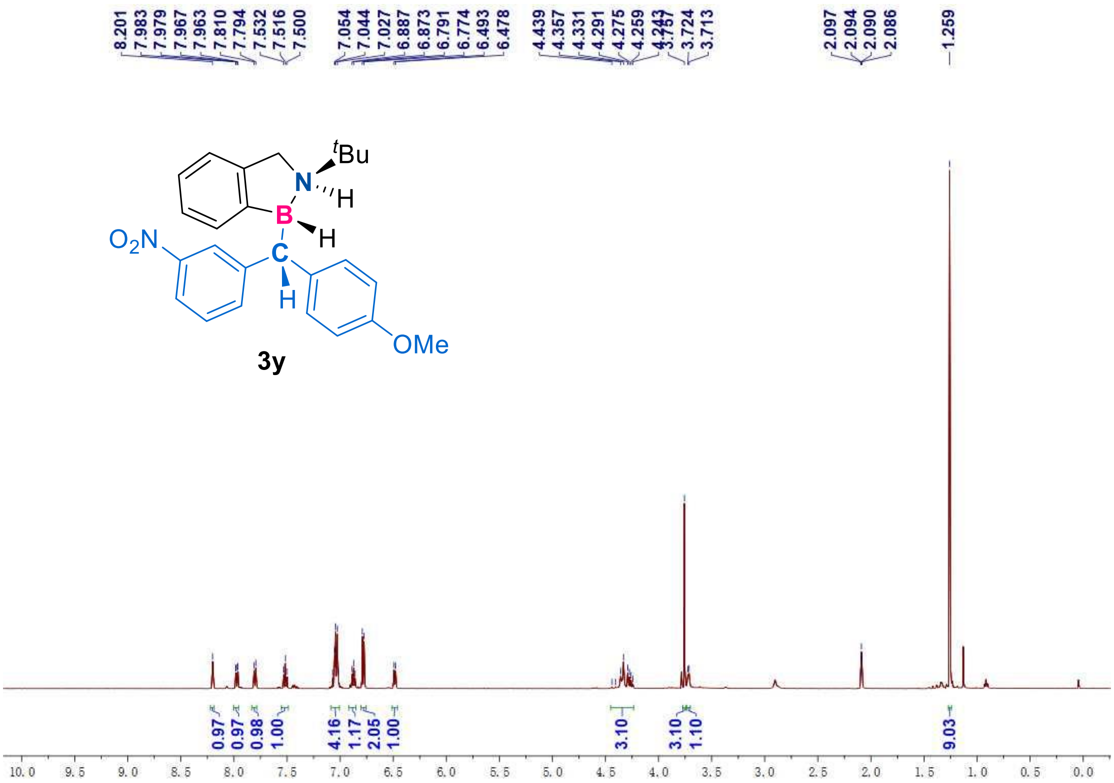

^{13}C NMR (126 MHz, Acetone-d_6)

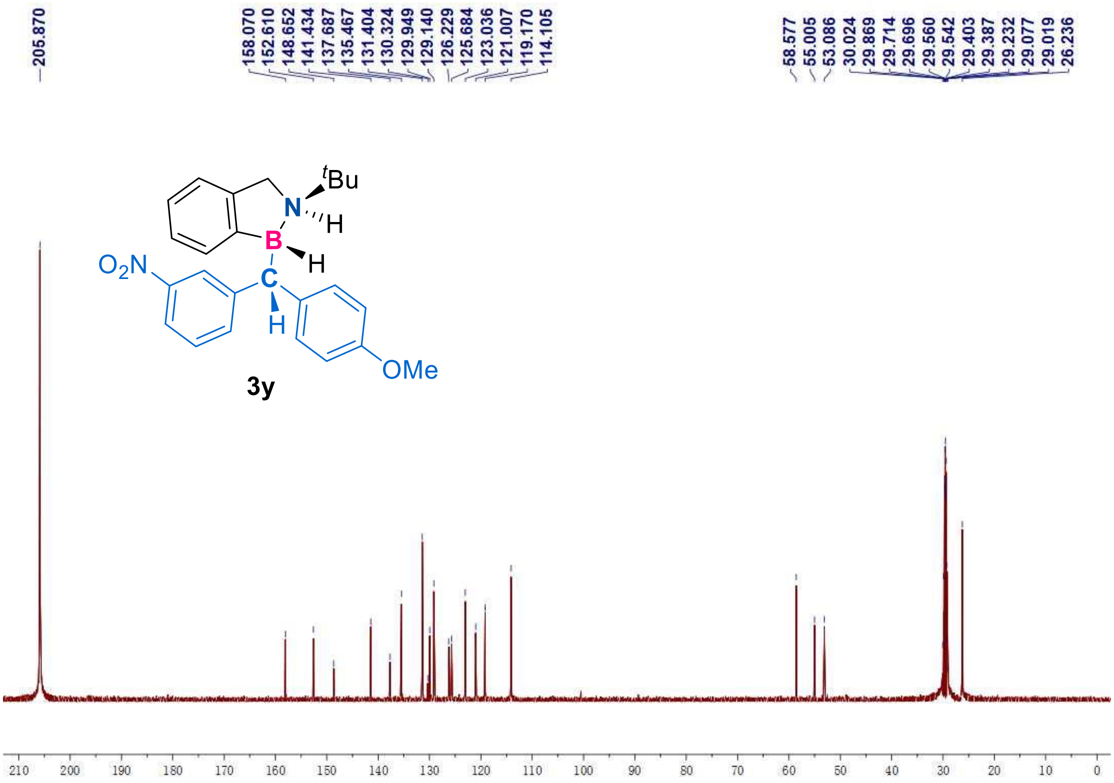

^{11}B NMR (160 MHz, Acetone-d_6)

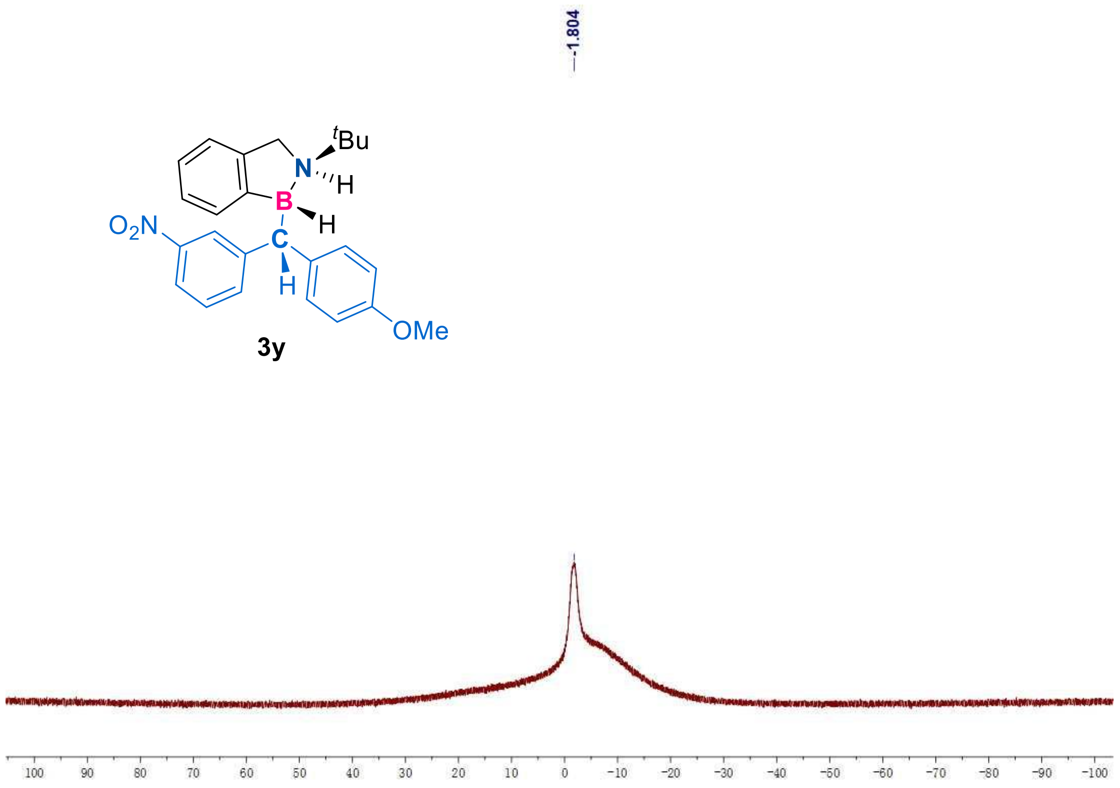

(R)-2-(tert-butyl)-1-((4-nitrophenyl)(p-tolyl)methyl)-2,3-dihydro-1H-benzo[c][1,2]azaborole (3z)

^{1}H NMR (500 MHz, $CDCl_3$)

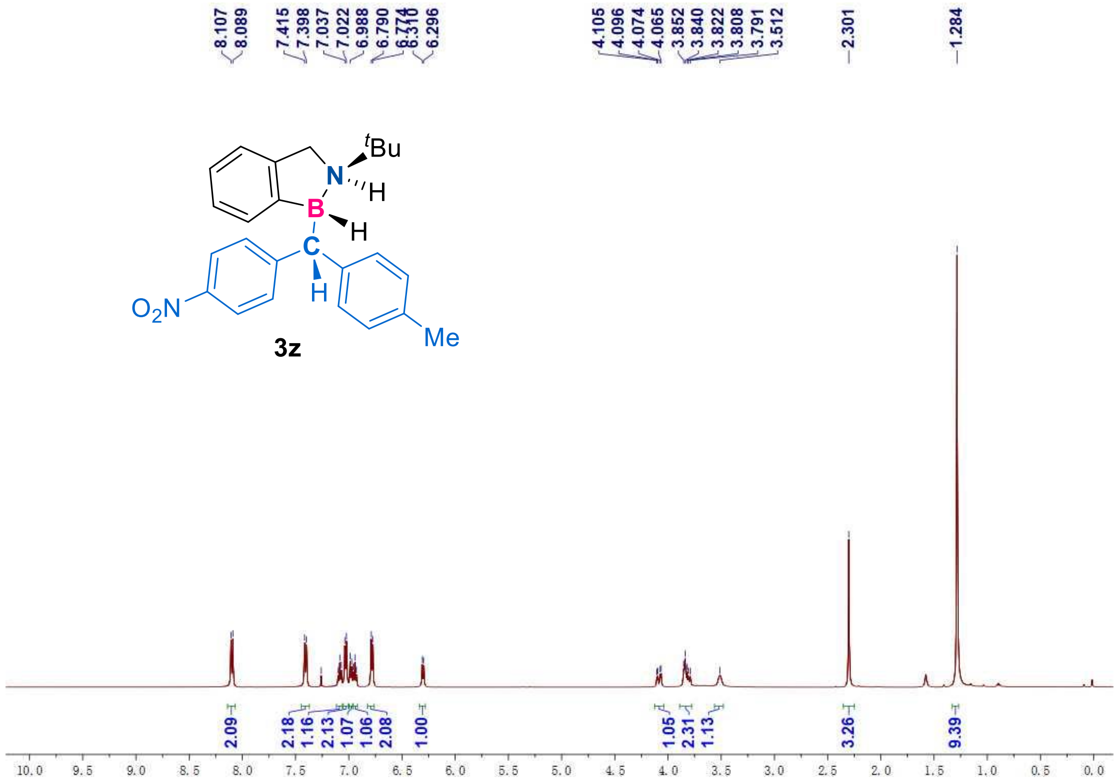

^{13}C NMR (126 MHz, $CDCl_3$)

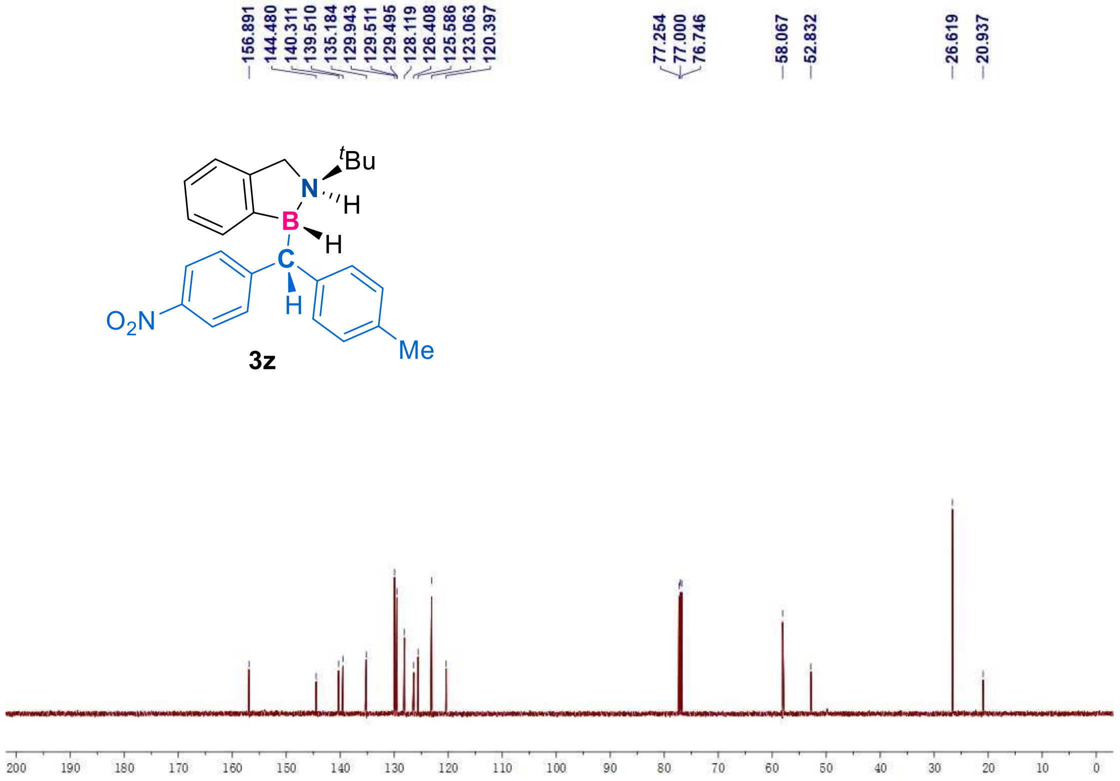

^{11}B NMR (160 MHz, $CDCl_3$)

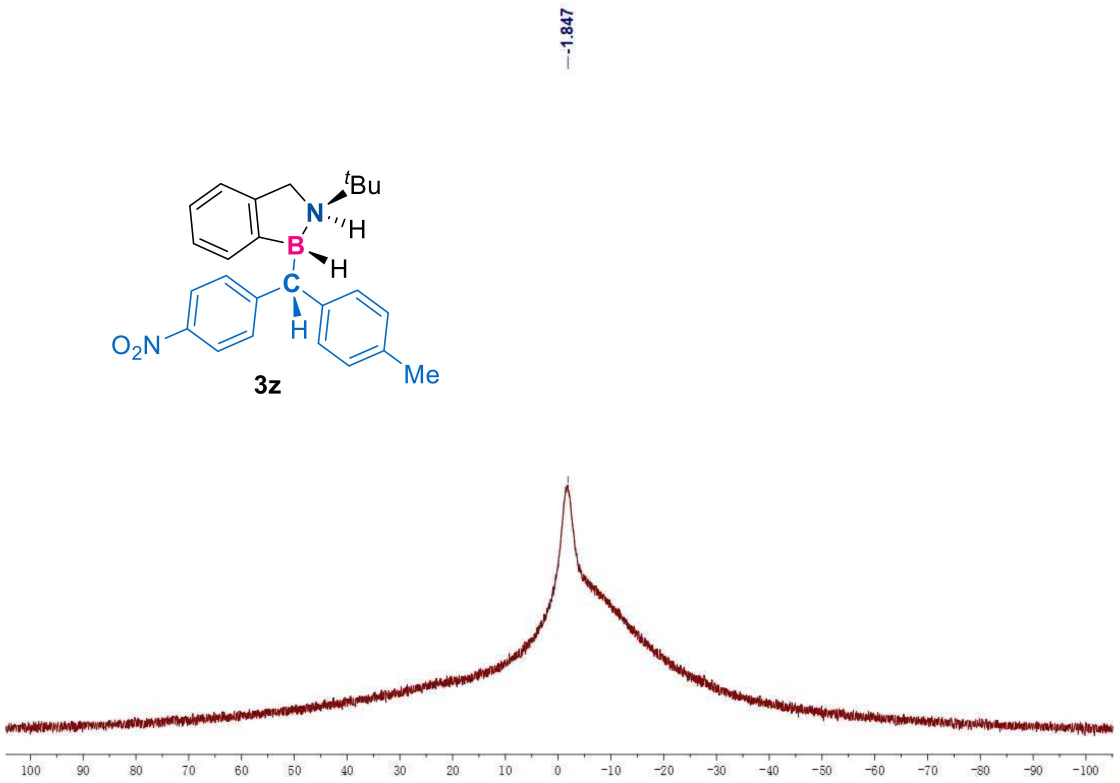

(S)-4-(benzo[d][1,3]dioxol-5-yl(2-(tert-butyl)-2,3-dihydro-1H-benzo[c][1,2]azaborol-1-yl)methyl)benzonitrile (3aa)

^{1}H NMR (500 MHz, $CDCl_3$)

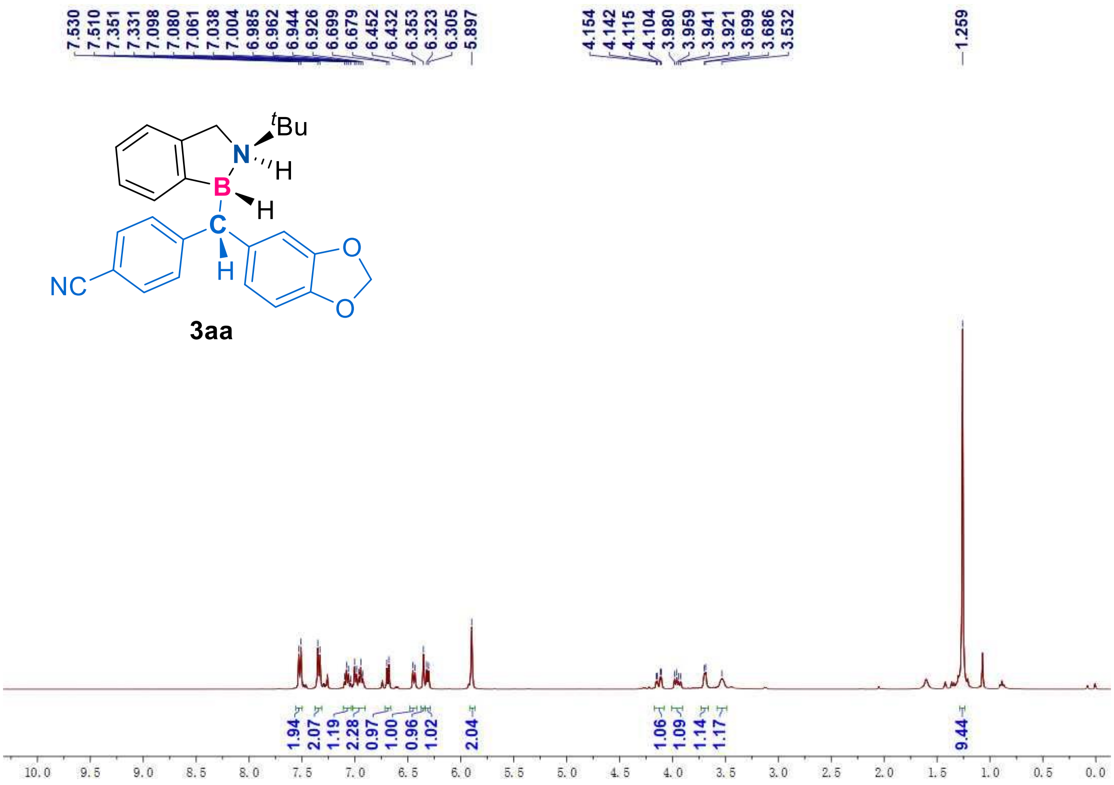

^{13}C NMR (126 MHz, $CDCl_3$)

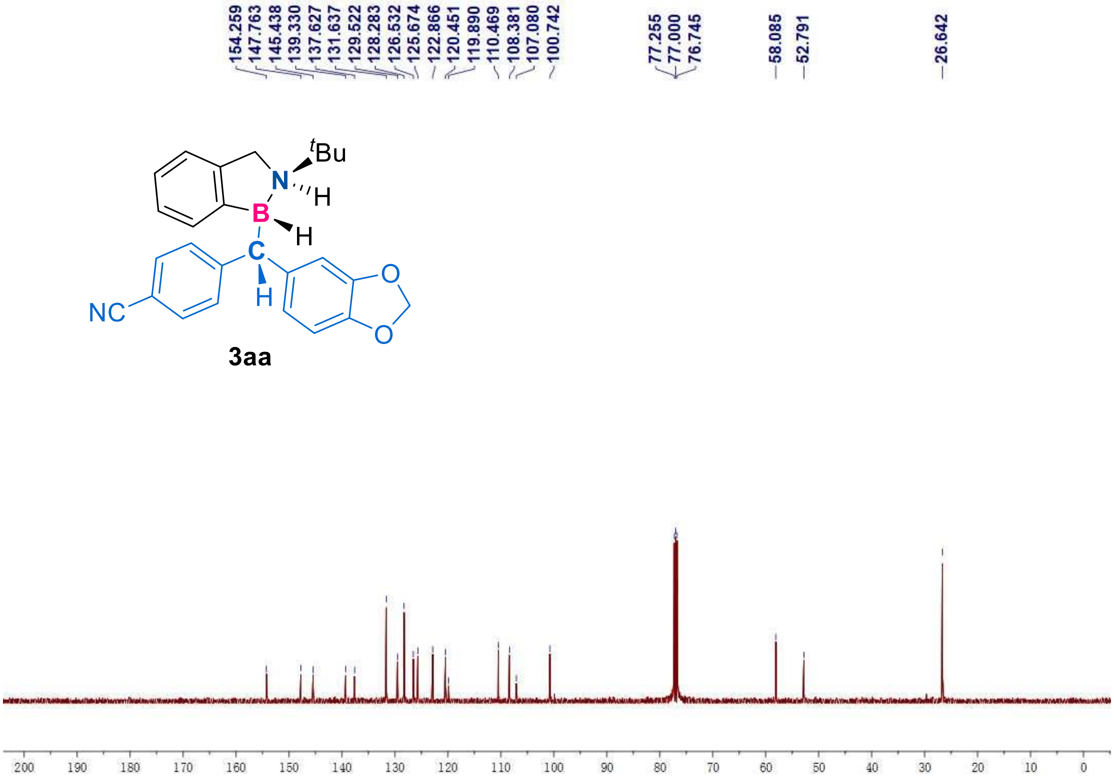

^{11}B NMR (128 MHz, $CDCl_3$)

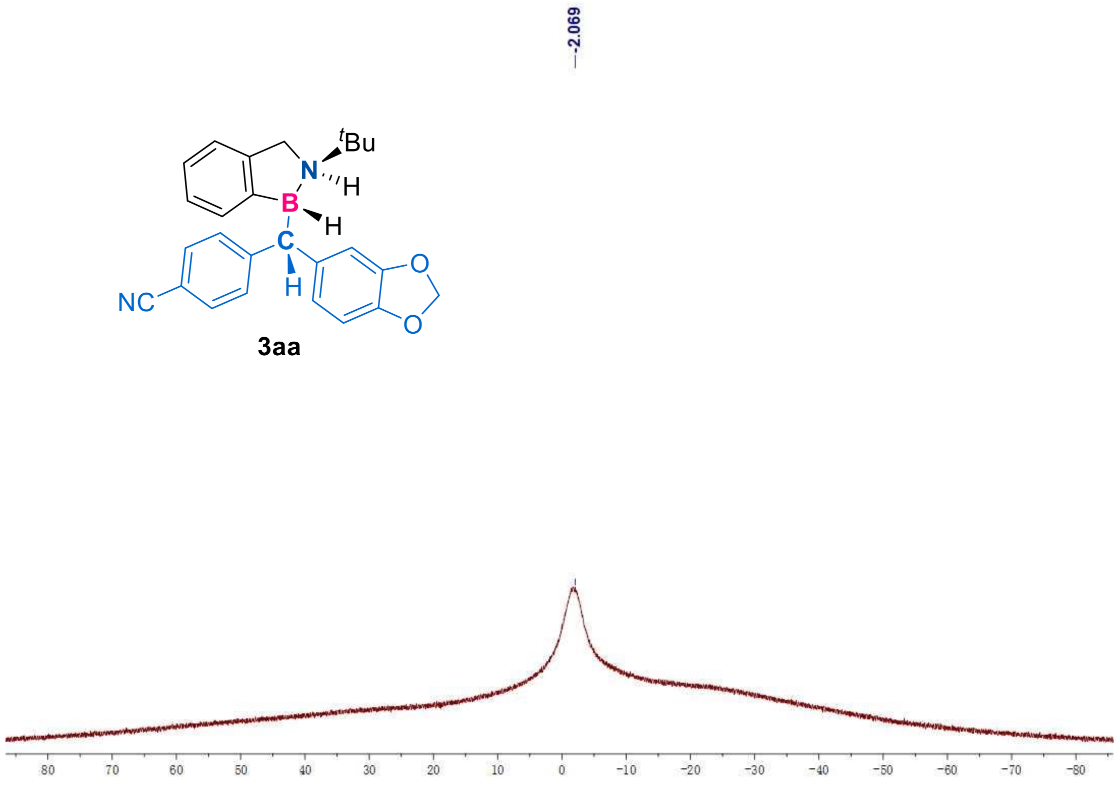

(S)-4-((2-(tert-butyl)-5-methoxy-2,3-dihydro-1H-benzo[c][1,2]azaborol-1-yl)(4-methoxyphenyl)methyl)benzonitrile (3ab)

^{1}H NMR (400 MHz, $CDCl_3$)

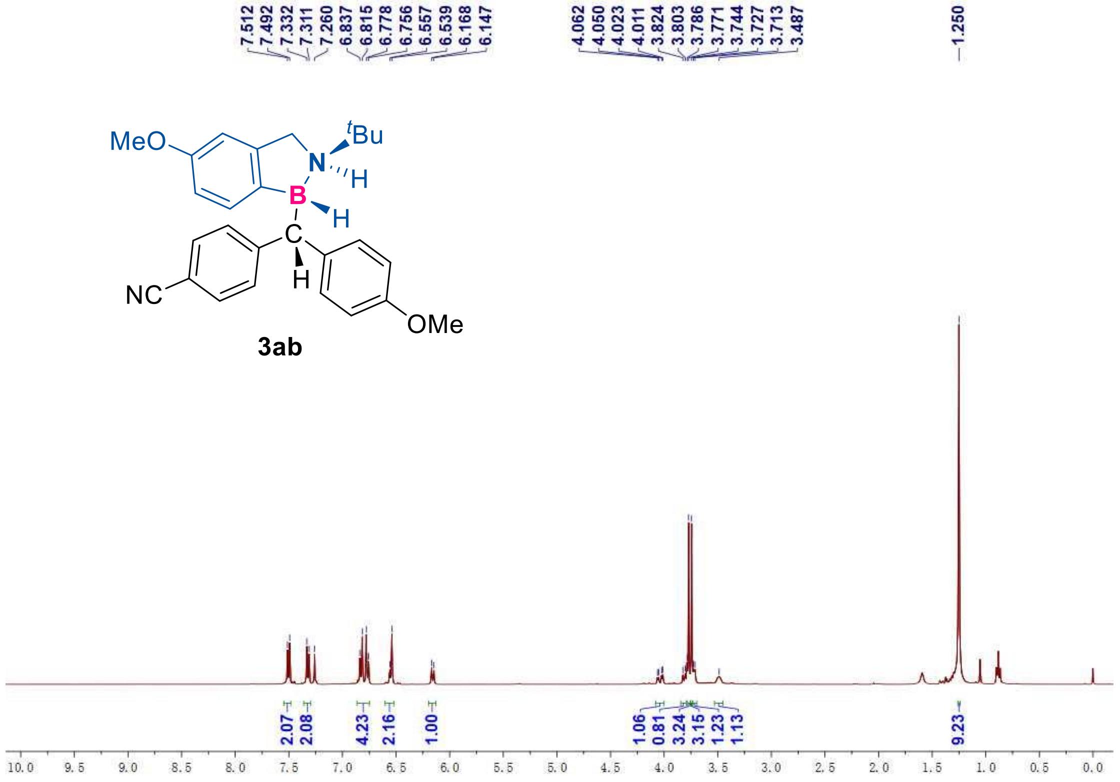

^{13}C **NMR** (126 MHz, $CDCl_3$)

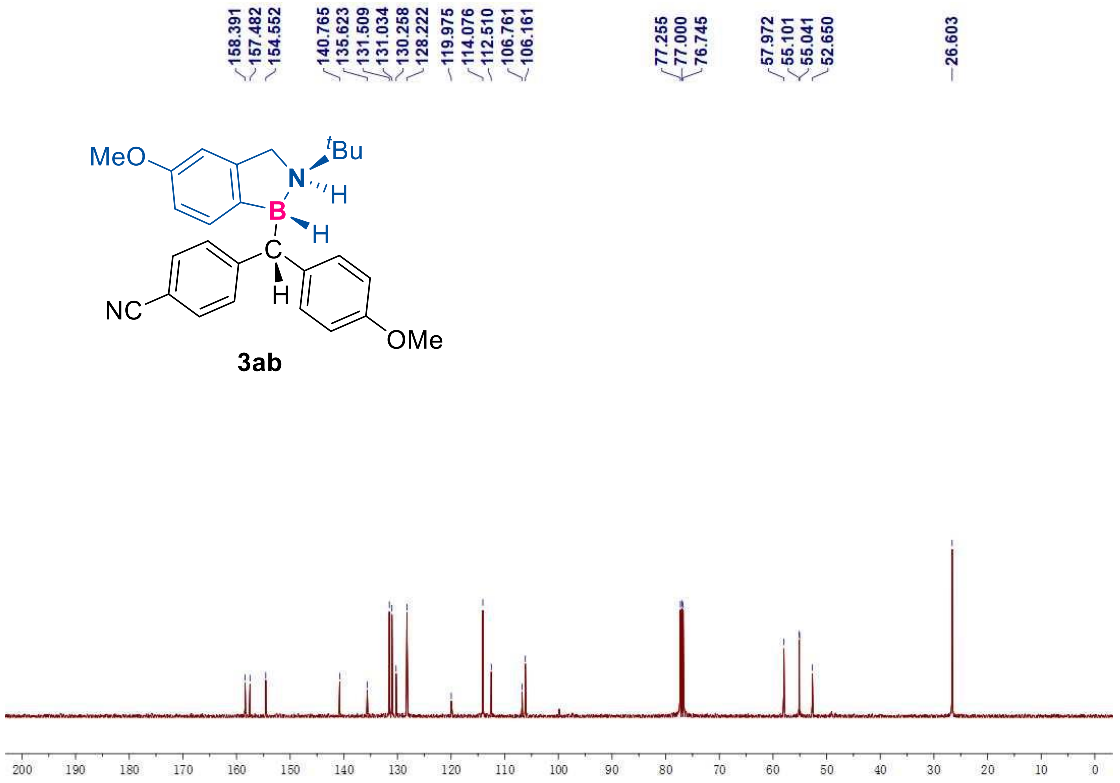

^{11}B **NMR** (128 MHz, $CDCl_3$)

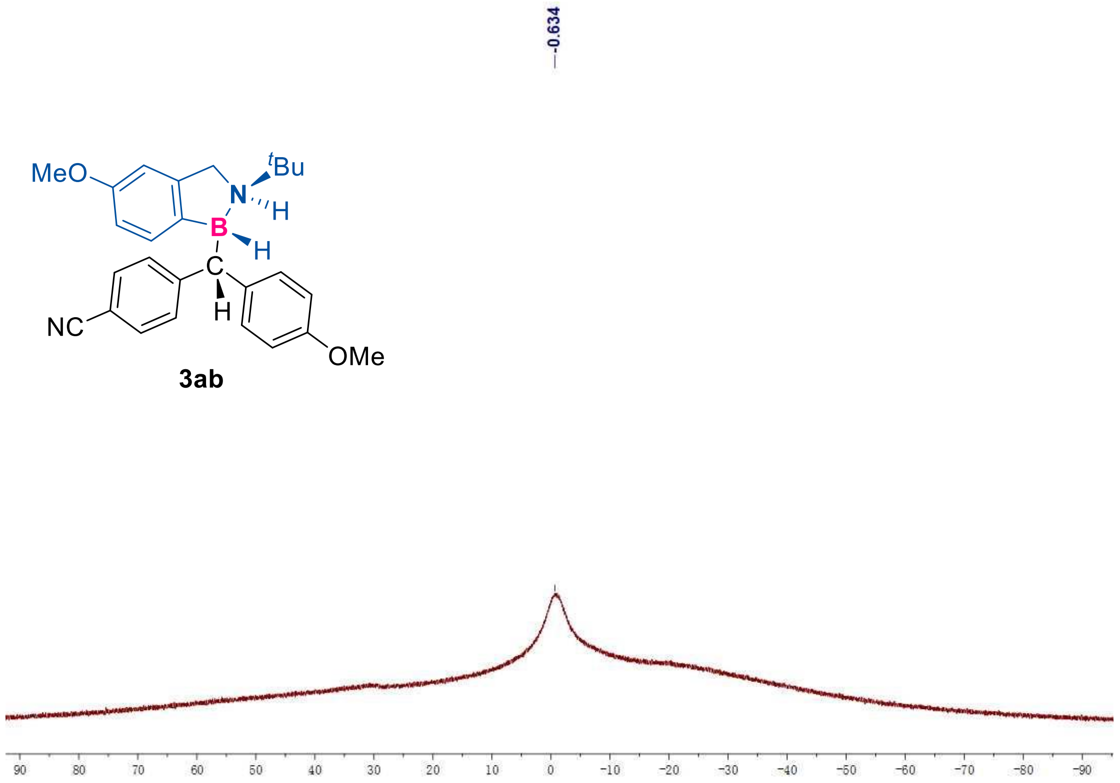

(S)-4-((2-(tert-butyl)-5-methyl-2,3-dihydro-1H-benzo[c][1,2]azaborol-1-yl)(4-methoxyphenyl)methyl)benzonitrile (3ac)

^{1}H NMR (400 MHz, $CDCl_3$)

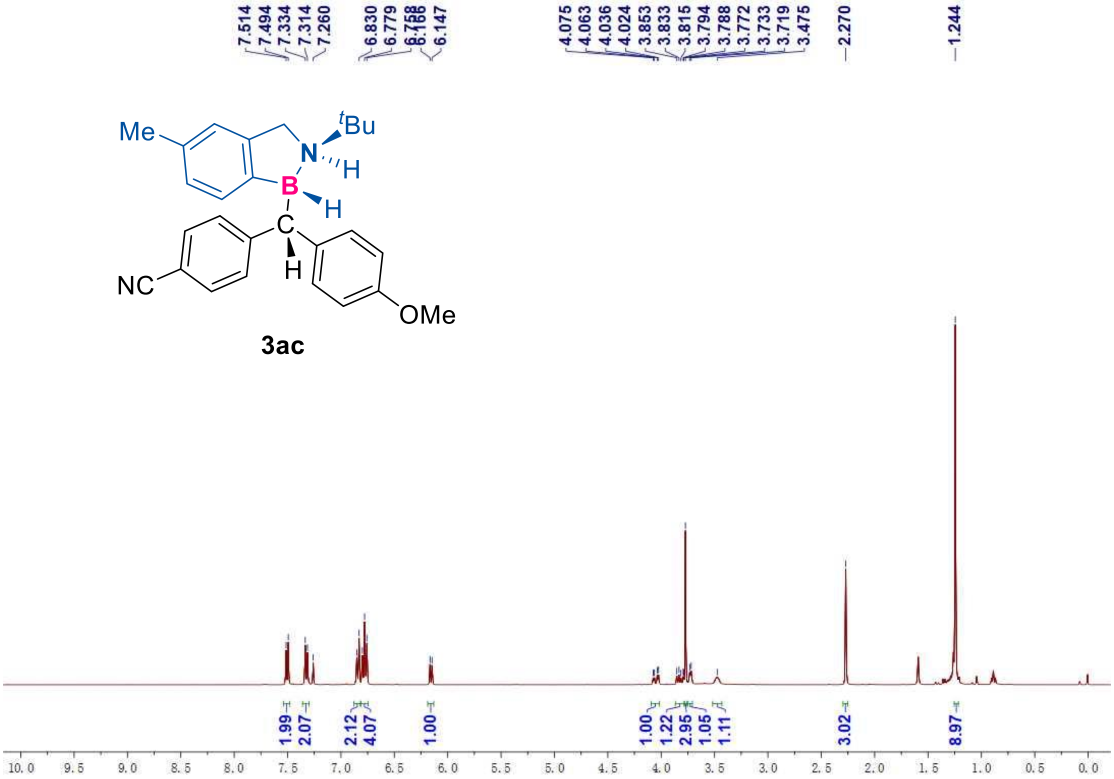

^{13}C NMR (101 MHz, $CDCl_3$)

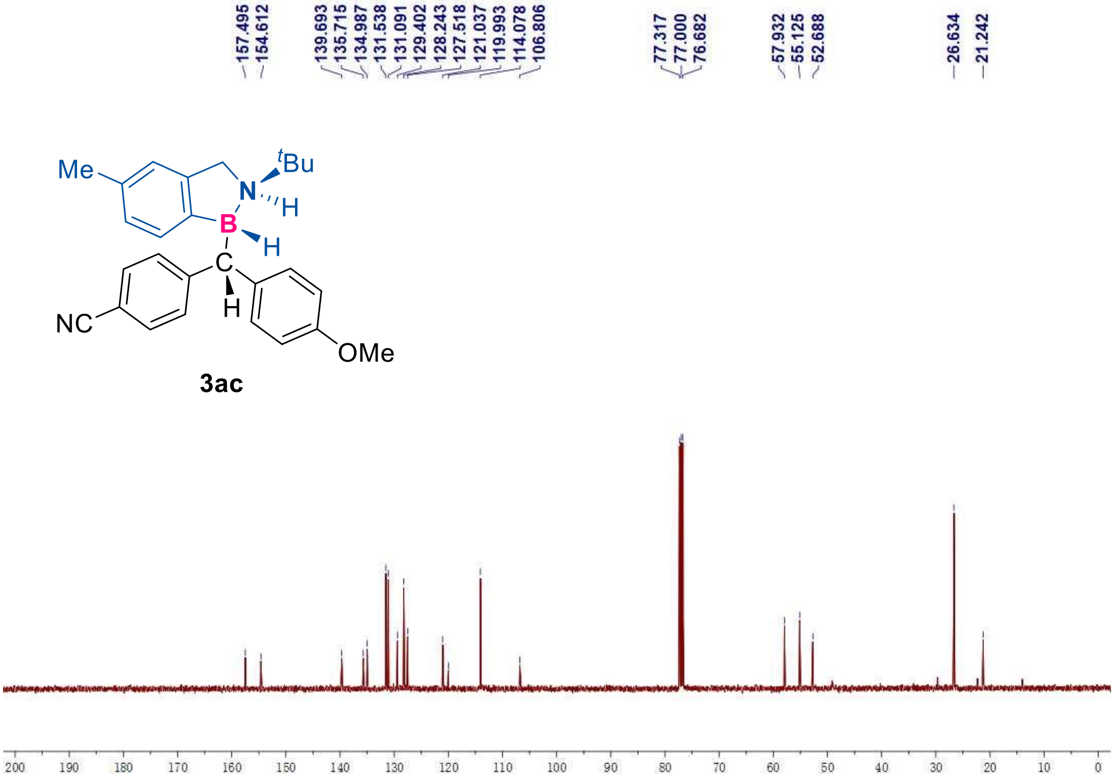

^{11}B NMR (128 MHz, $CDCl_3$)

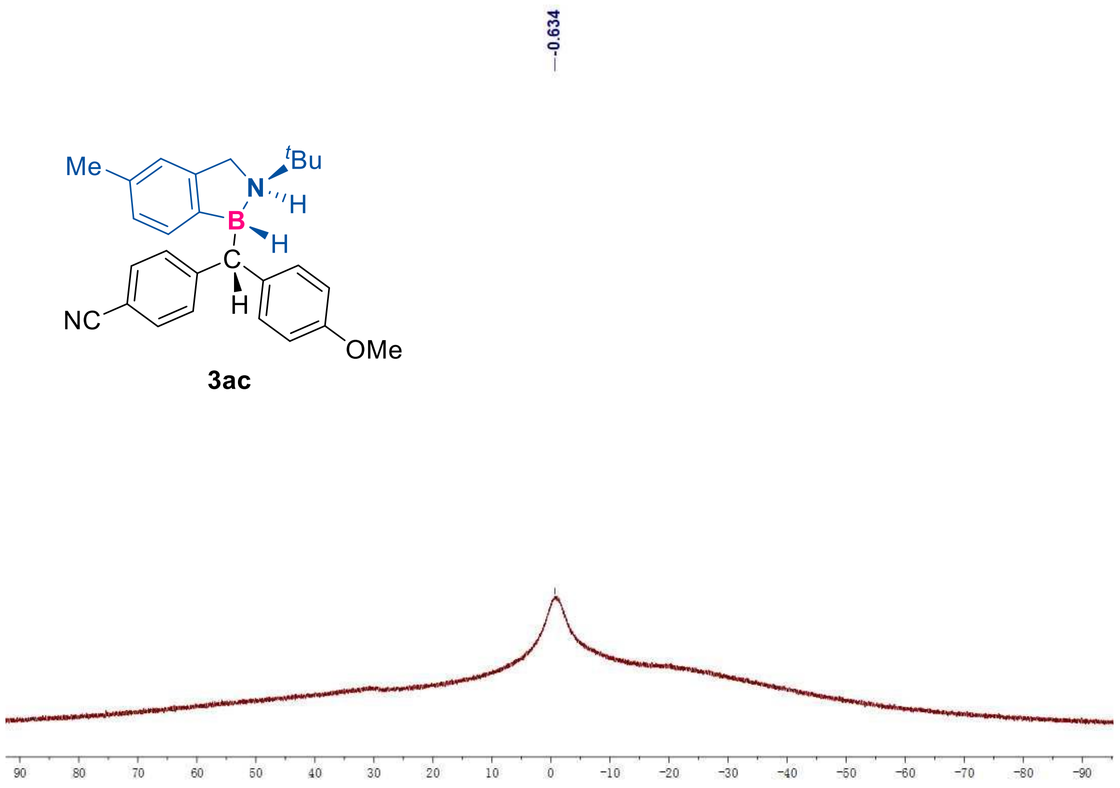

3ac

(S)-4-((2-(tert-butyl)-5-chloro-2,3-dihydro-1H-benzo[c][1,2]azaborol-1-yl)(4-methoxyphenyl)methyl)benzonitrile (**3ad**)

^{1}H NMR (400 MHz, $CDCl_3$)

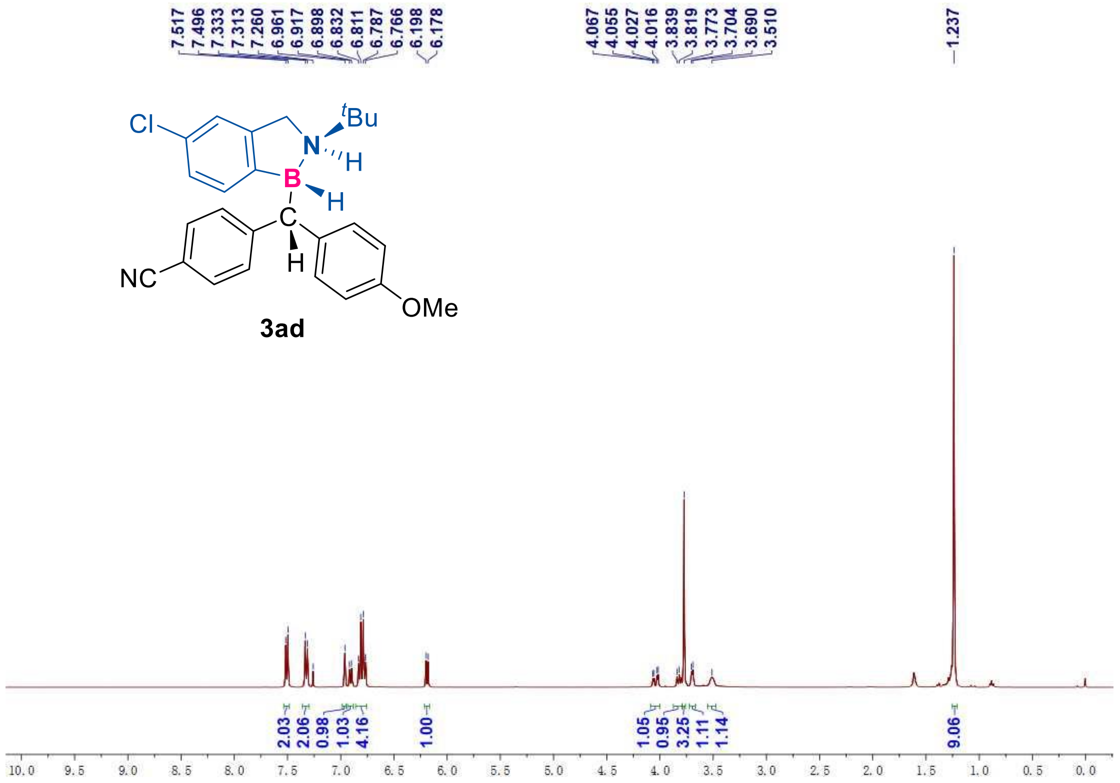

3ad

^{13}**C NMR** (101 MHz, CDCl$_3$)

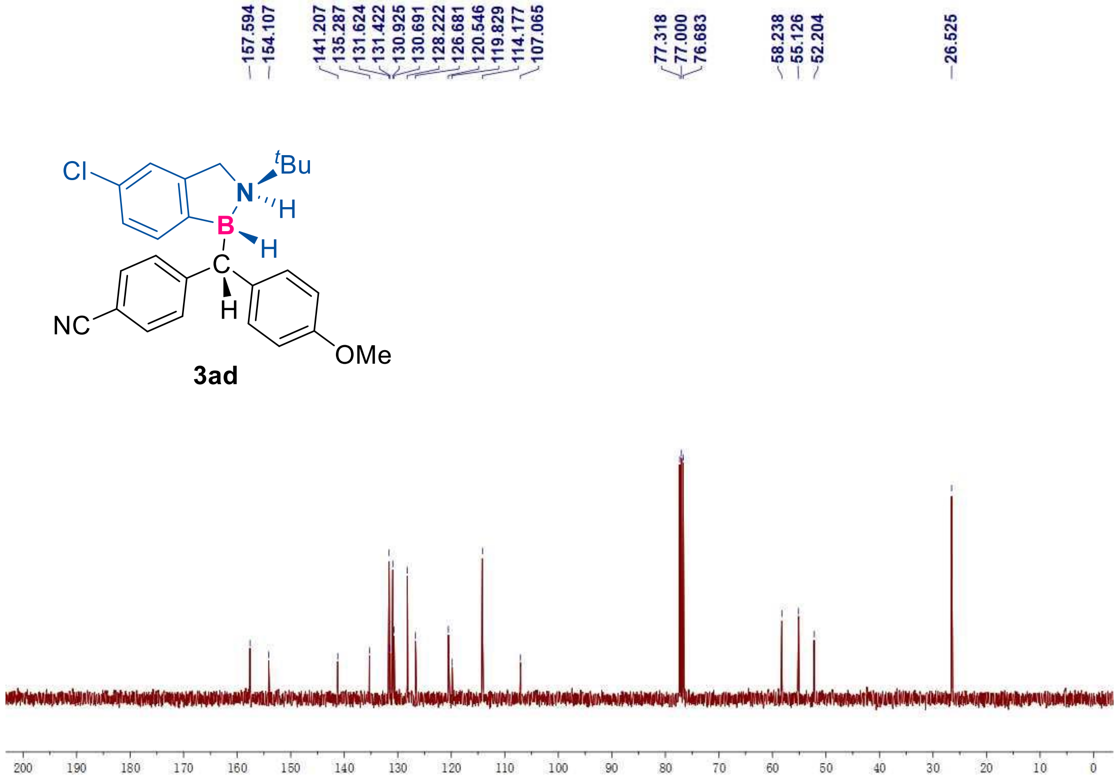

^{11}**B NMR** (128 MHz, CDCl$_3$)

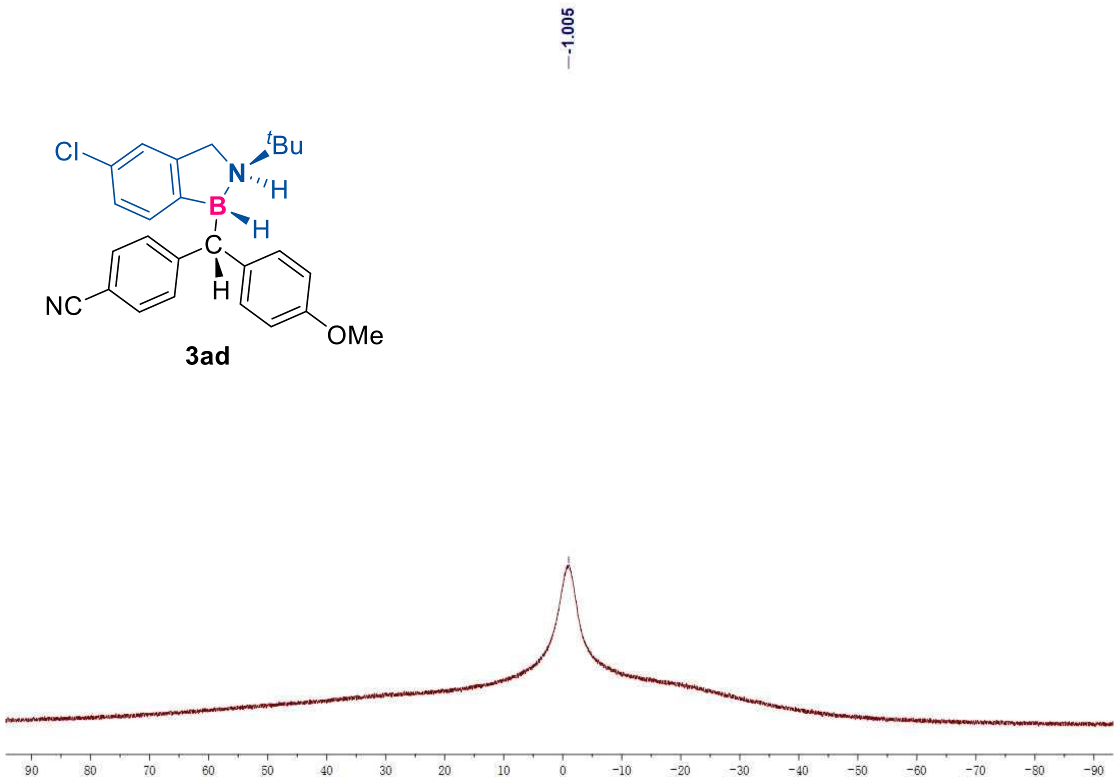

(S)-4-((2-(tert-butyl)-6-fluoro-2,3-dihydro-1H-benzo[c][1,2]azaborol-1-yl)(4-methoxyphenyl)methyl)benzonitrile (3ae)

^{1}H NMR (500 MHz, $CDCl_3$)

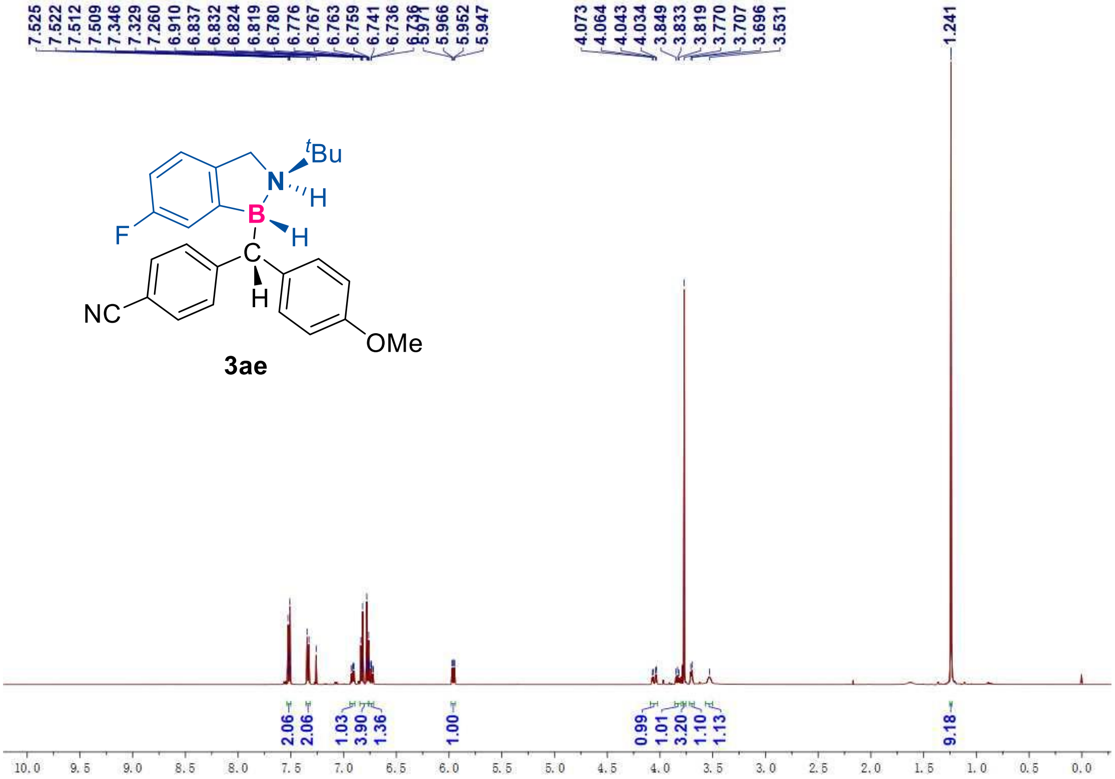

^{13}C NMR (126 MHz, $CDCl_3$)

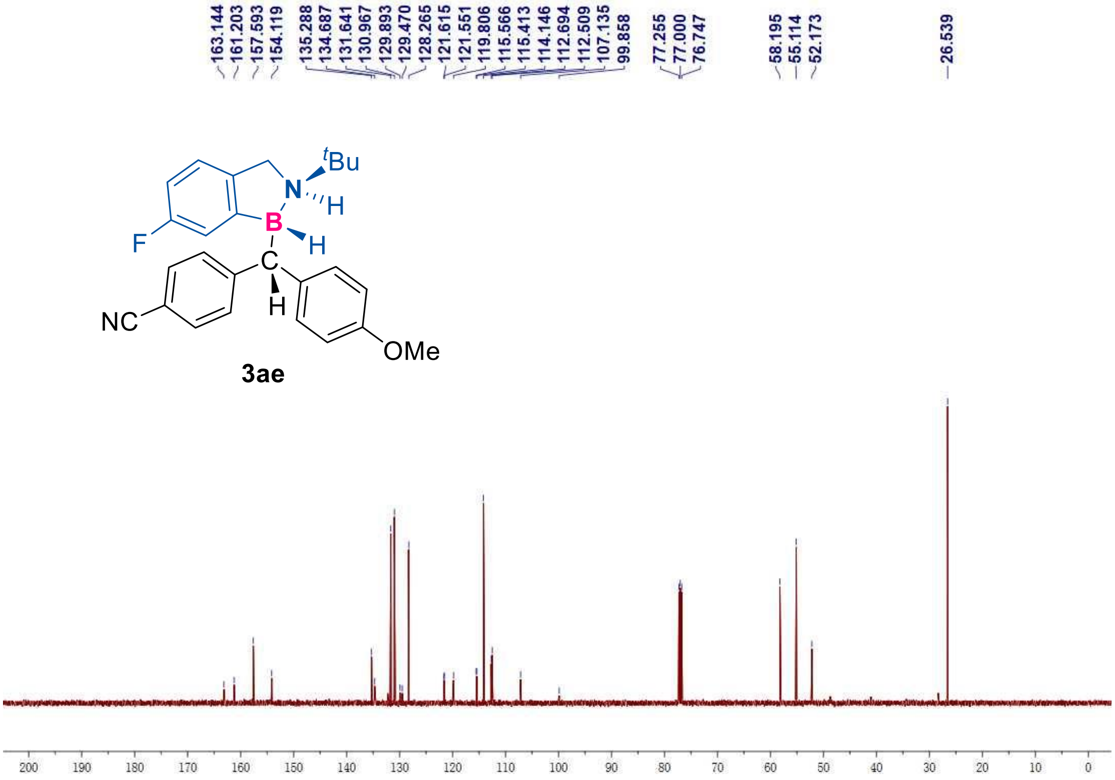

^{11}**B NMR** (160 MHz, $CDCl_3$)

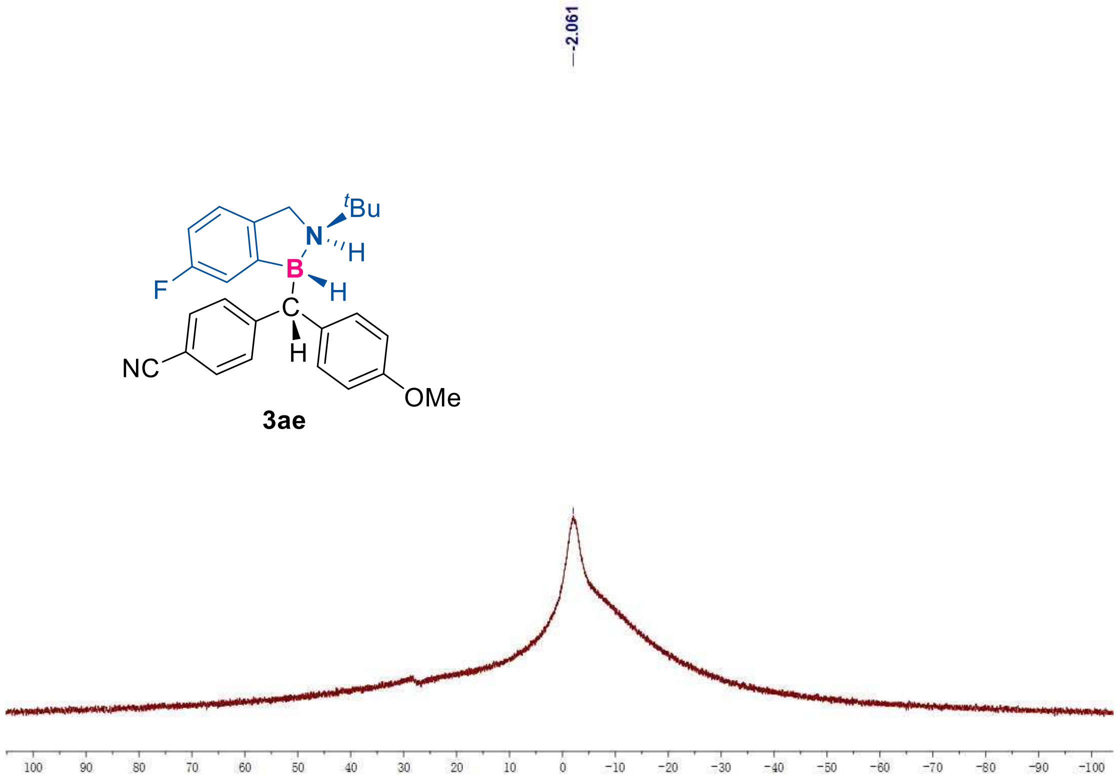

^{19}**F NMR** (471 MHz, $CDCl_3$)

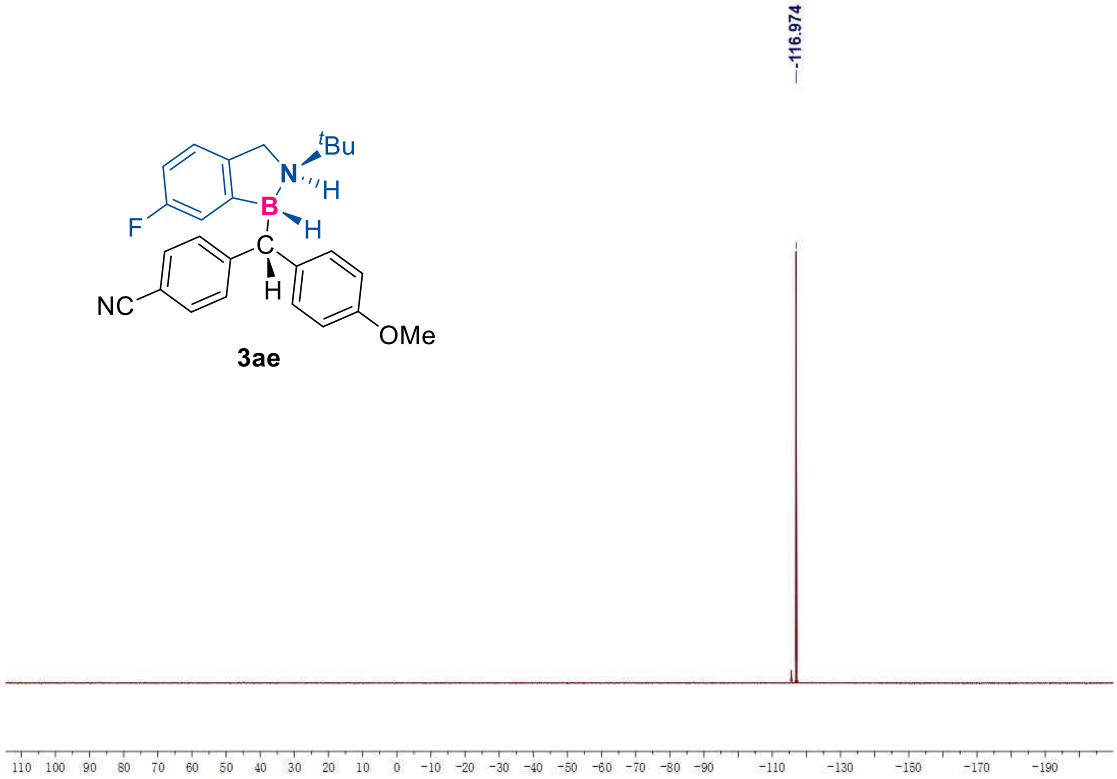

(S)-4-((4-methoxyphenyl)(2-(tert-pentyl)-2,3-dihydro-1H-benzo[c][1,2]azaborol-1-yl)methyl)benzonitrile (3af)

^{1}H NMR (500 MHz, $CDCl_3$)

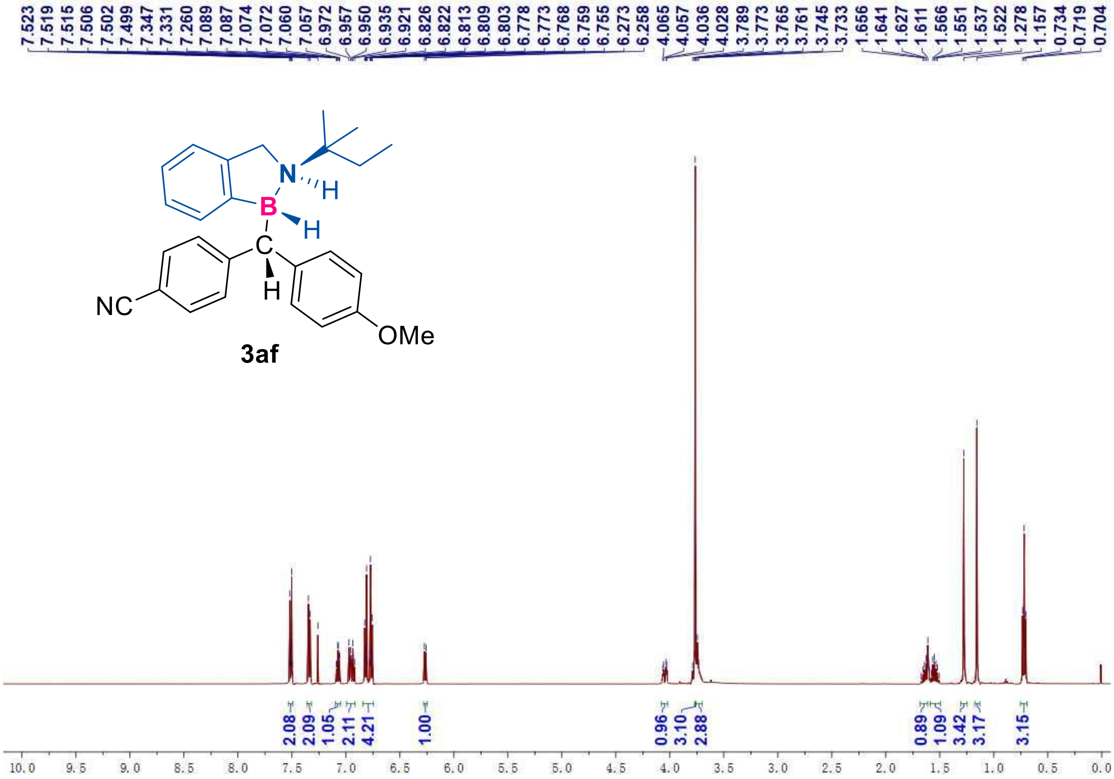

^{13}C NMR (126 MHz, $CDCl_3$)

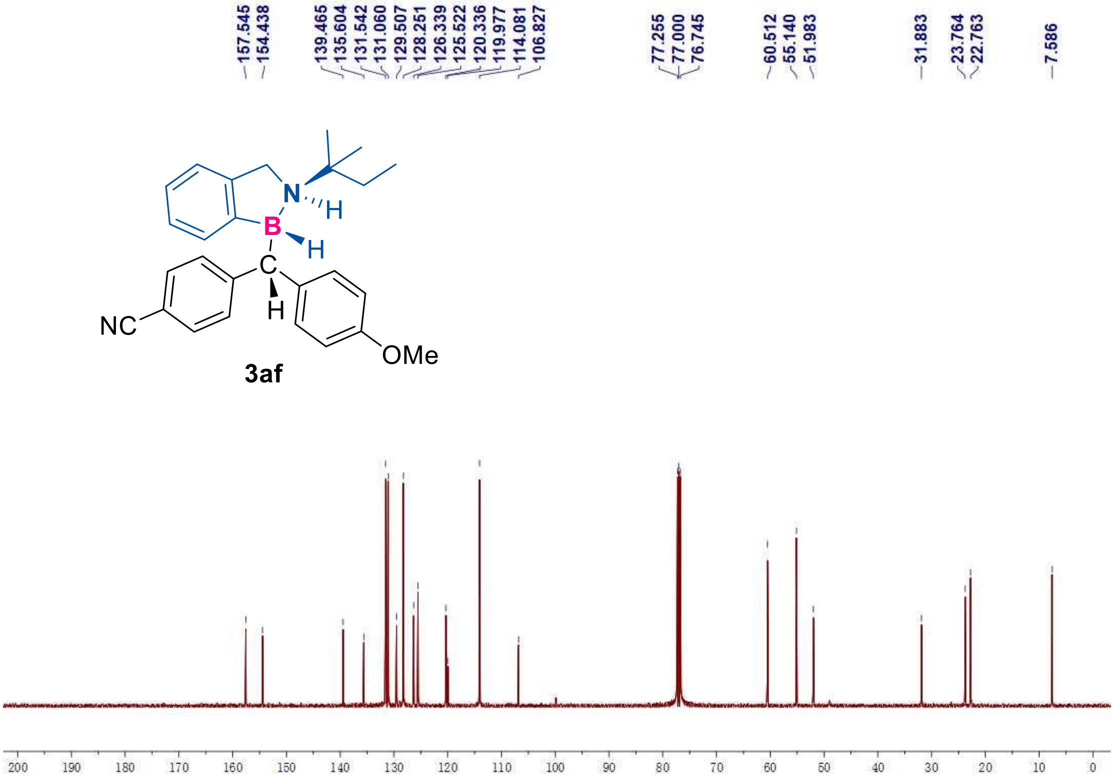

[11]**B NMR** (160 MHz, $CDCl_3$)

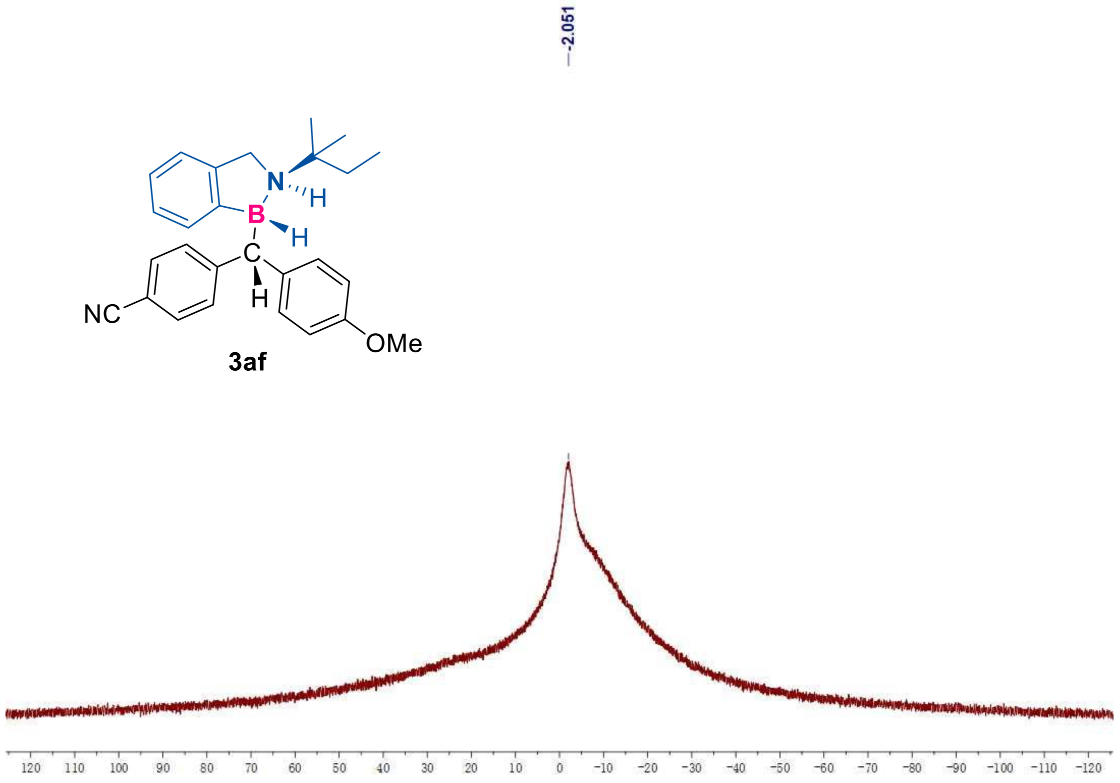

(S)-4-((4-methoxyphenyl)(2-(2,4,4-trimethylpentan-2-yl)-2,3-dihydro-1H-benzo[c][1,2]azaborol-1-yl)methyl)benzonitrile (3ag)

[1]**H NMR** (400 MHz, $CDCl_3$)

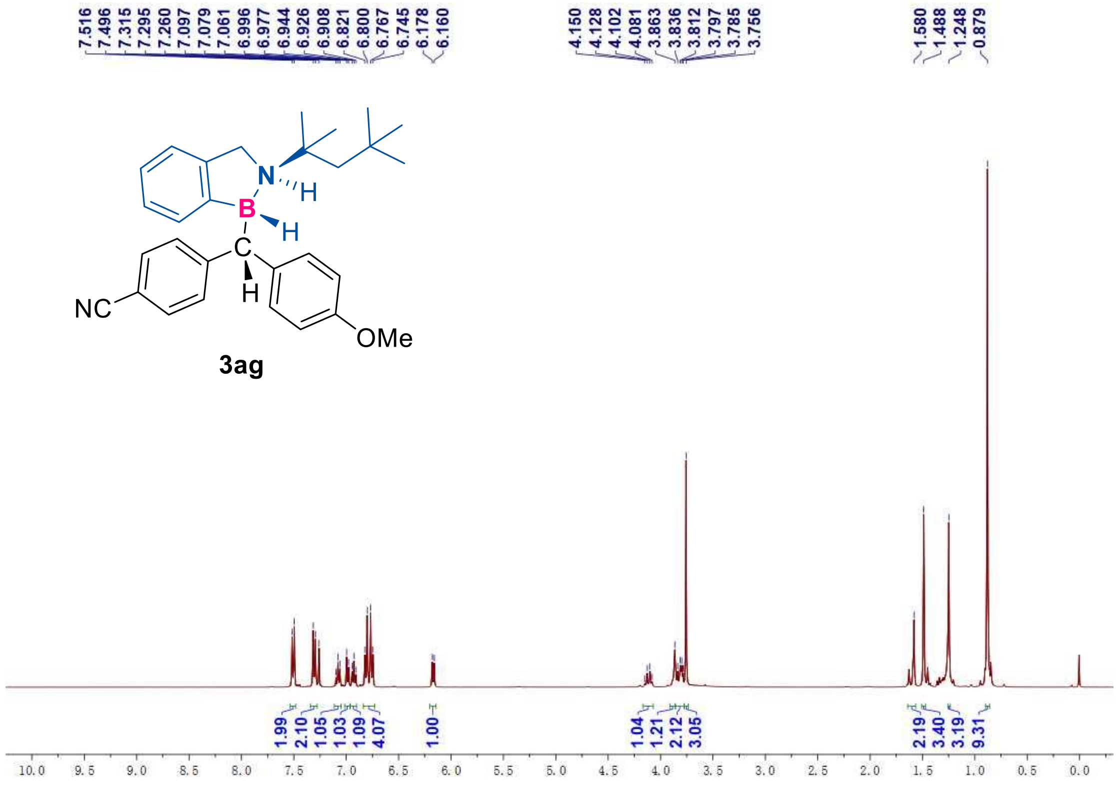

^{13}**C NMR** (101 MHz, $CDCl_3$)

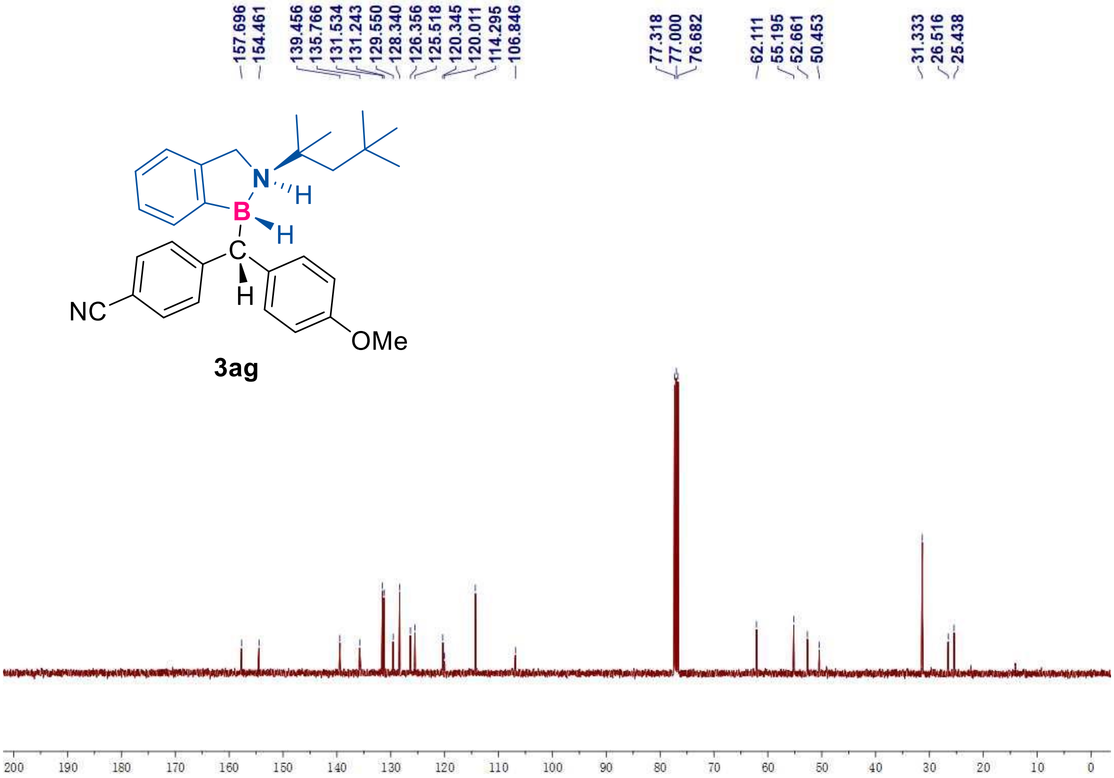

^{11}**B NMR** (128 MHz, $CDCl_3$)

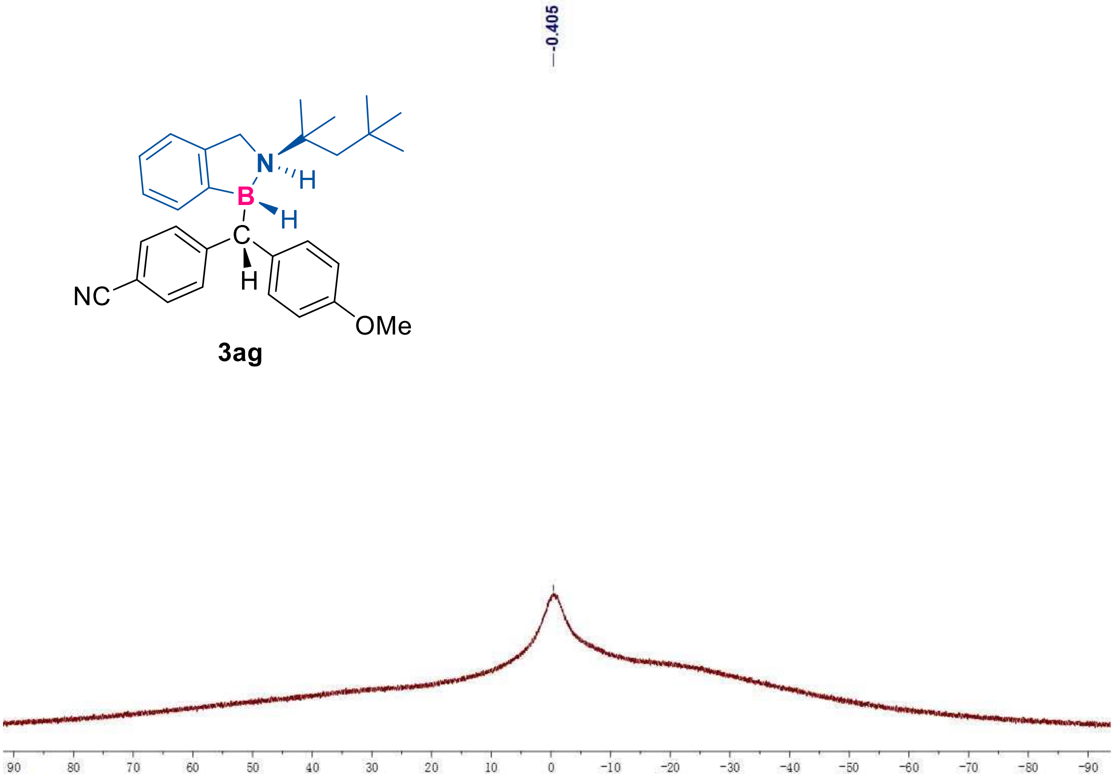

(S)-4-((4-methoxyphenyl)(2-(2-phenylpropan-2-yl)-2,3-dihydro-1H-benzo[c][1,2]azaborol-1-yl)methyl)benzonitrile (**3ah**)

^{1}H NMR (500 MHz, $CDCl_3$)

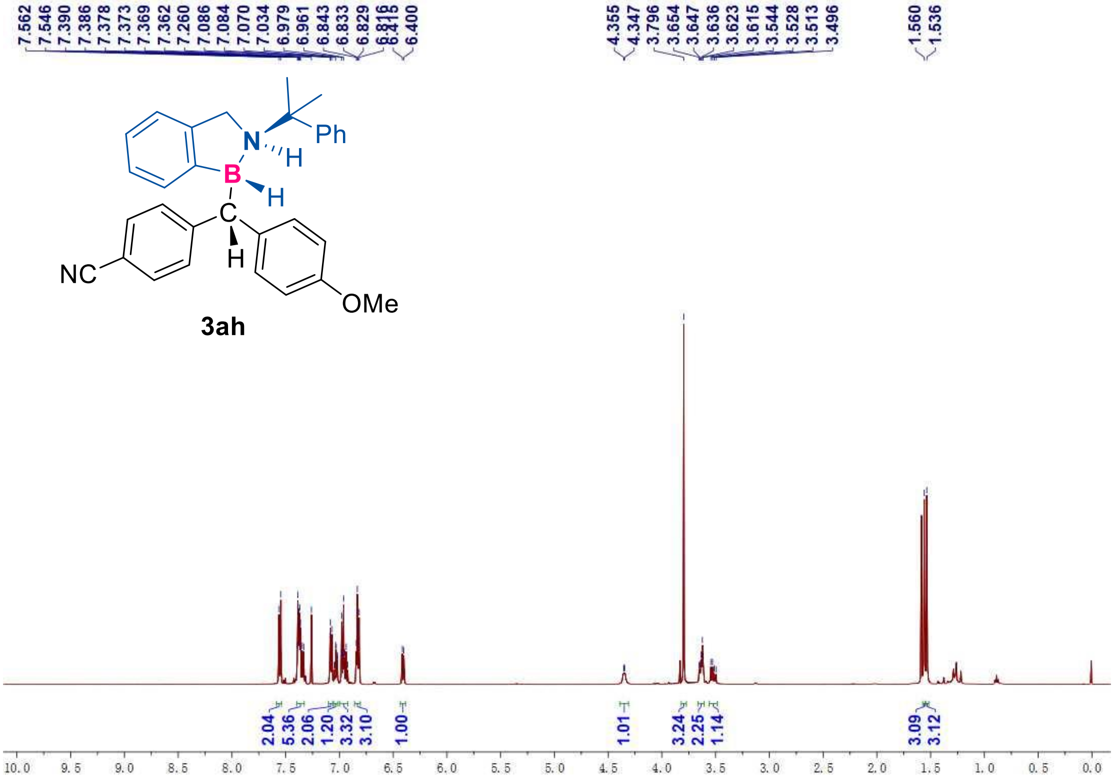

^{13}C NMR (101 MHz, $CDCl_3$)

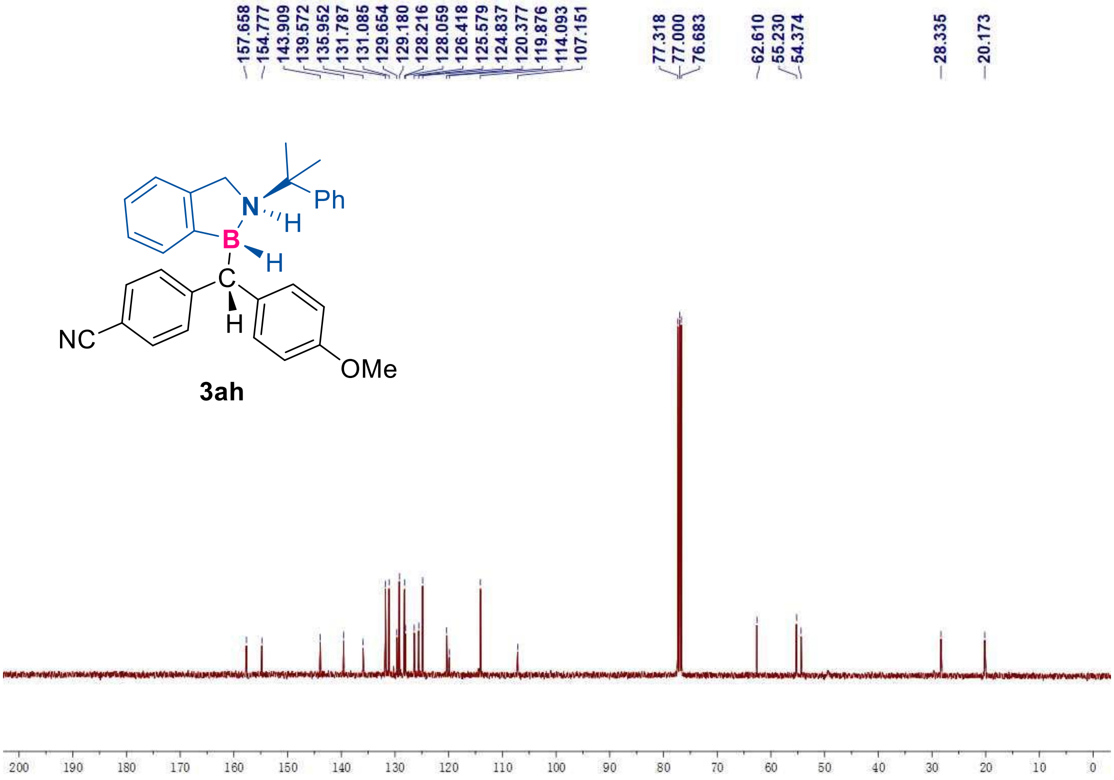

^{11}B NMR (128 MHz, $CDCl_3$)

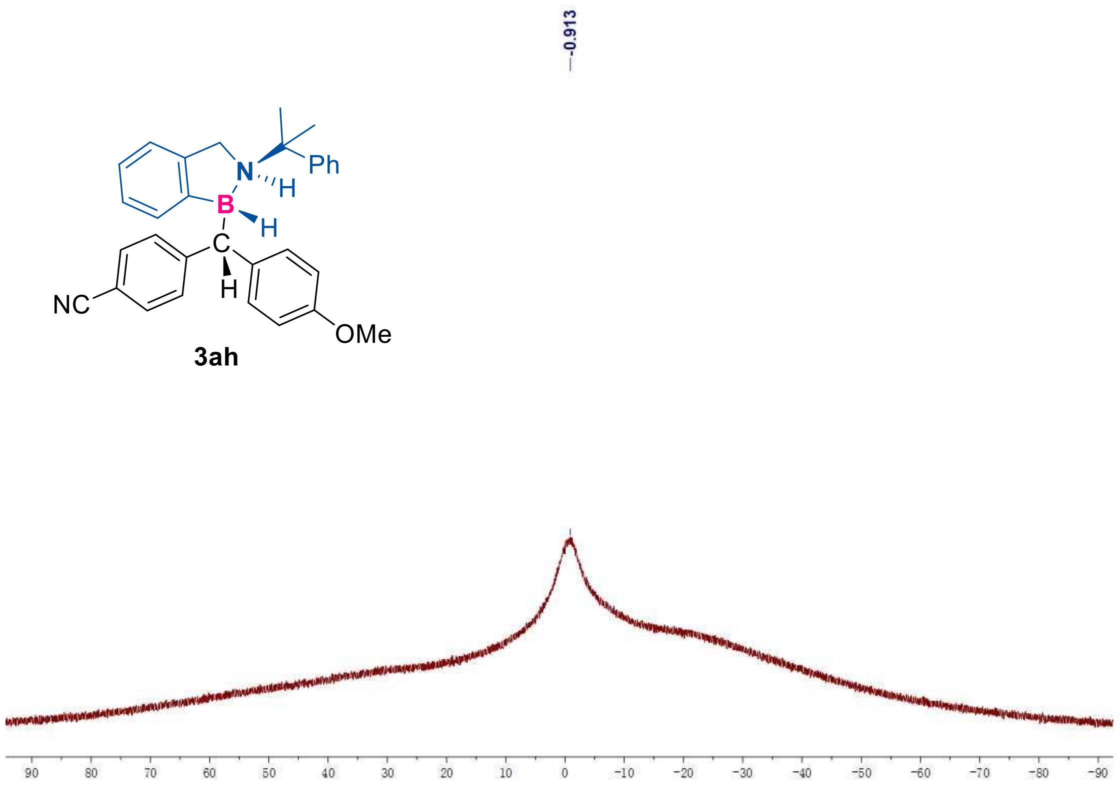

(S)-4-((2-(1-hydroxy-2-methylpropan-2-yl)-2,3-dihydro-1H-benzo[c][1,2]azaborol-1-yl)(4-methoxyphenyl)methyl)benzonitrile (3ai)

^{1}H NMR (400 MHz, $CDCl_3$)

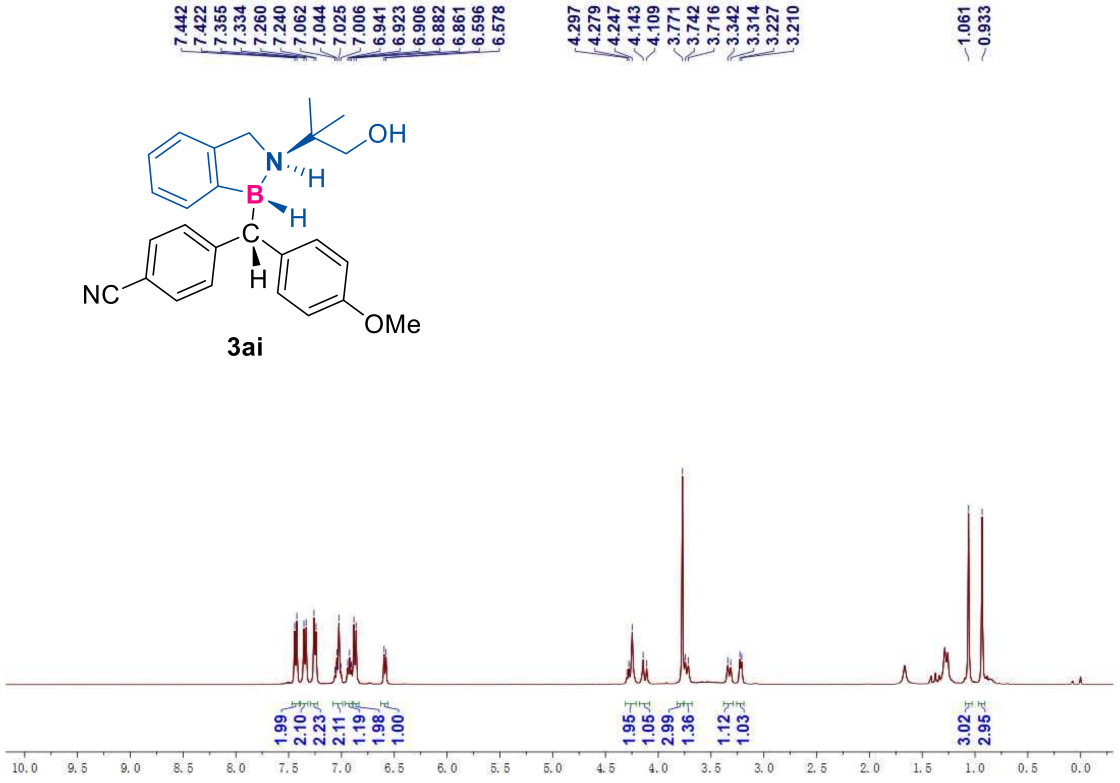

^{13}C NMR (101 MHz, $CDCl_3$)

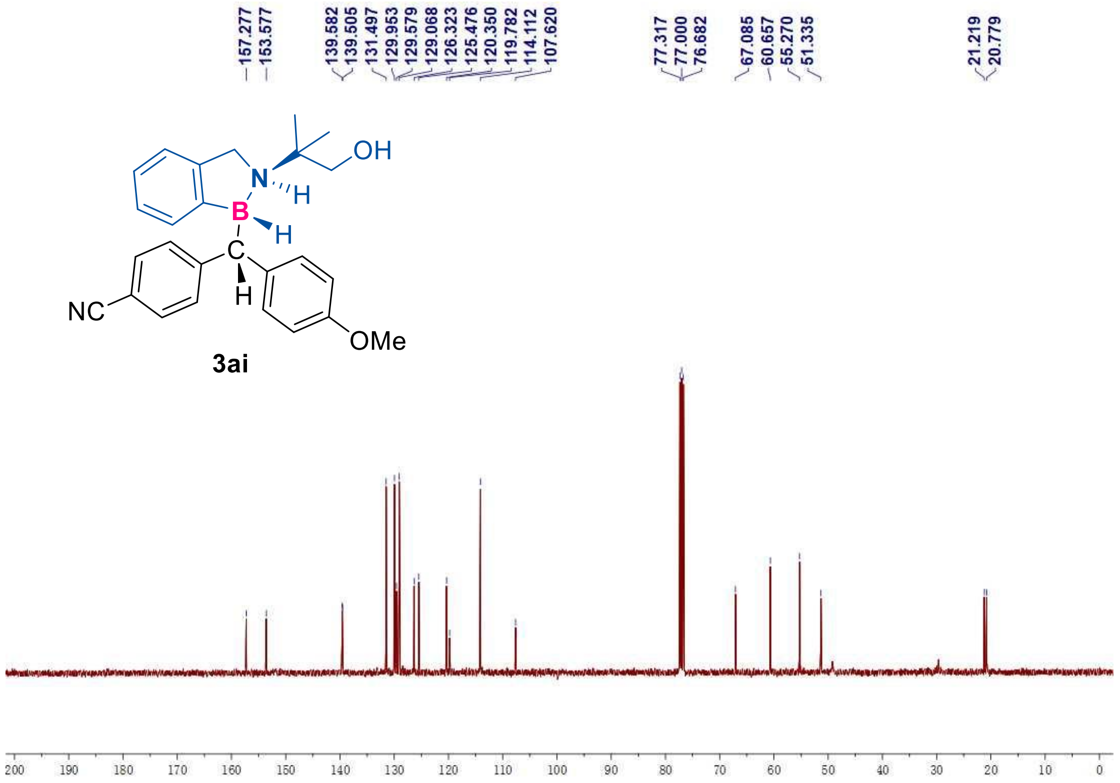

^{11}B NMR (128 MHz, $CDCl_3$)

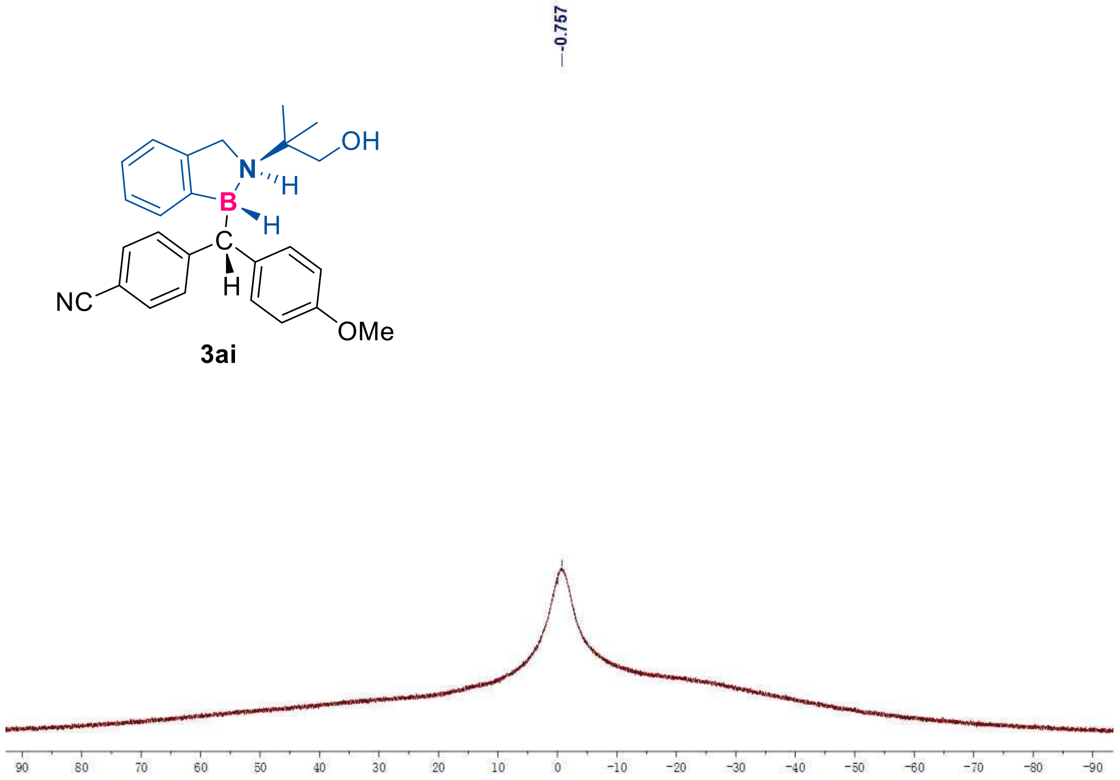

(S)-4-((4-methoxyphenyl)(2-(3-methylbenzyl)-2,3-dihydro-1H-benzo[c][1,2]azaborol-1-yl)methyl)benzonitrile (3aj)

^{1}H NMR (400 MHz, $CDCl_3$)

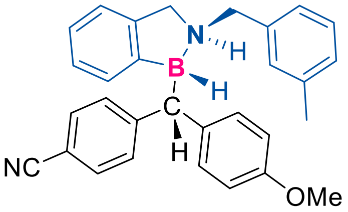

3aj

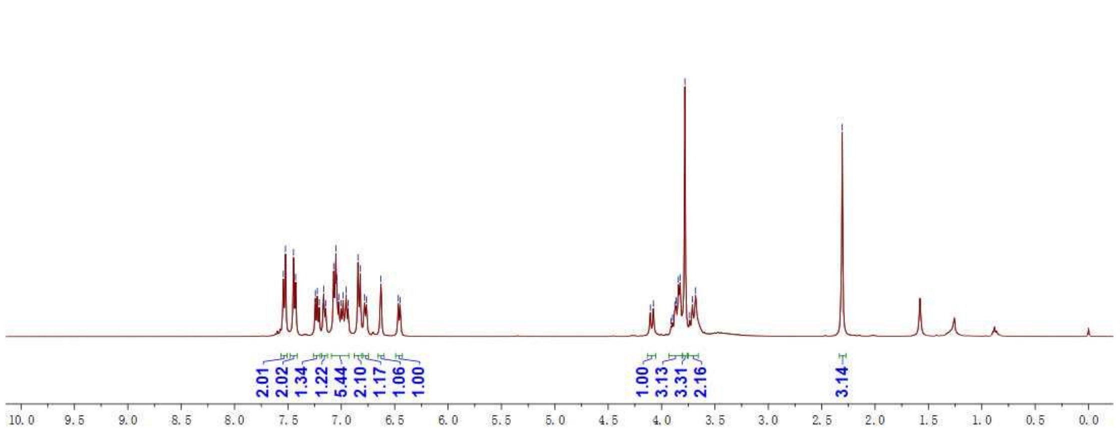

^{13}C NMR (126 MHz, $CDCl_3$)

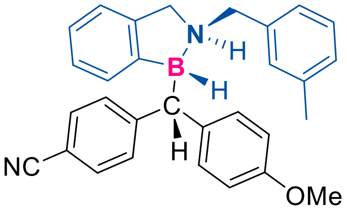

3aj

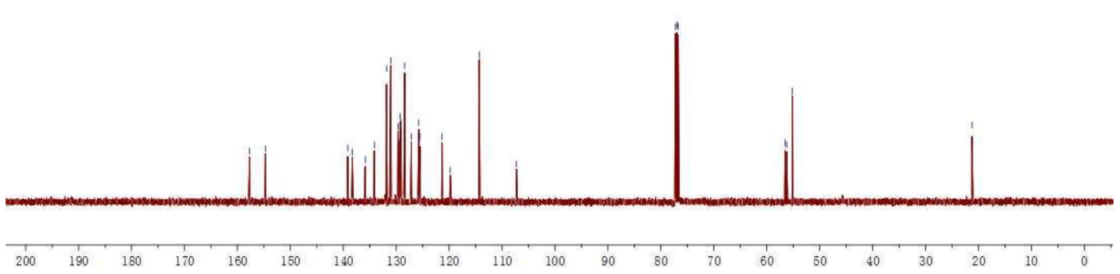

[11]**B NMR** (160 MHz, $CDCl_3$)

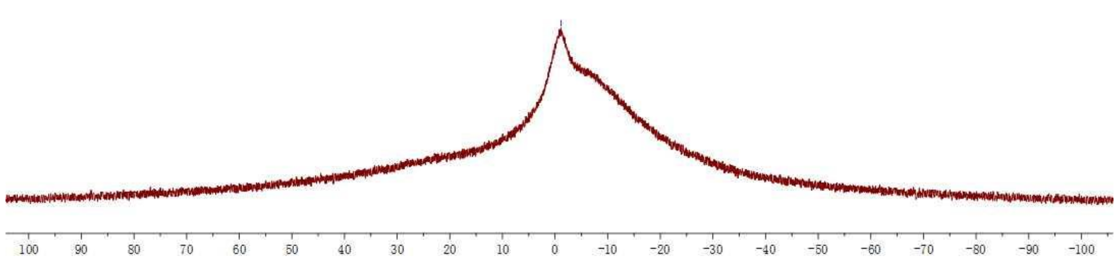

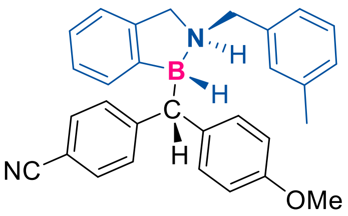

3aj

1-benzhydryl-2-(tert-butyl)-1,2,3,4-tetrahydrobenzo[c][1,2]azaborinine (**3ak**)

[1]**H NMR** (500 MHz, $CDCl_3$)

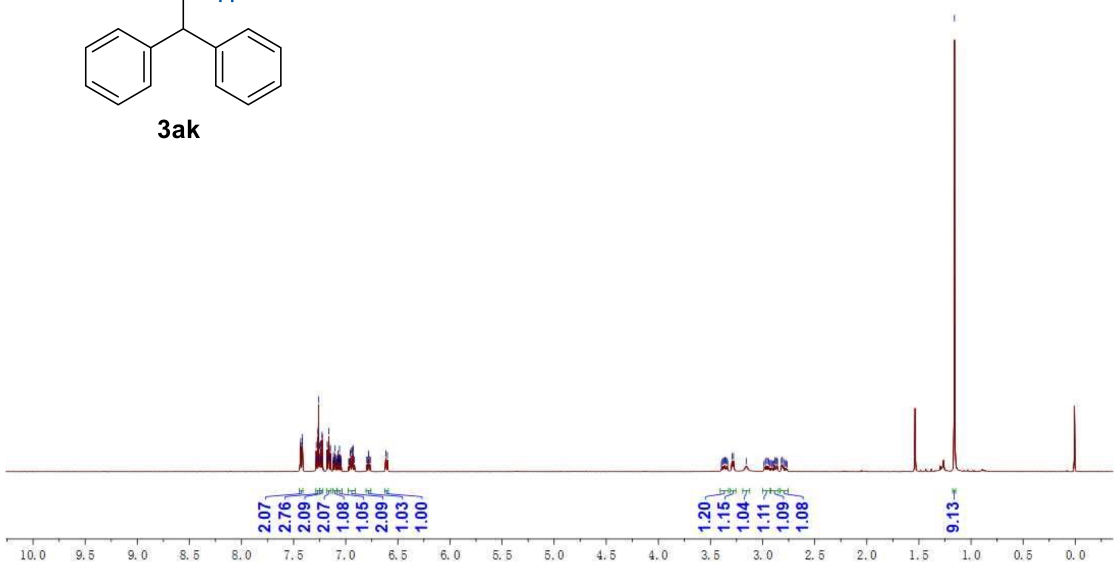

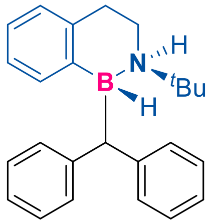

3ak

^{13}C **NMR** (126 MHz, $CDCl_3$)

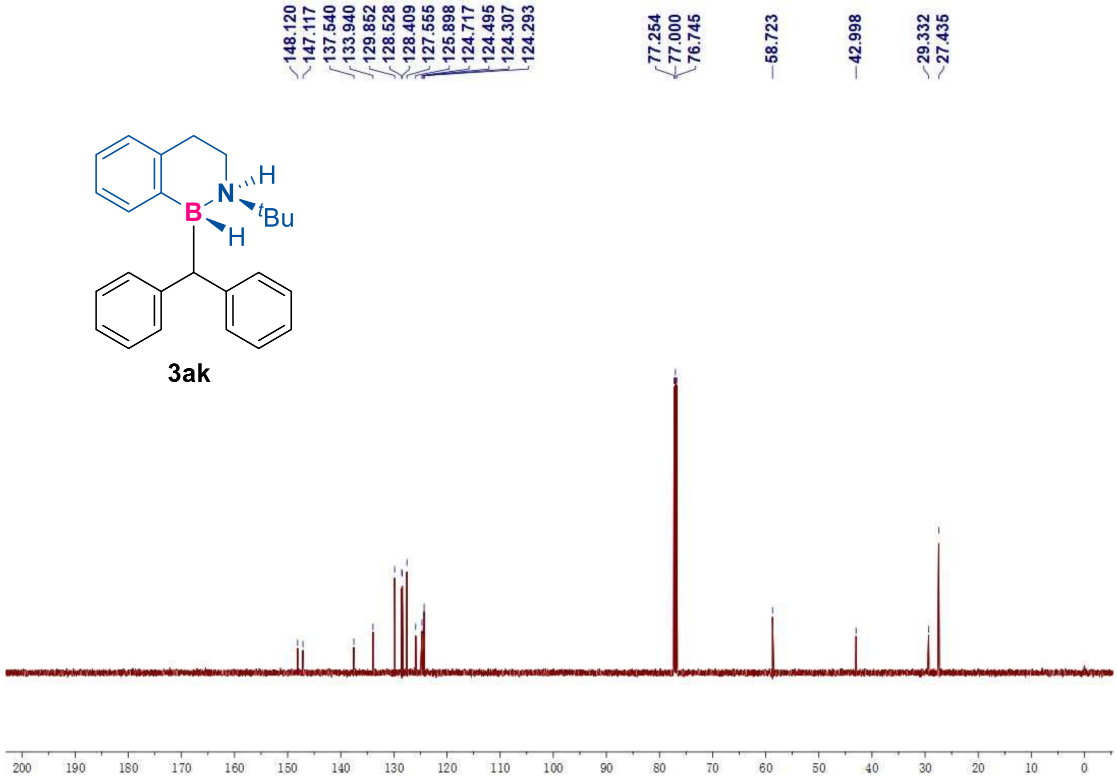

^{11}B **NMR** (160 MHz, $CDCl_3$)

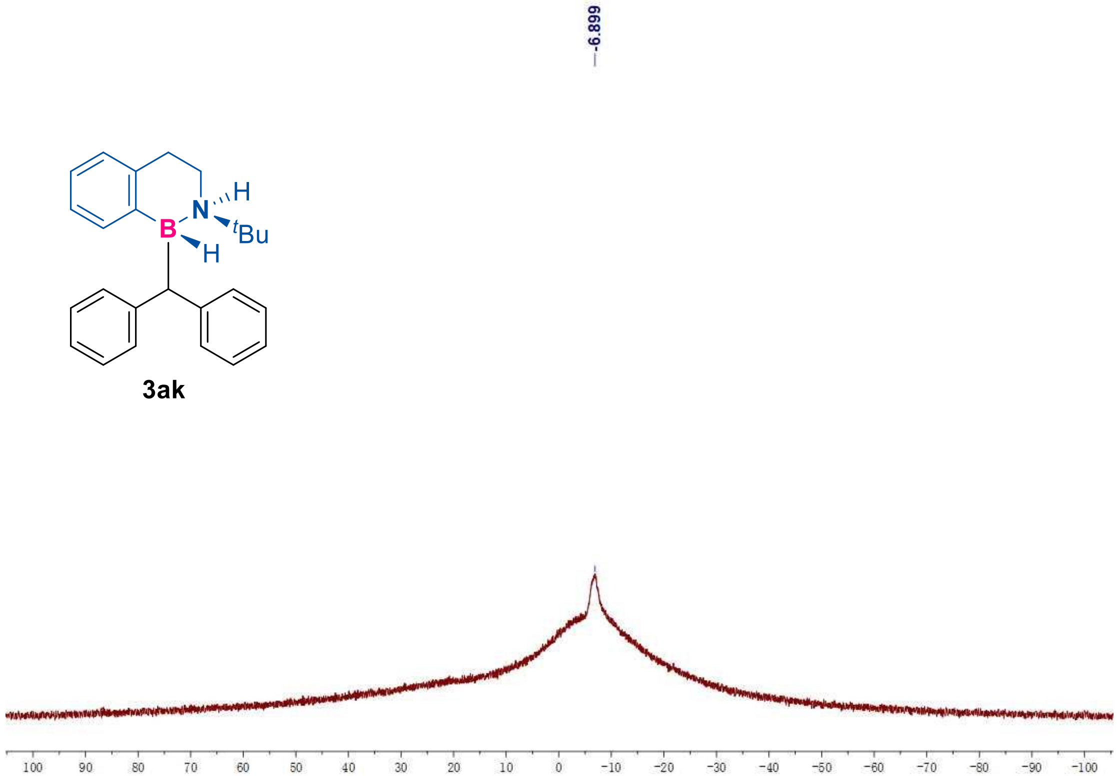

1-benzhydryl-2-(tert-butyl)-5,6-dimethoxy-2,3-dihydro-1H-benzo[c][1,2]azaborole (3al)

^{1}H NMR (500 MHz, $CDCl_3$)

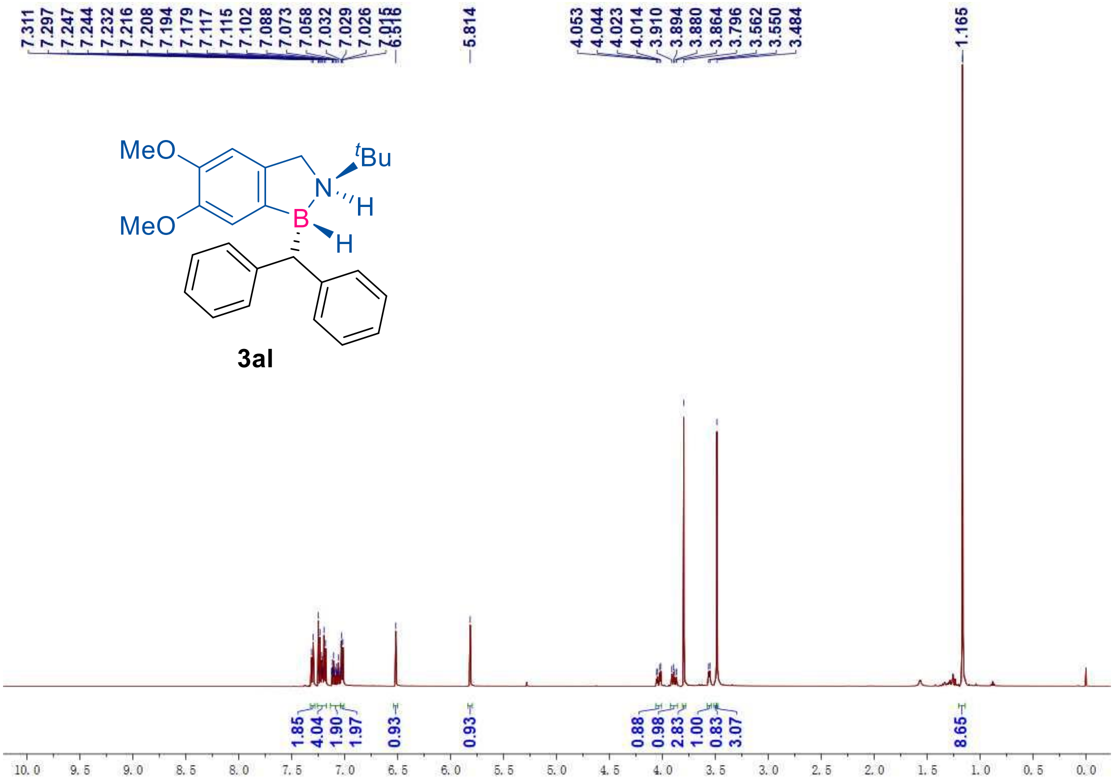

^{13}C NMR (126 MHz, $CDCl_3$)

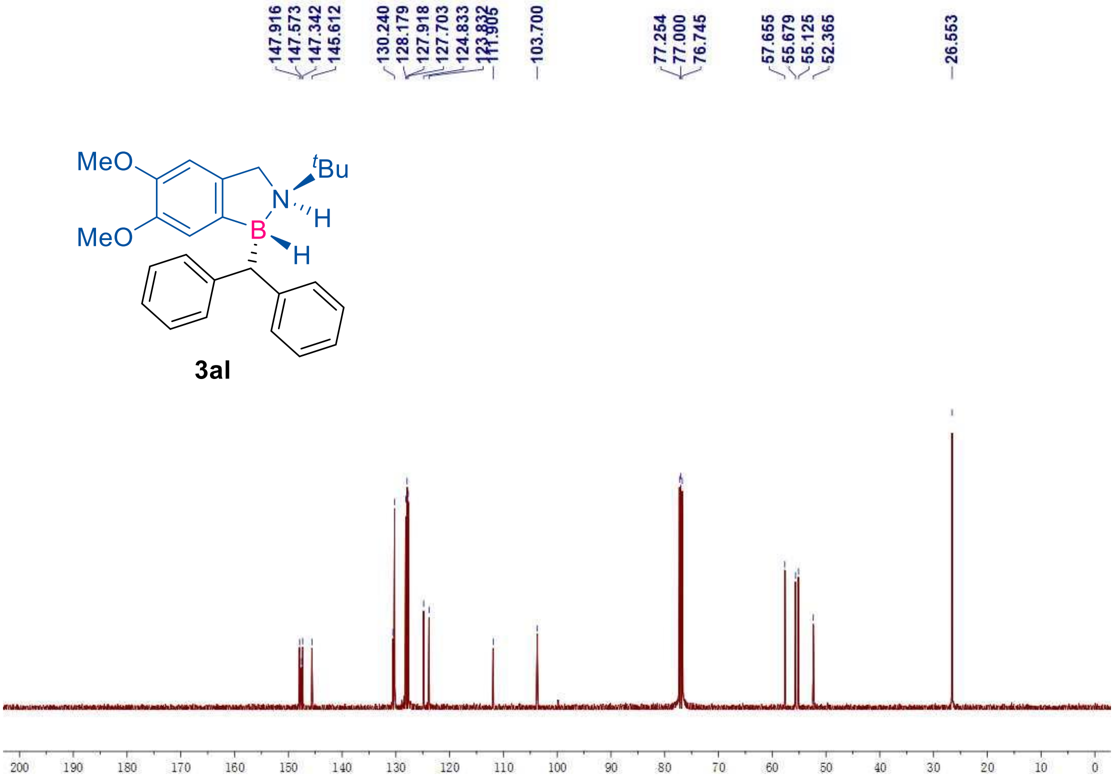

^{11}**B NMR** (160 MHz, $CDCl_3$)

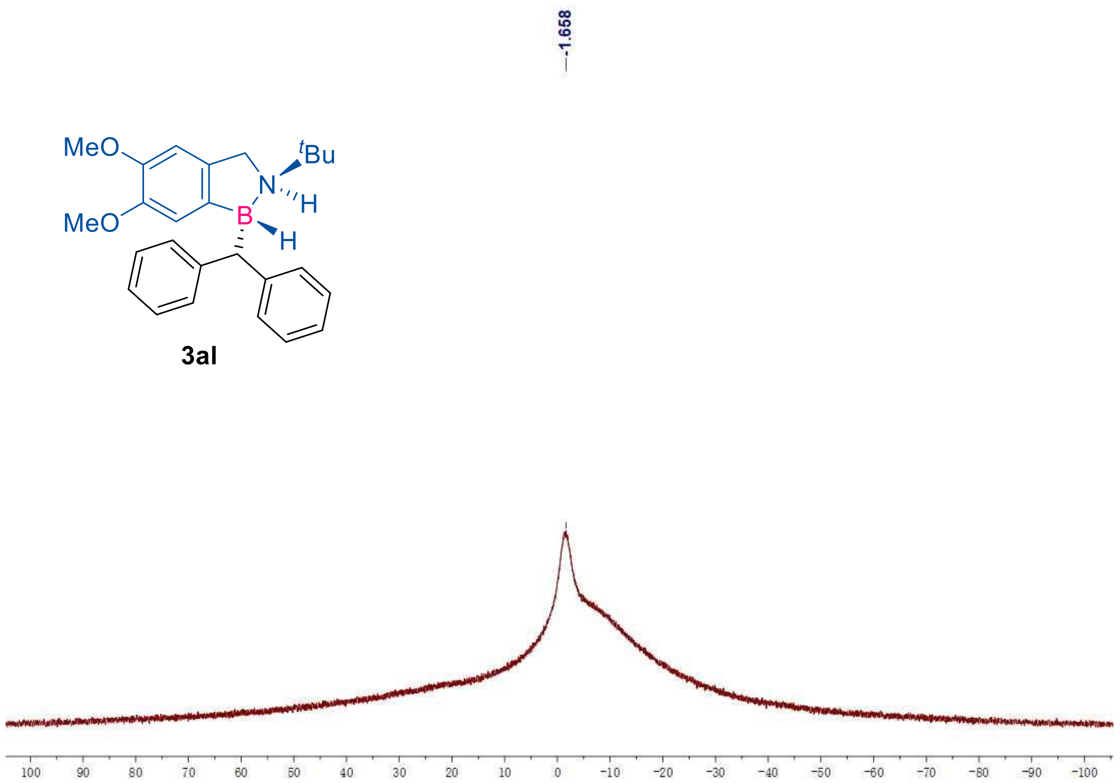

1-benzhydryl-2-(tert-butyl)-6-methoxy-2,3-dihydro-1H-benzo[c][1,2]azaborole (**3am**)

^{1}**H NMR** (500 MHz, Acetone-d_6)

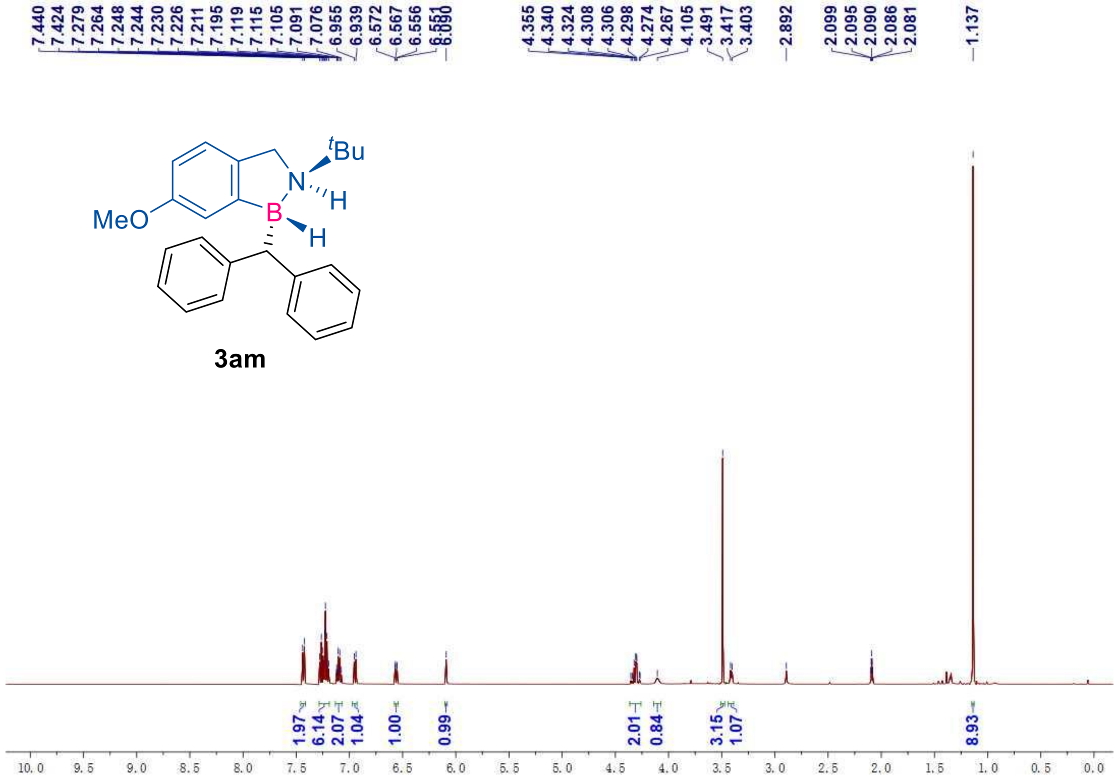

^{13}C NMR (126 MHz, Acetone-d_6)

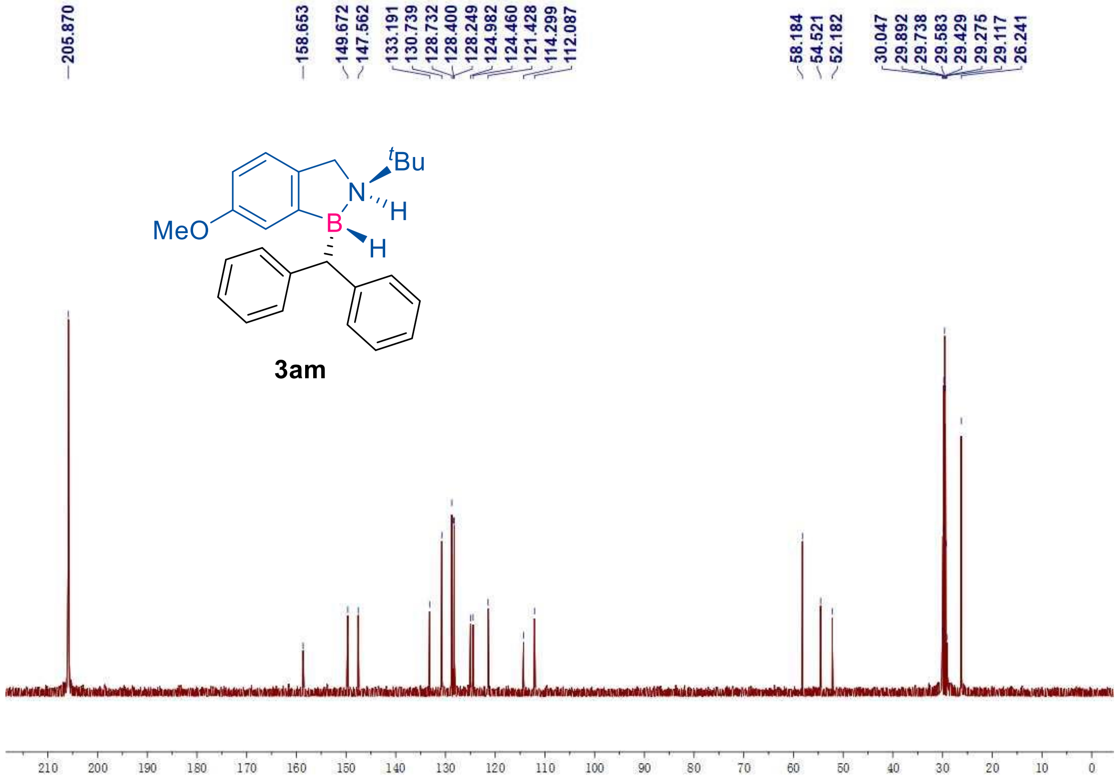

^{11}B NMR (128 MHz, Acetone-d_6)

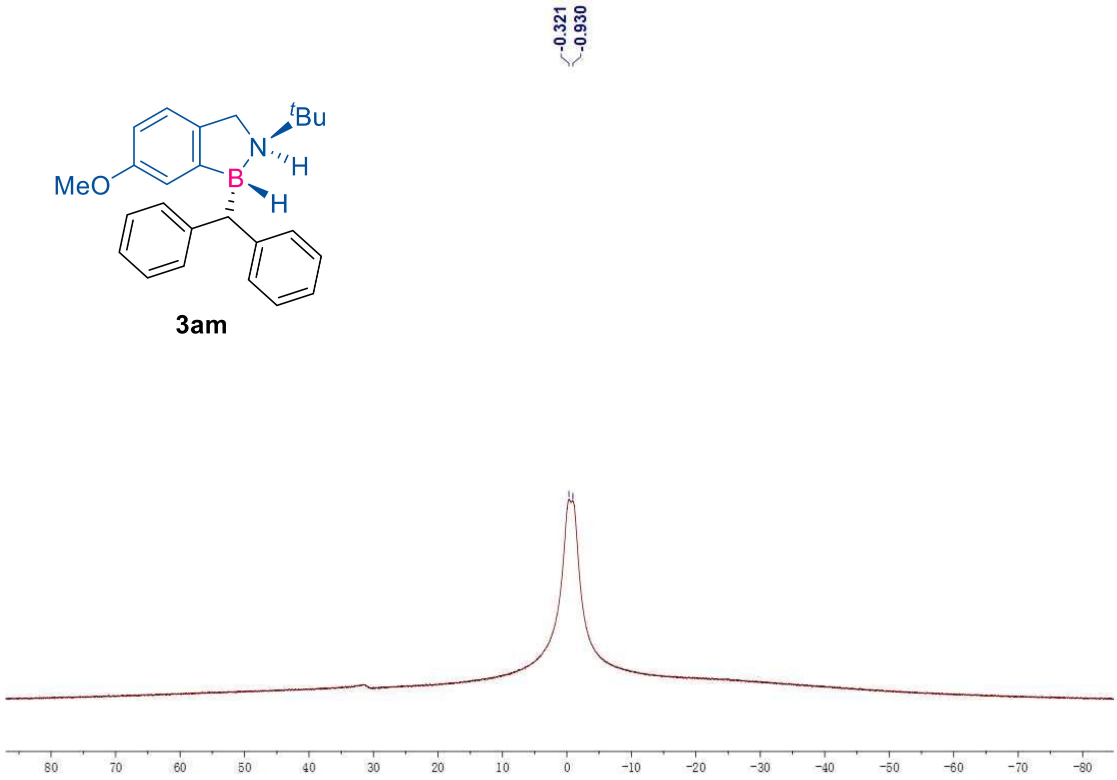

1-benzhydryl-2-(tert-butyl)-5-fluoro-2,3-dihydro-1H-benzo[c][1,2]azaborole (3an)

^{1}H NMR (500 MHz, $CDCl_3$)

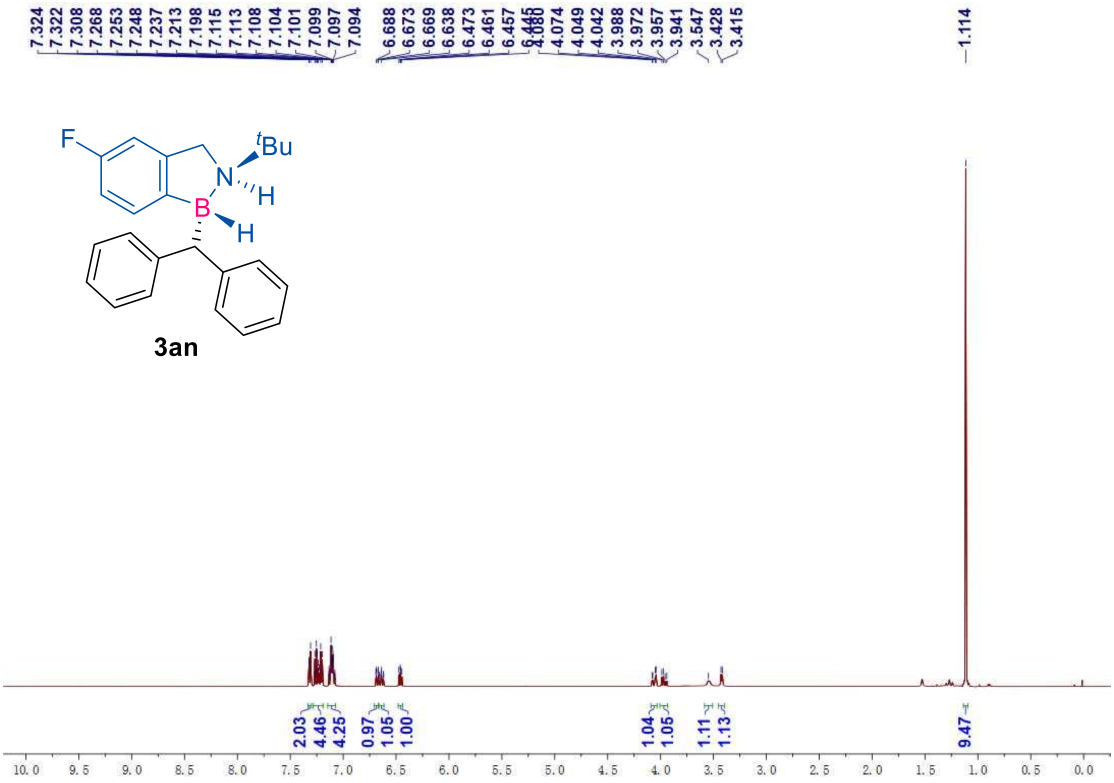

^{13}C NMR (126 MHz, $CDCl_3$)

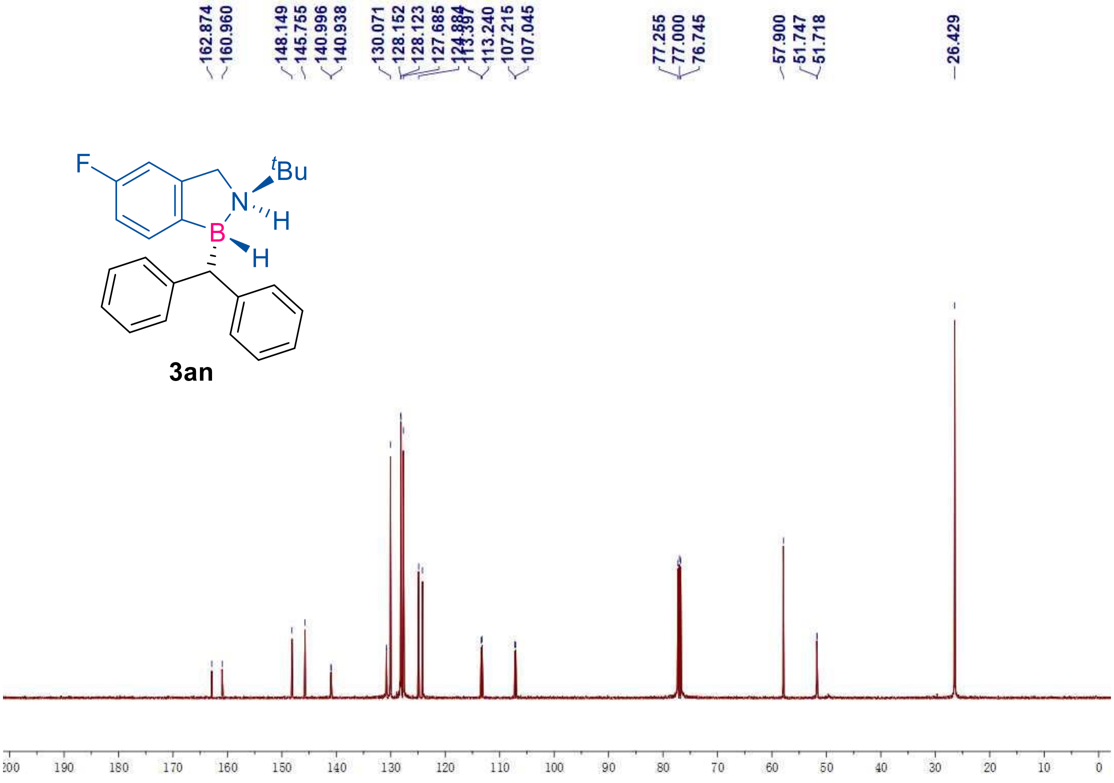

^{19}F NMR (471 MHz, CDCl$_3$)

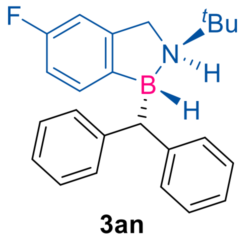

3an

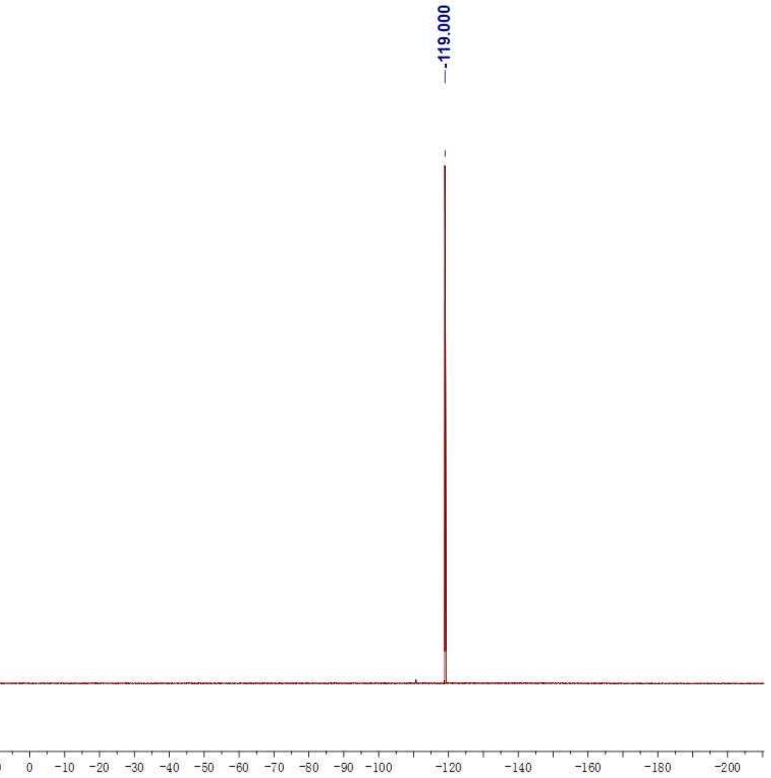

^{11}B NMR (160 MHz, CDCl$_3$)

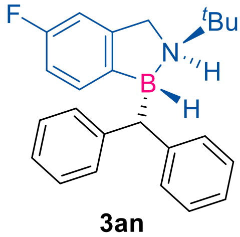

3an

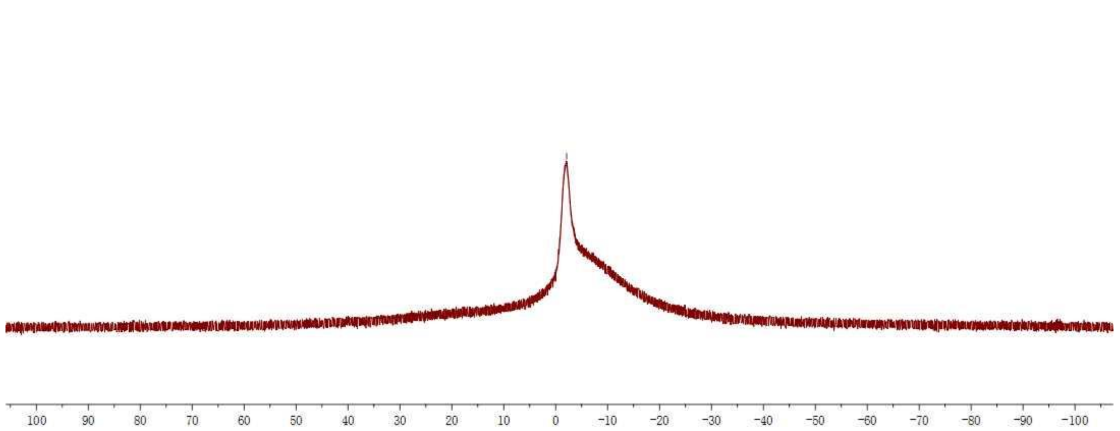

1-benzhydryl-2-benzyl-2-methyl-2,3-dihydro-1H-2l4-benzo[c][1,2]azaborole (3ao)

^{1}H NMR (500 MHz, $CDCl_3$)

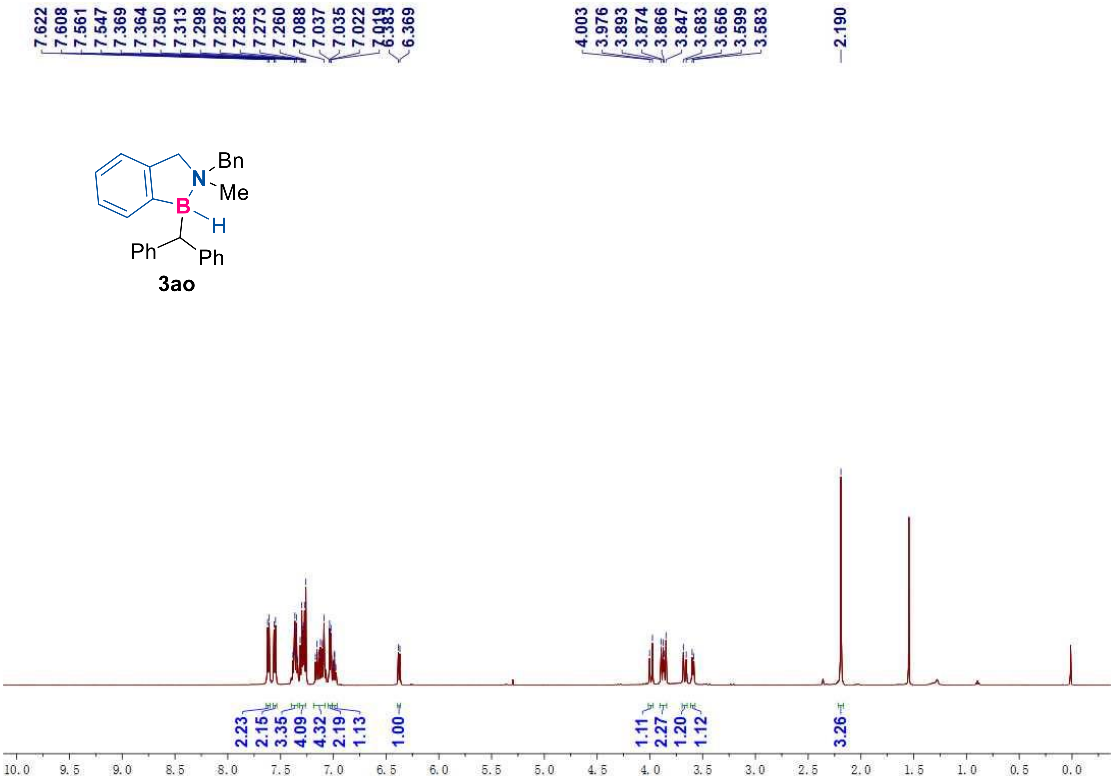

^{13}C NMR (126 MHz, $CDCl_3$)

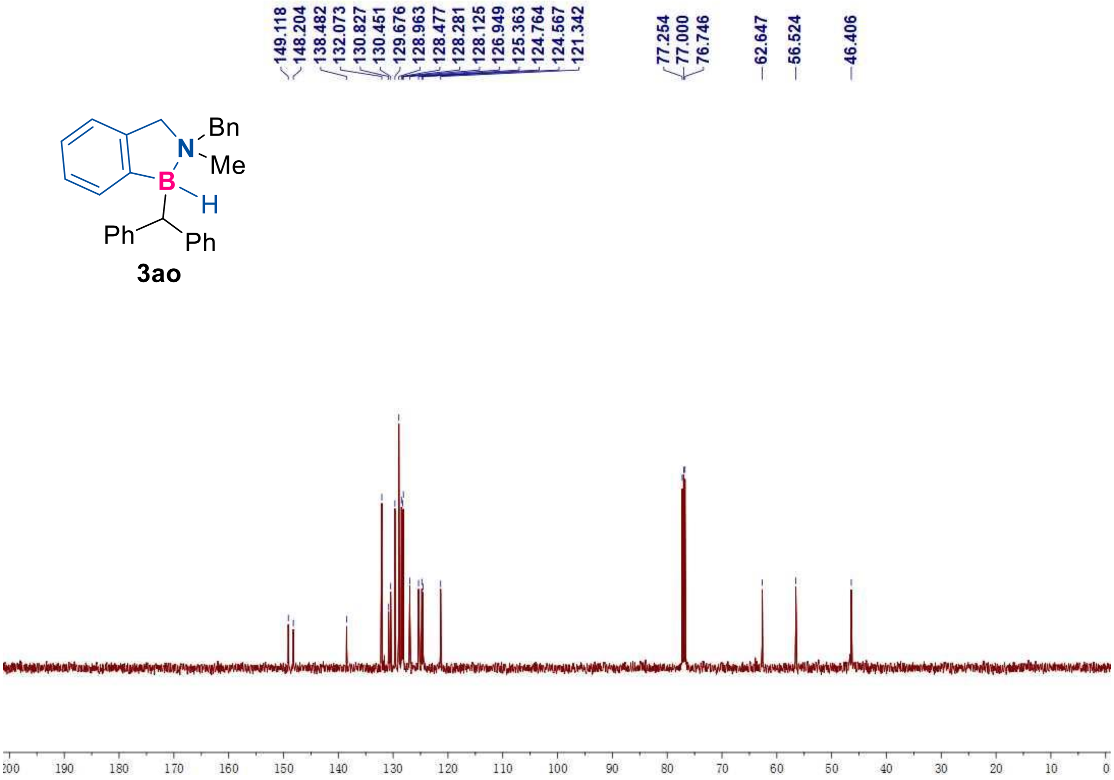

^{11}B NMR (160 MHz, $CDCl_3$)

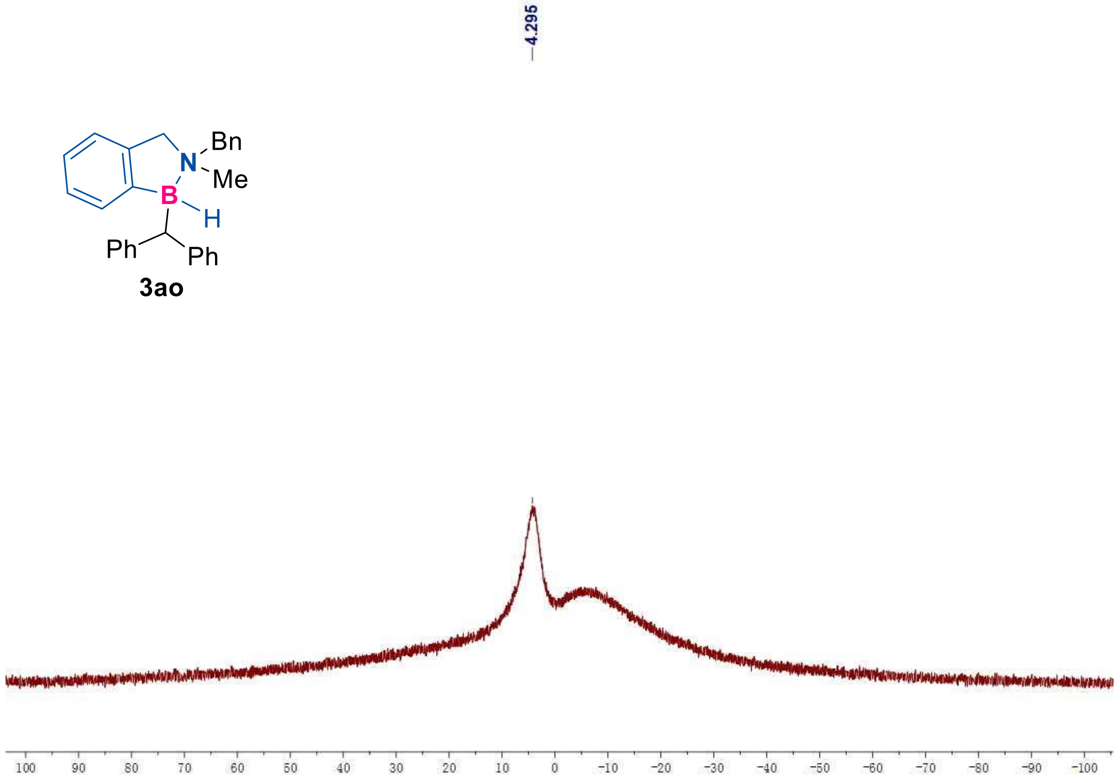

1-(5-((2-(tert-butyl)-2,3-dihydro-1H-benzo[c][1,2]azaborol-1-yl)(phenyl)methyl)-2-methylfuran-3-yl)ethan-1-ol (3ap)

^{1}H NMR (500 MHz, $CDCl_3$)

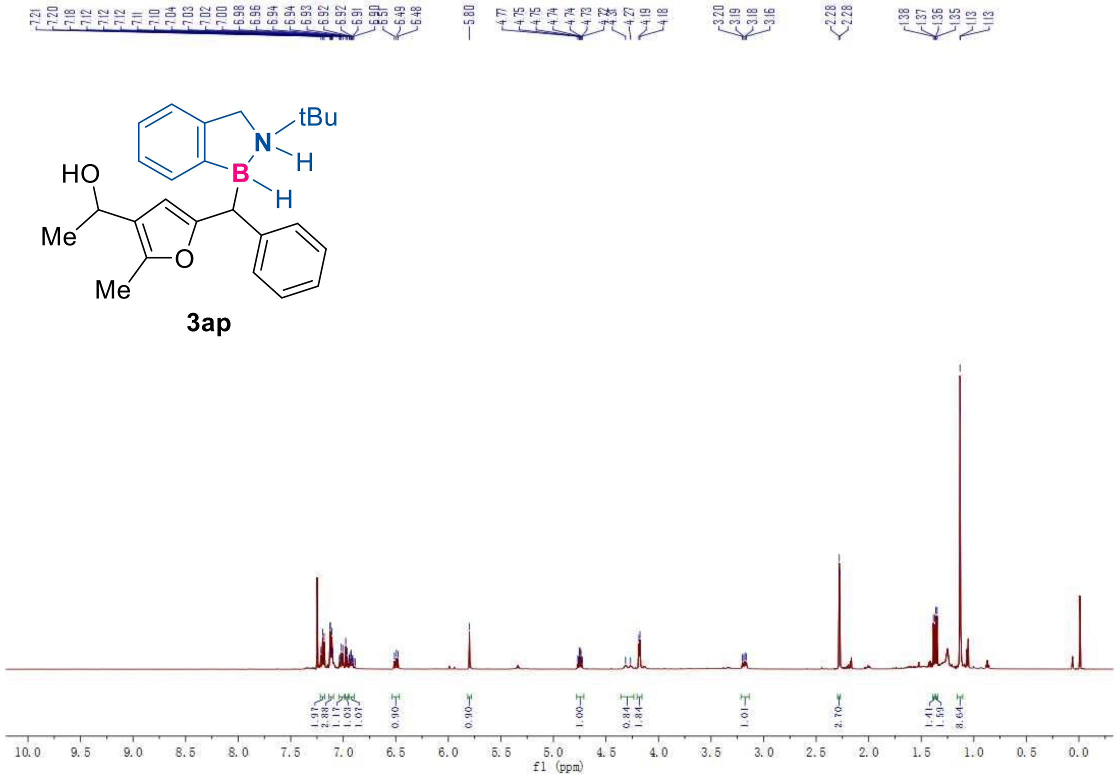

^{13}C NMR (126 MHz, $CDCl_3$)

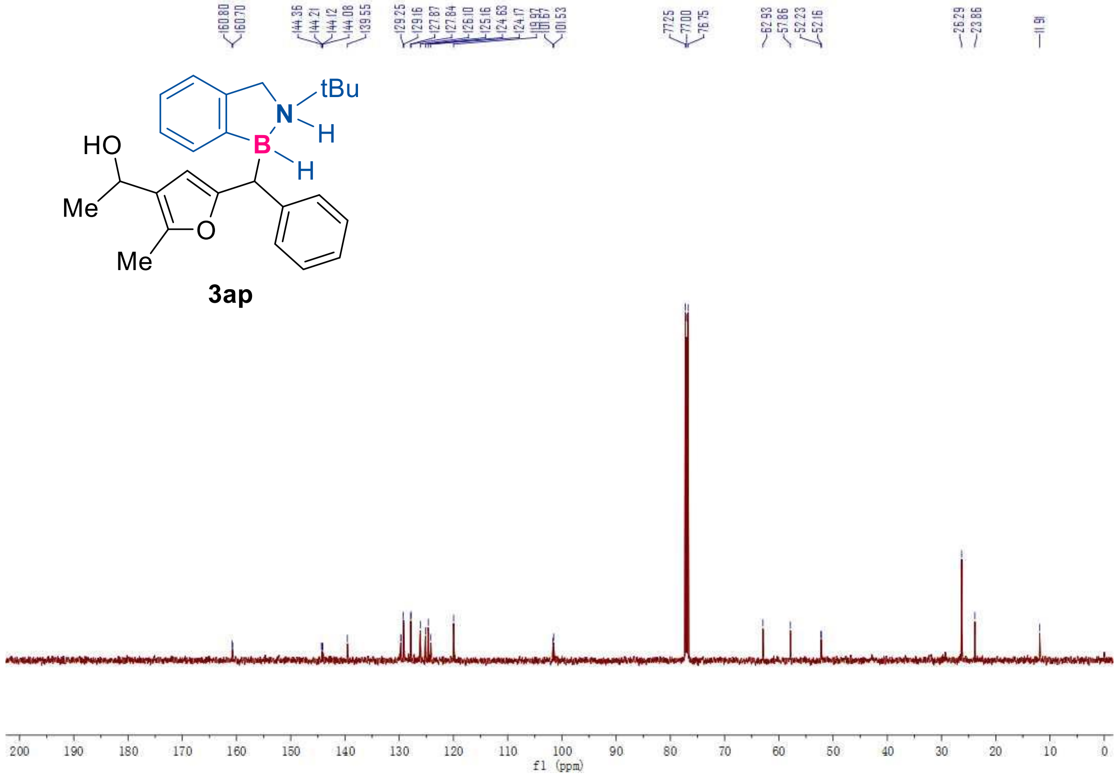

^{11}B NMR (160 MHz, $CDCl_3$)

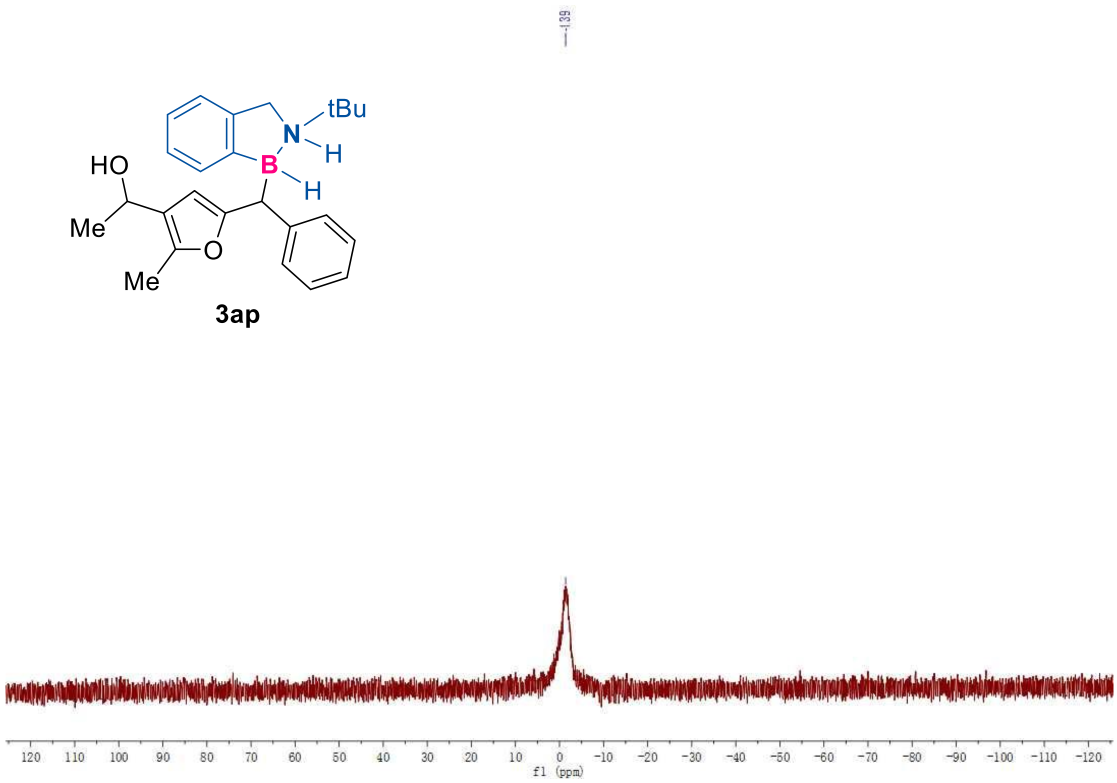

(1R)-2-(tert-butyl)-1-(diphenylmethyl-d)-2,3-dihydro-1H-1λ⁴-benzo[c][1,2]azaborole-1,3-d₂ (3a-*d₃*)

^{1}H NMR (500 MHz, CDCl$_3$)

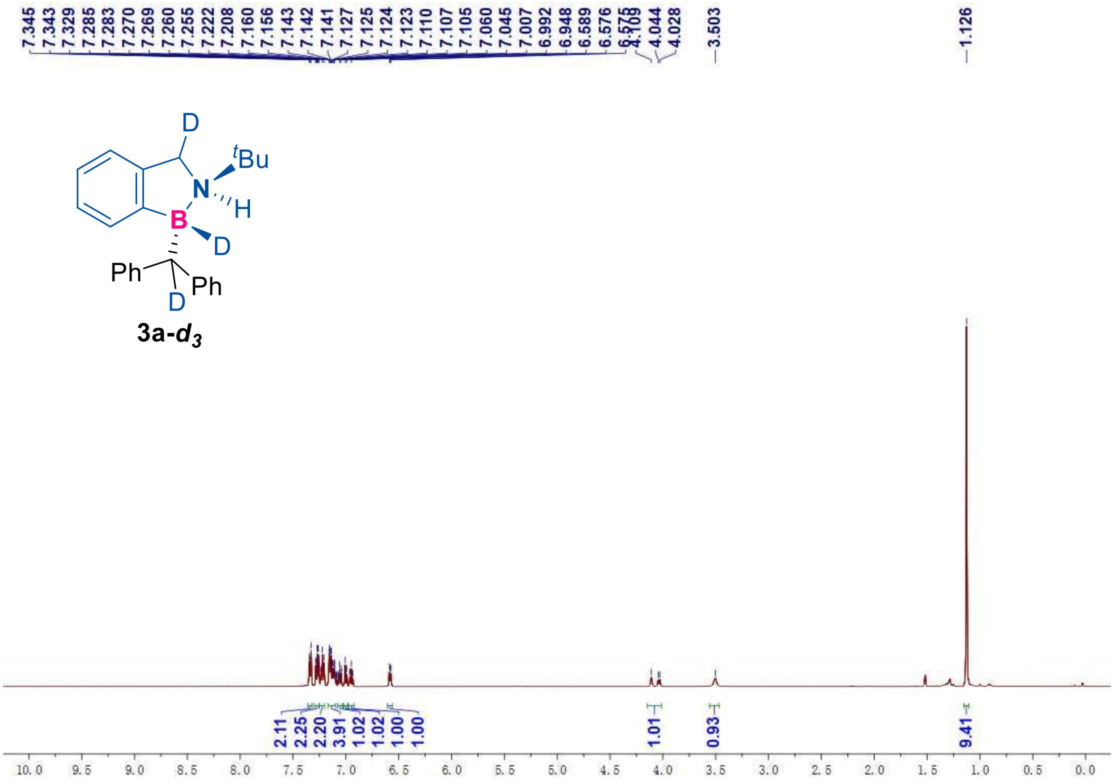

^{13}C NMR (126 MHz, CDCl$_3$)

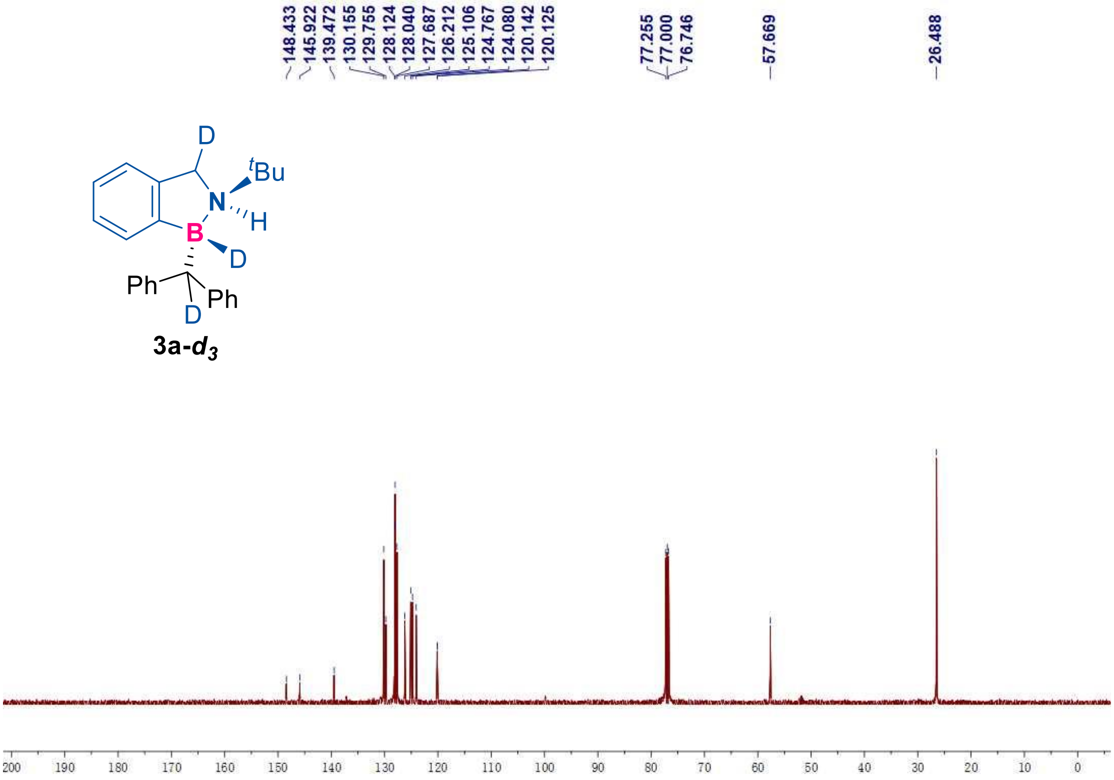

^{11}B NMR (160 MHz, CDCl$_3$)

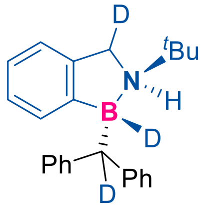

3a-*d*$_3$

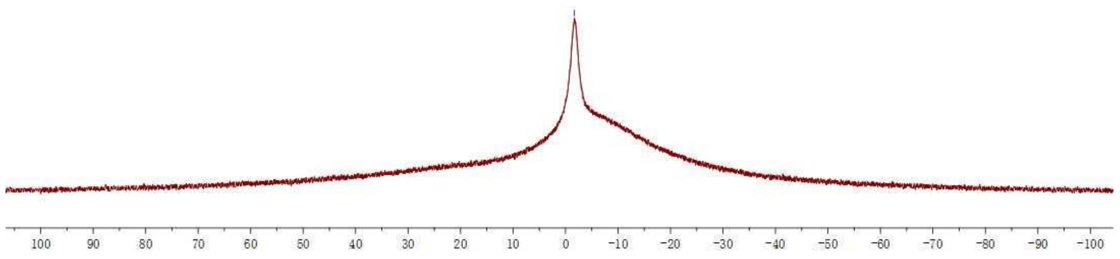

(S)-1-(naphthalen-2-yl)ethan-1-ol (5a')

^{1}H NMR (500 MHz, CDCl$_3$)

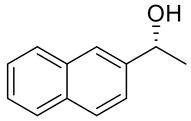

5a'

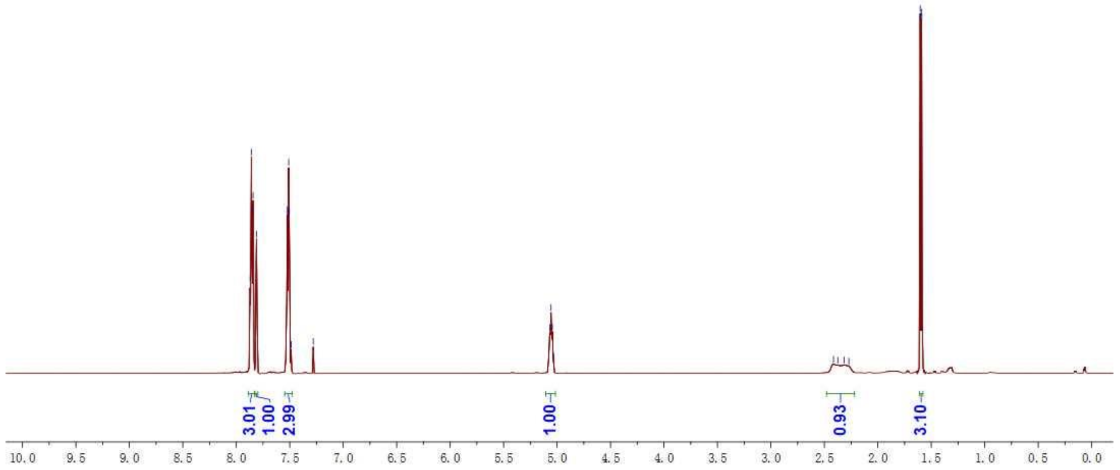

^{13}C NMR (126 MHz, CDCl$_3$)

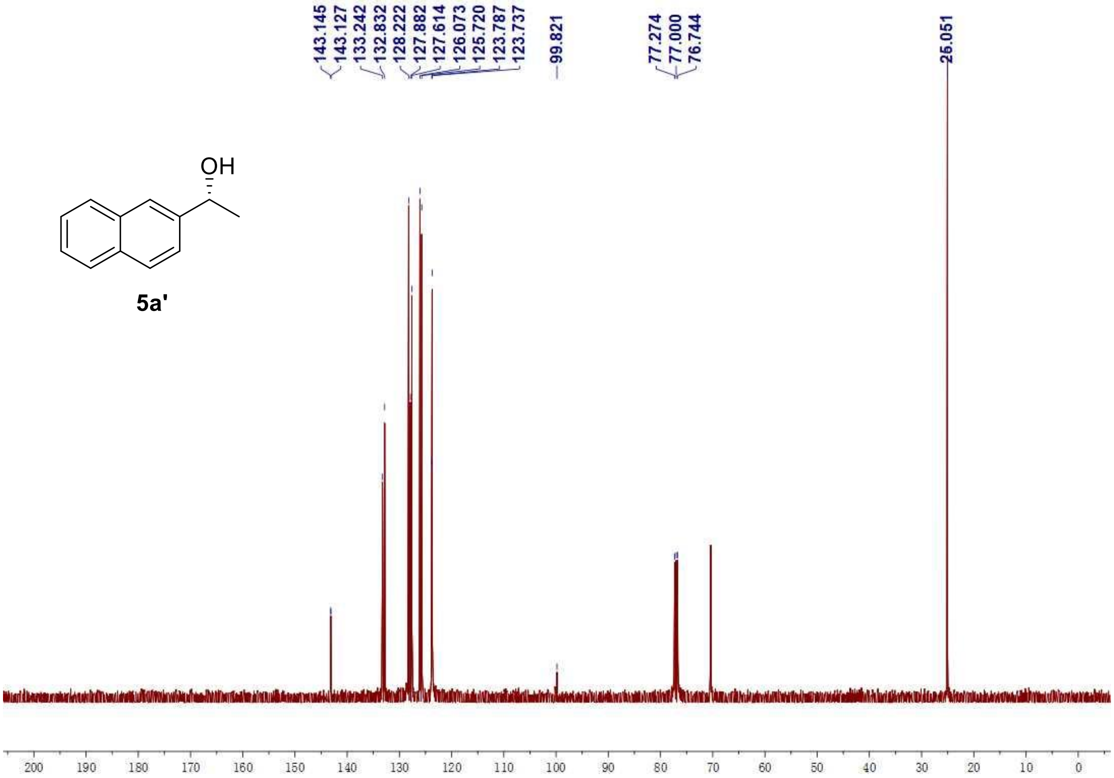

(S)-3-methyl-2,3-dihydrobenzo[d]isothiazole 1,1-dioxide (5b')

^{1}H NMR (500 MHz, CDCl$_3$)

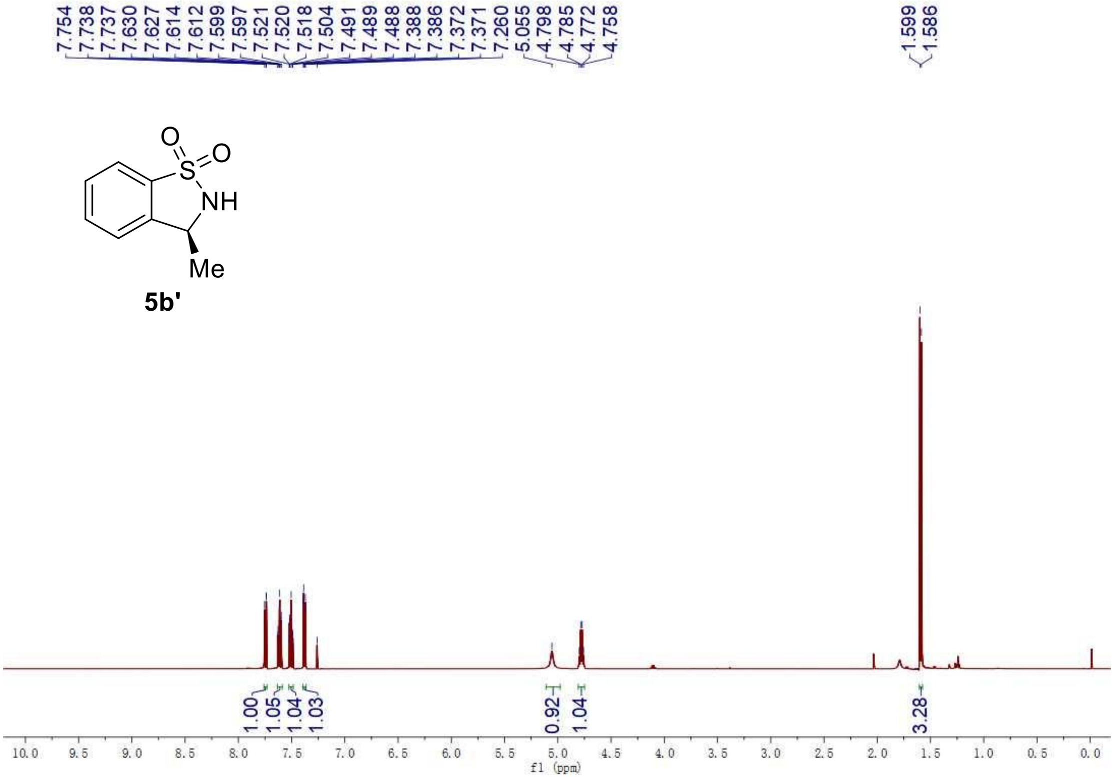

(R)-2-methyl-N-(1-phenylethyl)propane-2-sulfonamide (5c')

^{1}H NMR (500 MHz, $CDCl_3$)

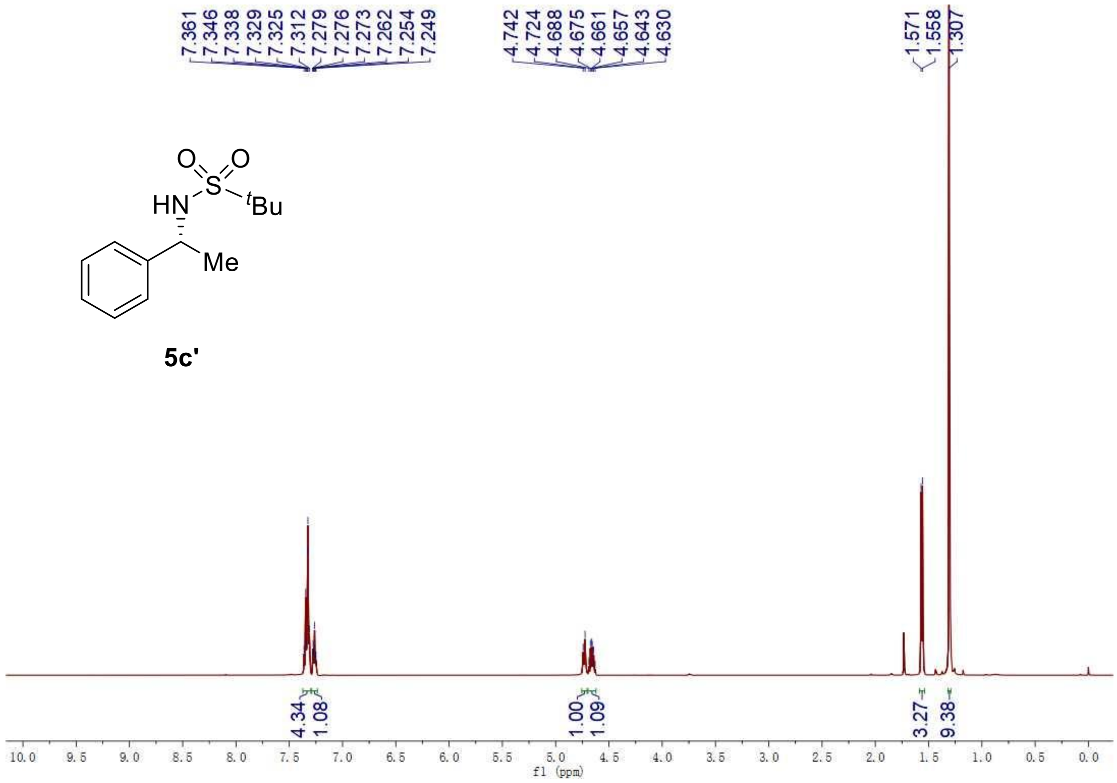

(S)-2-(tert-butyl)-1-((4'-methoxy-[1,1'-biphenyl]-4-yl)(4-methoxyphenyl)methyl)-2,3-dihydro-1H-benzo[c][1,2]azaborole (6)

^{1}H NMR (500 MHz, $CDCl_3$)

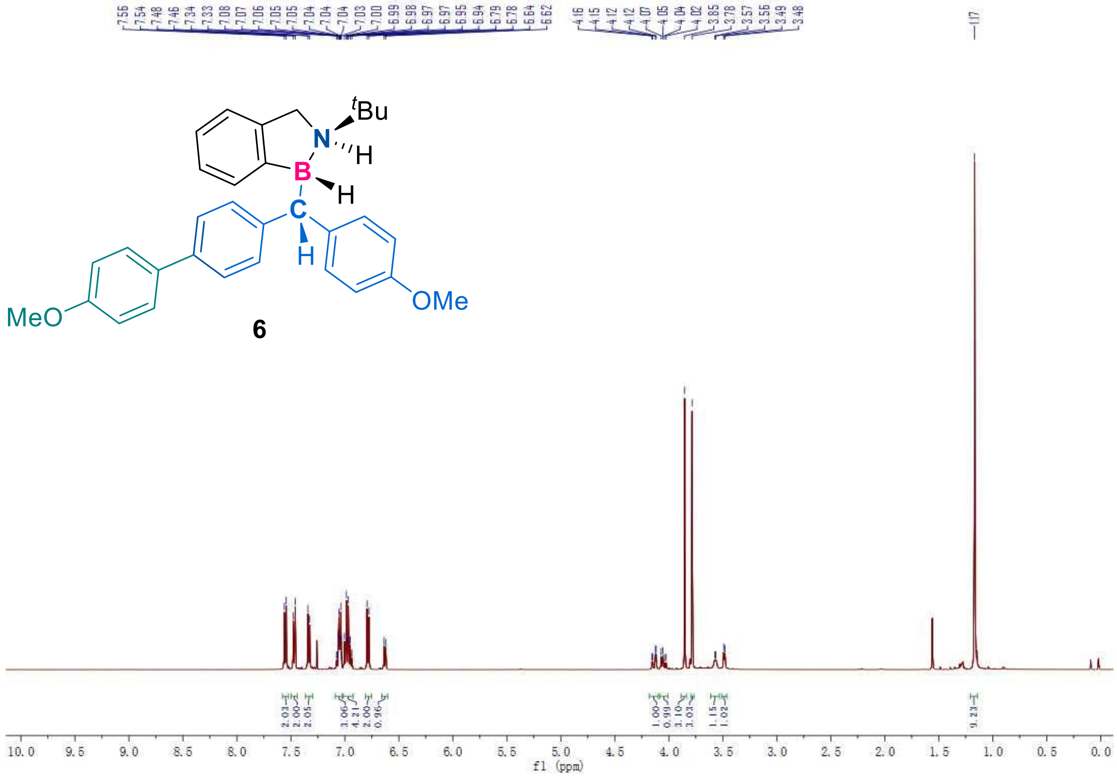

^{13}**C NMR** (126 MHz, CDCl$_3$)

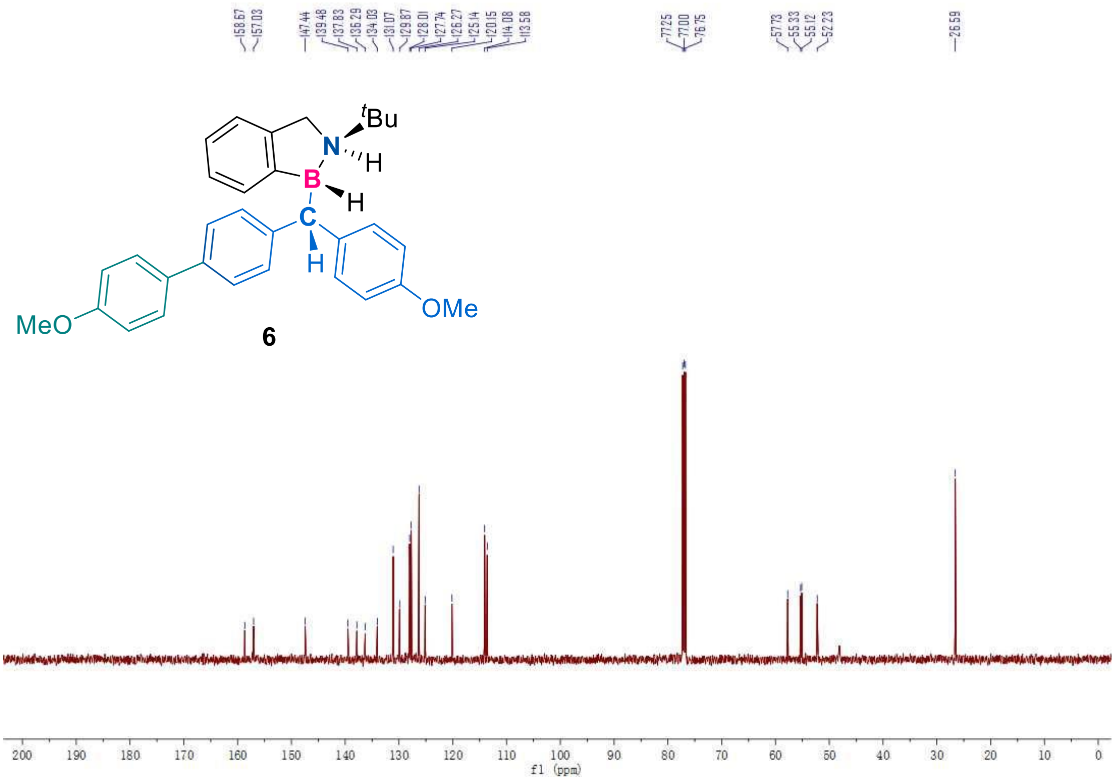

^{11}**B NMR** (160 MHz, CDCl$_3$)

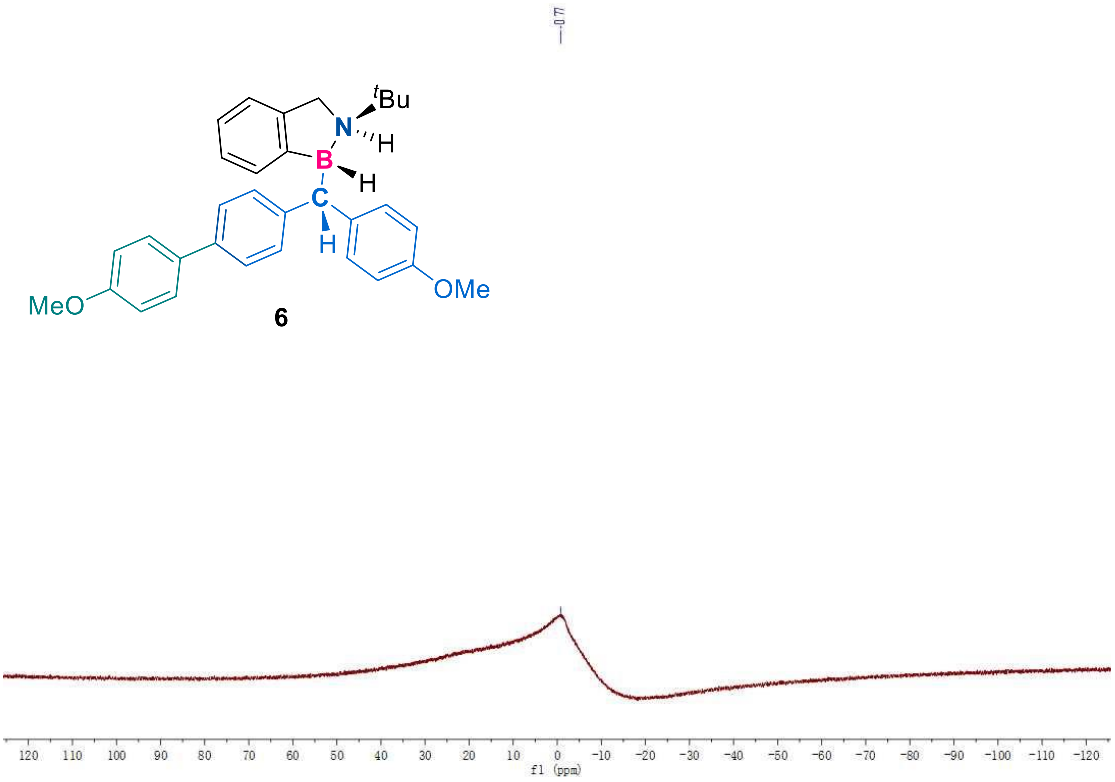

(S)-2-(tert-butyl)-1-((4-(4-methoxybenzyl)phenyl)(4-methoxyphenyl)methyl)-2,3-dihydro-1H-

benzo[c][1,2]azaborole (7)

^{1}H NMR (500 MHz, $CDCl_3$)

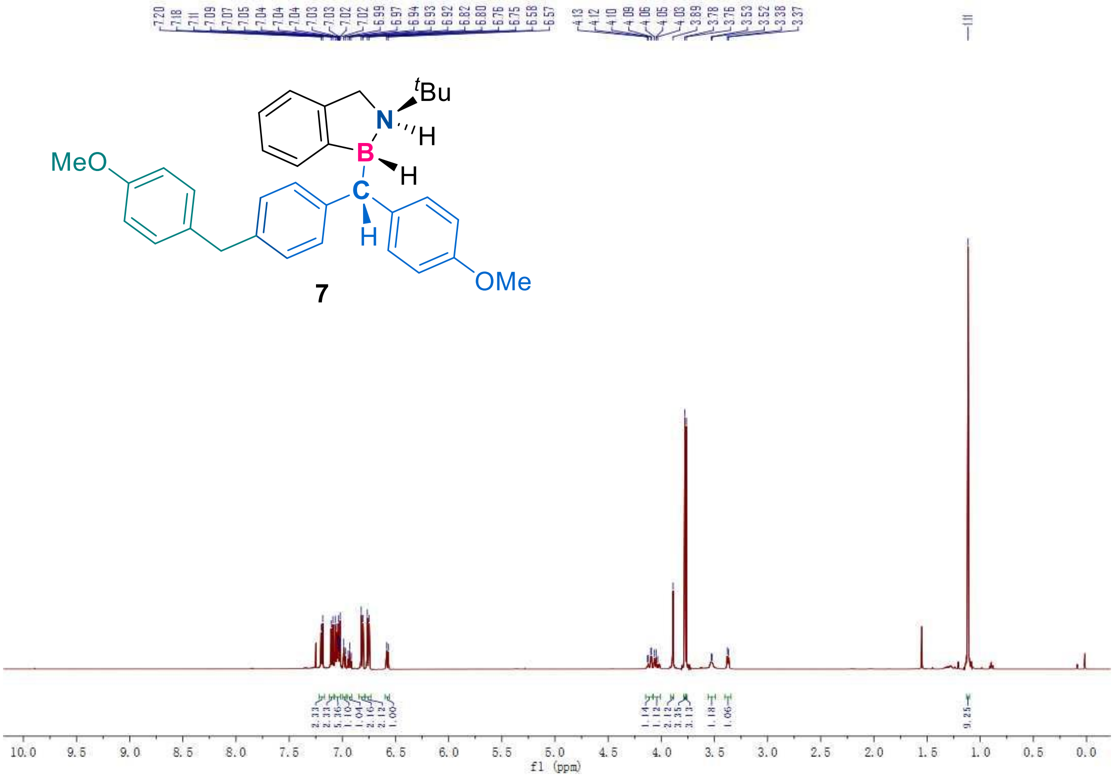

^{13}C NMR (126 MHz, $CDCl_3$)

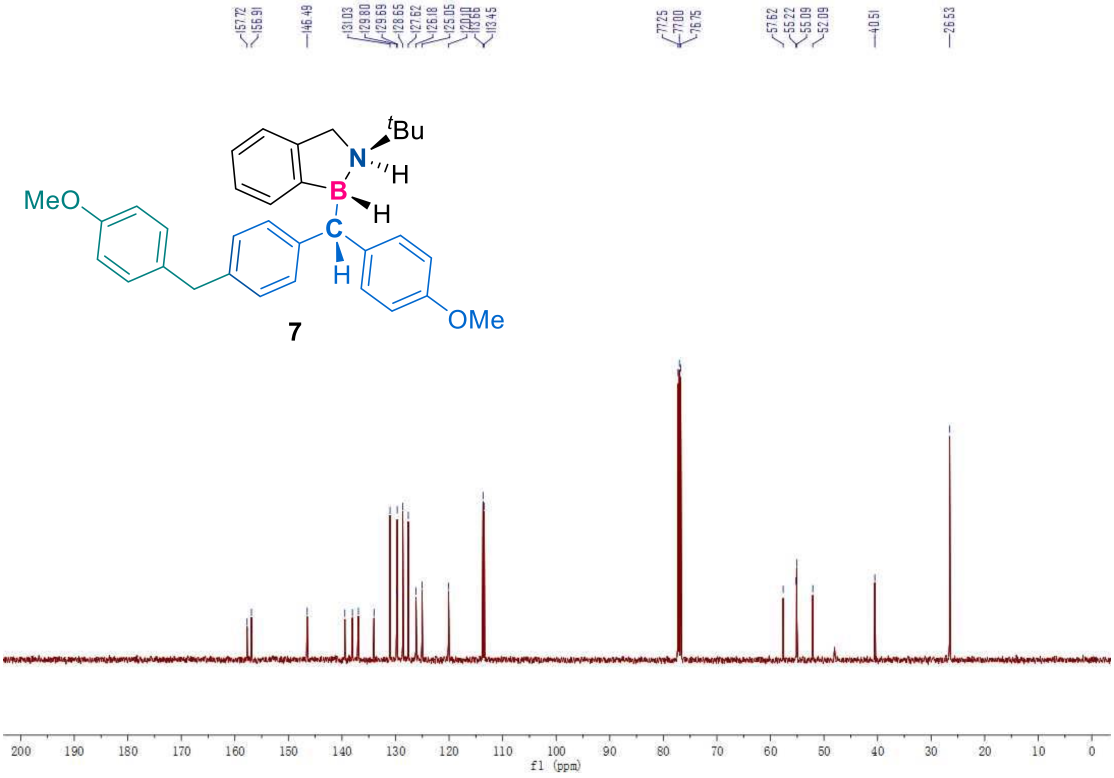

^{11}B NMR (160 MHz, $CDCl_3$)

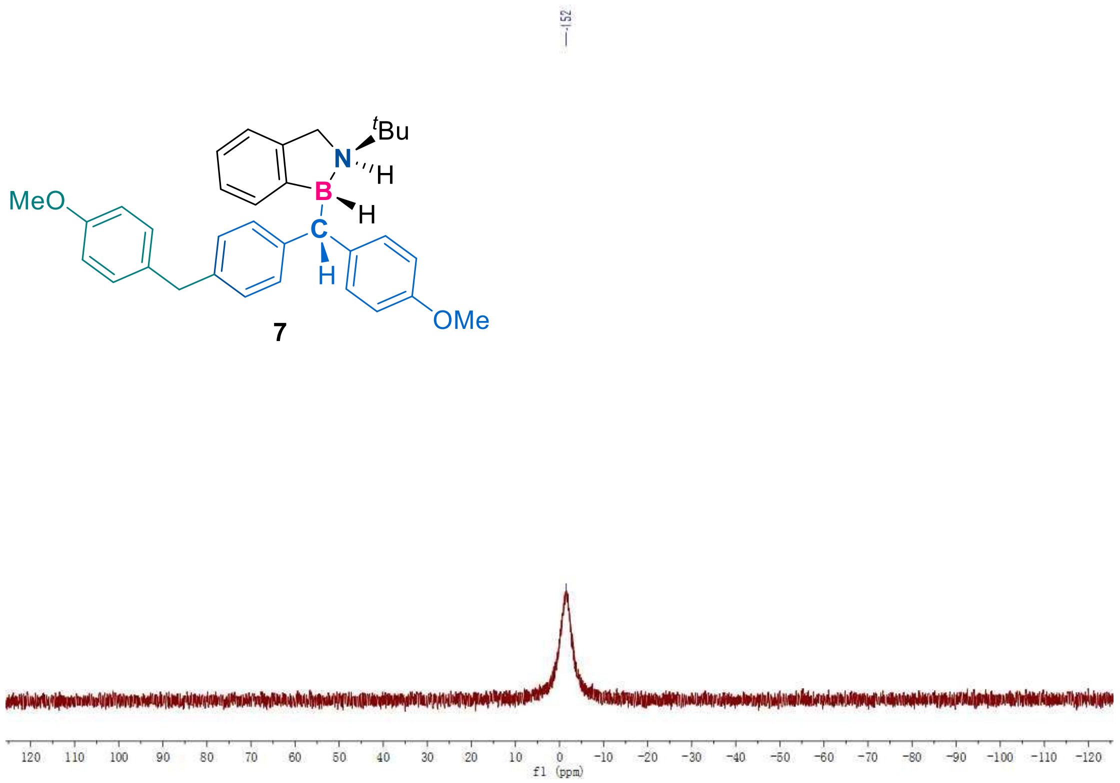

9 Supplementary references

1. G. Zhang, Z. Zhang, M. Hou, X. Cai, K. Yang, P. Yu, Q. Song, *Nat. Commun.* **2022**, 13, 2624.
2. Y. Zhao, Y. Su, X. Li, L. Yang, M. Huang, S. Zhu, *Angew. Chem. Int. Ed.* **2021**, *60*, 24214–24219..
3. Y. Pang, Q. He, Z.-Q. Li, J.-M. Yang, J.-H. Yu, S.-F. Zhu, Q.-L. Zhou, *J. Am. Chem. Soc.* **2018**, *140*, 10663–10668.
4. B. Li, J. Chen, Z. Zhang, I. D. Gridnev, W. Zhang. *Angew.Chem. Int.Ed.* **2019**, *58*,7329–7334.
5. M. J. Frisch, et al. Gaussian 16 Rev. C.01 (Wallingford, CT, 2016).
6. Becke, A. D. *J. Chem. Phys.* **1993**, *98*, 5648–5652.
7. C. Lee, W. Yang, R. G. Parr, *Phys. Rev. B.* **1988**, 37, 785–789.
8. F. Weigend, Ahlrichs, R. *Phys. Chem. Chem. Phys.* **2005**, 7, 3297-3305.
9. R. Ditchfield, W. J. Hehre, J. A. Pople, *J. Chem. Phys.* **1971**, *54*, 724-728.
10. R. F. Ribeiro, A. V. Marenich, C. J. Cramer, D. G. Truhlar, *J. Phys. Chem. B* **2011**, *115*, 14556-14562.
11. J.-D. Chai, M. Head-Gordon, *Phys. Chem. Chem. Phys.* **2008**,10, 6615-6620.
12. F. Weigend, R. Ahlrichs, *Phys. Chem. Chem. Phys.* **2005**, 7, 3297-3305.
13. S. Grimme, *J. Chem. Theory Comput.* **2019**, *15*, 2847–2862.
14. P. Pracht, F. Bohle, S. Grimme, *Phys. Chem. Chem. Phys.* **2020**, *22*, 7169–7192.

15. T. Lu, F. Chen, *J. Comput. Chem.* **2012**, *33*, 580-592.

16. CYLview, 1.0b. Legault, C. Y., Université de Sherbrooke, 2009 (http://www.cylview.org).
